# Identifying English Practices that Are High Antibiotic Prescribers Accounting for Comorbidities and Other Legitimate Medical Reasons for Variation

**DOI:** 10.1016/j.eclinm.2018.12.003

**Published:** 2018-12-12

**Authors:** Emma C. Hope, Ron E. Crump, T. Deirdre Hollingsworth, Timo Smieszek, Julie V. Robotham, Koen B. Pouwels

**Affiliations:** a Zeeman Institute, Mathematics Institute, University of Warwick, Coventry CV4 7AL, UK.; b Zeeman Institute, School of Life Sciences, University of Warwick, Coventry CV4 7AL, UK.; c Big Data Institute, Li Ka Shing Centre for Health Information and Discovery, University of Oxford, Oxford, OX3 7LF, UK; d Modelling and Economics Unit, National Infection Service, Public Health England, London NW9 5EQ, UK.; e MRC Centre for Outbreak Analysis and Modelling, Department of Infectious Disease Epidemiology, Imperial College School of Public Health, London W2 1PG, UK.; f Department of Health Sciences, University of Groningen, University Medical Center Groningen, 9713, GZ, Groningen, Netherlands

**Keywords:** Anti-bacterial agents, General practice, Electronic health records, Epidemiology, Inappropriate prescribing

## Abstract

**Background:**

Seeing one's practice as a high antibiotic prescriber compared to general practices with similar patient populations can be one of the best motivators for change. Current comparisons are based on age-sex weighting of the practice population for expected prescribing rates (STAR-PU). Here, we investigate whether there is a need to additionally account for further potentially legitimate medical reasons for higher antibiotic prescribing.

**Methods:**

Publicly available data from 7376 general practices in England between April 2014 and March 2015 were used. We built two different negative binomial regression models to compare observed versus expected antibiotic dispensing levels per practice: one including comorbidities as covariates and another with the addition of smoking prevalence and deprivation. We compared the ranking of practices in terms of items prescribed per STAR-PU according to i) conventional STAR-PU methodology, ii) observed vs expected prescribing levels using the comorbidity model, and iii) observed vs expected prescribing levels using the full model.

**Findings:**

The median number of antibiotic items prescribed per practice per STAR-PU was 1.09 (25th–75th percentile, 0.92–1.25). 1133 practices (76.8% of 1476) were consistently identified as being in the top 20% of high antibiotic prescribers. However, some practices that would be classified as high prescribers using the current STAR-PU methodology would not be classified as high prescribers if comorbidity was accounted for (n = 269, 18.2%) and if additionally smoking prevalence and deprivation were accounted for (n = 312, 21.1%).

**Interpretation:**

Current age-sex weighted comparisons of antibiotic prescribing rates in England are fair for many, but not all practices. This new metric that accounts for legitimate medical reasons for higher antibiotic prescribing may have more credibility among general practitioners and, thus, more likely to be acted upon.

**Outstanding Questions:**

Findings of this study indicate that the antibiotic prescribing metric by which practices are measured (and need to implement interventions determined) may be inadequate, and therefore raises the question of how they should be measured. Substantial variation between practices remains after accounting for comorbidities, deprivation and smoking. There is a need for a better understanding of why such variation remains and, more importantly, what can be done to reduce it. While antibiotics are more frequently indicated in patients with comorbidities, it is unclear to what extent antibiotic prescribing can be lowered among that patient population and how this could be achieved.

## Highlights

• Antibiotic prescribing rates vary substantially between general practices in England
• Some practices that are identified as high antibiotic prescribers would not be identified as such if legitimate reasons for variation, such as comorbidities, were taken into account.
• Substantial variation remains after accounting for comorbidities, smoking and deprivation.

**Research in Context**

**Evidence Before This Study**

We searched PubMed for studies evaluating variation in antibiotic prescribing in primary care. The papers included in the review were original research studies of any design in which the criteria for inclusion were: written in English; set in high-income countries; evaluating variation in antibiotic prescribing between general practices or benchmarking these practices based on their antibiotic prescribing rates. We used the following MeSH terms: “benchmarking” or “practice patterns physicians” or “small-area analysis” and “antibacterial agents” and any of “primary health care” or “general practice”. The overall picture was that non-medical reasons were explaining a substantial part of the variation in antibiotic prescribing. Identified factors included experience of physicians, the patient volume per physician, physicians being trained abroad, lack of access to diagnostic tests, patient expectations, time pressure and poor doctor-patient communication. Evidence related to what extent potential legitimate medical reasons explain the variation in antibiotic prescribing between practices was limited. In England, comparisons between practices were based on prescriptions per practice population or using Specific Therapeutic group Age-sex weight Related Prescribing Units (STAR-PUs) to account for differing age-sex distributions of patient populations.

**Added Value of This Study**

This study found that current STAR-PU based comparisons of antibiotic prescribing rates in England are fair for many, but not all practices. Some practices can legitimately claim that they have a frailer patient population (e.g., relatively high prevalence of chronic obstructive pulmonary disease, asthma and diabetes) and prescribe, in line with guidelines in England, more antibiotics than other practices. Our estimates allow for comparing one's individual practice with other practices with similar patient populations in terms of comorbidity prevalences, or also in terms of smoking prevalence and deprivation.

**Implications of All the Available Evidence**

Antibiotic prescribing rates vary substantially between general practices in England, even after taking into account potential legitimate medical reasons for higher prescribing. Our newly developed metric that accounts for legitimate medical reasons for higher antibiotic prescribing rates may have higher credibility among general practitioners and, thus, potentially more likely to be acted upon, helping endeavours to reduce inappropriate antimicrobial use in English primary care.

Alt-text: Unlabelled Box

## 1 Introduction

Antibiotic resistance (ABR) is increasingly recognised as an important threat to modern healthcare [1]. There is strong evidence that antibiotic use is one of the major drivers of ABR [2]. In many countries inappropriate antibiotic prescribing makes up a substantial fraction of the total prescribing levels, thereby unnecessary increasing ABR levels [3], [4], [5]. The substantial variation that is observed between different general practices and countries [3], [6] may be interpreted as further evidence that there is substantial overprescribing in certain practices and areas.

Implementation of interventions designed to lower unnecessary antibiotic prescribing in primary care are often prioritised in general practices that prescribe above the norm [7]. Prescribing rates between practices in England are often compared using Specific Therapeutic Group Age-sex weighting Related Prescribing Units (STAR-PU) weightings to take into account that specific age- and gender-groups legitimately receive more antibiotic prescriptions than other groups [8]. Instead of using the unweighted number of registered patients as the denominator for the antibiotic prescribing rate, the STAR-PU method gives different weights to registered patients dependent on their age and sex. For example, females aged between 65 and 74 get a weight of 1.0, while males aged between 25 and 34 get a weight of 0.2 (Table 1). This methodology has been developed to enable more fair and meaningful comparisons between general practices. Antibiotic prescribing guidelines indicate that antibiotic prescriptions are more frequently appropriate among very young children or elderly people [9]. In line with this, antibiotic overprescribing for respiratory tract indications has been found to be highest among patients between 18 and 65 years of age [10]. Accounting for sex may also be important because females are biologically more prone to acquiring urinary tract infections (UTIs) [11].

**Table 1:** Oral antibacterial item based STAR-PU weights.

Age band (y)	Male	Female
0–4	0.8	0.8
5–14	0.3	0.4
15–24	0.3	0.6
25–34	0.2	0.6
35–44	0.3	0.6
45–54	0.3	0.6
55–64	0.4	0.7
65–74	0.7	1.0
75 +	1.0	1.3

STAR-PU = Specific Therapeutic Group Age-sex weighting Related Prescribing Unit.

However, STAR-PU weighting does not account for several other legitimate reasons for variation in antibiotic prescribing. It has previously been shown that variation in prescribing for respiratory tract infections (RTIs) is the main driver of differences in antibiotic prescribing rates [3], [4], [6]. English guidelines indicate that patients with RTIs at high risk of serious complications due to pre-existing comorbidity should be offered an immediate antibiotic prescription and/or further appropriate investigation and management [9], [12], [13]. Hence, practices with a relatively high proportion of patients with comorbidities could legitimately prescribe more than a practice with relatively healthy patients. Accounting for differences in such comorbidities may be necessary for more fair comparisons of antibiotic prescribing rates of practices.

Ideally, one would be able to identify high prescribing practices after accounting for all legitimate reasons for variation in antibiotic prescribing. In this study we evaluate which practices in England prescribe above the norm using models that do and do not account for legitimate medical reasons for variation in antibiotic prescribing. This may help in reducing antibiotic prescribing, as communicating performance metrics (here, e.g., seeing one's practice as an outlier) can be one of the best motivators for change [14], especially if being an outlier cannot be explained by prevalence of comorbidities or other potential legitimate reasons for higher antibiotic prescribing rates. A method that accounts for comorbidities, smoking prevalence, and deprivation levels may be more believed by general practitioners and thus be acted upon.

## 2 Method

Publicly available systemic antibiotic (British National Formulary chapter 5.1) item-based dispensing data were obtained from NHS Digital for the financial year April 2014 – March 2015 (http://content.digital.nhs.uk). For each practice in England, antibiotics that are prescribed and subsequently dispensed in the community are included in this dataset. Other publicly available data that were obtained from NHS Digital included: the number of patients registered at each practice split into gender and five year age bands, comorbidity prevalences, and oral antibacterials items-based STAR-PU weights [8].

The prevalences of the following comorbidities were available via the Quality and Outcomes Framework (QOF) data and included: asthma (excluding patients who have not been prescribed asthma-related drugs in the preceding 12 months), chronic obstructive pulmonary disease (COPD), coronary heart disease, heart failure, cancer (excluding patients with non-melanotic skin cancers), chronic kidney disease (excluding patients with chronic kidney disease stage < 3), and diabetes. These comorbidities were selected as potential legitimate reasons for variation in antibiotic prescribing, because they are considered as factors that are associated with worse infection-related outcomes [13]. English guidelines recommend considering an immediate antibiotic prescription in patients with RTIs and these comorbidities [9], [12]. In contrast, in patients without such comorbidities immediate antibiotics are generally not recommended [12], [13].

Another legitimate cause of variation in antibiotic prescribing is the smoking prevalence of the patient population. Both active and passive smoking are associated with an increased risk of infection and worse outcomes [15], [16]. Local smoking prevalences were obtained from the Smoking prevalence for local and unitary authorities in England database [17].

The Index of Multiple Deprivation (IMD) was included, because there is evidence that living conditions are associated with the risk of respiratory tract infections [18], [19]. Moreover, other lifestyle factors partially captured by the IMD may be associated with an increased risk of infection [15]. This index is available from the Department for Communities and Local Government (now named the ‘Ministry of Housing, Communities & Local Government’) which ranks very small areas (~ 1500 people) called lower-layer super output areas (LSOAs), according to a weighted score for employment, income, education, health, crime, living environment, and barriers to housing and services [20]. The IMD was linked to each practice based on the LSOAs the practice was serving; the smoking prevalence was linked to each practice based on the local authority the practice served.

### 2.1 Analyses

For the primary analysis, we created a dataset including only practices with no missing data for any of the predictors. In line with a previous study [21], we excluded practices with a list size of fewer than 750 patients, as these practices were likely newly formed or about to close. The dispensing dataset initially contained data on 7934 practices. Of these, 486 (6%) practices were removed due to missing data. The most common reasons were missing information on comorbidity prevalences (n = 357) and smoking prevalence (n = 127). After excluding practices with missing information, 7448 practices remained. After additionally removing practices with less than 750 registered patients, 7376 practices remained.

The ranking of practices based on their antibiotic prescribing rate per STAR-PU is straightforward. This can be accomplished by dividing the number of antibiotic items by the amount of STAR-PU for each practice and subsequently sorting the practices based on the obtained values. To additionally take into account other legitimate medical reasons for variation in antibiotic prescribing, we build two negative binomial regression models in order to evaluate the association between included predictors and antibiotic prescribing rates per STAR-PU at the practice level. The natural logarithm of the amount of STAR-PU per practice was included as on offset to take into account differences in practice sizes and age- and gender distributions of the practices. We built two different models: i) a model with only comorbidity prevalences as potential predictors, ii) a model with comorbidities, deprivation score, and smoking prevalence as potential predictors. We did not consider non-linearity for comorbidities and the smoking prevalence, because we assumed that the medical legitimacy of an antibiotic prescription for an individual is independent of the total number of smokers or patients with comorbidities registered with a practice. The IMD was categorised based on the quintiles of its distribution.

For both negative binomial regression models, variables were selected for inclusion in the model using stepwise backward selection based on the Akaike Information Criterion (AIC). Subsequently, we removed variables that had a negative association with the antibiotic prescribing rate from the model, because to make the comparison between practices more fair we wanted to only take into account factors that both in theory and in practice are associated with higher (legitimate) antibiotic prescribing rates.

To evaluate to what extent the models could explain the differences in antibiotic prescribing, we estimated the amount of deviance explained by each model. To enable a fair comparison of the deviance explained by each model, we fixed the dispersion parameter at the estimate derived for the model which considered all variables as potential predictors.

We subsequently evaluated whether the same practices would be identified as being among the top 20% regarding their antibiotic prescribing rate when ranking them according to the current methodology using antibiotic items per STAR-PU versus to what extent a practice prescribes above or below their predicted antibiotic prescribing rate per STAR-PU. The latter was calculated by taking the observed prescribing rate per STAR-PU minus the predicted prescribing rate per STAR-PU. The predicted prescribing rate per STAR-PU was obtained using the model with comorbidity only and the model considering all factors listed above. An area-proportional Venn diagram was used to visualise to what extent different practices were among the top 20% of antibiotic prescribers using the current methodology and the new model-based methodologies. All analyses were performed using R, version 3.3.2.

### 2.2 Sensitivity Analyses

For the primary analysis, we excluded practices that had fewer than 750 registered patients. However, after excluding those practices, a few practices remained with questionable high/low antibiotic prescribing rates per STAR-PU (e.g. 0.05 or 17.14 antibiotics per STAR-PU). As we could not exclude the possibility that some of these practices were genuinely prescribing much more or less than most other practices, we kept them in the primary analyses. For sensitivity analyses, we further restricted the data used in the primary analyses by only using data from practices that had a prescribing rate between 1/3 and 3 antibiotics per STAR-PU. Values outside this range are less likely to be correct. Such implausible antibiotic prescribing rates may be due to data entry errors or due to the fact that a practice serves relatively many patients that are not registered at the practice, which increases the numerator but not the denominator.

Because one may argue whether the prevalence of some of the included comorbidities could be considered legitimate reasons for higher antibiotic prescribing rates, we added a sensitivity analysis with a much more restricted model only taking into account the COPD and diabetes prevalence.

## 3 Results

The characteristics of patients registered at the 7376 included practices are summarised in Table 2. The median number of antibiotic items prescribed per STAR-PU was 1.09 (25th -75th percentile, 0.92–1.25) between April 2014 and March 2015 (Table 2). In univariate analysis, most potential predictors were associated with a higher use of antibiotics per STAR-PU. The only exception was the cancer prevalence which had a negative association with the antibiotic prescribing rate (Table 2). The number of antibiotics prescriptions per STAR-PU was higher among practices from more deprived areas. Taking the first quintile of the IMD as a reference, the incidence rate ratios increased with each quintile (2nd: 1.010 (95% CI 0.978–1.043); 3rd: 1.028 (95% CI 0.995–1.061); 4th: 1.064 (95% CI 1.030–1.098); 5th 1.103 (95% CI 1.069–1.139)).

**Table 2:** Distribution of included variables and univariate association with number of antibiotics per STAR-PU.

	Median (25th–75th percentile)	Simple linear association with antibiotic/STAR-PU, relative risk (95% CI)
Antibiotics per STAR-PU	1.09 (0.92–1.25)	–
Smoking prevalence	19.77 (17.40–22.82)	1.014 (1.012–1.015)
CHD prevalence	3.27 (2.50–4.01)	1.043 (1.037–1.050)
Diabetes prevalence	6.52 (5.53–7.52)	1.020 (1.016–1.024)
HF prevalence	0.69 (0.49–0.90)	1.063 (1.042–1.085)
Asthma prevalence	5.99 (5.15–6.81)	1.048 (1.042–1.054)
COPD prevalence	1.75 (1.23–2.39)	1.090 (1.081–1.098)
Cancer prevalence	2.22 (1.60–2.77)	0.983 (0.975–0.992)
CKD prevalence	3.87 (2.67–5.24)	1.007 (1.004–1.011)

CHD = chronic heart disease. CKD = chronic kidney disease. COPD = chronic obstructive pulmonary disease. HF = heart failure. STAR-PU = Specific Therapeutic Group Age-sex weighting Related Prescribing Unit.

The negative binomial regression model, which only allowed comorbidities to be selected as predictors, included the asthma (RR 1.025, 95% CI 1.016–1.035), COPD (RR 1.070, 95% CI 1.056–1.084) and diabetes prevalence (RR 1.008, 95% CI 1.003–1.014) as predictors of higher antibiotic use per STAR-PU (Table 3). This model explained 7.5% of the deviance. The second negative binomial regression model additionally allowed the smoking prevalence and IMD to be selected as predictors. After stepwise variable selection, this second model included the same comorbidities as model 1, but additionally included the smoking prevalence (RR 1.005, 95% CI 1.003–1.007) and IMD quintiles as predictors (2nd: RR 1.000, 95% CI 0.978–1.022; 3rd: RR 1.010, 95% CI 0.987–1.033; 4th: RR 1.044, 95% CI 1.020–1.069; 5th: RR 1.095, 95% CI 1.068–1.122) (Table 3). This model explained 9.1% of the deviance.

**Table 3:** Estimated associations between predictor variables and number of antibiotics per STAR-PU.

Predictor	Comorbidity model Relative risk (95% CI)	Full model Relative risk (95% CI)
Asthma prevalence	1.025 (1.016–1.035)	1.034 (1.027–1.040)
COPD prevalence	1.070 (1.056–1.084)	1.059 (1.049–1.068)
Diabetes prevalence	1.008 (1.003–1.014)	1.002 (0.998–1.006)
Smoking prevalence	NA	1.005 (1.003–1.007)
Deprivation 1	NA	Ref.
Deprivation 2	NA	1.000 (0.978–1.022)
Deprivation 3	NA	1.010 (0.987–1.033)
Deprivation 4	NA	1.044 (1.020–1.069)
Deprivation 5	NA	1.095 (1.068–1.122)

COPD = chronic obstructive pulmonary disease. STAR-PU = Specific Therapeutic Group Age-sex weighting Related Prescribing Unit.

**Fig. 1:**
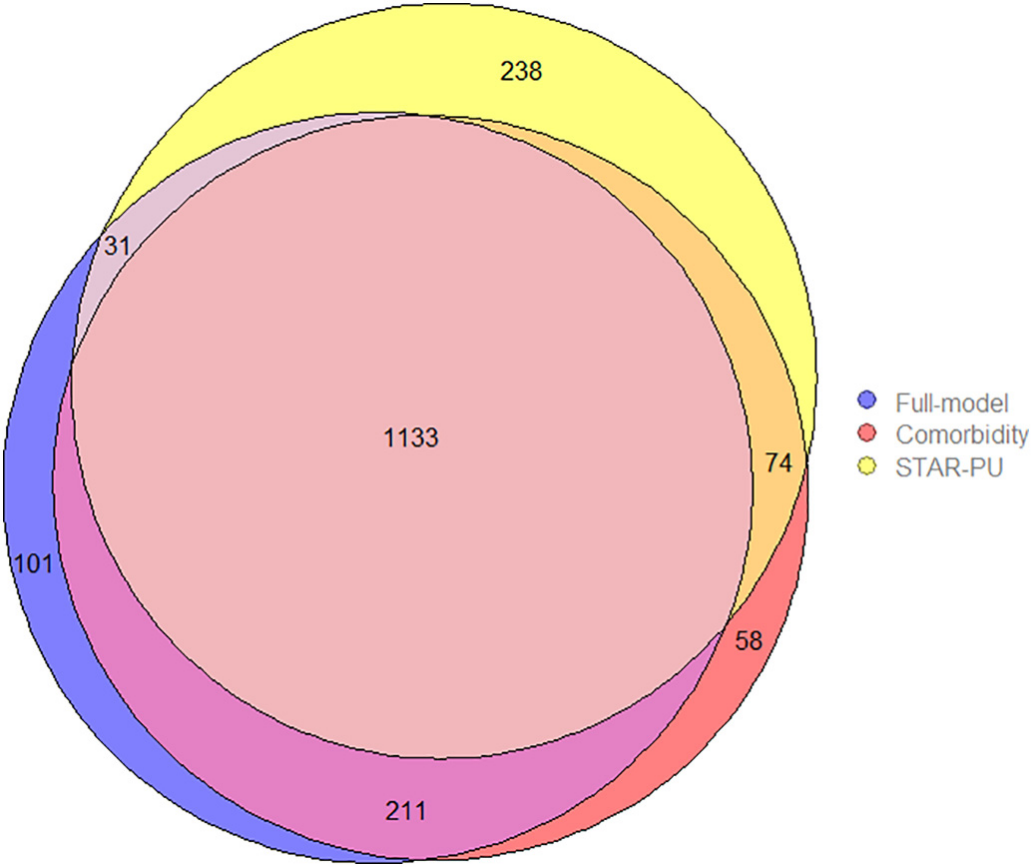
Venn diagram of identifying top 20% of practices in terms of antibiotic prescribing rate per STAR-PU using three different methodologies. The full model considered all variables (blue ellipse, see Table 2), the comorbidity model considered only comorbidities (pink ellipse, see Table 2) and the current methodology is based on ranking practices based on their antibiotic prescribing rate per STAR-PU without taking into account any other variables (yellow ellipse). STAR-PU: Specific Therapeutic Group Age-sex weighting Related Prescribing Unit.

The Venn diagram in Fig. 1 shows that there is substantial overlap between the current methodology based on STAR-PU and the model-based methodologies that take into account comorbidities alone or comorbidities, smoking prevalence, and IMD together. There was variation in which 1476 (top 20%) practices were included by the three different methods. The three different methods agreed on 1133 practices (76.8% of 1476) being high antibiotic prescribers. However, some practices that are classified as high prescribers using the current STAR-PU methodology are not classified as high prescribers when taking into account comorbidities alone (n = 269, 18.2%) or comorbidities, smoking prevalence, and IMD (n = 312, 21.1%). Similarly, some practices that are not identified as high prescribers using current STAR-PU methodology are classified as high prescribing using one (n = 159) or both (n = 211) of the new model-based approaches.

The difference in ranking of all included practices, including those never identified as being among the top 20% antibiotic prescribers, based on the different methodologies is shown in Table S1. The same identifiable practice code as used in the publicly available data is included in this table, to enable practices to identify their own practice.

### 3.1 Sensitivity Analysis

When only taking into account the prevalence of diabetes and COPD, results were very similar to the model that also allowed for other comorbidities. These two methods agreed on 1368 (92.7% of 1476) being high antibiotic prescribers.

In a subsequent sensitivity analysis, 7311 practices remained after excluding practices with antibiotic prescribing rates per STAR-PU below 1/3 or above 3. Besides the comorbidities selected in the main analysis, the coronary heart disease prevalence (RR 1.028, 95% 1.021–1.035) was also included in the final comorbidity only model (Table 4). This model explained 19.1% of the deviance. The final model, which considered all variables as potential predictors, was similar to the main analysis, except that the IMD was no longer included in the final model (19.4% of deviance explained).

**Table 4:** Estimated associations between predictor variables and number of antibiotics per STAR-PU after removing outer 1% in terms of antibiotic prescribing rate per STAR-PU.

Predictor	Comorbidity model Relative risk (95% CI)	Full model Relative risk (95% CI)
Asthma prevalence	1.021 (1.016–1.026)	1.022 (1.017–1.026)
COPD prevalence	1.058 (1.050–1.066)	1.050 (1.042–1.058)
Diabetes prevalence	1.023 (1.020–1.026)	1.022 (1.019–1.025)
CHD prevalence	1.028 (1.021–1.035)	1.029 (1.022–1.036)
Smoking prevalence	NA	1.004 (1.003–1.006)

CHD = coronary heart disease. COPD = chronic obstructive pulmonary disease. STAR-PU = Specific Therapeutic Group Age-sex weighting Related Prescribing Unit.

**Fig. 2:**
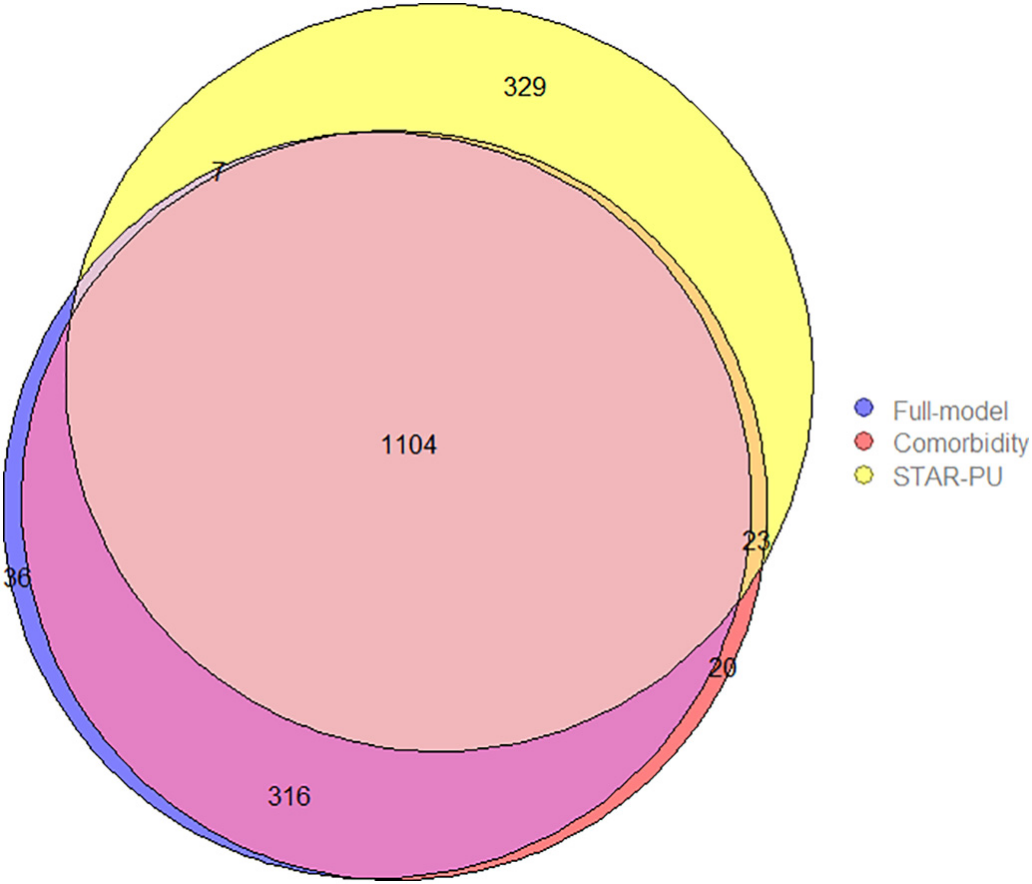
Venn diagram of identifying top 20% of practices in terms of antibiotic prescribing rate per STAR-PU using three different methodologies, after removing outer 1% of practices based on antibiotic prescribing rate per STAR-PU. The full model considered all variables (blue ellipse, see Table 2), the comorbidity model considered only comorbidities (pink ellipse, see Table 2) and the current methodology is based on ranking practices based on their antibiotic prescribing rate per STAR-PU without taking into account any other variables (yellow ellipse). STAR-PU: Specific Therapeutic Group Age-sex weighting Related Prescribing Unit.

Similar to the main analysis, there is substantial overlap between the different methodologies to identify the top 20% of antibiotic prescribers. In 1104 out of 1463 (75.5%) the STAR-PU based method and the model-based methods agree on which practices are among the top 20% highest antibiotic prescribers (Fig. 2). Table S2 shows the ranking of all practices based on the three different methodologies.

## 4 Discussion

This study found that current comparisons of antibiotic prescribing rates of general practices based on STAR-PU weighting only may be unfair for some practices. Although STAR-PU weighting does account for differences in the age and gender distribution of patient populations, using STAR-PU weighting alone results in practices being identified as high antibiotic prescribers which would not be ranked so highly when also taking into account other legitimate reasons for variation, such as comorbidities. Similarly, some practices with relatively healthy populations are missed as high prescribers when using only STAR-PU weightings.

### 4.1 Strength and Weaknesses of Study

This study used publicly available data, covering all patients registered with a general NHS practice in England. There are several studies that tried to find reasons why some practices prescribe substantially more antibiotics than other practices [6], [21], [22], [23], [24] and some have suggested that comparisons between practices should take into account potentially legitimate reasons for higher antibiotic prescribing rates [24]. However, to our knowledge no study has compared practices prescribing rates accounting for additional legitimate reasons for prescribing using data available at a national level. Our study has direct policy implications because individual practices that are still identified as prescribing above the norm even after accounting for comorbidities and other legitimate reasons for higher antibiotic prescribing rates, can be identified at a national level, thus helping to inform intervention decisions.

Although comorbidity prevalences were available at the practice level, other predictors could only be linked to a practice based on the local authorities the practice served. This could result in biased estimates if, for example, a practice in a relatively deprived area attracted patients with a higher socioeconomic status while all other patients attend other practices in the area. Potential legitimate reasons for higher prescribing in certain practices not available for this study include, for example, the prevalence of immunosuppressive diseases, consultation rates, and markers of the severity of infections [6], [25]. However, immunosuppressive diseases are relatively rare and are therefore unlikely to explain a lot of the variation in antibiotic prescribing [6]. Although RTI consultation rates seem to explain a substantial part of the variation in antibiotic prescribing [6], it is at least questionable whether these consultations differ due to true differences in medical need [6]. Differences in consultation rates may instead reflect difference in health-care seeking behaviour and it has previously been shown that high antibiotic prescribing rates result in higher consultation rates and medicalisation of self-limiting infections [26]. Because it is questionable whether (apparent) higher consultation rates reflect legitimate medical reasons for higher antibiotic prescribing rates, accounting for differences in consultation rates – if available – would not necessarily lead to more fair comparisons.

Beyond the issue of potentially not having included all medically legitimate reasons for variations in antibiotic prescribing, the model-based approaches require a valid model to obtain predicted antibiotic prescribing rates per STAR-PU for every practice given the characteristics of their patient population. A model additionally including comorbidities that had a negative association with the antibiotic prescribing rate and non-linear functions for continuous variables would be better at explaining the variation between practices [6]. However, we decided to include only variables for which clear theoretical medical reasons why they may be associated with higher antibiotic prescribing rates existed, i.e. according to guidelines or based on the literature, and for which in practice a positive association with the antibiotic prescribing rate per STAR-PU was observed. The percentage of deviance explained was relatively low, indicating that a large part of the variation in antibiotic prescribing is not explained by the considered legitimate medical reasons for variation in prescribing. Potential reasons for variation in antibiotic prescribing that do not represent legitimate medical reasons include for example variation in inappropriate prescribing and health-care seeking behaviour of the patient population [3], [4], [6].

Although guidelines indicate that practices with more patients with comorbidities could legitimately prescribe more antibiotics for RTIs, the relationship between comorbidities and antibiotic prescribing for suspected UTI is complex. Asymptomatic bacteriuria is associated with comorbidity and frequently inappropriately treated with antibiotics in nursing homes [27]. However, at the general practice level, variation in antibiotic prescribing for RTIs – for which guidelines indicate that comorbidities are legitimate reasons for a higher likelihood of prescribing – is much more relevant in explaining variation in total antibiotic prescribing [3], [6].

It is important to note that this study does not address the issues of what constitutes ‘safe’ levels of prescribing or what proportion of antibiotics is inappropriate. In other words, this study seeks to rank practices' antibiotic prescribing in a fair manner, but it provides no insight into appropriate absolute levels of prescribing. If the majority of practices were overprescribing, the expected antibiotic prescribing rate from the model would still include a substantial proportion of inappropriate antibiotic prescriptions. Other recent work has attempted to quantify inappropriate prescribing in English primary care [3], [4]. That work indicates that even currently low prescribing English practices overprescribe antibiotics [3], [4].

### 4.2 Implications

Several countries, including England, currently aim to reduce inappropriate antibiotic prescribing in order to tackle the problem of increasing levels of antibiotic resistance. The findings presented here can be used to identify practices that prescribe above the norm, even when taking into account age and sex distributions, the prevalence of comorbidities, smoking and deprivation of the patient population. These results can also be used to better identify high prescribing practices and target interventions designed to reduce antibiotic prescribing in those practices which might have a much greater potential to lower antibiotic prescribing rates. By identifying factors that are both in theory and in practice associated with higher antibiotic prescribing rates, this work may create a rational basis for incentivising stewardship with relation to different population groups. Although these factors can be considered as legitimate medical reasons for higher antibiotic prescribing rates, they include factors that can either be directly modified, such as smoking prevalence [28], or via underlying risk factors such as lifestyle interventions to prevent diabetes [29].

Given that the model is based on publicly available data which is regularly updated, the model can be updated on a regular basis to reflect changes in prescribing and underlying patient populations.

## 5 Conclusion

Current age- and sex-weighted comparisons of antibiotic prescribing rates in England are fair for most, but not all practices. Some practices can legitimately claim that they have a more frail patient population and prescribe, in line with guidelines in England, more antibiotics than other practices. After additionally accounting for prevalences in comorbidity such practices would no longer be identified as being among the top 20% of high prescribers (yet, it is still possible that they overprescribe antibiotics). Similarly, other, currently missed, practices would move into the ‘high prescriber’ category if more inclusive methods were adopted. Our study produced a tool that can be regularly updated and fine-tuned using publicly available data in order to facilitate more fair and up-to-date comparisons of antibiotic prescribing rates of general practices in England.

## Author Contributions

EH cleaned and analysed the data and drafted the manuscript. REC contributed statistical analysis code and revised the manuscript. TDH and TS designed the study and revised the manuscript. JVR conceptualised the study and revised the manuscript. KBP conceptualised and designed the work, analysed the data, and revised the manuscript. All authors contributed to critical review of manuscript drafts and reviewed and approved the final manuscript.

## Declaration of Interests

We declare no competing interests.

## Appendix A Supplementary data

Table S1. Ranking of general practices in the main analysis based on antibiotic prescribing per STAR-PU, the comorbidity and full model. Image 1

Table S2. Ranking of general practices in the sensitivity analysis based on antibiotic prescribing per STAR-PU, the comorbidity and full model. Image 2
