# Supplementary material for: Identifying English Practices that Are High Antibiotic Prescribers Accounting for Comorbidities and Other Legitimate Medical Reasons for Variation
Source: eClinicalMedicine. 2018 Dec 12;6:36–41. doi: 10.1016/j.eclinm.2018.12.003 (PMC6358038; doi:10.1016/j.eclinm.2018.12.003)
Supplement: Supplementary file 1 — Table S1. Ranking of general practices in the main analysis based on antibiotic prescribing per STAR-PU, the comorbidity and full model. [file mmc1.pdf]

Practice code	Observed prescribing rate per STAR-PU	Predicted prescribing rate per STAR-PU based on comorbidity model	Predicted prescribing rate per STAR-PU based on full model	Ranking based on STAR-PU	Ranking based on comorbidity model	Ranking based on full model
Y02568	17.141	0.981	1.034	1	1	1
Y00527	14.354	1.311	1.307	2	2	2
Y02499	13.160	1.171	1.298	3	3	3
Y02614	11.436	1.205	1.327	4	4	4
Y02611	10.963	1.000	1.034	5	5	5
Y02704	9.519	1.055	1.163	6	6	6
Y02586	9.166	1.034	1.119	7	7	7
Y02483	8.369	1.090	1.206	8	8	8
Y02596	7.464	1.036	1.133	9	9	9
Y02875	7.350	1.015	1.112	10	10	10
Y02509	6.868	0.961	1.066	11	11	11
Y03051	6.404	1.050	1.114	12	12	12
Y02795	6.317	1.175	1.283	13	15	15
Y02638	6.156	0.945	1.021	14	13	14
Y02463	6.088	0.883	0.915	15	14	13
Y02823	5.910	0.900	0.953	16	16	16
Y02736	5.395	1.212	1.328	17	20	20
Y02669	5.337	1.103	1.151	18	18	18
Y02961	5.256	0.915	0.956	19	17	17
Y02868	5.194	1.105	1.232	20	21	21
Y02494	5.153	0.928	1.020	21	19	19
Y02707	4.835	1.162	1.193	22	23	23
Y03054	4.686	0.993	1.017	23	22	22
Y02751	3.946	0.944	0.969	24	24	24
Y02962	3.902	0.921	0.984	25	25	25
Y02646	3.707	1.002	1.017	26	27	27
Y02692	3.682	0.877	0.869	27	26	26
Y02816	3.598	0.977	1.014	28	29	29
Y02946	3.502	0.876	0.847	29	28	28
Y02495	3.464	1.032	1.051	30	32	32
Y02442	3.457	0.956	1.022	31	30	30
Y02620	3.443	0.961	1.029	32	31	31
Y02519	3.376	1.067	1.087	33	33	33
Y02462	3.243	1.134	1.191	34	35	35
Y02526	3.085	0.963	1.014	35	34	34
Y02778	3.075	1.033	1.042	36	36	36
Y02684	3.002	1.191	1.302	37	38	39
Y02421	2.853	1.028	1.128	38	37	38
Y02625	2.740	1.880	1.774	39	75	60
Y02974	2.668	0.897	0.905	40	39	37
Y02566	2.651	0.919	1.026	41	40	40
Y02639	2.624	0.959	1.014	42	41	41
G82641	2.561	1.192	1.137	43	44	42

Y02570	2.502	1.079	1.122	44	43	43
F86686	2.456	1.020	1.081	45	42	44
Y02662	2.455	1.090	1.157	46	45	46
N82036	2.383	1.503	1.433	47	72	62
Y02973	2.362	1.007	1.035	48	46	45
Y02890	2.347	1.009	1.136	49	47	49
C86621	2.336	1.365	1.374	50	63	61
A82077	2.311	1.104	1.117	51	50	51
Y02720	2.246	1.111	1.183	52	55	56
Y02711	2.239	1.102	1.189	53	54	57
B81683	2.231	1.203	1.339	54	59	68
Y02968	2.221	1.013	1.018	55	51	50
Y00265	2.199	0.908	0.905	56	48	47
E85105	2.195	1.032	1.086	57	53	52
B81018	2.169	1.452	1.598	58	116	178
Y03296	2.161	0.893	0.944	59	49	48
B85048	2.157	1.139	1.223	60	60	64
C88042	2.153	1.308	1.313	61	78	74
Y02656	2.103	1.376	1.380	62	108	96
G82696	2.103	1.315	1.339	63	87	86
P84024	2.087	1.324	1.334	64	93	88
P85605	2.087	1.182	1.248	65	68	75
Y02849	2.083	0.888	1.000	66	52	54
M86604	2.082	1.033	1.087	67	58	59
Y02578	2.072	0.942	0.998	68	56	55
F81037	2.071	1.163	1.124	69	67	63
G82007	2.060	1.227	1.214	70	80	70
M91022	2.054	1.216	1.284	71	79	85
M91639	2.054	1.245	1.299	72	83	87
M85722	2.050	0.926	0.951	73	57	53
H85643	2.026	1.168	1.191	74	76	76
B83613	2.022	1.046	1.089	75	62	65
K81085	2.018	1.021	0.985	76	61	58
F83030	2.015	1.095	1.114	77	66	67
B81674	2.010	1.270	1.414	78	101	167
M85001	2.007	1.299	1.342	79	119	123
A89624	2.007	1.153	1.231	80	77	83
N82647	2.002	1.067	1.142	81	64	69
C84031	1.976	1.096	1.191	82	73	81
C88021	1.974	1.091	1.130	83	71	72
C84140	1.972	1.151	1.154	84	81	78
B87006	1.967	1.396	1.405	85	184	183
N82101	1.967	1.402	1.483	86	190	247
D81624	1.959	1.172	1.185	87	88	84
P81763	1.953	1.074	1.125	88	74	77
A82040	1.933	1.376	1.394	89	193	198
P85610	1.933	1.128	1.225	90	84	106
P87652	1.932	1.162	1.250	91	90	114
F84657	1.929	1.193	1.211	92	103	99
P84014	1.910	1.223	1.266	93	125	135

F82042	1.906	0.987	1.061	94	65	71
C84704	1.906	1.251	1.351	95	135	187
B85060	1.906	1.365	1.422	96	207	248
Y02718	1.903	1.134	1.208	97	91	110
J83649	1.900	1.111	1.208	98	86	112
N82678	1.899	1.138	1.234	99	94	124
F81212	1.898	1.270	1.238	100	150	126
B81012	1.896	1.100	1.183	101	85	102
N83031	1.892	1.146	1.214	102	97	116
F81156	1.890	1.441	1.410	103	303	252
A89041	1.890	1.181	1.273	104	118	151
P86619	1.890	1.078	1.073	105	82	79
F81104	1.886	1.119	1.172	106	92	101
B83069	1.885	1.161	1.247	107	109	139
G82722	1.883	1.160	1.161	108	110	97
B81087	1.882	1.142	1.183	109	99	108
B81606	1.872	1.268	1.377	110	161	235
C83028	1.871	1.189	1.136	111	126	90
A81038	1.870	1.265	1.321	112	162	192
Y00026	1.868	1.121	1.234	113	96	142
P91615	1.867	1.084	1.169	114	89	109
P84055	1.866	1.176	1.290	115	124	172
G81651	1.866	1.323	1.350	116	202	219
C87616	1.861	1.262	1.243	117	165	149
G82204	1.857	1.129	1.107	118	107	89
D81015	1.857	1.114	1.124	119	98	93
B85009	1.856	1.136	1.150	120	113	107
G82235	1.854	1.137	1.136	121	115	100
L83643	1.854	1.365	1.518	122	249	534
Y02162	1.854	1.131	1.212	123	111	136
A87023	1.853	1.149	1.143	124	120	104
H81611	1.851	0.966	0.921	125	70	66
K81039	1.851	1.333	1.313	126	220	199
N83611	1.847	1.345	1.414	127	234	304
P85608	1.847	1.424	1.534	128	341	608
A83617	1.844	1.251	1.238	129	170	158
P84025	1.841	1.101	1.120	130	100	98
P82033	1.839	1.170	1.229	131	131	153
G82233	1.838	1.119	1.127	132	114	103
C85624	1.837	1.552	1.616	133	715	1064
P87036	1.834	1.096	1.196	134	102	140
C83030	1.832	1.264	1.218	135	186	152
C86625	1.827	1.321	1.463	136	231	442
P84049	1.826	1.302	1.323	137	216	230
P82022	1.818	1.202	1.290	138	155	207
M85730	1.817	1.174	1.244	139	140	175
P84029	1.815	1.334	1.332	140	260	249
P88003	1.811	1.302	1.269	141	224	196
H85019	1.810	1.076	1.021	142	104	80
M91624	1.810	1.137	1.204	143	129	157

C82008	1.809	1.172	1.131	144	144	117
J82077	1.807	1.145	1.074	145	133	94
P81756	1.805	1.176	1.219	146	149	170
Y02354	1.795	1.190	1.194	147	160	164
N85640	1.794	1.308	1.379	148	254	333
B87604	1.793	1.300	1.336	149	239	272
Y02585	1.793	0.903	0.951	150	69	73
F84091	1.791	1.037	1.058	151	95	92
A89004	1.791	1.171	1.143	152	154	132
Y02414	1.791	1.113	1.150	153	127	137
Y02605	1.790	1.207	1.371	154	177	324
G82029	1.787	1.159	1.126	155	148	125
M85779	1.786	1.210	1.266	156	182	214
Y00469	1.786	1.487	1.501	157	664	726
Y01200	1.785	1.216	1.375	158	185	344
C88647	1.784	1.147	1.222	159	143	182
F81060	1.783	1.273	1.298	160	223	246
K82050	1.779	1.140	1.085	161	142	111
C82019	1.771	1.113	1.167	162	134	160
A89612	1.765	1.257	1.308	163	228	273
N81626	1.763	1.173	1.126	164	173	141
P91617	1.760	1.029	1.112	165	106	131
N83008	1.756	1.475	1.529	166	731	1020
G81075	1.755	1.179	1.197	167	181	186
M84026	1.754	1.210	1.194	168	201	185
P86019	1.753	1.148	1.189	169	158	181
M83616	1.751	1.107	1.124	170	138	145
C84629	1.751	1.262	1.273	171	245	254
G84028	1.743	1.079	1.071	172	132	119
B85020	1.742	1.218	1.234	173	217	225
P86022	1.740	1.178	1.180	174	192	184
C86616	1.740	1.511	1.520	175	989	1069
G81669	1.739	1.187	1.213	176	198	210
M91659	1.738	1.248	1.295	177	242	288
P86011	1.738	1.249	1.218	178	246	213
H83052	1.737	1.021	1.003	179	117	91
P92616	1.737	1.210	1.232	180	213	228
N81127	1.736	1.290	1.217	181	307	215
H82101	1.733	1.092	1.063	182	141	122
G82791	1.732	1.055	1.076	183	128	127
D82072	1.732	1.111	1.079	184	153	128
C81008	1.729	1.221	1.159	185	227	179
C88626	1.728	1.410	1.491	186	607	952
A83638	1.725	1.365	1.411	187	476	599
D81065	1.724	1.101	1.123	188	151	163
B87036	1.721	1.267	1.280	189	297	291
F82668	1.719	1.154	1.228	190	191	242
P86602	1.719	1.223	1.265	191	237	279
P83020	1.717	1.227	1.261	192	243	275
M85008	1.716	1.441	1.454	193	754	816

G82762	1.715	1.025	1.115	194	123	166
G83057	1.713	1.107	1.062	195	157	129
B83638	1.712	1.122	1.205	196	172	226
H85008	1.712	1.015	0.983	197	122	95
B81054	1.711	1.226	1.290	198	253	320
N82019	1.704	1.186	1.236	199	221	262
P81212	1.702	1.207	1.176	200	236	209
F81670	1.702	1.323	1.333	201	428	429
M85713	1.701	1.149	1.150	202	199	191
A82041	1.701	1.160	1.127	203	206	173
C88072	1.699	1.375	1.348	204	582	482
G83066	1.698	1.103	1.050	205	168	133
P87040	1.694	1.320	1.370	206	436	562
D81055	1.694	1.218	1.183	207	269	223
P81687	1.694	1.104	1.076	208	174	150
N82034	1.693	1.277	1.279	209	351	331
P81643	1.690	1.092	1.085	210	166	159
B81019	1.689	1.246	1.347	211	309	515
Y03023	1.687	0.986	1.007	212	121	115
F81635	1.686	1.084	1.080	213	163	156
B85619	1.682	1.258	1.308	214	339	413
F81014	1.682	1.086	1.048	215	167	143
M91028	1.680	1.277	1.268	216	370	341
P81196	1.678	1.034	0.987	217	139	113
B81101	1.677	1.156	1.135	218	218	195
Y00110	1.677	1.336	1.339	219	518	524
B81121	1.676	1.048	1.054	220	147	147
N83603	1.673	1.130	1.136	221	203	202
N83605	1.672	1.270	1.286	222	372	386
P81785	1.672	1.272	1.323	223	379	490
N84016	1.671	1.231	1.220	224	314	281
G83663	1.670	1.117	1.178	225	195	238
L83664	1.670	1.228	1.334	226	311	533
E83600	1.670	0.936	0.890	227	105	82
G82022	1.669	1.079	1.050	228	171	148
G82094	1.667	1.101	1.064	229	189	162
C83614	1.666	1.183	1.176	230	259	243
F86612	1.665	1.011	0.955	231	136	105
A83626	1.665	1.247	1.242	232	347	319
M91655	1.664	1.321	1.373	233	510	697
M87018	1.664	1.225	1.261	234	316	361
F81681	1.662	1.756	1.670	235	4459	3352
G82155	1.662	1.060	1.021	236	164	138
M86629	1.662	1.109	1.108	237	197	189
G82737	1.661	1.011	1.015	238	137	134
C86032	1.661	1.052	1.056	239	156	161
B84001	1.660	1.251	1.251	240	365	350
N83018	1.659	1.222	1.176	241	317	250
A89019	1.659	1.279	1.231	242	424	309
F81656	1.658	1.133	1.194	243	214	266

N84015	1.655	1.368	1.434	244	703	1059
B85646	1.655	1.198	1.208	245	294	284
A89610	1.654	1.201	1.280	246	299	416
G82023	1.652	1.250	1.286	247	374	437
F82680	1.652	1.237	1.342	248	355	617
P84028	1.651	1.157	1.130	249	238	212
P82010	1.650	1.274	1.289	250	432	451
M91641	1.648	1.212	1.290	251	318	458
B86089	1.647	1.130	1.129	252	222	216
A89621	1.647	1.254	1.341	253	392	634
C88006	1.646	1.374	1.468	254	761	1366
A83029	1.646	1.156	1.128	255	244	217
A86013	1.644	1.302	1.274	256	513	425
M83128	1.644	1.158	1.202	257	252	289
C82650	1.644	1.119	1.092	258	215	190
M85149	1.643	1.233	1.261	259	364	400
N82048	1.641	1.225	1.244	260	350	369
J81632	1.639	1.242	1.272	261	386	434
B83631	1.638	1.046	1.107	262	169	204
E82020	1.638	1.097	1.054	263	204	171
N85038	1.638	1.405	1.448	264	964	1267
D81071	1.637	1.299	1.265	265	528	422
Y02986	1.637	0.917	0.967	266	112	120
A83060	1.635	1.107	1.136	267	212	232
G82170	1.635	0.966	0.961	268	130	118
C82099	1.635	1.006	1.035	269	146	165
C88643	1.634	1.151	1.243	270	257	378
B81108	1.634	1.259	1.379	271	435	853
E85656	1.634	1.187	1.225	272	304	348
M85077	1.633	1.094	1.099	273	209	203
P84059	1.631	1.151	1.172	274	264	270
B83653	1.631	1.090	1.103	275	205	206
A89014	1.630	1.138	1.216	276	240	337
F81740	1.630	1.265	1.323	277	455	631
M85134	1.628	1.129	1.193	278	235	303
A89617	1.627	1.180	1.241	279	306	387
A83016	1.627	1.279	1.251	280	497	409
Y00286	1.627	1.197	1.183	281	323	286
N82001	1.627	1.362	1.308	282	799	582
L83037	1.626	1.284	1.333	283	516	688
E85090	1.624	1.040	1.036	284	176	168
J83609	1.624	1.002	0.976	285	152	130
C82676	1.621	1.138	1.125	286	258	234
F85634	1.621	1.042	1.066	287	178	188
H83027	1.620	1.016	0.996	288	159	146
C81661	1.619	1.258	1.236	289	474	398
H83620	1.617	0.987	0.947	290	145	121
L84081	1.617	1.190	1.153	291	329	265
P81167	1.617	1.349	1.501	292	776	1920
M85066	1.616	1.153	1.192	293	287	317

L83626	1.616	1.307	1.385	294	632	994
A87013	1.614	1.261	1.231	295	485	392
L81066	1.614	1.095	1.049	296	219	180
G82150	1.613	1.234	1.242	297	427	423
G82046	1.613	1.405	1.366	298	1115	893
P82634	1.613	1.219	1.305	299	391	626
P81764	1.612	1.156	1.183	300	296	307
A89030	1.611	1.242	1.265	301	446	499
F84006	1.606	1.032	0.996	302	183	154
K81005	1.605	1.098	1.104	303	229	231
B86638	1.603	1.218	1.348	304	412	850
A89022	1.601	1.214	1.187	305	404	330
N83021	1.600	1.176	1.202	306	340	367
F81679	1.599	1.033	1.025	307	188	174
A81045	1.599	1.281	1.248	308	605	480
N85037	1.598	1.254	1.335	309	505	812
A87027	1.598	1.258	1.263	310	524	535
D82038	1.598	1.205	1.173	311	393	315
N81637	1.598	1.021	1.083	312	180	220
M91654	1.596	1.042	1.098	313	194	233
C85022	1.596	1.170	1.154	314	333	290
B87021	1.596	1.334	1.326	315	815	779
Y00484	1.596	1.181	1.142	316	356	278
M83712	1.596	1.135	1.242	317	290	471
G83003	1.594	1.195	1.250	318	383	507
A86021	1.594	1.252	1.252	319	520	516
M85686	1.593	1.116	1.179	320	268	334
M83641	1.592	1.128	1.046	321	284	194
Y03595	1.591	1.272	1.251	322	598	520
G82113	1.591	1.110	1.147	323	261	287
C86002	1.590	1.194	1.182	324	388	351
Y02769	1.590	1.051	1.053	325	208	200
A83037	1.590	1.229	1.196	326	470	375
D83079	1.589	1.118	1.112	327	277	255
A83627	1.589	1.680	1.654	328	4406	4123
A89005	1.588	1.189	1.272	329	381	586
E81063	1.587	1.084	1.093	330	233	237
A86009	1.586	1.250	1.273	331	538	598
G82691	1.586	1.097	1.113	332	248	257
J81063	1.586	1.139	1.150	333	305	299
P81004	1.586	1.318	1.445	334	784	1710
N81625	1.585	1.158	1.231	335	331	470
F81683	1.585	1.032	1.014	336	196	177
B82045	1.585	1.184	1.146	337	376	293
Y01124	1.582	1.241	1.346	338	517	950
F86007	1.582	1.108	1.057	339	272	211
L85620	1.582	1.108	1.091	340	273	239
B83052	1.582	1.141	1.167	341	312	329
B87015	1.582	1.216	1.220	342	451	445
G82187	1.582	1.239	1.238	343	512	505

C87014	1.581	1.116	1.087	344	282	236
A83637	1.580	1.366	1.340	345	1069	938
B85640	1.578	1.289	1.365	346	693	1111
P83011	1.578	1.218	1.210	347	477	431
E84053	1.578	0.993	0.971	348	175	155
C83064	1.578	1.533	1.439	349	2600	1724
P88615	1.577	1.179	1.221	350	385	464
A83050	1.577	1.104	1.096	351	275	251
P81163	1.577	1.100	1.140	352	267	295
A89006	1.576	1.149	1.163	353	332	339
N81083	1.576	1.120	1.117	354	295	271
B85614	1.575	1.210	1.219	355	454	462
G82090	1.575	1.170	1.140	356	369	301
B86007	1.574	1.293	1.319	357	729	852
F81155	1.574	1.085	1.083	358	247	240
E84024	1.574	0.996	0.942	359	179	144
A81044	1.571	1.164	1.161	360	367	346
P81218	1.571	1.202	1.247	361	447	564
B85650	1.570	1.152	1.175	362	346	372
M84609	1.569	1.166	1.237	363	371	542
J82650	1.568	1.167	1.164	364	375	357
F81622	1.567	1.249	1.339	365	602	1017
G85082	1.567	1.117	1.155	366	301	342
Y02755	1.567	1.091	1.139	367	270	308
A81012	1.566	1.395	1.469	368	1362	2101
C84635	1.566	1.127	1.223	369	315	510
P81742	1.566	1.346	1.371	370	1028	1232
P84047	1.566	1.200	1.267	371	456	662
M83739	1.566	1.086	1.162	372	263	359
G82217	1.566	1.017	1.052	373	200	221
B86101	1.565	1.177	1.160	374	402	358
L83144	1.565	1.243	1.345	375	587	1073
C84135	1.564	1.147	1.191	376	349	415
B81066	1.563	1.167	1.291	377	387	771
J84602	1.563	1.194	1.202	378	448	448
G82726	1.562	1.225	1.242	379	537	577
P84663	1.561	1.181	1.309	380	425	871
N82670	1.560	1.263	1.356	381	667	1169
B83011	1.559	1.238	1.256	382	588	644
P92003	1.559	1.133	1.104	383	334	277
N82110	1.559	1.364	1.397	384	1180	1505
F81724	1.559	1.070	1.143	385	250	327
C84692	1.558	1.273	1.241	386	709	584
J81069	1.558	1.133	1.147	387	335	343
A89001	1.558	1.340	1.316	388	1038	923
D82065	1.558	1.211	1.141	389	501	326
L83663	1.556	1.168	1.142	390	403	336
G82622	1.555	1.094	1.186	391	291	427
G81662	1.555	1.284	1.320	392	765	961
F81696	1.552	1.170	1.237	393	417	589

P81204	1.552	1.244	1.334	394	633	1081
N83060	1.552	1.269	1.332	395	720	1074
F82665	1.552	1.061	1.095	396	241	274
H81113	1.551	1.045	0.979	397	230	176
D82049	1.551	1.270	1.196	398	733	465
F81649	1.551	1.168	1.203	399	416	493
P81709	1.550	1.393	1.519	400	1472	2833
F81052	1.549	1.316	1.268	401	965	738
P81685	1.548	1.157	1.200	402	397	495
Y02222	1.547	1.086	1.144	403	288	360
F81688	1.546	1.121	1.197	404	336	488
M85043	1.545	1.326	1.296	405	1030	882
E83034	1.544	1.035	0.996	406	226	193
A83027	1.543	1.152	1.121	407	395	318
P84048	1.543	1.384	1.380	408	1447	1488
P88624	1.543	1.156	1.230	409	408	602
K81003	1.542	1.063	1.015	410	265	208
E84033	1.542	1.065	1.071	411	266	261
N85629	1.542	1.205	1.259	412	539	734
A81070	1.541	1.186	1.272	413	481	783
P84045	1.541	1.214	1.242	414	573	661
A86022	1.540	1.128	1.128	415	360	340
M85170	1.540	1.236	1.214	416	653	553
A86038	1.540	1.413	1.386	417	1728	1573
M81062	1.540	1.252	1.221	418	701	581
P82004	1.539	1.161	1.156	419	430	393
C82643	1.539	1.000	0.952	420	210	169
F83021	1.537	1.033	1.067	421	232	260
P85601	1.537	1.267	1.360	422	773	1370
N82087	1.537	1.212	1.278	423	577	836
N81642	1.536	1.129	1.084	424	366	280
M85141	1.536	1.323	1.343	425	1082	1243
A89614	1.535	1.290	1.320	426	887	1093
K83616	1.535	1.157	1.126	427	429	347
H85110	1.535	1.055	1.080	428	262	276
N81096	1.535	1.232	1.201	429	657	540
M85746	1.534	1.162	1.223	430	442	611
P81727	1.534	1.359	1.459	431	1329	2334
C88074	1.534	1.106	1.132	432	328	364
C87610	1.534	1.304	1.398	433	979	1750
F81751	1.534	1.150	1.115	434	415	323
C82642	1.533	1.079	1.047	435	298	245
B86658	1.532	1.138	1.183	436	390	491
L81095	1.531	1.213	1.221	437	601	615
B85023	1.531	1.107	1.132	438	338	366
H81083	1.531	1.069	1.026	439	289	229
B86024	1.531	1.111	1.126	440	343	355
P81724	1.530	1.175	1.279	441	480	872
C82045	1.530	1.163	1.110	442	450	322
M92009	1.529	1.198	1.180	443	551	486

A81034	1.528	1.167	1.151	444	467	406
B85014	1.528	1.206	1.168	445	584	450
K81638	1.528	1.147	1.160	446	421	432
N82018	1.528	1.182	1.206	447	503	573
P87024	1.528	1.234	1.231	448	678	670
Y01108	1.528	1.060	1.120	449	279	353
G82231	1.527	1.112	1.096	450	354	305
Y02572	1.527	1.113	1.152	451	357	411
H85072	1.527	1.087	1.059	452	313	263
M85139	1.526	1.140	1.173	453	407	472
B85042	1.525	1.115	1.101	454	361	314
L81058	1.523	1.212	1.158	455	624	439
M85092	1.523	1.122	1.128	456	377	373
A87030	1.523	1.228	1.218	457	673	639
C83085	1.523	1.161	1.173	458	465	487
D82079	1.522	1.270	1.221	459	854	655
N81037	1.522	1.260	1.240	460	814	736
M83122	1.522	1.103	1.043	461	344	253
E85681	1.522	1.013	1.012	462	225	224
D83033	1.522	1.120	1.114	463	373	352
P86606	1.521	1.201	1.313	464	596	1150
B82046	1.521	1.139	1.094	465	418	310
J83006	1.521	1.047	1.005	466	274	218
L84029	1.520	1.213	1.182	467	637	525
P87017	1.520	1.305	1.358	468	1056	1498
P92623	1.520	1.108	1.177	469	359	506
P81683	1.519	1.060	1.081	470	292	294
A89011	1.517	1.311	1.300	471	1121	1083
L84085	1.517	1.186	1.153	472	553	440
N84034	1.516	1.293	1.344	473	1012	1416
P84041	1.516	1.185	1.272	474	556	912
P91026	1.515	1.317	1.286	475	1170	1006
B87042	1.515	1.300	1.306	476	1061	1136
M81087	1.515	1.197	1.210	477	603	637
A86011	1.515	1.367	1.316	478	1542	1210
P81022	1.514	1.164	1.183	479	489	544
P86007	1.514	1.206	1.210	480	636	640
N82633	1.514	1.326	1.395	481	1234	1893
N83045	1.514	1.302	1.330	482	1086	1314
A81018	1.514	1.427	1.366	483	2138	1633
P82008	1.514	1.330	1.305	484	1270	1144
N85625	1.513	1.298	1.354	485	1053	1520
D81061	1.513	1.180	1.203	486	545	618
A83616	1.513	1.397	1.452	487	1829	2496
H85070	1.512	1.084	1.049	488	327	268
C82660	1.512	1.027	1.066	489	256	285
F81017	1.511	1.340	1.279	490	1358	985
M85079	1.511	1.212	1.150	491	663	444
P85621	1.511	1.199	1.253	492	622	838
F82621	1.511	1.150	1.217	493	471	681

D81603	1.511	1.198	1.213	494	620	667
M83010	1.511	1.045	0.974	495	281	201
Y02610	1.511	1.023	0.982	496	251	205
Y01068	1.511	1.047	1.093	497	285	325
M81079	1.510	1.076	1.019	498	319	241
P87025	1.509	1.134	1.164	499	437	503
N82065	1.508	1.274	1.377	500	957	1778
C83042	1.508	1.231	1.218	501	742	703
K82054	1.508	1.127	1.083	502	420	316
A89008	1.507	1.320	1.365	503	1240	1695
P82014	1.506	1.093	1.136	504	358	426
J81045	1.505	1.215	1.181	505	689	563
F81743	1.505	1.161	1.150	506	509	466
Y02422	1.504	1.089	1.122	507	353	395
N81097	1.503	1.336	1.358	508	1396	1667
G82790	1.502	1.036	1.062	509	280	292
M85677	1.502	1.071	1.109	510	321	376
N82003	1.501	1.235	1.266	511	787	959
Y02753	1.501	1.289	1.406	512	1087	2126
N81122	1.500	1.111	1.092	513	400	349
B86092	1.500	1.219	1.187	514	737	604
P81160	1.499	1.167	1.212	515	549	716
Y02701	1.499	1.041	1.130	516	293	428
K84056	1.499	1.056	1.038	517	310	269
A83025	1.499	1.202	1.173	518	670	556
E82133	1.498	1.069	1.022	519	325	256
C85614	1.498	1.204	1.249	520	674	886
C85013	1.498	1.344	1.307	521	1491	1258
A82017	1.497	1.231	1.202	522	796	678
P86012	1.497	1.404	1.443	523	2085	2577
C82100	1.496	1.285	1.270	524	1093	1030
C82011	1.495	1.151	1.112	525	507	394
M84627	1.494	1.190	1.184	526	650	614
A88603	1.494	1.255	1.262	527	919	986
B84019	1.494	1.139	1.144	528	482	485
C86014	1.494	1.258	1.235	529	945	837
G82682	1.494	1.195	1.156	530	665	526
P83029	1.493	1.184	1.208	531	628	724
M84051	1.493	1.066	1.059	532	330	302
Y03364	1.492	1.130	1.191	533	464	657
L81051	1.491	1.176	1.106	534	617	390
F81110	1.491	1.288	1.290	535	1147	1192
F81707	1.490	1.027	1.102	536	286	382
K82003	1.489	1.103	1.062	537	410	313
F82660	1.488	1.160	1.160	538	566	548
C83021	1.488	1.221	1.251	539	785	949
G81102	1.488	1.056	1.016	540	322	259
N83609	1.487	1.247	1.303	541	907	1317
M86607	1.487	1.159	1.220	542	571	801
M83631	1.487	1.242	1.342	543	889	1663

M82058	1.486	1.158	1.139	544	565	496
E85061	1.486	1.076	1.134	545	362	479
P87019	1.485	1.224	1.206	546	818	742
A81049	1.485	1.235	1.304	547	868	1342
P82627	1.485	1.147	1.166	548	536	580
C86025	1.484	1.246	1.270	549	928	1107
C87013	1.484	1.258	1.253	550	999	990
L85061	1.484	1.192	1.166	551	683	583
C84688	1.484	1.244	1.297	552	917	1287
M88023	1.484	1.158	1.225	553	575	839
M88003	1.483	1.287	1.263	554	1177	1070
B87016	1.483	1.314	1.308	555	1379	1385
G81008	1.483	1.178	1.110	556	649	419
P92021	1.483	1.228	1.204	557	848	743
B83021	1.482	1.231	1.193	558	861	708
M85794	1.482	0.997	0.997	559	255	244
G82639	1.482	1.106	1.122	560	431	453
G81105	1.482	1.225	1.264	561	842	1085
B87007	1.482	1.143	1.139	562	530	513
M91009	1.481	1.338	1.291	563	1582	1260
F84713	1.481	0.951	0.968	564	211	222
M85061	1.480	1.152	1.160	565	568	576
C84087	1.480	1.217	1.146	566	810	538
M86044	1.480	1.156	1.208	567	583	772
C85024	1.479	1.061	1.087	568	345	377
B81040	1.479	1.179	1.187	569	661	690
N85031	1.479	1.327	1.380	570	1510	2090
M84608	1.479	1.292	1.312	571	1251	1457
B82106	1.478	1.253	1.303	572	1005	1388
B81620	1.478	1.185	1.291	573	679	1289
C82656	1.478	1.241	1.256	574	933	1054
M88612	1.478	1.189	1.224	575	695	861
Y02501	1.477	1.169	1.294	576	630	1324
A83634	1.477	1.305	1.245	577	1354	988
P85006	1.477	1.087	1.124	578	399	475
A89620	1.477	1.335	1.344	579	1588	1762
B83054	1.477	1.311	1.253	580	1404	1044
D82004	1.476	1.268	1.207	581	1112	788
M83668	1.476	1.026	1.039	582	302	296
A81021	1.476	1.143	1.120	583	546	463
P85012	1.476	1.129	1.164	584	500	610
N82671	1.475	1.190	1.266	585	712	1138
A83618	1.475	1.296	1.287	586	1303	1273
L83029	1.475	1.134	1.115	587	519	452
P81748	1.475	1.133	1.090	588	515	388
G82114	1.475	1.113	1.070	589	468	356
M82009	1.474	1.205	1.166	590	774	625
B81053	1.474	1.239	1.262	591	949	1120
M89606	1.474	1.010	1.046	592	283	306
N84014	1.474	1.287	1.289	593	1250	1306

P85615	1.474	1.197	1.290	594	744	1316
L83022	1.473	1.186	1.145	595	704	547
P86614	1.473	1.213	1.235	596	825	944
D82105	1.473	1.185	1.136	597	700	527
M81069	1.473	1.099	1.038	598	439	300
P82009	1.473	1.159	1.175	599	618	664
N81019	1.472	1.374	1.371	600	2015	2057
M84064	1.472	1.071	1.035	601	378	298
F84673	1.471	0.904	0.930	602	187	197
L81083	1.471	1.326	1.300	603	1570	1432
P86005	1.471	1.118	1.142	604	488	546
F82023	1.470	1.089	1.134	605	419	529
A81026	1.470	1.223	1.216	606	883	857
P92010	1.470	1.124	1.097	607	502	418
D81059	1.470	1.228	1.170	608	901	660
C83006	1.470	1.251	1.236	609	1034	974
F86624	1.470	1.018	1.006	610	300	267
E83042	1.470	1.206	1.231	611	801	942
P84054	1.470	1.174	1.242	612	671	1013
M84019	1.469	1.180	1.098	613	694	424
M85634	1.468	1.103	1.129	614	452	523
P81118	1.468	1.131	1.115	615	532	476
F81185	1.467	1.037	1.018	616	324	282
P81166	1.467	1.246	1.373	617	1020	2127
N81119	1.467	1.262	1.268	618	1127	1204
B85012	1.467	1.138	1.108	619	561	456
P84672	1.466	1.099	1.200	620	449	802
M83722	1.466	1.112	1.163	621	483	649
N82011	1.466	1.178	1.212	622	697	855
G82128	1.466	1.147	1.118	623	599	494
P85028	1.465	1.381	1.397	624	2158	2415
A81037	1.465	1.194	1.199	625	766	803
G82710	1.465	1.123	1.121	626	514	508
C83613	1.464	1.155	1.114	627	629	483
F81150	1.464	1.102	1.078	628	466	385
N81056	1.464	1.236	1.200	629	996	811
C83039	1.463	1.229	1.185	630	958	746
M85076	1.463	1.282	1.236	631	1298	1023
G85727	1.463	1.018	1.049	632	308	335
C85018	1.463	1.236	1.218	633	995	910
A83018	1.463	1.195	1.163	634	780	659
P84066	1.462	1.155	1.239	635	642	1048
N81123	1.462	1.172	1.107	636	691	467
M92019	1.461	1.154	1.179	637	641	733
A86017	1.461	1.312	1.285	638	1529	1379
N83022	1.460	1.335	1.368	639	1742	2154
H83006	1.460	1.095	1.086	640	457	414
G82757	1.459	0.989	1.072	641	278	384
A81065	1.459	1.299	1.294	642	1446	1469
M83057	1.459	1.073	1.032	643	411	312

P86016	1.458	1.270	1.299	644	1235	1514
B85645	1.458	1.192	1.216	645	792	920
D81052	1.458	1.289	1.309	646	1381	1626
Y00446	1.458	1.129	1.098	647	567	454
N82663	1.458	1.235	1.313	648	1014	1671
M85783	1.458	1.111	1.170	649	498	713
M83063	1.457	1.110	1.150	650	499	629
P83027	1.457	1.024	1.098	651	320	455
K82069	1.457	1.040	0.991	652	348	264
C85617	1.457	1.166	1.265	653	686	1247
A86040	1.457	1.283	1.306	654	1343	1606
G82103	1.456	1.093	1.061	655	461	374
C84096	1.456	1.119	1.211	656	534	906
P82021	1.455	1.142	1.184	657	621	774
A86025	1.455	1.188	1.161	658	783	684
C88069	1.455	1.163	1.181	659	682	765
P92615	1.454	1.188	1.240	660	795	1110
N82060	1.454	1.429	1.485	661	2849	3680
E86027	1.454	1.078	1.034	662	433	321
M87625	1.454	1.178	1.227	663	751	1024
Y02627	1.453	1.171	1.209	664	730	916
D81039	1.452	1.149	1.166	665	656	721
G82700	1.452	1.285	1.210	666	1398	925
G82686	1.452	1.165	1.129	667	706	568
P81749	1.451	1.052	1.192	668	380	834
H85022	1.451	1.066	1.066	669	413	389
G82141	1.451	1.111	1.088	670	522	443
A83021	1.451	1.298	1.246	671	1494	1163
M85031	1.450	1.184	1.147	672	789	650
J82085	1.450	1.240	1.249	673	1099	1193
B87012	1.449	1.121	1.125	674	564	559
M83005	1.448	1.167	1.116	675	728	543
M81076	1.448	1.061	1.042	676	406	354
G81084	1.448	1.244	1.291	677	1130	1539
P84033	1.447	1.182	1.277	678	800	1436
P83007	1.447	1.124	1.118	679	585	545
P81190	1.447	1.149	1.112	680	666	536
A87029	1.446	1.349	1.370	681	2028	2330
C82064	1.446	1.185	1.162	682	817	725
C84014	1.446	1.106	1.103	683	523	509
C81647	1.446	1.261	1.212	684	1258	968
H81672	1.445	0.972	0.939	685	276	227
G82721	1.444	1.107	1.151	686	533	687
P84020	1.444	1.130	1.154	687	619	701
M85018	1.444	1.267	1.251	688	1320	1246
P81201	1.443	1.108	1.082	689	543	447
M88015	1.443	1.080	1.106	690	462	530
B82035	1.443	1.020	0.971	691	342	258
F81675	1.442	1.148	1.181	692	675	819
M84049	1.442	1.148	1.127	693	677	592

P81119	1.442	1.178	1.146	694	805	677
M86023	1.441	1.061	1.138	695	423	645
A83020	1.441	1.212	1.189	696	986	873
P81771	1.441	1.134	1.207	697	644	979
E81612	1.440	1.044	1.049	698	389	379
P92042	1.440	1.313	1.295	699	1721	1666
B82022	1.440	1.095	1.082	700	504	459
B81072	1.439	1.087	1.220	701	486	1072
F81741	1.439	1.291	1.308	702	1538	1782
L83008	1.439	1.282	1.282	703	1473	1538
A87006	1.439	1.058	1.057	704	422	399
L83088	1.438	1.180	1.159	705	834	744
C87617	1.438	1.391	1.467	706	2574	3637
M81092	1.438	1.089	1.111	707	493	549
B81081	1.437	1.140	1.264	708	669	1409
F86707	1.437	1.038	1.073	709	382	441
M83706	1.436	1.238	1.273	710	1171	1483
J81648	1.436	1.064	1.144	711	443	692
F81049	1.436	1.097	1.047	712	527	383
C81655	1.435	1.185	1.165	713	864	776
E87751	1.435	1.132	1.166	714	654	789
P81171	1.435	1.129	1.086	715	646	492
F81177	1.435	1.084	1.089	716	490	501
F82675	1.435	1.010	0.986	717	337	283
P84019	1.435	1.279	1.266	718	1480	1448
M85642	1.434	1.059	1.069	719	434	438
C88020	1.434	1.428	1.394	720	3080	2731
G81096	1.434	1.139	1.186	721	672	887
P91003	1.434	1.094	1.120	722	525	600
P84067	1.433	1.150	1.130	723	719	646
J82021	1.433	1.200	1.120	724	961	601
L81099	1.433	1.218	1.182	725	1063	875
B81015	1.433	1.151	1.154	726	726	750
A83066	1.432	1.252	1.233	727	1301	1201
M89008	1.432	1.278	1.179	728	1487	865
B86054	1.431	1.412	1.433	729	2906	3265
Y02461	1.431	1.179	1.217	730	853	1108
G82086	1.431	1.210	1.205	731	1023	1033
P92633	1.431	1.102	1.117	732	563	591
M87012	1.431	1.146	1.164	733	711	797
G82706	1.430	1.059	1.166	734	444	807
G82729	1.430	1.166	1.152	735	803	752
P87618	1.430	1.405	1.489	736	2847	4056
N81012	1.430	1.229	1.208	737	1152	1060
C82091	1.429	1.164	1.169	738	797	827
B87026	1.429	1.363	1.312	739	2361	1908
F82627	1.428	1.075	1.071	740	484	460
A89020	1.428	1.295	1.330	741	1662	2087
C84647	1.428	1.147	1.227	742	735	1187
H85054	1.427	1.079	1.030	743	496	368

B86050	1.427	1.117	1.106	744	626	572
P85004	1.427	1.199	1.290	745	992	1739
B87023	1.427	1.218	1.230	746	1107	1220
B82086	1.427	1.089	1.101	747	535	554
G83641	1.426	1.074	1.123	748	487	643
A81063	1.426	1.298	1.330	749	1711	2110
N81054	1.426	1.213	1.205	750	1071	1058
F81011	1.426	1.121	1.103	751	648	566
N85619	1.426	1.246	1.284	752	1302	1691
B87011	1.426	1.291	1.258	753	1642	1451
P81622	1.425	1.088	1.116	754	531	619
A81613	1.425	1.128	1.187	755	668	945
P92026	1.425	1.152	1.134	756	757	700
B87033	1.424	1.228	1.240	757	1176	1308
P81707	1.424	1.154	1.183	758	770	929
M82039	1.424	1.154	1.115	759	771	621
M85770	1.424	1.175	1.221	760	875	1176
N81011	1.424	1.147	1.126	761	743	666
A83019	1.423	1.315	1.322	762	1913	2059
C86614	1.423	1.222	1.208	763	1151	1101
N84024	1.422	1.139	1.107	764	721	594
N85648	1.421	1.177	1.236	765	892	1303
M83717	1.421	1.057	1.177	766	459	914
C83079	1.421	1.155	1.232	767	791	1271
L84068	1.421	1.191	1.162	768	977	835
F82011	1.421	1.103	1.064	769	609	461
A81030	1.420	1.139	1.151	770	734	791
D81029	1.420	1.082	1.065	771	529	469
D81605	1.420	1.201	1.253	772	1032	1459
G85717	1.420	1.004	1.083	773	352	531
M83021	1.419	1.262	1.270	774	1467	1617
C83019	1.419	1.387	1.324	775	2755	2116
J84011	1.419	1.088	1.072	776	550	498
A81057	1.419	1.173	1.147	777	886	773
E81023	1.419	1.075	1.078	778	508	521
P92652	1.419	1.231	1.266	779	1238	1580
P81690	1.419	1.284	1.339	780	1649	2290
B85641	1.418	1.207	1.242	781	1090	1378
A83017	1.418	1.312	1.243	782	1922	1386
M83062	1.418	1.134	1.105	783	718	606
D83008	1.418	1.057	1.039	784	473	405
A82032	1.418	1.318	1.258	785	1997	1521
A83075	1.417	1.205	1.193	786	1081	1041
Y02521	1.417	1.156	1.187	787	819	993
G82119	1.417	1.168	1.139	788	870	751
D83060	1.417	1.130	1.068	789	705	489
C82026	1.417	1.096	1.072	790	590	502
B83007	1.417	1.095	1.121	791	586	673
C86033	1.417	1.365	1.395	792	2513	2955
B83005	1.416	1.081	1.092	793	542	561

J81071	1.416	1.132	1.114	794	716	656
B82064	1.415	1.139	1.109	795	747	632
H81126	1.415	1.032	1.001	796	414	332
M83004	1.415	1.141	1.156	797	756	833
M85051	1.415	1.086	1.120	798	562	680
C86024	1.414	1.254	1.242	799	1439	1413
F82676	1.414	1.154	1.174	800	824	939
M83022	1.414	1.081	1.036	801	548	407
H85693	1.413	1.099	1.052	802	616	446
P85005	1.413	1.147	1.160	803	788	864
C85622	1.413	1.269	1.338	804	1576	2340
Y03597	1.413	1.085	1.108	805	570	636
L83072	1.413	1.133	1.186	806	738	1022
C85030	1.413	1.229	1.196	807	1274	1090
P92006	1.413	1.236	1.240	808	1319	1411
H82033	1.412	1.025	1.010	809	405	363
N85017	1.412	1.209	1.258	810	1141	1562
P86609	1.412	1.064	1.119	811	495	686
Y00683	1.412	1.350	1.298	812	2407	1939
C83015	1.412	1.140	1.087	813	760	558
Y02754	1.411	1.021	0.998	814	398	338
M85128	1.411	1.207	1.194	815	1128	1087
N82623	1.411	1.304	1.370	816	1915	2724
A81008	1.410	1.391	1.425	817	2921	3451
K81048	1.410	1.048	1.059	818	463	481
B85636	1.410	1.092	1.096	819	606	597
Y01652	1.410	1.240	1.201	820	1365	1140
B85652	1.410	1.206	1.226	821	1136	1321
F82034	1.410	1.023	1.094	822	409	587
F81608	1.410	1.146	1.076	823	807	539
A81052	1.409	1.236	1.196	824	1346	1115
G82197	1.409	0.980	0.983	825	326	311
F81096	1.409	1.181	1.100	826	993	620
C84658	1.409	1.307	1.285	827	1960	1849
B84623	1.409	1.089	1.086	828	595	565
H82088	1.409	1.119	1.126	829	690	732
N85016	1.409	1.222	1.223	830	1248	1302
L84070	1.409	1.164	1.132	831	893	755
D81011	1.409	1.208	1.223	832	1153	1297
H81022	1.408	1.088	1.055	833	597	473
K82059	1.408	1.154	1.168	834	850	934
N84621	1.408	1.343	1.349	835	2374	2515
G81055	1.408	1.117	1.061	836	687	497
F81082	1.407	1.168	1.144	837	921	814
C88077	1.407	1.228	1.254	838	1304	1584
B84010	1.407	1.171	1.161	839	948	902
M83070	1.407	1.034	1.004	840	441	362
N81007	1.406	1.124	1.104	841	724	653
F81022	1.406	1.076	1.027	842	558	403
B81046	1.406	1.271	1.342	843	1646	2463

A83603	1.406	1.318	1.328	844	2136	2313
A81053	1.405	1.210	1.166	845	1182	941
C86001	1.405	1.326	1.280	846	2217	1845
C81056	1.405	1.302	1.261	847	1950	1669
H81011	1.405	1.062	1.016	848	511	381
B85016	1.405	1.145	1.136	849	823	794
N81095	1.404	1.190	1.122	850	1065	735
D82053	1.404	1.560	1.466	851	5229	4084
K82068	1.404	1.074	1.014	852	557	380
P81016	1.404	1.310	1.361	853	2068	2698
C88087	1.404	1.433	1.529	854	3534	4883
G82057	1.404	1.215	1.150	855	1230	860
G83655	1.404	1.147	1.203	856	837	1194
M85116	1.403	1.073	1.024	857	555	401
B81034	1.403	1.112	1.094	858	684	622
A81066	1.403	1.160	1.161	859	896	922
B81003	1.403	1.153	1.154	860	871	883
B81682	1.403	1.213	1.349	861	1221	2576
M87628	1.403	0.993	1.029	862	363	417
A81003	1.403	1.303	1.349	863	1988	2573
A83032	1.403	1.300	1.326	864	1957	2318
C84138	1.402	1.114	1.227	865	699	1390
D83017	1.402	1.101	1.087	866	660	590
P88044	1.402	1.013	1.027	867	401	412
C85017	1.401	1.378	1.348	868	2862	2579
L82044	1.401	1.256	1.237	869	1569	1477
F81168	1.401	1.192	1.186	870	1108	1105
N84021	1.401	1.113	1.087	871	702	595
Y01851	1.401	1.188	1.286	872	1083	1928
D81062	1.401	1.144	1.111	873	841	702
K83033	1.400	1.093	1.152	874	639	888
M86045	1.400	1.187	1.180	875	1080	1076
N85027	1.399	1.185	1.183	876	1066	1096
F81051	1.398	1.220	1.158	877	1309	935
M83682	1.398	1.232	1.334	878	1401	2468
E82042	1.397	1.115	1.082	879	727	588
A89017	1.397	1.181	1.163	880	1048	975
P81770	1.396	1.004	1.070	881	394	555
C83063	1.396	1.236	1.175	882	1448	1063
A81060	1.396	1.159	1.209	883	937	1295
A82075	1.396	1.211	1.228	884	1267	1458
A89003	1.395	1.230	1.219	885	1407	1381
E85056	1.395	1.068	1.016	886	574	404
M83032	1.394	1.130	1.096	887	802	663
C83026	1.394	1.177	1.145	888	1043	881
G82115	1.394	1.029	1.012	889	458	397
P81008	1.394	1.281	1.316	890	1863	2310
C83605	1.394	1.126	1.164	891	782	1001
L84052	1.394	1.190	1.145	892	1139	889
D81057	1.393	1.118	1.089	893	750	641

F82004	1.393	1.143	1.210	894	865	1327
B81038	1.393	1.283	1.287	895	1892	2009
C84637	1.393	1.143	1.185	896	872	1147
G83033	1.392	1.068	1.025	897	580	433
C82631	1.392	1.054	1.045	898	526	500
K84010	1.392	1.076	1.026	899	611	436
C88073	1.392	1.236	1.285	900	1479	1993
E82078	1.392	1.143	1.114	901	874	748
P91032	1.392	1.071	1.080	902	591	609
B82032	1.391	1.160	1.115	903	970	756
F81669	1.391	1.162	1.140	904	987	874
M88036	1.391	1.066	1.130	905	581	824
C86013	1.390	1.159	1.154	906	971	956
A83052	1.390	1.240	1.224	907	1525	1462
P81074	1.390	1.278	1.295	908	1870	2121
G82225	1.390	1.190	1.138	909	1159	870
D83020	1.390	1.049	0.990	910	521	365
N82078	1.390	1.334	1.391	911	2467	3266
H84049	1.390	1.060	1.088	912	560	654
K82040	1.389	1.134	1.086	913	849	648
Y00592	1.389	1.260	1.245	914	1704	1675
N81023	1.389	1.191	1.132	915	1168	845
G81641	1.389	1.072	1.190	916	608	1206
B86055	1.389	1.185	1.170	917	1138	1080
N81061	1.389	1.141	1.077	918	876	607
A81020	1.389	1.130	1.162	919	831	1028
P87630	1.388	1.265	1.339	920	1761	2636
A83076	1.388	1.330	1.309	921	2437	2289
M84615	1.388	1.153	1.142	922	944	898
M83014	1.388	1.227	1.233	923	1435	1561
F85650	1.387	1.038	1.068	924	492	578
G82605	1.387	1.080	1.061	925	640	552
B84613	1.387	1.335	1.348	926	2509	2743
L84011	1.386	1.147	1.108	927	913	747
P85013	1.386	1.100	1.094	928	708	691
B87005	1.386	1.247	1.231	929	1614	1557
C82120	1.386	1.176	1.139	930	1100	894
P87625	1.386	1.358	1.482	931	2807	4550
J82086	1.385	1.339	1.344	932	2586	2717
D81018	1.385	1.111	1.077	933	755	628
C88030	1.385	1.238	1.324	934	1547	2502
Y00399	1.385	1.211	1.246	935	1339	1720
E85120	1.385	1.036	1.030	936	494	468
Y01118	1.385	1.133	1.213	937	857	1423
K81086	1.385	1.121	1.087	938	804	668
F81055	1.384	1.015	0.947	939	445	297
G82050	1.384	1.054	1.017	940	559	435
F82053	1.384	1.022	1.041	941	469	511
P88014	1.384	1.216	1.235	942	1389	1629
G82093	1.383	1.133	1.101	943	862	731

M82020	1.383	1.128	1.099	944	845	727
E82012	1.383	1.052	1.024	945	554	457
P88620	1.383	1.101	1.188	946	725	1231
F81126	1.383	1.181	1.153	947	1145	997
F85059	1.383	1.077	1.085	948	647	665
Y00164	1.383	1.196	1.180	949	1241	1181
G85008	1.383	1.067	1.125	950	613	844
B83045	1.382	1.174	1.170	951	1111	1117
G81063	1.382	1.138	1.207	952	895	1392
E82090	1.382	1.046	1.021	953	541	449
Y02676	1.382	0.906	0.972	954	271	345
P85622	1.382	1.150	1.175	955	972	1155
C84032	1.382	1.191	1.144	956	1212	947
G82077	1.381	1.218	1.189	957	1427	1251
J82084	1.380	1.044	1.086	958	540	682
G82109	1.380	1.112	1.197	959	779	1329
A83041	1.380	1.242	1.193	960	1622	1288
C84680	1.380	1.200	1.326	961	1299	2581
N83006	1.380	1.235	1.323	962	1571	2541
J82120	1.379	1.116	1.083	963	806	672
P81713	1.379	1.143	1.265	964	942	1933
Y02624	1.379	1.051	1.133	965	572	904
A89616	1.379	1.148	1.234	966	976	1664
M88628	1.378	1.134	1.196	967	894	1328
J82040	1.378	1.197	1.169	968	1288	1141
F82624	1.378	1.077	1.089	969	658	709
L82003	1.378	1.144	1.137	970	955	930
M85803	1.378	1.022	1.065	971	479	605
F81106	1.378	1.157	1.149	972	1025	1003
G82232	1.378	1.070	1.087	973	638	698
D81007	1.377	1.108	1.106	974	772	775
H85067	1.377	1.154	1.114	975	1016	810
P91019	1.377	1.445	1.402	976	4088	3573
A81043	1.377	1.196	1.191	977	1293	1300
M83703	1.377	1.122	1.145	978	847	987
G82066	1.377	1.236	1.215	979	1593	1499
N83612	1.376	1.254	1.318	980	1767	2526
B85655	1.376	1.305	1.351	981	2285	2904
M89602	1.376	1.357	1.348	982	2919	2871
P92605	1.376	1.072	1.087	983	651	711
C83643	1.376	1.338	1.312	984	2696	2461
L81642	1.376	1.095	1.087	985	736	710
K81030	1.375	1.120	1.088	986	844	715
N84017	1.375	1.221	1.181	987	1486	1235
M86617	1.375	1.140	1.177	988	947	1214
N82650	1.374	1.278	1.402	989	2036	3622
F81211	1.374	1.066	1.081	990	631	683
P87668	1.374	1.192	1.317	991	1281	2530
P85008	1.374	1.230	1.245	992	1574	1802
P92647	1.374	1.185	1.205	993	1224	1447

G82184	1.374	1.115	1.202	994	826	1422
M83601	1.374	1.185	1.248	995	1226	1833
M85023	1.374	1.206	1.162	996	1388	1126
L83068	1.374	1.148	1.104	997	1006	780
N83014	1.373	1.206	1.220	998	1390	1574
K82019	1.373	1.125	1.066	999	882	633
P87014	1.373	1.115	1.096	1000	836	753
F84047	1.372	1.064	1.048	1001	634	560
A88010	1.372	1.300	1.339	1002	2276	2801
N83621	1.372	1.251	1.318	1003	1775	2557
A83074	1.372	1.210	1.169	1004	1432	1173
M88600	1.372	1.116	1.176	1005	840	1225
N83005	1.372	1.236	1.216	1006	1640	1546
M85053	1.372	1.163	1.211	1007	1106	1510
P84690	1.372	1.104	1.193	1008	781	1355
N83028	1.371	1.222	1.234	1009	1530	1736
A89010	1.370	1.183	1.141	1010	1247	1000
P81091	1.370	1.288	1.262	1011	2178	1987
N83023	1.370	1.377	1.366	1012	3240	3183
P81147	1.370	1.252	1.363	1013	1810	3152
N83024	1.369	1.196	1.155	1014	1338	1103
B84612	1.369	1.120	1.102	1015	869	796
N84027	1.369	1.101	1.204	1016	778	1466
F81181	1.369	1.045	1.028	1017	578	517
Y02567	1.369	1.102	1.135	1018	786	967
H85063	1.369	1.121	1.063	1019	877	635
A81017	1.369	1.080	1.060	1020	696	624
L83121	1.369	1.106	1.085	1021	813	728
L83651	1.369	1.305	1.317	1022	2383	2605
E86620	1.368	0.994	0.992	1023	438	410
L83028	1.368	1.218	1.214	1024	1520	1567
M84022	1.368	1.123	1.073	1025	890	674
Y00437	1.368	1.003	0.986	1026	453	396
H85023	1.368	1.152	1.139	1027	1051	1002
M88001	1.367	1.156	1.215	1028	1091	1589
B83034	1.367	1.193	1.278	1029	1342	2192
G82053	1.367	1.193	1.166	1030	1335	1195
P86017	1.367	1.279	1.313	1031	2129	2571
B84006	1.366	1.268	1.242	1032	2008	1847
G82083	1.366	1.128	1.086	1033	929	741
M83016	1.366	1.157	1.087	1034	1104	745
E86007	1.366	1.124	1.084	1035	904	737
B87009	1.366	1.363	1.356	1036	3126	3106
F81221	1.365	1.126	1.114	1037	915	876
G81004	1.365	1.232	1.161	1038	1671	1170
M88007	1.365	1.239	1.211	1039	1740	1575
G82708	1.364	1.033	1.091	1040	552	768
C84012	1.364	1.231	1.218	1041	1669	1651
D81611	1.363	1.197	1.217	1042	1400	1650
A87017	1.363	1.282	1.244	1043	2191	1890

P81633	1.363	1.211	1.310	1044	1501	2593
P89004	1.363	1.086	1.087	1045	741	757
J82630	1.363	1.047	1.022	1046	614	519
M88646	1.362	1.054	1.152	1047	635	1129
B83063	1.361	1.228	1.250	1048	1661	1965
F81026	1.361	1.200	1.164	1049	1437	1223
G82148	1.361	1.114	1.142	1050	884	1075
E85096	1.361	0.988	0.988	1051	440	420
P81755	1.361	1.297	1.357	1052	2385	3196
A81031	1.360	1.214	1.210	1053	1554	1611
M83715	1.360	1.171	1.127	1054	1222	981
C88014	1.360	1.285	1.244	1055	2251	1921
C81087	1.360	1.142	1.138	1056	1040	1053
A89018	1.360	1.309	1.356	1057	2523	3189
D81019	1.360	1.148	1.195	1058	1085	1468
A81040	1.360	1.179	1.147	1059	1290	1118
E82655	1.359	1.145	1.139	1060	1060	1067
N84617	1.359	1.058	1.084	1061	659	758
A86601	1.359	1.217	1.194	1062	1587	1474
M81046	1.359	1.134	1.091	1063	1008	798
E81632	1.359	1.124	1.148	1064	952	1127
H85092	1.358	0.968	1.006	1065	396	477
G81022	1.358	1.122	1.110	1066	938	885
E81040	1.358	1.093	1.083	1067	798	759
M83709	1.358	1.048	1.125	1068	627	982
P82609	1.357	1.148	1.152	1069	1103	1160
C83013	1.357	1.181	1.123	1070	1324	970
L83032	1.357	1.091	1.083	1071	790	764
F81652	1.357	1.109	1.122	1072	878	960
A83001	1.357	1.131	1.097	1073	1000	831
D83023	1.357	1.165	1.131	1074	1206	1031
A81622	1.357	1.356	1.418	1075	3150	4072
G83063	1.357	1.098	1.157	1076	827	1196
A83004	1.357	1.178	1.187	1077	1305	1438
D83034	1.357	1.233	1.201	1078	1759	1552
M82017	1.356	1.120	1.091	1079	941	804
P86008	1.356	1.234	1.261	1080	1770	2114
A82052	1.356	1.086	1.099	1081	768	843
H84054	1.356	1.078	1.056	1082	740	658
M85071	1.356	1.139	1.109	1083	1044	896
C88086	1.356	1.280	1.369	1084	2235	3425
G82044	1.356	1.118	1.087	1085	926	784
C83056	1.356	1.194	1.207	1086	1431	1625
M83069	1.356	1.107	1.069	1087	873	718
B81120	1.356	1.150	1.139	1088	1123	1088
A81032	1.355	1.140	1.108	1089	1050	892
K83037	1.355	1.126	1.085	1090	981	782
N82053	1.355	1.178	1.167	1091	1317	1279
Y04266	1.355	1.120	1.141	1092	951	1109
C88026	1.354	1.117	1.135	1093	932	1079

C84705	1.354	1.203	1.214	1094	1507	1708
P91006	1.354	1.253	1.252	1095	1965	2044
A86023	1.354	1.168	1.211	1096	1253	1682
F81641	1.354	1.088	1.059	1097	794	675
M83719	1.354	1.079	1.129	1098	752	1036
B86060	1.354	1.205	1.240	1099	1535	1941
D83056	1.353	1.164	1.149	1100	1227	1165
A83049	1.353	1.188	1.163	1101	1408	1261
B81689	1.353	1.100	1.200	1102	851	1576
M83143	1.353	1.268	1.410	1103	2153	4032
P91012	1.353	1.186	1.175	1104	1397	1367
A82016	1.353	1.116	1.111	1105	936	927
B82001	1.352	1.220	1.225	1106	1672	1823
C86006	1.352	1.203	1.276	1107	1528	2321
M85739	1.352	1.002	1.025	1108	491	550
P82007	1.352	1.227	1.190	1109	1748	1506
F81056	1.351	1.261	1.268	1110	2105	2248
L81670	1.351	1.311	1.252	1111	2654	2081
F85060	1.351	0.972	1.037	1112	426	593
D81042	1.351	1.120	1.076	1113	973	763
M81077	1.351	1.082	1.097	1114	777	862
H81111	1.351	0.987	0.935	1115	460	328
M85159	1.351	1.153	1.198	1116	1169	1583
P82011	1.350	1.174	1.206	1117	1326	1674
E85663	1.350	0.945	0.978	1118	368	421
D81629	1.350	1.121	1.142	1119	990	1148
C88096	1.350	1.239	1.210	1120	1888	1718
P82013	1.350	1.288	1.354	1121	2406	3301
Y03661	1.350	1.116	1.073	1122	960	754
D81027	1.350	1.136	1.105	1123	1075	913
A81054	1.349	1.233	1.212	1124	1828	1743
F81029	1.349	1.115	1.145	1125	954	1171
P92621	1.349	1.282	1.326	1126	2354	2933
P81123	1.349	1.224	1.240	1127	1743	1984
P81180	1.349	1.277	1.236	1128	2281	1947
P81674	1.349	1.116	1.115	1129	968	971
M85107	1.348	1.083	1.156	1130	793	1244
C86016	1.348	1.316	1.306	1131	2756	2706
J82622	1.348	1.174	1.197	1132	1333	1600
A81042	1.348	1.300	1.283	1133	2556	2446
J83040	1.348	1.126	1.096	1134	1018	867
C82624	1.348	1.105	1.114	1135	900	976
J83055	1.348	1.156	1.107	1136	1208	932
B82024	1.348	1.331	1.327	1137	2947	2962
G82777	1.347	1.003	1.044	1138	506	647
C82017	1.347	1.121	1.082	1139	1002	808
J83023	1.347	1.160	1.113	1140	1243	966
L84013	1.346	1.114	1.086	1141	966	828
G85736	1.346	1.120	1.172	1142	1004	1398
E81617	1.346	1.096	1.102	1143	867	918

L83013	1.346	1.155	1.131	1144	1211	1106
M85155	1.345	1.227	1.229	1145	1812	1922
J82150	1.345	1.115	1.081	1146	980	809
N85021	1.345	1.227	1.297	1147	1813	2656
F85003	1.345	1.052	1.009	1148	680	532
M82026	1.344	1.110	1.082	1149	956	818
H85665	1.344	1.111	1.119	1150	962	1034
B81008	1.344	1.174	1.201	1151	1369	1683
N82070	1.344	1.325	1.359	1152	2925	3461
F82018	1.343	1.049	1.030	1153	676	596
B85015	1.343	1.234	1.220	1154	1899	1854
G81014	1.343	1.040	1.051	1155	655	693
A82646	1.343	1.430	1.401	1156	4371	4045
M81058	1.342	1.174	1.116	1157	1383	1027
N82668	1.342	1.109	1.198	1158	967	1673
M83074	1.342	1.082	1.020	1159	822	569
P87620	1.342	1.208	1.210	1160	1650	1777
K81066	1.342	0.982	0.947	1161	475	371
E82032	1.342	1.091	1.052	1162	858	704
B85027	1.342	1.057	1.050	1163	714	695
C84679	1.342	1.290	1.408	1164	2514	4162
C83045	1.341	1.272	1.236	1165	2308	2010
F81053	1.341	1.083	1.019	1166	832	571
G81059	1.341	1.229	1.167	1167	1880	1399
A81629	1.341	1.274	1.287	1168	2343	2586
M91034	1.341	1.356	1.381	1169	3348	3784
M85024	1.341	1.140	1.184	1170	1154	1537
A86026	1.341	1.193	1.187	1171	1541	1572
B81099	1.341	1.133	1.140	1172	1113	1189
A82064	1.341	1.460	1.442	1173	4791	4609
N83019	1.341	1.155	1.143	1174	1259	1217
N81113	1.340	1.210	1.150	1175	1691	1259
M84045	1.340	1.120	1.077	1176	1029	813
P82616	1.340	1.111	1.188	1177	982	1591
M91003	1.340	1.150	1.175	1178	1220	1471
A86041	1.340	1.123	1.112	1179	1045	1014
M91623	1.340	1.231	1.340	1180	1908	3255
M86011	1.339	1.300	1.350	1181	2664	3403
K82067	1.339	1.011	0.989	1182	569	484
C88023	1.339	1.173	1.160	1183	1406	1359
C85016	1.339	1.278	1.235	1184	2419	2034
G82055	1.338	1.065	1.047	1185	758	699
B85037	1.338	1.128	1.139	1186	1098	1202
C83049	1.338	1.311	1.243	1187	2815	2120
A83026	1.338	1.180	1.158	1188	1462	1344
B86104	1.337	1.143	1.237	1189	1188	2060
B81025	1.337	1.387	1.300	1190	3821	2761
N84012	1.337	1.111	1.081	1191	1001	848
M84006	1.337	1.101	1.014	1192	946	570
C82018	1.337	1.147	1.133	1193	1225	1175

B83614	1.336	1.196	1.291	1194	1597	2670
E81048	1.336	1.084	1.082	1195	852	859
C84021	1.336	1.234	1.186	1196	1964	1613
L83074	1.336	1.207	1.187	1197	1696	1623
M86627	1.336	1.110	1.184	1198	998	1592
K81015	1.336	1.045	1.009	1199	685	551
C83036	1.336	1.202	1.161	1200	1665	1395
P82016	1.336	1.123	1.108	1201	1079	1018
A87012	1.335	1.213	1.181	1202	1773	1569
M83028	1.335	1.178	1.225	1203	1470	1969
F82038	1.335	1.145	1.216	1204	1218	1886
G82180	1.335	1.100	1.143	1205	953	1255
A88004	1.335	1.229	1.184	1206	1925	1610
C87017	1.335	1.150	1.129	1207	1261	1157
P81127	1.335	1.206	1.161	1208	1707	1408
C88036	1.334	1.182	1.183	1209	1504	1604
N84614	1.334	1.076	1.089	1210	835	909
M85027	1.334	1.177	1.116	1211	1468	1084
C84009	1.334	1.098	1.060	1212	943	767
G82147	1.334	1.211	1.192	1213	1768	1696
P81130	1.334	1.161	1.145	1214	1355	1274
F85025	1.334	1.027	1.008	1215	645	557
F81018	1.333	1.283	1.218	1216	2534	1927
N82086	1.333	1.277	1.355	1217	2460	3546
P82036	1.333	1.260	1.317	1218	2269	3030
Y02794	1.333	1.017	1.060	1219	615	770
C83027	1.332	1.189	1.183	1220	1579	1619
F81016	1.332	1.116	1.116	1221	1046	1094
N82676	1.332	1.007	1.099	1222	576	977
M83045	1.332	1.057	0.979	1223	753	478
P81169	1.332	1.137	1.120	1224	1184	1122
C83633	1.332	1.059	1.021	1225	759	612
F81067	1.332	1.091	1.070	1226	909	820
C85033	1.332	1.121	1.133	1227	1092	1205
N81049	1.332	1.206	1.154	1228	1734	1369
A88007	1.332	1.193	1.147	1229	1621	1311
P87634	1.331	1.180	1.226	1230	1514	2013
C84660	1.331	1.020	0.991	1231	625	522
P84051	1.331	1.138	1.163	1232	1195	1454
B81619	1.331	1.102	1.079	1233	984	868
D83012	1.331	1.167	1.146	1234	1424	1313
M83073	1.330	1.149	1.069	1235	1296	823
P84021	1.330	1.126	1.138	1236	1137	1254
D83055	1.329	1.231	1.206	1237	2010	1853
N81048	1.329	1.159	1.113	1238	1368	1095
J82125	1.329	1.057	1.022	1239	764	630
Y01221	1.329	1.087	1.128	1240	905	1191
E84004	1.329	1.017	0.952	1241	623	408
B87027	1.329	1.103	1.104	1242	1003	1039
L84027	1.328	1.052	1.037	1243	745	696

L82042	1.328	1.213	1.177	1244	1830	1602
K81083	1.328	1.007	0.975	1245	592	474
C82029	1.328	1.271	1.255	1246	2456	2360
N84002	1.328	1.323	1.412	1247	3095	4383
A88012	1.327	1.320	1.259	1248	3062	2407
B86081	1.327	1.119	1.144	1249	1109	1325
H81058	1.327	1.037	0.985	1250	692	512
K82615	1.327	1.020	1.014	1251	643	603
P85014	1.327	1.153	1.256	1252	1337	2391
A89032	1.327	1.209	1.187	1253	1809	1719
D81020	1.327	1.129	1.112	1254	1172	1099
D82104	1.326	1.120	1.096	1255	1118	995
P86014	1.326	1.176	1.196	1256	1523	1792
K84062	1.326	1.075	1.064	1257	863	817
F84004	1.326	1.121	1.056	1258	1129	778
E83668	1.326	0.927	0.941	1259	384	391
P86009	1.326	1.198	1.207	1260	1720	1900
Y01165	1.325	1.147	1.115	1261	1307	1131
G83064	1.325	1.021	1.015	1262	652	616
D82080	1.325	1.198	1.178	1263	1723	1643
P81031	1.325	1.109	1.059	1264	1054	805
P85015	1.325	1.158	1.168	1265	1395	1542
F81048	1.325	1.062	1.017	1266	811	627
P91020	1.325	1.146	1.203	1267	1310	1868
A82631	1.324	1.145	1.143	1268	1306	1337
G82028	1.324	1.164	1.087	1269	1441	951
P81684	1.324	1.271	1.403	1270	2499	4333
F81073	1.324	1.147	1.173	1271	1321	1607
A81022	1.324	1.212	1.181	1272	1872	1688
G82076	1.324	1.139	1.108	1273	1262	1092
F86652	1.323	0.990	0.927	1274	544	370
B82031	1.323	1.119	1.072	1275	1131	877
A83014	1.323	1.187	1.157	1276	1636	1460
K81067	1.323	1.101	1.095	1277	1017	1010
L82014	1.323	1.194	1.155	1278	1708	1455
Y00774	1.322	1.259	1.224	1279	2395	2096
H85053	1.321	1.002	1.001	1280	600	575
A88002	1.321	1.306	1.277	1281	2961	2681
D81060	1.321	1.137	1.092	1282	1273	1005
N84041	1.321	1.129	1.113	1283	1198	1146
M92627	1.321	1.254	1.328	1284	2353	3338
E82040	1.320	1.085	1.053	1285	950	799
B87028	1.320	1.272	1.284	1286	2561	2776
Y00078	1.320	1.073	1.001	1287	880	579
P81750	1.320	1.092	1.146	1288	991	1403
L83006	1.320	1.278	1.301	1289	2634	3005
M83065	1.320	1.141	1.074	1290	1308	900
B86070	1.320	1.258	1.357	1291	2412	3748
F81618	1.320	1.316	1.311	1292	3123	3127
P82652	1.319	1.303	1.384	1293	2955	4134

B83033	1.319	1.201	1.183	1294	1807	1751
G82650	1.319	1.120	1.164	1295	1165	1559
N83030	1.319	1.087	1.166	1296	969	1577
N83035	1.319	1.386	1.450	1297	4078	4976
C83628	1.318	1.089	1.095	1298	988	1049
A82022	1.318	1.097	1.100	1299	1022	1082
L84045	1.318	1.118	1.060	1300	1160	842
Y02129	1.318	1.223	1.261	1301	2050	2535
B86014	1.318	1.149	1.143	1302	1378	1394
A82024	1.318	1.202	1.193	1303	1827	1850
B86020	1.317	1.134	1.138	1304	1272	1351
N81114	1.317	1.133	1.095	1305	1264	1055
A83022	1.317	1.187	1.159	1306	1692	1533
N81111	1.317	1.134	1.083	1307	1277	973
G82712	1.317	1.040	1.062	1308	746	851
A82037	1.316	1.222	1.167	1309	2058	1616
N81045	1.316	1.091	1.065	1310	1009	878
D81040	1.316	1.228	1.170	1311	2131	1654
G82026	1.316	1.124	1.106	1312	1207	1130
L82023	1.316	1.176	1.143	1313	1602	1410
A86030	1.316	1.247	1.286	1314	2330	2845
F81130	1.315	1.109	1.059	1315	1120	847
A82044	1.315	1.228	1.217	1316	2134	2094
J83019	1.315	1.123	1.080	1317	1201	964
B82042	1.314	1.175	1.141	1318	1605	1407
P83019	1.314	1.183	1.213	1319	1683	2054
J82117	1.314	1.259	1.265	1320	2477	2639
G82158	1.314	1.044	1.027	1321	769	717
C86039	1.314	1.167	1.150	1322	1550	1481
A82012	1.314	1.206	1.196	1323	1911	1911
M81037	1.314	1.144	1.099	1324	1370	1102
C88045	1.313	1.149	1.121	1325	1413	1245
P81044	1.313	1.257	1.186	1326	2461	1825
P88020	1.313	1.125	1.103	1327	1231	1132
B83611	1.313	1.177	1.240	1328	1635	2357
D83010	1.313	1.282	1.232	1329	2773	2274
B82092	1.313	1.214	1.228	1330	2000	2242
J83044	1.313	1.089	1.060	1331	1013	869
A83015	1.312	1.176	1.148	1332	1638	1475
C83007	1.312	1.094	1.071	1333	1041	928
D82003	1.312	1.131	1.109	1334	1297	1179
J81633	1.312	1.171	1.141	1335	1600	1427
E84005	1.312	1.054	0.968	1336	833	504
P92004	1.311	1.135	1.109	1337	1318	1183
E85744	1.311	0.994	0.974	1338	610	528
E84040	1.311	0.979	0.943	1339	547	430
J82210	1.311	1.188	1.117	1340	1756	1234
B87019	1.311	1.207	1.217	1341	1938	2135
D81074	1.311	1.040	1.028	1342	767	730
F81691	1.311	1.060	1.092	1343	859	1078

P81681	1.311	1.269	1.314	1344	2648	3302
D81619	1.310	1.112	1.106	1345	1167	1166
B86009	1.310	1.151	1.139	1346	1452	1426
P81133	1.310	1.211	1.187	1347	2005	1857
P82613	1.310	1.220	1.301	1348	2107	3116
P84012	1.310	1.196	1.197	1349	1846	1946
C83083	1.310	1.142	1.130	1350	1386	1346
F81644	1.310	1.100	1.089	1351	1102	1062
M83050	1.310	1.067	1.022	1352	898	714
B87025	1.310	1.325	1.342	1353	3347	3695
M83037	1.310	1.103	1.005	1354	1122	642
C82068	1.310	1.216	1.172	1355	2075	1735
G83052	1.309	1.115	1.082	1356	1191	1021
C84074	1.309	1.209	1.219	1357	1991	2171
P83603	1.309	1.311	1.274	1358	3183	2783
M83033	1.309	1.177	1.224	1359	1684	2241
F81700	1.309	1.151	1.163	1360	1458	1659
C82047	1.308	1.077	1.062	1361	974	895
B81616	1.308	1.219	1.343	1362	2116	3712
C86611	1.308	1.172	1.184	1363	1637	1846
C87002	1.308	1.209	1.187	1364	2002	1874
L84055	1.308	1.093	1.055	1365	1059	863
E85728	1.308	1.104	1.149	1366	1133	1524
C82053	1.308	1.256	1.213	1367	2506	2118
D82062	1.308	1.094	1.061	1368	1073	897
P84043	1.308	1.211	1.215	1369	2035	2141
F82045	1.307	1.029	1.046	1370	739	821
C83008	1.307	1.139	1.072	1371	1385	957
M83698	1.307	1.092	1.045	1372	1057	815
B86094	1.307	1.173	1.252	1373	1657	2549
L81089	1.307	1.186	1.162	1374	1780	1665
G82049	1.307	1.198	1.165	1375	1905	1689
D83078	1.307	1.103	1.087	1376	1135	1071
P81054	1.307	1.284	1.382	1377	2866	4270
G82211	1.307	1.292	1.218	1378	2976	2188
N85024	1.307	1.275	1.292	1379	2762	3047
N84037	1.306	1.212	1.215	1380	2074	2158
G82715	1.306	1.068	1.045	1381	925	822
K81608	1.306	1.016	1.010	1382	688	671
M84050	1.306	1.166	1.129	1383	1601	1371
G82648	1.306	0.982	0.972	1384	579	537
M83111	1.306	1.422	1.453	1385	4751	5154
D82085	1.306	1.105	1.091	1386	1155	1100
M84065	1.306	1.123	1.069	1387	1279	953
M84008	1.306	1.069	1.008	1388	939	669
F82022	1.305	1.084	1.046	1389	1019	832
L84042	1.305	1.164	1.157	1390	1589	1631
C84712	1.305	1.050	1.128	1391	843	1374
P85007	1.305	1.155	1.244	1392	1516	2498
E84009	1.305	1.117	1.035	1393	1236	781

M81070	1.305	1.120	1.052	1394	1265	866
L82047	1.304	1.245	1.200	1395	2423	2027
F81164	1.304	1.140	1.136	1396	1418	1453
P92007	1.304	1.234	1.206	1397	2297	2095
G83007	1.304	1.079	1.115	1398	1010	1272
N81074	1.304	1.216	1.170	1399	2125	1758
P85025	1.304	1.032	1.033	1400	762	777
M88623	1.303	1.126	1.202	1401	1315	2052
N81057	1.303	1.169	1.146	1402	1652	1536
M84620	1.303	1.063	1.058	1403	910	905
E84713	1.303	1.042	0.980	1404	820	567
C87609	1.303	1.251	1.324	1405	2508	3531
B85611	1.303	1.222	1.321	1406	2195	3498
A86005	1.303	1.247	1.269	1407	2464	2799
G82091	1.303	1.092	1.093	1408	1089	1137
E85735	1.303	1.087	1.098	1409	1052	1162
K83004	1.303	1.122	1.058	1410	1295	907
P81607	1.302	1.141	1.205	1411	1434	2100
P89023	1.302	1.177	1.228	1412	1736	2344
G82006	1.302	1.060	1.042	1413	902	825
E82004	1.302	1.040	1.000	1414	816	651
C82073	1.302	1.137	1.113	1415	1414	1266
P86605	1.302	1.137	1.212	1416	1409	2179
G82667	1.302	1.026	1.103	1417	748	1208
M81083	1.302	1.084	1.010	1418	1037	694
M88618	1.302	1.064	1.132	1419	931	1440
C87031	1.302	1.379	1.367	1420	4216	4141
F86066	1.302	1.041	1.012	1421	821	707
L83113	1.301	1.235	1.230	1422	2356	2376
Y02653	1.301	1.253	1.242	1423	2546	2516
L81069	1.301	1.111	1.061	1424	1213	936
D82024	1.301	1.093	1.078	1425	1114	1047
A82004	1.301	1.192	1.122	1426	1902	1353
M86028	1.301	1.206	1.219	1427	2061	2270
P86013	1.301	1.130	1.169	1428	1361	1781
F81081	1.301	1.157	1.140	1429	1578	1511
M81017	1.300	1.097	1.075	1430	1146	1037
P87032	1.300	1.217	1.249	1431	2172	2622
N82109	1.299	1.248	1.293	1432	2517	3159
A83636	1.299	1.329	1.304	1433	3531	3304
L85048	1.299	1.126	1.106	1434	1352	1238
M91628	1.299	1.251	1.318	1435	2552	3511
N81117	1.299	1.284	1.237	1436	2966	2491
H83034	1.299	1.016	1.018	1437	723	739
Y03656	1.298	1.207	1.185	1438	2095	1940
L82617	1.298	1.246	1.254	1439	2511	2676
A81006	1.298	1.186	1.144	1440	1868	1564
C83037	1.298	1.131	1.112	1441	1394	1293
J82090	1.298	1.179	1.211	1442	1795	2207
D82044	1.298	1.235	1.188	1443	2388	1975

A89604	1.298	1.514	1.593	1444	5809	6458
P81659	1.298	1.090	1.118	1445	1116	1348
A88020	1.297	1.282	1.349	1446	2973	3955
J81047	1.297	1.064	1.063	1447	963	962
E86010	1.297	1.133	1.095	1448	1421	1178
L81119	1.297	1.367	1.287	1449	4120	3108
M85670	1.297	1.084	1.125	1450	1076	1412
J82066	1.297	1.085	1.040	1451	1084	846
K82036	1.297	1.094	1.037	1452	1143	830
L83103	1.297	1.206	1.167	1453	2099	1793
H85095	1.297	1.011	0.965	1454	710	541
B81004	1.297	1.219	1.150	1455	2224	1652
M81011	1.297	1.097	1.050	1456	1163	899
F82648	1.296	1.166	1.146	1457	1686	1608
E86019	1.296	1.129	1.085	1458	1393	1123
A82062	1.296	1.227	1.224	1459	2310	2363
P82018	1.296	1.213	1.233	1460	2169	2472
C88095	1.296	1.176	1.164	1461	1785	1776
G82744	1.296	1.078	1.158	1462	1035	1729
A89029	1.296	1.163	1.212	1463	1670	2251
P84032	1.296	1.211	1.247	1464	2156	2638
G82684	1.295	1.077	1.141	1465	1033	1568
C83617	1.295	1.112	1.082	1466	1275	1114
C84078	1.295	1.121	1.124	1467	1334	1424
P81735	1.295	1.158	1.228	1468	1631	2420
E85064	1.295	1.171	1.151	1469	1753	1672
H83023	1.295	1.033	1.021	1470	812	766
K82025	1.295	1.043	1.007	1471	855	712
G81057	1.295	1.195	1.113	1472	1987	1331
M85127	1.295	1.169	1.245	1473	1739	2629
G85723	1.295	1.019	1.067	1474	749	1015
M83638	1.295	1.066	1.061	1475	985	978
C84002	1.294	1.248	1.187	1476	2575	1996
M86008	1.294	1.218	1.202	1477	2238	2152
H81068	1.294	1.056	0.992	1478	923	652
K83028	1.294	1.194	1.167	1479	1989	1829
P81737	1.294	1.181	1.175	1480	1861	1892
C83071	1.294	1.222	1.252	1481	2277	2714
P81035	1.294	1.359	1.433	1482	4049	5074
F81090	1.294	1.072	1.004	1483	1021	705
F81219	1.294	1.104	1.130	1484	1216	1487
M83030	1.293	1.118	1.090	1485	1327	1174
P81099	1.293	1.154	1.129	1486	1610	1482
H85637	1.293	0.936	0.952	1487	478	518
C83038	1.293	1.096	1.064	1488	1175	1007
M84062	1.293	1.109	1.104	1489	1271	1269
P81121	1.293	1.290	1.279	1490	3119	3056
C85003	1.293	1.253	1.224	1491	2669	2402
A89013	1.293	1.368	1.354	1492	4194	4078
F81065	1.293	1.030	1.018	1493	809	760

P84040	1.293	1.089	1.168	1494	1134	1844
F81645	1.293	1.178	1.182	1495	1848	1967
E82083	1.292	1.082	1.037	1496	1094	849
C83626	1.292	1.077	1.145	1497	1058	1638
M85013	1.292	1.064	1.070	1498	994	1056
D83080	1.292	1.084	1.068	1499	1110	1043
C84001	1.292	1.259	1.204	1500	2758	2197
C81649	1.292	1.120	1.069	1501	1356	1052
E81065	1.292	1.036	1.047	1502	846	911
G83061	1.291	1.052	1.023	1503	912	795
M81038	1.291	1.135	1.050	1504	1478	931
C83058	1.291	1.148	1.099	1505	1585	1248
F81193	1.291	1.183	1.121	1506	1907	1434
C83652	1.291	1.301	1.427	1507	3279	5039
F81198	1.291	1.091	1.170	1508	1158	1875
E85643	1.291	1.068	1.113	1509	1015	1373
B85610	1.291	1.076	1.081	1510	1062	1135
C87024	1.291	1.096	1.172	1511	1189	1897
F82030	1.290	1.097	1.048	1512	1194	924
D82051	1.290	1.251	1.205	1513	2682	2238
A82019	1.290	1.110	1.098	1514	1300	1252
N81017	1.290	1.076	1.048	1515	1070	921
G85125	1.290	1.106	1.070	1516	1276	1068
K83043	1.290	1.099	1.097	1517	1210	1239
P86004	1.290	1.138	1.130	1518	1500	1518
P84016	1.290	1.187	1.228	1519	1958	2493
L84071	1.289	1.175	1.135	1520	1843	1563
A89027	1.289	1.222	1.282	1521	2344	3149
C84057	1.289	1.157	1.178	1522	1681	1960
G81041	1.289	1.220	1.133	1523	2316	1549
F85686	1.289	1.100	1.158	1524	1228	1785
J82002	1.289	1.261	1.242	1525	2808	2652
Y02867	1.289	1.166	1.281	1526	1763	3130
D81645	1.289	1.191	1.260	1527	2018	2869
N81068	1.289	1.170	1.110	1528	1804	1357
M92010	1.289	1.047	1.034	1529	903	854
A83632	1.288	1.321	1.293	1530	3592	3309
C84709	1.288	1.119	1.108	1531	1375	1338
G82038	1.288	1.278	1.231	1532	3031	2536
F81120	1.288	1.078	1.054	1533	1097	969
A83051	1.288	1.337	1.284	1534	3817	3192
A81036	1.287	1.237	1.230	1535	2526	2533
B82073	1.287	1.142	1.083	1536	1564	1168
C84695	1.287	1.243	1.360	1537	2611	4244
A82009	1.287	1.244	1.229	1538	2628	2525
J83014	1.287	1.095	1.062	1539	1202	1038
C87608	1.287	1.116	1.148	1540	1363	1722
M82059	1.287	1.103	1.111	1541	1268	1382
F85666	1.287	1.077	1.124	1542	1095	1494
M83629	1.287	1.281	1.396	1543	3089	4689

C88039	1.287	1.384	1.343	1544	4488	4016
E85707	1.287	1.056	1.085	1545	978	1184
M84029	1.286	1.047	1.017	1546	914	785
A83007	1.286	1.340	1.281	1547	3874	3176
J83642	1.286	1.193	1.150	1548	2089	1749
M83020	1.286	1.003	0.968	1549	722	585
E82657	1.285	1.038	1.016	1550	879	790
E85633	1.285	0.967	0.999	1551	604	719
A81039	1.285	1.073	1.079	1552	1088	1158
N81070	1.285	1.145	1.102	1553	1604	1322
P91625	1.285	1.372	1.396	1554	4366	4707
M84066	1.285	1.039	1.010	1555	888	761
M91036	1.285	1.205	1.262	1556	2203	2937
B82060	1.284	1.149	1.115	1557	1643	1442
B86015	1.284	1.158	1.156	1558	1730	1814
D82029	1.284	1.092	1.069	1559	1205	1098
B87018	1.284	1.152	1.152	1560	1677	1779
Y01139	1.284	1.137	1.125	1561	1551	1528
F83027	1.283	1.098	1.176	1562	1254	1991
F83007	1.283	1.147	1.156	1563	1633	1821
K83058	1.283	1.116	1.201	1564	1392	2260
K84024	1.283	1.084	1.040	1565	1164	915
P82006	1.283	1.142	1.138	1566	1591	1657
D81064	1.283	1.341	1.368	1567	3947	4408
E84653	1.283	0.963	0.988	1568	593	676
N82050	1.283	1.254	1.332	1569	2798	3922
Y02710	1.282	1.095	1.155	1570	1246	1828
P87639	1.282	1.324	1.435	1571	3721	5214
D83027	1.282	1.092	1.099	1572	1219	1320
B83028	1.282	1.183	1.196	1573	1995	2224
H85076	1.282	1.183	1.156	1574	1999	1835
A82623	1.282	1.208	1.181	1575	2265	2065
H81620	1.281	1.025	0.988	1576	839	685
B83602	1.281	1.041	1.013	1577	908	793
N82041	1.281	1.243	1.284	1578	2689	3281
B85030	1.281	1.148	1.145	1579	1667	1742
M85059	1.281	1.317	1.346	1580	3617	4118
M81010	1.281	1.228	1.182	1581	2490	2083
N81614	1.281	1.109	1.053	1582	1357	1009
F86008	1.281	0.996	0.939	1583	713	514
A81618	1.281	1.199	1.192	1584	2189	2189
A89021	1.280	1.232	1.203	1585	2545	2308
N84005	1.280	1.131	1.109	1586	1524	1428
H82011	1.280	1.135	1.117	1587	1565	1491
Y00955	1.280	1.372	1.503	1588	4424	5906
M84618	1.280	1.102	1.144	1589	1313	1748
E82115	1.280	1.066	1.034	1590	1068	903
H81132	1.280	1.042	1.032	1591	930	890
M85146	1.280	1.041	1.057	1592	927	1050
K81019	1.279	1.085	1.051	1593	1186	1011

G82058	1.279	1.115	1.051	1594	1415	1008
J82127	1.279	1.176	1.173	1595	1951	2002
G82018	1.279	1.065	1.065	1596	1064	1112
A86037	1.279	1.405	1.393	1597	4870	4743
H82069	1.279	1.245	1.231	1598	2741	2647
N82049	1.279	1.311	1.357	1599	3586	4318
Y02342	1.279	0.918	0.899	1600	472	402
N85020	1.278	1.242	1.266	1601	2705	3078
A82020	1.278	1.163	1.156	1602	1833	1867
Y02344	1.278	1.217	1.330	1603	2408	3961
A83073	1.278	1.340	1.297	1604	4002	3507
J83035	1.278	1.151	1.140	1605	1727	1728
B85638	1.278	1.114	1.125	1606	1422	1581
K82026	1.278	1.212	1.162	1607	2365	1924
P91629	1.277	1.134	1.161	1608	1583	1919
M85015	1.277	1.038	1.087	1609	920	1264
P81152	1.277	1.082	1.073	1610	1183	1161
A81058	1.277	1.156	1.147	1611	1776	1796
N81052	1.277	1.120	1.080	1612	1460	1221
C82614	1.277	1.084	1.081	1613	1197	1224
N83013	1.277	1.096	1.080	1614	1292	1222
M92004	1.277	1.122	1.219	1615	1481	2534
E85697	1.277	1.059	1.008	1616	1042	786
N85019	1.277	1.253	1.285	1617	2851	3347
P81087	1.277	1.363	1.280	1618	4335	3297
G82753	1.276	1.033	1.033	1619	897	917
C87620	1.276	1.391	1.365	1620	4722	4448
B83617	1.276	1.107	1.155	1621	1374	1873
M91013	1.276	1.212	1.214	1622	2381	2478
M81094	1.276	1.110	1.068	1623	1403	1145
M83036	1.276	1.136	1.078	1624	1595	1215
A87007	1.276	1.182	1.159	1625	2078	1907
K82020	1.276	1.094	1.050	1626	1286	1032
P84645	1.276	1.213	1.250	1627	2394	2895
C84024	1.276	1.137	1.078	1628	1607	1216
P81143	1.276	1.135	1.090	1629	1590	1296
M91640	1.276	1.261	1.358	1630	2974	4365
G82110	1.275	1.031	0.989	1631	891	720
L84606	1.275	1.097	1.061	1632	1312	1104
M91025	1.275	1.146	1.216	1633	1701	2519
H82020	1.275	1.146	1.091	1634	1706	1315
B85036	1.275	1.238	1.221	1635	2702	2565
Y02581	1.275	1.129	1.141	1636	1558	1756
C88090	1.275	1.367	1.389	1637	4426	4751
N81035	1.275	1.180	1.160	1638	2060	1930
K83021	1.275	1.123	1.096	1639	1502	1354
N85044	1.275	1.184	1.201	1640	2102	2347
A88011	1.275	1.241	1.302	1641	2743	3615
C83635	1.275	1.239	1.221	1642	2731	2590
C82116	1.274	1.162	1.183	1643	1869	2161

J81035	1.274	1.197	1.165	1644	2225	1978
B86048	1.274	1.122	1.114	1645	1499	1516
H85116	1.274	1.024	0.991	1646	866	729
N81053	1.274	1.082	1.029	1647	1204	908
P82030	1.274	1.105	1.161	1648	1384	1948
A81019	1.274	1.231	1.256	1649	2624	3009
H85113	1.274	1.035	1.037	1650	918	954
A86024	1.274	1.121	1.126	1651	1497	1639
C84045	1.273	1.108	1.075	1652	1405	1209
B84021	1.273	1.147	1.149	1653	1729	1858
K83619	1.273	1.001	1.042	1654	763	992
Y02469	1.273	0.986	1.039	1655	707	980
B85010	1.273	1.162	1.148	1656	1890	1843
D81048	1.272	1.145	1.127	1657	1722	1662
N82051	1.272	1.191	1.283	1658	2188	3397
M81019	1.272	1.192	1.173	1659	2198	2078
G82138	1.272	1.163	1.147	1660	1904	1841
C85014	1.272	1.174	1.139	1661	2022	1773
M86041	1.271	1.180	1.254	1662	2094	3008
B81037	1.271	1.209	1.177	1663	2403	2131
N84613	1.271	1.164	1.130	1664	1917	1699
E82107	1.271	1.037	0.996	1665	959	762
Y00758	1.271	1.125	1.192	1666	1556	2297
M83011	1.271	1.152	1.082	1667	1800	1268
B86016	1.271	1.237	1.246	1668	2740	2908
N81618	1.271	1.251	1.342	1669	2901	4211
N84036	1.271	1.024	1.011	1670	885	829
E81041	1.271	1.067	1.050	1671	1140	1065
C82680	1.270	1.146	1.195	1672	1746	2336
A87005	1.270	1.125	1.114	1673	1566	1547
B81075	1.270	0.954	1.049	1674	612	1061
B85038	1.270	1.176	1.140	1675	2072	1788
C84613	1.270	1.001	0.985	1676	775	723
M83044	1.270	1.168	1.095	1677	1967	1387
P81085	1.270	1.262	1.242	1678	3059	2875
A81011	1.270	1.326	1.330	1679	3913	4074
P86608	1.270	1.088	1.171	1680	1284	2089
N82115	1.270	1.427	1.448	1681	5244	5471
H85087	1.269	1.077	1.069	1682	1203	1197
C84672	1.269	1.050	1.085	1683	1031	1310
C83052	1.269	1.084	1.107	1684	1255	1495
F81151	1.269	1.234	1.240	1685	2732	2860
M86002	1.269	1.238	1.242	1686	2766	2879
B82049	1.269	1.132	1.076	1687	1630	1241
B85044	1.269	1.122	1.176	1688	1555	2145
C85026	1.269	1.203	1.223	1689	2370	2669
Y02644	1.269	1.220	1.290	1690	2555	3541
P82629	1.268	1.180	1.217	1691	2122	2618
Y03079	1.268	1.163	1.185	1692	1930	2259
C83082	1.268	1.148	1.165	1693	1790	2042

F81128	1.268	1.173	1.144	1694	2064	1860
C87020	1.268	1.274	1.288	1695	3229	3520
D83608	1.268	1.205	1.162	1696	2397	2005
M81072	1.268	1.081	1.062	1697	1245	1159
E81076	1.268	1.079	1.075	1698	1229	1249
H81118	1.267	1.029	1.002	1699	924	806
N85012	1.267	1.147	1.094	1700	1789	1406
G82719	1.267	1.009	1.013	1701	830	858
H81076	1.267	1.063	1.045	1702	1132	1057
F84010	1.267	1.019	0.958	1703	881	623
E84044	1.267	1.046	0.989	1704	1026	749
P84640	1.267	1.053	1.140	1705	1072	1830
P81040	1.267	1.123	1.098	1706	1577	1445
F84083	1.267	1.102	1.067	1707	1412	1199
D81021	1.266	1.133	1.090	1708	1658	1380
C85020	1.266	1.104	1.085	1709	1428	1332
P81057	1.266	1.115	1.071	1710	1509	1229
A89036	1.266	1.234	1.299	1711	2760	3687
N85013	1.266	1.107	1.078	1712	1445	1282
G81026	1.266	1.161	1.172	1713	1937	2132
P81005	1.266	1.262	1.313	1714	3121	3902
N81107	1.265	1.245	1.339	1715	2903	4248
C84052	1.265	1.051	1.026	1716	1074	940
P81070	1.265	1.240	1.314	1717	2834	3924
K84613	1.265	1.013	1.011	1718	856	856
B85011	1.265	1.096	1.095	1719	1376	1437
P81137	1.265	1.184	1.213	1720	2194	2599
E82061	1.265	1.109	1.080	1721	1476	1309
N85023	1.265	1.226	1.237	1722	2675	2876
D83082	1.265	1.093	1.066	1723	1359	1212
B86057	1.265	1.127	1.110	1724	1623	1566
H83012	1.264	1.040	1.035	1725	1011	999
N81059	1.264	1.095	1.063	1726	1380	1185
C83011	1.264	1.125	1.086	1727	1608	1360
J83034	1.264	1.116	1.074	1728	1539	1263
P88018	1.264	1.145	1.113	1729	1797	1601
E81021	1.264	1.173	1.135	1730	2104	1803
M86026	1.264	1.531	1.535	1731	6311	6288
B81119	1.264	1.198	1.329	1732	2364	4147
C82028	1.264	1.182	1.144	1733	2193	1888
L81032	1.263	1.211	1.197	1734	2500	2426
P83021	1.263	1.137	1.193	1735	1732	2393
N81025	1.263	1.148	1.099	1736	1841	1478
N83001	1.263	1.247	1.241	1737	2958	2944
P85018	1.263	1.228	1.303	1738	2729	3798
E86005	1.263	1.094	1.056	1739	1377	1152
B83628	1.263	1.098	1.181	1740	1410	2266
M88014	1.263	1.194	1.240	1741	2325	2932
G81019	1.263	1.047	1.005	1742	1049	841
L81067	1.263	1.196	1.168	1743	2358	2128

E84068	1.263	1.070	1.004	1744	1200	840
M84003	1.263	1.114	1.074	1745	1533	1277
F86691	1.262	1.046	1.030	1746	1047	984
N81065	1.262	1.176	1.194	1747	2141	2417
F85652	1.262	1.063	1.071	1748	1161	1257
C82082	1.262	1.143	1.110	1749	1801	1587
G83010	1.262	1.214	1.141	1750	2553	1879
N81015	1.262	1.112	1.061	1751	1521	1188
L84073	1.262	1.283	1.252	1752	3432	3113
M83042	1.262	1.128	1.070	1753	1668	1253
A82647	1.261	1.199	1.134	1754	2402	1827
D82035	1.261	1.298	1.268	1755	3635	3331
F85045	1.261	0.941	0.976	1756	594	722
K83005	1.261	1.160	1.169	1757	1974	2157
Y00140	1.261	1.327	1.382	1758	4058	4839
M84005	1.261	1.024	1.044	1759	935	1091
P81067	1.261	1.141	1.105	1760	1796	1551
B83055	1.260	1.157	1.133	1761	1956	1826
A83008	1.260	1.408	1.342	1762	5135	4362
P92041	1.260	1.090	1.082	1763	1373	1368
B86003	1.260	1.078	1.060	1764	1287	1198
A81016	1.260	1.155	1.193	1765	1939	2427
J82165	1.260	1.106	1.111	1766	1485	1624
A81047	1.260	1.195	1.142	1767	2375	1905
G82075	1.260	1.119	1.104	1768	1592	1550
C84051	1.259	1.087	1.104	1769	1360	1555
P81664	1.259	1.216	1.186	1770	2629	2356
H81644	1.259	1.016	0.964	1771	899	679
C84043	1.259	1.190	1.159	1772	2329	2074
C82081	1.259	1.129	1.193	1773	1693	2434
P86010	1.259	1.213	1.273	1774	2587	3449
N84615	1.259	1.250	1.309	1775	3052	3942
E85623	1.259	0.995	1.024	1776	808	963
H81618	1.258	1.093	1.031	1777	1411	1016
N85053	1.258	1.163	1.220	1778	2047	2752
L85030	1.258	1.176	1.140	1779	2180	1901
A82058	1.258	1.231	1.292	1780	2811	3706
P86018	1.258	1.214	1.200	1781	2610	2523
Y02477	1.258	1.049	1.121	1782	1105	1738
H81133	1.258	1.036	0.989	1783	1024	792
M89001	1.258	1.188	1.196	1784	2307	2485
A82005	1.258	1.065	1.025	1785	1199	983
C81014	1.258	1.223	1.193	1786	2737	2454
E81057	1.257	1.128	1.105	1787	1698	1588
N84035	1.257	1.205	1.182	1788	2501	2341
M81045	1.257	1.108	1.062	1789	1532	1227
M84043	1.257	1.070	1.063	1790	1239	1233
F81606	1.257	1.308	1.300	1791	3851	3845
L83642	1.256	1.143	1.223	1792	1857	2806
J82132	1.256	1.159	1.114	1793	2029	1697

P88010	1.256	1.750	1.728	1794	7228	7153
E82051	1.256	1.071	1.031	1795	1263	1042
G82085	1.256	1.066	1.014	1796	1217	926
F85004	1.255	1.141	1.097	1797	1853	1531
B81005	1.255	1.188	1.178	1798	2351	2312
G85106	1.255	1.030	1.059	1799	1007	1228
D82028	1.255	1.192	1.113	1800	2398	1703
P84056	1.254	1.180	1.275	1801	2252	3527
B81635	1.254	1.218	1.357	1802	2710	4619
E81006	1.254	1.054	1.020	1803	1156	972
B83042	1.254	1.286	1.277	1804	3565	3553
F81036	1.254	1.072	1.053	1805	1282	1186
P81184	1.254	1.186	1.155	1806	2335	2088
P81089	1.254	1.312	1.255	1807	3953	3269
J82004	1.254	1.151	1.185	1808	1959	2412
K82012	1.254	1.094	1.055	1809	1444	1211
P81046	1.254	1.108	1.108	1810	1563	1660
E81036	1.254	1.153	1.121	1811	1976	1768
K83024	1.253	1.177	1.149	1812	2233	2030
M88643	1.253	1.065	1.128	1813	1232	1837
C83650	1.253	1.233	1.213	1814	2902	2735
L83071	1.253	1.165	1.210	1815	2124	2692
D81008	1.253	1.197	1.202	1816	2462	2621
N81072	1.253	1.353	1.297	1817	4527	3869
D81084	1.253	1.095	1.055	1818	1459	1213
L82043	1.253	1.063	1.046	1819	1223	1154
P81159	1.253	1.412	1.478	1820	5266	5934
M87021	1.253	1.093	1.091	1821	1450	1508
D81046	1.252	1.144	1.153	1822	1909	2079
N81628	1.252	1.122	1.081	1823	1694	1431
P81039	1.252	1.157	1.152	1824	2057	2069
M83001	1.252	1.183	1.155	1825	2323	2102
Y00260	1.252	1.082	1.068	1826	1371	1318
K83036	1.252	1.173	1.133	1827	2218	1891
G82888	1.252	0.959	0.931	1828	681	574
Y02842	1.252	0.930	0.984	1829	589	800
L85020	1.252	1.140	1.106	1830	1878	1658
M91008	1.252	1.138	1.114	1831	1851	1734
K83069	1.252	1.339	1.346	1832	4369	4521
B81002	1.251	1.181	1.298	1833	2293	3896
A81610	1.251	1.122	1.112	1834	1695	1715
P81186	1.251	1.300	1.272	1835	3805	3524
A87016	1.251	1.244	1.232	1836	3067	2998
F85044	1.251	1.033	1.072	1837	1036	1352
C83078	1.251	1.098	1.130	1838	1490	1876
P87657	1.251	1.269	1.257	1839	3387	3320
B85051	1.251	1.175	1.160	1840	2239	2165
P82624	1.251	1.103	1.163	1841	1534	2200
M83108	1.251	1.136	1.044	1842	1831	1151
D81004	1.251	1.168	1.149	1843	2171	2051

H85065	1.251	1.012	0.970	1844	916	740
H85653	1.251	1.112	1.081	1845	1612	1443
M83023	1.251	1.181	1.145	1846	2300	2011
J82178	1.250	1.053	1.016	1847	1174	965
G82215	1.250	1.072	1.055	1848	1314	1226
F81697	1.250	1.125	1.140	1849	1741	1974
H81055	1.250	1.098	1.051	1850	1498	1207
B81031	1.250	1.146	1.152	1851	1948	2098
P92008	1.250	1.276	1.250	1852	3490	3251
L83015	1.250	1.206	1.239	1853	2622	3103
E81004	1.249	1.092	1.065	1854	1464	1312
C83035	1.249	1.119	1.077	1855	1690	1415
N81094	1.249	1.182	1.119	1856	2339	1790
F81122	1.249	1.104	1.063	1857	1561	1294
P89013	1.249	1.379	1.370	1858	4930	4836
J82115	1.249	1.140	1.130	1859	1898	1896
A88613	1.249	1.337	1.358	1860	4374	4697
F81035	1.249	1.115	1.094	1861	1653	1554
J82122	1.249	1.147	1.141	1862	1966	1990
M83113	1.249	1.235	1.232	1863	2982	3017
C83044	1.249	1.213	1.170	1864	2716	2294
C85623	1.249	1.008	1.065	1865	906	1319
D82041	1.248	1.148	1.096	1866	1984	1582
P84631	1.248	1.147	1.237	1867	1968	3091
G83006	1.248	1.123	1.069	1868	1745	1347
G81061	1.248	1.191	1.130	1869	2450	1899
C84667	1.248	1.149	1.149	1870	2003	2080
A82053	1.248	1.121	1.061	1871	1724	1292
N81067	1.248	1.084	1.034	1872	1419	1113
C86030	1.247	1.281	1.367	1873	3599	4816
P86003	1.247	1.071	1.075	1874	1323	1419
C88010	1.247	1.134	1.114	1875	1858	1770
L81094	1.247	1.399	1.373	1876	5186	4897
B82081	1.247	1.051	1.032	1877	1179	1097
E82043	1.247	1.044	1.026	1878	1144	1066
M84617	1.247	1.073	1.061	1879	1341	1299
A82063	1.247	1.088	1.076	1880	1453	1433
C86020	1.247	1.181	1.189	1881	2363	2538
P92011	1.247	1.172	1.150	1882	2257	2105
M83064	1.247	1.100	1.036	1883	1553	1133
C83057	1.246	1.192	1.124	1884	2480	1865
A83033	1.246	1.209	1.159	1885	2694	2214
D81032	1.246	1.109	1.068	1886	1627	1364
C81082	1.246	1.110	1.110	1887	1639	1744
M85179	1.246	1.227	1.225	1888	2916	2956
M84024	1.246	1.095	1.065	1889	1508	1335
A81612	1.246	1.170	1.221	1890	2243	2898
J83008	1.246	1.117	1.085	1891	1700	1509
B87008	1.246	1.253	1.248	1892	3233	3289
Y00902	1.246	0.987	1.034	1893	829	1125

E81059	1.246	1.155	1.146	1894	2106	2076
P87022	1.246	1.277	1.337	1895	3561	4491
C88656	1.246	1.133	1.178	1896	1866	2423
J82060	1.245	1.168	1.194	1897	2228	2611
A83003	1.245	1.217	1.173	1898	2795	2368
E81018	1.245	1.036	1.014	1899	1101	989
J81050	1.245	1.151	1.115	1900	2069	1786
H85005	1.245	1.040	0.997	1901	1125	884
P84052	1.245	1.248	1.316	1902	3199	4212
D82081	1.245	1.228	1.219	1903	2939	2886
K83077	1.245	1.152	1.203	1904	2091	2715
Y01695	1.245	1.130	1.216	1905	1847	2862
D81025	1.245	1.143	1.070	1906	1963	1397
K83003	1.245	1.099	1.121	1907	1557	1856
M82021	1.245	1.229	1.200	1908	2959	2675
G81030	1.245	1.038	0.999	1909	1117	901
P81170	1.244	1.164	1.210	1910	2200	2798
P81103	1.244	1.241	1.202	1911	3125	2699
G82126	1.244	1.191	1.175	1912	2491	2397
A82055	1.244	1.227	1.180	1913	2940	2458
N81005	1.244	1.116	1.066	1914	1713	1363
F81711	1.244	1.421	1.511	1915	5472	6258
H81024	1.244	1.017	0.996	1916	997	891
Y02815	1.244	1.280	1.382	1917	3636	5062
H82006	1.244	1.126	1.097	1918	1808	1645
M92040	1.243	1.124	1.214	1919	1793	2849
K81024	1.243	1.058	1.017	1920	1256	1026
M89027	1.243	1.252	1.189	1921	3260	2564
D82098	1.243	1.351	1.359	1922	4638	4773
J83057	1.243	1.109	1.086	1923	1659	1544
C88060	1.243	1.120	1.201	1924	1760	2712
B86643	1.243	1.216	1.236	1925	2814	3147
D81024	1.243	1.134	1.134	1926	1900	1983
G85120	1.243	1.102	1.101	1927	1594	1694
J81625	1.243	1.102	1.089	1928	1596	1571
J83047	1.243	1.075	1.066	1929	1391	1376
P81692	1.242	1.235	1.284	1930	3066	3818
P91604	1.242	1.049	1.035	1931	1193	1153
N81109	1.242	1.112	1.102	1932	1688	1711
B83035	1.242	1.209	1.230	1933	2750	3073
D82026	1.242	1.164	1.173	1934	2222	2408
E82082	1.242	1.184	1.150	1935	2444	2160
N82113	1.242	1.148	1.235	1936	2079	3151
N81091	1.242	1.047	1.002	1937	1185	937
D81081	1.242	1.198	1.179	1938	2614	2471
L83112	1.242	1.220	1.326	1939	2882	4392
J83005	1.242	1.103	1.063	1940	1619	1362
G83047	1.241	1.048	1.003	1941	1196	943
C88011	1.241	1.326	1.344	1942	4326	4616
M87623	1.241	1.092	1.160	1943	1531	2278

J81073	1.241	1.195	1.167	1944	2591	2348
N83053	1.241	1.110	1.166	1945	1687	2342
J82198	1.241	1.108	1.069	1946	1673	1420
P87015	1.241	1.424	1.511	1947	5533	6277
M92002	1.241	1.251	1.227	1948	3274	3059
B85028	1.241	1.154	1.112	1949	2145	1815
G82604	1.240	1.116	1.100	1950	1751	1712
M81056	1.240	1.146	1.098	1951	2071	1692
G81089	1.240	1.133	1.247	1952	1918	3328
F81072	1.240	1.086	1.029	1953	1483	1128
B86678	1.240	1.155	1.187	1954	2154	2592
E86640	1.240	1.053	1.023	1955	1244	1086
D83026	1.240	1.072	1.071	1956	1387	1446
A89031	1.240	1.241	1.256	1957	3176	3467
B83026	1.240	1.075	1.089	1958	1417	1605
H81638	1.239	1.065	1.027	1959	1330	1119
G81027	1.239	1.130	1.119	1960	1897	1882
C84656	1.239	1.255	1.235	1961	3349	3187
K82014	1.239	1.066	1.009	1962	1351	996
C88027	1.239	1.154	1.177	1963	2149	2484
A88608	1.239	1.282	1.255	1964	3740	3460
F81127	1.239	1.037	1.010	1965	1149	1004
N81069	1.239	1.038	1.003	1966	1150	955
C82072	1.239	1.114	1.061	1967	1747	1361
K84040	1.239	1.024	0.988	1968	1055	879
M86019	1.239	1.122	1.080	1969	1820	1522
J82152	1.239	1.105	1.107	1970	1660	1775
B82037	1.239	1.133	1.126	1971	1928	1953
P81029	1.238	1.169	1.173	1972	2304	2447
D83002	1.238	1.196	1.149	1973	2631	2181
B84012	1.238	1.149	1.146	1974	2119	2151
L82057	1.238	1.098	1.064	1975	1603	1396
M86016	1.238	1.158	1.205	1976	2201	2809
B81628	1.238	1.067	1.099	1977	1364	1725
C83080	1.238	1.081	1.029	1978	1474	1139
A87022	1.238	1.143	1.143	1979	2067	2125
P81086	1.237	1.218	1.166	1980	2920	2374
G82019	1.237	1.089	1.060	1981	1540	1375
M86033	1.237	1.310	1.358	1982	4157	4829
M83110	1.237	1.222	1.232	1983	2971	3171
M81025	1.237	1.175	1.144	1984	2396	2144
B81693	1.237	1.116	1.212	1985	1781	2900
K81069	1.237	1.042	1.001	1986	1187	958
E85083	1.236	1.187	1.222	1987	2541	3057
M84015	1.236	1.107	1.073	1988	1705	1484
B81634	1.236	1.190	1.323	1989	2588	4429
M91660	1.236	1.196	1.305	1990	2661	4188
P81088	1.236	1.254	1.239	1991	3396	3291
M81075	1.236	1.084	1.030	1992	1506	1156
P81014	1.236	1.119	1.107	1993	1825	1813

M83096	1.236	1.141	1.063	1994	2056	1414
A87003	1.236	1.260	1.249	1995	3460	3434
C81662	1.235	1.136	1.137	1996	1993	2082
E82002	1.235	1.093	1.046	1997	1586	1270
N83050	1.235	1.237	1.329	1998	3188	4520
P81059	1.235	1.165	1.131	1999	2301	2029
D81607	1.235	1.091	1.073	2000	1573	1502
E82050	1.235	1.121	1.088	2001	1856	1641
C82096	1.235	1.160	1.120	2002	2256	1926
F81025	1.235	1.151	1.136	2003	2165	2091
F86010	1.235	1.014	0.945	2004	1027	706
A82065	1.234	1.321	1.243	2005	4353	3350
B85033	1.234	1.072	1.072	2006	1429	1503
C86005	1.234	1.368	1.363	2007	4961	4936
B81013	1.234	1.077	1.032	2008	1469	1182
P82023	1.234	1.102	1.098	2009	1680	1741
E85018	1.234	0.983	0.965	2010	860	787
E81035	1.234	1.165	1.132	2011	2321	2050
L83657	1.234	1.101	1.068	2012	1666	1467
F81725	1.234	1.353	1.259	2013	4781	3590
A83028	1.234	1.118	1.101	2014	1837	1765
C83072	1.234	1.077	1.087	2015	1475	1647
G82681	1.233	1.055	1.031	2016	1311	1180
P81149	1.233	1.193	1.171	2017	2665	2490
A81051	1.233	1.109	1.162	2018	1758	2378
L83147	1.233	1.098	1.157	2019	1645	2332
H82055	1.233	1.041	0.995	2020	1209	946
N81041	1.233	1.085	1.047	2021	1544	1301
G82048	1.233	1.131	1.099	2022	1971	1763
H81656	1.232	1.047	0.983	2023	1260	880
M88022	1.232	1.111	1.172	2024	1778	2503
N83020	1.232	1.270	1.256	2025	3657	3560
F81223	1.232	1.087	1.142	2026	1562	2170
M87009	1.232	1.125	1.093	2027	1914	1713
P88618	1.232	1.123	1.189	2028	1901	2700
N81060	1.232	1.158	1.102	2029	2260	1799
Y02886	1.232	1.139	1.206	2030	2088	2889
G82229	1.232	1.085	1.101	2031	1552	1795
C82016	1.231	1.055	1.009	2032	1322	1051
A89035	1.231	1.277	1.250	2033	3775	3505
A82034	1.231	1.163	1.110	2034	2332	1877
C81023	1.231	1.114	1.069	2035	1814	1501
L84044	1.231	1.229	1.194	2036	3139	2759
F85707	1.230	0.949	0.938	2037	732	689
J81002	1.230	1.226	1.166	2038	3101	2456
M81049	1.230	1.125	1.105	2039	1933	1842
H81062	1.230	1.043	1.026	2040	1237	1164
M83103	1.230	1.105	1.027	2041	1744	1172
M83117	1.230	1.216	1.190	2042	2985	2733
M84012	1.230	1.112	1.088	2043	1811	1702

P89011	1.230	1.273	1.296	2044	3745	4165
D81073	1.230	1.060	1.058	2045	1372	1421
C81052	1.229	1.143	1.122	2046	2140	1994
D82031	1.229	1.082	1.049	2047	1546	1343
F81720	1.229	1.116	1.101	2048	1860	1818
A83009	1.229	1.158	1.140	2049	2280	2178
K81065	1.229	0.989	1.006	2050	911	1045
H81123	1.229	1.043	1.010	2051	1252	1077
M83700	1.229	1.048	1.142	2052	1291	2215
K83032	1.229	1.096	1.087	2053	1664	1698
P81197	1.229	1.112	1.162	2054	1818	2429
B81602	1.229	1.217	1.291	2055	3008	4089
M87017	1.229	1.192	1.188	2056	2706	2736
P87624	1.228	1.097	1.126	2057	1682	2045
F82006	1.228	1.077	1.041	2058	1513	1284
D83049	1.228	1.123	1.114	2059	1927	1932
H83046	1.228	1.265	1.228	2060	3633	3239
J81013	1.228	1.151	1.139	2061	2227	2182
B81039	1.228	1.187	1.187	2062	2647	2727
G85003	1.228	0.997	1.041	2063	975	1290
K83055	1.228	1.083	1.135	2064	1568	2142
L81117	1.228	1.127	1.090	2065	1980	1727
H85056	1.228	0.944	0.955	2066	717	769
M85007	1.228	1.178	1.236	2067	2529	3358
K83051	1.228	1.145	1.127	2068	2177	2064
C83046	1.228	1.078	1.063	2069	1518	1473
B81026	1.227	1.202	1.184	2070	2838	2685
M85045	1.227	1.164	1.218	2071	2389	3121
M83079	1.227	1.191	1.099	2072	2714	1808
C88048	1.227	1.183	1.253	2073	2613	3594
G82185	1.227	1.068	1.057	2074	1442	1439
B81007	1.227	1.116	1.107	2075	1883	1885
B83013	1.227	1.168	1.197	2076	2432	2847
K82051	1.227	1.051	0.999	2077	1325	1012
B86049	1.227	1.089	1.125	2078	1624	2048
B83018	1.226	1.108	1.083	2079	1803	1677
A87004	1.226	1.206	1.184	2080	2898	2707
Y02464	1.226	1.185	1.300	2081	2637	4250
K82037	1.226	1.099	1.057	2082	1725	1444
B81010	1.226	1.075	1.052	2083	1511	1400
G81033	1.226	1.125	1.144	2084	1970	2269
M92027	1.226	1.175	1.211	2085	2516	3038
J81081	1.226	1.130	1.089	2086	2038	1740
B81697	1.226	1.198	1.313	2087	2799	4428
J81059	1.226	1.098	1.091	2088	1709	1753
A88003	1.226	1.341	1.281	2089	4732	4004
P89609	1.226	1.240	1.314	2090	3324	4445
L83081	1.226	1.088	1.078	2091	1628	1634
P82643	1.226	1.066	1.084	2092	1443	1693
E81010	1.225	1.051	1.072	2093	1336	1578

P87003	1.225	1.191	1.299	2094	2733	4251
H85105	1.225	1.068	1.045	2095	1463	1341
F86083	1.225	1.066	1.027	2096	1451	1219
A81067	1.224	1.279	1.255	2097	3888	3663
B84610	1.224	1.060	1.072	2098	1420	1585
B81631	1.224	1.273	1.355	2099	3820	4962
C82051	1.224	1.100	1.090	2100	1754	1759
M81015	1.224	1.085	1.036	2101	1611	1280
K82061	1.224	1.125	1.062	2102	2006	1500
A83045	1.224	1.209	1.174	2103	2972	2634
L84069	1.224	1.239	1.173	2104	3339	2619
J83036	1.224	1.111	1.113	2105	1865	1970
K83006	1.223	1.201	1.185	2106	2873	2753
N82084	1.223	1.233	1.234	2107	3269	3391
M91006	1.223	1.245	1.269	2108	3431	3880
N81089	1.223	1.181	1.134	2109	2635	2185
M83146	1.223	1.229	1.264	2110	3228	3814
J81070	1.223	1.136	1.133	2111	2143	2177
P84065	1.223	1.139	1.247	2112	2162	3572
C88032	1.223	1.049	1.074	2113	1347	1622
F81746	1.223	1.060	1.116	2114	1430	2001
N85046	1.223	1.165	1.207	2115	2447	3039
M83701	1.222	1.267	1.261	2116	3750	3773
G83034	1.222	1.084	1.090	2117	1609	1772
K82603	1.222	1.130	1.066	2118	2090	1541
F82039	1.222	1.049	1.110	2119	1348	1949
N83027	1.222	1.414	1.476	2120	5598	6153
Y00969	1.222	1.121	1.094	2121	1975	1812
F81068	1.222	1.119	1.099	2122	1953	1864
A89007	1.222	1.194	1.218	2123	2800	3197
K82029	1.222	1.038	0.992	2124	1269	998
Y00090	1.222	1.146	1.139	2125	2245	2257
H84015	1.221	1.071	1.028	2126	1515	1236
L85016	1.221	1.221	1.166	2127	3154	2550
C82041	1.221	1.067	1.039	2128	1484	1330
P89002	1.221	1.175	1.180	2129	2589	2713
F81045	1.221	1.044	1.042	2130	1316	1358
G81025	1.221	1.364	1.263	2131	5068	3827
M92022	1.221	1.200	1.210	2132	2891	3100
P84626	1.221	1.048	1.121	2133	1349	2073
C86034	1.221	1.327	1.385	2134	4606	5341
B81074	1.221	1.391	1.519	2135	5388	6477
P84071	1.221	0.982	1.038	2136	922	1323
E81052	1.221	1.162	1.147	2137	2446	2351
C82044	1.221	1.126	1.077	2138	2065	1681
F86664	1.220	0.984	1.012	2139	940	1142
N83636	1.220	1.378	1.470	2140	5250	6131
A89611	1.220	0.963	1.049	2141	838	1429
F81138	1.220	1.109	1.071	2142	1885	1627
K82017	1.220	1.068	1.035	2143	1505	1305

F84014	1.220	1.047	1.057	2144	1350	1490
C88053	1.220	1.017	1.032	2145	1142	1285
N85034	1.220	1.221	1.199	2146	3182	2976
E82046	1.219	1.095	1.041	2147	1750	1356
M82046	1.219	1.238	1.210	2148	3394	3117
M85078	1.219	1.120	1.154	2149	1998	2450
M82027	1.219	1.132	1.106	2150	2133	1943
G82122	1.219	1.013	0.978	2151	1124	933
J82039	1.219	1.142	1.097	2152	2232	1866
H81097	1.219	1.071	1.036	2153	1543	1326
D83059	1.219	1.060	1.042	2154	1455	1377
K81622	1.218	0.981	1.030	2155	934	1276
N83610	1.218	1.320	1.389	2156	4552	5390
P82660	1.218	1.160	1.233	2157	2438	3454
C84694	1.218	1.103	1.207	2158	1836	3092
C88638	1.218	1.170	1.234	2159	2559	3468
N81008	1.218	1.148	1.100	2160	2309	1904
L81049	1.218	1.109	1.088	2161	1903	1794
L83655	1.218	1.146	1.066	2162	2275	1595
D81031	1.218	1.097	1.055	2163	1779	1489
G83037	1.218	1.037	1.019	2164	1294	1203
P85002	1.218	1.268	1.354	2165	3839	5038
C85010	1.218	1.235	1.196	2166	3383	2953
P83605	1.217	1.128	1.147	2167	2113	2389
E81026	1.217	1.126	1.154	2168	2098	2470
C83065	1.217	1.121	1.069	2169	2033	1635
B81027	1.217	1.267	1.389	2170	3829	5406
H81031	1.217	1.051	1.037	2171	1399	1349
N84625	1.217	0.988	1.013	2172	983	1167
C82106	1.217	1.274	1.287	2173	3938	4202
E81056	1.217	1.151	1.125	2174	2360	2149
M91609	1.217	1.152	1.225	2175	2376	3341
G81052	1.217	1.059	0.993	2176	1456	1046
G82097	1.217	1.111	1.093	2177	1926	1855
A81014	1.217	1.367	1.321	2178	5147	4634
E82070	1.217	1.035	1.035	2179	1283	1333
G82052	1.216	1.280	1.267	2180	4027	3938
C85001	1.216	1.317	1.362	2181	4541	5145
Y03052	1.216	1.205	1.282	2182	3028	4154
K81073	1.216	1.050	1.028	2183	1402	1278
K81006	1.216	1.009	0.979	2184	1119	948
P92016	1.216	1.120	1.094	2185	2044	1870
B81603	1.216	1.164	1.283	2186	2507	4178
N81120	1.216	1.072	1.022	2187	1580	1237
D82013	1.215	1.061	1.029	2188	1482	1286
A86036	1.215	1.055	1.052	2189	1440	1486
B86041	1.215	1.059	1.106	2190	1477	1977
N81062	1.215	1.125	1.088	2191	2108	1824
C81096	1.215	1.267	1.192	2192	3863	2930
P81732	1.215	1.143	1.173	2193	2279	2716

C82626	1.215	1.159	1.209	2194	2466	3166
K83601	1.214	1.131	1.164	2195	2164	2625
G82165	1.214	1.093	1.103	2196	1782	1959
Y00198	1.214	1.144	1.139	2197	2317	2345
H85030	1.214	1.075	1.026	2198	1613	1281
B83629	1.214	1.147	1.200	2199	2355	3067
C81033	1.213	1.317	1.254	2200	4577	3805
F81147	1.213	1.174	1.189	2201	2680	2913
K83618	1.213	1.099	1.138	2202	1854	2331
L83130	1.213	1.206	1.178	2203	3075	2781
C84033	1.213	1.172	1.120	2204	2649	2139
F81069	1.213	1.091	1.085	2205	1774	1817
B85644	1.213	1.069	1.056	2206	1575	1545
C84628	1.213	0.999	1.084	2207	1078	1816
N81021	1.212	1.075	1.043	2208	1626	1441
A82023	1.212	1.374	1.366	2209	5303	5227
F85076	1.212	1.017	0.987	2210	1181	1040
J83046	1.212	1.127	1.100	2211	2150	1954
F82020	1.212	1.115	1.102	2212	2027	1972
P84651	1.212	1.209	1.326	2213	3130	4740
D82054	1.212	1.164	1.121	2214	2566	2168
J82063	1.211	1.055	1.021	2215	1471	1262
B81685	1.211	1.197	1.329	2216	2980	4789
N85051	1.211	1.221	1.219	2217	3266	3346
G83015	1.211	1.163	1.141	2218	2549	2392
L84020	1.211	1.096	1.074	2219	1835	1733
L83624	1.211	1.351	1.380	2220	5025	5378
A82006	1.211	1.161	1.088	2221	2535	1862
C84103	1.211	1.107	1.142	2222	1940	2410
C81070	1.211	1.076	1.068	2223	1647	1687
B83070	1.211	1.138	1.189	2224	2271	2950
J81044	1.211	1.138	1.119	2225	2267	2159
D82032	1.211	1.169	1.153	2226	2639	2539
P81154	1.210	1.211	1.139	2227	3170	2382
D82039	1.210	1.202	1.184	2228	3057	2888
P87654	1.210	1.226	1.316	2229	3358	4650
Y02893	1.210	1.158	1.202	2230	2502	3141
C84648	1.210	1.095	1.064	2231	1838	1653
F82014	1.210	1.184	1.158	2232	2830	2596
C83059	1.210	1.134	1.072	2233	2244	1731
N83604	1.210	1.364	1.437	2234	5207	5949
B86672	1.210	1.248	1.378	2235	3672	5373
E86028	1.209	0.999	0.966	2236	1096	919
B81062	1.209	1.140	1.104	2237	2319	2021
P82037	1.209	1.231	1.275	2238	3438	4151
K81633	1.209	1.050	1.047	2239	1449	1497
M88009	1.209	1.162	1.182	2240	2582	2885
L83076	1.209	1.141	1.240	2241	2342	3670
A86028	1.209	1.111	1.199	2242	2014	3111
A83042	1.209	1.232	1.254	2243	3451	3873

N81016	1.209	1.081	1.034	2244	1716	1391
A88614	1.209	1.289	1.369	2245	4266	5305
M83082	1.208	1.212	1.207	2246	3209	3227
M89009	1.208	1.208	1.200	2247	3165	3128
C83023	1.208	1.155	1.110	2248	2489	2093
A86006	1.208	1.135	1.194	2249	2272	3049
L83608	1.208	1.101	1.061	2250	1912	1637
C84077	1.208	1.126	1.121	2251	2181	2212
C86018	1.208	1.208	1.166	2252	3169	2718
C88022	1.208	1.210	1.311	2253	3197	4620
G81097	1.208	1.125	1.080	2254	2179	1819
P81100	1.208	1.220	1.170	2255	3300	2756
A82047	1.207	1.162	1.120	2256	2597	2204
N81064	1.207	1.173	1.145	2257	2734	2483
P88017	1.207	1.218	1.176	2258	3287	2823
B83039	1.207	1.274	1.255	2259	4066	3903
P86015	1.207	1.190	1.291	2260	2941	4376
N84618	1.207	1.139	1.132	2261	2333	2335
M91029	1.207	1.307	1.392	2262	4531	5549
H85029	1.207	1.059	0.996	2263	1548	1134
H82061	1.206	1.068	1.042	2264	1620	1476
P89016	1.206	1.251	1.212	2265	3756	3322
P88604	1.206	1.274	1.243	2266	4083	3749
E81038	1.206	1.108	1.066	2267	2016	1714
A83038	1.206	1.108	1.099	2268	2020	1995
P92019	1.206	1.186	1.167	2269	2908	2746
D82068	1.206	1.176	1.129	2270	2782	2320
N82103	1.206	1.219	1.301	2271	3318	4544
L81034	1.205	1.155	1.117	2272	2522	2194
M91015	1.205	1.128	1.117	2273	2226	2196
M83637	1.205	1.107	1.163	2274	2004	2702
J82215	1.205	1.170	1.173	2275	2730	2810
M85011	1.205	1.166	1.153	2276	2674	2598
E81073	1.205	1.042	1.017	2277	1425	1275
M87013	1.205	1.111	1.138	2278	2062	2425
F85015	1.205	1.018	1.053	2279	1249	1593
P91004	1.205	1.031	1.023	2280	1344	1334
C81110	1.205	1.056	1.020	2281	1536	1307
M88031	1.205	1.201	1.296	2282	3116	4486
C82027	1.204	1.214	1.163	2283	3262	2711
H85055	1.204	1.072	1.033	2284	1674	1425
P89006	1.204	1.147	1.155	2285	2449	2633
G83026	1.204	1.119	1.100	2286	2151	2031
A89042	1.204	1.272	1.257	2287	4079	3980
A81041	1.204	1.210	1.194	2288	3224	3104
M86017	1.204	1.137	1.150	2289	2350	2574
N81036	1.204	1.144	1.145	2290	2424	2522
P91623	1.204	1.063	1.050	2291	1599	1570
H82077	1.204	1.072	1.049	2292	1678	1556
G82107	1.204	1.229	1.198	2293	3478	3164

C86021	1.204	1.260	1.225	2294	3924	3537
H84050	1.204	1.130	1.076	2295	2261	1822
C84064	1.204	1.156	1.219	2296	2564	3455
N81071	1.203	1.133	1.092	2297	2291	1961
B81065	1.203	1.136	1.126	2298	2346	2317
M82018	1.203	1.114	1.123	2299	2117	2281
K81051	1.203	1.065	1.018	2300	1616	1304
A83030	1.203	1.149	1.125	2301	2487	2305
F83053	1.203	1.045	1.072	2302	1457	1784
C81638	1.203	1.181	1.179	2303	2874	2918
C83641	1.203	1.180	1.149	2304	2872	2578
M87026	1.203	1.114	1.176	2305	2123	2883
C82066	1.203	1.099	1.059	2306	1945	1678
A83012	1.203	1.315	1.271	2307	4702	4185
G82735	1.202	1.043	0.994	2308	1454	1149
M81082	1.202	1.090	1.037	2309	1874	1470
P81066	1.202	1.309	1.353	2310	4626	5197
K81023	1.202	1.077	1.042	2311	1749	1515
G82164	1.202	1.115	1.108	2312	2135	2138
K81056	1.202	1.044	1.060	2313	1461	1701
B85019	1.201	1.098	1.067	2314	1949	1755
N85057	1.201	1.131	1.179	2315	2303	2940
K83030	1.201	1.164	1.126	2316	2703	2333
M81605	1.201	1.124	1.083	2317	2229	1906
Y02322	1.201	1.213	1.233	2318	3309	3691
L85013	1.201	1.179	1.126	2319	2884	2343
B86004	1.201	1.104	1.104	2320	2025	2106
G82198	1.200	1.078	1.083	2321	1764	1909
L85616	1.200	1.161	1.107	2322	2670	2134
M82028	1.200	1.100	1.092	2323	1985	1985
C82112	1.200	1.107	1.071	2324	2081	1800
P81128	1.200	1.201	1.124	2325	3168	2327
A82050	1.200	1.218	1.179	2326	3389	2963
E82117	1.200	1.050	1.034	2327	1527	1465
P82005	1.200	1.165	1.151	2328	2738	2641
F81739	1.200	1.334	1.296	2329	4964	4549
G81676	1.200	1.140	1.228	2330	2434	3631
D81023	1.200	1.046	1.024	2331	1489	1383
M85749	1.200	1.025	1.033	2332	1331	1461
C81012	1.200	1.165	1.123	2333	2739	2328
K82032	1.199	1.054	1.026	2334	1559	1404
A83036	1.199	1.087	1.081	2335	1871	1894
N85059	1.199	1.104	1.087	2336	2049	1956
A82008	1.199	1.175	1.168	2337	2848	2828
P81699	1.199	1.163	1.308	2338	2718	4695
A87009	1.199	1.109	1.095	2339	2103	2025
P83611	1.199	1.082	1.119	2340	1819	2284
M85776	1.199	1.120	1.154	2341	2215	2677
D82043	1.199	1.148	1.112	2342	2524	2219
N84009	1.199	1.163	1.184	2343	2717	3043

C82046	1.199	1.141	1.087	2344	2448	1957
E82132	1.199	1.146	1.131	2345	2504	2422
H83033	1.199	1.065	1.105	2346	1654	2129
M83692	1.199	1.210	1.164	2347	3285	2787
K84058	1.199	1.034	1.011	2348	1416	1283
C86038	1.199	1.153	1.204	2349	2595	3318
P81018	1.199	1.206	1.190	2350	3242	3133
M83025	1.199	1.073	1.058	2351	1735	1705
K82021	1.199	1.087	1.043	2352	1882	1553
M85081	1.198	1.209	1.201	2353	3280	3288
G81077	1.198	1.082	1.008	2354	1823	1265
M87015	1.198	1.272	1.229	2355	4174	3666
B82066	1.198	1.095	1.067	2356	1947	1787
B84013	1.198	1.127	1.129	2357	2288	2411
B82062	1.198	1.174	1.227	2358	2858	3652
C88025	1.198	1.256	1.258	2359	3954	4063
N85633	1.197	1.083	1.143	2360	1840	2567
P81701	1.197	1.137	1.101	2361	2416	2109
G81048	1.197	1.090	1.093	2362	1910	2026
B86056	1.197	0.983	0.986	2363	1067	1124
C83061	1.197	1.185	1.174	2364	3001	2924
M88032	1.197	1.191	1.199	2365	3087	3285
L82026	1.197	1.257	1.215	2366	3973	3493
P83012	1.197	1.129	1.114	2367	2341	2264
C83653	1.197	1.066	1.040	2368	1685	1540
F84015	1.197	1.047	1.104	2369	1519	2150
M83632	1.197	1.181	1.137	2370	2960	2509
D83014	1.196	1.141	1.077	2371	2469	1889
H82641	1.196	1.261	1.268	2372	4041	4233
F81651	1.196	1.070	1.053	2373	1731	1676
H81021	1.196	1.002	0.984	2374	1190	1121
D82036	1.196	1.107	1.091	2375	2110	2012
A88006	1.196	1.434	1.344	2376	6040	5161
B86075	1.196	1.116	1.108	2377	2207	2206
C85008	1.196	1.174	1.147	2378	2886	2642
D81010	1.196	1.139	1.100	2379	2457	2119
N83620	1.196	1.283	1.358	2380	4364	5330
P88011	1.195	1.197	1.185	2381	3185	3101
E81636	1.195	1.223	1.229	2382	3513	3705
F81118	1.195	1.120	1.072	2383	2242	1863
C84066	1.195	1.210	1.174	2384	3342	2961
B86023	1.195	1.048	1.032	2385	1549	1493
C85009	1.195	1.150	1.166	2386	2596	2863
L81018	1.195	1.110	1.087	2387	2152	1989
K82013	1.195	1.174	1.142	2388	2900	2594
G83017	1.195	1.067	1.106	2389	1714	2190
L82021	1.194	1.147	1.135	2390	2568	2511
B82611	1.194	1.210	1.199	2391	3356	3306
H82035	1.194	1.020	0.997	2392	1340	1218
F82019	1.194	1.146	1.225	2393	2563	3676

B85061	1.194	1.103	1.089	2394	2101	2016
E86033	1.194	1.078	1.050	2395	1832	1668
C87010	1.194	1.261	1.313	2396	4072	4807
P89015	1.194	1.154	1.153	2397	2672	2728
J83027	1.194	1.225	1.207	2398	3569	3440
F81047	1.194	1.163	1.157	2399	2776	2773
P89012	1.193	1.249	1.250	2400	3922	4029
N83009	1.193	1.285	1.250	2401	4419	4026
M91612	1.193	1.263	1.324	2402	4126	4961
P86006	1.193	1.167	1.179	2403	2827	3052
A82015	1.193	1.202	1.176	2404	3254	3023
F82625	1.193	1.002	1.061	2405	1214	1780
L83021	1.193	1.089	1.080	2406	1941	1944
M87601	1.193	1.160	1.146	2407	2751	2659
P84637	1.193	1.295	1.410	2408	4565	5857
K81636	1.192	1.104	1.115	2409	2128	2314
F81087	1.192	1.167	1.132	2410	2840	2501
C86015	1.192	1.228	1.192	2411	3619	3243
K81025	1.192	1.065	1.033	2412	1718	1519
L81042	1.192	1.070	1.049	2413	1765	1679
N81073	1.192	1.109	1.066	2414	2170	1838
C88047	1.192	1.286	1.319	2415	4458	4915
B86062	1.192	1.307	1.315	2416	4729	4856
A87615	1.192	1.168	1.157	2417	2852	2786
D83062	1.192	1.271	1.224	2418	4242	3697
Y01794	1.192	1.133	1.107	2419	2436	2239
A83031	1.191	1.185	1.163	2420	3081	2868
P81061	1.191	1.253	1.277	2421	3999	4422
P82029	1.191	1.381	1.446	2422	5588	6154
L83646	1.191	1.264	1.309	2423	4161	4787
B83058	1.191	1.081	1.089	2424	1895	2043
B86001	1.191	1.146	1.202	2425	2598	3410
J84016	1.191	1.230	1.195	2426	3684	3305
Y02274	1.191	1.168	1.287	2427	2868	4546
B81011	1.191	1.178	1.319	2428	2997	4927
E86017	1.191	1.009	0.964	2429	1285	1019
C86626	1.191	1.098	1.167	2430	2092	2919
Y04333	1.191	1.022	1.010	2431	1382	1339
M83718	1.191	1.126	1.051	2432	2377	1717
G82137	1.191	1.052	1.028	2433	1615	1496
A87011	1.190	1.101	1.106	2434	2109	2234
A82608	1.190	1.095	1.032	2435	2045	1525
A81064	1.190	1.105	1.154	2436	2147	2777
P82640	1.190	1.092	1.169	2437	2009	2947
P85010	1.190	1.181	1.200	2438	3042	3377
P88005	1.190	1.186	1.153	2439	3099	2765
B83009	1.190	1.261	1.213	2440	4132	3549
H82050	1.190	1.123	1.103	2441	2347	2216
G84004	1.190	1.040	1.009	2442	1517	1336
L83020	1.190	1.141	1.078	2443	2544	1952

M81068	1.190	1.191	1.128	2444	3175	2492
Y02583	1.190	0.890	0.885	2445	662	638
P81095	1.190	1.203	1.261	2446	3315	4224
K83008	1.189	1.116	1.112	2447	2266	2316
B86002	1.189	1.133	1.116	2448	2465	2358
H81043	1.189	1.063	1.056	2449	1733	1767
G82186	1.189	1.094	1.056	2450	2053	1764
M83018	1.189	1.077	0.998	2451	1875	1256
N81050	1.189	1.095	1.030	2452	2073	1527
E86026	1.189	1.072	1.038	2453	1821	1599
E85023	1.189	1.147	1.172	2454	2632	3024
P81020	1.189	1.278	1.308	2455	4391	4803
E82018	1.189	1.068	1.034	2456	1783	1565
A88008	1.189	1.375	1.357	2457	5560	5376
G82117	1.189	1.110	1.083	2458	2214	2004
L83089	1.189	1.118	1.127	2459	2296	2495
F81030	1.189	1.134	1.110	2460	2476	2298
C82093	1.188	1.058	1.028	2461	1689	1512
H85028	1.188	0.970	0.928	2462	1039	826
P87020	1.188	1.160	1.212	2463	2803	3558
E85121	1.188	1.015	1.018	2464	1345	1435
E86020	1.188	1.003	0.993	2465	1257	1230
J82213	1.188	1.270	1.286	2466	4287	4564
K81087	1.188	1.068	1.050	2467	1791	1732
E81061	1.188	1.071	1.052	2468	1817	1752
F85663	1.187	1.053	1.100	2469	1655	2205
N81645	1.187	1.168	1.201	2470	2923	3437
B81658	1.187	1.116	1.190	2471	2289	3292
K83042	1.187	1.124	1.122	2472	2386	2440
M84018	1.187	1.098	1.070	2473	2112	1913
L83080	1.187	1.262	1.351	2474	4190	5342
A81608	1.187	1.260	1.344	2475	4170	5275
F86057	1.187	1.070	1.000	2476	1816	1291
B81090	1.187	1.239	1.265	2477	3861	4321
H81065	1.186	1.076	1.027	2478	1894	1517
H85112	1.186	0.898	0.876	2479	698	613
F82008	1.186	1.091	1.046	2480	2048	1707
B84002	1.186	1.064	1.037	2481	1772	1621
B86061	1.186	1.090	1.164	2482	2043	2936
D83045	1.186	1.199	1.164	2483	3307	2943
N82621	1.186	1.317	1.384	2484	4939	5685
C86019	1.186	1.120	1.112	2485	2366	2350
N83012	1.186	1.133	1.165	2486	2497	2969
L84015	1.186	1.067	1.062	2487	1806	1859
E81003	1.186	1.112	1.080	2488	2262	2006
F86026	1.186	1.063	1.098	2489	1766	2211
H85031	1.185	1.042	0.993	2490	1584	1250
Y00445	1.185	1.119	1.162	2491	2362	2929
G82087	1.185	1.136	1.103	2492	2539	2265
C83649	1.185	1.113	1.098	2493	2283	2213

G82063	1.185	1.159	1.125	2494	2821	2504
C82012	1.185	1.210	1.164	2495	3477	2964
C84127	1.185	1.280	1.286	2496	4473	4600
C87606	1.185	1.315	1.413	2497	4922	5959
F81200	1.185	1.189	1.262	2498	3206	4300
M88635	1.185	1.081	1.148	2499	1946	2762
A89016	1.184	1.201	1.241	2500	3377	4023
N83010	1.184	1.260	1.261	2501	4207	4292
Y00297	1.184	1.191	1.144	2502	3232	2730
P86021	1.184	1.141	1.170	2503	2627	3060
J81012	1.184	1.084	1.054	2504	1990	1798
P84673	1.184	1.141	1.245	2505	2626	4082
P83006	1.184	1.098	1.072	2506	2146	1958
H83008	1.184	1.065	1.109	2507	1798	2338
B82023	1.184	1.078	1.026	2508	1923	1529
N85620	1.184	1.213	1.216	2509	3535	3688
H85024	1.183	1.020	0.967	2510	1423	1089
M92607	1.183	1.118	1.126	2511	2371	2532
J82059	1.183	1.032	1.009	2512	1512	1401
N85028	1.183	1.237	1.195	2513	3877	3421
D82020	1.183	1.097	1.069	2514	2144	1935
B86667	1.183	1.143	1.196	2515	2657	3428
F81132	1.183	1.100	1.054	2516	2167	1806
N83017	1.183	1.134	1.224	2517	2543	3806
J83018	1.183	1.106	1.058	2518	2234	1839
C83611	1.183	1.089	1.070	2519	2070	1945
B84017	1.183	1.180	1.234	2520	3124	3951
K83041	1.183	1.013	1.003	2521	1367	1350
B81043	1.183	1.090	1.080	2522	2082	2036
B81092	1.183	1.257	1.255	2523	4184	4232
C84026	1.183	1.193	1.140	2524	3273	2693
E85035	1.183	1.095	1.074	2525	2132	1986
N81108	1.182	1.030	1.008	2526	1503	1402
Y01291	1.182	1.034	1.022	2527	1537	1513
J81018	1.182	1.113	1.085	2528	2311	2103
H81099	1.182	0.985	0.956	2529	1173	1029
J81609	1.182	1.199	1.154	2530	3380	2877
J84019	1.182	1.131	1.111	2531	2519	2384
B81022	1.182	1.189	1.179	2532	3235	3201
F81729	1.182	1.222	1.234	2533	3692	3969
M82604	1.182	1.084	1.048	2534	2019	1760
J83013	1.182	1.164	1.133	2535	2942	2637
M83007	1.181	1.133	1.162	2536	2548	2990
M91004	1.181	1.283	1.305	2537	4563	4871
N84626	1.181	1.086	1.105	2538	2051	2325
N82095	1.181	1.352	1.423	2539	5403	6068
G81012	1.181	1.193	1.131	2540	3301	2627
L81021	1.181	1.134	1.081	2541	2580	2067
B83659	1.181	1.288	1.359	2542	4619	5474
L84046	1.180	1.152	1.116	2543	2796	2460

A81631	1.180	1.243	1.259	2544	4011	4322
N84605	1.180	1.233	1.273	2545	3869	4495
M85679	1.180	1.041	1.059	2546	1606	1878
M84028	1.180	1.132	1.107	2547	2567	2361
L83128	1.180	1.144	1.139	2548	2726	2723
F82025	1.180	1.217	1.290	2549	3640	4702
L81124	1.180	1.111	1.061	2550	2334	1895
P81081	1.180	1.235	1.358	2551	3905	5477
D81086	1.179	1.100	1.040	2552	2212	1716
P86620	1.179	1.457	1.494	2553	6404	6557
A89026	1.179	1.150	1.160	2554	2785	2993
G83647	1.179	1.056	1.040	2555	1755	1721
M82601	1.179	1.153	1.110	2556	2828	2399
K83052	1.179	1.102	1.044	2557	2231	1754
G83049	1.179	1.141	1.089	2558	2691	2173
G83028	1.179	1.079	1.026	2559	1982	1579
M92024	1.179	1.079	1.062	2560	1992	1915
G85099	1.179	1.146	1.225	2561	2759	3886
B83056	1.179	1.268	1.260	2562	4401	4354
J81058	1.178	1.068	1.028	2563	1889	1612
P83010	1.178	1.262	1.344	2564	4304	5353
D81047	1.178	1.113	1.091	2565	2369	2208
J84008	1.178	1.168	1.142	2566	3032	2774
M86021	1.178	1.098	1.078	2567	2204	2070
D81044	1.178	1.141	1.084	2568	2699	2133
E81631	1.178	0.995	1.023	2569	1280	1560
P81083	1.178	1.176	1.121	2570	3137	2542
L83070	1.178	1.120	1.089	2571	2445	2180
K83018	1.178	1.061	1.010	2572	1824	1450
B86071	1.178	1.166	1.231	2573	3005	3976
G85104	1.178	1.180	1.151	2574	3192	2881
N85018	1.178	1.238	1.228	2575	3984	3936
K81078	1.178	1.048	1.035	2576	1697	1685
L81037	1.177	1.186	1.189	2577	3259	3405
M84070	1.177	0.987	0.978	2578	1215	1200
D83005	1.177	1.132	1.097	2579	2606	2286
M87034	1.177	1.119	1.190	2580	2451	3432
A86016	1.177	1.081	1.093	2581	2042	2247
Y00293	1.177	1.080	1.055	2582	2026	1869
C81604	1.177	1.286	1.322	2583	4646	5138
L83627	1.177	1.176	1.111	2584	3149	2442
B85001	1.177	1.158	1.142	2585	2926	2788
P89008	1.177	1.210	1.203	2586	3600	3601
F85072	1.176	1.013	1.002	2587	1426	1393
G82808	1.176	1.129	1.105	2588	2576	2381
J82015	1.176	1.093	1.066	2589	2163	1973
F81031	1.176	1.258	1.318	2590	4279	5105
N81039	1.176	1.172	1.113	2591	3103	2469
M85111	1.176	1.206	1.241	2592	3539	4135
N82074	1.176	1.145	1.244	2593	2772	4180

F82679	1.176	0.993	1.056	2594	1278	1884
F85678	1.176	1.023	1.077	2595	1495	2084
A87015	1.176	1.225	1.194	2596	3825	3506
L83125	1.175	1.039	1.030	2597	1632	1661
K81001	1.175	1.060	1.034	2598	1834	1706
Y02767	1.175	1.018	1.130	2599	1466	2674
P84679	1.175	1.090	1.174	2600	2155	3234
L83092	1.175	1.127	1.115	2601	2562	2505
L82618	1.175	1.238	1.190	2602	4021	3453
E82079	1.175	1.093	1.074	2603	2187	2058
B83032	1.175	1.146	1.196	2604	2794	3529
A81621	1.175	1.294	1.418	2605	4784	6083
J82034	1.175	1.122	1.080	2606	2498	2122
H82015	1.174	1.105	1.066	2607	2305	1982
P81053	1.174	1.200	1.246	2608	3480	4223
E81016	1.174	1.081	1.082	2609	2084	2156
G81661	1.174	1.096	1.186	2610	2219	3419
C87016	1.174	1.138	1.126	2611	2713	2645
Y02606	1.174	1.173	1.265	2612	3151	4481
C81104	1.174	1.153	1.142	2613	2889	2815
L82010	1.174	1.155	1.122	2614	2924	2613
J83010	1.173	1.098	1.069	2615	2248	2022
N82083	1.173	1.148	1.184	2616	2837	3388
P92002	1.173	1.125	1.143	2617	2558	2840
F82641	1.173	0.968	1.009	2618	1126	1479
H82031	1.173	1.100	1.096	2619	2268	2319
Y02735	1.173	1.200	1.301	2620	3501	4929
N85052	1.173	1.159	1.209	2621	2991	3734
C84061	1.173	1.158	1.198	2622	2977	3583
L81053	1.173	1.198	1.183	2623	3473	3393
D81622	1.173	1.160	1.166	2624	3002	3158
C88070	1.173	1.202	1.235	2625	3532	4094
P81082	1.173	1.128	1.079	2626	2605	2136
E82080	1.173	1.124	1.083	2627	2550	2175
C88009	1.172	1.327	1.378	2628	5213	5744
P88600	1.172	1.045	1.086	2629	1717	2225
G82666	1.172	1.250	1.206	2630	4225	3704
M85006	1.172	1.193	1.181	2631	3430	3353
M85756	1.172	1.068	1.110	2632	1942	2482
M84035	1.172	1.073	1.025	2633	2007	1642
D82632	1.172	1.241	1.362	2634	4100	5606
J81028	1.172	1.183	1.124	2635	3297	2648
Y03366	1.172	1.102	1.172	2636	2315	3261
D82046	1.172	1.156	1.134	2637	2953	2760
A86004	1.172	1.078	1.060	2638	2083	1962
K82033	1.172	1.119	1.073	2639	2505	2085
G82061	1.171	1.073	1.098	2640	2012	2364
L83097	1.171	1.060	1.023	2641	1881	1632
H81080	1.171	1.018	0.978	2642	1492	1240
F84681	1.171	0.984	1.013	2643	1242	1535

H81015	1.171	0.987	0.946	2644	1266	1035
C81004	1.171	1.113	1.070	2645	2442	2062
F81713	1.171	0.989	1.042	2646	1289	1810
P81003	1.171	1.147	1.121	2647	2860	2631
B87032	1.171	1.173	1.149	2648	3191	2945
D82070	1.170	1.177	1.154	2649	3234	3029
B81035	1.170	1.119	1.202	2650	2518	3677
P92637	1.170	1.108	1.152	2651	2400	3006
M81065	1.170	1.161	1.117	2652	3045	2591
D82034	1.170	1.127	1.094	2653	2623	2324
G82079	1.170	1.221	1.180	2654	3845	3386
G84005	1.170	1.134	1.087	2655	2722	2261
D82002	1.170	1.201	1.166	2656	3558	3194
M89021	1.170	1.192	1.159	2657	3434	3096
E81033	1.170	1.041	1.019	2658	1703	1609
Y00486	1.170	1.024	1.061	2659	1560	1981
C82075	1.170	1.122	1.088	2660	2570	2267
L83011	1.170	1.108	1.056	2661	2409	1938
M83139	1.170	1.178	1.187	2662	3248	3485
P82031	1.170	1.114	1.124	2663	2470	2666
C82023	1.170	1.128	1.154	2664	2643	3032
C82667	1.169	1.141	1.137	2665	2797	2813
D81085	1.169	1.056	1.021	2666	1859	1628
M83627	1.169	1.224	1.312	2667	3897	5114
F81091	1.169	1.179	1.158	2668	3265	3087
B84014	1.169	1.147	1.133	2669	2879	2778
F82604	1.169	1.021	1.029	2670	1545	1709
N83624	1.169	1.238	1.318	2671	4119	5172
G82073	1.169	1.028	1.007	2672	1598	1504
N82046	1.168	1.071	1.126	2673	2024	2710
M84004	1.168	1.072	1.031	2674	2031	1737
Y02321	1.168	1.148	1.174	2675	2896	3323
M87037	1.168	1.079	1.087	2676	2118	2275
M88024	1.168	1.086	1.112	2677	2182	2547
A83644	1.168	1.248	1.220	2678	4244	3964
B82036	1.168	1.107	1.085	2679	2415	2252
N82093	1.168	1.184	1.240	2680	3370	4229
L81038	1.168	1.084	1.063	2681	2159	2024
Y02688	1.168	0.954	0.937	2682	1077	991
G82799	1.168	1.141	1.146	2683	2817	2946
H85052	1.168	1.125	1.088	2684	2625	2287
M85652	1.168	1.007	1.042	2685	1438	1840
D81063	1.167	1.018	0.999	2686	1526	1449
G82206	1.167	0.968	1.026	2687	1162	1700
B85660	1.167	1.090	1.105	2688	2223	2477
L84028	1.167	1.298	1.261	2689	4943	4512
E86004	1.167	1.053	1.009	2690	1855	1530
M84061	1.167	1.074	1.062	2691	2080	2020
C83068	1.167	1.103	1.078	2692	2379	2183
Y02811	1.167	0.967	0.964	2693	1157	1177

M92630	1.167	1.310	1.439	2694	5064	6295
B86039	1.167	1.048	1.095	2695	1802	2375
K81607	1.167	1.062	0.986	2696	1936	1340
M92031	1.167	1.097	1.176	2697	2306	3363
F82017	1.166	1.113	1.109	2698	2493	2540
F86012	1.166	1.032	0.991	2699	1651	1389
J82094	1.166	1.078	1.033	2700	2130	1761
Y00028	1.166	1.060	1.095	2701	1921	2380
P92031	1.166	1.152	1.236	2702	2986	4206
N82107	1.166	1.138	1.199	2703	2802	3699
N81619	1.166	1.252	1.296	2704	4337	4957
B81024	1.166	1.022	1.000	2705	1572	1464
A81035	1.166	1.203	1.203	2706	3648	3745
L84043	1.166	1.097	1.077	2707	2327	2187
F84641	1.166	1.044	1.042	2708	1769	1848
H81119	1.166	1.040	0.986	2709	1738	1345
M85706	1.166	1.142	1.167	2710	2856	3268
F86637	1.166	1.053	1.079	2711	1862	2209
L83628	1.166	1.175	1.101	2712	3263	2448
M85801	1.165	1.047	1.082	2713	1799	2253
M83100	1.165	1.197	1.248	2714	3568	4368
C82024	1.165	1.051	0.988	2715	1850	1372
D82015	1.165	1.238	1.190	2716	4160	3570
C81025	1.165	1.110	1.083	2717	2478	2258
B84016	1.165	1.171	1.146	2718	3220	2992
P87002	1.165	1.204	1.219	2719	3682	3988
P81731	1.165	1.085	1.182	2720	2202	3477
L84031	1.165	1.145	1.098	2721	2907	2430
H82058	1.165	1.107	1.066	2722	2452	2077
M84059	1.165	1.152	1.105	2723	2998	2518
C81050	1.164	1.184	1.132	2724	3409	2817
N85008	1.164	1.093	1.057	2725	2286	1997
C84081	1.164	1.174	1.165	2726	3268	3263
B81021	1.164	1.226	1.316	2727	3998	5210
G81043	1.164	1.093	1.058	2728	2287	2007
P81063	1.164	1.304	1.349	2729	5032	5535
L82053	1.164	1.211	1.191	2730	3787	3607
M81627	1.164	1.094	1.048	2731	2299	1923
L85004	1.164	1.178	1.150	2732	3330	3058
N85616	1.164	1.161	1.223	2733	3128	4049
F85048	1.164	1.066	1.077	2734	2013	2223
P88607	1.164	1.218	1.319	2735	3885	5254
F85058	1.163	1.069	1.075	2736	2063	2195
N84029	1.163	1.215	1.234	2737	3854	4209
C81063	1.163	1.106	1.069	2738	2453	2130
E85718	1.163	0.975	0.970	2739	1233	1242
F86085	1.162	1.030	1.059	2740	1675	2032
L85008	1.162	1.126	1.080	2741	2708	2262
K82039	1.162	1.078	1.047	2742	2160	1929
D83018	1.162	1.162	1.130	2743	3155	2821

D83039	1.162	1.143	1.129	2744	2912	2808
F82677	1.162	1.086	1.159	2745	2241	3211
C88033	1.162	1.321	1.357	2746	5263	5647
L82018	1.162	1.151	1.130	2747	3015	2827
J81006	1.162	1.154	1.122	2748	3060	2739
A81013	1.162	1.161	1.113	2749	3148	2640
G82218	1.161	1.051	0.998	2750	1887	1492
P87016	1.161	1.079	1.085	2751	2185	2329
M85005	1.161	1.123	1.123	2752	2686	2747
L81026	1.161	1.093	1.077	2753	2345	2250
F81010	1.161	1.152	1.108	2754	3050	2595
L83057	1.160	1.155	1.106	2755	3085	2568
N82081	1.160	1.472	1.634	2756	6625	7157
C81095	1.160	1.116	1.082	2757	2604	2306
B81688	1.160	1.308	1.424	2758	5133	6239
J82041	1.160	1.211	1.165	2759	3836	3313
J82155	1.160	1.155	1.132	2760	3093	2870
G83031	1.160	1.082	1.073	2761	2220	2210
M81035	1.160	1.128	1.113	2762	2761	2661
A88005	1.160	1.243	1.227	2763	4306	4175
G81614	1.160	1.114	1.046	2764	2590	1936
C82031	1.160	1.091	1.057	2765	2331	2040
K82065	1.160	1.032	1.003	2766	1726	1543
P82001	1.159	1.232	1.203	2767	4150	3858
F81113	1.159	1.006	1.000	2768	1488	1523
C82114	1.159	1.066	1.127	2769	2086	2812
L82050	1.159	1.221	1.199	2770	4005	3790
D83029	1.159	1.105	1.100	2771	2486	2514
F86638	1.159	1.088	1.148	2772	2292	3097
A82617	1.159	1.106	1.073	2773	2494	2227
L81029	1.159	1.063	1.053	2774	2037	2008
H85683	1.159	1.048	1.033	2775	1884	1834
M83054	1.159	1.201	1.187	2776	3730	3640
K81075	1.159	1.084	1.055	2777	2250	2035
B86103	1.158	1.175	1.254	2778	3374	4543
N85058	1.158	1.079	1.056	2779	2205	2039
B87002	1.158	1.122	1.134	2780	2715	2914
M88008	1.158	1.142	1.159	2781	2954	3256
F81163	1.158	1.155	1.096	2782	3117	2481
G82754	1.158	1.114	1.072	2783	2617	2226
B85024	1.158	1.171	1.144	2784	3319	3055
E81027	1.158	1.199	1.151	2785	3713	3150
P91011	1.158	1.020	1.011	2786	1625	1640
P92634	1.158	1.055	1.089	2787	1952	2403
D82600	1.158	1.298	1.307	2788	5031	5183
N85003	1.158	1.212	1.165	2789	3881	3337
G82670	1.158	1.134	1.260	2790	2853	4613
H82092	1.158	1.061	1.025	2791	2032	1766
M81029	1.158	1.075	1.023	2792	2173	1757
B87004	1.158	1.190	1.169	2793	3588	3404

F86030	1.157	1.039	1.062	2794	1805	2111
P85020	1.157	1.173	1.168	2795	3366	3389
E82663	1.157	1.034	1.060	2796	1762	2097
M86612	1.157	1.086	1.132	2797	2282	2902
J82100	1.157	1.238	1.218	2798	4258	4080
K83015	1.157	1.124	1.091	2799	2746	2436
F82630	1.157	1.191	1.186	2800	3605	3628
C81101	1.157	1.127	1.112	2801	2774	2680
Y02517	1.157	1.117	1.095	2802	2668	2488
E81077	1.157	1.074	1.047	2803	2174	1971
G83062	1.157	1.094	1.052	2804	2393	2015
C82048	1.157	0.982	0.971	2805	1332	1298
M92029	1.157	1.192	1.186	2806	3616	3656
P81025	1.157	1.250	1.269	2807	4451	4726
B83061	1.157	1.169	1.193	2808	3312	3743
B82054	1.156	1.151	1.145	2809	3086	3086
C88050	1.156	1.259	1.266	2810	4571	4698
K83013	1.156	1.138	1.111	2811	2934	2671
N82090	1.156	1.230	1.260	2812	4176	4629
G82203	1.156	1.077	1.076	2813	2213	2282
L82004	1.156	1.129	1.091	2814	2818	2441
A81602	1.156	1.152	1.161	2815	3105	3307
L84080	1.156	1.151	1.118	2816	3092	2757
E85069	1.156	1.061	1.071	2817	2054	2232
P81710	1.156	1.161	1.145	2818	3218	3098
N84630	1.156	1.129	1.140	2819	2819	3040
J81004	1.155	1.055	1.023	2820	1979	1771
E81014	1.155	1.145	1.114	2821	3024	2720
K83020	1.155	1.064	1.149	2822	2097	3156
C86003	1.155	1.334	1.327	2823	5491	5416
K83039	1.155	1.199	1.161	2824	3743	3315
F81080	1.155	1.089	1.099	2825	2359	2546
P92023	1.155	1.232	1.211	2826	4211	4022
B86673	1.155	1.236	1.220	2827	4256	4133
F81134	1.155	1.219	1.164	2828	4040	3368
M85105	1.155	1.256	1.269	2829	4547	4744
J81644	1.155	1.002	1.018	2830	1496	1746
A83023	1.155	1.336	1.289	2831	5519	5018
C81086	1.155	1.054	1.026	2832	1978	1811
P81153	1.155	1.224	1.180	2833	4117	3589
P81117	1.154	1.216	1.210	2834	3997	4013
P81714	1.154	1.211	1.344	2835	3937	5601
A82010	1.154	1.184	1.204	2836	3537	3934
E86036	1.154	1.044	1.003	2837	1891	1596
L81044	1.154	1.145	1.099	2838	3046	2555
P89027	1.154	1.147	1.135	2839	3069	2997
H82025	1.154	1.070	1.054	2840	2161	2068
P81181	1.154	1.107	1.071	2841	2572	2256
J82187	1.154	1.219	1.204	2842	4045	3943
H81081	1.154	1.118	1.068	2843	2719	2229

A87002	1.154	1.300	1.248	2844	5101	4519
E83028	1.154	1.130	1.096	2845	2859	2529
G82015	1.154	1.118	1.100	2846	2723	2588
F81074	1.154	1.143	1.097	2847	3019	2543
L82056	1.154	1.147	1.115	2848	3074	2740
H81131	1.154	1.019	0.981	2849	1656	1417
F81186	1.153	1.164	1.174	2850	3282	3530
G83046	1.153	1.062	1.021	2851	2096	1769
N81018	1.153	1.044	0.998	2852	1896	1558
D82007	1.153	1.267	1.233	2853	4716	4340
G82060	1.153	1.137	1.116	2854	2957	2764
C85619	1.153	1.203	1.218	2855	3823	4121
L84022	1.153	1.127	1.124	2856	2824	2861
H81013	1.153	1.052	1.017	2857	1972	1745
J81055	1.153	1.195	1.162	2858	3732	3372
E82106	1.153	1.050	1.024	2859	1955	1801
G83029	1.153	1.029	1.001	2860	1757	1603
L81098	1.153	1.212	1.235	2861	3976	4372
B85621	1.153	1.336	1.421	2862	5534	6267
J82151	1.152	1.126	1.112	2863	2820	2729
J83039	1.152	1.109	1.071	2864	2615	2273
F84735	1.152	0.990	1.014	2865	1433	1723
B85612	1.152	1.322	1.298	2866	5385	5144
P81051	1.152	1.201	1.216	2867	3814	4105
J83601	1.152	1.058	1.041	2868	2077	1966
C84065	1.152	1.085	1.053	2869	2349	2075
K81644	1.152	1.020	1.014	2870	1679	1730
M83089	1.152	1.193	1.095	2871	3718	2537
M83052	1.152	1.014	0.985	2872	1629	1463
L83131	1.152	1.147	1.132	2873	3097	2989
A87008	1.152	1.066	1.062	2874	2148	2174
L84072	1.151	1.114	1.072	2875	2692	2288
H81667	1.151	1.018	0.981	2876	1663	1430
P85026	1.151	1.213	1.220	2877	3996	4192
A89012	1.151	1.202	1.184	2878	3848	3698
M82012	1.151	1.147	1.127	2879	3104	2912
C88084	1.151	1.289	1.357	2880	4995	5748
N83002	1.151	1.252	1.252	2881	4540	4602
P81721	1.151	1.217	1.328	2882	4053	5463
B82033	1.151	1.144	1.136	2883	3070	3044
C88061	1.151	0.993	0.999	2884	1465	1597
C83055	1.151	1.131	1.122	2885	2917	2858
M86633	1.151	1.062	1.087	2886	2127	2466
K81084	1.151	1.034	1.009	2887	1822	1704
D81050	1.151	1.142	1.128	2888	3048	2935
M85699	1.150	1.174	1.214	2889	3450	4108
K83076	1.150	1.099	1.177	2890	2515	3597
B83020	1.150	1.178	1.180	2891	3506	3653
C81027	1.150	1.219	1.190	2892	4099	3787
J81076	1.150	1.200	1.167	2893	3818	3470

F81097	1.150	1.077	1.065	2894	2264	2235
L82028	1.150	1.058	1.045	2895	2087	2017
M83148	1.150	1.086	1.058	2896	2384	2155
N85022	1.150	1.261	1.311	2897	4678	5303
Y02787	1.150	1.043	1.102	2898	1919	2646
M81054	1.150	1.134	1.086	2899	2956	2464
G82021	1.150	1.072	1.032	2900	2221	1902
L83003	1.150	1.227	1.197	2901	4222	3898
B86033	1.150	1.082	1.090	2902	2340	2513
Y03124	1.150	1.221	1.317	2903	4139	5369
J84010	1.150	1.222	1.192	2904	4151	3841
P92653	1.149	1.231	1.321	2905	4273	5405
C81611	1.149	1.076	1.053	2906	2263	2108
D81043	1.149	1.130	1.109	2907	2910	2738
P86026	1.149	1.166	1.194	2908	3375	3868
P81734	1.149	1.272	1.311	2909	4833	5323
L81006	1.149	1.089	1.083	2910	2425	2432
E85696	1.149	0.950	0.948	2911	1166	1190
K82008	1.149	1.021	0.975	2912	1715	1405
Y02660	1.149	1.230	1.297	2913	4274	5165
P83608	1.149	1.199	1.182	2914	3828	3700
A81004	1.149	1.179	1.199	2915	3549	3941
M82051	1.148	1.152	1.128	2916	3201	2980
F83678	1.148	1.225	1.307	2917	4212	5288
G82671	1.148	1.023	1.036	2918	1737	1951
G81694	1.148	1.021	1.113	2919	1719	2784
J82033	1.148	1.073	1.045	2920	2253	2033
C84144	1.148	1.078	1.057	2921	2302	2162
B85025	1.148	1.118	1.102	2922	2788	2664
P87008	1.148	1.222	1.224	2923	4180	4290
K83610	1.147	1.034	1.041	2924	1852	1999
F81152	1.147	1.122	1.061	2925	2829	2221
H81060	1.147	1.042	0.991	2926	1932	1548
G82118	1.147	1.123	1.084	2927	2850	2467
Y01839	1.147	1.139	1.220	2928	3051	4242
K83079	1.147	1.085	1.117	2929	2401	2844
J81612	1.147	1.256	1.172	2930	4651	3576
L81039	1.147	1.061	1.042	2931	2142	2023
F81758	1.147	1.208	1.225	2932	3986	4315
B85623	1.147	1.143	1.218	2933	3102	4217
C88038	1.147	1.172	1.208	2934	3468	4083
M83109	1.147	1.140	1.215	2935	3079	4190
M85171	1.147	1.058	1.091	2936	2126	2548
G82698	1.147	1.029	1.054	2937	1815	2143
G81050	1.147	1.183	1.190	2938	3630	3846
C82043	1.147	1.117	1.084	2939	2784	2474
K82043	1.147	1.097	1.045	2940	2536	2053
G82160	1.146	1.140	1.144	2941	3082	3213
L82035	1.146	1.184	1.171	2942	3658	3585
J83049	1.146	1.172	1.137	2943	3476	3123

N85643	1.146	1.187	1.237	2944	3711	4477
M84001	1.146	1.162	1.125	2945	3361	2977
M83076	1.146	1.100	1.155	2946	2583	3362
F81125	1.146	1.097	1.044	2947	2547	2049
N82646	1.146	1.161	1.238	2948	3353	4500
G83635	1.146	1.048	1.091	2949	2011	2562
P81072	1.146	1.226	1.252	2950	4268	4668
E85058	1.145	1.102	1.065	2951	2621	2283
B85620	1.145	1.050	1.134	2952	2046	3090
M83625	1.145	1.182	1.249	2953	3641	4622
P89612	1.145	1.157	1.160	2954	3305	3446
F81108	1.145	1.136	1.125	2955	3047	2978
M82031	1.145	1.149	1.076	2956	3211	2406
G83043	1.145	1.095	1.091	2957	2532	2570
F82021	1.145	1.025	1.014	2958	1788	1789
L84023	1.145	1.159	1.090	2959	3333	2560
F84670	1.145	1.025	1.043	2960	1792	2046
P84682	1.145	1.087	1.137	2961	2454	3146
F81019	1.145	1.087	1.056	2962	2455	2191
P88013	1.145	1.144	1.107	2963	3159	2755
L83056	1.145	1.162	1.089	2964	3386	2545
E83003	1.144	1.030	0.977	2965	1845	1456
B84615	1.144	1.123	1.126	2966	2885	3004
P81215	1.144	1.261	1.275	2967	4755	4971
A83044	1.144	1.261	1.192	2968	4754	3905
K81034	1.144	1.039	1.014	2969	1931	1791
M81004	1.144	1.111	1.071	2970	2748	2366
P84684	1.144	1.118	1.137	2971	2825	3148
M87027	1.144	1.197	1.262	2972	3864	4797
P82015	1.144	1.171	1.161	2973	3496	3486
B86106	1.144	1.166	1.196	2974	3437	3971
H81128	1.144	1.031	0.997	2975	1867	1646
E85699	1.144	1.189	1.121	2976	3770	2926
B81656	1.144	1.386	1.427	2977	6089	6376
P81115	1.143	1.334	1.403	2978	5592	6198
C84646	1.143	1.067	1.031	2979	2237	1950
N81024	1.143	1.214	1.141	2980	4130	3214
B86006	1.143	1.124	1.110	2981	2911	2811
G82016	1.143	1.011	0.991	2982	1676	1586
B85018	1.143	1.226	1.233	2983	4297	4462
C84016	1.143	1.243	1.209	2984	4534	4167
N84023	1.143	1.358	1.358	2985	5798	5839
L85050	1.142	1.151	1.089	2986	3245	2589
M83141	1.142	1.146	1.122	2987	3204	2970
G85726	1.142	1.046	1.110	2988	2034	2820
M91604	1.142	1.251	1.250	2989	4644	4676
K82049	1.142	1.091	1.063	2990	2521	2300
F81092	1.142	1.045	1.074	2991	2030	2414
L81087	1.142	1.092	1.092	2992	2538	2626
F86064	1.142	1.041	1.057	2993	1973	2245

B86051	1.142	1.158	1.207	2994	3373	4142
P83620	1.142	1.105	1.138	2995	2704	3198
F86696	1.142	0.981	1.041	2996	1436	2061
A81056	1.142	1.172	1.178	2997	3552	3736
N82009	1.141	1.099	1.143	2998	2633	3276
B81068	1.141	1.129	1.078	2999	2996	2473
Y02319	1.141	0.966	1.044	3000	1328	2099
P82020	1.141	1.177	1.224	3001	3620	4366
M84069	1.141	1.100	1.094	3002	2652	2650
M83046	1.141	1.133	1.066	3003	3064	2337
E87663	1.141	1.006	1.041	3004	1648	2072
E82035	1.141	1.148	1.098	3005	3239	2701
F85684	1.141	1.110	1.116	3006	2771	2905
K83047	1.141	1.139	1.151	3007	3142	3379
L83027	1.141	1.149	1.142	3008	3252	3271
M84011	1.141	1.107	1.071	3009	2744	2400
M88013	1.141	1.206	1.180	3010	4048	3788
J82134	1.141	1.159	1.099	3011	3403	2722
M85694	1.140	1.203	1.284	3012	4015	5115
M87030	1.140	1.246	1.247	3013	4608	4663
L84001	1.140	1.081	1.032	3014	2430	1988
M87020	1.140	1.045	1.016	3015	2052	1852
F82643	1.140	1.020	1.027	3016	1787	1942
K81027	1.140	1.103	1.086	3017	2697	2575
L85021	1.140	1.114	1.049	3018	2826	2164
H81033	1.140	1.038	0.964	3019	1969	1384
C81020	1.140	1.238	1.153	3020	4506	3430
M83068	1.140	1.195	1.243	3021	3912	4627
J82180	1.140	1.107	1.068	3022	2754	2377
C81055	1.140	1.392	1.324	3023	6175	5542
P81700	1.140	1.166	1.257	3024	3486	4784
M84036	1.140	1.044	1.020	3025	2041	1880
J81051	1.140	1.177	1.143	3026	3662	3294
B83025	1.140	1.101	1.158	3027	2679	3500
K81077	1.139	1.128	1.117	3028	3011	2942
C84698	1.139	1.207	1.291	3029	4089	5205
G82080	1.139	1.088	1.047	3030	2520	2147
N81118	1.139	1.032	0.974	3031	1920	1472
M85663	1.139	1.024	1.058	3032	1839	2276
F86006	1.139	1.004	1.072	3033	1641	2424
D83076	1.139	1.095	1.076	3034	2620	2480
M82043	1.139	1.130	1.114	3035	3044	2909
Y02657	1.138	1.201	1.258	3036	4014	4815
J81043	1.138	1.128	1.076	3037	3034	2476
J81075	1.138	1.140	1.122	3038	3187	3028
K83064	1.138	1.083	1.086	3039	2471	2609
G83027	1.138	1.130	1.135	3040	3055	3210
G84007	1.138	1.069	1.028	3041	2326	1976
G82111	1.138	1.114	1.094	3042	2861	2684
K84028	1.138	1.088	1.054	3043	2528	2246

G83672	1.137	1.037	1.070	3044	1983	2421
M83726	1.137	1.023	0.986	3045	1842	1598
M83031	1.137	1.077	1.001	3046	2420	1747
P81712	1.137	1.205	1.275	3047	4084	5060
Y00542	1.137	1.131	1.151	3048	3090	3444
G82763	1.137	1.037	1.119	3049	1996	3007
B83037	1.137	1.217	1.208	3050	4263	4225
C81017	1.137	1.205	1.189	3051	4094	3970
J84004	1.137	1.116	1.082	3052	2894	2558
K81036	1.137	1.095	1.044	3053	2638	2140
N84043	1.137	1.224	1.312	3054	4357	5437
M88002	1.137	1.200	1.223	3055	4029	4425
B86034	1.137	1.077	1.075	3056	2429	2486
B81113	1.136	1.187	1.184	3057	3835	3909
C86029	1.136	1.154	1.164	3058	3390	3609
C86009	1.136	1.164	1.166	3059	3516	3650
E82630	1.136	1.007	0.990	3060	1702	1648
C84036	1.136	1.133	1.135	3061	3110	3228
A89009	1.136	1.184	1.173	3062	3804	3758
J81054	1.136	1.036	1.019	3063	1994	1914
N81079	1.136	1.096	1.052	3064	2656	2243
M92015	1.136	1.094	1.142	3065	2646	3329
L82066	1.136	1.198	1.183	3066	4007	3899
C88054	1.136	1.096	1.118	3067	2671	3012
B86059	1.135	1.107	1.110	3068	2801	2899
C82094	1.135	1.066	1.044	3069	2318	2166
M82005	1.135	1.077	1.062	3070	2440	2367
A81009	1.135	1.119	1.155	3071	2949	3522
F85035	1.135	1.023	0.992	3072	1873	1680
N81030	1.135	1.110	1.057	3073	2846	2311
L82012	1.135	1.127	1.103	3074	3065	2826
A81002	1.135	1.251	1.221	3075	4744	4421
M85016	1.135	1.165	1.196	3076	3550	4087
F81044	1.134	1.128	1.106	3077	3073	2865
D81623	1.134	1.109	1.095	3078	2842	2741
M88018	1.134	1.164	1.144	3079	3542	3371
M81033	1.134	1.095	1.039	3080	2681	2123
D81082	1.134	1.104	1.055	3081	2777	2291
G81685	1.134	1.143	1.176	3082	3261	3829
J81065	1.134	1.146	1.151	3083	3299	3480
D81033	1.134	1.081	1.060	3084	2496	2349
B87041	1.134	1.305	1.348	3085	5409	5828
E81074	1.134	1.106	1.082	3086	2806	2615
B86028	1.134	1.260	1.271	3087	4873	5058
B81047	1.133	1.220	1.366	3088	4345	6003
L85045	1.133	1.173	1.197	3089	3690	4106
H82032	1.133	1.059	1.041	3090	2255	2153
Y00159	1.133	1.202	1.171	3091	4103	3766
D82010	1.133	1.279	1.269	3092	5097	5023
H83048	1.133	1.111	1.158	3093	2880	3571

P84022	1.133	1.190	1.226	3094	3931	4501
C87007	1.133	1.104	1.111	3095	2793	2941
J82020	1.133	1.071	1.028	3096	2404	2014
G82092	1.133	1.038	1.004	3097	2059	1805
A81033	1.133	1.402	1.474	3098	6326	6709
G81037	1.133	1.036	0.990	3099	2023	1686
H85021	1.133	1.064	1.036	3100	2324	2107
J81052	1.133	1.195	1.173	3101	4016	3792
E81062	1.133	1.062	1.048	3102	2290	2237
P81006	1.132	1.088	1.064	3103	2612	2413
K82058	1.132	1.066	1.004	3104	2352	1807
M85056	1.132	1.175	1.164	3105	3738	3684
J84018	1.132	1.209	1.198	3106	4215	4155
M89024	1.132	1.141	1.161	3107	3256	3630
B83620	1.132	1.107	1.088	3108	2836	2682
M87023	1.132	1.131	1.108	3109	3144	2920
F82663	1.132	1.027	1.095	3110	1934	2772
L82046	1.132	1.090	1.064	3111	2645	2416
G85013	1.132	1.012	0.989	3112	1794	1684
M83009	1.132	1.110	1.042	3113	2878	2184
P81780	1.132	1.202	1.321	3114	4123	5593
L85011	1.132	1.095	1.077	3115	2712	2554
M83097	1.132	1.104	1.036	3116	2810	2113
K81017	1.132	1.113	1.102	3117	2931	2857
D82060	1.131	1.210	1.247	3118	4233	4768
G82035	1.131	1.114	1.089	3119	2945	2696
K84007	1.131	1.068	1.014	3120	2391	1912
G82124	1.131	1.211	1.125	3121	4240	3165
K82024	1.131	1.070	1.012	3122	2414	1887
F81131	1.131	1.125	1.083	3123	3077	2643
L85007	1.131	1.129	1.096	3124	3134	2791
N83003	1.131	1.390	1.394	3125	6249	6233
G84013	1.131	1.068	1.053	3126	2399	2309
P84644	1.131	1.051	1.133	3127	2210	3287
M85123	1.131	1.072	1.100	3128	2435	2834
G81011	1.130	1.153	1.181	3129	3439	3946
N85054	1.130	1.106	1.129	3130	2845	3222
P81646	1.130	1.170	1.164	3131	3694	3707
M84624	1.130	1.095	1.144	3132	2724	3441
N82067	1.130	1.174	1.254	3133	3749	4867
H83024	1.130	1.006	0.972	3134	1752	1534
P86023	1.130	1.250	1.308	3135	4799	5476
G81024	1.130	1.025	0.982	3136	1929	1630
K82066	1.130	1.115	1.078	3137	2969	2610
E85007	1.130	1.008	0.980	3138	1771	1618
C84053	1.130	1.159	1.156	3139	3527	3602
H82060	1.130	1.095	1.045	3140	2728	2236
G82121	1.130	1.149	1.172	3141	3413	3825
L81079	1.130	1.110	1.072	3142	2914	2527
C83041	1.129	1.172	1.207	3143	3734	4306

D83019	1.129	1.106	1.098	3144	2865	2829
F81111	1.129	1.097	1.044	3145	2757	2228
K82005	1.129	1.075	1.043	3146	2481	2217
A81048	1.129	1.157	1.126	3147	3519	3212
F81623	1.129	1.067	1.142	3148	2411	3433
F81216	1.129	1.060	1.015	3149	2320	1937
N81066	1.129	1.162	1.140	3150	3593	3415
G81074	1.128	1.167	1.173	3151	3673	3867
K83056	1.128	1.072	1.146	3152	2463	3494
F86655	1.128	0.935	0.956	3153	1192	1418
P85003	1.128	1.046	1.060	3154	2175	2409
C88024	1.128	1.068	1.083	3155	2421	2668
A86018	1.128	1.039	1.037	3156	2115	2167
L83115	1.128	1.214	1.137	3157	4336	3365
C85006	1.128	1.118	1.130	3158	3035	3274
C84046	1.128	1.212	1.174	3159	4311	3881
G82647	1.128	1.019	1.002	3160	1906	1836
N85634	1.128	1.470	1.497	3161	6787	6846
C83002	1.128	1.125	1.086	3162	3129	2721
M83727	1.127	1.227	1.263	3163	4525	5022
M86032	1.127	1.172	1.177	3164	3760	3923
J82008	1.127	1.127	1.084	3165	3157	2690
A89025	1.127	1.079	1.062	3166	2551	2455
C82061	1.127	1.158	1.136	3167	3566	3354
L82037	1.127	1.182	1.152	3168	3904	3584
C88043	1.127	1.081	1.098	3169	2593	2866
B81664	1.127	1.241	1.315	3170	4715	5586
H85018	1.127	1.010	0.977	3171	1826	1614
B82020	1.127	1.151	1.079	3172	3459	2654
K82011	1.127	1.006	0.968	3173	1784	1526
K84027	1.126	1.125	1.048	3174	3140	2302
C85004	1.126	1.155	1.154	3175	3521	3616
M88645	1.126	1.168	1.216	3176	3719	4465
P82626	1.126	1.257	1.270	3177	4937	5124
M86020	1.126	1.144	1.135	3178	3393	3357
G82221	1.126	1.039	1.034	3179	2137	2148
B87044	1.126	1.135	1.241	3180	3249	4755
A82071	1.126	1.152	1.209	3181	3483	4371
Y02626	1.126	1.185	1.266	3182	3958	5079
E82638	1.126	1.078	1.060	3183	2557	2431
Y02807	1.126	0.973	0.974	3184	1493	1590
J82184	1.126	1.195	1.164	3185	4116	3764
B82059	1.126	1.153	1.119	3186	3504	3162
P83023	1.125	1.110	1.104	3187	2970	2954
E86014	1.125	0.997	0.962	3188	1710	1485
J82206	1.125	1.042	0.994	3189	2166	1783
G82688	1.125	1.028	0.993	3190	2021	1774
B82069	1.125	1.079	1.051	3191	2585	2355
P81185	1.125	1.114	1.091	3192	3027	2796
M81034	1.125	1.107	1.042	3193	2936	2255

B83038	1.125	1.156	1.145	3194	3563	3519
P88009	1.125	1.309	1.323	3195	5544	5684
B83008	1.125	1.162	1.122	3196	3642	3219
L85046	1.125	1.267	1.210	3197	5052	4401
M81039	1.125	1.075	1.014	3198	2542	1968
N84004	1.125	1.205	1.237	3199	4247	4725
C83033	1.124	1.195	1.180	3200	4128	4012
B85008	1.124	1.095	1.155	3201	2786	3671
J82104	1.124	1.103	1.065	3202	2888	2510
M81001	1.124	1.176	1.172	3203	3852	3910
C83062	1.124	1.181	1.151	3204	3933	3605
J81010	1.124	1.210	1.123	3205	4330	3232
B86077	1.124	1.167	1.162	3206	3736	3767
H84630	1.124	0.951	0.972	3207	1353	1594
F81159	1.124	1.084	1.109	3208	2658	3045
M88639	1.124	1.184	1.251	3209	3972	4913
P81078	1.124	1.189	1.209	3210	4052	4407
C87004	1.124	1.301	1.288	3211	5484	5334
P82034	1.124	1.113	1.198	3212	3030	4256
E82019	1.124	1.055	1.042	3213	2322	2272
N83032	1.124	1.367	1.329	3214	6098	5747
F82031	1.124	1.033	1.094	3215	2100	2850
Y00996	1.123	0.865	0.910	3216	828	1116
M82029	1.123	1.200	1.187	3217	4213	4114
M81009	1.123	1.054	1.006	3218	2314	1917
M83725	1.123	1.168	1.202	3219	3763	4330
Y00912	1.123	1.184	1.272	3220	3990	5168
N82035	1.123	1.164	1.165	3221	3709	3821
F81012	1.123	1.119	1.089	3222	3114	2797
N81092	1.123	1.112	1.058	3223	3017	2459
M92609	1.123	1.162	1.253	3224	3680	4964
B81048	1.122	1.079	1.156	3225	2616	3702
C88008	1.122	1.125	1.160	3226	3198	3755
C87015	1.122	1.154	1.122	3227	3585	3242
M84007	1.122	1.145	1.036	3228	3446	2222
D81034	1.122	1.155	1.112	3229	3590	3110
B83657	1.122	0.952	1.049	3230	1366	2365
M83693	1.122	1.018	0.998	3231	1943	1851
M83072	1.122	1.153	1.118	3232	3557	3193
J82026	1.122	1.121	1.077	3233	3152	2679
G83025	1.122	1.111	1.066	3234	3029	2553
G82096	1.122	1.047	1.057	3235	2247	2453
C88648	1.121	1.176	1.162	3236	3893	3802
C82611	1.121	1.083	1.037	3237	2678	2244
M84042	1.121	1.071	0.993	3238	2531	1809
N82665	1.121	1.202	1.283	3239	4252	5322
L83136	1.121	1.175	1.101	3240	3879	2974
J82022	1.121	1.158	1.142	3241	3649	3525
E81022	1.121	1.037	1.010	3242	2157	1964
G82072	1.121	1.170	1.167	3243	3811	3891

L81036	1.121	1.190	1.153	3244	4115	3686
H82003	1.121	1.117	1.091	3245	3111	2848
B83642	1.121	1.151	1.225	3246	3543	4631
D82030	1.121	1.101	1.075	3247	2909	2662
A83061	1.121	1.083	1.089	3248	2690	2818
D83007	1.121	1.121	1.087	3249	3171	2804
G85052	1.121	1.028	1.090	3250	2093	2843
G82732	1.120	1.055	1.035	3251	2372	2231
P83005	1.120	1.292	1.298	3252	5411	5464
M92001	1.120	1.411	1.561	3253	6489	7081
N82077	1.120	1.370	1.425	3254	6154	6516
J82646	1.120	1.143	1.075	3255	3445	2672
E85077	1.120	0.985	1.048	3256	1644	2373
M92013	1.120	1.178	1.183	3257	3952	4101
B82628	1.120	1.296	1.302	3258	5467	5513
M85117	1.120	1.217	1.208	3259	4500	4441
B83626	1.120	1.081	1.095	3260	2685	2917
F81636	1.119	1.050	1.041	3261	2312	2301
B84011	1.119	1.146	1.157	3262	3492	3750
L83111	1.119	1.164	1.155	3263	3764	3732
J82083	1.119	1.183	1.185	3264	4032	4148
E81028	1.119	1.113	1.092	3265	3078	2878
H81017	1.119	1.008	0.955	3266	1877	1480
A83047	1.119	1.207	1.173	3267	4377	3984
C83031	1.119	1.213	1.178	3268	4452	4051
Y00025	1.119	1.208	1.305	3269	4388	5552
D83001	1.119	1.104	1.055	3270	2975	2465
L81024	1.119	1.133	1.111	3271	3322	3132
B81647	1.119	1.184	1.168	3272	4050	3921
L83054	1.119	1.236	1.150	3273	4761	3672
G85129	1.119	0.976	1.005	3274	1581	1934
M89019	1.119	1.096	1.046	3275	2864	2359
H83053	1.119	1.021	0.974	3276	2017	1670
C81028	1.119	1.109	1.078	3277	3039	2732
B87040	1.119	1.205	1.213	3278	4341	4517
E85117	1.119	1.120	1.137	3279	3186	3496
J83017	1.119	1.086	1.049	3280	2752	2396
E84083	1.118	1.013	0.950	3281	1935	1452
M88042	1.118	1.071	1.157	3282	2573	3779
E83624	1.118	1.007	0.976	3283	1886	1690
L83018	1.118	1.036	1.069	3284	2183	2635
P84064	1.118	1.242	1.321	3285	4849	5721
E81635	1.118	1.096	1.125	3286	2875	3332
B85041	1.118	1.226	1.202	3287	4629	4378
M84044	1.118	1.093	1.082	3288	2839	2769
F86702	1.118	0.980	0.960	3289	1618	1532
D83061	1.118	1.072	1.066	3290	2581	2607
E82640	1.118	1.128	1.095	3291	3267	2925
M81022	1.118	1.045	1.024	3292	2274	2137
M83035	1.118	1.039	1.018	3293	2216	2071

D83035	1.118	1.149	1.125	3294	3567	3333
A83013	1.117	1.142	1.105	3295	3469	3079
M91625	1.117	1.082	1.152	3296	2725	3710
C83032	1.117	1.133	1.111	3297	3354	3155
E86018	1.117	1.117	1.064	3298	3161	2585
M87629	1.117	1.206	1.297	3299	4392	5493
M91019	1.117	1.272	1.238	3300	5222	4843
E84007	1.117	1.040	0.970	3301	2236	1649
C84025	1.117	1.079	1.037	3302	2701	2280
M87003	1.117	1.087	1.055	3303	2787	2489
C83025	1.117	1.127	1.085	3304	3270	2825
K84034	1.117	1.041	0.987	3305	2240	1804
B81006	1.117	1.359	1.281	3306	6088	5346
C87006	1.117	1.237	1.243	3307	4798	4900
G82809	1.116	1.049	1.033	3308	2337	2254
N81063	1.116	1.157	1.103	3309	3706	3065
H82034	1.116	1.137	1.109	3310	3421	3144
B81023	1.116	1.165	1.209	3311	3801	4499
P81107	1.116	1.133	1.096	3312	3367	2986
Y00155	1.116	1.033	1.086	3313	2168	2851
B87039	1.116	1.179	1.173	3314	4026	4030
M92003	1.116	1.228	1.268	3315	4691	5208
L83107	1.116	1.245	1.327	3316	4911	5796
C88018	1.116	1.097	1.087	3317	2929	2867
P87610	1.116	1.179	1.171	3318	4028	4010
B83002	1.116	1.029	1.029	3319	2139	2220
J82133	1.116	1.216	1.231	3320	4537	4777
H82008	1.115	1.079	1.055	3321	2711	2500
L85607	1.115	1.101	1.090	3322	2978	2897
M87621	1.115	1.108	1.168	3323	3061	3975
M81003	1.115	1.151	1.116	3324	3623	3264
G84627	1.115	1.026	0.998	3325	2114	1916
M83669	1.115	1.174	1.292	3326	3963	5448
G82056	1.115	1.068	1.045	3327	2569	2388
F85026	1.115	1.161	1.160	3328	3774	3865
Y00411	1.115	1.380	1.368	3329	6296	6149
M81608	1.115	1.194	1.180	3330	4235	4119
N81029	1.115	1.147	1.090	3331	3583	2901
M83126	1.115	1.167	1.243	3332	3853	4930
D83058	1.115	1.116	1.114	3333	3178	3237
B83014	1.115	1.123	1.115	3334	3247	3259
A81046	1.115	1.156	1.127	3335	3724	3423
H84062	1.114	1.026	0.988	3336	2120	1831
K81020	1.114	1.034	0.994	3337	2197	1883
A81001	1.114	1.239	1.229	3338	4856	4760
H82042	1.114	1.134	1.117	3339	3411	3284
J82113	1.114	1.109	1.128	3340	3091	3442
B86107	1.114	1.092	1.104	3341	2883	3115
F81009	1.114	1.072	1.017	3342	2642	2104
E85727	1.114	1.019	1.049	3343	2055	2457

J83029	1.114	1.142	1.084	3344	3524	2854
P85016	1.114	1.086	1.088	3345	2809	2896
B85606	1.113	1.245	1.251	3346	4947	5052
N81082	1.113	1.149	1.090	3347	3628	2927
J82166	1.113	1.149	1.112	3348	3618	3230
M84034	1.113	1.031	1.100	3349	2184	3061
L85006	1.113	1.132	1.078	3350	3398	2792
J83045	1.113	1.094	1.066	3351	2913	2657
A88001	1.113	1.233	1.262	3352	4788	5176
K84047	1.113	1.138	1.093	3353	3465	2987
P83024	1.113	1.180	1.238	3354	4080	4882
L84009	1.113	1.051	1.014	3355	2405	2086
K82048	1.113	1.205	1.156	3356	4420	3839
C84011	1.113	1.128	1.145	3357	3338	3690
H85682	1.113	1.043	1.028	3358	2298	2233
G83642	1.113	1.073	1.042	3359	2667	2385
B86666	1.113	1.150	1.212	3360	3643	4584
J82053	1.112	1.177	1.131	3361	4038	3503
Y02838	1.112	1.128	1.184	3362	3344	4222
B81014	1.112	1.308	1.245	3363	5645	4995
F81119	1.112	1.083	1.069	3364	2790	2688
M85164	1.112	1.057	1.086	3365	2479	2884
B85634	1.112	1.131	1.124	3366	3399	3411
F81142	1.112	1.140	1.096	3367	3507	3035
M83125	1.112	1.132	1.096	3368	3415	3036
J82139	1.112	1.086	1.045	3369	2833	2428
P81689	1.112	1.199	1.170	3370	4355	4043
B86067	1.112	1.097	1.092	3371	2979	2985
M87008	1.112	1.086	1.069	3372	2832	2695
P83009	1.112	1.093	1.081	3373	2927	2836
H81005	1.112	1.088	1.024	3374	2855	2201
P81096	1.112	1.155	1.147	3375	3737	3724
N82669	1.112	1.415	1.479	3376	6576	6838
B85004	1.112	1.223	1.217	3377	4679	4652
E86609	1.112	1.037	1.089	3378	2259	2938
D82063	1.112	1.192	1.125	3379	4260	3438
M88633	1.112	1.095	1.155	3380	2950	3857
H84030	1.111	0.999	0.962	3381	1864	1620
M89010	1.111	1.023	0.973	3382	2121	1726
F84642	1.111	0.915	0.950	3383	1178	1507
M84047	1.111	1.076	1.040	3384	2721	2372
G82037	1.111	1.046	1.011	3385	2368	2066
C82080	1.111	1.143	1.198	3386	3580	4435
P81772	1.111	1.174	1.140	3387	4012	3625
Y00918	1.111	1.045	1.073	3388	2357	2754
F81099	1.111	1.066	1.021	3389	2603	2172
E83037	1.111	1.099	1.095	3390	3013	3037
A87019	1.111	1.155	1.187	3391	3761	4295
K83012	1.111	1.045	1.053	3392	2367	2528
L81062	1.111	1.101	1.098	3393	3033	3071

C84131	1.111	1.165	1.122	3394	3891	3409
C88040	1.110	1.121	1.136	3395	3271	3581
J81090	1.110	1.280	1.243	3396	5394	4994
B82028	1.110	1.231	1.183	3397	4808	4246
J81038	1.110	1.208	1.218	3398	4511	4682
A88016	1.110	1.144	1.109	3399	3608	3229
F84050	1.110	1.010	0.992	3400	1981	1903
E84702	1.110	1.030	1.058	3401	2199	2600
G81040	1.110	1.095	1.039	3402	2967	2390
H82014	1.110	1.126	1.080	3403	3368	2853
M86046	1.110	1.149	1.211	3404	3693	4605
M91613	1.110	1.069	1.145	3405	2650	3720
F84739	1.110	0.988	1.005	3406	1777	2019
B85055	1.109	1.079	1.090	3407	2770	2988
M86013	1.109	1.097	1.153	3408	3000	3860
C82055	1.109	1.067	1.040	3409	2630	2401
H82029	1.109	1.108	1.097	3410	3138	3072
P85001	1.109	1.106	1.180	3411	3115	4219
K81026	1.109	1.028	1.009	3412	2186	2063
H85009	1.109	0.960	0.931	3413	1522	1365
F81088	1.109	1.108	1.125	3414	3153	3462
C82663	1.109	1.095	1.078	3415	2988	2831
K83031	1.109	1.105	1.093	3416	3108	3034
C83022	1.109	1.120	1.072	3417	3290	2768
C84683	1.109	1.172	1.218	3418	4025	4699
K83066	1.109	1.196	1.185	3419	4372	4281
B81077	1.109	1.148	1.177	3420	3695	4183
H81095	1.109	1.047	1.000	3421	2410	1980
N81093	1.109	1.129	1.065	3422	3417	2691
M81005	1.108	1.195	1.128	3423	4352	3510
C82001	1.108	1.062	1.028	3424	2578	2279
N81047	1.108	1.090	1.043	3425	2930	2449
M92011	1.108	1.038	1.033	3426	2313	2339
Y00050	1.108	1.131	1.193	3427	3447	4405
C87008	1.108	1.182	1.177	3428	4182	4193
B86096	1.108	1.282	1.349	3429	5441	6055
G82224	1.108	1.028	1.006	3430	2211	2055
M91614	1.108	1.219	1.285	3431	4682	5462
M83075	1.108	1.097	1.131	3432	3022	3562
M91647	1.107	1.268	1.275	3433	5281	5363
B81017	1.107	1.211	1.335	3434	4576	5961
G84003	1.107	1.105	1.072	3435	3136	2790
F85632	1.107	0.993	1.027	3436	1849	2277
K82053	1.107	1.079	1.034	3437	2804	2354
B84003	1.107	1.065	1.052	3438	2636	2551
A89038	1.107	1.163	1.192	3439	3911	4400
J82609	1.107	1.162	1.124	3440	3898	3473
A81025	1.107	1.156	1.183	3441	3810	4277
M82057	1.107	1.068	1.093	3442	2676	3054
F81001	1.107	1.145	1.103	3443	3668	3191

G82059	1.107	1.032	0.999	3444	2246	1992
C82107	1.107	1.070	1.036	3445	2707	2386
J81049	1.107	1.098	1.072	3446	3049	2789
N81042	1.107	1.089	1.049	3447	2933	2524
A82060	1.107	1.203	1.286	3448	4482	5484
C82103	1.107	1.144	1.092	3449	3646	3046
L83014	1.107	1.184	1.165	3450	4220	4044
L83038	1.107	1.067	1.033	3451	2659	2352
M86638	1.106	1.196	1.214	3452	4396	4674
L84075	1.106	1.238	1.218	3453	4946	4714
D83084	1.106	1.056	1.035	3454	2527	2387
P81073	1.106	1.329	1.329	3455	5885	5911
L85039	1.106	1.126	1.077	3456	3410	2855
D82005	1.106	1.346	1.272	3457	6067	5349
C82036	1.106	1.068	1.034	3458	2688	2370
H82043	1.106	1.245	1.173	3459	5013	4177
M89013	1.106	1.188	1.196	3460	4286	4469
B81056	1.106	1.119	1.148	3461	3321	3834
P91017	1.106	1.085	1.069	3462	2893	2770
N85015	1.106	1.159	1.166	3463	3878	4068
L84059	1.105	1.102	1.097	3464	3113	3125
H81026	1.105	1.074	1.052	3465	2767	2584
L81068	1.105	0.998	0.979	3466	1916	1832
H83039	1.105	1.117	1.099	3467	3302	3163
D82057	1.105	1.336	1.261	3468	5966	5252
Y01795	1.105	0.999	1.061	3469	1924	2686
B81666	1.105	0.995	1.023	3470	1893	2268
P81172	1.105	1.198	1.276	3471	4442	5403
G82125	1.105	1.051	1.009	3472	2485	2115
D83064	1.105	1.186	1.122	3473	4275	3479
B83050	1.105	1.244	1.206	3474	5020	4610
P88023	1.105	1.081	1.050	3475	2854	2566
K81656	1.104	1.029	1.021	3476	2249	2249
P81076	1.104	1.140	1.094	3477	3625	3105
L82045	1.104	1.121	1.105	3478	3379	3253
K82055	1.104	1.104	1.091	3479	3164	3063
H81613	1.104	0.959	0.896	3480	1567	1143
F81078	1.104	0.990	0.997	3481	1844	1998
J82079	1.104	1.118	1.092	3482	3327	3077
P81058	1.104	1.212	1.254	3483	4631	5189
B82101	1.104	1.128	1.114	3484	3461	3384
K83029	1.104	1.088	1.093	3485	2968	3095
J82142	1.103	1.044	0.994	3486	2431	1979
D81633	1.103	1.103	1.082	3487	3156	2952
M88016	1.103	1.401	1.451	3488	6529	6746
B84004	1.103	1.127	1.154	3489	3456	3944
G82228	1.103	1.134	1.116	3490	3556	3426
K82042	1.103	1.123	1.078	3491	3422	2903
H84608	1.103	1.020	1.014	3492	2176	2186
J81046	1.103	1.112	1.112	3493	3264	3361

Y02597	1.103	1.181	1.262	3494	4231	5293
J81005	1.103	1.207	1.162	3495	4582	4053
M83713	1.103	1.268	1.306	3496	5332	5727
K83027	1.103	1.089	1.097	3497	2994	3167
K83620	1.103	1.147	1.082	3498	3755	2981
F81075	1.102	1.145	1.121	3499	3735	3497
J83060	1.102	1.163	1.124	3500	3982	3540
F81215	1.102	1.030	0.955	3501	2273	1644
M83613	1.102	1.091	1.085	3502	3018	3014
P92020	1.102	1.232	1.252	3503	4924	5181
C86606	1.102	1.169	1.260	3504	4074	5277
G81023	1.102	1.138	1.068	3505	3637	2805
N83043	1.102	1.402	1.446	3506	6553	6728
E86029	1.102	1.055	1.019	3507	2577	2263
B83015	1.102	1.237	1.220	3508	4981	4801
M83047	1.102	1.151	1.210	3509	3815	4680
C83054	1.102	1.141	1.094	3510	3688	3140
P87039	1.101	1.303	1.294	3511	5683	5632
F84679	1.101	1.047	1.077	3512	2482	2911
E82048	1.101	1.064	1.025	3513	2693	2322
P88034	1.101	1.179	1.184	3514	4223	4374
C82009	1.101	1.128	1.085	3515	3503	3027
C83014	1.101	1.200	1.199	3516	4514	4560
K81042	1.101	1.061	1.035	3517	2666	2439
M91602	1.101	1.265	1.293	3518	5323	5631
G81085	1.101	1.169	1.096	3519	4086	3175
Y02887	1.101	0.972	1.079	3520	1699	2958
F81665	1.101	1.007	0.998	3521	2076	2041
F84043	1.101	1.079	1.137	3522	2877	3746
M86015	1.100	1.131	1.101	3523	3547	3260
C82003	1.100	1.029	1.016	3524	2278	2240
C82033	1.100	1.143	1.110	3525	3733	3369
F81704	1.100	1.059	1.018	3526	2640	2271
A82621	1.100	1.069	1.164	3527	2768	4115
P81013	1.100	1.195	1.178	3528	4462	4314
D83054	1.100	1.136	1.121	3529	3622	3526
C82007	1.100	1.116	1.081	3530	3357	2994
E81009	1.100	1.128	1.095	3531	3518	3178
G81017	1.100	1.165	1.143	3532	4055	3835
K83070	1.100	1.132	1.081	3533	3587	3003
N83635	1.100	1.095	1.178	3534	3094	4325
K82022	1.100	1.066	1.007	3535	2749	2146
M83034	1.100	1.256	1.219	3536	5237	4812
P88002	1.099	1.092	1.082	3537	3063	3011
E84708	1.099	1.045	1.087	3538	2483	3082
J84015	1.099	1.060	1.045	3539	2662	2563
P88632	1.099	1.189	1.177	3540	4395	4305
F81632	1.099	1.219	1.183	3541	4802	4390
G82200	1.099	1.062	1.030	3542	2698	2404
A82045	1.099	1.213	1.192	3543	4724	4505

L81122	1.099	1.136	1.110	3544	3645	3407
L83096	1.099	1.176	1.134	3545	4217	3716
F84032	1.099	0.960	0.980	3546	1617	1898
C88037	1.099	1.135	1.167	3547	3629	4186
H82615	1.098	1.055	1.054	3548	2619	2678
P89025	1.098	1.119	1.214	3549	3424	4762
C81029	1.098	1.210	1.155	3550	4684	4034
B83622	1.098	1.038	1.109	3551	2417	3399
K83011	1.098	1.114	1.115	3552	3360	3484
B81112	1.098	1.106	1.224	3553	3246	4904
K81616	1.098	1.137	1.161	3554	3681	4104
L84616	1.098	1.096	1.098	3555	3132	3246
H85038	1.098	1.087	1.024	3556	3021	2353
G81103	1.098	1.120	1.246	3557	3441	5163
P87027	1.098	1.131	1.242	3558	3591	5134
B81055	1.098	1.144	1.229	3559	3778	4979
P81774	1.098	1.236	1.226	3560	5003	4935
J82007	1.098	1.130	1.085	3561	3577	3076
J82214	1.098	1.127	1.091	3562	3533	3154
A82048	1.097	1.125	1.092	3563	3509	3173
G81036	1.097	1.066	1.062	3564	2764	2785
G83004	1.097	1.088	1.046	3565	3043	2616
L82068	1.097	1.153	1.145	3566	3918	3901
B83010	1.097	1.215	1.233	3567	4768	5036
J81027	1.097	1.183	1.156	3568	4344	4055
D82084	1.097	1.138	1.099	3569	3707	3272
J83037	1.097	1.037	1.009	3570	2428	2198
N84018	1.097	1.093	1.061	3571	3106	2775
H81664	1.097	0.977	0.969	3572	1786	1820
N82037	1.097	1.177	1.178	3573	4248	4359
M86030	1.097	1.047	1.029	3574	2540	2418
C88041	1.097	1.059	1.047	3575	2695	2630
C82102	1.096	1.111	1.093	3576	3331	3202
B82074	1.096	1.144	1.080	3577	3795	3033
G81663	1.096	1.044	1.097	3578	2512	3250
N82004	1.096	1.256	1.250	3579	5277	5237
G82020	1.096	1.193	1.170	3580	4487	4261
P83609	1.096	1.102	1.121	3581	3225	3577
F82015	1.096	1.110	1.177	3582	3335	4356
L83004	1.096	1.108	1.074	3583	3310	2959
M83127	1.095	1.126	1.175	3584	3560	4341
L81008	1.095	1.133	1.113	3585	3656	3488
C84092	1.095	1.111	1.189	3586	3352	4515
P85017	1.095	1.210	1.202	3587	4730	4660
C84091	1.095	1.179	1.215	3588	4323	4821
J81068	1.095	1.168	1.106	3589	4167	3413
H81054	1.095	1.092	1.018	3590	3122	2323
Y03663	1.095	0.983	0.965	3591	1879	1797
M85070	1.094	1.114	1.106	3592	3412	3420
K83026	1.094	1.109	1.120	3593	3341	3586

L85012	1.094	1.103	1.043	3594	3253	2620
D83003	1.094	1.132	1.092	3595	3660	3215
F81214	1.094	1.131	1.094	3596	3644	3245
L83019	1.094	1.210	1.203	3597	4741	4687
M92044	1.094	1.189	1.230	3598	4471	5026
F81027	1.094	1.137	1.123	3599	3741	3627
L84047	1.094	1.083	1.015	3600	3023	2295
M89004	1.094	1.091	1.033	3601	3120	2497
D83619	1.094	1.094	1.096	3602	3163	3286
N85048	1.094	1.147	1.157	3603	3868	4111
B81109	1.094	1.115	1.089	3604	3433	3190
M85030	1.094	1.134	1.101	3605	3698	3342
P81150	1.094	1.120	1.058	3606	3488	2779
P84676	1.093	1.079	1.120	3607	2987	3603
Y02812	1.093	0.891	0.867	3608	1148	1025
E81046	1.093	1.068	1.033	3609	2841	2506
C84150	1.093	1.132	1.146	3610	3691	3978
G84025	1.093	1.080	1.041	3611	3003	2612
L83053	1.093	1.134	1.062	3612	3717	2839
B83627	1.093	1.131	1.218	3613	3674	4887
G82679	1.092	1.075	1.066	3614	2944	2890
D82027	1.092	1.245	1.207	3615	5194	4753
K82028	1.092	1.039	0.986	3616	2495	2000
N82076	1.092	1.145	1.173	3617	3866	4353
M82060	1.092	1.084	1.082	3618	3058	3102
F81095	1.092	1.155	1.127	3619	4020	3726
J83009	1.092	1.144	1.123	3620	3858	3665
M91033	1.092	1.151	1.239	3621	3964	5151
G83053	1.092	1.086	1.048	3622	3084	2689
D82050	1.092	1.105	1.072	3623	3323	2983
M83051	1.092	1.141	1.065	3624	3827	2882
M92008	1.092	1.087	1.067	3625	3100	2906
C82058	1.092	1.183	1.191	3626	4413	4591
B83017	1.091	1.155	1.139	3627	4037	3907
E81044	1.091	1.095	1.077	3628	3203	3051
L83005	1.091	1.198	1.156	3629	4622	4126
G85055	1.091	1.027	1.031	3630	2382	2507
M87011	1.091	1.105	1.074	3631	3325	3019
A89623	1.091	1.037	1.120	3632	2492	3632
K84063	1.091	0.963	0.941	3633	1712	1615
P92635	1.091	1.175	1.281	3634	4324	5605
D83009	1.091	1.147	1.114	3635	3923	3559
J82101	1.090	1.181	1.162	3636	4404	4220
D82011	1.090	1.128	1.127	3637	3665	3742
E82008	1.090	1.096	1.048	3638	3217	2705
E85716	1.090	1.001	0.988	3639	2111	2047
B82041	1.090	1.181	1.131	3640	4405	3812
C84717	1.090	1.218	1.352	3641	4899	6211
J82121	1.090	1.177	1.131	3642	4356	3803
G81051	1.090	1.102	1.011	3643	3298	2299

Y00079	1.090	1.088	1.133	3644	3141	3850
M84037	1.090	1.191	1.176	3645	4543	4418
M82023	1.090	1.101	1.093	3646	3284	3290
E83016	1.090	0.990	0.944	3647	1986	1655
N82091	1.090	1.220	1.302	3648	4933	5814
M83661	1.090	1.277	1.362	3649	5564	6296
B83023	1.089	1.181	1.165	3650	4421	4284
G82062	1.089	1.025	1.011	3651	2380	2303
H82047	1.089	1.075	1.066	3652	2983	2922
G85035	1.089	1.075	1.063	3653	2984	2891
D83081	1.089	1.155	1.135	3654	4057	3875
K82044	1.089	1.033	0.999	3655	2468	2169
C84123	1.089	1.307	1.256	3656	5830	5364
F81034	1.089	1.093	1.041	3657	3210	2653
F81213	1.089	1.196	1.179	3658	4628	4464
N83033	1.089	1.227	1.228	3659	5001	5072
G85005	1.088	1.144	1.111	3660	3921	3552
M85026	1.088	1.048	1.015	3661	2653	2362
C82054	1.088	1.124	1.100	3662	3627	3422
P88617	1.088	1.291	1.245	3663	5697	5272
C81616	1.088	1.157	1.131	3664	4101	3844
G82032	1.088	1.066	1.022	3665	2881	2437
C84018	1.088	1.143	1.189	3666	3906	4596
G83628	1.088	0.987	0.966	3667	1977	1871
M91032	1.088	1.155	1.200	3668	4069	4718
J82619	1.088	1.139	1.157	3669	3856	4194
C82010	1.088	1.118	1.044	3670	3551	2683
H81050	1.088	1.015	0.990	3671	2270	2092
J82099	1.087	0.993	0.940	3672	2066	1636
B87030	1.087	1.224	1.228	3673	4993	5088
H85115	1.087	1.151	1.115	3674	4039	3610
N82104	1.087	1.235	1.279	3675	5126	5624
P81176	1.087	1.258	1.235	3676	5407	5157
F84727	1.087	1.076	1.093	3677	3026	3326
L84058	1.087	1.185	1.152	3678	4510	4131
A82651	1.087	1.019	0.972	3679	2336	1931
E82001	1.087	1.041	1.019	3680	2592	2419
H81615	1.087	0.991	0.981	3681	2039	2003
J82073	1.087	1.123	1.158	3682	3624	4228
E82088	1.087	1.121	1.082	3683	3612	3185
J83002	1.086	1.119	1.104	3684	3584	3491
P87658	1.086	1.139	1.237	3685	3865	5191
L84016	1.086	1.150	1.129	3686	4035	3849
M83619	1.086	1.360	1.517	3687	6356	7042
Y00984	1.086	1.135	1.120	3688	3803	3709
C87005	1.086	1.222	1.197	3689	4985	4712
E84011	1.086	1.019	1.040	3690	2348	2663
M85115	1.086	1.069	1.119	3691	2946	3703
E81001	1.086	1.036	1.099	3692	2530	3431
F81112	1.086	1.048	1.054	3693	2687	2819

K84060	1.086	1.017	1.042	3694	2328	2687
F86642	1.085	1.037	1.073	3695	2554	3074
F86013	1.085	1.021	0.982	3696	2378	2037
G81002	1.085	1.134	1.150	3697	3807	4129
L85001	1.085	1.114	1.059	3698	3530	2887
D83610	1.085	1.078	1.067	3699	3071	3002
P91021	1.085	1.131	1.128	3700	3772	3854
L82011	1.085	1.172	1.164	3701	4367	4338
H82048	1.085	1.213	1.159	3702	4905	4259
B81622	1.085	1.138	1.121	3703	3867	3739
A83024	1.085	1.278	1.245	3704	5621	5301
H81134	1.085	1.057	1.014	3705	2812	2383
C82610	1.085	1.026	1.027	3706	2439	2531
M81617	1.085	1.190	1.219	3707	4597	5017
H81072	1.085	1.089	1.042	3708	3208	2704
G82069	1.084	1.088	1.090	3709	3205	3321
E83030	1.084	1.039	0.990	3710	2594	2124
G83022	1.084	1.072	1.162	3711	3007	4309
E84690	1.084	0.948	0.968	3712	1634	1918
G81053	1.084	1.275	1.190	3713	5597	4649
J83618	1.084	1.050	1.036	3714	2745	2651
M81090	1.084	1.083	1.049	3715	3143	2793
M89012	1.084	1.301	1.238	3716	5823	5229
J82169	1.084	1.072	1.037	3717	3014	2660
B81060	1.084	1.429	1.322	3718	6807	6034
B86109	1.084	0.985	1.093	3719	2001	3367
K82027	1.084	1.048	1.015	3720	2727	2405
G85662	1.083	0.981	1.047	3721	1954	2766
B83049	1.083	1.183	1.203	3722	4523	4824
J84014	1.083	1.120	1.103	3723	3647	3516
C82039	1.083	1.024	0.988	3724	2433	2117
K81076	1.083	0.981	0.937	3725	1961	1656
F81222	1.083	1.053	1.049	3726	2783	2800
K83010	1.083	1.114	1.117	3727	3548	3708
M82041	1.083	1.111	1.085	3728	3510	3280
L84033	1.083	1.116	1.093	3729	3595	3390
H81091	1.083	1.060	0.995	3730	2871	2202
A86010	1.082	1.069	1.083	3731	2990	3249
P85011	1.082	1.127	1.160	3732	3758	4308
H81028	1.082	1.063	1.003	3733	2915	2293
N82092	1.082	1.106	1.104	3734	3455	3533
M87024	1.082	1.184	1.142	3735	4558	4058
M84017	1.082	1.051	1.030	3736	2769	2606
P81132	1.082	1.222	1.200	3737	5030	4799
M83697	1.082	1.251	1.292	3738	5375	5790
G82156	1.082	1.052	1.087	3739	2780	3317
L81128	1.082	1.175	1.172	3740	4446	4466
G82768	1.082	1.083	1.076	3741	3179	3174
L81043	1.082	1.275	1.222	3742	5613	5087
K82076	1.082	1.041	1.020	3743	2651	2494

P88019	1.082	1.164	1.175	3744	4284	4514
K81041	1.081	1.068	1.059	3745	2989	2939
P82012	1.081	1.225	1.236	3746	5073	5244
C86007	1.081	1.163	1.198	3747	4276	4779
J84017	1.081	1.191	1.171	3748	4661	4459
G81087	1.081	1.097	1.026	3749	3369	2559
C81080	1.081	1.176	1.153	3750	4463	4235
N85005	1.081	1.155	1.112	3751	4178	3674
N82106	1.081	1.198	1.225	3752	4767	5121
J81025	1.081	1.147	1.073	3753	4063	3137
C88092	1.081	1.150	1.124	3754	4118	3838
M87620	1.081	1.021	1.028	3755	2426	2597
E82073	1.081	1.030	1.022	3756	2525	2520
E87057	1.081	1.036	1.060	3757	2609	2971
J84012	1.081	1.060	1.042	3758	2895	2744
H81056	1.081	1.059	1.014	3759	2887	2433
C84084	1.080	1.121	1.059	3760	3700	2951
B82011	1.080	1.210	1.220	3761	4919	5077
L84057	1.080	1.099	1.059	3762	3404	2949
L81030	1.080	1.088	1.071	3763	3241	3114
M85781	1.080	1.087	1.100	3764	3230	3518
P91627	1.080	1.195	1.169	3765	4723	4451
C88085	1.080	1.210	1.205	3766	4929	4891
L85005	1.080	1.215	1.171	3767	4973	4473
D81012	1.080	1.050	0.995	3768	2781	2230
E81060	1.080	1.054	1.063	3769	2835	3018
M82030	1.080	1.219	1.212	3770	5022	4997
M83133	1.079	1.088	1.188	3771	3258	4681
P83004	1.079	1.127	1.101	3772	3793	3538
M88030	1.079	1.368	1.426	3773	6478	6741
C86011	1.079	1.165	1.185	3774	4340	4648
L83100	1.079	1.105	1.108	3775	3479	3642
P81704	1.079	1.191	1.222	3776	4686	5113
C84063	1.079	1.119	1.135	3777	3699	4020
G81100	1.079	1.165	1.126	3778	4349	3904
E86625	1.079	1.008	1.040	3779	2294	2748
H84058	1.079	1.052	0.989	3780	2816	2176
E84699	1.079	1.109	1.111	3781	3541	3693
H81090	1.079	1.079	1.029	3782	3166	2628
B87031	1.078	1.162	1.146	3783	4314	4170
M87006	1.078	1.238	1.247	3784	5274	5381
L84050	1.078	1.079	1.041	3785	3174	2763
E86012	1.078	1.062	1.030	3786	2952	2644
P81691	1.078	1.013	1.076	3787	2373	3217
B82017	1.078	1.188	1.180	3788	4656	4603
B83006	1.078	1.177	1.161	3789	4520	4370
M83015	1.078	1.213	1.180	3790	4980	4607
H81023	1.078	1.037	0.974	3791	2644	2028
L82052	1.078	1.122	1.102	3792	3754	3566
P84046	1.078	1.104	1.193	3793	3482	4767

L83120	1.078	1.133	1.099	3794	3900	3528
D81087	1.078	1.197	1.170	3795	4785	4492
B84618	1.078	1.251	1.306	3796	5435	5968
P88007	1.078	1.053	1.039	3797	2843	2751
P89019	1.077	1.294	1.325	3798	5816	6108
E81049	1.077	1.109	1.085	3799	3581	3339
G82065	1.077	1.065	1.058	3800	2999	2999
C83024	1.077	1.132	1.101	3801	3895	3568
M81055	1.077	1.217	1.174	3802	5038	4557
B82068	1.077	1.111	1.107	3803	3606	3659
M85172	1.077	1.178	1.156	3804	4539	4336
D83015	1.077	1.252	1.210	3805	5454	4996
E85046	1.077	1.054	1.027	3806	2870	2624
M85156	1.077	1.151	1.228	3807	4185	5199
E81024	1.077	1.107	1.095	3808	3545	3502
M85753	1.077	1.297	1.358	3809	5848	6362
A82068	1.077	1.205	1.144	3810	4904	4171
M83012	1.077	1.198	1.107	3811	4822	3657
E81015	1.077	1.147	1.131	3812	4122	3994
A83630	1.077	1.258	1.228	3813	5520	5196
Y03362	1.077	1.045	1.162	3814	2763	4398
A81005	1.077	1.118	1.090	3815	3716	3436
L84053	1.076	1.147	1.111	3816	4135	3718
L85036	1.076	1.073	1.006	3817	3112	2394
P88606	1.076	1.107	1.112	3818	3559	3727
L84024	1.076	1.269	1.244	3819	5617	5368
B82100	1.076	1.218	1.188	3820	5050	4717
D81058	1.076	1.055	1.024	3821	2890	2614
N81087	1.076	1.130	1.083	3822	3875	3334
M88038	1.076	1.179	1.154	3823	4568	4312
H81044	1.076	1.118	1.074	3824	3725	3218
K83049	1.076	1.105	1.055	3825	3523	2972
D82058	1.076	1.218	1.187	3826	5053	4710
B86037	1.076	1.096	1.188	3827	3420	4719
E85012	1.076	1.173	1.213	3828	4503	5054
E85118	1.076	0.972	0.980	3829	1944	2112
D81612	1.076	1.103	1.071	3830	3502	3188
Y00304	1.075	1.096	1.055	3831	3427	2965
G81658	1.075	1.230	1.287	3832	5212	5809
C81018	1.075	1.087	1.053	3833	3291	2934
J81626	1.075	1.122	1.086	3834	3780	3402
J81056	1.075	1.162	1.131	3835	4362	4027
K81061	1.075	1.096	1.092	3836	3429	3476
H85032	1.075	1.025	1.029	3837	2537	2665
L81035	1.075	1.101	1.091	3838	3485	3463
D82059	1.075	1.195	1.148	3839	4796	4249
G82024	1.075	1.184	1.155	3840	4658	4342
J83630	1.074	1.148	1.126	3841	4169	3959
H83028	1.074	1.063	1.116	3842	3020	3816
F81105	1.074	1.091	1.083	3843	3378	3349

Y02827	1.074	1.076	1.162	3844	3184	4447
C84019	1.074	1.103	1.072	3845	3522	3216
E81072	1.074	1.075	1.045	3846	3173	2856
D81049	1.074	1.075	1.046	3847	3177	2873
M82025	1.074	1.150	1.111	3848	4202	3741
C83017	1.074	1.094	1.044	3849	3426	2852
G82012	1.074	1.059	1.055	3850	2964	2991
N82014	1.074	1.154	1.158	3851	4255	4382
J82149	1.074	1.069	1.047	3852	3096	2880
L83036	1.074	1.147	1.082	3853	4177	3360
P92643	1.074	1.227	1.283	3854	5203	5782
B84009	1.073	1.091	1.088	3855	3392	3447
N84001	1.073	1.030	1.012	3856	2618	2487
F84619	1.073	1.021	1.102	3857	2510	3641
A83070	1.073	1.126	1.110	3858	3873	3752
K83025	1.073	1.049	1.039	3859	2857	2795
E83011	1.073	1.122	1.065	3860	3826	3145
P85602	1.073	1.023	1.071	3861	2533	3223
M88627	1.073	1.149	1.207	3862	4206	5011
B82004	1.073	1.071	1.044	3863	3135	2864
J82648	1.073	1.142	1.163	3864	4106	4467
J83048	1.073	1.179	1.148	3865	4617	4263
P92642	1.073	1.403	1.454	3866	6723	6891
F81021	1.073	1.262	1.239	3867	5585	5360
L85609	1.072	1.116	1.092	3868	3747	3512
C88035	1.072	1.123	1.112	3869	3837	3785
P89618	1.072	1.252	1.293	3870	5507	5882
D82604	1.072	1.176	1.128	3871	4581	4019
B83064	1.072	1.220	1.181	3872	5124	4684
F81006	1.072	1.190	1.168	3873	4769	4547
B81057	1.072	1.140	1.262	3874	4091	5618
F82666	1.072	1.072	1.077	3875	3162	3316
E85107	1.072	1.059	1.046	3876	3006	2893
G82099	1.072	1.033	1.007	3877	2677	2451
N82662	1.071	1.160	1.236	3878	4390	5344
N82648	1.071	1.099	1.188	3879	3500	4780
D81028	1.071	1.051	1.025	3880	2897	2658
B86093	1.071	1.156	1.196	3881	4322	4877
L81009	1.071	1.206	1.189	3882	4969	4786
C82095	1.071	1.111	1.095	3883	3686	3561
M83049	1.071	1.169	1.115	3884	4507	3856
P82633	1.071	1.135	1.203	3885	4033	4982
E86042	1.071	1.026	0.995	3886	2602	2326
L81085	1.071	1.166	1.106	3887	4464	3721
G82234	1.071	1.201	1.151	3888	4918	4344
M88041	1.071	1.156	1.242	3889	4327	5396
M83038	1.071	1.152	1.166	3890	4265	4527
B87003	1.071	1.166	1.179	3891	4469	4679
P84070	1.071	1.162	1.188	3892	4412	4790
B82079	1.071	1.088	1.071	3893	3385	3257

Y02790	1.070	1.017	1.095	3894	2488	3574
L81055	1.070	1.167	1.128	3895	4480	4037
J82058	1.070	1.081	1.028	3896	3276	2708
F85039	1.070	0.968	0.958	3897	1962	1955
N81027	1.070	1.095	1.053	3898	3466	3022
D83006	1.070	1.069	1.005	3899	3158	2443
F81084	1.070	1.151	1.113	3900	4261	3853
C88083	1.070	1.052	1.090	3901	2938	3521
J83059	1.070	1.153	1.124	3902	4312	3991
H85025	1.070	1.114	1.095	3903	3757	3578
G82162	1.070	1.129	1.114	3904	3968	3871
E85682	1.070	1.093	1.139	3905	3449	4199
M85084	1.070	1.157	1.138	3906	4375	4184
F81184	1.069	1.070	1.017	3907	3167	2602
F86009	1.069	1.012	0.990	3908	2458	2304
L83047	1.069	1.155	1.116	3909	4348	3895
H81034	1.069	1.039	1.017	3910	2778	2608
M83061	1.069	1.263	1.294	3911	5629	5935
F86034	1.069	1.022	0.997	3912	2579	2369
L82070	1.069	1.095	1.062	3913	3491	3153
C81045	1.068	1.155	1.095	3914	4347	3599
F84121	1.068	1.108	1.048	3915	3689	2984
F81737	1.068	0.987	0.951	3916	2190	1910
G81088	1.068	1.072	1.028	3917	3207	2737
K83014	1.068	1.073	1.065	3918	3216	3200
B82105	1.068	1.163	1.114	3919	4474	3890
M85087	1.068	1.185	1.137	3920	4753	4195
B85059	1.068	1.119	1.123	3921	3842	4007
L83105	1.068	1.124	1.087	3922	3927	3508
E85712	1.068	1.038	1.100	3923	2779	3694
F81170	1.068	1.015	0.990	3924	2503	2307
L84010	1.068	1.181	1.135	3925	4708	4182
L83017	1.067	1.234	1.191	3926	5353	4869
B82075	1.067	1.130	1.089	3927	4024	3547
H85077	1.067	0.993	1.027	3928	2258	2734
A83071	1.067	1.167	1.135	3929	4536	4187
C81026	1.067	1.122	1.081	3930	3916	3443
G82704	1.067	1.105	1.112	3931	3667	3876
C81002	1.067	1.173	1.116	3932	4613	3928
Y02506	1.067	1.135	1.127	3933	4104	4069
K84005	1.067	1.105	1.037	3934	3676	2859
E85053	1.067	1.057	1.076	3935	3040	3378
E82072	1.066	1.153	1.131	3936	4351	4117
J83016	1.066	1.085	1.057	3937	3397	3120
G85009	1.066	1.386	1.399	3938	6671	6665
K81040	1.066	1.078	1.061	3939	3294	3179
B84008	1.066	1.072	1.076	3940	3222	3387
H81040	1.066	1.097	1.047	3941	3555	3000
B82010	1.066	1.114	1.066	3942	3790	3244
G85089	1.066	1.065	1.065	3943	3160	3240

L85017	1.066	1.136	1.109	3944	4124	3842
E81008	1.066	1.096	1.076	3945	3540	3394
M81048	1.065	1.277	1.215	3946	5773	5175
Y02466	1.065	1.025	1.011	3947	2655	2556
P81214	1.065	1.141	1.188	3948	4204	4848
P92626	1.065	1.265	1.308	3949	5665	6071
D81078	1.065	1.096	1.077	3950	3554	3414
B81064	1.065	1.174	1.173	3951	4641	4672
L81025	1.065	1.125	1.087	3952	3978	3545
M85624	1.065	1.140	1.126	3953	4191	4077
P84074	1.065	1.084	1.122	3954	3401	4031
M81091	1.065	1.135	1.095	3955	4114	3662
C86609	1.065	1.081	1.121	3956	3359	4018
K81082	1.065	1.002	0.985	3957	2390	2285
G81098	1.065	1.116	1.058	3958	3840	3160
C82076	1.065	1.026	1.002	3959	2683	2475
C82644	1.065	1.166	1.135	3960	4544	4201
F81721	1.065	1.017	0.986	3961	2560	2296
H83002	1.065	1.067	1.126	3962	3194	4085
M81089	1.064	1.170	1.193	3963	4611	4940
G82227	1.064	1.160	1.161	3964	4475	4553
P88028	1.064	1.102	1.070	3965	3653	3327
C81001	1.064	1.252	1.187	3966	5566	4854
H85103	1.064	1.136	1.128	3967	4149	4116
E82095	1.064	1.009	0.976	3968	2474	2203
P81002	1.064	1.216	1.201	3969	5170	5051
M84038	1.064	1.144	1.131	3970	4264	4176
L83045	1.064	1.094	1.049	3971	3546	3048
P92012	1.064	1.116	1.133	3972	3862	4197
J83011	1.064	1.183	1.136	3973	4794	4243
J81087	1.063	1.112	1.104	3974	3806	3793
C81022	1.063	1.216	1.150	3975	5193	4430
P88610	1.063	1.228	1.279	3976	5327	5849
E85750	1.063	0.984	0.942	3977	2209	1872
L84051	1.063	1.130	1.109	3978	4073	3889
M86037	1.063	1.027	1.074	3979	2720	3408
P81634	1.063	1.223	1.282	3980	5275	5868
C85005	1.063	1.307	1.284	3981	6099	5898
N85009	1.063	1.265	1.226	3982	5694	5329
M82010	1.063	1.088	1.055	3983	3474	3143
H82076	1.063	1.092	1.079	3984	3536	3465
N82099	1.063	1.297	1.392	3985	5994	6643
A82030	1.063	1.069	1.031	3986	3227	2822
C83029	1.063	1.163	1.122	3987	4532	4054
C84116	1.063	1.160	1.188	3988	4501	4892
D83067	1.062	1.099	1.100	3989	3634	3765
A81029	1.062	1.178	1.187	3990	4748	4880
N81032	1.062	1.073	1.038	3991	3289	2916
F81728	1.062	1.097	1.079	3992	3615	3478
C84142	1.062	1.149	1.136	3993	4354	4252

C84067	1.062	1.251	1.220	3994	5580	5284
A82650	1.062	1.134	1.098	3995	4143	3731
M81600	1.062	1.123	1.087	3996	3994	3593
H82045	1.061	1.178	1.153	3997	4756	4485
H85649	1.061	0.987	0.941	3998	2254	1881
N84013	1.061	1.144	1.120	3999	4290	4052
M91007	1.061	1.145	1.123	4000	4320	4090
M92026	1.061	1.038	1.058	4001	2867	3207
M87019	1.061	1.149	1.122	4002	4378	4076
H82021	1.061	1.083	1.056	4003	3435	3181
C84034	1.061	1.239	1.261	4004	5487	5694
H81027	1.061	0.998	0.973	4005	2392	2199
H81094	1.061	1.015	0.959	4006	2584	2056
H82009	1.061	1.130	1.115	4007	4108	3992
C83009	1.060	1.185	1.197	4008	4851	5046
B81036	1.060	1.117	1.089	4009	3941	3626
B81045	1.060	1.158	1.118	4010	4494	4040
A83048	1.060	1.102	1.111	4011	3723	3945
D81014	1.060	1.159	1.116	4012	4513	4008
G82142	1.060	1.075	1.055	4013	3345	3184
G82074	1.060	1.020	1.001	4014	2660	2517
J82135	1.060	1.067	1.015	4015	3238	2673
D82066	1.059	1.185	1.135	4016	4862	4273
H81075	1.059	1.071	1.038	4017	3303	2948
M85158	1.059	1.133	1.159	4018	4164	4585
C81075	1.059	1.097	1.081	4019	3659	3539
J81634	1.059	1.093	1.085	4020	3607	3588
P89014	1.059	1.228	1.215	4021	5382	5258
N82039	1.058	1.121	1.212	4022	4008	5225
F86020	1.058	0.947	0.947	4023	1876	1963
M84009	1.058	1.075	1.080	4024	3365	3532
A83054	1.058	1.254	1.243	4025	5643	5537
J81077	1.058	1.104	1.085	4026	3785	3618
E81025	1.057	1.176	1.252	4027	4772	5646
A82035	1.057	1.092	1.046	4028	3611	3089
L85611	1.057	1.108	1.085	4029	3847	3620
J82141	1.057	1.155	1.133	4030	4509	4283
N82655	1.057	1.284	1.321	4031	5929	6238
M88006	1.057	1.147	1.189	4032	4398	4988
H84016	1.057	1.040	0.997	4033	2948	2512
H82083	1.057	1.037	1.026	4034	2905	2842
E84022	1.057	1.056	1.026	4035	3146	2837
D83050	1.057	1.118	1.107	4036	3992	3948
N82100	1.057	1.146	1.180	4037	4393	4863
D81035	1.056	1.119	1.117	4038	4013	4073
J81019	1.056	1.123	1.099	4039	4061	3828
N83049	1.056	1.149	1.155	4040	4441	4575
P92029	1.056	1.117	1.132	4041	3988	4271
C83004	1.056	1.061	1.017	4042	3214	2749
C82088	1.056	1.052	1.033	4043	3109	2928

M91010	1.056	1.161	1.160	4044	4589	4635
M91011	1.056	1.068	1.094	4045	3304	3776
H82099	1.056	1.225	1.174	4046	5381	4796
M82024	1.055	1.063	1.051	4047	3243	3180
A82629	1.055	1.353	1.374	4048	6535	6583
B81655	1.055	1.217	1.347	4049	5300	6428
K84008	1.055	1.109	1.057	4050	3882	3283
P81032	1.055	1.214	1.228	4051	5262	5425
N82024	1.055	1.122	1.109	4052	4065	3990
E84013	1.055	1.067	1.044	4053	3295	3088
A82007	1.055	1.184	1.207	4054	4908	5206
J81048	1.055	1.010	0.990	4055	2599	2452
M81006	1.055	1.050	1.031	4056	3098	2921
E85739	1.055	1.020	1.013	4057	2736	2709
L81013	1.055	1.118	1.110	4058	4022	4000
N83614	1.055	1.082	1.153	4059	3505	4562
C82600	1.055	1.095	1.070	4060	3701	3457
E85006	1.054	1.066	1.068	4061	3288	3435
F83056	1.054	1.216	1.259	4062	5290	5739
G82711	1.054	1.075	1.132	4063	3428	4302
M91629	1.054	1.079	1.153	4064	3470	4572
C81658	1.054	1.070	1.099	4065	3364	3870
C82078	1.054	1.122	1.091	4066	4075	3744
Y02933	1.054	1.258	1.348	4067	5713	6445
G82105	1.054	1.099	1.069	4068	3762	3448
N81010	1.054	1.132	1.101	4069	4227	3897
M87001	1.054	1.067	1.046	4070	3313	3136
C83631	1.054	1.060	1.041	4071	3221	3069
A82018	1.054	1.137	1.144	4072	4296	4461
K83059	1.054	1.291	1.300	4073	6034	6102
J83064	1.054	1.111	1.109	4074	3940	4009
A82057	1.054	1.062	1.059	4075	3250	3310
M84030	1.054	1.120	1.100	4076	4059	3887
M91616	1.054	1.148	1.137	4077	4449	4379
F86703	1.054	1.022	1.006	4078	2765	2649
M85046	1.054	1.067	1.028	4079	3314	2894
E81069	1.054	1.294	1.246	4080	6073	5630
F81121	1.054	1.014	1.029	4081	2673	2915
M83624	1.054	1.394	1.459	4082	6779	6972
L83094	1.053	1.159	1.138	4083	4607	4391
L84615	1.053	1.085	1.063	4084	3572	3364
P89003	1.053	1.153	1.158	4085	4533	4638
J83030	1.053	1.108	1.084	4086	3903	3667
K81092	1.053	1.015	0.983	4087	2684	2395
C84124	1.053	1.136	1.132	4088	4300	4335
C84047	1.053	1.174	1.107	4089	4809	3987
G84016	1.053	1.042	1.015	4090	3016	2758
P88016	1.053	1.120	1.087	4091	4085	3714
K81089	1.053	1.023	1.060	4092	2791	3345
L81059	1.053	1.091	1.053	4093	3669	3247

K82030	1.052	1.067	0.997	4094	3336	2552
F81024	1.052	1.084	1.050	4095	3574	3220
A83043	1.052	1.160	1.138	4096	4635	4419
E82034	1.052	1.136	1.078	4097	4302	3596
L82013	1.052	1.178	1.148	4098	4869	4540
K83080	1.052	1.077	1.042	4099	3471	3109
G85021	1.052	1.023	1.079	4100	2792	3611
P91014	1.052	1.122	1.121	4101	4134	4196
P82625	1.052	1.054	1.097	4102	3193	3877
M83041	1.052	1.113	1.052	4103	3993	3254
P86002	1.052	1.286	1.376	4104	5999	6612
A86003	1.052	0.988	1.000	4105	2387	2604
E83044	1.051	1.007	0.936	4106	2601	1925
F82653	1.051	0.972	0.987	4107	2206	2462
L85026	1.051	1.142	1.137	4108	4409	4413
N82002	1.051	1.343	1.407	4109	6499	6783
E85126	1.051	0.981	0.928	4110	2295	1861
L83083	1.051	1.160	1.132	4111	4649	4350
P88042	1.051	1.066	1.034	4112	3340	3025
J82097	1.051	1.162	1.118	4113	4677	4174
K82009	1.051	1.068	1.035	4114	3376	3031
M87638	1.051	1.219	1.269	4115	5369	5861
M82050	1.051	1.154	1.142	4116	4578	4478
N82097	1.051	1.212	1.300	4117	5293	6128
E86629	1.051	0.991	0.991	4118	2422	2508
F86708	1.050	1.030	1.125	4119	2904	4258
N81028	1.050	1.095	1.089	4120	3753	3774
C83060	1.050	1.131	1.093	4121	4262	3843
A81611	1.050	1.126	1.157	4122	4195	4670
F84038	1.050	1.126	1.200	4123	4196	5193
P84611	1.050	0.973	1.042	4124	2230	3138
C83043	1.050	1.199	1.170	4125	5140	4826
H82095	1.050	1.155	1.137	4126	4598	4433
M88021	1.050	1.070	1.057	4127	3423	3340
L83145	1.050	1.279	1.217	4128	5946	5367
F86731	1.050	0.993	0.980	4129	2459	2398
M83066	1.050	1.163	1.179	4130	4709	4945
M86010	1.049	1.155	1.125	4131	4604	4268
J83644	1.049	1.041	1.118	4132	3053	4189
L82058	1.049	1.144	1.129	4133	4465	4337
E87756	1.049	0.995	1.035	4134	2484	3050
D81602	1.049	1.132	1.103	4135	4299	3989
Y02117	1.049	0.989	1.032	4136	2427	3013
L83138	1.049	1.106	1.122	4137	3934	4245
B82072	1.049	1.119	1.092	4138	4131	3848
N85617	1.049	1.055	1.078	4139	3231	3654
A88023	1.049	1.136	1.090	4140	4361	3810
M89005	1.049	1.122	1.052	4141	4165	3293
F83018	1.049	1.154	1.157	4142	4603	4690
F81038	1.048	1.117	1.035	4143	4092	3062

B85054	1.048	1.183	1.167	4144	4974	4810
F82650	1.048	0.977	1.028	4145	2284	2973
K83023	1.048	1.113	1.084	4146	4047	3729
P81173	1.048	1.105	1.097	4147	3939	3920
N81084	1.048	1.064	1.026	4148	3350	2931
J82157	1.048	1.097	1.062	4149	3809	3445
M83071	1.048	1.209	1.132	4150	5283	4387
F85676	1.048	0.987	0.971	4151	2413	2315
P84053	1.048	1.092	1.086	4152	3748	3759
J81062	1.048	1.108	1.085	4153	3987	3761
P84037	1.048	1.006	1.088	4154	2641	3796
K83614	1.047	1.245	1.272	4155	5652	5932
B82005	1.047	1.075	1.048	4156	3517	3252
P92001	1.047	1.198	1.235	4157	5163	5579
C84612	1.047	1.057	1.088	4158	3272	3807
J82112	1.047	1.119	1.090	4159	4146	3847
N81020	1.047	1.105	1.056	4160	3946	3370
F82609	1.047	1.118	1.113	4161	4138	4152
B81042	1.047	1.070	1.056	4162	3448	3373
E85021	1.047	1.038	0.995	4163	3054	2603
D82023	1.047	1.104	1.073	4164	3935	3598
B86025	1.047	0.986	0.990	4165	2418	2544
F81061	1.047	1.220	1.149	4166	5434	4614
L81031	1.046	1.074	1.049	4167	3515	3279
G85015	1.046	1.069	1.070	4168	3442	3556
E83633	1.046	1.139	1.167	4169	4444	4832
M81073	1.046	1.212	1.156	4170	5341	4706
P92024	1.046	1.168	1.207	4171	4820	5304
M82056	1.046	1.029	1.037	4172	2943	3119
C83016	1.046	1.124	1.184	4173	4230	5061
B83030	1.046	1.262	1.248	4174	5810	5712
Y02332	1.046	1.095	1.111	4175	3819	4144
H83031	1.046	1.079	1.113	4176	3598	4168
M85741	1.045	1.066	1.095	4177	3425	3925
L85051	1.045	1.139	1.094	4178	4447	3916
F81004	1.045	1.138	1.084	4179	4435	3778
L84008	1.045	1.114	1.099	4180	4102	3983
L82030	1.045	1.117	1.091	4181	4142	3878
L85601	1.045	1.114	1.110	4182	4112	4145
H81048	1.045	1.011	0.991	4183	2747	2582
F85027	1.045	1.071	1.041	4184	3489	3195
F84070	1.045	1.041	1.064	4185	3118	3514
P89602	1.045	1.139	1.186	4186	4457	5096
N82058	1.045	1.215	1.303	4187	5392	6186
L82622	1.045	1.059	1.057	4188	3329	3424
E83036	1.045	1.014	0.992	4189	2775	2601
B85005	1.044	1.029	1.004	4190	2962	2726
C87003	1.044	1.197	1.199	4191	5192	5242
J83053	1.044	1.071	1.042	4192	3497	3221
K83002	1.044	1.165	1.158	4193	4804	4739

D83069	1.044	1.141	1.132	4194	4484	4449
G82089	1.044	1.037	1.013	4195	3072	2835
B86035	1.044	1.269	1.229	4196	5899	5543
C88088	1.044	1.367	1.439	4197	6684	6935
Y02885	1.044	1.183	1.266	4198	5017	5903
C81034	1.044	1.106	1.082	4199	4001	3762
M81020	1.044	1.092	1.092	4200	3788	3913
H83050	1.044	1.034	1.007	4201	3036	2771
C82623	1.043	1.046	1.023	4202	3196	2979
A86008	1.043	1.131	1.113	4203	4373	4203
A87014	1.043	1.173	1.191	4204	4916	5160
D81017	1.043	1.061	1.009	4205	3395	2794
P91035	1.043	1.171	1.175	4206	4901	4987
P92030	1.043	1.070	1.090	4207	3494	3893
Y02960	1.043	0.964	1.052	4208	2208	3356
B81645	1.043	1.228	1.368	4209	5553	6621
H83021	1.043	1.056	1.079	4210	3320	3740
C83003	1.043	1.091	1.051	4211	3800	3348
E85026	1.043	1.006	0.984	4212	2709	2521
N81085	1.043	1.083	1.027	4213	3702	3042
H82064	1.043	1.115	1.121	4214	4158	4317
B86625	1.042	1.135	1.134	4215	4438	4483
B82078	1.042	1.130	1.092	4216	4363	3927
P92015	1.042	1.219	1.178	4217	5473	5033
F84017	1.042	1.045	0.989	4218	3195	2587
J82052	1.042	1.074	1.010	4219	3570	2816
L84026	1.042	1.097	1.062	4220	3884	3517
E82644	1.042	0.946	0.951	4221	2040	2163
J82147	1.042	1.024	0.999	4222	2937	2703
B81663	1.042	1.047	1.161	4223	3219	4813
F84696	1.042	1.180	1.175	4224	5012	4999
B87020	1.042	1.165	1.178	4225	4844	5035
C83074	1.042	1.188	1.143	4226	5105	4604
D83040	1.041	1.099	1.085	4227	3944	3859
A89028	1.041	1.164	1.172	4228	4829	4966
C81615	1.041	1.461	1.401	4229	7073	6802
H81039	1.041	1.014	0.980	4230	2813	2499
M87007	1.041	1.122	1.090	4231	4269	3915
K82038	1.041	1.123	1.058	4232	4282	3471
M88640	1.041	1.181	1.264	4233	5033	5909
F84658	1.041	1.053	1.070	4234	3296	3648
H83010	1.041	0.982	0.987	4235	2441	2580
P88623	1.041	1.127	1.159	4236	4342	4802
G82751	1.041	1.013	0.989	4237	2805	2617
N81125	1.041	1.109	1.051	4238	4087	3398
M84060	1.041	1.087	1.069	4239	3777	3629
G82002	1.041	1.129	1.108	4240	4382	4169
Y03363	1.040	1.051	1.165	4241	3275	4876
K83007	1.040	1.163	1.126	4242	4836	4411
M87010	1.040	1.235	1.221	4243	5627	5492

K81059	1.040	1.069	1.044	4244	3520	3300
D81022	1.040	1.095	1.076	4245	3890	3733
C81074	1.040	1.165	1.128	4246	4859	4444
J82069	1.040	1.088	1.041	4247	3798	3270
E83638	1.040	0.993	1.002	4248	2571	2750
M87025	1.040	1.276	1.347	4249	6013	6525
P89029	1.040	1.228	1.226	4250	5576	5568
L85010	1.040	1.226	1.183	4251	5562	5122
E84696	1.040	1.232	1.325	4252	5603	6385
L81027	1.040	1.055	1.019	4253	3346	2960
G85722	1.040	1.090	1.196	4254	3833	5259
P88625	1.040	1.190	1.159	4255	5152	4809
D83038	1.039	1.123	1.111	4256	4305	4231
G84017	1.039	1.056	1.023	4257	3371	3021
C81106	1.039	1.254	1.212	4258	5796	5423
L82022	1.039	1.242	1.202	4259	5698	5331
A86031	1.039	1.095	1.115	4260	3925	4289
C82669	1.039	1.099	1.171	4261	3971	4989
E84069	1.039	1.115	1.049	4262	4205	3381
M85055	1.039	1.023	1.021	4263	2951	3010
M85047	1.039	1.113	1.134	4264	4181	4528
G85124	1.039	1.163	1.148	4265	4853	4691
L81132	1.039	1.111	1.095	4266	4145	4021
B87013	1.039	1.216	1.213	4267	5485	5431
B82030	1.039	1.075	1.054	4268	3639	3459
E81031	1.039	1.112	1.097	4269	4166	4038
A83010	1.039	1.147	1.109	4270	4645	4214
C81076	1.038	1.084	1.067	4271	3766	3639
F81013	1.038	1.043	1.049	4272	3213	3385
F81102	1.038	1.034	0.964	4273	3107	2346
E82011	1.038	1.093	1.073	4274	3901	3723
A82031	1.038	1.164	1.090	4275	4865	3956
F81020	1.038	1.098	1.076	4276	3979	3754
M84016	1.038	1.085	1.040	4277	3786	3273
C84035	1.038	1.114	1.065	4278	4208	3604
C88019	1.038	1.160	1.143	4279	4830	4640
M86038	1.038	1.068	1.095	4280	3544	4035
B81063	1.038	1.089	1.082	4281	3843	3861
E83017	1.038	1.100	1.061	4282	4017	3555
F81733	1.038	1.258	1.408	4283	5850	6849
M81021	1.037	1.082	1.043	4284	3752	3319
N81006	1.037	1.062	1.032	4285	3467	3168
J83001	1.037	1.133	1.103	4286	4470	4150
A81007	1.037	1.264	1.281	4287	5916	6080
J83026	1.037	1.092	1.054	4288	3909	3483
E82027	1.037	1.039	1.025	4289	3189	3081
Y02181	1.037	1.162	1.174	4290	4867	5049
P91008	1.037	1.041	1.020	4291	3212	3015
M83094	1.037	1.176	1.213	4292	5027	5449
M85019	1.037	1.175	1.124	4293	5014	4436

E84077	1.037	1.047	1.075	4294	3278	3768
C88031	1.036	1.134	1.164	4295	4505	4924
L83069	1.036	1.187	1.143	4296	5153	4669
F85625	1.036	0.981	1.035	4297	2475	3231
B83029	1.036	1.293	1.267	4298	6233	5988
P92017	1.036	1.165	1.161	4299	4915	4881
J83043	1.036	1.067	1.037	4300	3562	3267
L82051	1.036	1.099	1.058	4301	4031	3543
D82096	1.036	1.177	1.164	4302	5044	4928
N84028	1.035	1.309	1.304	4303	6354	6268
G82600	1.035	1.203	1.280	4304	5368	6085
F81041	1.035	1.168	1.142	4305	4954	4665
F81674	1.035	1.026	1.059	4306	3038	3565
F84669	1.035	1.022	1.045	4307	2992	3383
H81057	1.035	1.091	1.052	4308	3926	3474
K83044	1.035	1.142	1.102	4309	4616	4173
F86045	1.035	1.054	1.143	4310	3400	4685
M83140	1.035	1.195	1.204	4311	5272	5380
G85121	1.035	1.016	1.094	4312	2918	4048
B87602	1.035	1.149	1.207	4313	4726	5421
J81041	1.035	1.109	1.092	4314	4189	4036
J83052	1.035	1.040	1.005	4315	3223	2846
M82019	1.034	1.050	1.033	4316	3355	3224
P81733	1.034	1.066	1.022	4317	3575	3080
B81088	1.034	1.093	1.053	4318	3962	3499
J83024	1.034	1.256	1.209	4319	5873	5435
P86001	1.034	1.202	1.246	4320	5362	5800
K82018	1.034	1.115	1.062	4321	4267	3617
K84052	1.034	1.073	1.043	4322	3685	3351
L84036	1.034	1.105	1.101	4323	4137	4166
K84075	1.034	1.071	1.025	4324	3638	3122
J81640	1.034	1.107	1.086	4325	4152	3958
P82002	1.034	1.180	1.187	4326	5106	5218
F81157	1.034	1.578	1.467	4327	7274	7056
J82208	1.034	1.175	1.191	4328	5041	5278
B86064	1.034	1.112	1.112	4329	4229	4326
L81050	1.033	1.085	1.079	4330	3860	3883
N83054	1.033	1.191	1.327	4331	5255	6446
C87029	1.033	1.213	1.195	4332	5509	5317
Y01163	1.033	1.226	1.263	4333	5609	5974
E83613	1.033	1.127	1.103	4334	4455	4207
G82810	1.033	1.217	1.224	4335	5541	5620
L81103	1.033	1.093	1.078	4336	3975	3874
L81655	1.033	1.006	0.986	4337	2822	2655
P84030	1.033	1.067	1.095	4338	3609	4092
N85006	1.033	1.071	1.057	4339	3666	3579
E82009	1.033	1.009	0.961	4340	2863	2379
M84063	1.032	1.065	1.036	4341	3589	3298
P81027	1.032	1.236	1.218	4342	5711	5564
K81645	1.032	1.039	1.062	4343	3237	3664

C87604	1.032	1.162	1.193	4344	4936	5314
L83123	1.032	1.198	1.303	4345	5340	6286
G81016	1.032	1.189	1.137	4346	5247	4646
P83612	1.031	1.162	1.180	4347	4934	5170
E82652	1.031	1.113	1.108	4348	4280	4288
N81043	1.031	1.096	1.054	4349	4043	3554
K83017	1.031	1.177	1.142	4350	5112	4711
D81053	1.031	1.134	1.113	4351	4572	4360
A82027	1.031	1.141	1.077	4352	4663	3884
G82036	1.031	1.088	1.057	4353	3942	3595
J82669	1.031	1.055	1.048	4354	3462	3487
E84678	1.031	0.949	0.965	4355	2192	2435
K84003	1.031	1.044	1.027	4356	3316	3205
L85023	1.030	1.057	1.000	4357	3487	2841
J82154	1.030	1.121	1.068	4358	4407	3757
M84603	1.030	1.169	1.143	4359	5011	4728
H83018	1.030	1.044	1.005	4360	3332	2907
M84031	1.030	1.099	1.123	4361	4105	4503
G84625	1.030	1.024	1.029	4362	3083	3236
G82064	1.030	1.126	1.119	4363	4485	4457
M86014	1.030	1.060	1.029	4364	3553	3235
L81082	1.030	1.091	1.079	4365	4000	3933
M92030	1.030	1.271	1.394	4366	6079	6821
M81604	1.030	1.176	1.145	4367	5104	4759
F81046	1.030	1.138	1.158	4368	4642	4933
G84023	1.029	1.096	1.059	4369	4062	3658
F82647	1.029	1.049	1.103	4370	3418	4255
J82054	1.029	1.078	1.071	4371	3816	3820
K82034	1.029	1.130	1.078	4372	4557	3931
L81649	1.029	0.989	0.988	4373	2663	2725
P88026	1.029	1.122	1.156	4374	4436	4923
P84004	1.029	1.179	1.204	4375	5160	5438
H82096	1.029	1.190	1.170	4376	5291	5101
E85034	1.029	1.079	1.126	4377	3832	4563
C83005	1.029	1.244	1.210	4378	5802	5503
N84006	1.029	1.202	1.158	4379	5425	4939
F81040	1.028	1.175	1.133	4380	5119	4637
B86019	1.028	1.013	1.038	4381	2965	3375
C81066	1.028	1.048	1.008	4382	3419	2975
A87600	1.028	1.079	1.071	4383	3838	3837
M85034	1.028	1.062	1.058	4384	3604	3660
B85058	1.028	1.116	1.182	4385	4379	5240
H82066	1.028	1.165	1.113	4386	4996	4416
C84060	1.028	1.182	1.165	4387	5210	5047
K82062	1.028	1.200	1.249	4388	5415	5899
K81046	1.028	1.071	1.020	4389	3746	3139
M85733	1.028	1.142	1.215	4390	4717	5576
P81182	1.028	1.189	1.248	4391	5294	5887
G84029	1.027	1.101	1.093	4392	4173	4143
J82128	1.027	1.177	1.175	4393	5146	5158

H81009	1.027	1.052	0.993	4394	3463	2802
B82013	1.027	1.056	1.031	4395	3528	3295
J82064	1.027	1.143	1.108	4396	4747	4355
M91611	1.027	1.198	1.237	4397	5410	5785
N81086	1.027	1.030	1.017	4398	3202	3112
C81041	1.027	1.141	1.111	4399	4711	4393
F81738	1.027	0.993	1.010	4400	2742	3026
M85154	1.027	1.059	1.051	4401	3576	3575
F84708	1.026	0.994	1.017	4402	2753	3118
D82042	1.026	1.145	1.117	4403	4771	4480
B87022	1.026	1.308	1.289	4404	6427	6222
A83057	1.026	1.390	1.379	4405	6901	6769
B83044	1.026	1.235	1.212	4406	5748	5556
J82065	1.026	1.070	1.024	4407	3751	3209
F82646	1.026	1.104	1.069	4408	4228	3852
K83040	1.026	1.146	1.149	4409	4787	4859
C82063	1.026	1.154	1.114	4410	4891	4452
F85654	1.026	1.209	1.245	4411	5530	5878
M89002	1.026	1.251	1.162	4412	5908	5045
C84003	1.025	1.146	1.124	4413	4807	4581
C85019	1.025	1.205	1.282	4414	5508	6173
F86001	1.025	1.052	1.081	4415	3493	4011
B82034	1.025	1.075	1.042	4416	3830	3472
L81063	1.025	1.147	1.118	4417	4827	4506
B85031	1.025	1.094	1.066	4418	4109	3817
L82054	1.025	1.056	1.032	4419	3571	3336
H81016	1.025	1.049	0.992	4420	3464	2814
L81022	1.025	1.130	1.124	4421	4601	4576
D83047	1.025	1.172	1.151	4422	5128	4901
J82144	1.025	1.056	1.014	4423	3573	3093
F86086	1.024	0.980	1.065	4424	2608	3800
L85028	1.024	1.263	1.213	4425	6062	5590
C88057	1.024	1.093	1.165	4426	4096	5083
M91018	1.024	1.287	1.349	4427	6277	6613
A83622	1.024	1.449	1.432	4428	7089	6982
M83048	1.024	1.083	1.005	4429	3961	2995
J82062	1.024	1.307	1.327	4430	6433	6505
M83027	1.024	1.147	1.084	4431	4832	4061
P92034	1.024	1.217	1.210	4432	5614	5558
C83075	1.024	1.073	1.018	4433	3813	3170
L85060	1.024	1.194	1.137	4434	5389	4729
M84021	1.024	1.047	1.034	4435	3443	3380
F85679	1.024	1.101	1.160	4436	4218	5042
G82727	1.024	1.126	1.170	4437	4559	5148
N81034	1.024	1.096	1.059	4438	4154	3728
C86605	1.024	1.248	1.290	4439	5890	6254
Y00278	1.024	1.203	1.280	4440	5504	6167
M85038	1.024	1.035	1.010	4441	3283	3068
K83625	1.023	1.090	1.086	4442	4067	4099
A83035	1.023	1.174	1.147	4443	5157	4870

C87621	1.023	1.066	1.119	4444	3742	4537
L84060	1.023	1.157	1.137	4445	4962	4742
M81024	1.023	1.109	1.097	4446	4333	4254
E85130	1.023	1.038	1.110	4447	3337	4437
C81084	1.023	1.142	1.105	4448	4786	4367
L83116	1.023	1.128	1.063	4449	4587	3795
H83022	1.023	1.060	1.015	4450	3654	3142
B86042	1.023	1.226	1.199	4451	5704	5447
J82001	1.023	1.135	1.146	4452	4690	4866
M92043	1.022	1.148	1.189	4453	4871	5358
H84031	1.022	1.040	0.983	4454	3388	2742
M92640	1.022	1.038	1.135	4455	3351	4730
P88021	1.022	1.190	1.181	4456	5364	5291
H81067	1.022	0.954	0.917	4457	2338	2018
F84092	1.022	1.013	1.053	4458	3041	3675
L84048	1.022	1.116	1.057	4459	4454	3725
L84017	1.022	1.125	1.077	4460	4570	4006
G85682	1.022	1.109	1.166	4461	4359	5136
G84015	1.022	1.004	0.971	4462	2935	2623
G85097	1.022	1.084	1.060	4463	4004	3771
B82061	1.022	1.206	1.145	4464	5550	4862
C82079	1.022	1.104	1.075	4465	4281	3979
H83626	1.022	1.093	1.155	4466	4144	5007
H81641	1.021	1.010	0.956	4467	3010	2444
M87041	1.021	1.143	1.116	4468	4813	4525
K84038	1.021	1.149	1.106	4469	4888	4395
F84661	1.021	1.067	1.076	4470	3767	4003
M83678	1.021	1.204	1.359	4471	5536	6694
P81042	1.021	1.374	1.419	4472	6851	6950
D83036	1.021	1.121	1.119	4473	4538	4565
F81043	1.021	1.077	1.015	4474	3932	3172
B81644	1.021	1.095	1.212	4475	4183	5625
F86038	1.020	0.962	0.978	4476	2443	2697
E84062	1.020	1.044	1.024	4477	3457	3299
J83028	1.020	1.137	1.106	4478	4760	4414
G81065	1.020	1.155	1.153	4479	4975	5000
L81072	1.020	1.062	1.017	4480	3722	3204
D81051	1.020	1.132	1.122	4481	4699	4612
F82674	1.020	1.039	1.022	4482	3402	3275
D82045	1.020	1.135	1.113	4483	4739	4513
G82129	1.020	1.040	1.027	4484	3414	3344
M85033	1.020	1.092	1.062	4485	4153	3824
F81089	1.019	1.091	1.042	4486	4140	3551
G84011	1.019	1.005	0.984	4487	2981	2782
L85014	1.019	1.098	1.014	4488	4236	3182
C84133	1.019	1.117	1.122	4489	4502	4623
F86658	1.019	1.027	1.030	4490	3244	3400
B83621	1.019	1.077	1.150	4491	3949	4974
M82011	1.019	1.174	1.142	4492	5228	4861
M81074	1.019	1.190	1.131	4493	5404	4721

D83057	1.019	1.060	1.048	4494	3712	3645
L85015	1.019	1.108	1.081	4495	4397	4097
M85068	1.018	1.041	1.010	4496	3440	3126
E84020	1.018	1.130	1.120	4497	4692	4611
F81116	1.018	1.186	1.163	4498	5359	5128
M91643	1.018	1.159	1.198	4499	5040	5489
K84015	1.018	1.128	1.098	4500	4650	4332
G82039	1.018	1.131	1.127	4501	4706	4692
J82029	1.018	1.268	1.232	4502	6156	5827
M83129	1.018	1.159	1.177	4503	5049	5296
A83034	1.018	1.150	1.133	4504	4953	4764
F81613	1.017	1.225	1.199	4505	5746	5510
C81115	1.017	1.365	1.303	4506	6823	6387
H81077	1.017	1.110	1.046	4507	4443	3644
F81149	1.017	1.101	1.078	4508	4313	4075
J82119	1.017	1.082	1.027	4509	4051	3374
B82622	1.017	1.169	1.132	4510	5189	4766
F86627	1.017	1.065	1.060	4511	3794	3840
M84046	1.017	1.044	1.014	4512	3499	3203
L85608	1.017	1.323	1.274	4513	6595	6183
A88025	1.017	1.174	1.132	4514	5249	4754
N83633	1.017	1.265	1.329	4515	6147	6543
E82041	1.017	1.043	1.029	4516	3484	3427
Y00105	1.017	1.065	1.048	4517	3796	3679
E81037	1.017	1.119	1.076	4518	4569	4057
K84020	1.017	1.085	1.057	4519	4098	3794
F85017	1.017	1.029	1.082	4520	3308	4146
C84010	1.016	1.131	1.089	4521	4725	4239
B82018	1.016	1.028	0.996	4522	3293	2968
N84003	1.016	1.098	1.072	4523	4271	4017
G85034	1.016	1.074	1.051	4524	3943	3722
C84049	1.016	1.084	1.070	4525	4082	3981
Y02884	1.016	1.190	1.305	4526	5439	6404
P81028	1.016	1.150	1.269	4527	4960	6147
D83037	1.016	1.109	1.066	4528	4433	3935
K83022	1.016	1.123	1.074	4529	4621	4042
M86001	1.016	1.256	1.312	4530	6066	6466
M88004	1.016	1.219	1.187	4531	5710	5399
E81030	1.016	1.234	1.256	4532	5841	6050
L83031	1.015	1.189	1.161	4533	5436	5142
P81036	1.015	1.204	1.234	4534	5574	5872
C81081	1.015	1.228	1.194	4535	5781	5480
H83014	1.015	1.062	1.032	4536	3779	3469
B83027	1.015	1.163	1.149	4537	5131	5010
E82098	1.015	1.061	1.048	4538	3771	3696
E85001	1.015	1.053	1.001	4539	3664	3053
M81057	1.015	1.163	1.119	4540	5129	4636
A82049	1.015	1.133	1.101	4541	4766	4417
L81019	1.015	1.075	1.044	4542	3985	3649
D81618	1.015	1.117	1.222	4543	4560	5763

M81081	1.015	1.098	1.036	4544	4291	3535
L85003	1.015	1.144	1.109	4545	4914	4522
J83615	1.015	1.142	1.110	4546	4878	4536
M81041	1.015	1.170	1.142	4547	5219	4922
G85123	1.015	1.003	0.995	4548	3009	2982
J82203	1.015	1.103	1.095	4549	4384	4343
E82123	1.015	1.082	1.043	4550	4077	3636
G82071	1.015	1.126	1.106	4551	4675	4490
F81165	1.014	0.970	0.928	4552	2607	2218
G81035	1.014	1.089	1.045	4553	4186	3678
H81087	1.014	1.082	1.031	4554	4093	3482
M86003	1.014	1.060	1.032	4555	3769	3492
K84065	1.014	1.056	1.051	4556	3731	3756
B81051	1.014	1.039	1.019	4557	3475	3314
G81099	1.014	1.152	1.111	4558	5002	4561
M81064	1.014	1.099	1.027	4559	4331	3439
L85031	1.013	1.149	1.140	4560	4986	4909
B82044	1.013	1.024	1.005	4561	3281	3129
L83637	1.013	1.352	1.470	4562	6763	7117
G82025	1.013	1.076	1.061	4563	4019	3917
B83016	1.013	1.275	1.285	4564	6275	6297
B85021	1.013	1.250	1.235	4565	6031	5902
H82059	1.012	1.155	1.119	4566	5057	4655
D82019	1.012	1.254	1.227	4567	6084	5834
F85032	1.012	0.991	0.950	4568	2892	2479
M84612	1.012	1.151	1.096	4569	5009	4380
P91631	1.012	1.039	1.072	4570	3498	4066
E82025	1.012	1.060	1.011	4571	3799	3233
M84002	1.012	1.098	1.075	4572	4343	4103
F82614	1.012	1.050	1.027	4573	3661	3456
G85119	1.012	1.095	1.073	4574	4292	4071
D83013	1.012	1.158	1.118	4575	5099	4654
G83009	1.012	1.046	1.010	4576	3603	3226
M82045	1.012	1.245	1.233	4577	5989	5894
M89026	1.012	1.104	1.023	4578	4440	3416
N85025	1.011	1.124	1.136	4579	4705	4884
B87029	1.011	1.222	1.230	4580	5756	5867
L85038	1.011	1.125	1.057	4581	4710	3885
P84652	1.011	1.060	1.161	4582	3812	5177
G82230	1.011	1.046	1.039	4583	3613	3624
M84041	1.011	1.095	1.088	4584	4307	4298
M82032	1.011	1.115	1.096	4585	4579	4406
G84035	1.011	1.040	1.040	4586	3526	3643
G82120	1.011	1.023	0.990	4587	3306	2957
E82077	1.011	1.014	0.999	4588	3200	3084
G84020	1.011	1.077	1.040	4589	4056	3638
P81092	1.011	1.326	1.323	4590	6651	6546
A82014	1.011	1.129	1.110	4591	4776	4583
H81036	1.010	0.986	0.956	4592	2844	2561
H84040	1.010	1.025	0.979	4593	3334	2824

G85026	1.010	1.019	1.062	4594	3255	3963
P88008	1.010	1.162	1.154	4595	5180	5126
C82062	1.010	1.046	1.034	4596	3632	3564
L83007	1.010	1.185	1.109	4597	5456	4580
B82006	1.010	1.107	1.065	4598	4493	3998
J83004	1.010	1.066	1.044	4599	3914	3715
P86624	1.010	1.259	1.363	4600	6153	6774
P82607	1.010	1.248	1.269	4601	6059	6195
C82109	1.010	1.074	1.033	4602	4044	3557
N85002	1.010	1.098	1.062	4603	4381	3967
E82626	1.009	1.069	1.037	4604	3974	3614
D81030	1.009	1.174	1.147	4605	5324	5057
N84011	1.009	1.222	1.178	4606	5785	5383
E81064	1.009	1.020	1.045	4607	3277	3730
K81101	1.009	1.060	1.042	4608	3834	3701
C86613	1.009	1.236	1.281	4609	5934	6298
G81104	1.009	1.152	1.138	4610	5069	4943
M86007	1.009	1.154	1.164	4611	5087	5243
H83015	1.009	1.025	0.994	4612	3372	3041
F81085	1.009	1.069	0.981	4613	3969	2874
M88026	1.009	1.212	1.269	4614	5707	6203
C82627	1.009	1.140	1.139	4615	4948	4967
J81616	1.008	1.048	1.007	4616	3687	3225
F82607	1.008	1.046	1.030	4617	3655	3542
B82025	1.008	1.135	1.138	4618	4874	4953
K82079	1.008	1.130	1.085	4619	4816	4296
G82631	1.008	1.047	1.082	4620	3683	4253
B85026	1.008	1.065	1.056	4621	3936	3908
G84032	1.008	1.086	1.050	4622	4226	3822
M85042	1.008	1.032	1.050	4623	3453	3826
P81079	1.008	1.097	1.068	4624	4386	4064
B82029	1.008	1.088	1.050	4625	4246	3823
F86032	1.008	1.019	0.974	4626	3292	2803
E83038	1.008	1.114	1.036	4627	4615	3619
E85015	1.008	1.043	1.061	4628	3621	3986
K82074	1.007	1.048	1.026	4629	3705	3501
K82073	1.007	1.092	1.040	4630	4318	3692
P81642	1.007	1.098	1.095	4631	4403	4438
J83054	1.007	1.131	1.151	4632	4845	5118
C84055	1.007	1.129	1.084	4633	4823	4303
C84017	1.007	1.100	1.050	4634	4445	3855
E85044	1.007	0.970	0.968	4635	2700	2745
C86017	1.007	1.158	1.164	4636	5169	5271
K82031	1.007	1.091	1.045	4637	4319	3775
M83088	1.007	1.146	1.112	4638	5021	4645
C84113	1.007	1.179	1.105	4639	5423	4569
K81055	1.006	1.013	0.975	4640	3226	2832
F81158	1.006	1.228	1.230	4641	5877	5920
P81726	1.006	1.227	1.378	4642	5863	6852
M82035	1.006	1.078	1.057	4643	4155	3954

A82070	1.006	1.055	1.008	4644	3822	3277
B82063	1.006	1.199	1.202	4645	5620	5666
C81640	1.006	1.042	1.002	4646	3631	3199
A88022	1.006	1.232	1.183	4647	5918	5456
N82026	1.006	1.107	1.160	4648	4554	5241
J81067	1.005	1.157	1.177	4649	5188	5409
J82212	1.005	1.036	1.067	4650	3564	4091
B87017	1.005	1.147	1.143	4651	5054	5063
F81218	1.004	1.032	1.043	4652	3508	3772
G83651	1.004	1.055	1.101	4653	3846	4555
F86692	1.004	1.066	1.091	4654	4003	4431
M84067	1.004	1.106	1.070	4655	4561	4149
M81066	1.004	1.111	1.069	4656	4625	4122
C81100	1.004	1.197	1.147	4657	5611	5111
K82631	1.004	0.991	0.973	4658	2995	2838
H83625	1.004	1.002	1.014	4659	3131	3395
B85022	1.004	1.112	1.096	4660	4636	4497
J81086	1.004	1.083	1.115	4661	4241	4716
C84654	1.004	1.105	1.143	4662	4556	5076
M82616	1.004	1.228	1.262	4663	5896	6190
H82091	1.004	1.167	1.131	4664	5315	4918
B82038	1.004	1.184	1.197	4665	5512	5634
K81012	1.003	1.044	1.040	4666	3703	3735
B82007	1.003	1.191	1.130	4667	5572	4906
B83012	1.003	1.129	1.211	4668	4866	5764
L81077	1.003	1.110	1.146	4669	4612	5106
E84684	1.003	1.062	1.091	4670	3956	4442
C88034	1.003	1.029	1.046	4671	3481	3831
E85075	1.003	1.091	1.143	4672	4368	5081
N82073	1.003	1.087	1.098	4673	4308	4531
J83025	1.003	1.102	1.124	4674	4521	4842
H84637	1.003	1.063	1.066	4675	3983	4095
P88031	1.003	1.266	1.239	4676	6280	6028
J82207	1.003	1.020	1.037	4677	3381	3713
L81016	1.003	1.146	1.102	4678	5066	4577
A88013	1.003	1.245	1.213	4679	6086	5787
C84107	1.003	1.067	1.034	4680	4042	3685
J81061	1.003	1.133	1.083	4681	4932	4349
E84056	1.002	1.056	1.074	4682	3876	4226
M81042	1.002	1.103	1.034	4683	4535	3683
K83019	1.002	1.089	1.061	4684	4346	4050
P92005	1.002	1.193	1.231	4685	5595	5971
D83021	1.002	1.130	1.086	4686	4893	4381
E86605	1.002	1.078	1.086	4687	4197	4384
C82119	1.002	1.026	1.071	4688	3454	4198
M84616	1.002	1.161	1.143	4689	5259	5090
F81640	1.002	1.176	1.274	4690	5446	6300
P81655	1.002	1.043	1.046	4691	3714	3862
N84020	1.002	1.087	1.066	4692	4332	4113
J82051	1.002	1.064	1.019	4693	4006	3481

D81630	1.002	0.983	0.989	4694	2932	3070
P81754	1.001	1.228	1.329	4695	5921	6636
B81020	1.001	1.145	1.203	4696	5076	5711
M85732	1.001	1.335	1.387	4697	6738	6905
P81069	1.001	1.118	1.076	4698	4759	4260
J82625	1.001	1.060	1.042	4699	3960	3801
J82035	1.001	1.015	0.964	4700	3326	2767
G82687	1.001	1.206	1.183	4701	5716	5516
E82081	1.001	0.999	0.984	4702	3133	3020
H83627	1.001	0.994	0.977	4703	3068	2923
H83631	1.001	1.017	1.012	4704	3362	3401
L81046	1.001	1.074	1.051	4705	4171	3937
J82188	1.001	1.047	1.047	4706	3776	3888
J82016	1.001	1.060	1.036	4707	3970	3719
H84043	1.001	0.966	0.921	4708	2735	2292
A89002	1.001	1.248	1.303	4709	6129	6504
F84031	1.000	1.160	1.112	4710	5273	4720
C84048	1.000	1.119	1.086	4711	4773	4412
J83038	1.000	1.236	1.189	4712	6016	5599
P81055	1.000	1.168	1.114	4713	5366	4748
C81090	1.000	1.265	1.216	4714	6297	5846
J82220	1.000	0.980	0.950	4715	2899	2632
B81665	1.000	1.099	1.201	4716	4515	5710
H83004	1.000	1.028	1.003	4717	3514	3296
D81637	1.000	0.981	0.979	4718	2928	2966
A86029	1.000	1.194	1.209	4719	5628	5774
C88015	1.000	1.189	1.182	4720	5590	5521
G84010	1.000	1.038	1.007	4721	3679	3343
M87002	0.999	1.127	1.110	4722	4890	4705
M89017	0.999	1.093	1.010	4723	4456	3406
M88020	0.999	1.054	1.026	4724	3902	3608
C84691	0.999	0.973	1.008	4725	2823	3366
L83086	0.999	1.160	1.147	4726	5292	5159
L85054	0.999	1.133	1.093	4727	4966	4518
B81041	0.999	1.125	1.071	4728	4877	4238
D82073	0.999	1.136	1.125	4729	4998	4911
F85681	0.998	0.943	0.944	4730	2472	2572
N81051	0.998	1.173	1.114	4731	5452	4763
E85708	0.998	0.992	0.986	4732	3076	3085
N81077	0.998	1.100	1.062	4733	4555	4112
E85088	0.998	1.147	1.198	4734	5139	5697
N81026	0.998	1.085	1.051	4735	4350	3974
C82025	0.998	1.159	1.098	4736	5285	4595
Y03222	0.998	1.350	1.291	4737	6844	6436
F82661	0.998	0.975	1.040	4738	2869	3833
E84646	0.998	0.917	0.895	4739	2196	2038
P84023	0.998	1.024	1.074	4740	3495	4287
H82067	0.997	1.182	1.178	4741	5547	5494
P85021	0.997	1.172	1.209	4742	5450	5801
P81104	0.997	1.289	1.337	4743	6495	6700

M83059	0.997	1.036	0.980	4744	3678	3016
C86623	0.997	1.394	1.521	4745	7010	7216
M83102	0.997	1.173	1.269	4746	5466	6291
M82040	0.997	1.053	1.044	4747	3915	3894
C81021	0.997	1.176	1.110	4748	5496	4736
P81730	0.997	1.148	1.208	4749	5156	5791
E85098	0.997	0.987	1.052	4750	3037	4002
K84055	0.997	1.087	1.061	4751	4399	4125
C84120	0.997	1.097	1.081	4752	4530	4388
H82056	0.997	1.016	0.999	4753	3407	3278
C81099	0.997	1.155	1.098	4754	5256	4601
J82056	0.997	1.148	1.100	4755	5172	4621
J82174	0.997	1.041	1.012	4756	3759	3458
M85113	0.997	1.114	1.154	4757	4757	5276
M88624	0.997	1.117	1.202	4758	4797	5743
N84007	0.996	1.233	1.290	4759	6025	6443
F81690	0.996	1.247	1.226	4760	6167	5979
B86008	0.996	1.026	1.015	4761	3538	3504
E85745	0.996	1.045	1.087	4762	3802	4474
P81065	0.996	1.137	1.161	4763	5043	5343
B85002	0.996	1.115	1.091	4764	4779	4524
N83055	0.996	1.256	1.319	4765	6253	6606
B81084	0.996	1.093	1.068	4766	4492	4227
C81654	0.996	1.123	1.152	4767	4881	5262
L83041	0.996	1.080	1.037	4768	4321	3811
B82050	0.996	1.132	1.104	4769	4987	4678
M83639	0.996	1.228	1.262	4770	5976	6250
M81008	0.996	1.120	1.082	4771	4848	4426
H85686	0.996	0.974	0.953	4772	2876	2694
G85024	0.996	1.249	1.236	4773	6194	6047
G82031	0.996	1.044	1.016	4774	3808	3523
A82003	0.996	1.231	1.148	4775	6004	5216
J81020	0.996	1.149	1.079	4776	5202	4375
C86012	0.995	1.119	1.111	4777	4850	4774
C86037	0.995	1.204	1.177	4778	5752	5507
L85612	0.995	1.158	1.153	4779	5311	5282
A83005	0.995	1.233	1.173	4780	6053	5481
G82133	0.995	1.078	1.058	4781	4301	4100
K84031	0.995	1.111	1.059	4782	4750	4124
C82123	0.995	1.049	1.023	4783	3887	3633
H85035	0.995	1.072	1.019	4784	4209	3563
A82021	0.995	1.131	1.124	4785	4990	4942
G81031	0.994	1.091	1.109	4786	4481	4758
J84005	0.994	1.077	1.052	4787	4285	4039
M84040	0.994	1.086	1.061	4788	4427	4160
F81057	0.994	1.026	1.001	4789	3579	3330
H85007	0.994	1.001	0.952	4790	3236	2719
E81029	0.994	1.139	1.114	4791	5092	4819
G84629	0.994	0.986	0.982	4792	3056	3083
M81084	0.994	1.044	0.991	4793	3831	3206

C81118	0.993	1.123	1.120	4794	4917	4903
Y00252	0.993	1.220	1.286	4795	5930	6432
J81053	0.993	1.161	1.120	4796	5355	4902
M85098	0.993	1.009	1.060	4797	3363	4164
A88601	0.993	1.444	1.453	4798	7156	7127
G85036	0.993	1.084	1.063	4799	4415	4205
F81007	0.993	1.163	1.116	4800	5390	4858
J84020	0.993	1.130	1.115	4801	4994	4846
H83016	0.993	1.035	1.003	4802	3726	3376
E84017	0.993	0.990	0.921	4803	3127	2371
F81101	0.993	1.017	0.993	4804	3452	3248
L83085	0.993	1.107	1.088	4805	4720	4535
M91637	0.993	1.207	1.174	4806	5793	5515
C81083	0.993	1.094	1.041	4807	4548	3911
M82606	0.993	1.081	1.105	4808	4389	4722
A82013	0.992	1.112	1.059	4809	4790	4163
M87612	0.992	1.060	1.123	4810	4081	4969
F81153	0.992	1.137	1.126	4811	5089	5012
M85097	0.992	1.089	1.143	4812	4490	5201
C82056	0.992	1.031	0.993	4813	3677	3258
H85674	0.992	0.991	0.979	4814	3145	3075
P89021	0.992	1.236	1.278	4815	6104	6389
C84621	0.992	1.043	1.023	4816	3850	3681
L83099	0.992	1.228	1.186	4817	6012	5644
P87661	0.992	1.201	1.317	4818	5753	6618
M88010	0.991	1.186	1.158	4819	5632	5356
B87600	0.991	1.249	1.279	4820	6239	6402
J82146	0.991	1.060	1.016	4821	4095	3580
A82025	0.991	1.107	1.056	4822	4743	4138
P81125	0.991	1.153	1.155	4823	5298	5335
G84021	0.991	1.024	1.003	4824	3594	3412
G82067	0.991	1.038	1.009	4825	3782	3490
D83030	0.991	1.148	1.114	4826	5241	4857
G82658	0.991	1.069	1.073	4827	4232	4369
Y02900	0.991	0.936	0.926	4828	2473	2445
P81778	0.991	1.325	1.423	4829	6741	7055
F86036	0.991	0.985	0.980	4830	3088	3099
C81114	0.991	1.112	1.067	4831	4812	4299
L83098	0.990	1.105	1.081	4832	4719	4475
K82610	0.990	1.118	1.084	4833	4885	4509
H85662	0.990	0.943	0.925	4834	2565	2438
E83035	0.990	1.019	0.986	4835	3525	3186
B86013	0.990	1.057	1.063	4836	4076	4240
E84658	0.990	0.978	0.901	4837	3004	2193
J81064	0.990	1.172	1.171	4838	5526	5498
A83610	0.990	1.277	1.369	4839	6464	6878
E82071	0.990	1.009	0.995	4840	3406	3311
L81014	0.990	1.086	1.061	4841	4478	4216
F81749	0.990	1.088	1.031	4842	4508	3819
F84620	0.990	1.028	1.100	4843	3675	4703

D82025	0.989	1.200	1.123	4844	5763	5001
P84665	0.989	1.095	1.174	4845	4594	5533
Y03587	0.989	1.044	1.068	4846	3899	4324
J82103	0.989	1.140	1.085	4847	5150	4541
F81109	0.989	1.047	1.051	4848	3948	4086
M85689	0.989	1.061	1.142	4849	4148	5220
P92038	0.989	1.068	1.093	4850	4238	4626
K82004	0.989	1.044	1.005	4851	3908	3466
M85133	0.989	1.193	1.276	4852	5714	6395
J81040	0.989	1.235	1.178	4853	6123	5604
M82006	0.989	1.209	1.174	4854	5856	5544
C84037	0.988	1.080	1.040	4855	4418	3960
M83138	0.988	1.079	1.201	4856	4408	5812
C84693	0.988	1.132	1.246	4857	5080	6185
P92033	0.988	1.145	1.182	4858	5240	5642
J82093	0.988	1.269	1.250	4859	6424	6223
E85003	0.987	0.988	1.034	4860	3172	3892
N85014	0.987	1.042	1.053	4861	3892	4153
L83067	0.987	1.144	1.076	4862	5239	4456
C85007	0.987	1.243	1.213	4863	6212	5944
F82013	0.987	0.988	0.959	4864	3181	2872
M85792	0.987	1.142	1.173	4865	5225	5562
L84084	0.987	1.149	1.081	4866	5308	4510
G83630	0.987	1.038	0.993	4867	3844	3325
E86615	0.987	1.046	1.083	4868	3967	4539
P87035	0.987	1.147	1.165	4869	5284	5470
A83641	0.987	1.214	1.172	4870	5932	5554
K84017	0.987	1.083	1.047	4871	4489	4067
K83048	0.986	1.199	1.188	4872	5779	5713
J82114	0.986	1.272	1.242	4873	6455	6166
N85041	0.986	1.114	1.108	4874	4889	4845
H81032	0.986	1.045	1.029	4875	3965	3851
G82106	0.986	1.088	1.091	4876	4566	4639
L84038	0.986	1.080	1.044	4877	4453	4046
M81027	0.986	1.046	1.008	4878	3980	3544
H81052	0.986	1.061	1.036	4879	4200	3939
C81113	0.986	1.096	1.138	4880	4666	5211
N82052	0.986	1.250	1.335	4881	6294	6756
E84685	0.985	0.960	0.932	4882	2831	2583
H85006	0.985	1.098	1.040	4883	4703	3999
Y00351	0.985	1.113	1.102	4884	4900	4782
K84037	0.985	1.091	1.045	4885	4605	4060
P84017	0.985	1.172	1.224	4886	5565	6040
H84623	0.985	1.023	0.991	4887	3670	3324
L85024	0.985	1.190	1.149	4888	5721	5347
J81021	0.985	1.108	1.084	4889	4842	4578
B82609	0.985	1.102	1.128	4890	4765	5120
C81058	0.984	1.273	1.213	4891	6472	5963
L81669	0.984	1.113	1.118	4892	4903	5008
F81730	0.984	1.101	1.065	4893	4758	4346

E85040	0.984	0.999	0.974	4894	3343	3107
F84698	0.984	1.031	0.993	4895	3781	3359
D81045	0.984	1.136	1.111	4896	5185	4908
F86011	0.984	1.027	1.088	4897	3744	4632
D81606	0.984	1.032	1.021	4898	3789	3760
L82059	0.983	1.184	1.173	4899	5675	5602
B82019	0.983	1.043	1.013	4900	3966	3646
B82067	0.983	1.097	1.075	4901	4712	4482
N83608	0.983	1.238	1.261	4902	6210	6333
F86022	0.983	1.090	1.054	4903	4618	4215
L81028	0.983	1.145	1.107	4904	5306	4865
H81107	0.983	0.985	0.958	4905	3190	2910
M83092	0.983	1.056	1.007	4906	4159	3567
J81032	0.983	1.199	1.139	4907	5807	5261
F84711	0.983	1.052	1.101	4908	4111	4798
H82007	0.982	1.147	1.103	4909	5331	4828
H81047	0.982	1.010	0.951	4910	3511	2830
E84706	0.982	1.133	1.158	4911	5159	5445
F81076	0.982	1.033	0.993	4912	3841	3392
D82100	0.982	1.193	1.132	4913	5766	5184
E84061	0.982	1.114	1.066	4914	4949	4389
M83738	0.982	1.150	1.143	4915	5371	5313
D83004	0.982	1.062	1.056	4916	4253	4257
M82047	0.981	1.154	1.148	4917	5424	5361
B86036	0.981	1.152	1.142	4918	5405	5309
H81089	0.981	1.079	1.063	4919	4498	4357
B86017	0.981	1.043	1.048	4920	3995	4172
F81079	0.981	1.132	1.095	4921	5165	4741
P88024	0.981	1.064	1.043	4922	4298	4088
G83013	0.981	1.088	1.094	4923	4632	4727
C88052	0.981	1.083	1.117	4924	4564	5031
H83030	0.981	1.021	1.058	4925	3697	4301
G84607	0.980	1.098	1.105	4926	4764	4886
J82197	0.980	0.998	0.980	4927	3384	3238
C81013	0.980	1.114	1.099	4928	4967	4805
E81034	0.980	1.122	1.095	4929	5048	4747
F82649	0.980	1.102	1.073	4930	4826	4508
F85697	0.980	0.992	1.056	4931	3311	4285
H82078	0.980	1.104	1.101	4932	4855	4835
L81120	0.980	1.127	1.111	4933	5122	4977
G85029	0.980	0.991	0.999	4934	3286	3513
H82030	0.979	1.057	1.005	4935	4219	3591
L81123	0.979	1.107	1.071	4936	4887	4489
M83617	0.979	1.160	1.136	4937	5517	5273
K84072	0.979	1.129	1.082	4938	5142	4617
Y01177	0.979	0.960	0.995	4939	2922	3464
H81051	0.979	1.049	1.001	4940	4125	3548
P88025	0.979	1.053	1.028	4941	4179	3926
J83003	0.979	1.143	1.093	4942	5322	4745
G82212	0.978	1.024	1.007	4943	3765	3635

J81631	0.978	1.120	1.059	4944	5046	4348
G81003	0.978	1.225	1.221	4945	6130	6074
A81023	0.978	1.136	1.172	4946	5254	5639
L83118	0.978	1.254	1.297	4947	6377	6580
P92651	0.978	1.154	1.256	4948	5471	6336
E82063	0.978	1.058	1.038	4949	4254	4062
M89007	0.978	1.131	1.107	4950	5199	4948
D82099	0.978	1.067	1.044	4951	4394	4157
E82099	0.977	1.088	1.063	4952	4672	4404
K82035	0.977	1.030	0.982	4953	3870	3312
F81023	0.977	1.033	0.959	4954	3920	3001
C81642	0.977	1.084	1.073	4955	4623	4545
F84702	0.977	0.964	0.932	4956	2993	2667
J83643	0.977	1.082	1.074	4957	4593	4552
J82126	0.977	1.075	1.105	4958	4495	4919
G82027	0.977	1.102	1.083	4959	4864	4657
J84003	0.977	1.140	1.098	4960	5313	4841
P81165	0.977	1.265	1.355	4961	6473	6874
C81053	0.977	1.167	1.108	4962	5587	4980
A89015	0.977	1.185	1.183	4963	5744	5752
J82181	0.977	1.082	1.029	4964	4585	3965
J82023	0.977	0.985	0.968	4965	3257	3124
M81067	0.977	1.015	0.970	4966	3671	3157
N81115	0.977	1.111	1.071	4967	4965	4516
F86639	0.977	1.012	1.097	4968	3626	4827
M85028	0.976	1.069	1.041	4969	4428	4132
K84043	0.976	1.091	1.029	4970	4728	3977
L84007	0.976	1.039	1.027	4971	4010	3940
G82013	0.976	1.097	1.068	4972	4803	4487
F81716	0.976	1.066	1.072	4973	4402	4542
C82122	0.976	1.136	1.160	4974	5269	5532
P88041	0.976	1.019	1.007	4975	3739	3669
M86040	0.976	1.133	1.140	4976	5238	5336
B86030	0.976	1.018	1.073	4977	3728	4551
Y00280	0.976	0.947	0.968	4978	2789	3131
K82047	0.976	1.106	1.059	4979	4920	4373
P81136	0.976	1.288	1.295	4980	6629	6585
C85023	0.976	1.315	1.318	4981	6770	6719
K81022	0.975	1.045	1.005	4982	4113	3647
L84021	0.975	1.169	1.116	4983	5623	5086
E82059	0.975	1.046	1.021	4984	4129	3882
J81057	0.975	1.112	1.065	4985	4989	4460
G82793	0.975	1.257	1.246	4986	6425	6284
C84076	0.975	1.149	1.136	4987	5445	5307
E85113	0.975	1.016	1.016	4988	3708	3808
C87030	0.975	1.205	1.168	4989	5951	5641
M82055	0.975	1.138	1.100	4990	5314	4896
L81004	0.975	1.059	1.009	4991	4317	3711
L82048	0.975	1.084	1.059	4992	4653	4386
E82022	0.975	1.083	1.045	4993	4648	4213

G83005	0.974	1.191	1.129	4994	5813	5245
M85177	0.974	1.228	1.164	4995	6201	5613
G82108	0.974	1.243	1.268	4996	6320	6444
J82190	0.974	1.059	1.030	4997	4329	4024
D82056	0.974	1.170	1.124	4998	5640	5188
N82645	0.974	1.191	1.269	4999	5820	6457
P89020	0.974	1.187	1.200	5000	5790	5946
E84620	0.974	1.040	1.074	5001	4064	4594
A87020	0.974	1.062	1.064	5002	4385	4472
L85624	0.973	1.162	1.124	5003	5577	5200
C81037	0.973	1.075	1.044	5004	4553	4221
M88035	0.973	1.210	1.280	5005	6023	6524
D83016	0.973	1.174	1.124	5006	5678	5198
F81195	0.972	1.114	1.108	5007	5055	5024
C82002	0.972	1.111	1.083	5008	5019	4713
K82057	0.972	1.086	1.072	5009	4718	4593
L82620	0.972	1.290	1.295	5010	6660	6603
P81191	0.972	1.172	1.140	5011	5664	5374
F85687	0.972	1.048	1.057	5012	4201	4415
K84045	0.972	1.079	1.047	5013	4624	4269
C84029	0.972	1.092	1.047	5014	4795	4267
F85055	0.972	1.026	0.999	5015	3889	3621
H81037	0.972	1.062	1.012	5016	4411	3809
K82633	0.971	0.970	0.958	5017	3147	3064
P81113	0.971	1.103	1.047	5018	4950	4274
B83661	0.971	1.207	1.284	5019	6017	6548
P84050	0.971	1.284	1.289	5020	6636	6579
M86012	0.971	1.129	1.190	5021	5252	5871
E85672	0.970	1.022	1.108	5022	3857	5053
H81035	0.970	1.051	1.010	5023	4257	3782
C81036	0.970	1.123	1.130	5024	5196	5292
J83022	0.970	1.033	1.045	5025	4023	4266
L83025	0.970	1.179	1.159	5026	5751	5589
L84018	0.970	1.090	1.046	5027	4792	4280
G85623	0.970	0.989	0.971	5028	3408	3262
L83129	0.970	1.247	1.204	5029	6402	6010
C86023	0.970	1.206	1.211	5030	6014	6062
N81081	0.970	1.058	1.017	5031	4380	3900
E85111	0.969	0.992	1.010	5032	3444	3804
K84041	0.969	1.045	0.991	5033	4199	3534
P81210	0.969	1.196	1.132	5034	5931	5327
L84613	0.969	1.172	1.155	5035	5696	5557
N82066	0.969	1.136	1.144	5036	5351	5440
C87012	0.969	1.212	1.194	5037	6097	5939
J81036	0.969	1.197	1.172	5038	5935	5723
J81621	0.969	1.169	1.177	5039	5669	5767
E87017	0.969	0.982	1.020	5040	3317	3947
M85143	0.969	1.201	1.159	5041	5971	5610
H83040	0.969	0.958	0.963	5042	3025	3169
L82009	0.969	1.143	1.133	5043	5443	5337

E85721	0.969	1.078	1.149	5044	4654	5496
E82044	0.969	1.041	1.017	5045	4147	3918
H85033	0.968	1.048	0.986	5046	4237	3495
L83058	0.968	1.146	1.092	5047	5489	4864
L85052	0.968	1.141	1.066	5048	5419	4556
B87001	0.968	1.120	1.118	5049	5174	5185
E87026	0.968	1.508	1.488	5050	7269	7214
E81050	0.968	1.080	1.053	5051	4697	4399
F82003	0.968	1.157	1.238	5052	5581	6278
G82690	0.968	1.055	1.044	5053	4360	4279
D82048	0.968	1.097	1.104	5054	4912	5040
B81016	0.968	1.065	1.075	5055	4491	4671
E84057	0.968	1.022	0.959	5056	3883	3134
M85175	0.968	0.987	0.970	5057	3405	3282
C81065	0.967	1.203	1.142	5058	6009	5434
J83603	0.967	1.049	1.028	5059	4270	4065
M85124	0.967	1.036	1.032	5060	4097	4128
C84059	0.967	1.232	1.195	5061	6303	5957
B86642	0.967	1.185	1.261	5062	5832	6450
D82621	0.967	1.299	1.231	5063	6726	6237
A81015	0.967	1.223	1.237	5064	6214	6281
G82760	0.967	1.199	1.239	5065	5980	6294
F85016	0.967	0.951	0.913	5066	2963	2569
H81079	0.967	1.075	1.005	5067	4639	3769
B82080	0.967	1.104	1.075	5068	4999	4683
D81016	0.967	1.118	1.055	5069	5164	4450
K84004	0.967	1.088	1.043	5070	4815	4297
M82038	0.966	1.093	1.071	5071	4876	4641
D82067	0.966	1.273	1.253	5072	6601	6400
A87612	0.966	1.186	1.179	5073	5849	5821
M81040	0.966	1.157	1.097	5074	5594	4973
E87052	0.966	1.013	1.031	5075	3784	4127
G82730	0.966	1.074	1.048	5076	4643	4364
M89015	0.966	1.052	0.997	5077	4338	3673
M81002	0.966	1.069	1.058	5078	4580	4498
C81061	0.965	1.171	1.117	5079	5724	5207
M83006	0.965	1.018	0.991	5080	3872	3592
L83639	0.965	1.022	1.004	5081	3930	3781
F81115	0.965	1.080	1.055	5082	4721	4468
K84001	0.965	0.987	0.944	5083	3436	2967
E86011	0.965	1.103	1.063	5084	5004	4559
Y00268	0.965	0.973	0.932	5085	3251	2807
H85003	0.965	1.021	1.007	5086	3919	3832
C83020	0.965	1.115	1.079	5087	5143	4746
C84086	0.965	1.038	1.003	5088	4168	3770
D82001	0.965	1.120	1.075	5089	5226	4700
J83625	0.965	1.057	1.040	5090	4434	4264
M84014	0.964	1.023	1.014	5091	3959	3930
G84027	0.964	1.002	0.981	5092	3650	3475
L81101	0.964	1.134	1.101	5093	5391	5050

K82007	0.964	1.037	0.995	5094	4163	3668
F85030	0.964	1.064	1.064	5095	4529	4589
E86006	0.964	1.037	1.005	5096	4162	3813
M85062	0.964	1.126	1.136	5097	5307	5414
N81112	0.964	1.046	1.000	5098	4288	3738
C84020	0.964	1.123	1.182	5099	5268	5862
K84035	0.964	1.075	1.036	5100	4680	4236
A82046	0.964	1.094	1.039	5101	4927	4275
M84023	0.964	1.035	1.001	5102	4141	3747
M81080	0.964	1.133	1.099	5103	5383	5032
H83009	0.963	1.003	0.958	5104	3696	3177
P88012	0.963	1.165	1.130	5105	5681	5359
M83665	0.963	1.113	1.148	5106	5144	5545
J82025	0.963	1.062	1.032	5107	4524	4191
E82054	0.963	1.021	0.973	5108	3951	3382
G81086	0.963	1.060	1.021	5109	4499	4041
M85037	0.963	1.108	1.084	5110	5094	4834
C83067	0.963	1.159	1.088	5111	5642	4894
L85025	0.963	1.078	1.049	5112	4736	4420
H84033	0.963	1.014	0.971	5113	3855	3355
M83067	0.963	1.103	1.098	5114	5035	5030
G81642	0.962	1.097	1.107	5115	4977	5127
A89024	0.962	1.257	1.279	5116	6515	6571
H84618	0.962	1.077	1.063	5117	4734	4597
C82678	0.962	1.128	1.176	5118	5344	5826
G81032	0.962	1.067	1.063	5119	4590	4599
D81038	0.962	1.087	1.071	5120	4861	4694
K82617	0.962	1.117	1.090	5121	5220	4921
L83055	0.962	1.229	1.179	5122	6309	5856
L85047	0.962	1.087	1.047	5123	4857	4410
B84007	0.962	1.063	1.084	5124	4549	4847
J82091	0.962	1.088	1.086	5125	4879	4874
P92028	0.961	1.286	1.285	5126	6694	6609
M83078	0.961	1.056	1.016	5127	4466	3995
F84624	0.961	1.090	1.173	5128	4902	5810
C84710	0.961	1.206	1.260	5129	6110	6481
H84007	0.961	0.995	0.972	5130	3602	3396
D83011	0.961	1.162	1.134	5131	5679	5420
C88078	0.961	1.140	1.113	5132	5494	5202
F86635	0.961	1.073	1.081	5133	4689	4823
G81049	0.961	1.174	1.118	5134	5791	5269
J82082	0.961	1.120	1.083	5135	5265	4851
G84019	0.961	1.224	1.166	5136	6287	5746
J83058	0.961	1.036	1.013	5137	4198	3966
K84014	0.961	1.055	1.011	5138	4461	3949
M85029	0.960	1.154	1.124	5139	5618	5332
E82091	0.960	1.088	1.051	5140	4884	4479
F82051	0.960	1.082	1.098	5141	4819	5059
P81015	0.960	1.154	1.125	5142	5631	5345
G85690	0.960	1.002	1.082	5143	3729	4849

M82022	0.960	1.150	1.113	5144	5591	5224
M85052	0.960	1.134	1.128	5145	5449	5379
B81082	0.959	1.047	1.035	5146	4376	4272
J82640	0.959	1.103	1.074	5147	5072	4757
F85688	0.959	0.994	1.024	5148	3610	4130
N83628	0.959	1.225	1.334	5149	6307	6864
M81013	0.959	1.185	1.118	5150	5906	5290
B83041	0.959	1.166	1.170	5151	5734	5795
D82613	0.959	1.231	1.222	5152	6345	6228
H83029	0.959	1.000	0.963	5153	3710	3308
F84111	0.959	1.004	1.001	5154	3768	3836
C83634	0.959	1.228	1.213	5155	6323	6158
J83632	0.959	1.106	1.091	5156	5120	4993
F84666	0.959	1.013	1.058	5157	3894	4590
M85693	0.958	1.047	1.106	5158	4383	5162
C88046	0.958	1.162	1.220	5159	5703	6216
E84617	0.958	0.975	0.932	5160	3382	2892
C81091	0.958	1.080	1.037	5161	4828	4328
P83623	0.958	1.087	1.120	5162	4909	5315
J82092	0.958	1.101	1.097	5163	5060	5066
B83067	0.958	1.202	1.213	5164	6106	6160
N83047	0.958	1.074	1.148	5165	4746	5603
K81102	0.958	1.041	1.035	5166	4294	4311
C82035	0.958	1.100	1.077	5167	5061	4808
J82074	0.958	1.041	1.002	5168	4309	3863
C81054	0.958	1.093	1.075	5169	4982	4783
E83639	0.957	1.077	1.095	5170	4793	5065
B81049	0.957	1.193	1.229	5171	6018	6301
M86035	0.957	1.251	1.328	5172	6510	6850
N81044	0.957	1.145	1.101	5173	5575	5130
M88040	0.957	1.069	1.131	5174	4688	5430
E82661	0.957	1.099	1.062	5175	5065	4643
K83622	0.957	1.096	1.118	5176	5023	5311
P84689	0.957	1.012	1.101	5177	3907	5135
Y00137	0.957	1.109	1.166	5178	5179	5776
J81082	0.957	1.120	1.082	5179	5318	4890
P81010	0.956	1.108	1.073	5180	5176	4778
G82123	0.956	1.025	1.051	5181	4110	4530
A89040	0.956	1.287	1.344	5182	6724	6915
F86641	0.956	1.036	1.105	5183	4243	5171
C84106	0.956	1.151	1.141	5184	5634	5546
H81104	0.956	1.020	0.971	5185	4030	3452
D81036	0.956	1.167	1.134	5186	5765	5473
M85009	0.956	1.052	1.069	5187	4476	4738
P92602	0.956	1.180	1.255	5188	5889	6487
F82001	0.956	1.020	1.087	5189	4036	4985
C88652	0.956	1.039	1.076	5190	4310	4818
F84087	0.956	1.013	0.985	5191	3950	3651
F84022	0.956	1.026	1.019	5192	4127	4102
L81093	0.956	1.112	1.142	5193	5236	5567

E83025	0.955	1.011	0.953	5194	3917	3208
A82028	0.955	1.070	1.062	5195	4735	4667
C84005	0.955	1.090	1.037	5196	4972	4361
B81653	0.955	1.101	1.179	5197	5110	5923
E84021	0.955	0.989	0.936	5198	3601	2996
G82017	0.955	1.073	1.042	5199	4778	4440
L81064	0.955	1.231	1.186	5200	6384	5994
G85651	0.955	0.989	1.015	5201	3614	4070
M91016	0.954	1.102	1.120	5202	5121	5350
F84714	0.954	1.037	1.036	5203	4295	4358
P84678	0.954	1.205	1.295	5204	6162	6708
Y02663	0.954	1.124	1.143	5205	5379	5594
H83005	0.954	1.000	1.021	5206	3773	4159
P92619	0.954	1.064	1.131	5207	4665	5451
K83065	0.954	1.162	1.141	5208	5741	5569
E82066	0.954	0.978	0.983	5209	3458	3634
H81019	0.954	1.097	1.070	5210	5067	4771
L84037	0.954	1.060	1.025	5211	4610	4218
G81082	0.954	1.073	1.017	5212	4775	4107
D82106	0.954	1.073	1.129	5213	4783	5441
C83001	0.954	1.077	1.087	5214	4838	5005
D83028	0.954	1.127	1.118	5215	5429	5333
L81054	0.954	1.244	1.215	5216	6485	6210
L85042	0.953	1.173	1.137	5217	5847	5528
K84006	0.953	1.065	1.021	5218	4685	4179
E87733	0.953	1.158	1.188	5219	5720	6011
B85032	0.953	1.124	1.127	5220	5395	5428
H82084	0.953	1.098	1.068	5221	5088	4752
C84129	0.953	1.128	1.125	5222	5453	5418
H83019	0.953	1.001	1.018	5223	3797	4136
G85012	0.953	1.119	1.102	5224	5343	5169
C82030	0.953	1.078	1.051	5225	4863	4571
L82025	0.953	1.130	1.096	5226	5479	5119
E83024	0.953	1.038	0.980	5227	4328	3613
F85033	0.953	1.013	0.984	5228	3977	3682
J82027	0.953	1.060	1.023	5229	4633	4210
G83060	0.953	1.040	1.019	5230	4370	4156
B86047	0.953	1.062	1.059	5231	4659	4661
E85743	0.952	1.033	1.086	5232	4251	5006
C83637	0.952	1.230	1.190	5233	6400	6033
A89023	0.952	1.196	1.172	5234	6096	5876
E85054	0.952	1.052	1.004	5235	4526	3953
H85041	0.952	1.013	1.007	5236	3981	3997
K82060	0.952	0.993	0.992	5237	3704	3791
C81044	0.952	1.212	1.161	5238	6259	5770
M87036	0.952	1.129	1.201	5239	5476	6123
H84010	0.952	1.002	0.959	5240	3824	3335
K84618	0.952	1.050	1.025	5241	4512	4247
J82061	0.952	1.067	1.013	5242	4731	4081
B81677	0.952	1.134	1.140	5243	5525	5581

A86015	0.952	0.990	1.016	5244	3663	4120
K81070	0.952	1.032	1.001	5245	4245	3932
M86027	0.952	1.075	1.039	5246	4841	4443
A82029	0.951	1.181	1.175	5247	5952	5912
F81698	0.951	1.097	1.112	5248	5093	5302
H82065	0.951	1.220	1.198	5249	6315	6104
F85682	0.951	0.939	0.978	5250	3012	3606
B83022	0.951	1.191	1.193	5251	6069	6066
G84030	0.951	1.102	1.094	5252	5149	5108
L83106	0.951	1.147	1.116	5253	5646	5348
L83647	0.951	1.180	1.127	5254	5942	5450
N85032	0.951	1.128	1.111	5255	5481	5297
F81066	0.951	1.145	1.083	5256	5630	4992
H84635	0.951	0.988	0.940	5257	3652	3094
N81080	0.951	1.117	1.077	5258	5342	4899
P83017	0.951	1.126	1.142	5259	5457	5623
Y03602	0.951	1.087	1.081	5260	4992	4958
J81074	0.951	1.061	1.021	5261	4664	4204
E85127	0.950	1.009	0.980	5262	3955	3655
L83051	0.950	1.134	1.102	5263	5537	5209
P84027	0.950	1.060	1.083	5264	4652	5002
G83044	0.950	1.060	1.039	5265	4655	4453
E82028	0.950	1.042	1.023	5266	4425	4237
M83705	0.950	1.093	1.115	5267	5059	5340
K83053	0.950	1.050	1.029	5268	4528	4331
M81032	0.950	1.123	1.070	5269	5422	4817
F82612	0.950	1.084	1.131	5270	4968	5511
P81736	0.950	1.231	1.386	5271	6426	7065
L84032	0.950	1.190	1.146	5272	6076	5657
L83048	0.950	1.161	1.137	5273	5770	5571
A82036	0.950	1.176	1.108	5274	5923	5285
M91021	0.950	1.307	1.408	5275	6880	7119
E85605	0.950	1.065	1.030	5276	4742	4347
C82037	0.949	1.118	1.060	5277	5373	4708
A83040	0.949	1.188	1.147	5278	6055	5679
D82037	0.949	1.077	1.041	5279	4886	4496
M85103	0.949	1.090	1.066	5280	5042	4785
H84629	0.949	0.977	0.961	5281	3512	3417
C88044	0.949	1.104	1.137	5282	5221	5588
M81023	0.949	1.101	1.062	5283	5181	4733
L84041	0.949	1.090	1.098	5284	5045	5179
C88076	0.949	1.131	1.132	5285	5529	5527
D82016	0.949	1.124	1.091	5286	5458	5107
C81032	0.949	1.204	1.127	5287	6213	5478
L83079	0.948	1.093	1.033	5288	5084	4396
L85041	0.948	1.226	1.175	5289	6398	5947
C81089	0.948	1.334	1.326	5290	6977	6876
N81014	0.948	1.011	0.996	5291	4018	3912
G83019	0.948	1.129	1.178	5292	5515	5984
C83010	0.948	1.018	0.974	5293	4121	3600

A83619	0.947	1.530	1.573	5294	7305	7311
E86024	0.947	1.155	1.156	5295	5737	5773
L84063	0.947	1.053	1.043	5296	4595	4538
C88051	0.947	1.101	1.126	5297	5200	5483
E81005	0.947	1.136	1.116	5298	5578	5377
L83061	0.947	1.121	1.147	5299	5437	5692
K82010	0.947	0.999	0.951	5300	3849	3303
H83001	0.947	1.024	0.987	5301	4210	3786
P81686	0.947	1.131	1.211	5302	5546	6235
J82106	0.947	1.070	1.031	5303	4834	4385
C84072	0.947	1.166	1.149	5304	5842	5716
G85696	0.947	1.004	1.010	5305	3945	4110
C87009	0.947	1.091	1.090	5306	5078	5117
K84610	0.947	1.042	0.998	5307	4472	3952
D83044	0.946	1.312	1.324	5308	6910	6873
M81016	0.946	1.166	1.114	5309	5846	5366
F84720	0.946	1.058	1.115	5310	4693	5375
Y01127	0.946	1.148	1.142	5311	5689	5660
C82662	0.946	0.960	1.021	5312	3328	4262
B86012	0.946	1.184	1.156	5313	6046	5784
F84088	0.946	1.080	1.112	5314	4963	5352
N82033	0.946	1.287	1.272	5315	6783	6627
B83040	0.946	1.067	1.070	5316	4821	4878
M89601	0.946	1.025	0.973	5317	4234	3612
A83072	0.946	1.102	1.100	5318	5234	5230
H84055	0.946	0.947	0.911	5319	3180	2780
K81043	0.946	1.057	1.028	5320	4683	4363
P84062	0.946	1.135	1.203	5321	5589	6184
M88620	0.945	1.090	1.169	5322	5086	5915
E84029	0.945	1.035	1.053	5323	4400	4677
H82049	0.945	1.096	1.078	5324	5148	4998
M86624	0.945	1.116	1.102	5325	5400	5267
D82040	0.945	1.182	1.180	5326	6022	6020
E84645	0.945	0.965	0.981	5327	3416	3737
F82638	0.945	0.993	1.049	5328	3791	4628
P91009	0.945	1.193	1.166	5329	6139	5892
G82143	0.945	1.105	1.093	5330	5279	5167
H85048	0.945	0.978	0.937	5331	3596	3135
L81065	0.945	1.096	1.072	5332	5175	4914
K81004	0.945	1.144	1.135	5333	5670	5619
B86038	0.945	1.090	1.087	5334	5103	5112
L82036	0.944	1.141	1.114	5335	5650	5387
K81018	0.944	1.024	0.999	5336	4250	3993
C81038	0.944	1.108	1.094	5337	5325	5192
B81080	0.944	1.151	1.247	5338	5732	6508
Y00352	0.944	0.997	0.999	5339	3871	4005
J83007	0.944	1.086	1.054	5340	5056	4701
J81011	0.944	1.141	1.056	5341	5651	4723
H82023	0.943	1.250	1.209	5342	6598	6248
C84696	0.943	1.088	1.068	5343	5079	4879

G85134	0.943	0.969	1.015	5344	3472	4234
G81613	0.943	1.060	1.091	5345	4749	5155
E82062	0.943	1.072	1.046	5346	4907	4615
N83026	0.943	1.190	1.253	5347	6133	6533
J81039	0.943	1.196	1.191	5348	6191	6115
F84668	0.943	0.948	0.964	5349	3215	3536
J83031	0.943	1.166	1.155	5350	5888	5811
M83024	0.943	1.081	1.038	5351	5005	4532
M85035	0.942	1.274	1.266	5352	6730	6610
P89026	0.942	1.297	1.328	5353	6863	6908
H81004	0.942	1.044	1.003	5354	4562	4079
E83006	0.942	0.984	0.929	5355	3727	3066
L83143	0.942	1.072	1.019	5356	4935	4291
F81633	0.942	1.071	1.096	5357	4913	5234
K81045	0.942	1.048	1.037	5358	4602	4526
J81647	0.942	1.049	1.018	5359	4634	4293
C81010	0.942	1.114	1.056	5360	5417	4750
H82046	0.942	1.129	1.114	5361	5570	5419
L81106	0.942	1.117	1.103	5362	5464	5318
D82071	0.941	1.107	1.076	5363	5337	5016
J82087	0.941	1.206	1.186	5364	6300	6092
K84042	0.941	1.046	0.998	5365	4586	4033
E81011	0.941	1.088	1.063	5366	5116	4855
P81179	0.941	1.144	1.164	5367	5702	5913
K83068	0.941	1.086	1.029	5368	5090	4446
J81014	0.941	1.080	1.057	5369	5024	4776
G82100	0.940	1.107	1.077	5370	5338	5037
L85055	0.940	1.072	1.056	5371	4945	4770
N83601	0.940	1.432	1.516	5372	7223	7275
H82643	0.940	1.164	1.130	5373	5891	5608
M85736	0.940	1.228	1.293	5374	6469	6766
P81741	0.940	1.211	1.253	5375	6335	6549
B86100	0.940	1.108	1.149	5376	5363	5771
M86034	0.940	1.100	1.068	5377	5278	4932
E85700	0.940	1.147	1.182	5378	5739	6067
H85011	0.940	1.007	0.959	5379	4070	3509
L81131	0.940	1.025	1.026	5380	4325	4424
F86023	0.940	1.012	0.972	5381	4156	3689
D83041	0.940	1.101	1.110	5382	5289	5394
E83012	0.939	1.059	0.995	5383	4789	4014
L81635	0.939	1.225	1.191	5384	6451	6141
L81073	0.939	1.071	1.047	5385	4944	4673
J82195	0.939	1.063	1.048	5386	4847	4688
G82051	0.939	1.101	1.094	5387	5304	5249
G83018	0.939	1.090	1.032	5388	5151	4502
K81094	0.939	0.968	0.939	5389	3529	3241
E85714	0.939	1.036	1.116	5390	4486	5454
C82659	0.939	0.957	0.967	5391	3391	3623
D83070	0.939	1.189	1.145	5392	6157	5753
H82094	0.939	1.248	1.219	5393	6606	6352

P81140	0.939	1.458	1.478	5394	7254	7236
K82070	0.939	1.051	1.001	5395	4695	4093
E85041	0.939	1.002	1.022	5396	4034	4377
P91018	0.939	1.212	1.184	5397	6351	6099
E84680	0.938	1.019	0.982	5398	4259	3864
C88016	0.938	1.049	1.056	5399	4674	4791
L83108	0.938	1.136	1.165	5400	5656	5950
L85027	0.938	1.100	1.075	5401	5297	5048
M92654	0.938	1.178	1.269	5402	6070	6655
P92646	0.938	1.114	1.135	5403	5470	5675
E86003	0.938	1.046	1.014	5404	4637	4294
F81062	0.938	1.034	0.984	5405	4479	3879
K82001	0.938	1.039	0.989	5406	4545	3962
F86679	0.938	1.043	1.088	5407	4596	5190
P92639	0.937	1.168	1.169	5408	5955	5989
G81046	0.937	1.104	1.099	5409	5349	5320
N84025	0.937	1.209	1.162	5410	6338	5933
M83080	0.937	1.067	1.077	5411	4926	5078
L81041	0.937	1.174	1.137	5412	6026	5698
L81052	0.937	1.092	1.061	5413	5214	4872
D83051	0.937	1.090	1.083	5414	5191	5146
C83018	0.937	1.189	1.130	5415	6174	5637
P84061	0.937	1.147	1.194	5416	5758	6177
N84026	0.937	1.044	1.056	5417	4614	4811
G85088	0.937	1.035	1.050	5418	4504	4732
D82009	0.937	1.276	1.210	5419	6766	6313
G83001	0.937	1.089	1.059	5420	5178	4850
H82004	0.936	1.012	0.961	5421	4203	3582
K84059	0.936	1.080	1.050	5422	5081	4737
E84023	0.936	1.028	1.078	5423	4417	5093
H81002	0.936	0.968	0.930	5424	3578	3161
M83115	0.936	1.146	1.139	5425	5760	5728
Y01756	0.936	1.170	1.184	5426	5997	6113
H85082	0.936	1.003	1.017	5427	4068	4351
H82013	0.936	1.020	1.001	5428	4315	4140
H81053	0.936	1.064	0.995	5429	4896	4059
H82017	0.936	1.084	1.035	5430	5138	4588
G82135	0.935	1.004	0.976	5431	4107	3797
M92649	0.935	1.260	1.319	5432	6695	6902
C84602	0.935	1.049	1.161	5433	4707	5940
H82038	0.935	1.168	1.125	5434	5974	5609
F85036	0.935	1.006	1.011	5435	4136	4278
L85002	0.935	1.124	1.078	5436	5583	5109
M83123	0.935	1.200	1.325	5437	6299	6919
B86633	0.935	1.546	1.622	5438	7322	7336
N81002	0.935	1.052	1.014	5439	4763	4329
C84136	0.935	1.120	1.216	5440	5549	6357
F86621	0.934	0.991	1.074	5441	3929	5071
G85046	0.934	1.047	1.071	5442	4700	5041
H84032	0.934	0.972	0.947	5443	3651	3429

L82038	0.934	1.180	1.132	5444	6114	5682
F84122	0.934	1.088	1.053	5445	5208	4804
C82628	0.934	1.073	1.061	5446	5018	4907
C81069	0.934	1.107	1.078	5447	5430	5131
L83059	0.934	1.134	1.119	5448	5671	5548
M85142	0.934	1.275	1.300	5449	6782	6835
A88014	0.934	1.336	1.374	5450	7030	7079
F81718	0.934	1.037	1.118	5451	4573	5539
L83607	0.934	1.281	1.247	5452	6820	6551
E82104	0.933	1.088	1.074	5453	5218	5089
F82639	0.933	1.014	1.101	5454	4278	5372
E81019	0.933	1.155	1.113	5455	5880	5491
N81632	0.932	1.074	1.039	5456	5051	4666
F86607	0.932	0.986	1.024	5457	3880	4493
Y00185	0.932	1.006	0.980	5458	4172	3906
N85040	0.932	1.205	1.236	5459	6353	6511
C84004	0.932	1.197	1.220	5460	6302	6401
E82076	0.932	0.994	0.970	5461	4009	3777
B82002	0.932	1.032	1.011	5462	4542	4327
M84621	0.932	1.120	1.131	5463	5573	5693
H81071	0.932	1.080	1.030	5464	5130	4567
E81032	0.931	1.006	0.984	5465	4192	3968
D83024	0.931	1.059	1.062	5466	4906	4981
C81060	0.931	1.155	1.087	5467	5893	5266
H81109	0.931	0.987	0.956	5468	3928	3587
M85048	0.931	1.188	1.143	5469	6236	5813
F81619	0.931	1.023	1.043	5470	4437	4724
M83134	0.931	1.076	1.179	5471	5091	6116
E82613	0.931	1.185	1.181	5472	6202	6137
P81037	0.930	1.227	1.162	5473	6527	5990
J82110	0.930	1.018	0.961	5474	4358	3661
C87022	0.930	1.257	1.227	5475	6712	6469
H81029	0.930	1.054	1.006	5476	4843	4282
M86005	0.930	1.190	1.265	5477	6262	6679
L82016	0.930	1.229	1.195	5478	6550	6246
F85703	0.930	0.997	1.059	5479	4071	4951
H81064	0.929	1.076	1.008	5480	5115	4319
G83633	0.929	1.050	1.083	5481	4806	5233
P81056	0.929	1.205	1.183	5482	6368	6152
J82164	0.929	1.091	1.071	5483	5302	5104
N82116	0.929	1.083	1.082	5484	5211	5221
M81629	0.929	1.076	1.018	5485	5125	4458
Y02260	0.929	1.013	1.095	5486	4316	5354
F85615	0.928	1.094	1.158	5487	5336	5980
M85697	0.928	1.305	1.340	5488	6954	6995
H81642	0.928	1.048	1.026	5489	4800	4558
F86666	0.928	0.989	1.062	5490	3991	5009
K81032	0.928	1.039	1.016	5491	4681	4454
Y02757	0.928	1.021	1.119	5492	4439	5626
L84056	0.928	1.100	1.099	5493	5421	5402

D82064	0.927	1.084	1.059	5494	5245	4983
J82129	0.927	1.052	1.019	5495	4852	4494
H84034	0.927	1.075	1.014	5496	5134	4434
D81041	0.927	1.037	1.016	5497	4668	4455
N85001	0.927	1.078	1.074	5498	5161	5150
B81052	0.927	1.054	1.183	5499	4882	6172
H85026	0.927	0.968	0.938	5500	3715	3418
D82618	0.927	1.102	1.088	5501	5462	5310
D83053	0.926	1.127	1.100	5502	5676	5424
A82072	0.926	1.554	1.600	5503	7330	7332
J81034	0.926	1.083	1.057	5504	5246	4956
F83673	0.926	1.081	1.151	5505	5223	5936
E82015	0.926	1.018	0.978	5506	4430	3957
H81658	0.926	1.032	0.965	5507	4600	3780
B85622	0.926	1.136	1.152	5508	5755	5943
J83021	0.926	1.100	1.067	5509	5438	5097
D82087	0.926	1.150	1.164	5510	5892	6038
F81209	0.926	1.156	1.229	5511	5954	6506
C81092	0.926	1.038	1.016	5512	4687	4471
Y00475	0.926	1.293	1.283	5513	6915	6793
C81067	0.925	1.106	1.078	5514	5510	5212
H85695	0.925	1.038	1.080	5515	4701	5235
B86052	0.925	1.064	1.070	5516	5008	5132
L83137	0.925	1.172	1.131	5517	6126	5750
M84032	0.925	1.074	1.052	5518	5132	4910
N81088	0.925	1.097	1.054	5519	5414	4937
G85027	0.925	1.100	1.104	5520	5451	5479
P81711	0.925	1.127	1.266	5521	5690	6711
P81077	0.925	1.172	1.093	5522	6124	5370
J82688	0.925	1.024	1.002	5523	4518	4304
L83101	0.925	1.185	1.134	5524	6261	5781
E86632	0.925	0.966	0.939	5525	3720	3450
G82764	0.925	1.164	1.278	5526	6061	6772
E85042	0.925	1.051	1.055	5527	4880	4965
L84617	0.924	1.040	1.074	5528	4733	5180
P92014	0.924	1.095	1.067	5529	5406	5102
C84105	0.924	1.092	1.123	5530	5356	5686
P82025	0.924	1.133	1.156	5531	5749	5992
E85736	0.924	1.033	0.989	5532	4640	4139
P85019	0.924	1.163	1.268	5533	6057	6725
M84010	0.924	1.124	1.102	5534	5663	5461
Y01008	0.924	1.234	1.203	5535	6611	6342
M87014	0.924	1.113	1.108	5536	5579	5531
L83087	0.924	1.244	1.181	5537	6672	6176
H81070	0.924	1.007	0.965	5538	4303	3815
M86605	0.924	1.047	1.028	5539	4839	4633
L83073	0.924	1.280	1.233	5540	6873	6534
E82049	0.924	1.101	1.052	5541	5482	4931
C81051	0.924	1.070	1.051	5542	5107	4916
L83040	0.924	1.120	1.058	5543	5649	5019

E85733	0.924	1.029	1.054	5544	4588	4960
Y00212	0.924	0.957	1.021	5545	3597	4566
M86018	0.923	1.054	1.030	5546	4931	4656
B86011	0.923	1.105	1.112	5547	5516	5592
P81134	0.923	1.221	1.258	5548	6534	6674
F81144	0.923	1.093	1.094	5549	5386	5404
N81022	0.923	1.051	1.022	5550	4898	4574
D81009	0.923	1.075	1.063	5551	5187	5080
J83042	0.923	1.044	1.001	5552	4818	4316
J84013	0.923	1.248	1.214	5553	6700	6424
B86648	0.923	1.260	1.353	5554	6754	7041
C81049	0.923	1.215	1.173	5555	6502	6133
F81169	0.923	1.004	0.960	5556	4272	3753
M85086	0.923	1.178	1.142	5557	6209	5881
L83064	0.923	1.114	1.111	5558	5601	5584
J82010	0.923	1.138	1.081	5559	5799	5281
F86644	0.923	1.015	1.008	5560	4423	4403
G82802	0.923	1.032	1.057	5561	4657	5014
L85066	0.922	1.107	1.098	5562	5545	5439
E84058	0.922	1.076	1.017	5563	5204	4523
C88622	0.922	1.200	1.270	5564	6401	6747
E87045	0.922	0.987	1.038	5565	4046	4772
L85032	0.922	1.084	1.084	5566	5296	5325
J82116	0.922	1.076	1.027	5567	5209	4647
F86025	0.922	1.043	1.025	5568	4817	4618
B81089	0.922	1.133	1.248	5569	5774	6632
P81695	0.922	1.069	1.067	5570	5123	5143
N84627	0.921	1.199	1.193	5571	6396	6292
J81022	0.921	1.265	1.217	5572	6798	6462
H81101	0.921	1.013	0.961	5573	4422	3789
C81634	0.921	1.180	1.137	5574	6250	5840
P84026	0.921	1.029	1.110	5575	4630	5595
J81042	0.921	1.080	1.103	5576	5257	5512
C84125	0.921	1.072	1.051	5577	5168	4959
L81102	0.921	1.142	1.099	5578	5871	5472
J81030	0.921	1.126	1.092	5579	5718	5397
F84009	0.921	0.996	1.043	5580	4188	4852
B86670	0.921	1.103	1.178	5581	5528	6178
C83048	0.920	1.087	1.051	5582	5339	4963
B81642	0.920	1.256	1.351	5583	6745	7045
M91650	0.920	1.311	1.315	5584	6990	6932
B81095	0.920	1.171	1.253	5585	6170	6666
E82037	0.920	0.981	0.960	5586	3989	3783
M84025	0.920	1.072	1.056	5587	5177	5027
E82013	0.920	1.067	1.025	5588	5117	4642
M83084	0.920	1.185	1.150	5589	6298	5978
E84014	0.920	1.006	0.942	5590	4339	3550
J82067	0.920	0.990	0.960	5591	4133	3799
B86044	0.920	1.073	1.079	5592	5205	5294
C84633	0.920	1.023	1.117	5593	4574	5678

C82098	0.920	1.129	1.102	5594	5754	5522
C81077	0.920	1.274	1.251	5595	6860	6658
H84632	0.920	0.978	0.975	5596	3957	4001
M85108	0.919	1.144	1.179	5597	5900	6204
F81071	0.919	1.079	1.060	5598	5282	5095
B81069	0.919	1.316	1.242	5599	7011	6607
P81760	0.919	1.356	1.516	5600	7121	7292
L84065	0.919	1.055	1.036	5601	4988	4794
P81033	0.918	1.293	1.208	5602	6947	6411
G84624	0.918	1.011	1.063	5603	4432	5137
M83026	0.918	1.116	1.036	5604	5657	4788
L82039	0.918	1.039	1.018	5605	4811	4586
P81647	0.918	1.083	1.125	5606	5330	5759
N82054	0.918	1.201	1.273	5607	6439	6782
G85057	0.918	1.074	1.100	5608	5233	5514
H82640	0.918	0.983	0.938	5609	4054	3515
M81026	0.918	1.158	1.105	5610	6074	5575
Y02633	0.918	1.223	1.185	5611	6592	6265
L83002	0.918	1.231	1.162	5612	6643	6087
L83658	0.918	1.104	1.235	5613	5559	6574
L83084	0.917	1.164	1.108	5614	6127	5616
L85062	0.917	1.197	1.121	5615	6418	5733
H83608	0.917	1.006	1.046	5616	4387	4938
E83020	0.917	0.985	0.934	5617	4090	3489
N83622	0.917	1.403	1.481	5618	7212	7261
B81029	0.917	1.174	1.131	5619	6229	5829
L81023	0.917	1.032	1.057	5620	4740	5084
E85024	0.917	1.158	1.145	5621	6083	5962
D82078	0.916	1.094	1.065	5622	5483	5166
M85065	0.916	1.136	1.107	5623	5843	5615
K84023	0.916	1.219	1.153	5624	6573	6029
H81003	0.916	1.056	1.011	5625	5029	4534
J82196	0.916	1.173	1.106	5626	6228	5611
F85020	0.916	1.008	0.989	5627	4416	4241
E84042	0.916	1.050	1.047	5628	4971	4968
J82124	0.915	1.032	0.986	5629	4745	4208
E81012	0.915	1.061	1.033	5630	5102	4793
K81657	0.915	0.995	0.982	5631	4249	4161
F85010	0.915	1.011	1.041	5632	4477	4893
D81631	0.915	0.967	0.952	5633	3859	3763
M85083	0.915	1.040	1.001	5634	4868	4427
P81047	0.915	1.191	1.203	5635	6380	6408
F86078	0.915	1.030	1.025	5636	4738	4709
F84039	0.915	1.012	0.957	5637	4497	3830
M85020	0.914	1.146	1.192	5638	5962	6332
M83681	0.914	1.070	1.013	5639	5235	4570
L81020	0.914	0.991	0.967	5640	4214	3972
M83056	0.914	1.153	1.133	5641	6056	5873
M83640	0.914	1.061	0.981	5642	5109	4158
J81024	0.914	1.049	1.035	5643	4979	4833

P81138	0.914	1.218	1.244	5644	6585	6650
H85047	0.914	1.018	0.958	5645	4584	3866
K81047	0.914	1.045	1.011	5646	4941	4554
F81137	0.914	1.059	1.056	5647	5100	5100
H84625	0.914	1.054	1.049	5648	5039	5020
N82062	0.913	1.080	1.119	5649	5350	5749
L85044	0.913	1.140	1.110	5650	5925	5676
N81102	0.913	1.278	1.248	5651	6907	6678
C81046	0.913	1.099	1.063	5652	5558	5186
M85715	0.913	1.089	1.120	5653	5465	5758
E82124	0.913	1.039	1.021	5654	4875	4686
E82055	0.912	1.098	1.064	5655	5552	5203
F84716	0.912	0.945	1.016	5656	3582	4624
J82009	0.912	1.112	1.076	5657	5673	5339
L85619	0.912	1.170	1.141	5658	6244	5975
B81050	0.912	1.157	1.107	5659	6112	5655
G82011	0.911	1.032	1.081	5660	4814	5392
E85049	0.911	0.993	0.986	5661	4283	4265
H81110	0.911	1.012	0.996	5662	4546	4394
H84051	0.911	1.035	1.022	5663	4854	4715
F81757	0.911	1.130	1.127	5664	5844	5850
G81039	0.911	1.142	1.050	5665	5970	5070
D82076	0.910	1.121	1.180	5666	5769	6274
G83030	0.910	1.060	1.090	5667	5141	5488
M83132	0.910	1.050	1.002	5668	5026	4484
F84034	0.910	1.163	1.118	5669	6182	5760
E85103	0.910	1.073	1.094	5670	5310	5529
J81003	0.910	1.007	1.000	5671	4496	4463
K81021	0.910	1.013	0.967	5672	4575	4028
L81017	0.910	1.009	1.006	5673	4517	4548
B86010	0.910	1.048	1.064	5674	5007	5239
H81042	0.910	1.102	1.024	5675	5608	4749
E81020	0.910	1.117	1.082	5676	5735	5417
M81044	0.910	1.160	1.112	5677	6160	5717
G82652	0.909	1.215	1.253	5678	6590	6724
E85045	0.909	1.095	1.119	5679	5555	5780
D83074	0.909	1.071	1.070	5680	5301	5308
A81027	0.909	1.054	1.040	5681	5082	4972
P81740	0.909	1.053	1.097	5682	5085	5582
E83010	0.909	1.029	0.978	5683	4801	4200
G81680	0.909	1.212	1.266	5684	6583	6788
C81007	0.909	1.098	1.120	5685	5584	5794
E85029	0.909	1.130	1.188	5686	5864	6348
E82017	0.909	1.013	0.964	5687	4583	4015
C82022	0.909	1.031	0.988	5688	4824	4334
L83035	0.909	1.149	1.108	5689	6071	5690
C83040	0.909	1.115	1.055	5690	5731	5152
C84094	0.909	1.140	1.085	5691	5963	5446
J83041	0.908	1.059	1.030	5692	5162	4838
J81017	0.908	1.095	1.050	5693	5563	5099

C81015	0.908	1.141	1.094	5694	5984	5565
K81072	0.908	0.963	0.972	5695	3886	4109
P87627	0.908	1.120	1.236	5696	5778	6638
M85757	0.908	1.016	1.065	5697	4627	5274
F82033	0.908	1.054	1.021	5698	5108	4735
M92643	0.908	1.019	1.086	5699	4676	5482
B86058	0.908	1.159	1.194	5700	6172	6390
G81029	0.908	1.183	1.123	5701	6369	5836
J82145	0.908	1.079	1.043	5702	5408	5029
B83019	0.908	1.030	1.033	5703	4831	4885
C81035	0.908	1.161	1.127	5704	6196	5874
P89022	0.908	1.155	1.242	5705	6128	6675
J82088	0.907	1.163	1.152	5706	6217	6090
E85732	0.907	1.087	1.050	5707	5503	5110
C87018	0.907	1.132	1.127	5708	5904	5885
N83637	0.907	1.135	1.214	5709	5941	6523
B86018	0.907	1.136	1.115	5710	5945	5769
E83653	0.906	1.017	1.006	5711	4670	4582
N82617	0.906	0.993	1.036	5712	4365	4949
H82051	0.906	1.029	1.033	5713	4835	4917
M85075	0.906	1.095	1.139	5714	5582	6005
L82024	0.906	1.327	1.351	5715	7081	7090
A83006	0.906	1.254	1.224	5716	6826	6581
H82016	0.905	1.164	1.120	5717	6248	5831
Y00184	0.905	0.971	1.005	5718	4060	4592
E84008	0.905	1.068	0.983	5719	5316	4320
C83073	0.905	1.107	1.190	5720	5688	6384
J82136	0.904	1.022	0.995	5721	4762	4470
P81762	0.904	1.121	1.193	5722	5817	6409
G81684	0.904	0.997	1.102	5723	4431	5677
P87026	0.904	1.170	1.225	5724	6305	6594
J82036	0.904	1.055	1.000	5725	5154	4533
F83051	0.904	1.036	1.127	5726	4952	5907
J83646	0.904	1.034	1.024	5727	4925	4830
E86612	0.904	1.005	0.991	5728	4550	4432
G84006	0.904	1.063	1.031	5729	5267	4912
E84015	0.904	1.114	1.062	5730	5762	5286
E85734	0.904	1.022	1.010	5731	4774	4653
J81637	0.904	1.128	1.120	5732	5898	5853
M88630	0.903	1.054	1.124	5733	5166	5891
M87016	0.903	1.136	1.122	5734	5987	5870
E82045	0.903	1.008	0.952	5735	4591	3929
L83095	0.903	1.163	1.112	5736	6258	5777
M88625	0.903	1.008	1.072	5737	4592	5385
J82018	0.903	1.058	1.029	5738	5227	4905
G85685	0.902	1.116	1.099	5739	5789	5672
B82012	0.902	1.073	1.063	5740	5399	5306
D82017	0.902	1.211	1.224	5741	6607	6601
J82123	0.902	1.063	1.008	5742	5288	4651
C87603	0.902	1.225	1.302	5743	6686	6952

H82027	0.902	1.020	0.973	5744	4770	4230
C82052	0.902	1.130	1.072	5745	5940	5393
M88616	0.902	1.230	1.294	5746	6715	6927
P81017	0.902	1.075	1.043	5747	5440	5092
C82034	0.902	1.032	1.021	5748	4938	4822
E81045	0.901	1.127	1.082	5749	5909	5502
A82038	0.901	1.066	1.017	5750	5329	4769
D81625	0.900	0.975	0.963	5751	4187	4098
P88015	0.900	1.056	1.037	5752	5231	5055
F86018	0.900	1.035	1.062	5753	4978	5321
A88009	0.900	1.091	1.064	5754	5600	5338
F84086	0.900	1.087	1.130	5755	5569	5982
P92630	0.900	1.135	1.204	5756	6008	6514
N81121	0.900	1.128	1.079	5757	5939	5486
C88005	0.900	1.098	1.095	5758	5658	5651
L81644	0.900	1.062	1.031	5759	5305	4978
M91014	0.899	1.156	1.227	5760	6224	6634
H81074	0.899	1.025	0.976	5761	4872	4286
M92629	0.899	1.156	1.241	5762	6221	6713
M88005	0.899	1.099	1.096	5763	5662	5670
M81616	0.899	1.043	1.085	5764	5074	5553
H81116	0.899	1.078	1.022	5765	5497	4860
G83673	0.899	1.073	1.152	5766	5444	6144
G85081	0.899	1.020	1.045	5767	4810	5147
H82053	0.899	1.148	1.134	5768	6150	6016
E87739	0.899	0.996	0.962	5769	4483	4096
H85057	0.899	0.947	0.924	5770	3792	3569
L81088	0.899	1.158	1.145	5771	6247	6097
L83146	0.899	1.126	1.141	5772	5924	6065
B83624	0.899	1.065	1.054	5773	5347	5255
H81086	0.899	1.011	0.986	5774	4694	4439
F83052	0.899	1.041	1.050	5775	5063	5204
L85035	0.899	1.119	1.091	5776	5858	5628
J82046	0.899	1.052	1.008	5777	5198	4696
C81005	0.898	1.189	1.140	5778	6491	6063
H81046	0.898	1.022	0.976	5779	4846	4310
N81040	0.898	1.146	1.083	5780	6138	5536
H81129	0.898	0.992	0.946	5781	4450	3914
F81205	0.898	1.301	1.253	5782	7034	6784
F82610	0.898	1.080	1.133	5783	5524	6019
L85019	0.897	1.146	1.083	5784	6146	5561
Y03755	0.897	0.988	1.018	5785	4410	4840
C81016	0.897	1.092	1.069	5786	5636	5413
G82649	0.897	1.261	1.269	5787	6905	6853
P82003	0.897	1.120	1.120	5788	5883	5914
E83031	0.897	0.999	0.962	5789	4567	4137
C81073	0.896	1.121	1.095	5790	5895	5688
C81003	0.896	1.121	1.089	5791	5897	5633
A88611	0.896	1.267	1.328	5792	6931	7051
A86033	0.896	1.007	1.026	5793	4667	4944

L83666	0.896	1.196	1.139	5794	6548	6077
P84042	0.896	1.129	1.146	5795	5988	6132
D82624	0.896	1.048	1.016	5796	5182	4820
M91621	0.896	1.097	1.110	5797	5684	5832
C84030	0.896	1.153	1.130	5798	6240	6015
G82139	0.895	1.111	1.107	5799	5805	5803
L84078	0.895	1.063	1.039	5800	5358	5123
J82042	0.895	1.094	1.058	5801	5661	5326
L85033	0.895	1.144	1.107	5802	6148	5806
M91626	0.895	1.163	1.150	5803	6314	6164
H84014	0.894	0.941	0.939	5804	3783	3872
B82008	0.894	1.141	1.099	5805	6120	5738
F81684	0.894	1.202	1.243	5806	6602	6753
Y02212	0.894	1.170	1.167	5807	6373	6309
P81129	0.894	1.157	1.120	5808	6281	5942
G84002	0.894	0.980	0.948	5809	4334	3985
K82023	0.894	1.050	1.009	5810	5230	4765
G82098	0.894	1.087	1.061	5811	5616	5362
K81097	0.894	0.995	0.950	5812	4551	4025
A86035	0.894	1.166	1.257	5813	6348	6819
H83025	0.894	1.047	1.014	5814	5197	4825
L82061	0.893	1.116	1.102	5815	5884	5772
M81014	0.893	1.121	1.076	5816	5938	5524
L83148	0.893	1.193	1.163	5817	6552	6276
E85657	0.893	1.000	0.984	5818	4620	4476
C82067	0.893	1.078	1.052	5819	5554	5287
K82618	0.893	1.027	0.993	5820	4970	4598
C84703	0.893	1.039	1.009	5821	5113	4781
N81033	0.893	1.021	0.996	5822	4910	4625
C81062	0.892	1.120	1.090	5823	5933	5681
G81076	0.892	1.014	1.013	5824	4825	4831
J83062	0.892	1.146	1.118	5825	6189	5945
C82121	0.892	1.129	1.088	5826	6033	5658
P87004	0.892	1.298	1.403	5827	7041	7204
H81088	0.892	1.031	1.010	5828	5016	4800
N84611	0.892	1.314	1.329	5829	7086	7070
E83629	0.892	1.234	1.174	5830	6789	6366
B86086	0.891	1.021	1.088	5831	4923	5661
P85606	0.891	1.128	1.169	5832	6024	6331
L85056	0.891	1.136	1.124	5833	6108	6001
F86081	0.891	1.001	0.977	5834	4662	4423
K84002	0.891	1.083	1.032	5835	5604	5085
K81014	0.891	1.061	1.067	5836	5387	5443
L83009	0.891	1.191	1.177	5837	6549	6388
F84677	0.891	1.060	1.079	5838	5380	5580
E82075	0.891	1.139	1.112	5839	6144	5895
E81007	0.890	1.067	1.044	5840	5475	5226
M83093	0.890	1.073	0.994	5841	5527	4630
M82014	0.890	1.107	1.093	5842	5819	5718
G84001	0.890	0.982	0.958	5843	4429	4181

Y02164	0.890	1.060	1.106	5844	5398	5841
C82092	0.890	1.098	1.164	5845	5742	6315
L85009	0.890	1.156	1.093	5846	6308	5724
M83691	0.890	1.043	1.031	5847	5195	5091
K81007	0.890	1.060	1.086	5848	5397	5656
L83050	0.890	1.182	1.131	5849	6501	6057
G85102	0.889	1.012	1.067	5850	4840	5467
C83051	0.889	1.064	1.085	5851	5448	5653
E86015	0.889	1.089	1.036	5852	5668	5153
E82658	0.889	1.035	0.995	5853	5098	4658
B81070	0.889	1.373	1.314	5854	7209	7030
M84055	0.889	1.106	1.086	5855	5826	5680
N81009	0.889	1.126	1.085	5856	6037	5663
E83018	0.888	1.041	0.982	5857	5184	4511
K81062	0.888	1.032	1.017	5858	5071	4941
K81060	0.888	0.987	0.968	5859	4519	4339
M92006	0.888	1.113	1.148	5860	5903	6202
E86038	0.888	1.016	0.987	5861	4895	4573
C88091	0.888	1.348	1.453	5862	7170	7263
E83032	0.888	0.998	0.939	5863	4669	3950
M83650	0.887	1.030	1.123	5864	5058	6024
M82016	0.887	1.163	1.140	5865	6365	6145
N81055	0.887	1.121	1.069	5866	5991	5518
F82016	0.887	1.085	1.069	5867	5654	5509
M88637	0.887	1.175	1.287	5868	6476	6955
L84067	0.887	1.126	1.090	5869	6064	5730
J81031	0.886	1.055	1.033	5870	5376	5149
J81628	0.886	1.190	1.158	5871	6578	6287
F86049	0.886	1.019	1.083	5872	4958	5669
L85065	0.886	1.170	1.135	5873	6445	6127
A86012	0.886	1.142	1.170	5874	6220	6381
G82088	0.885	1.054	1.024	5875	5377	5064
P91603	0.885	1.079	1.063	5876	5622	5465
B81692	0.885	1.113	1.271	5877	5937	6909
P83001	0.885	1.052	1.040	5878	5352	5246
M82003	0.885	1.154	1.135	5879	6322	6130
H82026	0.885	1.043	1.044	5880	5253	5289
C81097	0.884	1.163	1.106	5881	6410	5901
G83058	0.884	1.128	1.207	5882	6101	6604
E85115	0.884	1.103	1.070	5883	5838	5560
C84676	0.884	1.048	1.140	5884	5317	6165
K84049	0.884	0.939	0.933	5885	3896	3919
J82012	0.884	1.160	1.109	5886	6370	5930
E81002	0.884	1.144	1.131	5887	6256	6105
C85015	0.884	1.245	1.246	5888	6899	6811
J84007	0.884	1.190	1.145	5889	6596	6212
G81006	0.884	1.144	1.187	5890	6265	6512
B84005	0.884	1.122	1.127	5891	6052	6081
L83043	0.883	1.079	1.064	5892	5638	5504
G85039	0.883	1.030	1.013	5893	5114	4947

G85032	0.883	1.038	1.013	5894	5217	4946
L84002	0.883	1.160	1.139	5895	6397	6168
E87038	0.883	1.060	1.099	5896	5480	5845
P83015	0.883	1.274	1.237	5897	6995	6780
P89017	0.883	1.215	1.269	5898	6734	6910
C81030	0.882	1.185	1.156	5899	6568	6311
P92648	0.882	1.181	1.256	5900	6538	6858
J82168	0.882	1.159	1.186	5901	6395	6513
K82046	0.882	1.005	0.963	5902	4837	4352
L85022	0.882	1.181	1.145	5903	6544	6232
J82177	0.882	1.136	1.161	5904	6199	6343
M84020	0.882	1.055	1.018	5905	5426	5044
G81073	0.882	1.165	1.181	5906	6438	6488
C88049	0.882	1.156	1.187	5907	6362	6519
J82072	0.882	1.085	1.042	5908	5706	5300
G85042	0.881	1.069	1.124	5909	5567	6072
M86022	0.881	1.099	1.140	5910	5834	6192
P89033	0.881	1.266	1.283	5911	6974	6963
M85717	0.881	1.455	1.479	5912	7296	7293
C84039	0.881	1.033	1.084	5913	5183	5725
K81052	0.881	1.061	1.036	5914	5511	5250
C81039	0.881	1.137	1.093	5915	6216	5808
K84033	0.881	1.054	0.998	5916	5427	4792
L84006	0.880	0.993	0.965	5917	4696	4397
H81010	0.880	0.989	0.934	5918	4647	3982
J81009	0.880	1.212	1.172	5919	6731	6429
E85060	0.880	1.082	1.091	5920	5695	5793
E85640	0.880	0.962	1.022	5921	4289	5098
K81630	0.880	1.020	0.975	5922	5028	4529
G82041	0.880	1.012	0.993	5923	4951	4734
M83623	0.880	1.098	1.189	5924	5833	6529
G83039	0.880	1.193	1.261	5925	6638	6893
H84017	0.879	0.961	0.917	5926	4277	3751
C82021	0.879	1.128	1.076	5927	6152	5667
M85176	0.879	1.109	1.118	5928	5950	6037
G81667	0.879	1.059	1.079	5929	5502	5702
H85656	0.879	0.978	1.019	5930	4516	5073
M92007	0.879	1.192	1.227	5931	6642	6752
N81651	0.879	1.054	1.100	5932	5459	5886
N81046	0.879	1.095	1.050	5933	5818	5407
Y01922	0.879	1.139	1.113	5934	6260	6013
H81020	0.879	0.991	0.959	5935	4698	4345
K82015	0.879	1.095	1.039	5936	5814	5299
G83016	0.878	1.032	1.054	5937	5206	5444
E82064	0.878	1.034	1.019	5938	5232	5082
M82004	0.878	1.112	1.085	5939	5990	5755
E82129	0.878	1.005	0.979	5940	4883	4606
H82072	0.878	1.048	1.027	5941	5393	5174
J82005	0.878	1.037	1.067	5942	5271	5597
N83007	0.878	1.233	1.293	5943	6867	7009

A82039	0.877	1.178	1.193	5944	6557	6564
M92039	0.877	1.116	1.141	5945	6054	6234
P89007	0.877	1.203	1.201	5946	6701	6611
J82138	0.877	1.056	1.009	5947	5493	4984
C82014	0.877	1.163	1.132	5948	6456	6156
E85114	0.877	1.071	1.046	5949	5626	5384
K81647	0.877	0.956	0.954	5950	4239	4307
E85119	0.877	1.053	1.092	5951	5461	5835
K81613	0.877	1.017	1.021	5952	5037	5129
K84044	0.877	1.038	1.009	5953	5295	4991
N81038	0.877	1.119	1.067	5954	6093	5621
E85052	0.876	0.991	0.968	5955	4727	4488
G85086	0.876	1.026	1.048	5956	5155	5411
P81667	0.876	1.171	1.223	5957	6513	6743
P87660	0.876	1.078	1.188	5958	5686	6544
H82086	0.876	1.164	1.172	5959	6471	6465
D82102	0.876	1.198	1.173	5960	6680	6476
C83047	0.876	1.071	1.020	5961	5641	5133
E85125	0.876	0.990	1.026	5962	4714	5194
Y00075	0.875	1.178	1.233	5963	6570	6797
J81083	0.875	1.111	1.082	5964	6007	5754
P84035	0.875	1.045	1.075	5965	5396	5701
Y01011	0.875	1.030	1.083	5966	5224	5768
J82156	0.875	1.137	1.097	5967	6274	5904
P81043	0.875	1.344	1.411	5968	7189	7232
F86028	0.875	1.016	0.988	5969	5047	4731
Y01051	0.875	1.116	1.102	5970	6081	5960
H82040	0.874	1.040	1.006	5971	5334	4986
M85163	0.874	1.115	1.119	5972	6072	6091
M88012	0.874	1.151	1.175	5973	6385	6496
K82016	0.874	1.076	1.052	5974	5691	5469
L82006	0.874	1.080	1.074	5975	5726	5700
L81127	0.874	1.022	1.014	5976	5127	5075
B82053	0.874	1.121	1.057	5977	6134	5525
L83039	0.874	1.144	1.162	5978	6331	6405
G82653	0.874	1.018	1.110	5979	5083	6032
F81032	0.873	1.106	1.070	5980	5981	5662
F85701	0.873	1.102	1.173	5981	5948	6493
E83650	0.873	1.008	1.042	5982	4984	5388
E83027	0.873	0.950	0.931	5983	4221	4047
L83075	0.872	1.083	1.076	5984	5761	5732
P89030	0.872	1.185	1.256	5985	6631	6901
F85029	0.872	1.058	1.026	5986	5557	5231
H82052	0.872	1.017	1.011	5987	5095	5067
L82029	0.872	1.068	1.049	5988	5647	5455
E84032	0.872	1.033	1.027	5989	5287	5248
L83044	0.872	1.194	1.182	5990	6681	6536
C84619	0.872	1.097	1.209	5991	5901	6690
E84657	0.872	1.039	0.993	5992	5348	4844
Y02378	0.872	1.243	1.266	5993	6935	6931

F81117	0.872	1.038	1.006	5994	5346	5015
E85128	0.872	1.004	1.005	5995	4955	5003
Y01281	0.871	1.152	1.106	5996	6420	6014
C81031	0.871	1.160	1.110	5997	6475	6043
N82108	0.871	1.045	1.103	5998	5442	5993
P81062	0.871	1.172	1.102	5999	6561	5985
H81122	0.871	0.982	0.924	6000	4673	3973
C82086	0.871	1.108	1.111	6001	6041	6048
G85113	0.871	1.077	1.150	6002	5725	6346
M85174	0.871	1.031	1.084	6003	5286	5822
M86006	0.871	1.042	1.025	6004	5413	5238
H81663	0.870	0.944	0.905	6005	4175	3717
Y00347	0.870	1.133	1.067	6006	6273	5659
G85041	0.870	0.981	1.002	6007	4671	4975
E82085	0.870	1.029	1.025	6008	5270	5253
L84025	0.870	1.033	1.019	6009	5319	5178
P91007	0.870	1.092	1.163	6010	5882	6441
B82104	0.869	1.046	1.026	6011	5468	5268
F84097	0.869	1.049	1.041	6012	5501	5412
L83026	0.869	1.156	1.126	6013	6459	6171
M84013	0.869	1.047	1.019	6014	5486	5182
M85153	0.869	1.068	1.083	6015	5660	5830
H81045	0.869	1.036	0.983	6016	5361	4756
H82010	0.868	1.083	1.049	6017	5800	5501
F82002	0.868	1.027	0.998	6018	5261	4955
H81066	0.868	1.011	0.953	6019	5075	4409
C84085	0.867	1.114	1.124	6020	6121	6179
G82634	0.867	1.118	1.088	6021	6165	5897
F81083	0.867	1.097	1.079	6022	5958	5807
L83030	0.867	1.203	1.241	6023	6747	6862
K82006	0.867	1.048	0.986	6024	5518	4806
C84682	0.867	1.020	1.137	6025	5201	6280
L83644	0.867	1.157	1.195	6026	6488	6642
F86675	0.866	0.979	1.005	6027	4704	5069
G84009	0.866	1.015	1.028	6028	5136	5316
G81083	0.866	1.045	1.051	6029	5495	5538
B86069	0.866	0.994	1.040	6030	4892	5433
H82036	0.866	1.080	1.047	6031	5792	5508
G85028	0.866	1.036	1.016	6032	5401	5187
G84609	0.866	1.097	1.084	6033	5960	5866
K84071	0.865	1.086	1.051	6034	5862	5563
J82633	0.865	1.132	1.094	6035	6310	5970
E82105	0.865	1.076	1.079	6036	5764	5823
Y01964	0.865	1.038	1.021	6037	5428	5264
L83016	0.865	0.995	0.943	6038	4928	4323
M81621	0.865	1.077	1.031	6039	5783	5357
F81742	0.865	0.942	0.971	6040	4224	4662
P84018	0.865	0.970	1.036	6041	4599	5410
C82639	0.864	1.086	1.091	6042	5861	5948
P81177	0.864	1.062	1.092	6042	5653	5956

E84704	0.864	1.097	1.143	6043	5982	6341
D82628	0.864	1.188	1.147	6044	6691	6369
E82069	0.864	0.992	0.963	6045	4897	4579
M85735	0.864	1.117	1.157	6046	6188	6439
K84624	0.864	1.080	1.050	6047	5812	5559
E82643	0.864	1.078	1.085	6048	5794	5900
B81061	0.863	1.118	1.088	6049	6203	5938
L81060	0.863	1.058	1.019	6050	5633	5265
E84018	0.863	1.037	0.964	6051	5447	4608
B86682	0.863	1.115	1.162	6052	6180	6491
H82041	0.863	1.181	1.142	6053	6662	6345
Y00092	0.863	0.918	0.988	6054	3910	4889
D83022	0.862	1.173	1.162	6055	6612	6492
J83645	0.862	1.063	1.093	6056	5674	5987
F83674	0.862	1.118	1.205	6057	6222	6718
G84039	0.862	1.052	1.068	6058	5586	5745
Y00033	0.862	0.993	0.971	6059	4940	4693
M82033	0.861	1.061	1.039	6060	5666	5466
J82049	0.861	1.122	1.054	6061	6266	5638
G82152	0.860	1.104	1.092	6062	6102	5995
F85675	0.860	0.954	1.014	6063	4448	5232
C88028	0.860	0.993	1.014	6064	4956	5223
G82226	0.860	1.029	1.048	6065	5374	5578
M87005	0.860	1.128	1.097	6066	6317	6031
J81016	0.860	1.086	1.056	6067	5920	5665
H82070	0.860	1.101	1.042	6068	6087	5519
H85090	0.860	1.022	1.003	6069	5309	5116
C82040	0.859	1.081	1.058	6070	5868	5683
E85057	0.859	1.041	1.030	6071	5521	5391
K84622	0.859	1.043	0.983	6072	5543	4873
N84008	0.859	1.178	1.168	6073	6669	6530
H81632	0.859	1.076	1.063	6074	5828	5737
C82015	0.859	1.292	1.353	6075	7109	7178
G85006	0.859	1.019	1.016	6076	5280	5280
P91013	0.859	1.085	1.048	6077	5915	5596
H83013	0.859	1.062	1.031	6078	5701	5422
F81744	0.859	1.195	1.304	6079	6752	7089
E83041	0.859	0.950	0.980	6080	4414	4837
F84046	0.858	1.059	1.086	6081	5680	5958
F81003	0.858	1.147	1.179	6082	6479	6595
N84038	0.858	1.303	1.367	6083	7141	7202
M85064	0.858	1.009	1.059	6084	5167	5708
E82084	0.858	1.062	1.040	6085	5715	5520
B86066	0.858	1.075	1.078	6086	5824	5888
K82078	0.858	1.012	0.965	6087	5215	4675
G82154	0.858	1.188	1.199	6088	6721	6714
D82022	0.857	1.144	1.099	6089	6458	6056
B82095	0.857	1.090	1.070	6090	5985	5816
L81080	0.857	1.087	1.058	6091	5949	5707
J81066	0.857	1.059	1.023	6092	5685	5351

L83065	0.857	1.181	1.186	6093	6697	6646
G85045	0.856	1.055	1.092	6094	5659	6023
L81648	0.856	1.066	1.127	6095	5757	6285
F84741	0.856	1.048	1.049	6096	5599	5635
P85612	0.856	1.122	1.167	6097	6306	6540
E85649	0.856	0.989	1.067	6098	4957	5799
F81672	0.856	1.202	1.224	6099	6818	6841
G82082	0.856	0.992	0.984	6100	4991	4934
E81047	0.855	1.017	1.000	6101	5299	5140
J83629	0.855	1.076	1.034	6102	5874	5485
E86016	0.855	1.046	1.040	6103	5596	5550
A89034	0.855	1.072	1.171	6104	5829	6569
D82047	0.855	1.211	1.220	6105	6871	6831
B82003	0.855	1.059	1.032	6106	5717	5457
F84672	0.855	1.289	1.326	6107	7112	7150
N82641	0.854	1.134	1.170	6108	6417	6563
G85050	0.854	1.109	1.152	6109	6200	6470
H82012	0.854	1.171	1.127	6110	6657	6310
G82095	0.854	1.112	1.109	6111	6237	6159
P81041	0.854	1.097	1.095	6112	6092	6058
B82097	0.854	1.130	1.088	6113	6381	6006
G83668	0.854	1.056	1.130	6114	5692	6320
K81010	0.854	0.997	0.964	6115	5070	4704
M81047	0.854	1.217	1.157	6116	6902	6509
G85087	0.853	1.010	1.023	6117	5243	5382
Y02594	0.853	1.125	1.035	6118	6337	5517
F84016	0.853	1.087	1.098	6119	5993	6089
F86712	0.853	0.948	1.007	6120	4467	5228
A83046	0.853	1.158	1.135	6121	6591	6365
G82780	0.852	1.049	1.014	6122	5648	5312
E87735	0.852	1.224	1.240	6123	6934	6913
C82649	0.852	1.103	1.046	6124	6166	5645
J82161	0.852	1.100	1.048	6125	6143	5654
H84048	0.852	0.990	0.957	6126	5000	4644
B81097	0.852	1.297	1.434	6127	7139	7283
G84022	0.852	1.039	1.032	6128	5568	5495
J83063	0.852	0.971	0.988	6129	4782	5043
B86655	0.852	1.078	1.167	6130	5926	6562
M85136	0.852	1.134	1.182	6131	6434	6653
G82205	0.851	1.025	0.987	6132	5433	5028
A89603	0.851	1.271	1.352	6133	7074	7188
F85043	0.851	1.032	1.061	6134	5514	5786
M83711	0.851	1.193	1.263	6135	6786	6999
G82161	0.851	1.120	1.123	6136	6321	6289
B81030	0.851	1.126	1.134	6137	6358	6367
E85008	0.851	1.056	1.057	6138	5719	5751
M85069	0.851	1.082	1.064	6139	5959	5818
M84629	0.851	1.127	1.134	6140	6379	6370
C82657	0.851	1.112	1.140	6141	6272	6412
M92016	0.850	1.076	1.143	6142	5907	6434

L83023	0.850	1.172	1.124	6143	6679	6312
J81613	0.850	1.147	1.128	6144	6524	6335
M82002	0.850	1.056	1.055	6145	5727	5742
C86026	0.850	1.251	1.255	6146	7028	6969
P85022	0.849	1.113	1.201	6147	6290	6764
L81084	0.849	1.137	1.127	6148	6468	6330
P81084	0.849	1.125	1.113	6149	6374	6242
G85133	0.849	1.060	1.059	6150	5768	5779
G85705	0.849	0.984	0.975	6151	4976	4895
L83024	0.849	1.147	1.100	6152	6530	6139
H84053	0.849	1.101	1.070	6153	6183	5893
Y02325	0.849	1.118	1.202	6154	6324	6770
C84122	0.849	1.086	1.095	6155	6036	6098
E85051	0.849	1.032	1.011	6156	5538	5324
C84115	0.849	1.028	0.996	6157	5505	5156
H81078	0.849	1.048	1.004	6158	5667	5251
H82028	0.849	1.051	0.990	6159	5687	5094
P92035	0.848	1.152	1.229	6160	6581	6887
A82074	0.848	1.160	1.099	6161	6619	6138
E81043	0.848	1.032	0.999	6162	5539	5195
L84040	0.848	1.112	1.114	6163	6292	6249
C84042	0.848	1.246	1.217	6164	7014	6842
P84038	0.848	1.130	1.162	6165	6429	6555
Y03049	0.848	0.947	0.972	6166	4522	4875
P89005	0.848	1.160	1.182	6167	6634	6676
N82089	0.848	1.147	1.233	6168	6551	6906
F81756	0.847	1.270	1.271	6169	7082	7026
Y00412	0.847	1.154	1.197	6170	6599	6755
F86616	0.847	1.073	1.121	6171	5919	6316
G81020	0.847	0.984	1.087	6172	4997	6046
F84676	0.847	1.061	1.128	6173	5797	6358
J83050	0.846	1.078	1.046	6174	5969	5689
N85007	0.846	1.116	1.096	6175	6325	6129
L85018	0.846	1.281	1.242	6176	7114	6942
M82619	0.845	1.065	1.073	6177	5845	5953
C84008	0.845	1.079	1.016	6178	5992	5400
P84010	0.845	1.172	1.226	6179	6709	6884
M81078	0.845	1.060	1.005	6180	5803	5298
G82741	0.845	1.052	1.108	6181	5733	6225
B83031	0.844	1.166	1.209	6182	6678	6828
G82775	0.844	1.048	1.139	6183	5712	6451
P83621	0.844	1.156	1.276	6184	6624	7052
E85726	0.844	0.969	1.028	6185	4858	5530
B82014	0.844	1.061	1.034	6186	5825	5614
F86042	0.844	1.039	1.015	6187	5637	5398
E85099	0.844	0.989	1.060	6188	5096	5847
Y02423	0.843	0.971	0.921	6189	4894	4313
L84005	0.843	1.069	1.052	6190	5912	5775
F83002	0.843	1.080	1.083	6191	6029	6049
P84009	0.843	1.099	1.194	6192	6219	6758

G84008	0.843	0.938	0.918	6193	4468	4276
F85007	0.843	1.028	1.044	6194	5556	5709
P81146	0.842	1.086	1.145	6195	6103	6503
M86009	0.842	0.980	0.970	6196	5006	4920
B81058	0.842	1.249	1.369	6197	7047	7221
N81101	0.842	1.053	1.018	6198	5775	5453
F82686	0.842	0.955	1.029	6199	4713	5583
G82104	0.841	1.055	1.062	6200	5788	5890
G84024	0.841	1.026	0.995	6201	5551	5222
H82089	0.841	1.058	1.009	6202	5821	5371
E83657	0.841	0.951	0.961	6203	4660	4814
L83046	0.841	1.137	1.110	6204	6518	6272
Y03112	0.841	1.240	1.369	6205	7024	7225
L81061	0.841	1.092	1.082	6206	6168	6054
N82094	0.841	1.240	1.210	6207	7019	6844
G85019	0.841	1.044	1.103	6208	5709	6217
A86007	0.841	0.961	0.986	6209	4805	5141
D82018	0.840	1.051	1.008	6210	5767	5365
M82044	0.840	1.140	1.097	6211	6545	6175
L81632	0.840	1.006	0.996	6212	5333	5257
M83130	0.840	1.180	1.192	6213	6776	6762
C81648	0.840	1.062	1.093	6214	5876	6146
F86004	0.840	1.017	1.011	6215	5477	5401
E81013	0.840	1.043	1.027	6216	5700	5577
F81114	0.840	1.147	1.117	6217	6600	6327
F83648	0.840	1.117	1.198	6218	6394	6800
F85645	0.840	0.975	1.030	6219	4983	5612
E87024	0.839	1.137	1.165	6220	6528	6624
G84033	0.839	0.982	0.946	6221	5062	4664
C82038	0.839	1.110	1.063	6222	6334	5921
C81094	0.839	1.065	1.033	6223	5914	5643
E81633	0.838	1.099	1.201	6224	6263	6815
E85116	0.838	1.131	1.169	6225	6503	6657
F83023	0.838	1.050	1.023	6226	5780	5555
F83033	0.838	1.088	1.157	6227	6159	6582
M86039	0.838	1.073	1.048	6228	6005	5789
M82034	0.838	1.092	1.083	6229	6197	6095
E85723	0.837	1.073	1.148	6230	6006	6538
D81066	0.837	1.016	0.967	6231	5498	4952
H84018	0.837	1.005	0.997	6232	5357	5295
M91619	0.837	1.084	1.181	6233	6122	6729
H82621	0.837	1.129	1.087	6234	6500	6135
E81626	0.837	0.988	0.993	6235	5158	5260
C81047	0.837	1.180	1.143	6236	6793	6521
M88619	0.837	1.188	1.243	6237	6840	6973
Y00206	0.837	0.989	1.026	6238	5190	5607
J82131	0.837	1.119	1.085	6239	6436	6121
J82030	0.836	1.074	1.017	6240	6043	5499
J82192	0.836	0.986	0.952	6241	5145	4761
F84053	0.836	0.993	1.021	6242	5242	5540

N81001	0.836	1.106	1.065	6243	6327	5973
F85002	0.836	1.055	1.017	6244	5839	5497
H85002	0.836	1.008	0.962	6245	5412	4898
H82087	0.836	1.144	1.092	6246	6603	6163
E85050	0.836	1.010	0.990	6247	5432	5236
P91016	0.836	1.096	1.094	6248	6252	6189
Y02571	0.836	0.995	1.052	6249	5260	5855
P81071	0.836	1.186	1.136	6250	6834	6495
C88062	0.836	0.951	0.966	6251	4737	4954
L85043	0.835	1.095	1.041	6252	6254	5741
P84650	0.835	1.087	1.134	6253	6171	6486
H85061	0.835	0.954	0.920	6254	4780	4402
J82017	0.835	1.125	1.080	6255	6484	6093
H85114	0.835	0.918	0.890	6256	4293	3996
G85011	0.835	0.960	0.968	6257	4860	5004
E85635	0.835	0.941	0.957	6258	4609	4853
E86610	0.835	1.028	1.038	6259	5615	5731
G84040	0.834	1.037	1.021	6260	5693	5572
C82005	0.834	1.159	1.124	6261	6698	6414
G81094	0.834	0.981	1.041	6262	5118	5762
N81013	0.834	1.067	1.011	6263	5983	5459
G84018	0.834	0.991	0.966	6264	5251	4990
D82006	0.834	1.127	1.077	6265	6507	6079
C81068	0.833	1.139	1.096	6266	6593	6229
F81197	0.833	1.067	1.068	6267	5996	6018
J82089	0.833	1.092	1.069	6268	6251	6027
G82042	0.833	1.087	1.044	6269	6190	5797
M81043	0.833	1.164	1.125	6270	6725	6430
H83037	0.833	0.999	0.989	6271	5345	5263
L85053	0.833	1.176	1.132	6272	6790	6489
J82218	0.832	0.997	0.971	6273	5326	5068
F83666	0.832	1.053	1.074	6274	5854	6070
F84717	0.832	0.984	0.974	6275	5171	5103
H85037	0.832	1.007	0.948	6276	5463	4775
F82007	0.832	1.053	1.020	6277	5875	5591
L81125	0.831	1.027	1.030	6278	5635	5687
E84012	0.831	0.999	1.002	6279	5365	5395
M85759	0.831	1.227	1.281	6280	7007	7098
B83604	0.831	1.189	1.276	6281	6877	7088
F83682	0.831	1.014	1.021	6282	5531	5600
C87622	0.831	0.971	1.065	6283	5036	6007
L82049	0.831	1.229	1.184	6284	7018	6768
J82163	0.831	1.113	1.083	6285	6428	6142
E82131	0.831	1.092	1.075	6286	6270	6086
G85016	0.831	1.003	1.018	6287	5420	5570
P85607	0.831	1.222	1.325	6288	6992	7176
E87047	0.830	0.979	1.019	6289	5137	5585
B81085	0.830	1.112	1.189	6290	6423	6801
H82100	0.830	1.102	1.091	6291	6344	6206
L83010	0.830	1.102	1.057	6292	6339	5951

L82008	0.830	1.133	1.100	6293	6571	6273
E81017	0.830	1.128	1.107	6294	6537	6329
C87023	0.830	1.411	1.492	6295	7302	7328
P81769	0.830	1.068	1.123	6296	6051	6438
M81061	0.830	1.117	1.078	6297	6470	6119
E86001	0.830	1.048	1.001	6298	5836	5408
F83008	0.830	1.100	1.096	6299	6332	6255
J81646	0.829	1.097	1.052	6300	6313	5910
F81123	0.829	1.101	1.039	6301	6340	5783
K81063	0.829	1.021	1.006	6302	5606	5452
L85040	0.829	1.093	1.089	6303	6291	6199
D81070	0.829	0.958	0.921	6304	4921	4504
Y01066	0.829	1.051	1.041	6305	5881	5817
K84032	0.828	1.045	1.021	6306	5815	5629
F86705	0.828	0.997	1.061	6307	5372	5999
H85045	0.828	1.012	0.963	6308	5540	5021
A83635	0.828	1.174	1.149	6309	6816	6593
F81070	0.828	1.141	1.078	6310	6637	6136
C81071	0.828	1.107	1.077	6311	6413	6124
F84621	0.828	1.046	1.112	6312	5835	6377
E87649	0.828	0.972	1.001	6313	5077	5426
E84693	0.828	1.046	1.012	6314	5837	5541
L83102	0.827	1.142	1.112	6315	6648	6382
C81040	0.827	1.035	1.032	6316	5747	5734
G85001	0.827	1.078	1.076	6317	6164	6117
J82201	0.827	1.058	1.046	6318	5965	5875
H81103	0.827	1.033	0.982	6319	5730	5247
J83633	0.827	1.138	1.141	6320	6620	6556
H84060	0.827	0.994	0.975	6321	5360	5164
E87006	0.827	1.019	1.023	6322	5605	5668
B86675	0.827	1.290	1.374	6323	7177	7242
C84650	0.826	1.103	1.170	6324	6386	6723
E87706	0.826	1.103	1.149	6325	6387	6608
B86029	0.826	1.109	1.115	6326	6431	6407
Y02936	0.826	1.081	1.151	6327	6206	6620
E85030	0.826	1.005	1.011	6328	5500	5534
E87037	0.826	0.957	0.954	6329	4942	4926
E81075	0.825	1.079	1.092	6330	6186	6259
G83002	0.825	1.102	1.063	6331	6388	6035
F83615	0.825	0.942	0.961	6332	4752	5025
P81112	0.825	1.231	1.206	6333	7042	6890
G84621	0.825	1.030	1.038	6334	5722	5825
P84635	0.825	1.084	1.171	6335	6255	6739
H83042	0.824	1.036	1.081	6336	5777	6181
H84039	0.824	0.964	0.924	6337	5034	4587
G82635	0.823	1.138	1.175	6338	6647	6760
A83055	0.823	1.082	1.085	6339	6246	6214
G83012	0.823	1.047	1.003	6340	5886	5490
D82008	0.823	1.134	1.150	6341	6613	6628
M85716	0.823	1.038	1.090	6342	5801	6256

E82006	0.823	1.095	1.059	6343	6342	6030
H83020	0.823	1.106	1.108	6344	6437	6386
G81021	0.823	1.039	1.000	6345	5808	5460
E85129	0.823	1.041	1.064	6346	5840	6061
K81002	0.822	1.053	1.039	6347	5957	5852
F84012	0.822	1.054	1.041	6348	5975	5869
J81029	0.822	1.093	1.053	6349	6333	5991
M81093	0.822	1.211	1.168	6350	6987	6740
M85082	0.821	1.073	1.082	6351	6178	6209
M83121	0.821	1.067	0.999	6352	6113	5475
P81045	0.821	1.033	1.034	6353	5784	5819
N81075	0.820	1.014	0.983	6354	5619	5328
E85059	0.820	0.989	0.973	6355	5378	5215
D81616	0.820	1.110	1.165	6356	6483	6733
G81056	0.820	1.049	1.056	6357	5944	6022
P81668	0.820	1.324	1.319	6358	7239	7186
Y00020	0.820	1.069	1.131	6359	6149	6537
M82048	0.820	1.078	1.064	6360	6245	6084
G81009	0.820	1.015	1.027	6361	5644	5761
F84060	0.820	0.985	1.061	6362	5335	6059
B82016	0.819	1.118	1.094	6363	6540	6317
L83077	0.819	1.130	1.060	6364	6614	6053
E83050	0.819	0.976	0.944	6365	5248	4888
K84051	0.819	1.269	1.198	6366	7155	6880
E84003	0.818	1.050	1.014	6367	5977	5664
F84710	0.818	1.029	0.999	6368	5776	5505
F81206	0.818	1.174	1.145	6369	6875	6635
K81057	0.817	1.034	1.012	6370	5822	5648
F84018	0.817	1.030	1.033	6371	5786	5844
E85110	0.817	1.009	1.082	6372	5607	6244
C81627	0.817	1.142	1.114	6373	6702	6468
L82001	0.816	1.065	1.051	6374	6151	6017
G83631	0.816	1.056	1.074	6375	6065	6188
G85681	0.816	0.968	1.021	6376	5173	5736
F86700	0.816	1.207	1.243	6377	6994	7036
F85008	0.816	0.992	0.974	6378	5460	5279
H81085	0.816	0.962	0.922	6379	5111	4659
N83041	0.816	1.207	1.206	6380	6993	6920
J83056	0.815	1.031	0.993	6381	5804	5458
F85053	0.815	0.986	0.968	6382	5402	5217
G85644	0.815	1.040	1.031	6383	5905	5854
J83020	0.815	1.077	1.039	6384	6276	5917
M88019	0.815	1.018	1.095	6385	5705	6350
F84033	0.815	1.008	0.998	6386	5625	5526
H85049	0.814	0.933	0.908	6387	4777	4507
G85708	0.814	0.990	1.029	6388	5455	5833
M83733	0.814	1.108	1.103	6389	6509	6410
E85004	0.814	1.087	1.044	6390	6349	5977
C82634	0.814	1.058	1.116	6391	6109	6499
E84084	0.814	1.116	1.153	6392	6567	6697

F83671	0.813	1.035	1.092	6393	5865	6337
G84604	0.813	0.972	1.002	6394	5258	5587
L81047	0.813	1.124	1.090	6395	6618	6323
F83039	0.813	1.090	1.127	6396	6389	6559
L81057	0.813	1.112	1.099	6397	6543	6391
Y01050	0.813	1.209	1.211	6398	7008	6946
C81064	0.813	1.092	1.070	6399	6415	6182
L81045	0.813	1.103	1.073	6400	6486	6208
F82012	0.812	1.104	1.097	6401	6496	6379
F84093	0.812	0.982	0.936	6402	5384	4868
K82064	0.812	0.984	0.957	6403	5416	5139
B82071	0.812	1.052	1.029	6404	6068	5858
C81072	0.811	1.090	1.078	6405	6411	6257
F84044	0.811	1.048	1.052	6406	6030	6052
K84019	0.811	1.069	1.013	6407	6243	5722
C81019	0.811	1.142	1.151	6408	6722	6704
D83615	0.811	1.195	1.266	6409	6970	7112
H83622	0.810	0.990	1.014	6410	5506	5729
F84685	0.810	1.013	1.102	6411	5699	6426
H83623	0.810	0.975	1.033	6412	5328	5905
H82002	0.810	1.080	1.078	6413	6329	6266
Y00522	0.809	1.038	1.045	6414	5943	6021
G82219	0.809	1.185	1.156	6415	6952	6744
M86622	0.809	0.985	0.994	6416	5474	5551
F81173	0.809	1.184	1.147	6417	6950	6695
F84054	0.808	1.144	1.130	6418	6744	6597
L83042	0.808	1.089	1.030	6419	6422	5896
E82113	0.808	1.000	0.967	6420	5602	5283
D82012	0.808	1.182	1.190	6421	6940	6897
H82022	0.808	1.034	0.982	6422	5911	5429
F84724	0.808	0.986	0.986	6423	5490	5468
A86020	0.808	0.987	1.051	6424	5499	6073
M87028	0.808	1.135	1.105	6425	6711	6467
F85065	0.807	1.062	1.130	6426	6198	6605
C81048	0.807	1.071	1.054	6427	6286	6107
B83641	0.807	1.153	1.168	6428	6811	6809
F81098	0.807	1.092	1.050	6429	6447	6078
Y00057	0.807	0.940	0.973	6430	4959	5355
K82045	0.807	1.075	1.038	6431	6316	5986
M88043	0.807	1.086	1.168	6432	6409	6810
M87602	0.806	1.118	1.091	6433	6621	6383
G81001	0.806	1.144	1.156	6434	6761	6754
J81033	0.806	1.024	1.013	6435	5827	5756
H85101	0.806	1.013	0.976	6436	5738	5389
M85600	0.806	1.180	1.187	6437	6943	6886
F81086	0.806	1.139	1.124	6438	6735	6577
D83043	0.806	1.192	1.180	6439	6980	6859
E86009	0.805	1.108	1.059	6440	6574	6151
J81624	0.805	1.067	1.138	6441	6269	6667
M82013	0.805	1.060	1.077	6442	6208	6303

G82014	0.805	1.079	1.081	6443	6360	6321
F83022	0.805	1.026	1.007	6444	5867	5720
E85055	0.805	1.012	1.015	6445	5736	5792
F81028	0.805	1.060	1.057	6446	6211	6143
H81025	0.804	1.052	1.029	6447	6137	5926
H84025	0.804	1.083	1.033	6448	6407	5965
F85046	0.804	1.004	1.071	6449	5672	6264
G85053	0.804	1.015	1.006	6450	5771	5714
E87008	0.804	1.069	1.032	6451	6301	5967
N81100	0.804	1.060	1.031	6452	6223	5955
Y00454	0.803	0.997	1.050	6453	5624	6111
G85118	0.802	1.073	1.132	6454	6330	6644
L84012	0.802	1.087	1.065	6455	6449	6231
L84004	0.802	1.163	1.092	6456	6896	6417
B85006	0.802	1.095	1.093	6457	6506	6425
J82028	0.802	1.048	1.063	6458	6116	6213
B86031	0.801	1.076	1.144	6459	6367	6720
K83621	0.800	1.008	1.043	6460	5740	6075
B86005	0.800	1.202	1.237	6461	7032	7069
M83013	0.800	1.094	1.008	6462	6512	5765
G85114	0.800	1.078	1.080	6463	6406	6353
L81118	0.799	1.087	1.072	6464	6462	6302
K83009	0.799	1.046	1.049	6465	6132	6134
C82042	0.798	1.114	1.082	6466	6650	6374
F81094	0.798	1.111	1.103	6467	6639	6515
P88006	0.798	1.151	1.143	6468	6845	6731
P83008	0.798	1.165	1.195	6469	6914	6943
P81722	0.798	1.376	1.501	6470	7298	7340
M86610	0.798	1.039	1.113	6471	6080	6561
C82050	0.798	1.139	1.100	6472	6784	6500
E82031	0.797	1.044	1.023	6473	6118	5941
M83090	0.797	1.276	1.358	6474	7201	7258
F84080	0.797	1.060	1.128	6475	6282	6656
F83064	0.797	1.129	1.109	6476	6732	6547
E84624	0.796	1.079	1.100	6477	6432	6507
E85617	0.796	0.935	0.999	6478	5015	5726
M87618	0.796	1.073	1.163	6479	6403	6837
B82057	0.796	1.096	1.076	6480	6556	6349
M92647	0.796	1.160	1.216	6481	6906	7019
J82628	0.796	1.043	0.988	6482	6125	5627
E85108	0.796	1.034	1.012	6483	6050	5851
F86005	0.795	1.067	1.065	6484	6347	6282
E84080	0.795	0.963	0.925	6485	5367	4950
E82058	0.795	0.954	0.948	6486	5264	5219
B86651	0.794	1.096	1.160	6487	6564	6830
G83067	0.794	1.027	1.002	6488	5972	5766
E87065	0.794	1.117	1.132	6489	6683	6689
C84095	0.794	1.147	1.131	6490	6849	6692
Y02713	0.794	1.191	1.308	6491	7013	7205
E85600	0.794	1.046	1.038	6492	6179	6088

E84070	0.794	0.970	0.937	6493	5469	5125
J82639	0.793	1.095	1.058	6494	6572	6247
K83035	0.793	1.078	1.084	6495	6454	6423
F86074	0.793	1.000	1.035	6496	5743	6064
C84069	0.792	1.070	1.071	6497	6399	6344
E87011	0.792	1.083	1.069	6498	6492	6325
E84601	0.792	0.983	0.975	6499	5593	5523
L83052	0.792	1.074	1.068	6500	6430	6322
C81059	0.792	1.234	1.186	6501	7134	6930
H83609	0.792	1.044	1.068	6502	6181	6324
B82091	0.792	1.085	1.070	6503	6508	6338
L81010	0.792	1.173	1.140	6504	6960	6751
K84036	0.792	1.042	0.991	6505	6161	5695
C82651	0.792	1.150	1.156	6506	6883	6823
E84059	0.791	0.975	0.929	6507	5535	5056
H83007	0.791	1.037	1.000	6508	6115	5778
G81007	0.791	1.043	1.011	6509	6173	5879
K81080	0.791	1.002	0.984	6510	5772	5640
L81600	0.790	1.062	1.034	6511	6346	6082
H84006	0.790	1.027	0.977	6512	6035	5573
G82733	0.790	1.020	1.002	6513	5953	5815
M85014	0.790	1.136	1.186	6514	6815	6940
A82026	0.789	1.127	1.056	6515	6758	6252
C82671	0.789	1.007	1.036	6516	5831	6110
N83015	0.789	1.196	1.255	6517	7050	7140
E85019	0.789	1.004	0.974	6518	5806	5547
E87746	0.788	0.955	0.988	6519	5354	5696
M92042	0.788	1.091	1.156	6520	6580	6840
N85047	0.788	1.143	1.113	6521	6868	6615
F86625	0.788	1.039	1.121	6522	6176	6668
E83053	0.787	1.025	0.969	6523	6044	5506
J82130	0.787	1.010	0.973	6524	5887	5566
G85096	0.787	0.964	1.010	6525	5478	5916
P87659	0.786	1.121	1.220	6526	6742	7061
E85122	0.786	0.949	0.943	6527	5312	5270
E84076	0.786	1.063	1.028	6528	6391	6060
E83008	0.786	0.987	0.942	6529	5677	5256
L81033	0.786	1.131	1.117	6530	6806	6652
J82006	0.786	1.130	1.139	6531	6799	6771
P91633	0.786	1.139	1.150	6532	6846	6820
F84021	0.786	1.007	1.014	6533	5869	5966
P87651	0.786	0.861	0.904	6534	4193	4795
L83066	0.786	1.067	1.015	6535	6421	5972
F84119	0.785	1.047	1.056	6536	6267	6283
G84630	0.785	1.145	1.149	6537	6887	6818
G84628	0.785	1.072	1.074	6538	6460	6406
L81656	0.785	1.017	1.059	6539	5968	6314
N82082	0.785	1.230	1.305	6540	7138	7212
E87003	0.785	1.049	1.102	6541	6288	6570
M81012	0.785	1.076	1.034	6542	6490	6122

F83025	0.785	1.070	1.009	6543	6450	5919
E85109	0.785	0.965	1.030	6544	5513	6094
P81758	0.785	1.154	1.197	6545	6928	7001
K81050	0.784	1.045	1.047	6546	6268	6226
Y03402	0.783	1.010	1.002	6547	5917	5865
E84663	0.783	1.040	1.042	6548	6226	6194
E85636	0.783	1.009	1.028	6549	5910	6096
E81611	0.783	1.033	1.039	6550	6163	6169
L85064	0.782	1.121	1.119	6551	6762	6686
F81207	0.782	1.069	1.122	6552	6461	6706
G85051	0.782	0.946	0.944	6553	5320	5319
F81133	0.782	1.136	1.118	6554	6854	6683
B86095	0.782	1.220	1.298	6555	7128	7207
M86048	0.782	1.054	1.044	6556	6343	6219
M81018	0.781	1.121	1.067	6557	6772	6392
G83654	0.781	1.092	1.138	6558	6622	6790
Y01132	0.781	0.949	0.909	6559	5370	4925
P84034	0.781	1.015	1.038	6560	5995	6180
H82063	0.780	1.134	1.123	6561	6853	6717
K84050	0.780	1.057	1.021	6562	6393	6051
M85671	0.780	1.189	1.239	6563	7052	7125
M85025	0.780	1.001	1.019	6564	5866	6036
C84028	0.780	1.028	0.971	6565	6142	5617
F84105	0.780	1.021	1.048	6566	6077	6269
E86022	0.780	1.013	0.970	6567	5986	5622
L81012	0.780	1.016	1.029	6568	6021	6125
G85048	0.779	1.001	1.026	6569	5872	6106
E82060	0.779	0.995	0.974	6570	5811	5650
E84626	0.779	1.029	1.037	6571	6155	6191
B82088	0.779	1.188	1.179	6572	7053	6953
E86041	0.779	1.034	1.025	6573	6207	6101
L85029	0.778	1.133	1.088	6574	6862	6532
H82005	0.778	1.081	1.069	6575	6579	6419
P87648	0.777	0.872	0.912	6576	4460	5013
E85016	0.777	1.043	1.050	6577	6304	6308
L85037	0.777	1.121	1.062	6578	6795	6380
L84077	0.777	1.117	1.106	6579	6773	6641
H82044	0.777	1.053	1.041	6580	6371	6243
G85061	0.777	1.087	1.073	6581	6610	6464
J82605	0.777	0.969	1.019	6582	5610	6069
C81660	0.777	1.228	1.223	6583	7157	7092
G83021	0.777	1.024	0.997	6584	6135	5883
N81623	0.776	1.064	1.073	6585	6477	6473
N82079	0.776	1.011	1.129	6586	6003	6767
K81610	0.776	1.016	0.995	6587	6075	5880
K81074	0.776	0.936	0.907	6588	5276	4970
G85083	0.775	1.014	1.090	6589	6049	6560
E84075	0.775	0.996	0.950	6590	5870	5436
H84042	0.775	1.051	1.000	6591	6376	5929
E84676	0.775	1.049	1.036	6592	6355	6207

L81078	0.775	1.019	1.026	6593	6100	6140
P81157	0.775	1.121	1.059	6594	6812	6375
E85014	0.775	0.962	0.944	6595	5571	5386
B82083	0.774	1.086	1.065	6596	6627	6418
B86623	0.774	0.929	0.999	6597	5216	5925
F83658	0.774	1.065	1.107	6598	6493	6664
P81208	0.774	1.209	1.194	6599	7111	7016
M82008	0.774	1.081	1.061	6600	6597	6396
P84669	0.774	1.017	1.067	6601	6095	6442
M89030	0.774	1.000	0.949	6602	5922	5442
E82053	0.773	1.048	1.030	6603	6363	6174
F86689	0.773	1.001	1.091	6604	5936	6578
L84003	0.773	0.993	0.997	6605	5851	5928
K83607	0.773	1.159	1.251	6606	6979	7166
K81103	0.772	1.056	1.054	6607	6442	6360
K84082	0.772	1.026	0.988	6608	6192	5848
K84048	0.772	1.073	1.041	6609	6563	6270
L81056	0.772	1.035	1.004	6610	6283	5997
J82143	0.772	1.011	0.992	6611	6063	5889
E85028	0.772	0.998	0.991	6612	5928	5877
N82664	0.771	1.107	1.203	6613	6748	7053
P89010	0.771	1.167	1.162	6614	7005	6921
G81028	0.771	1.027	1.058	6615	6215	6397
Y00999	0.771	0.991	0.986	6616	5853	5838
F86058	0.771	0.981	0.981	6617	5759	5788
P83025	0.770	1.002	0.988	6618	5967	5859
M83608	0.770	1.074	1.039	6619	6586	6271
P91029	0.770	1.195	1.179	6620	7090	6990
F85049	0.769	1.008	1.052	6621	6047	6371
C84090	0.769	1.039	1.068	6622	6328	6483
F82619	0.769	0.977	1.029	6623	5750	6201
K84079	0.769	1.001	0.965	6624	5978	5671
B86654	0.769	1.021	1.053	6625	6185	6378
M91020	0.768	1.093	1.148	6626	6693	6883
F85011	0.768	1.234	1.306	6627	7181	7234
L83648	0.768	1.005	1.031	6628	6019	6227
G81634	0.768	1.128	1.089	6629	6892	6596
E85032	0.768	0.992	1.021	6630	5894	6148
L85034	0.768	1.055	1.016	6631	6466	6120
P88633	0.767	1.153	1.175	6632	6976	6983
H81069	0.767	1.014	0.969	6633	6131	5715
H81084	0.767	1.003	0.954	6634	6011	5574
K84073	0.767	1.035	1.020	6635	6318	6150
H85618	0.766	1.111	1.114	6636	6800	6748
M82007	0.766	1.241	1.201	6637	7196	7063
E87762	0.766	1.065	1.059	6638	6547	6433
E84026	0.766	0.978	0.998	6639	5782	5996
P92620	0.765	1.148	1.237	6640	6964	7151
F82028	0.765	1.065	1.020	6641	6555	6161
D81003	0.765	1.050	1.004	6642	6453	6042

E85112	0.765	1.022	0.989	6643	6230	5924
K81028	0.765	0.973	0.939	6644	5745	5427
Y00312	0.765	1.091	1.085	6645	6710	6588
H84057	0.765	0.971	0.999	6646	5729	6012
H85111	0.764	1.021	0.976	6647	6227	5805
P87649	0.764	1.093	1.224	6648	6718	7129
F83015	0.764	1.087	1.091	6649	6690	6637
Y00403	0.763	0.969	1.061	6650	5723	6478
Y02520	0.763	1.018	1.053	6651	6205	6416
P81213	0.763	1.000	0.993	6652	6032	5981
J82216	0.763	1.034	0.995	6653	6341	5998
E84002	0.762	1.000	0.987	6654	6038	5927
F83060	0.761	1.062	1.157	6655	6558	6938
F83681	0.761	1.110	1.131	6656	6828	6845
G83024	0.761	1.005	0.966	6657	6105	5735
H84059	0.761	1.012	0.979	6658	6169	5863
F84041	0.761	1.009	1.079	6659	6141	6575
H85680	0.761	0.948	0.897	6660	5561	5034
F86082	0.761	1.096	1.121	6661	6743	6805
L84039	0.761	1.107	1.091	6662	6808	6647
G81042	0.761	1.024	1.077	6663	6285	6567
F84008	0.761	1.040	1.087	6664	6412	6622
K81068	0.760	0.980	0.932	6665	5852	5415
K81640	0.760	0.924	0.984	6666	5321	5918
G85706	0.760	0.998	1.052	6667	6048	6431
C82084	0.759	1.043	1.097	6668	6443	6696
F82005	0.759	1.119	1.124	6669	6889	6832
K84046	0.758	1.062	1.018	6670	6587	6196
E82014	0.758	0.994	0.969	6671	6015	5798
E83021	0.758	1.015	0.963	6672	6231	5740
M85669	0.758	1.060	1.115	6673	6575	6795
J83032	0.757	1.085	1.065	6674	6714	6526
H81041	0.757	1.042	1.033	6675	6446	6319
C82620	0.757	0.983	1.020	6676	5913	6224
M85041	0.757	0.941	0.973	6677	5542	5843
E85123	0.757	0.929	0.957	6678	5418	5705
Y01090	0.757	0.939	0.954	6679	5532	5673
E87002	0.756	0.991	0.957	6680	6002	5706
C82070	0.756	1.202	1.166	6681	7143	6991
J82183	0.756	1.097	1.156	6682	6785	6960
J82075	0.756	1.089	1.048	6683	6739	6435
H81130	0.755	1.010	0.974	6684	6204	5864
E84067	0.755	0.953	0.969	6685	5655	5824
M88629	0.755	1.072	1.179	6686	6658	7025
G81095	0.755	1.137	1.201	6687	6962	7091
E85683	0.755	0.987	1.004	6688	5973	6126
G81018	0.755	1.033	1.068	6689	6408	6550
P81757	0.754	0.993	1.052	6690	6060	6479
H81643	0.754	0.979	0.951	6691	5902	5674
E81054	0.753	1.066	1.154	6692	6630	6956

G85136	0.753	0.955	0.954	6693	5682	5704
G85002	0.753	0.938	0.974	6694	5548	5884
F83004	0.753	1.093	1.186	6695	6780	7058
Y00189	0.753	1.096	1.096	6696	6796	6722
F81183	0.753	1.066	1.024	6697	6645	6293
H83049	0.752	1.120	1.101	6698	6919	6750
M85074	0.752	1.048	1.033	6699	6521	6361
G85022	0.752	1.054	1.050	6700	6569	6485
M87605	0.751	1.098	1.159	6701	6819	6980
H84619	0.751	0.974	0.951	6702	5879	5691
M86613	0.751	1.078	1.106	6703	6713	6781
L81075	0.751	0.971	0.979	6704	5855	5952
E83649	0.751	0.933	0.900	6705	5522	5173
J82019	0.751	1.026	1.005	6706	6366	6155
H85659	0.751	0.972	1.032	6707	5860	6356
F83652	0.750	1.004	1.012	6708	6187	6218
G85673	0.750	0.992	1.012	6709	6085	6221
K84617	0.750	1.027	1.020	6710	6392	6275
Y03664	0.749	0.932	0.930	6711	5523	5500
K84025	0.749	1.033	0.982	6712	6440	6000
A88015	0.749	1.329	1.314	6713	7301	7262
N81607	0.749	1.336	1.319	6714	7307	7268
P87028	0.748	1.171	1.224	6715	7085	7160
Y02121	0.747	1.228	1.170	6716	7202	7022
Y00560	0.747	1.068	1.049	6717	6677	6502
J81078	0.747	1.051	1.024	6718	6588	6326
Y02928	0.747	0.974	0.974	6719	5927	5954
F83012	0.746	1.064	1.098	6720	6661	6765
C81108	0.746	1.100	1.051	6721	6859	6522
F84003	0.745	1.089	1.124	6722	6797	6879
F83020	0.745	1.020	1.004	6723	6364	6193
E84681	0.745	1.006	1.013	6724	6264	6262
E83039	0.745	0.883	0.843	6725	5010	4568
M89608	0.744	1.024	0.962	6726	6414	5860
M92028	0.744	1.050	1.082	6727	6594	6693
E83045	0.744	1.055	1.034	6728	6615	6420
E87034	0.744	1.000	0.976	6729	6218	6002
E82094	0.744	1.001	0.990	6730	6238	6103
E85713	0.743	1.039	1.063	6731	6517	6587
E84638	0.743	0.958	0.922	6732	5795	5487
M88626	0.743	1.175	1.235	6733	7103	7174
L84014	0.743	1.089	1.037	6734	6814	6448
F84077	0.743	1.006	0.989	6735	6278	6100
H83624	0.742	0.985	1.024	6736	6090	6363
P84005	0.742	0.921	1.003	6737	5492	6205
L83134	0.742	1.168	1.166	6738	7092	7023
E85020	0.742	0.978	1.021	6739	6010	6339
F84062	0.742	1.152	1.094	6740	7054	6761
M91024	0.742	1.260	1.328	6741	7253	7286
Y00726	0.742	1.262	1.351	6742	7255	7300

H81073	0.742	0.978	0.944	6743	6020	5719
E84039	0.742	1.045	0.980	6744	6584	6041
F85066	0.741	1.005	0.996	6745	6284	6157
F83006	0.741	1.076	1.035	6746	6746	6447
N83025	0.741	1.377	1.449	6747	7336	7343
C84624	0.740	1.130	1.149	6748	6989	6986
H85001	0.740	0.918	0.892	6749	5488	5213
E85685	0.740	0.961	0.962	6750	5859	5908
C86022	0.740	1.158	1.153	6751	7070	7004
F84118	0.739	0.968	0.978	6752	5947	6039
H81082	0.739	1.079	1.033	6753	6778	6452
M89016	0.739	1.109	1.053	6754	6930	6553
E84030	0.739	1.028	1.035	6755	6482	6463
J82171	0.739	1.092	1.097	6756	6850	6798
Y01812	0.739	1.144	1.073	6757	7040	6672
P89018	0.738	1.285	1.318	6758	7275	7282
J83033	0.738	1.151	1.121	6759	7060	6898
G81047	0.738	0.991	1.052	6760	6184	6554
H82039	0.738	1.142	1.092	6761	7036	6776
M85145	0.737	1.076	1.063	6762	6764	6626
E83049	0.737	1.009	0.952	6763	6336	5837
M89028	0.737	1.013	0.966	6764	6378	5969
G85109	0.737	0.989	1.027	6765	6177	6413
F84079	0.737	1.000	0.976	6766	6279	6044
N81031	0.737	1.096	1.038	6767	6886	6497
M85110	0.737	1.060	1.073	6768	6687	6685
P85620	0.736	1.173	1.178	6769	7117	7083
J82076	0.736	1.142	1.160	6770	7044	7024
Y00471	0.736	1.061	1.100	6771	6696	6825
L83139	0.736	1.215	1.194	6772	7200	7118
K82621	0.735	1.053	1.015	6773	6663	6347
J82663	0.734	0.947	0.998	6774	5787	6240
L81070	0.733	1.051	1.043	6775	6665	6531
F83680	0.733	1.063	1.138	6776	6719	6970
F83045	0.733	1.031	1.047	6777	6533	6552
F83624	0.733	0.955	0.995	6778	5878	6220
G85030	0.733	0.981	1.036	6779	6145	6510
F84052	0.733	1.087	1.078	6780	6864	6734
H81030	0.732	1.082	1.049	6781	6831	6572
F84719	0.732	1.012	1.077	6782	6419	6735
E87754	0.732	0.927	0.932	6783	5639	5703
F84682	0.731	1.007	0.965	6784	6372	6004
G85132	0.731	1.027	1.056	6785	6519	6619
P84639	0.731	1.086	1.162	6786	6861	7044
L81108	0.731	1.005	1.030	6787	6359	6490
Y00058	0.731	0.999	0.979	6788	6319	6118
G85716	0.730	1.141	1.188	6789	7055	7122
E82023	0.730	1.022	0.986	6790	6498	6170
H83611	0.730	1.027	1.078	6791	6532	6749
G85695	0.729	0.975	1.056	6792	6111	6633

D81620	0.729	1.062	1.152	6793	6736	7021
M85701	0.728	1.081	1.113	6794	6847	6903
G85112	0.728	0.966	0.992	6795	6042	6236
Y03063	0.728	0.970	1.000	6796	6091	6306
G82665	0.728	1.275	1.247	6797	7278	7211
B86043	0.727	1.174	1.163	6798	7145	7064
J82194	0.727	1.133	1.153	6799	7039	7034
L83082	0.727	1.066	1.011	6800	6765	6373
F85069	0.727	0.973	1.019	6801	6117	6427
F83660	0.727	1.019	1.017	6802	6504	6422
D81056	0.726	0.964	0.921	6803	6045	5649
D82069	0.726	1.066	1.135	6804	6774	6987
F84096	0.726	1.042	1.038	6805	6652	6542
G81090	0.726	1.083	1.129	6806	6878	6964
J83636	0.726	1.062	1.034	6807	6753	6527
E84074	0.726	1.020	0.991	6808	6514	6245
P89613	0.725	1.095	1.135	6809	6925	6988
D81002	0.725	0.983	0.941	6810	6234	5842
G85073	0.725	1.023	1.061	6811	6536	6681
C85028	0.725	1.118	1.119	6812	6999	6933
E83026	0.725	0.972	0.955	6813	6136	5983
G85698	0.724	1.025	1.061	6814	6554	6684
B82077	0.724	1.108	1.106	6815	6968	6892
P84039	0.723	1.107	1.151	6816	6969	7038
J82102	0.723	1.114	1.111	6817	6991	6917
L83127	0.722	1.125	1.099	6818	7033	6869
H83011	0.722	0.959	0.994	6819	6027	6290
K83050	0.722	1.032	1.102	6820	6609	6882
E82068	0.722	1.013	0.984	6821	6497	6215
C84044	0.722	1.006	1.017	6822	6444	6454
E83046	0.722	1.136	1.088	6823	7063	6833
B81100	0.721	1.088	1.104	6824	6916	6899
G81054	0.721	1.073	1.128	6825	6842	6975
C82032	0.721	1.140	1.122	6826	7072	6959
E85658	0.721	0.941	0.981	6827	5857	6200
Y02636	0.721	0.959	1.043	6828	6039	6600
G82820	0.720	1.205	1.289	6829	7211	7267
K81651	0.720	1.009	1.037	6830	6481	6573
E84028	0.719	1.020	1.040	6831	6560	6592
L81007	0.719	1.035	1.052	6832	6649	6662
L84030	0.719	1.070	1.060	6833	6835	6707
B86074	0.719	1.062	1.076	6834	6792	6791
Y00561	0.719	1.045	1.063	6835	6707	6726
Y00328	0.719	1.016	1.015	6836	6526	6459
P88043	0.719	1.469	1.488	6837	7358	7354
H84027	0.719	0.951	0.908	6838	5979	5598
J82098	0.718	1.081	1.051	6839	6900	6669
F86060	0.718	1.012	0.943	6840	6511	5937
E87016	0.718	0.949	0.930	6841	5961	5802
F85642	0.717	1.006	1.012	6842	6474	6453

Y01206	0.717	0.952	0.910	6843	6001	5636
E86626	0.717	1.076	1.051	6844	6884	6670
D81615	0.717	1.035	1.082	6845	6666	6829
B81617	0.717	1.059	1.049	6846	6788	6660
F85019	0.717	1.020	1.034	6847	6577	6576
F86087	0.717	0.975	0.941	6848	6242	5922
L81091	0.716	0.963	0.964	6849	6119	6112
H84012	0.716	0.970	0.929	6850	6195	5820
E83007	0.716	1.071	0.997	6851	6865	6355
F84747	0.716	0.909	0.890	6852	5612	5432
F84051	0.716	1.039	1.076	6853	6688	6803
L83049	0.716	1.122	1.069	6854	7043	6775
M86004	0.715	0.952	0.944	6855	6028	5964
Y00612	0.715	0.950	0.991	6856	6000	6318
G81045	0.715	0.979	0.958	6857	6293	6076
M85782	0.715	0.889	0.949	6858	5431	6009
M88610	0.715	1.087	1.157	6859	6936	7084
Y03035	0.714	1.018	1.013	6860	6582	6482
F82670	0.714	1.025	1.039	6861	6617	6614
E81615	0.714	1.064	1.074	6862	6833	6804
P84616	0.714	1.031	1.119	6863	6659	6971
F82040	0.714	1.019	1.087	6864	6589	6856
L84049	0.714	1.074	1.074	6865	6891	6807
K84012	0.713	1.034	0.986	6866	6675	6299
H85100	0.713	0.945	0.909	6867	5964	5652
F86073	0.713	0.989	1.018	6868	6382	6517
L81130	0.712	1.105	1.082	6869	7000	6847
Y02177	0.712	1.061	1.103	6870	6830	6924
M85072	0.712	1.078	1.098	6871	6911	6912
L84034	0.711	1.141	1.110	6872	7099	6949
E87755	0.711	0.945	0.991	6873	5998	6351
H85051	0.710	0.960	0.922	6874	6158	5804
C88068	0.710	0.996	1.003	6875	6452	6437
H82098	0.710	1.066	1.047	6876	6876	6687
E85740	0.710	0.967	1.035	6877	6232	6623
C81042	0.709	1.026	1.000	6878	6655	6421
F81100	0.709	1.070	1.061	6879	6897	6763
M85676	0.709	1.107	1.136	6880	7015	7035
F86062	0.709	1.025	1.041	6881	6654	6663
M92612	0.708	1.168	1.234	6882	7168	7218
G85085	0.708	0.995	0.971	6883	6463	6230
G85642	0.708	0.984	0.963	6884	6383	6162
H83051	0.707	0.994	1.033	6885	6465	6631
C81006	0.706	1.196	1.163	6886	7219	7116
F84734	0.705	0.953	0.986	6887	6140	6354
H84002	0.705	0.969	0.935	6888	6289	5976
E82603	0.705	1.092	1.084	6889	6981	6881
F84115	0.705	1.058	1.106	6890	6848	6962
H85088	0.705	0.966	0.999	6891	6271	6449
M83107	0.705	1.549	1.622	6892	7367	7370

L83012	0.704	1.125	1.103	6893	7078	6947
M87617	0.704	1.142	1.200	6894	7124	7179
E84031	0.704	1.059	1.004	6895	6866	6494
G85712	0.704	0.971	1.024	6896	6312	6590
E83656	0.704	1.005	1.044	6897	6565	6702
F83683	0.704	1.103	1.093	6898	7022	6918
E87681	0.704	0.947	1.002	6899	6094	6480
N83619	0.704	1.136	1.212	6900	7105	7196
E83658	0.704	0.957	0.976	6901	6193	6305
F84081	0.703	1.014	0.990	6902	6623	6398
G85095	0.702	1.051	1.025	6903	6824	6602
F83632	0.702	1.048	1.095	6904	6810	6928
E85715	0.702	1.003	1.021	6905	6559	6586
N81090	0.702	1.085	1.045	6906	6967	6727
M81007	0.701	1.086	1.056	6907	6973	6779
M85021	0.701	0.984	1.035	6908	6441	6677
G85084	0.701	1.010	1.074	6909	6605	6857
F83063	0.700	1.071	1.116	6910	6929	7010
F83032	0.700	1.013	1.016	6911	6641	6566
Y00316	0.699	0.984	0.970	6912	6448	6279
J81072	0.699	1.015	0.996	6913	6653	6472
F85024	0.698	1.022	1.044	6914	6692	6736
J82050	0.698	1.010	0.962	6915	6626	6241
F83010	0.697	1.017	1.073	6916	6673	6867
M92041	0.697	1.079	1.141	6917	6961	7087
G85038	0.697	1.010	1.038	6918	6640	6712
E87648	0.697	0.900	0.931	6919	5708	6008
C82060	0.697	1.058	1.104	6920	6898	6984
E85033	0.696	1.047	1.054	6921	6837	6792
C88079	0.696	1.053	1.090	6922	6874	6929
P85614	0.696	1.299	1.337	6923	7316	7316
M85058	0.696	1.022	0.989	6924	6708	6440
E87029	0.696	0.992	0.993	6925	6523	6471
K84054	0.695	1.080	1.037	6926	6971	6715
Y02476	0.695	0.901	0.901	6927	5728	5757
J82071	0.692	1.094	1.069	6928	7029	6872
F84013	0.691	0.936	0.927	6929	6107	6026
E87714	0.691	1.005	1.007	6930	6644	6565
F83048	0.691	0.987	0.989	6931	6520	6474
C81652	0.691	1.130	1.207	6932	7129	7206
G85100	0.691	1.012	1.053	6933	6676	6812
H81007	0.690	1.000	0.963	6934	6608	6304
E85630	0.690	0.979	0.988	6935	6480	6475
B81675	0.690	1.177	1.322	6936	7214	7314
M85060	0.690	1.152	1.114	6937	7175	7028
L82041	0.689	1.134	1.122	6938	7140	7060
F81176	0.689	1.178	1.267	6939	7216	7281
M85774	0.689	1.072	1.141	6940	6965	7104
H82057	0.689	1.058	1.042	6941	6924	6773
H85634	0.688	0.984	0.954	6942	6516	6253

F86698	0.687	1.055	1.086	6943	6920	6948
C81653	0.687	1.070	1.163	6944	6966	7161
D81005	0.687	0.917	0.887	6945	5956	5699
P87613	0.687	1.313	1.394	6946	7329	7342
K81100	0.687	0.946	0.982	6947	6257	6456
Y00589	0.686	1.072	1.026	6948	6978	6703
F84069	0.685	1.065	1.060	6949	6957	6860
M91017	0.685	1.206	1.191	6950	7256	7193
G82693	0.685	1.134	1.116	6951	7149	7039
M88647	0.685	1.052	1.116	6952	6912	7040
G85724	0.685	0.927	1.017	6953	6082	6661
G85054	0.685	1.074	1.057	6954	6985	6854
E85025	0.685	0.982	1.027	6955	6525	6716
E87682	0.684	0.959	1.011	6956	6361	6630
H84044	0.684	1.020	0.989	6957	6750	6520
F84706	0.683	0.982	1.013	6958	6539	6649
J82044	0.683	1.118	1.088	6959	7115	6967
M83723	0.683	1.191	1.179	6960	7243	7180
L81090	0.683	0.994	1.009	6961	6616	6625
G81070	0.683	1.019	1.033	6962	6749	6757
J83619	0.682	1.051	1.047	6963	6923	6826
F85014	0.682	0.962	0.961	6964	6416	6340
F81666	0.682	1.164	1.192	6965	7206	7203
E87050	0.682	1.030	1.083	6966	6825	6961
E87021	0.681	1.055	1.092	6967	6942	6994
C81009	0.680	1.178	1.134	6968	7232	7109
E87009	0.680	1.017	1.011	6969	6755	6659
Y00568	0.679	1.086	1.086	6970	7046	6978
L81071	0.679	0.976	0.966	6971	6522	6393
H83043	0.679	0.998	1.043	6972	6667	6822
F85063	0.679	0.957	0.957	6973	6405	6334
F85623	0.679	0.998	1.057	6974	6670	6875
F82671	0.678	1.052	1.084	6975	6944	6976
F83017	0.677	1.027	1.007	6976	6832	6645
F84117	0.677	1.015	1.058	6977	6768	6896
P91619	0.676	1.051	1.140	6978	6946	7134
B82021	0.676	1.073	1.051	6979	7012	6863
F84731	0.676	1.075	1.129	6980	7023	7107
M92014	0.675	1.076	1.146	6981	7027	7147
E87609	0.675	0.948	0.911	6982	6350	6025
K84076	0.675	1.007	0.990	6983	6728	6558
E87071	0.675	0.914	0.914	6984	6058	6045
G81038	0.675	0.989	1.009	6985	6646	6671
E83009	0.675	0.960	0.922	6986	6457	6109
H85078	0.674	1.044	1.071	6987	6927	6941
E86637	0.674	1.056	1.090	6988	6963	7008
L81081	0.674	0.947	0.957	6989	6352	6372
H85012	0.674	0.932	0.899	6990	6241	5931
G85010	0.673	1.027	1.114	6991	6856	7082
C84112	0.673	1.113	1.085	6992	7130	6998

P84072	0.672	0.997	1.019	6993	6705	6745
K84009	0.672	1.021	0.992	6994	6829	6589
E85693	0.671	0.948	0.931	6995	6390	6197
E83654	0.671	0.993	1.010	6996	6682	6701
D81013	0.668	0.967	0.936	6997	6541	6263
F84749	0.668	0.958	0.949	6998	6487	6364
F82027	0.668	1.046	1.104	6999	6955	7066
H85066	0.667	0.959	0.934	7000	6494	6260
A82654	0.667	1.158	1.191	7001	7222	7217
L82015	0.666	1.105	1.098	7002	7127	7050
F83003	0.666	0.965	0.953	7003	6546	6394
L83078	0.665	1.221	1.193	7004	7284	7223
J82081	0.665	0.962	0.960	7005	6531	6460
F82055	0.663	1.076	1.059	7006	7061	6939
F85669	0.663	0.982	1.007	7007	6668	6732
G82702	0.663	1.034	1.100	7008	6933	7072
F81192	0.662	1.194	1.161	7009	7260	7184
M83017	0.662	1.318	1.290	7010	7340	7313
M85010	0.661	1.093	1.098	7011	7102	7067
G83680	0.661	1.022	0.999	7012	6894	6691
C84080	0.661	1.065	1.039	7013	7038	6877
M85797	0.660	1.008	1.060	7014	6821	6954
B83043	0.660	1.035	1.045	7015	6949	6904
G81638	0.659	0.985	1.040	7016	6703	6889
F84601	0.659	1.030	1.059	7017	6932	6958
E84048	0.659	1.005	0.936	7018	6813	6328
E85680	0.659	0.987	0.961	7019	6716	6501
F83677	0.658	1.012	1.078	7020	6852	7014
E85071	0.658	1.116	1.153	7021	7167	7177
F81015	0.658	1.095	1.039	7022	7119	6888
F83042	0.657	0.983	1.024	7023	6704	6836
G85004	0.656	1.080	1.067	7024	7088	6993
E85748	0.655	0.896	0.976	7025	6078	6598
E82656	0.655	0.981	0.974	7026	6706	6584
G85633	0.655	1.047	1.067	7027	6996	7003
M85611	0.655	1.015	1.067	7028	6893	7000
M85085	0.654	0.993	1.052	7029	6767	6944
K81081	0.654	0.991	1.061	7030	6751	6977
H81038	0.654	0.942	0.920	7031	6467	6251
H83044	0.654	0.974	0.949	7032	6674	6455
G82662	0.652	0.981	0.969	7033	6717	6568
G82175	0.652	1.061	1.069	7034	7051	7012
J82629	0.651	1.107	1.092	7035	7165	7080
H81061	0.651	1.002	0.990	7036	6836	6699
L82007	0.651	1.132	1.114	7037	7204	7133
F85064	0.650	0.995	1.065	7038	6803	7006
Y00507	0.649	1.056	1.115	7039	7045	7137
G85130	0.649	1.019	1.004	7040	6926	6778
L81637	0.649	1.082	1.056	7041	7107	6981
H81647	0.649	0.948	0.907	7042	6542	6187

G81656	0.648	1.025	1.126	7043	6953	7165
C84104	0.648	0.986	1.064	7044	6759	7011
F84730	0.647	0.904	0.936	7045	6225	6403
G82812	0.646	1.121	1.180	7046	7197	7230
E84635	0.645	0.977	0.947	7047	6729	6498
F85071	0.644	1.004	1.012	7048	6895	6843
G85632	0.644	1.055	1.056	7049	7057	7002
B86032	0.642	1.318	1.320	7050	7344	7333
F83011	0.642	0.983	0.994	7051	6777	6759
E84709	0.641	1.009	1.004	7052	6918	6816
M85178	0.641	1.327	1.370	7053	7347	7349
E85625	0.641	0.992	1.052	7054	6838	6996
E83621	0.640	1.038	1.071	7055	7016	7046
F83034	0.640	1.093	1.169	7056	7160	7226
H84061	0.640	0.965	0.936	7057	6699	6461
C84664	0.639	1.056	1.172	7058	7069	7228
B82026	0.639	1.100	1.049	7059	7173	6992
F85031	0.638	0.970	0.979	7060	6733	6705
F83019	0.638	1.053	1.019	7061	7064	6895
F84035	0.637	1.073	1.158	7062	7116	7213
E83644	0.637	1.101	1.114	7063	7176	7164
M85063	0.637	1.098	1.049	7064	7172	6997
N84010	0.636	1.080	1.153	7065	7137	7208
F83664	0.636	1.009	1.031	7066	6939	6937
K84026	0.634	0.979	0.959	7067	6805	6617
H85069	0.634	0.973	0.946	7068	6771	6545
F84030	0.634	1.032	1.040	7069	7020	6974
E84036	0.633	1.027	1.064	7070	7001	7047
L81112	0.633	0.993	1.017	7071	6890	6900
Y00080	0.632	1.055	1.053	7072	7084	7018
Y02686	0.632	0.997	1.091	7073	6904	7120
B82027	0.632	1.060	1.038	7074	7097	6979
G85138	0.631	1.034	1.007	7075	7031	6868
H85664	0.631	0.889	0.937	7076	6235	6518
M92012	0.631	1.119	1.133	7077	7215	7190
E84667	0.631	1.058	1.103	7078	7093	7154
D81001	0.630	0.960	0.920	7079	6720	6415
K84080	0.630	0.981	1.006	7080	6841	6866
D81054	0.630	0.923	0.878	7081	6505	6114
F83665	0.630	0.953	0.941	7082	6689	6539
F83055	0.629	0.980	0.958	7083	6839	6639
M85721	0.629	0.942	0.988	7084	6632	6799
H82037	0.629	1.287	1.238	7085	7342	7301
K84011	0.627	1.001	0.984	7086	6941	6787
M85088	0.627	1.064	1.076	7087	7123	7096
H83017	0.627	1.019	1.001	7088	6997	6861
Y02721	0.626	1.143	1.238	7089	7250	7303
E83632	0.626	1.119	1.050	7090	7227	7027
G85031	0.625	1.037	1.064	7091	7056	7074
F84063	0.623	1.010	1.082	7092	6982	7126

F82009	0.623	1.083	1.048	7093	7169	7033
E84051	0.623	1.041	0.998	7094	7071	6865
E87753	0.622	1.049	1.130	7095	7094	7194
E85091	0.622	0.934	0.985	7096	6633	6817
F83686	0.621	0.989	1.087	7097	6921	7139
E85628	0.621	0.996	0.994	7098	6945	6855
E87010	0.621	0.977	0.988	7099	6870	6839
H81006	0.621	1.041	0.991	7100	7076	6848
F84647	0.621	0.967	0.951	7101	6817	6648
E85038	0.620	0.976	1.030	7102	6872	6989
E85062	0.619	0.921	0.887	7103	6566	6261
G83065	0.618	0.956	0.954	7104	6757	6682
H85075	0.618	0.972	1.009	7105	6858	6923
K81655	0.617	1.065	1.020	7106	7148	6966
F85013	0.617	0.971	0.954	7107	6857	6688
E85066	0.615	0.949	0.980	7108	6740	6824
G81044	0.614	1.013	1.073	7109	7021	7124
G82697	0.613	1.177	1.281	7110	7291	7329
E86619	0.613	1.002	0.994	7111	6984	6885
F84123	0.613	0.936	0.954	7112	6685	6710
E87005	0.613	0.978	0.989	7113	6908	6870
B81091	0.612	1.045	1.100	7114	7108	7172
M86029	0.612	0.964	0.943	7115	6843	6651
G85105	0.612	0.968	0.969	7116	6869	6789
Y02957	0.612	0.876	0.910	7117	6295	6484
F82642	0.611	1.229	1.306	7118	7328	7338
C84117	0.611	0.887	0.942	7119	6375	6654
E83005	0.611	0.990	0.982	7120	6956	6851
Y00449	0.611	0.955	0.971	7121	6804	6808
F84036	0.610	1.047	1.083	7122	7122	7156
E84705	0.608	0.949	0.970	7123	6781	6813
E87701	0.608	0.920	0.889	7124	6628	6359
Y02672	0.608	0.941	0.916	7125	6737	6528
Y03506	0.607	0.966	1.029	7126	6885	7020
K81605	0.606	0.889	0.889	7127	6435	6368
B81032	0.606	1.115	1.228	7128	7244	7310
A83011	0.606	0.945	0.952	7129	6769	6742
G85044	0.606	1.072	1.077	7130	7183	7149
F84718	0.605	1.037	1.013	7131	7104	6985
E85005	0.604	0.997	1.071	7132	6998	7138
F84074	0.604	1.106	1.081	7133	7238	7163
E87004	0.603	0.957	0.932	7134	6855	6640
E87061	0.603	0.911	0.928	7135	6604	6616
F84124	0.602	0.947	0.988	7136	6801	6907
E84674	0.601	0.933	0.922	7137	6727	6591
E84669	0.601	1.001	0.957	7138	7026	6786
P84683	0.601	0.944	1.056	7139	6791	7111
H85027	0.601	0.937	0.912	7140	6756	6541
E85719	0.600	0.943	0.954	7141	6794	6777
Y02671	0.600	0.901	0.873	7142	6562	6307

B86108	0.600	1.050	1.127	7143	7154	7224
E85694	0.600	0.945	0.921	7144	6809	6599
F86626	0.599	0.917	0.999	7145	6664	6957
F85061	0.599	0.964	1.052	7146	6909	7106
M85002	0.598	0.986	0.993	7147	6983	6936
F84686	0.597	0.872	0.885	7148	6357	6399
F85023	0.597	1.138	1.118	7149	7272	7215
F85700	0.596	0.960	1.013	7150	6903	7013
C84605	0.595	1.062	1.075	7151	7184	7167
J81645	0.595	1.029	1.063	7152	7110	7145
F84631	0.595	1.002	1.031	7153	7048	7068
G85025	0.594	1.001	0.998	7154	7049	6968
G85647	0.593	0.973	1.012	7155	6959	7015
E87013	0.593	0.959	0.938	7156	6913	6737
F84656	0.592	0.972	0.957	7157	6958	6827
E83013	0.592	0.964	0.927	7158	6937	6680
F83059	0.592	1.030	0.995	7159	7126	6965
C84151	0.591	1.052	1.144	7160	7171	7247
P81038	0.590	1.196	1.129	7161	7317	7235
F82010	0.590	1.254	1.191	7162	7343	7296
F84055	0.587	0.985	1.045	7163	7017	7121
Y02471	0.586	1.003	1.055	7164	7068	7144
G85023	0.586	1.006	1.030	7165	7075	7086
F83623	0.586	0.944	0.930	7166	6881	6730
F86088	0.586	1.055	1.113	7167	7187	7220
C83053	0.584	1.093	1.040	7168	7245	7113
E87007	0.584	0.929	0.942	7169	6802	6796
G85094	0.584	1.037	1.011	7170	7159	7037
F83057	0.584	1.048	1.034	7171	7178	7099
F85628	0.584	1.065	1.128	7172	7203	7240
E83637	0.583	0.943	0.893	7173	6888	6535
Y00200	0.583	0.950	1.013	7174	6917	7043
C84101	0.583	1.129	1.141	7175	7277	7254
E84006	0.580	1.066	1.012	7176	7213	7054
C84013	0.578	1.211	1.184	7177	7333	7298
Y02987	0.576	0.980	0.915	7178	7037	6698
L81040	0.574	1.126	1.078	7179	7281	7192
L84054	0.574	1.120	1.034	7180	7276	7128
F84742	0.574	0.949	0.968	7181	6948	6934
F84729	0.573	1.005	1.013	7182	7101	7076
Y03441	0.573	0.911	0.931	7183	6760	6794
B83660	0.573	1.153	1.235	7184	7300	7327
P81011	0.573	1.113	1.121	7185	7271	7243
Y04664	0.572	1.029	0.997	7186	7166	7029
P81155	0.570	1.213	1.301	7187	7338	7350
K84605	0.569	0.886	0.913	7188	6656	6721
C84114	0.569	1.184	1.217	7189	7327	7319
L81643	0.568	1.144	1.104	7190	7297	7233
K84621	0.566	1.020	1.010	7191	7161	7085
D83073	0.566	1.110	1.138	7192	7273	7273

H85020	0.566	1.165	1.122	7193	7313	7252
E85677	0.566	0.952	1.015	7194	6975	7097
B85062	0.564	0.877	0.890	7195	6635	6629
E86034	0.564	1.018	1.012	7196	7163	7094
E86618	0.564	1.054	1.062	7197	7218	7183
M92035	0.563	0.979	1.032	7198	7065	7146
B86068	0.563	1.060	1.105	7199	7231	7238
M85100	0.563	1.099	1.140	7200	7264	7280
G85674	0.562	0.938	1.025	7201	6951	7131
K84016	0.562	0.957	0.943	7202	7003	6894
M91026	0.561	1.341	1.410	7203	7361	7362
Y03135	0.561	0.957	0.976	7204	7006	7007
F84114	0.560	0.972	0.951	7205	7059	6922
K84030	0.559	1.093	1.041	7206	7262	7169
L81015	0.557	1.163	1.152	7207	7318	7291
B82098	0.555	1.046	1.025	7208	7221	7148
G85711	0.554	0.991	1.062	7209	7120	7195
F84740	0.554	0.912	0.942	7210	6879	6916
Y01962	0.554	0.902	0.920	7211	6827	6834
M83670	0.553	0.893	0.913	7212	6775	6806
G85014	0.553	1.023	1.009	7213	7191	7115
G84041	0.552	1.037	1.060	7214	7210	7197
E87741	0.549	1.013	1.082	7215	7180	7229
E84665	0.548	0.969	0.986	7216	7079	7073
E85074	0.548	0.960	1.015	7217	7058	7141
H84020	0.547	0.905	0.881	7218	6882	6673
P81093	0.547	1.105	1.147	7219	7286	7294
E84647	0.546	1.019	1.040	7220	7193	7175
E84086	0.546	0.935	0.908	7221	6986	6814
K84021	0.545	0.949	0.933	7222	7035	6914
Y01719	0.544	1.014	1.097	7223	7186	7249
P84630	0.544	0.986	1.044	7224	7135	7187
N82059	0.539	0.988	1.098	7225	7151	7255
E87063	0.538	0.933	0.990	7226	7002	7103
Y02589	0.537	0.926	0.977	7227	6988	7078
J82024	0.536	0.985	1.013	7228	7153	7162
E87720	0.536	0.936	0.950	7229	7025	7005
E85692	0.535	0.975	0.966	7230	7131	7048
E87742	0.533	1.072	1.109	7231	7267	7274
Y02847	0.533	0.962	1.084	7232	7098	7245
F86044	0.532	1.036	1.123	7233	7240	7289
G85047	0.531	1.001	1.034	7234	7190	7189
F84660	0.531	0.967	0.982	7235	7113	7100
G81034	0.531	1.002	1.016	7236	7192	7171
M83708	0.531	1.210	1.328	7237	7345	7357
E85673	0.530	1.032	1.117	7238	7237	7287
P81064	0.529	0.955	0.960	7239	7095	7049
F83633	0.524	0.940	0.916	7240	7066	6925
D81037	0.523	0.908	0.869	7241	6972	6738
D83046	0.522	1.075	1.083	7242	7283	7256

B82103	0.522	1.089	1.093	7243	7293	7272
E87046	0.521	0.917	0.897	7244	7009	6871
H84005	0.519	0.961	0.918	7245	7133	6951
F85067	0.518	1.019	1.089	7246	7236	7271
F81141	0.517	0.913	0.910	7247	7004	6926
Y01655	0.517	0.938	0.992	7248	7080	7158
B81118	0.516	1.108	1.105	7249	7311	7288
L81133	0.512	0.860	0.868	7250	6822	6785
J82055	0.512	1.080	1.124	7251	7294	7304
G85721	0.511	0.977	1.030	7252	7182	7210
E84049	0.511	1.069	1.087	7253	7285	7276
E85725	0.508	1.067	1.058	7254	7288	7244
G85020	0.507	0.928	0.946	7255	7077	7075
C82105	0.506	0.989	1.032	7256	7207	7219
E84066	0.505	1.005	1.023	7257	7234	7209
F84072	0.505	0.941	0.985	7258	7118	7168
E87702	0.504	0.920	0.969	7259	7067	7136
E87665	0.503	0.976	0.951	7260	7194	7095
F85034	0.501	0.970	1.009	7261	7188	7201
C82124	0.500	0.872	0.886	7262	6938	6911
D81026	0.498	1.145	1.112	7263	7339	7305
G85091	0.498	0.972	1.033	7264	7195	7231
E84701	0.497	0.959	0.930	7265	7174	7057
G85715	0.496	0.949	0.959	7266	7158	7132
G85135	0.494	1.234	1.266	7267	7356	7355
M82042	0.493	1.180	1.218	7268	7348	7347
E87745	0.491	0.946	0.924	7269	7164	7059
G81646	0.490	1.022	1.143	7270	7261	7323
F83635	0.490	1.050	1.091	7271	7289	7297
F84635	0.488	0.989	0.985	7272	7235	7181
E87737	0.488	0.915	0.921	7273	7096	7062
M85684	0.484	0.930	0.948	7274	7144	7135
C88007	0.483	0.907	0.934	7275	7087	7101
D82088	0.480	0.894	0.918	7276	7062	7071
M85118	0.480	1.067	1.074	7277	7308	7290
P84605	0.479	1.111	1.193	7278	7332	7345
C82020	0.478	0.911	0.926	7279	7106	7093
E87637	0.478	1.091	1.146	7280	7326	7331
F84636	0.475	0.965	1.041	7281	7217	7264
G85137	0.472	0.963	0.999	7282	7220	7222
E87715	0.472	0.920	0.926	7283	7146	7108
F83005	0.471	0.839	0.897	7284	6922	7031
L81086	0.471	1.081	1.041	7285	7320	7270
B82047	0.470	0.919	0.909	7286	7152	7077
P84068	0.469	1.082	1.122	7287	7325	7322
G81013	0.468	1.081	1.209	7288	7324	7352
H84023	0.466	0.963	0.928	7289	7230	7130
C81629	0.466	1.048	1.037	7290	7304	7269
F83058	0.465	0.903	0.963	7291	7125	7182
E83622	0.463	0.946	0.938	7292	7208	7159

E85746	0.462	0.984	0.993	7293	7257	7227
E84025	0.462	0.974	0.952	7294	7246	7173
P92607	0.461	1.145	1.188	7295	7346	7348
E84637	0.461	0.978	1.001	7296	7252	7237
Y02622	0.460	0.890	1.011	7297	7100	7246
E85013	0.455	0.995	1.033	7298	7270	7279
E87066	0.453	0.945	0.920	7299	7224	7142
Y01929	0.451	1.152	1.187	7300	7351	7351
N82117	0.449	0.928	0.957	7301	7199	7199
K84013	0.449	0.917	0.900	7302	7185	7102
H84639	0.448	0.896	0.902	7303	7147	7110
E84656	0.448	1.059	1.091	7304	7321	7317
L83616	0.447	1.085	1.063	7305	7337	7308
F85640	0.447	0.964	1.014	7306	7251	7266
M85167	0.445	0.867	0.866	7307	7083	7017
E84078	0.444	1.075	1.028	7308	7331	7285
F83061	0.441	0.993	0.995	7309	7282	7250
K84078	0.441	0.867	0.839	7310	7091	6945
G85127	0.441	1.000	1.073	7311	7287	7315
F86704	0.438	0.953	0.981	7312	7248	7239
F84025	0.438	1.031	1.053	7313	7312	7307
M85680	0.438	0.962	0.990	7314	7258	7248
L81622	0.437	0.972	1.064	7315	7263	7312
F84694	0.432	1.014	1.090	7316	7303	7325
E86030	0.425	0.868	0.850	7317	7136	7032
Y02002	0.423	0.870	0.923	7318	7142	7185
E84063	0.423	0.901	0.882	7319	7198	7123
F86701	0.419	0.998	1.071	7320	7299	7321
Y00492	0.418	0.933	0.981	7321	7247	7259
F84692	0.418	1.020	1.066	7322	7315	7318
G85040	0.414	1.021	1.071	7323	7319	7324
B86110	0.407	0.847	0.860	7324	7132	7105
C84023	0.403	0.857	0.875	7325	7162	7152
Y02747	0.402	0.993	1.085	7326	7310	7335
E87048	0.401	0.907	0.957	7327	7241	7253
J82199	0.399	0.848	0.855	7328	7150	7114
E87722	0.398	0.890	0.871	7329	7225	7155
C82111	0.396	0.860	0.864	7330	7179	7143
F81708	0.393	0.954	0.959	7331	7290	7265
E85124	0.387	1.161	1.273	7332	7360	7366
E87069	0.384	0.935	0.991	7333	7280	7299
L81617	0.379	0.872	0.862	7334	7226	7170
E87043	0.375	0.904	0.883	7335	7259	7200
B81104	0.375	0.925	1.068	7336	7279	7337
J82080	0.369	0.868	0.877	7337	7233	7198
G85076	0.368	0.905	0.952	7338	7265	7284
F83050	0.368	0.967	1.017	7339	7314	7320
N81624	0.367	1.082	1.036	7340	7352	7330
F83043	0.364	0.846	0.910	7341	7205	7241
F85705	0.362	0.995	1.020	7342	7334	7326

C84714	0.358	0.875	0.980	7343	7249	7309
M88044	0.356	1.014	1.075	7344	7341	7346
F83044	0.343	1.066	1.024	7345	7354	7334
F81042	0.342	1.043	1.043	7346	7350	7339
E87070	0.338	0.949	0.952	7347	7323	7306
E87738	0.325	0.892	0.902	7348	7292	7277
A86027	0.325	0.821	0.828	7349	7229	7191
E87677	0.325	0.831	0.886	7350	7242	7257
H84041	0.324	0.908	0.879	7351	7306	7251
P81694	0.322	1.221	1.344	7352	7371	7372
J81620	0.317	1.158	1.135	7353	7365	7361
C88627	0.316	0.855	0.878	7354	7268	7260
M85778	0.309	0.848	0.910	7355	7266	7295
G82140	0.308	0.877	0.884	7356	7295	7278
F82678	0.304	1.174	1.229	7357	7368	7371
F81136	0.290	1.006	1.004	7358	7353	7344
E87067	0.288	1.111	1.149	7359	7363	7363
G81071	0.262	0.851	0.872	7360	7309	7302
E87750	0.238	0.986	1.038	7361	7357	7358
H83634	0.227	0.981	1.044	7362	7359	7360
E87768	0.226	0.860	0.931	7363	7335	7341
M85766	0.224	1.057	1.087	7364	7364	7364
M89003	0.223	1.179	1.094	7365	7372	7365
Y04273	0.219	0.918	0.987	7366	7349	7353
Y03671	0.214	1.056	1.108	7367	7366	7367
F81732	0.211	1.087	1.116	7368	7369	7369
Y02575	0.171	0.955	0.977	7369	7362	7359
G82796	0.170	1.311	1.401	7370	7375	7376
C81633	0.137	1.202	1.262	7371	7373	7374
Y04809	0.123	1.266	1.318	7372	7376	7375
B83051	0.110	0.843	0.887	7373	7355	7356
J82096	0.079	1.189	1.126	7374	7374	7373
E85686	0.050	0.950	0.951	7375	7370	7368
