# Supplementary material for: Identifying English Practices that Are High Antibiotic Prescribers Accounting for Comorbidities and Other Legitimate Medical Reasons for Variation
Source: eClinicalMedicine. 2018 Dec 12;6:36–41. doi: 10.1016/j.eclinm.2018.12.003 (PMC6358038; doi:10.1016/j.eclinm.2018.12.003)
Supplement: Supplementary file 2 — Table S2. Ranking of general practices in the sensitivity analysis based on antibiotic prescribing per STAR-PU, the comorbidity and full model. [file mmc2.pdf]

| Practice code | Observed prescribing rate per STAR-PU | Predicted prescribing rate per STAR-PU based on comorbidity model | Predicted prescribing rate per STAR-PU based on full model | Ranking based on STAR-PU | Ranking based on comorbidity model | Ranking based on full model |
|---|---|---|---|---|---|---|
| Y02421 | 2.853 | 0.90 | 0.91 | 1 | 1 | 1 |
| Y02625 | 2.740 | 3.19 | 3.14 | 2 | 7162 | 7069 |
| Y02974 | 2.668 | 0.81 | 0.81 | 3 | 2 | 2 |
| Y02566 | 2.651 | 0.79 | 0.81 | 4 | 3 | 3 |
| Y02639 | 2.624 | 0.83 | 0.84 | 5 | 4 | 4 |
| G82641 | 2.561 | 1.13 | 1.12 | 6 | 9 | 8 |
| Y02570 | 2.502 | 0.98 | 0.99 | 7 | 5 | 5 |
| F86686 | 2.456 | 1.04 | 1.06 | 8 | 10 | 10 |
| Y02662 | 2.455 | 0.96 | 0.98 | 9 | 6 | 6 |
| N82036 | 2.383 | 1.50 | 1.48 | 10 | 36 | 33 |
| Y02973 | 2.362 | 0.90 | 0.90 | 11 | 7 | 7 |
| Y02890 | 2.347 | 0.90 | 0.92 | 12 | 8 | 9 |
| C86621 | 2.336 | 1.37 | 1.39 | 13 | 26 | 27 |
| A82077 | 2.311 | 1.04 | 1.07 | 14 | 15 | 16 |
| Y02720 | 2.246 | 1.05 | 1.06 | 15 | 18 | 18 |
| Y02711 | 2.239 | 1.01 | 1.02 | 16 | 17 | 17 |
| B81683 | 2.231 | 1.14 | 1.18 | 17 | 21 | 23 |
| Y02968 | 2.221 | 0.92 | 0.91 | 18 | 14 | 13 |
| Y00265 | 2.199 | 0.83 | 0.84 | 19 | 11 | 11 |
| E85105 | 2.195 | 1.03 | 1.04 | 20 | 19 | 19 |
| B81018 | 2.169 | 1.43 | 1.46 | 21 | 78 | 85 |
| Y03296 | 2.161 | 0.81 | 0.81 | 22 | 12 | 12 |
| B85048 | 2.157 | 1.09 | 1.09 | 23 | 22 | 21 |
| C88042 | 2.153 | 1.35 | 1.35 | 24 | 49 | 49 |
| Y02656 | 2.103 | 1.32 | 1.29 | 25 | 57 | 45 |
| G82696 | 2.103 | 1.32 | 1.34 | 26 | 59 | 62 |
| P84024 | 2.087 | 1.32 | 1.33 | 27 | 65 | 68 |
| P85605 | 2.087 | 1.22 | 1.24 | 28 | 38 | 39 |
| Y02849 | 2.083 | 0.76 | 0.78 | 29 | 13 | 14 |
| M86604 | 2.082 | 1.03 | 1.03 | 30 | 23 | 22 |
| Y02578 | 2.072 | 0.81 | 0.82 | 31 | 16 | 15 |
| F81037 | 2.071 | 1.18 | 1.19 | 32 | 33 | 34 |
| G82007 | 2.060 | 1.28 | 1.31 | 33 | 62 | 71 |
| M91022 | 2.054 | 1.20 | 1.21 | 34 | 41 | 40 |
| M91639 | 2.054 | 1.28 | 1.29 | 35 | 66 | 65 |
| M85722 | 2.050 | 0.92 | 0.91 | 36 | 20 | 20 |
| H85643 | 2.026 | 1.13 | 1.10 | 37 | 32 | 30 |
| B83613 | 2.022 | 1.10 | 1.12 | 38 | 30 | 32 |
| K81085 | 2.018 | 1.10 | 1.11 | 39 | 31 | 31 |
| F83030 | 2.015 | 1.07 | 1.05 | 40 | 28 | 26 |
| B81674 | 2.010 | 1.22 | 1.26 | 41 | 53 | 70 |
| M85001 | 2.007 | 1.29 | 1.27 | 42 | 84 | 73 |
| A89624 | 2.007 | 1.12 | 1.13 | 43 | 34 | 35 |
| N82647 | 2.002 | 1.05 | 1.07 | 44 | 27 | 29 |

| | | | | | | |
|---|---|---|---|---|---|---|
| C84031 | 1.976 | 1.09 | 1.12 | 45 | 35 | 37 |
| C88021 | 1.974 | 1.10 | 1.12 | 46 | 37 | 38 |
| C84140 | 1.972 | 1.18 | 1.20 | 47 | 54 | 63 |
| B87006 | 1.967 | 1.46 | 1.48 | 48 | 217 | 229 |
| N82101 | 1.967 | 1.35 | 1.35 | 49 | 127 | 124 |
| D81624 | 1.959 | 1.15 | 1.15 | 50 | 44 | 44 |
| P81763 | 1.953 | 1.09 | 1.09 | 51 | 39 | 36 |
| A82040 | 1.933 | 1.48 | 1.49 | 52 | 272 | 296 |
| P85610 | 1.933 | 1.08 | 1.10 | 53 | 40 | 41 |
| P87652 | 1.932 | 1.09 | 1.11 | 54 | 42 | 43 |
| F84657 | 1.929 | 1.17 | 1.14 | 55 | 72 | 51 |
| P84014 | 1.910 | 1.23 | 1.25 | 56 | 93 | 100 |
| F82042 | 1.906 | 0.92 | 0.94 | 57 | 25 | 25 |
| C84704 | 1.906 | 1.26 | 1.28 | 58 | 111 | 117 |
| B85060 | 1.906 | 1.44 | 1.42 | 59 | 262 | 239 |
| Y02718 | 1.903 | 1.09 | 1.10 | 60 | 45 | 48 |
| J83649 | 1.900 | 1.09 | 1.11 | 61 | 46 | 50 |
| N82678 | 1.899 | 1.10 | 1.12 | 62 | 51 | 55 |
| F81212 | 1.898 | 1.29 | 1.29 | 63 | 128 | 128 |
| B81012 | 1.896 | 1.12 | 1.14 | 64 | 63 | 69 |
| N83031 | 1.892 | 1.10 | 1.10 | 65 | 52 | 53 |
| F81156 | 1.890 | 1.42 | 1.41 | 66 | 258 | 240 |
| A89041 | 1.890 | 1.15 | 1.16 | 67 | 77 | 78 |
| P86619 | 1.890 | 1.19 | 1.21 | 68 | 88 | 92 |
| F81104 | 1.886 | 1.09 | 1.10 | 69 | 50 | 52 |
| B83069 | 1.885 | 1.22 | 1.24 | 70 | 100 | 108 |
| G82722 | 1.883 | 1.10 | 1.07 | 71 | 55 | 47 |
| B81087 | 1.882 | 1.13 | 1.16 | 72 | 73 | 80 |
| B81606 | 1.872 | 1.22 | 1.24 | 73 | 105 | 113 |
| C83028 | 1.871 | 1.25 | 1.25 | 74 | 125 | 121 |
| A81038 | 1.870 | 1.28 | 1.28 | 75 | 141 | 144 |
| Y00026 | 1.868 | 1.06 | 1.08 | 76 | 47 | 54 |
| P91615 | 1.867 | 1.12 | 1.13 | 77 | 74 | 76 |
| P84055 | 1.866 | 1.09 | 1.11 | 78 | 64 | 66 |
| G81651 | 1.866 | 1.29 | 1.31 | 79 | 157 | 167 |
| C87616 | 1.861 | 1.28 | 1.29 | 80 | 155 | 157 |
| G82204 | 1.857 | 1.10 | 1.10 | 81 | 71 | 67 |
| D81015 | 1.857 | 1.15 | 1.20 | 82 | 86 | 101 |
| B85009 | 1.856 | 1.16 | 1.17 | 83 | 92 | 88 |
| G82235 | 1.854 | 1.07 | 1.07 | 84 | 60 | 56 |
| L83643 | 1.854 | 1.23 | 1.23 | 85 | 118 | 123 |
| Y02162 | 1.854 | 1.05 | 1.04 | 86 | 48 | 46 |
| A87023 | 1.853 | 1.15 | 1.16 | 87 | 87 | 87 |
| H81611 | 1.851 | 0.93 | 0.90 | 88 | 29 | 28 |
| K81039 | 1.851 | 1.31 | 1.30 | 89 | 174 | 169 |
| N83611 | 1.847 | 1.36 | 1.36 | 90 | 233 | 232 |
| P85608 | 1.847 | 1.36 | 1.36 | 91 | 241 | 243 |
| A83617 | 1.844 | 1.27 | 1.28 | 92 | 161 | 161 |
| P84025 | 1.841 | 1.06 | 1.07 | 93 | 56 | 61 |
| P82033 | 1.839 | 1.19 | 1.20 | 94 | 107 | 109 |

| | | | | | | |
|---|---|---|---|---|---|---|
| G82233 | 1.838 | 1.07 | 1.10 | 95 | 67 | 74 |
| C85624 | 1.837 | 1.63 | 1.61 | 96 | 1208 | 1090 |
| P87036 | 1.834 | 1.09 | 1.12 | 97 | 76 | 83 |
| C83030 | 1.832 | 1.32 | 1.31 | 98 | 208 | 191 |
| C86625 | 1.827 | 1.25 | 1.27 | 99 | 154 | 164 |
| P84049 | 1.826 | 1.31 | 1.31 | 100 | 187 | 199 |
| P82022 | 1.818 | 1.21 | 1.22 | 101 | 133 | 140 |
| M85730 | 1.817 | 1.09 | 1.08 | 102 | 81 | 75 |
| P84029 | 1.815 | 1.30 | 1.31 | 103 | 191 | 201 |
| P88003 | 1.811 | 1.31 | 1.29 | 104 | 219 | 195 |
| H85019 | 1.810 | 1.05 | 1.03 | 105 | 69 | 57 |
| M91624 | 1.810 | 1.11 | 1.12 | 106 | 90 | 89 |
| C82008 | 1.809 | 1.15 | 1.14 | 107 | 99 | 95 |
| J82077 | 1.807 | 1.09 | 1.06 | 108 | 83 | 72 |
| P81756 | 1.805 | 1.22 | 1.25 | 109 | 147 | 166 |
| Y02354 | 1.795 | 1.20 | 1.23 | 110 | 138 | 162 |
| N85640 | 1.794 | 1.24 | 1.22 | 111 | 169 | 156 |
| B87604 | 1.793 | 1.30 | 1.31 | 112 | 227 | 241 |
| Y02585 | 1.793 | 0.78 | 0.80 | 113 | 24 | 24 |
| F84091 | 1.791 | 1.13 | 1.12 | 114 | 102 | 93 |
| A89004 | 1.791 | 1.24 | 1.26 | 115 | 170 | 175 |
| Y02414 | 1.791 | 1.03 | 1.02 | 116 | 68 | 64 |
| Y02605 | 1.790 | 1.14 | 1.19 | 117 | 108 | 133 |
| G82029 | 1.787 | 1.20 | 1.21 | 118 | 148 | 149 |
| M85779 | 1.786 | 1.20 | 1.19 | 119 | 151 | 134 |
| Y00469 | 1.786 | 1.54 | 1.53 | 120 | 985 | 876 |
| Y01200 | 1.785 | 1.18 | 1.22 | 121 | 132 | 163 |
| C88647 | 1.784 | 1.13 | 1.15 | 122 | 104 | 112 |
| F81060 | 1.783 | 1.25 | 1.25 | 123 | 180 | 179 |
| K82050 | 1.779 | 1.11 | 1.09 | 124 | 96 | 91 |
| C82019 | 1.771 | 1.11 | 1.11 | 125 | 101 | 99 |
| A89612 | 1.765 | 1.16 | 1.16 | 126 | 136 | 131 |
| N81626 | 1.763 | 1.12 | 1.10 | 127 | 115 | 96 |
| P91617 | 1.760 | 0.98 | 0.98 | 128 | 58 | 60 |
| N83008 | 1.756 | 1.45 | 1.45 | 129 | 672 | 662 |
| G81075 | 1.755 | 1.12 | 1.14 | 130 | 117 | 125 |
| M84026 | 1.754 | 1.16 | 1.16 | 131 | 144 | 141 |
| P86019 | 1.753 | 1.16 | 1.18 | 132 | 142 | 154 |
| M83616 | 1.751 | 1.09 | 1.12 | 133 | 97 | 114 |
| C84629 | 1.751 | 1.24 | 1.25 | 134 | 213 | 209 |
| G84028 | 1.743 | 1.05 | 1.05 | 135 | 91 | 86 |
| B85020 | 1.742 | 1.21 | 1.21 | 136 | 181 | 176 |
| P86022 | 1.740 | 1.17 | 1.18 | 137 | 158 | 168 |
| C86616 | 1.740 | 1.47 | 1.46 | 138 | 800 | 750 |
| G81669 | 1.739 | 1.13 | 1.14 | 139 | 134 | 138 |
| M91659 | 1.738 | 1.42 | 1.44 | 140 | 617 | 683 |
| P86011 | 1.738 | 1.24 | 1.25 | 141 | 225 | 228 |
| H83052 | 1.737 | 1.01 | 1.00 | 142 | 79 | 77 |
| P92616 | 1.737 | 1.21 | 1.21 | 143 | 185 | 189 |
| N81127 | 1.736 | 1.32 | 1.29 | 144 | 357 | 285 |

| | | | | | | |
|---|---|---|---|---|---|---|
| H82101 | 1.733 | 1.09 | 1.09 | 145 | 112 | 107 |
| G82791 | 1.732 | 1.03 | 1.05 | 146 | 89 | 90 |
| D82072 | 1.732 | 1.15 | 1.16 | 147 | 153 | 152 |
| C81008 | 1.729 | 1.28 | 1.27 | 148 | 286 | 275 |
| C88626 | 1.728 | 1.48 | 1.48 | 149 | 960 | 923 |
| A83638 | 1.725 | 1.40 | 1.38 | 150 | 570 | 517 |
| D81065 | 1.724 | 1.09 | 1.10 | 151 | 116 | 119 |
| B87036 | 1.721 | 1.32 | 1.33 | 152 | 385 | 401 |
| F82668 | 1.719 | 1.11 | 1.11 | 153 | 130 | 132 |
| P86602 | 1.719 | 1.21 | 1.22 | 154 | 211 | 225 |
| P83020 | 1.717 | 1.18 | 1.19 | 155 | 179 | 180 |
| M85008 | 1.716 | 1.45 | 1.40 | 156 | 832 | 622 |
| G82762 | 1.715 | 0.97 | 1.00 | 157 | 75 | 81 |
| G83057 | 1.713 | 1.10 | 1.09 | 158 | 126 | 120 |
| B83638 | 1.712 | 1.04 | 1.05 | 159 | 94 | 97 |
| H85008 | 1.712 | 1.01 | 0.98 | 160 | 85 | 79 |
| B81054 | 1.711 | 1.24 | 1.29 | 161 | 261 | 328 |
| N82019 | 1.704 | 1.23 | 1.24 | 162 | 251 | 264 |
| P81212 | 1.702 | 1.22 | 1.21 | 163 | 235 | 233 |
| F81670 | 1.702 | 1.38 | 1.37 | 164 | 576 | 576 |
| M85713 | 1.701 | 1.33 | 1.31 | 165 | 440 | 402 |
| A82041 | 1.701 | 1.21 | 1.23 | 166 | 230 | 263 |
| C88072 | 1.699 | 1.41 | 1.42 | 167 | 749 | 748 |
| G83066 | 1.698 | 1.10 | 1.09 | 168 | 140 | 130 |
| P87040 | 1.694 | 1.30 | 1.32 | 169 | 399 | 430 |
| D81055 | 1.694 | 1.18 | 1.17 | 170 | 207 | 186 |
| P81687 | 1.694 | 1.14 | 1.13 | 171 | 166 | 165 |
| N82034 | 1.693 | 1.28 | 1.29 | 172 | 361 | 364 |
| P81643 | 1.690 | 1.14 | 1.17 | 173 | 171 | 196 |
| B81019 | 1.689 | 1.20 | 1.22 | 174 | 234 | 259 |
| Y03023 | 1.687 | 0.91 | 0.91 | 175 | 61 | 59 |
| F81635 | 1.686 | 1.06 | 1.06 | 176 | 121 | 116 |
| B85619 | 1.682 | 1.28 | 1.27 | 177 | 379 | 353 |
| F81014 | 1.682 | 1.07 | 1.07 | 178 | 129 | 127 |
| M91028 | 1.680 | 1.33 | 1.33 | 179 | 497 | 489 |
| P81196 | 1.678 | 1.03 | 1.03 | 180 | 110 | 105 |
| B81101 | 1.677 | 1.12 | 1.11 | 181 | 163 | 159 |
| Y00110 | 1.677 | 1.29 | 1.29 | 182 | 414 | 399 |
| B81121 | 1.676 | 0.96 | 0.97 | 183 | 82 | 84 |
| N83603 | 1.673 | 1.13 | 1.14 | 184 | 172 | 178 |
| N83605 | 1.672 | 1.23 | 1.23 | 185 | 295 | 299 |
| P81785 | 1.672 | 1.26 | 1.24 | 186 | 364 | 310 |
| N84016 | 1.671 | 1.21 | 1.20 | 187 | 269 | 257 |
| G83663 | 1.670 | 1.08 | 1.07 | 188 | 146 | 136 |
| L83664 | 1.670 | 1.30 | 1.32 | 189 | 455 | 507 |
| E83600 | 1.670 | 0.91 | 0.89 | 190 | 70 | 58 |
| G82022 | 1.669 | 1.02 | 1.02 | 191 | 109 | 104 |
| G82094 | 1.667 | 1.08 | 1.07 | 192 | 143 | 135 |
| C83614 | 1.666 | 1.20 | 1.19 | 193 | 263 | 247 |
| F86612 | 1.665 | 1.04 | 1.03 | 194 | 123 | 111 |

| | | | | | | |
|---|---|---|---|---|---|---|
| A83626 | 1.665 | 1.34 | 1.34 | 195 | 598 | 609 |
| M91655 | 1.664 | 1.34 | 1.34 | 196 | 597 | 596 |
| M87018 | 1.664 | 1.34 | 1.33 | 197 | 590 | 532 |
| F81681 | 1.662 | 1.92 | 1.88 | 198 | 6449 | 6098 |
| G82155 | 1.662 | 1.04 | 1.04 | 199 | 119 | 118 |
| M86629 | 1.662 | 1.09 | 1.08 | 200 | 156 | 148 |
| G82737 | 1.661 | 0.99 | 1.02 | 201 | 95 | 110 |
| C86032 | 1.661 | 1.04 | 1.07 | 202 | 124 | 142 |
| B84001 | 1.660 | 1.21 | 1.22 | 203 | 280 | 302 |
| N83018 | 1.659 | 1.23 | 1.24 | 204 | 325 | 329 |
| A89019 | 1.659 | 1.31 | 1.31 | 205 | 514 | 498 |
| F81656 | 1.658 | 1.12 | 1.12 | 206 | 173 | 174 |
| N84015 | 1.655 | 1.37 | 1.35 | 207 | 743 | 647 |
| B85646 | 1.655 | 1.20 | 1.20 | 208 | 273 | 274 |
| A89610 | 1.654 | 1.20 | 1.21 | 209 | 275 | 291 |
| G82023 | 1.652 | 1.23 | 1.24 | 210 | 341 | 359 |
| F82680 | 1.652 | 1.16 | 1.17 | 211 | 232 | 238 |
| P84028 | 1.651 | 1.14 | 1.15 | 212 | 195 | 206 |
| P82010 | 1.650 | 1.25 | 1.25 | 213 | 378 | 373 |
| M91641 | 1.648 | 1.25 | 1.26 | 214 | 381 | 397 |
| B86089 | 1.647 | 1.13 | 1.15 | 215 | 194 | 214 |
| A89621 | 1.647 | 1.20 | 1.21 | 216 | 291 | 297 |
| C88006 | 1.646 | 1.39 | 1.39 | 217 | 871 | 865 |
| A83029 | 1.646 | 1.21 | 1.22 | 218 | 303 | 324 |
| A86013 | 1.644 | 1.37 | 1.38 | 219 | 804 | 813 |
| M83128 | 1.644 | 1.16 | 1.20 | 220 | 243 | 287 |
| C82650 | 1.644 | 1.07 | 1.07 | 221 | 159 | 155 |
| M85149 | 1.643 | 1.40 | 1.39 | 222 | 992 | 898 |
| N82048 | 1.641 | 1.25 | 1.26 | 223 | 396 | 411 |
| J81632 | 1.639 | 1.19 | 1.19 | 224 | 288 | 283 |
| B83631 | 1.638 | 1.10 | 1.12 | 225 | 175 | 194 |
| E82020 | 1.638 | 1.06 | 1.04 | 226 | 152 | 139 |
| N85038 | 1.638 | 1.36 | 1.32 | 227 | 780 | 635 |
| D81071 | 1.637 | 1.36 | 1.39 | 228 | 768 | 932 |
| Y02986 | 1.637 | 0.82 | 0.81 | 229 | 43 | 42 |
| A83060 | 1.635 | 1.12 | 1.14 | 230 | 203 | 219 |
| G82170 | 1.635 | 0.91 | 0.92 | 231 | 80 | 82 |
| C82099 | 1.635 | 1.08 | 1.09 | 232 | 168 | 171 |
| C88643 | 1.634 | 1.12 | 1.13 | 233 | 201 | 212 |
| B81108 | 1.634 | 1.27 | 1.29 | 234 | 462 | 533 |
| E85656 | 1.634 | 1.44 | 1.44 | 235 | 1292 | 1319 |
| M85077 | 1.633 | 1.07 | 1.06 | 236 | 165 | 158 |
| P84059 | 1.631 | 1.12 | 1.13 | 237 | 200 | 215 |
| B83653 | 1.631 | 1.10 | 1.12 | 238 | 182 | 197 |
| A89014 | 1.630 | 1.12 | 1.13 | 239 | 202 | 208 |
| F81740 | 1.630 | 1.26 | 1.24 | 240 | 453 | 404 |
| M85134 | 1.628 | 1.11 | 1.10 | 241 | 199 | 188 |
| A89617 | 1.627 | 1.19 | 1.19 | 242 | 298 | 307 |
| A83016 | 1.627 | 1.29 | 1.29 | 243 | 532 | 525 |
| Y00286 | 1.627 | 1.17 | 1.17 | 244 | 270 | 280 |

| | | | | | | |
|---|---|---|---|---|---|---|
| N82001 | 1.627 | 1.40 | 1.39 | 245 | 1057 | 1044 |
| L83037 | 1.626 | 1.29 | 1.30 | 246 | 535 | 584 |
| E85090 | 1.624 | 1.11 | 1.12 | 247 | 197 | 203 |
| J83609 | 1.624 | 0.97 | 0.97 | 248 | 106 | 103 |
| C82676 | 1.621 | 1.14 | 1.13 | 249 | 240 | 234 |
| F85634 | 1.621 | 1.08 | 1.08 | 250 | 177 | 172 |
| H83027 | 1.620 | 0.98 | 0.97 | 251 | 113 | 102 |
| C81661 | 1.619 | 1.34 | 1.34 | 252 | 757 | 744 |
| H83620 | 1.617 | 0.96 | 0.95 | 253 | 103 | 98 |
| L84081 | 1.617 | 1.14 | 1.13 | 254 | 257 | 235 |
| P81167 | 1.617 | 1.38 | 1.41 | 255 | 1038 | 1225 |
| M85066 | 1.616 | 1.20 | 1.19 | 256 | 353 | 316 |
| L83626 | 1.616 | 1.29 | 1.29 | 257 | 584 | 605 |
| A87013 | 1.614 | 1.28 | 1.28 | 258 | 541 | 562 |
| L81066 | 1.614 | 1.02 | 1.00 | 259 | 145 | 129 |
| G82150 | 1.613 | 1.18 | 1.19 | 260 | 319 | 325 |
| G82046 | 1.613 | 1.39 | 1.38 | 261 | 1082 | 1051 |
| P82634 | 1.613 | 1.23 | 1.24 | 262 | 419 | 434 |
| P81764 | 1.612 | 1.18 | 1.19 | 263 | 317 | 342 |
| A89030 | 1.611 | 1.26 | 1.26 | 264 | 485 | 495 |
| F84006 | 1.606 | 1.09 | 1.08 | 265 | 196 | 183 |
| K81005 | 1.605 | 1.12 | 1.13 | 266 | 247 | 258 |
| B86638 | 1.603 | 1.16 | 1.17 | 267 | 285 | 311 |
| A89022 | 1.601 | 1.22 | 1.22 | 268 | 412 | 421 |
| N83021 | 1.600 | 1.17 | 1.18 | 269 | 305 | 327 |
| F81679 | 1.599 | 0.99 | 1.00 | 270 | 135 | 137 |
| A81045 | 1.599 | 1.28 | 1.28 | 271 | 609 | 607 |
| N85037 | 1.598 | 1.23 | 1.21 | 272 | 446 | 406 |
| A87027 | 1.598 | 1.30 | 1.30 | 273 | 676 | 688 |
| D82038 | 1.598 | 1.21 | 1.19 | 274 | 413 | 367 |
| N81637 | 1.598 | 0.96 | 0.95 | 275 | 114 | 106 |
| M91654 | 1.596 | 1.01 | 1.02 | 276 | 149 | 153 |
| C85022 | 1.596 | 1.20 | 1.21 | 277 | 393 | 413 |
| B87021 | 1.596 | 1.34 | 1.35 | 278 | 889 | 978 |
| Y00484 | 1.596 | 1.09 | 1.08 | 279 | 206 | 192 |
| M83712 | 1.596 | 1.16 | 1.20 | 280 | 297 | 378 |
| G83003 | 1.594 | 1.16 | 1.15 | 281 | 310 | 288 |
| A86021 | 1.594 | 1.31 | 1.31 | 282 | 737 | 731 |
| M85686 | 1.593 | 1.11 | 1.10 | 283 | 236 | 227 |
| M83641 | 1.592 | 1.16 | 1.12 | 284 | 308 | 256 |
| Y03595 | 1.591 | 1.25 | 1.23 | 285 | 536 | 466 |
| G82113 | 1.591 | 1.07 | 1.10 | 286 | 192 | 224 |
| C86002 | 1.590 | 1.20 | 1.21 | 287 | 409 | 426 |
| Y02769 | 1.590 | 0.93 | 0.92 | 288 | 98 | 94 |
| A83037 | 1.590 | 1.31 | 1.32 | 289 | 758 | 791 |
| D83079 | 1.589 | 1.09 | 1.10 | 290 | 224 | 231 |
| A83627 | 1.589 | 1.66 | 1.62 | 291 | 4374 | 3810 |
| A89005 | 1.588 | 1.22 | 1.24 | 292 | 465 | 491 |
| E81063 | 1.587 | 1.15 | 1.16 | 293 | 293 | 315 |
| A86009 | 1.586 | 1.22 | 1.23 | 294 | 466 | 469 |

| | | | | | | |
|---|---|---|---|---|---|---|
| G82691 | 1.586 | 1.08 | 1.08 | 295 | 218 | 210 |
| J81063 | 1.586 | 1.20 | 1.20 | 296 | 407 | 403 |
| P81004 | 1.586 | 1.31 | 1.34 | 297 | 787 | 987 |
| N81625 | 1.585 | 1.13 | 1.13 | 298 | 266 | 282 |
| F81683 | 1.585 | 0.96 | 0.96 | 299 | 120 | 122 |
| B82045 | 1.585 | 1.15 | 1.14 | 300 | 311 | 292 |
| Y01124 | 1.582 | 1.20 | 1.22 | 301 | 426 | 452 |
| F86007 | 1.582 | 1.07 | 1.05 | 302 | 210 | 181 |
| L85620 | 1.582 | 1.11 | 1.11 | 303 | 254 | 260 |
| B83052 | 1.582 | 1.15 | 1.16 | 304 | 304 | 332 |
| B87015 | 1.582 | 1.19 | 1.20 | 305 | 394 | 420 |
| G82187 | 1.582 | 1.23 | 1.25 | 306 | 487 | 570 |
| C87014 | 1.581 | 1.10 | 1.11 | 307 | 239 | 252 |
| A83637 | 1.580 | 1.45 | 1.44 | 308 | 1837 | 1704 |
| B85640 | 1.578 | 1.19 | 1.18 | 309 | 418 | 379 |
| P83011 | 1.578 | 1.22 | 1.22 | 310 | 493 | 459 |
| E84053 | 1.578 | 1.01 | 0.99 | 311 | 160 | 143 |
| C83064 | 1.578 | 1.84 | 1.84 | 312 | 6492 | 6486 |
| P88615 | 1.577 | 1.13 | 1.12 | 313 | 287 | 268 |
| A83050 | 1.577 | 1.10 | 1.12 | 314 | 256 | 273 |
| P81163 | 1.577 | 1.11 | 1.10 | 315 | 259 | 249 |
| A89006 | 1.576 | 1.16 | 1.17 | 316 | 354 | 372 |
| N81083 | 1.576 | 1.07 | 1.06 | 317 | 216 | 193 |
| B85614 | 1.575 | 1.33 | 1.34 | 318 | 957 | 1006 |
| G82090 | 1.575 | 1.21 | 1.22 | 319 | 477 | 484 |
| B86007 | 1.574 | 1.29 | 1.29 | 320 | 742 | 754 |
| F81155 | 1.574 | 1.08 | 1.08 | 321 | 223 | 221 |
| E84024 | 1.574 | 0.98 | 0.96 | 322 | 139 | 126 |
| A81044 | 1.571 | 1.15 | 1.18 | 323 | 340 | 405 |
| P81218 | 1.571 | 1.19 | 1.24 | 324 | 425 | 569 |
| B85650 | 1.570 | 1.16 | 1.17 | 325 | 368 | 377 |
| M84609 | 1.569 | 1.14 | 1.14 | 326 | 318 | 308 |
| J82650 | 1.568 | 1.18 | 1.17 | 327 | 416 | 375 |
| F81622 | 1.567 | 1.19 | 1.20 | 328 | 441 | 437 |
| G85082 | 1.567 | 1.08 | 1.07 | 329 | 229 | 216 |
| Y02755 | 1.567 | 0.98 | 0.99 | 330 | 150 | 151 |
| A81012 | 1.566 | 1.34 | 1.33 | 331 | 1067 | 1023 |
| C84635 | 1.566 | 1.13 | 1.15 | 332 | 301 | 351 |
| P81742 | 1.566 | 1.46 | 1.44 | 333 | 2116 | 1846 |
| P84047 | 1.566 | 1.22 | 1.23 | 334 | 512 | 541 |
| M83739 | 1.566 | 1.04 | 1.08 | 335 | 186 | 242 |
| G82217 | 1.566 | 1.00 | 1.04 | 336 | 164 | 184 |
| B86101 | 1.565 | 1.21 | 1.22 | 337 | 486 | 518 |
| L83144 | 1.565 | 1.27 | 1.29 | 338 | 716 | 778 |
| C84135 | 1.564 | 1.08 | 1.10 | 339 | 246 | 267 |
| B81066 | 1.563 | 1.16 | 1.20 | 340 | 380 | 467 |
| J84602 | 1.563 | 1.15 | 1.14 | 341 | 362 | 340 |
| G82726 | 1.562 | 1.19 | 1.18 | 342 | 432 | 419 |
| P84663 | 1.561 | 1.13 | 1.15 | 343 | 315 | 361 |
| N82670 | 1.560 | 1.21 | 1.21 | 344 | 492 | 508 |

| | | | | | | |
|---|---|---|---|---|---|---|
| B83011 | 1.559 | 1.24 | 1.25 | 345 | 610 | 639 |
| P92003 | 1.559 | 1.13 | 1.13 | 346 | 307 | 314 |
| N82110 | 1.559 | 1.35 | 1.35 | 347 | 1183 | 1224 |
| F81724 | 1.559 | 1.05 | 1.06 | 348 | 214 | 222 |
| C84692 | 1.558 | 1.27 | 1.26 | 349 | 723 | 680 |
| J81069 | 1.558 | 1.14 | 1.13 | 350 | 331 | 323 |
| A89001 | 1.558 | 1.36 | 1.36 | 351 | 1295 | 1301 |
| D82065 | 1.558 | 1.28 | 1.26 | 352 | 792 | 702 |
| L83663 | 1.556 | 1.25 | 1.25 | 353 | 674 | 661 |
| G82622 | 1.555 | 1.06 | 1.09 | 354 | 228 | 265 |
| G81662 | 1.555 | 1.28 | 1.31 | 355 | 805 | 938 |
| F81696 | 1.552 | 1.17 | 1.18 | 356 | 424 | 428 |
| P81204 | 1.552 | 1.27 | 1.29 | 357 | 763 | 859 |
| N83060 | 1.552 | 1.33 | 1.33 | 358 | 1103 | 1122 |
| F82665 | 1.552 | 1.10 | 1.11 | 359 | 282 | 300 |
| H81113 | 1.551 | 1.01 | 0.98 | 360 | 176 | 160 |
| D82049 | 1.551 | 1.33 | 1.31 | 361 | 1137 | 992 |
| F81649 | 1.551 | 1.14 | 1.15 | 362 | 365 | 376 |
| P81709 | 1.550 | 1.30 | 1.31 | 363 | 922 | 984 |
| F81052 | 1.549 | 1.39 | 1.39 | 364 | 1575 | 1570 |
| P81685 | 1.548 | 1.27 | 1.26 | 365 | 769 | 730 |
| Y02222 | 1.547 | 1.03 | 1.02 | 366 | 190 | 187 |
| F81688 | 1.546 | 1.04 | 1.05 | 367 | 221 | 217 |
| M85043 | 1.545 | 1.32 | 1.29 | 368 | 1096 | 896 |
| E83034 | 1.544 | 0.98 | 0.96 | 369 | 162 | 147 |
| A83027 | 1.543 | 1.17 | 1.18 | 370 | 442 | 455 |
| P84048 | 1.543 | 1.35 | 1.35 | 371 | 1281 | 1333 |
| P88624 | 1.543 | 1.10 | 1.09 | 372 | 289 | 286 |
| K81003 | 1.542 | 0.99 | 0.96 | 373 | 167 | 150 |
| E84033 | 1.542 | 1.02 | 1.00 | 374 | 183 | 173 |
| N85629 | 1.542 | 1.18 | 1.17 | 375 | 479 | 429 |
| A81070 | 1.541 | 1.11 | 1.14 | 376 | 321 | 368 |
| P84045 | 1.541 | 1.19 | 1.20 | 377 | 509 | 544 |
| A86022 | 1.540 | 1.11 | 1.13 | 378 | 323 | 360 |
| M85170 | 1.540 | 1.55 | 1.53 | 379 | 3580 | 3313 |
| A86038 | 1.540 | 1.37 | 1.36 | 380 | 1469 | 1402 |
| M81062 | 1.540 | 1.23 | 1.20 | 381 | 651 | 547 |
| P82004 | 1.539 | 1.23 | 1.24 | 382 | 634 | 669 |
| C82643 | 1.539 | 1.05 | 1.06 | 383 | 231 | 244 |
| F83021 | 1.537 | 0.94 | 0.95 | 384 | 137 | 145 |
| P85601 | 1.537 | 1.23 | 1.24 | 385 | 659 | 699 |
| N82087 | 1.537 | 1.19 | 1.20 | 386 | 528 | 543 |
| N81642 | 1.536 | 1.08 | 1.06 | 387 | 267 | 255 |
| M85141 | 1.536 | 1.39 | 1.36 | 388 | 1692 | 1404 |
| A89614 | 1.535 | 1.29 | 1.30 | 389 | 963 | 997 |
| K83616 | 1.535 | 1.11 | 1.11 | 390 | 333 | 321 |
| H85110 | 1.535 | 1.05 | 1.03 | 391 | 237 | 207 |
| N81096 | 1.535 | 1.23 | 1.23 | 392 | 662 | 670 |
| M85746 | 1.534 | 1.13 | 1.12 | 393 | 377 | 338 |
| P81727 | 1.534 | 1.39 | 1.39 | 394 | 1695 | 1705 |

| | | | | | | |
|---|---|---|---|---|---|---|
| C88074 | 1.534 | 1.10 | 1.12 | 395 | 312 | 348 |
| C87610 | 1.534 | 1.23 | 1.23 | 396 | 652 | 646 |
| F81751 | 1.534 | 1.11 | 1.10 | 397 | 337 | 304 |
| C82642 | 1.533 | 1.21 | 1.22 | 398 | 602 | 637 |
| B86658 | 1.532 | 1.13 | 1.15 | 399 | 387 | 417 |
| L81095 | 1.531 | 1.12 | 1.12 | 400 | 367 | 352 |
| B85023 | 1.531 | 1.05 | 1.05 | 401 | 238 | 237 |
| H81083 | 1.531 | 1.03 | 1.02 | 402 | 226 | 198 |
| B86024 | 1.531 | 1.11 | 1.12 | 403 | 329 | 357 |
| P81724 | 1.530 | 1.19 | 1.22 | 404 | 546 | 650 |
| C82045 | 1.530 | 1.11 | 1.10 | 405 | 344 | 305 |
| M92009 | 1.529 | 1.23 | 1.24 | 406 | 675 | 712 |
| A81034 | 1.528 | 1.11 | 1.12 | 407 | 350 | 356 |
| B85014 | 1.528 | 1.21 | 1.20 | 408 | 592 | 574 |
| K81638 | 1.528 | 1.08 | 1.05 | 409 | 279 | 251 |
| N82018 | 1.528 | 1.18 | 1.19 | 410 | 500 | 534 |
| P87024 | 1.528 | 1.21 | 1.24 | 411 | 632 | 710 |
| Y01108 | 1.528 | 1.05 | 1.05 | 412 | 253 | 246 |
| G82231 | 1.527 | 1.08 | 1.10 | 413 | 284 | 309 |
| Y02572 | 1.527 | 1.02 | 1.03 | 414 | 212 | 223 |
| H85072 | 1.527 | 1.03 | 1.02 | 415 | 222 | 200 |
| M85139 | 1.526 | 1.23 | 1.22 | 416 | 705 | 671 |
| B85042 | 1.525 | 1.08 | 1.09 | 417 | 290 | 295 |
| L81058 | 1.523 | 1.18 | 1.15 | 418 | 533 | 440 |
| M85092 | 1.523 | 1.09 | 1.08 | 419 | 320 | 298 |
| A87030 | 1.523 | 1.17 | 1.18 | 420 | 507 | 514 |
| C83085 | 1.523 | 1.14 | 1.17 | 421 | 422 | 478 |
| D82079 | 1.522 | 1.28 | 1.27 | 422 | 989 | 929 |
| N81037 | 1.522 | 1.24 | 1.25 | 423 | 760 | 776 |
| M83122 | 1.522 | 1.10 | 1.06 | 424 | 332 | 271 |
| E85681 | 1.522 | 1.00 | 1.00 | 425 | 193 | 190 |
| D83033 | 1.522 | 1.08 | 1.09 | 426 | 300 | 312 |
| P86606 | 1.521 | 1.15 | 1.16 | 427 | 437 | 465 |
| B82046 | 1.521 | 1.19 | 1.20 | 428 | 574 | 608 |
| J83006 | 1.521 | 1.04 | 1.03 | 429 | 242 | 230 |
| L84029 | 1.520 | 1.20 | 1.19 | 430 | 615 | 560 |
| P87017 | 1.520 | 1.31 | 1.33 | 431 | 1152 | 1307 |
| P92623 | 1.520 | 1.15 | 1.16 | 432 | 436 | 453 |
| P81683 | 1.519 | 1.09 | 1.12 | 433 | 326 | 386 |
| A89011 | 1.517 | 1.32 | 1.32 | 434 | 1282 | 1245 |
| L84085 | 1.517 | 1.16 | 1.15 | 435 | 488 | 447 |
| N84034 | 1.516 | 1.28 | 1.26 | 436 | 1004 | 867 |
| P84041 | 1.516 | 1.16 | 1.18 | 437 | 496 | 531 |
| P91026 | 1.515 | 1.31 | 1.30 | 438 | 1196 | 1140 |
| B87042 | 1.515 | 1.27 | 1.27 | 439 | 952 | 976 |
| M81087 | 1.515 | 1.16 | 1.18 | 440 | 505 | 528 |
| A86011 | 1.515 | 1.41 | 1.40 | 441 | 2075 | 1973 |
| P81022 | 1.514 | 1.15 | 1.18 | 442 | 470 | 558 |
| P86007 | 1.514 | 1.22 | 1.23 | 443 | 702 | 725 |
| N82633 | 1.514 | 1.30 | 1.30 | 444 | 1145 | 1132 |

| | | | | | | |
|---|---|---|---|---|---|---|
| N83045 | 1.514 | 1.25 | 1.26 | 445 | 875 | 870 |
| A81018 | 1.514 | 1.46 | 1.44 | 446 | 2726 | 2498 |
| P82008 | 1.514 | 1.32 | 1.32 | 447 | 1296 | 1290 |
| N85625 | 1.513 | 1.26 | 1.24 | 448 | 935 | 804 |
| D81061 | 1.513 | 1.19 | 1.24 | 449 | 586 | 774 |
| A83616 | 1.513 | 1.43 | 1.41 | 450 | 2321 | 2155 |
| H85070 | 1.512 | 1.11 | 1.09 | 451 | 388 | 334 |
| C82660 | 1.512 | 1.08 | 1.09 | 452 | 306 | 322 |
| F81017 | 1.511 | 1.43 | 1.42 | 453 | 2342 | 2293 |
| M85079 | 1.511 | 1.24 | 1.22 | 454 | 813 | 705 |
| P85621 | 1.511 | 1.25 | 1.26 | 455 | 862 | 948 |
| F82621 | 1.511 | 1.09 | 1.09 | 456 | 342 | 349 |
| D81603 | 1.511 | 1.25 | 1.30 | 457 | 866 | 1183 |
| M83010 | 1.511 | 1.03 | 1.00 | 458 | 249 | 202 |
| Y02610 | 1.511 | 1.00 | 1.00 | 459 | 215 | 204 |
| Y01068 | 1.511 | 1.04 | 1.03 | 460 | 255 | 248 |
| M81079 | 1.510 | 1.05 | 1.03 | 461 | 268 | 245 |
| P87025 | 1.509 | 1.10 | 1.13 | 462 | 371 | 433 |
| N82065 | 1.508 | 1.22 | 1.23 | 463 | 730 | 758 |
| C83042 | 1.508 | 1.30 | 1.32 | 464 | 1184 | 1340 |
| K82054 | 1.508 | 1.09 | 1.08 | 465 | 347 | 319 |
| A89008 | 1.507 | 1.30 | 1.29 | 466 | 1226 | 1153 |
| P82014 | 1.506 | 1.06 | 1.08 | 467 | 294 | 326 |
| J81045 | 1.505 | 1.25 | 1.24 | 468 | 868 | 821 |
| F81743 | 1.505 | 1.14 | 1.14 | 469 | 450 | 451 |
| Y02422 | 1.504 | 1.04 | 1.05 | 470 | 264 | 284 |
| N81097 | 1.503 | 1.38 | 1.35 | 471 | 1915 | 1592 |
| G82790 | 1.502 | 1.00 | 1.01 | 472 | 220 | 226 |
| M85677 | 1.502 | 1.10 | 1.09 | 473 | 382 | 362 |
| N82003 | 1.501 | 1.25 | 1.27 | 474 | 940 | 1037 |
| Y02753 | 1.501 | 1.23 | 1.24 | 475 | 833 | 891 |
| N81122 | 1.500 | 1.06 | 1.06 | 476 | 302 | 294 |
| B86092 | 1.500 | 1.21 | 1.21 | 477 | 711 | 734 |
| P81160 | 1.499 | 1.22 | 1.26 | 478 | 755 | 1017 |
| Y02701 | 1.499 | 0.98 | 1.00 | 479 | 198 | 218 |
| K84056 | 1.499 | 1.02 | 1.02 | 480 | 252 | 253 |
| A83025 | 1.499 | 1.24 | 1.25 | 481 | 890 | 937 |
| E82133 | 1.498 | 1.07 | 1.06 | 482 | 309 | 303 |
| C85614 | 1.498 | 1.21 | 1.22 | 483 | 713 | 773 |
| C85013 | 1.498 | 1.37 | 1.37 | 484 | 1907 | 1899 |
| A82017 | 1.497 | 1.24 | 1.26 | 485 | 898 | 985 |
| P86012 | 1.497 | 1.38 | 1.38 | 486 | 1934 | 1938 |
| C82100 | 1.496 | 1.24 | 1.24 | 487 | 925 | 885 |
| C82011 | 1.495 | 1.14 | 1.14 | 488 | 483 | 472 |
| M84627 | 1.494 | 1.22 | 1.22 | 489 | 820 | 772 |
| A88603 | 1.494 | 1.25 | 1.24 | 490 | 941 | 889 |
| B84019 | 1.494 | 1.11 | 1.13 | 491 | 423 | 457 |
| C86014 | 1.494 | 1.21 | 1.22 | 492 | 774 | 812 |
| G82682 | 1.494 | 1.20 | 1.20 | 493 | 695 | 706 |
| P83029 | 1.493 | 1.16 | 1.16 | 494 | 549 | 553 |

| | | | | | | |
|---|---|---|---|---|---|---|
| M84051 | 1.493 | 1.01 | 1.02 | 495 | 248 | 254 |
| Y03364 | 1.492 | 1.14 | 1.14 | 496 | 484 | 502 |
| L81051 | 1.491 | 1.18 | 1.15 | 497 | 624 | 510 |
| F81110 | 1.491 | 1.35 | 1.34 | 498 | 1711 | 1644 |
| F81707 | 1.490 | 0.98 | 0.99 | 499 | 205 | 211 |
| K82003 | 1.489 | 1.04 | 1.03 | 500 | 277 | 270 |
| F82660 | 1.488 | 1.15 | 1.15 | 501 | 537 | 540 |
| C83021 | 1.488 | 1.34 | 1.36 | 502 | 1658 | 1874 |
| G81102 | 1.488 | 1.01 | 0.99 | 503 | 244 | 220 |
| N83609 | 1.487 | 1.23 | 1.23 | 504 | 882 | 871 |
| M86607 | 1.487 | 1.16 | 1.15 | 505 | 593 | 565 |
| M83631 | 1.487 | 1.28 | 1.31 | 506 | 1203 | 1457 |
| M82058 | 1.486 | 1.14 | 1.14 | 507 | 523 | 505 |
| E85061 | 1.486 | 1.10 | 1.10 | 508 | 406 | 410 |
| P87019 | 1.485 | 1.25 | 1.27 | 509 | 1030 | 1149 |
| A81049 | 1.485 | 1.26 | 1.27 | 510 | 1095 | 1151 |
| P82627 | 1.485 | 1.08 | 1.08 | 511 | 363 | 370 |
| C86025 | 1.484 | 1.27 | 1.28 | 512 | 1143 | 1260 |
| C87013 | 1.484 | 1.27 | 1.26 | 513 | 1132 | 1111 |
| L85061 | 1.484 | 1.15 | 1.14 | 514 | 554 | 527 |
| C84688 | 1.484 | 1.19 | 1.21 | 515 | 726 | 789 |
| M88023 | 1.484 | 1.15 | 1.16 | 516 | 566 | 595 |
| M88003 | 1.483 | 1.34 | 1.35 | 517 | 1750 | 1812 |
| B87016 | 1.483 | 1.27 | 1.28 | 518 | 1188 | 1263 |
| G81008 | 1.483 | 1.18 | 1.15 | 519 | 686 | 579 |
| P92021 | 1.483 | 1.26 | 1.27 | 520 | 1129 | 1146 |
| B83021 | 1.482 | 1.26 | 1.26 | 521 | 1085 | 1121 |
| M85794 | 1.482 | 1.09 | 1.08 | 522 | 402 | 381 |
| G82639 | 1.482 | 1.06 | 1.06 | 523 | 330 | 341 |
| G81105 | 1.482 | 1.21 | 1.24 | 524 | 802 | 961 |
| B87007 | 1.482 | 1.14 | 1.16 | 525 | 529 | 606 |
| M91009 | 1.481 | 1.35 | 1.34 | 526 | 1806 | 1780 |
| F84713 | 1.481 | 0.96 | 0.95 | 527 | 189 | 182 |
| M85061 | 1.480 | 1.15 | 1.14 | 528 | 569 | 537 |
| C84087 | 1.480 | 1.24 | 1.22 | 529 | 998 | 857 |
| M86044 | 1.480 | 1.11 | 1.10 | 530 | 456 | 416 |
| C85024 | 1.479 | 1.12 | 1.14 | 531 | 469 | 530 |
| B81040 | 1.479 | 1.18 | 1.22 | 532 | 692 | 887 |
| N85031 | 1.479 | 1.30 | 1.27 | 533 | 1415 | 1229 |
| M84608 | 1.479 | 1.22 | 1.21 | 534 | 873 | 796 |
| B82106 | 1.478 | 1.34 | 1.35 | 535 | 1748 | 1912 |
| B81620 | 1.478 | 1.15 | 1.18 | 536 | 587 | 673 |
| C82656 | 1.478 | 1.20 | 1.20 | 537 | 785 | 742 |
| M88612 | 1.478 | 1.15 | 1.16 | 538 | 577 | 618 |
| Y02501 | 1.477 | 1.12 | 1.16 | 539 | 495 | 612 |
| A83634 | 1.477 | 1.43 | 1.43 | 540 | 2782 | 2754 |
| P85006 | 1.477 | 1.08 | 1.11 | 541 | 398 | 438 |
| A89620 | 1.477 | 1.34 | 1.34 | 542 | 1814 | 1803 |
| B83054 | 1.477 | 1.29 | 1.28 | 543 | 1345 | 1275 |
| D82004 | 1.476 | 1.30 | 1.27 | 544 | 1413 | 1179 |

| | | | | | | |
|---|---|---|---|---|---|---|
| M83668 | 1.476 | 1.02 | 0.99 | 545 | 278 | 236 |
| A81021 | 1.476 | 1.16 | 1.16 | 546 | 604 | 633 |
| P85012 | 1.476 | 1.10 | 1.12 | 547 | 434 | 480 |
| N82671 | 1.475 | 1.16 | 1.17 | 548 | 626 | 659 |
| A83618 | 1.475 | 1.27 | 1.27 | 549 | 1237 | 1252 |
| L83029 | 1.475 | 1.14 | 1.14 | 550 | 543 | 561 |
| P81748 | 1.475 | 1.14 | 1.13 | 551 | 542 | 501 |
| G82114 | 1.475 | 1.10 | 1.10 | 552 | 443 | 422 |
| M82009 | 1.474 | 1.19 | 1.19 | 553 | 736 | 724 |
| B81053 | 1.474 | 1.25 | 1.30 | 554 | 1110 | 1426 |
| M89606 | 1.474 | 0.96 | 0.94 | 555 | 209 | 177 |
| N84014 | 1.474 | 1.32 | 1.31 | 556 | 1612 | 1497 |
| P85615 | 1.474 | 1.20 | 1.22 | 557 | 825 | 925 |
| L83022 | 1.473 | 1.19 | 1.18 | 558 | 766 | 709 |
| P86614 | 1.473 | 1.33 | 1.36 | 559 | 1742 | 1985 |
| D82105 | 1.473 | 1.19 | 1.19 | 560 | 771 | 741 |
| M81069 | 1.473 | 1.08 | 1.06 | 561 | 405 | 350 |
| P82009 | 1.473 | 1.20 | 1.22 | 562 | 827 | 893 |
| N81019 | 1.472 | 1.39 | 1.40 | 563 | 2391 | 2448 |
| M84064 | 1.472 | 1.05 | 1.05 | 564 | 339 | 331 |
| F84673 | 1.471 | 0.85 | 0.84 | 565 | 122 | 115 |
| L81083 | 1.471 | 1.27 | 1.26 | 566 | 1230 | 1164 |
| P86005 | 1.471 | 1.11 | 1.12 | 567 | 468 | 504 |
| F82023 | 1.470 | 1.09 | 1.08 | 568 | 415 | 396 |
| A81026 | 1.470 | 1.21 | 1.23 | 569 | 880 | 972 |
| P92010 | 1.470 | 1.14 | 1.15 | 570 | 564 | 599 |
| D81059 | 1.470 | 1.26 | 1.24 | 571 | 1177 | 1081 |
| C83006 | 1.470 | 1.29 | 1.31 | 572 | 1433 | 1552 |
| F86624 | 1.470 | 1.03 | 1.02 | 573 | 299 | 279 |
| E83042 | 1.470 | 1.17 | 1.13 | 574 | 667 | 519 |
| P84054 | 1.470 | 1.20 | 1.21 | 575 | 811 | 882 |
| M84019 | 1.469 | 1.14 | 1.09 | 576 | 565 | 423 |
| M85634 | 1.468 | 1.18 | 1.17 | 577 | 722 | 687 |
| P81118 | 1.468 | 1.12 | 1.12 | 578 | 508 | 511 |
| F81185 | 1.467 | 0.99 | 0.99 | 579 | 250 | 250 |
| P81166 | 1.467 | 1.27 | 1.32 | 580 | 1307 | 1678 |
| N81119 | 1.467 | 1.25 | 1.25 | 581 | 1113 | 1107 |
| B85012 | 1.467 | 1.12 | 1.12 | 582 | 516 | 500 |
| P84672 | 1.466 | 1.06 | 1.08 | 583 | 376 | 409 |
| M83722 | 1.466 | 1.09 | 1.13 | 584 | 435 | 524 |
| N82011 | 1.466 | 1.15 | 1.16 | 585 | 613 | 668 |
| G82128 | 1.466 | 1.13 | 1.14 | 586 | 558 | 598 |
| P85028 | 1.465 | 1.42 | 1.43 | 587 | 2795 | 2880 |
| A81037 | 1.465 | 1.17 | 1.18 | 588 | 729 | 749 |
| G82710 | 1.465 | 1.07 | 1.07 | 589 | 401 | 389 |
| C83613 | 1.464 | 1.18 | 1.18 | 590 | 753 | 722 |
| F81150 | 1.464 | 1.07 | 1.07 | 591 | 397 | 390 |
| N81056 | 1.464 | 1.21 | 1.19 | 592 | 877 | 780 |
| C83039 | 1.463 | 1.30 | 1.30 | 593 | 1561 | 1514 |
| M85076 | 1.463 | 1.26 | 1.23 | 594 | 1210 | 1061 |

| | | | | | | |
|---|---|---|---|---|---|---|
| G85727 | 1.463 | 0.95 | 0.96 | 595 | 204 | 213 |
| C85018 | 1.463 | 1.28 | 1.28 | 596 | 1376 | 1388 |
| A83018 | 1.463 | 1.23 | 1.24 | 597 | 1072 | 1110 |
| P84066 | 1.462 | 1.17 | 1.19 | 598 | 734 | 816 |
| N81123 | 1.462 | 1.12 | 1.09 | 599 | 520 | 431 |
| M92019 | 1.461 | 1.13 | 1.14 | 600 | 563 | 603 |
| A86017 | 1.461 | 1.29 | 1.28 | 601 | 1439 | 1435 |
| N83022 | 1.460 | 1.35 | 1.35 | 602 | 2007 | 2028 |
| H83006 | 1.460 | 1.05 | 1.03 | 603 | 358 | 317 |
| G82757 | 1.459 | 0.93 | 0.95 | 604 | 184 | 205 |
| A81065 | 1.459 | 1.30 | 1.29 | 605 | 1535 | 1460 |
| M83057 | 1.459 | 1.04 | 1.03 | 606 | 352 | 320 |
| P86016 | 1.458 | 1.27 | 1.27 | 607 | 1310 | 1378 |
| B85645 | 1.458 | 1.23 | 1.23 | 608 | 1050 | 1089 |
| D81052 | 1.458 | 1.30 | 1.35 | 609 | 1610 | 2039 |
| Y00446 | 1.458 | 1.11 | 1.11 | 610 | 525 | 496 |
| N82663 | 1.458 | 1.19 | 1.20 | 611 | 853 | 897 |
| M85783 | 1.458 | 1.11 | 1.11 | 612 | 527 | 494 |
| M83063 | 1.457 | 1.14 | 1.18 | 613 | 630 | 785 |
| P83027 | 1.457 | 1.04 | 1.05 | 614 | 345 | 369 |
| K82069 | 1.457 | 1.01 | 0.99 | 615 | 283 | 262 |
| C85617 | 1.457 | 1.16 | 1.18 | 616 | 700 | 761 |
| A86040 | 1.457 | 1.23 | 1.23 | 617 | 1092 | 1096 |
| G82103 | 1.456 | 1.00 | 1.00 | 618 | 271 | 269 |
| C84096 | 1.456 | 1.16 | 1.19 | 619 | 718 | 832 |
| P82021 | 1.455 | 1.11 | 1.12 | 620 | 513 | 552 |
| A86025 | 1.455 | 1.18 | 1.19 | 621 | 803 | 822 |
| C88069 | 1.455 | 1.18 | 1.20 | 622 | 812 | 866 |
| P92615 | 1.454 | 1.19 | 1.20 | 623 | 840 | 902 |
| N82060 | 1.454 | 1.42 | 1.41 | 624 | 2937 | 2787 |
| E86027 | 1.454 | 1.05 | 1.04 | 625 | 372 | 344 |
| M87625 | 1.454 | 1.24 | 1.23 | 626 | 1154 | 1094 |
| Y02627 | 1.453 | 1.14 | 1.15 | 627 | 614 | 666 |
| D81039 | 1.452 | 1.16 | 1.20 | 628 | 696 | 917 |
| G82700 | 1.452 | 1.30 | 1.29 | 629 | 1656 | 1556 |
| G82686 | 1.452 | 1.18 | 1.18 | 630 | 791 | 818 |
| P81749 | 1.451 | 0.98 | 1.03 | 631 | 260 | 333 |
| H85022 | 1.451 | 1.01 | 1.00 | 632 | 292 | 277 |
| G82141 | 1.451 | 1.07 | 1.07 | 633 | 428 | 425 |
| A83021 | 1.451 | 1.31 | 1.31 | 634 | 1749 | 1700 |
| M85031 | 1.450 | 1.18 | 1.17 | 635 | 841 | 756 |
| J82085 | 1.450 | 1.19 | 1.20 | 636 | 885 | 935 |
| B87012 | 1.449 | 1.10 | 1.12 | 637 | 494 | 568 |
| M83005 | 1.448 | 1.22 | 1.22 | 638 | 1054 | 1058 |
| M81076 | 1.448 | 1.02 | 1.02 | 639 | 328 | 306 |
| G81084 | 1.448 | 1.21 | 1.24 | 640 | 1043 | 1209 |
| P84033 | 1.447 | 1.14 | 1.15 | 641 | 642 | 696 |
| P83007 | 1.447 | 1.10 | 1.11 | 642 | 522 | 529 |
| P81190 | 1.447 | 1.15 | 1.15 | 643 | 697 | 684 |
| A87029 | 1.446 | 1.37 | 1.37 | 644 | 2454 | 2449 |

| | | | | | | |
|---|---|---|---|---|---|---|
| C82064 | 1.446 | 1.19 | 1.20 | 645 | 905 | 924 |
| C84014 | 1.446 | 1.10 | 1.12 | 646 | 526 | 597 |
| C81647 | 1.446 | 1.30 | 1.28 | 647 | 1659 | 1553 |
| H81672 | 1.445 | 0.91 | 0.90 | 648 | 178 | 170 |
| G82721 | 1.444 | 1.10 | 1.13 | 649 | 521 | 615 |
| P84020 | 1.444 | 1.09 | 1.11 | 650 | 504 | 548 |
| M85018 | 1.444 | 1.30 | 1.27 | 651 | 1701 | 1479 |
| P81201 | 1.443 | 1.09 | 1.10 | 652 | 498 | 512 |
| M88015 | 1.443 | 1.08 | 1.10 | 653 | 472 | 509 |
| B82035 | 1.443 | 1.03 | 1.03 | 654 | 356 | 346 |
| F81675 | 1.442 | 1.12 | 1.09 | 655 | 578 | 477 |
| M84049 | 1.442 | 1.12 | 1.12 | 656 | 579 | 585 |
| P81119 | 1.442 | 1.13 | 1.12 | 657 | 637 | 590 |
| M86023 | 1.441 | 0.99 | 0.99 | 658 | 281 | 281 |
| A83020 | 1.441 | 1.24 | 1.25 | 659 | 1278 | 1344 |
| P81771 | 1.441 | 1.18 | 1.21 | 660 | 894 | 1062 |
| E81612 | 1.440 | 1.05 | 1.06 | 661 | 410 | 427 |
| P92042 | 1.440 | 1.35 | 1.35 | 662 | 2280 | 2275 |
| B82022 | 1.440 | 1.10 | 1.11 | 663 | 539 | 555 |
| B81072 | 1.439 | 1.05 | 1.10 | 664 | 411 | 526 |
| F81741 | 1.439 | 1.33 | 1.34 | 665 | 2104 | 2114 |
| L83008 | 1.439 | 1.25 | 1.26 | 666 | 1343 | 1415 |
| A87006 | 1.439 | 1.07 | 1.09 | 667 | 451 | 493 |
| L83088 | 1.438 | 1.16 | 1.16 | 668 | 801 | 793 |
| C87617 | 1.438 | 1.34 | 1.33 | 669 | 2161 | 2019 |
| M81092 | 1.438 | 1.06 | 1.09 | 670 | 429 | 490 |
| B81081 | 1.437 | 1.16 | 1.21 | 671 | 779 | 1059 |
| F86707 | 1.437 | 1.01 | 0.99 | 672 | 322 | 290 |
| M83706 | 1.436 | 1.14 | 1.13 | 673 | 712 | 663 |
| J81648 | 1.436 | 1.05 | 1.05 | 674 | 408 | 415 |
| F81049 | 1.436 | 1.06 | 1.05 | 675 | 444 | 393 |
| C81655 | 1.435 | 1.25 | 1.24 | 676 | 1379 | 1328 |
| E87751 | 1.435 | 1.17 | 1.16 | 677 | 837 | 766 |
| P81171 | 1.435 | 1.10 | 1.07 | 678 | 540 | 461 |
| F81177 | 1.435 | 1.07 | 1.07 | 679 | 461 | 463 |
| F82675 | 1.435 | 0.97 | 0.98 | 680 | 265 | 272 |
| P84019 | 1.435 | 1.23 | 1.24 | 681 | 1249 | 1321 |
| M85642 | 1.434 | 1.16 | 1.16 | 682 | 818 | 771 |
| C88020 | 1.434 | 1.41 | 1.40 | 683 | 3088 | 3012 |
| G81096 | 1.434 | 1.10 | 1.13 | 684 | 561 | 677 |
| P91003 | 1.434 | 1.03 | 1.05 | 685 | 389 | 407 |
| P84067 | 1.433 | 1.13 | 1.14 | 686 | 658 | 715 |
| J82021 | 1.433 | 1.21 | 1.18 | 687 | 1089 | 909 |
| L81099 | 1.433 | 1.23 | 1.23 | 688 | 1231 | 1213 |
| B81015 | 1.433 | 1.18 | 1.20 | 689 | 886 | 1073 |
| A83066 | 1.432 | 1.23 | 1.23 | 690 | 1253 | 1270 |
| M89008 | 1.432 | 1.28 | 1.22 | 691 | 1652 | 1200 |
| B86054 | 1.431 | 1.38 | 1.38 | 692 | 2731 | 2669 |
| Y02461 | 1.431 | 1.14 | 1.16 | 693 | 708 | 792 |
| G82086 | 1.431 | 1.16 | 1.18 | 694 | 810 | 953 |

| | | | | | | |
|---|---|---|---|---|---|---|
| P92633 | 1.431 | 1.18 | 1.19 | 695 | 909 | 991 |
| M87012 | 1.431 | 1.11 | 1.10 | 696 | 591 | 577 |
| G82706 | 1.430 | 1.01 | 1.04 | 697 | 338 | 391 |
| G82729 | 1.430 | 1.13 | 1.13 | 698 | 663 | 694 |
| P87618 | 1.430 | 1.40 | 1.40 | 699 | 2974 | 3054 |
| N81012 | 1.430 | 1.19 | 1.18 | 700 | 1017 | 915 |
| C82091 | 1.429 | 1.15 | 1.15 | 701 | 770 | 745 |
| B87026 | 1.429 | 1.40 | 1.41 | 702 | 3066 | 3135 |
| F82627 | 1.428 | 1.02 | 1.01 | 703 | 360 | 347 |
| A89020 | 1.428 | 1.28 | 1.29 | 704 | 1679 | 1723 |
| C84647 | 1.428 | 1.23 | 1.25 | 705 | 1265 | 1436 |
| H85054 | 1.427 | 1.04 | 1.03 | 706 | 417 | 382 |
| B86050 | 1.427 | 1.07 | 1.08 | 707 | 473 | 503 |
| P85004 | 1.427 | 1.18 | 1.20 | 708 | 964 | 1080 |
| B87023 | 1.427 | 1.19 | 1.21 | 709 | 1006 | 1118 |
| B82086 | 1.427 | 1.01 | 1.03 | 710 | 359 | 380 |
| G83641 | 1.426 | 1.09 | 1.09 | 711 | 556 | 556 |
| A81063 | 1.426 | 1.26 | 1.28 | 712 | 1512 | 1698 |
| N81054 | 1.426 | 1.19 | 1.20 | 713 | 999 | 1067 |
| F81011 | 1.426 | 1.07 | 1.07 | 714 | 491 | 470 |
| N85619 | 1.426 | 1.26 | 1.23 | 715 | 1474 | 1314 |
| B87011 | 1.426 | 1.29 | 1.30 | 716 | 1769 | 1868 |
| P81622 | 1.425 | 1.14 | 1.18 | 717 | 761 | 945 |
| A81613 | 1.425 | 1.11 | 1.15 | 718 | 640 | 795 |
| P92026 | 1.425 | 1.16 | 1.16 | 719 | 836 | 855 |
| B87033 | 1.424 | 1.28 | 1.29 | 720 | 1673 | 1801 |
| P81707 | 1.424 | 1.20 | 1.23 | 721 | 1119 | 1332 |
| M82039 | 1.424 | 1.16 | 1.17 | 722 | 846 | 888 |
| M85770 | 1.424 | 1.17 | 1.15 | 723 | 892 | 794 |
| N81011 | 1.424 | 1.15 | 1.16 | 724 | 790 | 834 |
| A83019 | 1.423 | 1.32 | 1.31 | 725 | 2102 | 2015 |
| C86614 | 1.423 | 1.22 | 1.24 | 726 | 1225 | 1377 |
| N84024 | 1.422 | 1.17 | 1.17 | 727 | 920 | 916 |
| N85648 | 1.421 | 1.18 | 1.17 | 728 | 1020 | 944 |
| M83717 | 1.421 | 1.02 | 1.06 | 729 | 386 | 458 |
| C83079 | 1.421 | 1.13 | 1.18 | 730 | 738 | 964 |
| L84068 | 1.421 | 1.15 | 1.13 | 731 | 795 | 700 |
| F82011 | 1.421 | 1.06 | 1.06 | 732 | 475 | 450 |
| A81030 | 1.420 | 1.10 | 1.12 | 733 | 596 | 674 |
| D81029 | 1.420 | 1.06 | 1.07 | 734 | 476 | 483 |
| D81605 | 1.420 | 1.27 | 1.27 | 735 | 1688 | 1703 |
| G85717 | 1.420 | 0.94 | 0.95 | 736 | 245 | 261 |
| M83021 | 1.419 | 1.27 | 1.30 | 737 | 1622 | 1922 |
| C83019 | 1.419 | 1.53 | 1.54 | 738 | 4980 | 5017 |
| J84011 | 1.419 | 1.04 | 1.05 | 739 | 433 | 436 |
| A81057 | 1.419 | 1.15 | 1.15 | 740 | 809 | 806 |
| E81023 | 1.419 | 1.04 | 1.04 | 741 | 438 | 414 |
| P92652 | 1.419 | 1.22 | 1.23 | 742 | 1275 | 1313 |
| P81690 | 1.419 | 1.38 | 1.37 | 743 | 2855 | 2705 |
| B85641 | 1.418 | 1.21 | 1.21 | 744 | 1193 | 1208 |

| | | | | | | |
|---|---|---|---|---|---|---|
| A83017 | 1.418 | 1.38 | 1.37 | 745 | 2916 | 2756 |
| M83062 | 1.418 | 1.12 | 1.12 | 746 | 693 | 675 |
| D83008 | 1.418 | 1.04 | 1.05 | 747 | 427 | 445 |
| A82032 | 1.418 | 1.37 | 1.36 | 748 | 2737 | 2636 |
| A83075 | 1.417 | 1.26 | 1.26 | 749 | 1563 | 1538 |
| Y02521 | 1.417 | 1.11 | 1.15 | 750 | 669 | 827 |
| G82119 | 1.417 | 1.16 | 1.16 | 751 | 864 | 879 |
| D83060 | 1.417 | 1.10 | 1.07 | 752 | 616 | 497 |
| C82026 | 1.417 | 1.07 | 1.08 | 753 | 519 | 520 |
| B83007 | 1.417 | 1.11 | 1.13 | 754 | 649 | 723 |
| C86033 | 1.417 | 1.33 | 1.34 | 755 | 2313 | 2434 |
| B83005 | 1.416 | 1.07 | 1.08 | 756 | 515 | 550 |
| J81071 | 1.416 | 1.15 | 1.15 | 757 | 823 | 810 |
| B82064 | 1.415 | 1.08 | 1.07 | 758 | 551 | 522 |
| H81126 | 1.415 | 0.96 | 0.95 | 759 | 276 | 266 |
| M83004 | 1.415 | 1.19 | 1.24 | 760 | 1115 | 1424 |
| M85051 | 1.415 | 1.17 | 1.16 | 761 | 939 | 910 |
| C86024 | 1.414 | 1.24 | 1.26 | 762 | 1434 | 1603 |
| F82676 | 1.414 | 1.08 | 1.08 | 763 | 555 | 572 |
| M83022 | 1.414 | 1.06 | 1.05 | 764 | 510 | 462 |
| H85693 | 1.413 | 1.03 | 1.02 | 765 | 421 | 387 |
| P85005 | 1.413 | 1.11 | 1.13 | 766 | 671 | 763 |
| C85622 | 1.413 | 1.26 | 1.26 | 767 | 1609 | 1642 |
| Y03597 | 1.413 | 1.18 | 1.17 | 768 | 1036 | 993 |
| L83072 | 1.413 | 1.11 | 1.14 | 769 | 688 | 775 |
| C85030 | 1.413 | 1.24 | 1.25 | 770 | 1481 | 1557 |
| P92006 | 1.413 | 1.28 | 1.28 | 771 | 1860 | 1850 |
| H82033 | 1.412 | 0.99 | 1.00 | 772 | 335 | 363 |
| N85017 | 1.412 | 1.20 | 1.18 | 773 | 1179 | 1072 |
| P86609 | 1.412 | 1.10 | 1.12 | 774 | 643 | 718 |
| Y00683 | 1.412 | 1.41 | 1.41 | 775 | 3392 | 3367 |
| C83015 | 1.412 | 1.17 | 1.17 | 776 | 1019 | 966 |
| Y02754 | 1.411 | 0.89 | 0.88 | 777 | 188 | 185 |
| M85128 | 1.411 | 1.27 | 1.25 | 778 | 1706 | 1573 |
| N82623 | 1.411 | 1.28 | 1.28 | 779 | 1793 | 1799 |
| A81008 | 1.410 | 1.43 | 1.41 | 780 | 3695 | 3391 |
| K81048 | 1.410 | 0.99 | 0.99 | 781 | 336 | 336 |
| B85636 | 1.410 | 1.04 | 1.05 | 782 | 459 | 468 |
| Y01652 | 1.410 | 1.28 | 1.26 | 783 | 1856 | 1674 |
| B85652 | 1.410 | 1.24 | 1.24 | 784 | 1445 | 1475 |
| F82034 | 1.410 | 0.97 | 0.98 | 785 | 296 | 313 |
| F81608 | 1.410 | 1.12 | 1.09 | 786 | 727 | 619 |
| A81052 | 1.409 | 1.27 | 1.27 | 787 | 1753 | 1776 |
| G82197 | 1.409 | 0.98 | 0.99 | 788 | 327 | 339 |
| F81096 | 1.409 | 1.22 | 1.20 | 789 | 1321 | 1204 |
| C84658 | 1.409 | 1.32 | 1.33 | 790 | 2240 | 2464 |
| B84623 | 1.409 | 1.04 | 1.05 | 791 | 439 | 473 |
| H82088 | 1.409 | 1.06 | 1.07 | 792 | 503 | 545 |
| N85016 | 1.409 | 1.18 | 1.16 | 793 | 1058 | 942 |
| L84070 | 1.409 | 1.13 | 1.12 | 794 | 759 | 717 |

| | | | | | | |
|---|---|---|---|---|---|---|
| D81011 | 1.409 | 1.22 | 1.26 | 795 | 1334 | 1707 |
| H81022 | 1.408 | 1.04 | 1.04 | 796 | 457 | 435 |
| K82059 | 1.408 | 1.09 | 1.08 | 797 | 595 | 563 |
| N84621 | 1.408 | 1.33 | 1.31 | 798 | 2455 | 2224 |
| G81055 | 1.408 | 1.10 | 1.08 | 799 | 654 | 580 |
| F81082 | 1.407 | 1.18 | 1.18 | 800 | 1066 | 1075 |
| C88077 | 1.407 | 1.23 | 1.24 | 801 | 1409 | 1474 |
| B84010 | 1.407 | 1.15 | 1.17 | 802 | 906 | 1007 |
| M83070 | 1.407 | 1.02 | 1.02 | 803 | 420 | 398 |
| N81007 | 1.406 | 1.11 | 1.11 | 804 | 715 | 686 |
| F81022 | 1.406 | 1.05 | 1.04 | 805 | 499 | 446 |
| B81046 | 1.406 | 1.27 | 1.31 | 806 | 1825 | 2266 |
| A83603 | 1.406 | 1.32 | 1.32 | 807 | 2356 | 2309 |
| A81053 | 1.405 | 1.22 | 1.22 | 808 | 1398 | 1368 |
| C86001 | 1.405 | 1.35 | 1.35 | 809 | 2636 | 2729 |
| C81056 | 1.405 | 1.37 | 1.36 | 810 | 2956 | 2844 |
| H81011 | 1.405 | 1.00 | 0.99 | 811 | 384 | 343 |
| B85016 | 1.405 | 1.13 | 1.13 | 812 | 783 | 799 |
| N81095 | 1.404 | 1.21 | 1.18 | 813 | 1277 | 1077 |
| D82053 | 1.404 | 1.66 | 1.60 | 814 | 6429 | 5847 |
| K82068 | 1.404 | 1.03 | 1.01 | 815 | 452 | 383 |
| P81016 | 1.404 | 1.31 | 1.35 | 816 | 2248 | 2650 |
| C88087 | 1.404 | 1.37 | 1.36 | 817 | 2962 | 2866 |
| G82057 | 1.404 | 1.26 | 1.26 | 818 | 1703 | 1722 |
| G83655 | 1.404 | 1.11 | 1.10 | 819 | 703 | 655 |
| M85116 | 1.403 | 1.14 | 1.13 | 820 | 863 | 790 |
| B81034 | 1.403 | 1.14 | 1.14 | 821 | 844 | 849 |
| A81066 | 1.403 | 1.12 | 1.13 | 822 | 773 | 805 |
| B81003 | 1.403 | 1.19 | 1.22 | 823 | 1151 | 1346 |
| B81682 | 1.403 | 1.22 | 1.27 | 824 | 1401 | 1806 |
| M87628 | 1.403 | 1.03 | 1.04 | 825 | 447 | 444 |
| A81003 | 1.403 | 1.25 | 1.27 | 826 | 1640 | 1863 |
| A83032 | 1.403 | 1.45 | 1.46 | 827 | 4038 | 4198 |
| C84138 | 1.402 | 1.08 | 1.10 | 828 | 580 | 667 |
| D83017 | 1.402 | 1.08 | 1.08 | 829 | 581 | 617 |
| P88044 | 1.402 | 0.99 | 1.00 | 830 | 370 | 374 |
| C85017 | 1.401 | 1.40 | 1.40 | 831 | 3372 | 3382 |
| L82044 | 1.401 | 1.34 | 1.33 | 832 | 2559 | 2552 |
| F81168 | 1.401 | 1.16 | 1.16 | 833 | 993 | 963 |
| N84021 | 1.401 | 1.11 | 1.11 | 834 | 706 | 708 |
| Y01851 | 1.401 | 1.10 | 1.10 | 835 | 698 | 695 |
| D81062 | 1.401 | 1.11 | 1.10 | 836 | 733 | 682 |
| K83033 | 1.400 | 1.10 | 1.11 | 837 | 680 | 707 |
| M86045 | 1.400 | 1.27 | 1.26 | 838 | 1824 | 1757 |
| N85027 | 1.399 | 1.17 | 1.16 | 839 | 1080 | 994 |
| F81051 | 1.398 | 1.22 | 1.20 | 840 | 1403 | 1306 |
| M83682 | 1.398 | 1.26 | 1.29 | 841 | 1724 | 2045 |
| E82042 | 1.397 | 1.08 | 1.07 | 842 | 606 | 589 |
| A89017 | 1.397 | 1.17 | 1.18 | 843 | 1097 | 1148 |
| P81770 | 1.396 | 0.94 | 0.94 | 844 | 274 | 278 |

| | | | | | | |
|---|---|---|---|---|---|---|
| C83063 | 1.396 | 1.31 | 1.30 | 845 | 2286 | 2175 |
| A81060 | 1.396 | 1.16 | 1.20 | 846 | 1035 | 1264 |
| A82075 | 1.396 | 1.22 | 1.25 | 847 | 1444 | 1702 |
| A89003 | 1.395 | 1.21 | 1.22 | 848 | 1391 | 1464 |
| E85056 | 1.395 | 1.03 | 1.01 | 849 | 454 | 412 |
| M83032 | 1.394 | 1.14 | 1.13 | 850 | 876 | 842 |
| C83026 | 1.394 | 1.16 | 1.15 | 851 | 1059 | 970 |
| G82115 | 1.394 | 0.99 | 1.00 | 852 | 391 | 394 |
| P81008 | 1.394 | 1.25 | 1.26 | 853 | 1716 | 1821 |
| C83605 | 1.394 | 1.17 | 1.19 | 854 | 1074 | 1233 |
| L84052 | 1.394 | 1.17 | 1.16 | 855 | 1105 | 1014 |
| D81057 | 1.393 | 1.08 | 1.07 | 856 | 618 | 602 |
| F82004 | 1.393 | 1.10 | 1.11 | 857 | 724 | 728 |
| B81038 | 1.393 | 1.31 | 1.35 | 858 | 2348 | 2806 |
| C84637 | 1.393 | 1.16 | 1.19 | 859 | 1028 | 1199 |
| G83033 | 1.392 | 1.06 | 1.06 | 860 | 571 | 546 |
| C82631 | 1.392 | 0.99 | 0.99 | 861 | 373 | 366 |
| K84010 | 1.392 | 1.05 | 1.03 | 862 | 545 | 460 |
| C88073 | 1.392 | 1.20 | 1.21 | 863 | 1297 | 1374 |
| E82078 | 1.392 | 1.09 | 1.09 | 864 | 682 | 679 |
| P91032 | 1.392 | 1.03 | 1.04 | 865 | 458 | 479 |
| B82032 | 1.391 | 1.15 | 1.14 | 866 | 990 | 918 |
| F81669 | 1.391 | 1.08 | 1.07 | 867 | 620 | 621 |
| M88036 | 1.391 | 1.11 | 1.13 | 868 | 777 | 854 |
| C86013 | 1.390 | 1.19 | 1.22 | 869 | 1268 | 1473 |
| A83052 | 1.390 | 1.26 | 1.27 | 870 | 1838 | 1908 |
| P81074 | 1.390 | 1.32 | 1.36 | 871 | 2537 | 3069 |
| G82225 | 1.390 | 1.15 | 1.13 | 872 | 1025 | 863 |
| D83020 | 1.390 | 1.05 | 1.02 | 873 | 538 | 449 |
| N82078 | 1.390 | 1.32 | 1.32 | 874 | 2536 | 2504 |
| H84049 | 1.390 | 1.05 | 1.03 | 875 | 548 | 481 |
| K82040 | 1.389 | 1.09 | 1.07 | 876 | 691 | 594 |
| Y00592 | 1.389 | 1.31 | 1.34 | 877 | 2332 | 2700 |
| N81023 | 1.389 | 1.19 | 1.16 | 878 | 1250 | 1066 |
| G81641 | 1.389 | 1.03 | 1.06 | 879 | 464 | 583 |
| B86055 | 1.389 | 1.20 | 1.22 | 880 | 1375 | 1472 |
| N81061 | 1.389 | 1.15 | 1.13 | 881 | 1012 | 840 |
| A81020 | 1.389 | 1.16 | 1.18 | 882 | 1048 | 1162 |
| P87630 | 1.388 | 1.28 | 1.30 | 883 | 2129 | 2288 |
| A83076 | 1.388 | 1.37 | 1.36 | 884 | 3116 | 2990 |
| M84615 | 1.388 | 1.11 | 1.11 | 885 | 764 | 747 |
| M83014 | 1.388 | 1.19 | 1.22 | 886 | 1251 | 1486 |
| F85650 | 1.387 | 1.08 | 1.08 | 887 | 645 | 638 |
| G82605 | 1.387 | 1.06 | 1.07 | 888 | 582 | 600 |
| B84613 | 1.387 | 1.26 | 1.27 | 889 | 1917 | 1965 |
| L84011 | 1.386 | 1.15 | 1.14 | 890 | 1018 | 949 |
| P85013 | 1.386 | 1.08 | 1.10 | 891 | 655 | 743 |
| B87005 | 1.386 | 1.25 | 1.27 | 892 | 1790 | 1942 |
| C82120 | 1.386 | 1.12 | 1.11 | 893 | 861 | 802 |
| P87625 | 1.386 | 1.29 | 1.30 | 894 | 2189 | 2381 |

| | | | | | | |
|---|---|---|---|---|---|---|
| J82086 | 1.385 | 1.34 | 1.33 | 895 | 2763 | 2680 |
| D81018 | 1.385 | 1.05 | 1.04 | 896 | 553 | 499 |
| C88030 | 1.385 | 1.25 | 1.25 | 897 | 1759 | 1847 |
| Y00399 | 1.385 | 1.23 | 1.26 | 898 | 1581 | 1867 |
| E85120 | 1.385 | 0.95 | 0.96 | 899 | 313 | 318 |
| Y01118 | 1.385 | 1.11 | 1.12 | 900 | 817 | 856 |
| K81086 | 1.385 | 1.24 | 1.26 | 901 | 1725 | 1873 |
| F81055 | 1.384 | 0.97 | 0.93 | 902 | 346 | 276 |
| G82050 | 1.384 | 1.02 | 1.01 | 903 | 460 | 441 |
| F82053 | 1.384 | 0.97 | 0.97 | 904 | 355 | 358 |
| P88014 | 1.384 | 1.19 | 1.19 | 905 | 1335 | 1281 |
| G82093 | 1.383 | 1.13 | 1.13 | 906 | 887 | 895 |
| M82020 | 1.383 | 1.06 | 1.05 | 907 | 575 | 573 |
| E82012 | 1.383 | 1.02 | 1.03 | 908 | 471 | 476 |
| P88620 | 1.383 | 1.08 | 1.08 | 909 | 668 | 676 |
| F81126 | 1.383 | 1.17 | 1.16 | 910 | 1194 | 1101 |
| F85059 | 1.383 | 1.17 | 1.17 | 911 | 1142 | 1137 |
| Y00164 | 1.383 | 1.14 | 1.15 | 912 | 970 | 1036 |
| G85008 | 1.383 | 1.02 | 1.03 | 913 | 478 | 482 |
| B83045 | 1.382 | 1.16 | 1.18 | 914 | 1124 | 1201 |
| G81063 | 1.382 | 1.13 | 1.14 | 915 | 928 | 1009 |
| E82090 | 1.382 | 1.02 | 1.03 | 916 | 480 | 475 |
| Y02676 | 1.382 | 0.77 | 0.79 | 917 | 131 | 146 |
| P85622 | 1.382 | 1.20 | 1.22 | 918 | 1385 | 1587 |
| C84032 | 1.382 | 1.23 | 1.23 | 919 | 1675 | 1611 |
| G82077 | 1.381 | 1.22 | 1.24 | 920 | 1554 | 1728 |
| J82084 | 1.380 | 1.06 | 1.10 | 921 | 605 | 736 |
| G82109 | 1.380 | 1.09 | 1.12 | 922 | 747 | 847 |
| A83041 | 1.380 | 1.29 | 1.29 | 923 | 2278 | 2281 |
| C84680 | 1.380 | 1.13 | 1.15 | 924 | 959 | 1093 |
| N83006 | 1.380 | 1.23 | 1.24 | 925 | 1674 | 1737 |
| J82120 | 1.379 | 1.06 | 1.05 | 926 | 608 | 587 |
| P81713 | 1.379 | 1.10 | 1.14 | 927 | 776 | 999 |
| Y02624 | 1.379 | 1.02 | 1.03 | 928 | 463 | 513 |
| A89616 | 1.379 | 1.13 | 1.15 | 929 | 968 | 1052 |
| M88628 | 1.378 | 1.22 | 1.24 | 930 | 1595 | 1751 |
| J82040 | 1.378 | 1.13 | 1.13 | 931 | 923 | 922 |
| F82624 | 1.378 | 1.05 | 1.05 | 932 | 567 | 571 |
| L82003 | 1.378 | 1.10 | 1.11 | 933 | 782 | 797 |
| M85803 | 1.378 | 1.01 | 1.00 | 934 | 449 | 432 |
| F81106 | 1.378 | 1.12 | 1.14 | 935 | 870 | 986 |
| G82232 | 1.378 | 1.02 | 1.06 | 936 | 501 | 610 |
| D81007 | 1.377 | 1.09 | 1.10 | 937 | 740 | 768 |
| H85067 | 1.377 | 1.09 | 1.05 | 938 | 741 | 592 |
| P91019 | 1.377 | 1.43 | 1.41 | 939 | 4206 | 3913 |
| A81043 | 1.377 | 1.24 | 1.25 | 940 | 1786 | 1881 |
| M83703 | 1.377 | 1.11 | 1.14 | 941 | 816 | 1034 |
| G82066 | 1.377 | 1.22 | 1.22 | 942 | 1550 | 1635 |
| N83612 | 1.376 | 1.25 | 1.25 | 943 | 1919 | 1923 |
| B85655 | 1.376 | 1.33 | 1.32 | 944 | 2826 | 2626 |

| | | | | | | |
|---|---|---|---|---|---|---|
| M89602 | 1.376 | 1.36 | 1.30 | 945 | 3199 | 2388 |
| P92605 | 1.376 | 1.07 | 1.10 | 946 | 646 | 787 |
| C83643 | 1.376 | 1.41 | 1.42 | 947 | 3909 | 4049 |
| L81642 | 1.376 | 1.04 | 1.04 | 948 | 550 | 523 |
| K81030 | 1.375 | 1.07 | 1.06 | 949 | 647 | 625 |
| N84017 | 1.375 | 1.24 | 1.24 | 950 | 1835 | 1774 |
| M86617 | 1.375 | 1.22 | 1.21 | 951 | 1634 | 1558 |
| N82650 | 1.374 | 1.23 | 1.25 | 952 | 1726 | 1877 |
| F81211 | 1.374 | 1.02 | 1.02 | 953 | 489 | 492 |
| P87668 | 1.374 | 1.13 | 1.15 | 954 | 942 | 1100 |
| P85008 | 1.374 | 1.21 | 1.23 | 955 | 1527 | 1697 |
| P92647 | 1.374 | 1.22 | 1.22 | 956 | 1598 | 1664 |
| G82184 | 1.374 | 1.12 | 1.15 | 957 | 910 | 1071 |
| M83601 | 1.374 | 1.20 | 1.23 | 958 | 1416 | 1753 |
| M85023 | 1.374 | 1.20 | 1.18 | 959 | 1424 | 1300 |
| L83068 | 1.374 | 1.12 | 1.11 | 960 | 929 | 836 |
| N83014 | 1.373 | 1.21 | 1.22 | 961 | 1557 | 1631 |
| K82019 | 1.373 | 1.07 | 1.04 | 962 | 650 | 549 |
| P87014 | 1.373 | 1.14 | 1.17 | 963 | 1070 | 1257 |
| F84047 | 1.372 | 1.01 | 0.99 | 964 | 467 | 418 |
| A88010 | 1.372 | 1.37 | 1.35 | 965 | 3313 | 3132 |
| N83621 | 1.372 | 1.28 | 1.28 | 966 | 2244 | 2295 |
| A83074 | 1.372 | 1.21 | 1.21 | 967 | 1526 | 1539 |
| M88600 | 1.372 | 1.17 | 1.18 | 968 | 1220 | 1326 |
| N83005 | 1.372 | 1.25 | 1.26 | 969 | 1899 | 1976 |
| M85053 | 1.372 | 1.19 | 1.18 | 970 | 1389 | 1309 |
| P84690 | 1.372 | 1.02 | 1.03 | 971 | 502 | 542 |
| N83028 | 1.371 | 1.21 | 1.22 | 972 | 1590 | 1646 |
| A89010 | 1.370 | 1.21 | 1.22 | 973 | 1555 | 1636 |
| P81091 | 1.370 | 1.32 | 1.32 | 974 | 2778 | 2789 |
| N83023 | 1.370 | 1.38 | 1.36 | 975 | 3488 | 3336 |
| P81147 | 1.370 | 1.25 | 1.29 | 976 | 1974 | 2400 |
| N83024 | 1.369 | 1.26 | 1.27 | 977 | 2057 | 2151 |
| B84612 | 1.369 | 1.12 | 1.13 | 978 | 904 | 995 |
| N84027 | 1.369 | 1.04 | 1.06 | 979 | 583 | 658 |
| F81181 | 1.369 | 1.00 | 1.02 | 980 | 445 | 486 |
| Y02567 | 1.369 | 1.09 | 1.07 | 981 | 754 | 693 |
| H85063 | 1.369 | 1.08 | 1.06 | 982 | 725 | 648 |
| A81017 | 1.369 | 1.04 | 1.04 | 983 | 562 | 591 |
| L83121 | 1.369 | 1.12 | 1.13 | 984 | 969 | 979 |
| L83651 | 1.369 | 1.21 | 1.20 | 985 | 1542 | 1512 |
| E86620 | 1.368 | 0.95 | 0.95 | 986 | 343 | 330 |
| L83028 | 1.368 | 1.21 | 1.22 | 987 | 1540 | 1667 |
| M84022 | 1.368 | 1.11 | 1.11 | 988 | 908 | 878 |
| Y00437 | 1.368 | 0.96 | 0.97 | 989 | 375 | 385 |
| H85023 | 1.368 | 1.16 | 1.14 | 990 | 1185 | 1074 |
| M88001 | 1.367 | 1.17 | 1.18 | 991 | 1313 | 1354 |
| B83034 | 1.367 | 1.33 | 1.36 | 992 | 2953 | 3310 |
| G82053 | 1.367 | 1.23 | 1.22 | 993 | 1770 | 1677 |
| P86017 | 1.367 | 1.39 | 1.41 | 994 | 3730 | 4075 |

| | | | | | | |
|---|---|---|---|---|---|---|
| B84006 | 1.366 | 1.24 | 1.25 | 995 | 1930 | 1986 |
| G82083 | 1.366 | 1.07 | 1.05 | 996 | 694 | 640 |
| M83016 | 1.366 | 1.17 | 1.12 | 997 | 1252 | 969 |
| E86007 | 1.366 | 1.08 | 1.07 | 998 | 739 | 685 |
| B87009 | 1.366 | 1.39 | 1.39 | 999 | 3815 | 3815 |
| F81221 | 1.365 | 1.11 | 1.12 | 1000 | 872 | 955 |
| G81004 | 1.365 | 1.29 | 1.26 | 1001 | 2445 | 2052 |
| M88007 | 1.365 | 1.26 | 1.27 | 1002 | 2128 | 2171 |
| G82708 | 1.364 | 0.95 | 0.98 | 1003 | 351 | 400 |
| C84012 | 1.364 | 1.24 | 1.26 | 1004 | 1909 | 2059 |
| D81611 | 1.363 | 1.22 | 1.26 | 1005 | 1705 | 2172 |
| A87017 | 1.363 | 1.28 | 1.28 | 1006 | 2367 | 2410 |
| P81633 | 1.363 | 1.17 | 1.19 | 1007 | 1318 | 1458 |
| P89004 | 1.363 | 1.08 | 1.11 | 1008 | 765 | 884 |
| J82630 | 1.363 | 0.96 | 0.95 | 1009 | 369 | 354 |
| M88646 | 1.362 | 1.06 | 1.09 | 1010 | 690 | 783 |
| B83063 | 1.361 | 1.19 | 1.20 | 1011 | 1478 | 1508 |
| F81026 | 1.361 | 1.28 | 1.29 | 1012 | 2346 | 2469 |
| G82148 | 1.361 | 1.10 | 1.12 | 1013 | 893 | 981 |
| E85096 | 1.361 | 0.99 | 1.00 | 1014 | 448 | 454 |
| P81755 | 1.361 | 1.26 | 1.31 | 1015 | 2187 | 2755 |
| A81031 | 1.360 | 1.20 | 1.24 | 1016 | 1602 | 1915 |
| M83715 | 1.360 | 1.12 | 1.07 | 1017 | 1009 | 727 |
| C88014 | 1.360 | 1.32 | 1.33 | 1018 | 2919 | 2954 |
| C81087 | 1.360 | 1.12 | 1.12 | 1019 | 1024 | 980 |
| A89018 | 1.360 | 1.38 | 1.37 | 1020 | 3639 | 3602 |
| D81019 | 1.360 | 1.19 | 1.18 | 1021 | 1436 | 1446 |
| A81040 | 1.360 | 1.17 | 1.17 | 1022 | 1342 | 1359 |
| E82655 | 1.359 | 1.09 | 1.07 | 1023 | 845 | 729 |
| N84617 | 1.359 | 1.02 | 1.02 | 1024 | 530 | 535 |
| A86601 | 1.359 | 1.21 | 1.21 | 1025 | 1647 | 1696 |
| M81046 | 1.359 | 1.13 | 1.12 | 1026 | 1071 | 983 |
| E81632 | 1.359 | 1.11 | 1.11 | 1027 | 912 | 957 |
| H85092 | 1.358 | 0.95 | 0.94 | 1028 | 366 | 345 |
| G81022 | 1.358 | 1.12 | 1.14 | 1029 | 1032 | 1129 |
| E81040 | 1.358 | 1.07 | 1.07 | 1030 | 714 | 738 |
| M83709 | 1.358 | 1.01 | 1.05 | 1031 | 517 | 660 |
| P82609 | 1.357 | 1.20 | 1.22 | 1032 | 1616 | 1762 |
| C83013 | 1.357 | 1.23 | 1.22 | 1033 | 1883 | 1785 |
| L83032 | 1.357 | 1.04 | 1.05 | 1034 | 636 | 657 |
| F81652 | 1.357 | 1.09 | 1.09 | 1035 | 819 | 819 |
| A83001 | 1.357 | 1.16 | 1.17 | 1036 | 1244 | 1315 |
| D83023 | 1.357 | 1.12 | 1.12 | 1037 | 1042 | 1011 |
| A81622 | 1.357 | 1.29 | 1.32 | 1038 | 2580 | 2875 |
| G83063 | 1.357 | 1.07 | 1.07 | 1039 | 751 | 719 |
| A83004 | 1.357 | 1.20 | 1.21 | 1040 | 1635 | 1675 |
| D83034 | 1.357 | 1.22 | 1.22 | 1041 | 1797 | 1755 |
| M82017 | 1.356 | 1.12 | 1.12 | 1042 | 1002 | 1010 |
| P86008 | 1.356 | 1.27 | 1.28 | 1043 | 2266 | 2435 |
| A82052 | 1.356 | 1.10 | 1.12 | 1044 | 869 | 1048 |

| | | | | | | |
|---|---|---|---|---|---|---|
| H84054 | 1.356 | 1.06 | 1.04 | 1045 | 704 | 626 |
| M85071 | 1.356 | 1.11 | 1.10 | 1046 | 981 | 908 |
| C88086 | 1.356 | 1.28 | 1.29 | 1047 | 2482 | 2549 |
| G82044 | 1.356 | 1.08 | 1.08 | 1048 | 807 | 779 |
| C83056 | 1.356 | 1.20 | 1.22 | 1049 | 1597 | 1835 |
| M83069 | 1.356 | 1.12 | 1.11 | 1050 | 1015 | 941 |
| B81120 | 1.356 | 1.17 | 1.18 | 1051 | 1394 | 1405 |
| A81032 | 1.355 | 1.14 | 1.14 | 1052 | 1133 | 1161 |
| K83037 | 1.355 | 1.08 | 1.08 | 1053 | 808 | 764 |
| N82053 | 1.355 | 1.17 | 1.19 | 1054 | 1382 | 1490 |
| Y04266 | 1.355 | 1.14 | 1.14 | 1055 | 1128 | 1152 |
| C88026 | 1.354 | 1.08 | 1.10 | 1056 | 788 | 890 |
| C84705 | 1.354 | 1.15 | 1.14 | 1057 | 1221 | 1143 |
| P91006 | 1.354 | 1.21 | 1.21 | 1058 | 1719 | 1745 |
| A86023 | 1.354 | 1.14 | 1.15 | 1059 | 1157 | 1247 |
| F81641 | 1.354 | 1.11 | 1.12 | 1060 | 996 | 1024 |
| M83719 | 1.354 | 1.08 | 1.12 | 1061 | 789 | 1004 |
| B86060 | 1.354 | 1.19 | 1.20 | 1062 | 1508 | 1615 |
| D83056 | 1.353 | 1.14 | 1.15 | 1063 | 1186 | 1212 |
| A83049 | 1.353 | 1.17 | 1.17 | 1064 | 1364 | 1407 |
| B81689 | 1.353 | 1.09 | 1.12 | 1065 | 865 | 1049 |
| M83143 | 1.353 | 1.25 | 1.29 | 1066 | 2134 | 2545 |
| P91012 | 1.353 | 1.17 | 1.17 | 1067 | 1355 | 1386 |
| A82016 | 1.353 | 1.15 | 1.18 | 1068 | 1246 | 1454 |
| B82001 | 1.352 | 1.25 | 1.27 | 1069 | 2122 | 2313 |
| C86006 | 1.352 | 1.18 | 1.20 | 1070 | 1430 | 1670 |
| M85739 | 1.352 | 1.05 | 1.04 | 1071 | 657 | 644 |
| P82007 | 1.352 | 1.22 | 1.23 | 1072 | 1884 | 1924 |
| F81056 | 1.351 | 1.22 | 1.24 | 1073 | 1843 | 2002 |
| L81670 | 1.351 | 1.23 | 1.19 | 1074 | 1935 | 1546 |
| F85060 | 1.351 | 0.93 | 0.94 | 1075 | 334 | 355 |
| D81042 | 1.351 | 1.05 | 1.03 | 1076 | 685 | 616 |
| M81077 | 1.351 | 1.07 | 1.09 | 1077 | 750 | 875 |
| H81111 | 1.351 | 0.93 | 0.91 | 1078 | 349 | 301 |
| M85159 | 1.351 | 1.15 | 1.14 | 1079 | 1274 | 1160 |
| P82011 | 1.350 | 1.19 | 1.20 | 1080 | 1524 | 1654 |
| E85663 | 1.350 | 0.97 | 0.98 | 1081 | 430 | 439 |
| D81629 | 1.350 | 1.09 | 1.09 | 1082 | 856 | 869 |
| C88096 | 1.350 | 1.24 | 1.25 | 1083 | 2054 | 2122 |
| P82013 | 1.350 | 1.32 | 1.32 | 1084 | 2966 | 3011 |
| Y03661 | 1.350 | 1.09 | 1.07 | 1085 | 850 | 737 |
| D81027 | 1.350 | 1.10 | 1.10 | 1086 | 949 | 903 |
| A81054 | 1.349 | 1.20 | 1.21 | 1087 | 1709 | 1734 |
| F81029 | 1.349 | 1.06 | 1.07 | 1088 | 728 | 740 |
| P92621 | 1.349 | 1.24 | 1.24 | 1089 | 2055 | 2062 |
| P81123 | 1.349 | 1.21 | 1.25 | 1090 | 1807 | 2118 |
| P81180 | 1.349 | 1.26 | 1.22 | 1091 | 2289 | 1822 |
| P81674 | 1.349 | 1.11 | 1.12 | 1092 | 977 | 1087 |
| M85107 | 1.348 | 1.01 | 1.01 | 1093 | 547 | 516 |
| C86016 | 1.348 | 1.29 | 1.31 | 1094 | 2693 | 2908 |

| | | | | | | |
|---|---|---|---|---|---|---|
| J82622 | 1.348 | 1.15 | 1.15 | 1095 | 1235 | 1291 |
| A81042 | 1.348 | 1.30 | 1.29 | 1096 | 2772 | 2625 |
| J83040 | 1.348 | 1.10 | 1.09 | 1097 | 914 | 839 |
| C82624 | 1.348 | 1.03 | 1.03 | 1098 | 601 | 604 |
| J83055 | 1.348 | 1.13 | 1.12 | 1099 | 1144 | 1047 |
| B82024 | 1.348 | 1.34 | 1.35 | 1100 | 3339 | 3481 |
| G82777 | 1.347 | 0.95 | 0.96 | 1101 | 383 | 392 |
| C82017 | 1.347 | 1.09 | 1.08 | 1102 | 888 | 831 |
| J83023 | 1.347 | 1.15 | 1.13 | 1103 | 1263 | 1155 |
| L84013 | 1.346 | 1.09 | 1.09 | 1104 | 896 | 846 |
| G85736 | 1.346 | 1.12 | 1.12 | 1105 | 1060 | 1082 |
| E81617 | 1.346 | 1.08 | 1.09 | 1106 | 857 | 881 |
| L83013 | 1.346 | 1.15 | 1.15 | 1107 | 1257 | 1296 |
| M85155 | 1.345 | 1.23 | 1.21 | 1108 | 2001 | 1823 |
| J82150 | 1.345 | 1.11 | 1.10 | 1109 | 1041 | 971 |
| N85021 | 1.345 | 1.21 | 1.19 | 1110 | 1758 | 1638 |
| F85003 | 1.345 | 1.04 | 1.03 | 1111 | 648 | 631 |
| M82026 | 1.344 | 1.09 | 1.09 | 1112 | 900 | 907 |
| H85665 | 1.344 | 1.10 | 1.09 | 1113 | 948 | 874 |
| B81008 | 1.344 | 1.17 | 1.21 | 1114 | 1429 | 1831 |
| N82070 | 1.344 | 1.28 | 1.29 | 1115 | 2577 | 2646 |
| F82018 | 1.343 | 1.05 | 1.06 | 1116 | 709 | 757 |
| B85015 | 1.343 | 1.23 | 1.23 | 1117 | 2038 | 1997 |
| G81014 | 1.343 | 0.99 | 1.01 | 1118 | 506 | 575 |
| A82646 | 1.343 | 1.50 | 1.50 | 1119 | 5445 | 5466 |
| M81058 | 1.342 | 1.16 | 1.14 | 1120 | 1393 | 1255 |
| N82668 | 1.342 | 1.10 | 1.12 | 1121 | 1007 | 1128 |
| M83074 | 1.342 | 1.05 | 1.02 | 1122 | 731 | 601 |
| P87620 | 1.342 | 1.16 | 1.18 | 1123 | 1412 | 1593 |
| K81066 | 1.342 | 0.91 | 0.90 | 1124 | 314 | 293 |
| E82032 | 1.342 | 1.04 | 1.03 | 1125 | 661 | 628 |
| B85027 | 1.342 | 1.01 | 1.02 | 1126 | 572 | 614 |
| C84679 | 1.342 | 1.28 | 1.30 | 1127 | 2575 | 2849 |
| C83045 | 1.341 | 1.37 | 1.39 | 1128 | 3884 | 4103 |
| F81053 | 1.341 | 1.03 | 0.99 | 1129 | 631 | 506 |
| G81059 | 1.341 | 1.24 | 1.21 | 1130 | 2170 | 1841 |
| A81629 | 1.341 | 1.26 | 1.25 | 1131 | 2333 | 2292 |
| M91034 | 1.341 | 1.37 | 1.38 | 1132 | 3900 | 3960 |
| M85024 | 1.341 | 1.18 | 1.17 | 1133 | 1571 | 1488 |
| A86026 | 1.341 | 1.20 | 1.21 | 1134 | 1740 | 1854 |
| B81099 | 1.341 | 1.16 | 1.18 | 1135 | 1372 | 1584 |
| A82064 | 1.341 | 1.52 | 1.53 | 1136 | 5694 | 5792 |
| N83019 | 1.341 | 1.18 | 1.20 | 1137 | 1568 | 1724 |
| N81113 | 1.340 | 1.15 | 1.12 | 1138 | 1348 | 1133 |
| M84045 | 1.340 | 1.15 | 1.15 | 1139 | 1315 | 1318 |
| P82616 | 1.340 | 1.09 | 1.10 | 1140 | 915 | 996 |
| M91003 | 1.340 | 1.12 | 1.13 | 1141 | 1120 | 1194 |
| A86041 | 1.340 | 1.12 | 1.13 | 1142 | 1104 | 1181 |
| M91623 | 1.340 | 1.24 | 1.26 | 1143 | 2211 | 2431 |
| M86011 | 1.339 | 1.27 | 1.25 | 1144 | 2535 | 2240 |

| | | | | | | |
|---|---|---|---|---|---|---|
| K82067 | 1.339 | 0.95 | 0.95 | 1145 | 404 | 395 |
| C88023 | 1.339 | 1.18 | 1.20 | 1146 | 1582 | 1720 |
| C85016 | 1.339 | 1.27 | 1.27 | 1147 | 2495 | 2518 |
| G82055 | 1.338 | 1.02 | 1.02 | 1148 | 627 | 632 |
| B85037 | 1.338 | 1.10 | 1.10 | 1149 | 1047 | 1035 |
| C83049 | 1.338 | 1.38 | 1.36 | 1150 | 4025 | 3723 |
| A83026 | 1.338 | 1.16 | 1.17 | 1151 | 1418 | 1468 |
| B86104 | 1.337 | 1.14 | 1.16 | 1152 | 1262 | 1399 |
| B81025 | 1.337 | 1.49 | 1.46 | 1153 | 5392 | 5023 |
| N84012 | 1.337 | 1.13 | 1.13 | 1154 | 1181 | 1185 |
| M84006 | 1.337 | 1.09 | 1.05 | 1155 | 938 | 714 |
| C82018 | 1.337 | 1.20 | 1.20 | 1156 | 1819 | 1808 |
| B83614 | 1.336 | 1.27 | 1.29 | 1157 | 2563 | 2834 |
| E81048 | 1.336 | 1.07 | 1.08 | 1158 | 848 | 852 |
| C84021 | 1.336 | 1.26 | 1.24 | 1159 | 2373 | 2221 |
| L83074 | 1.336 | 1.19 | 1.20 | 1160 | 1698 | 1791 |
| M86627 | 1.336 | 1.02 | 1.02 | 1161 | 639 | 613 |
| K81015 | 1.336 | 1.01 | 1.00 | 1162 | 594 | 539 |
| C83036 | 1.336 | 1.24 | 1.24 | 1163 | 2233 | 2185 |
| P82016 | 1.336 | 1.12 | 1.14 | 1164 | 1153 | 1265 |
| A87012 | 1.335 | 1.30 | 1.31 | 1165 | 2949 | 3134 |
| M83028 | 1.335 | 1.17 | 1.20 | 1166 | 1511 | 1815 |
| F82038 | 1.335 | 1.08 | 1.09 | 1167 | 927 | 950 |
| G82180 | 1.335 | 1.07 | 1.09 | 1168 | 835 | 988 |
| A88004 | 1.335 | 1.23 | 1.22 | 1169 | 2086 | 1999 |
| C87017 | 1.335 | 1.14 | 1.15 | 1170 | 1319 | 1379 |
| P81127 | 1.335 | 1.20 | 1.18 | 1171 | 1849 | 1566 |
| C88036 | 1.334 | 1.19 | 1.20 | 1172 | 1712 | 1857 |
| N84614 | 1.334 | 1.03 | 1.03 | 1173 | 681 | 672 |
| M85027 | 1.334 | 1.16 | 1.14 | 1174 | 1463 | 1338 |
| C84009 | 1.334 | 1.08 | 1.08 | 1175 | 924 | 892 |
| G82147 | 1.334 | 1.28 | 1.31 | 1176 | 2667 | 3063 |
| P81130 | 1.334 | 1.15 | 1.17 | 1177 | 1402 | 1519 |
| F85025 | 1.334 | 0.98 | 0.98 | 1178 | 511 | 485 |
| F81018 | 1.333 | 1.39 | 1.39 | 1179 | 4254 | 4214 |
| N82086 | 1.333 | 1.32 | 1.33 | 1180 | 3276 | 3436 |
| P82036 | 1.333 | 1.25 | 1.25 | 1181 | 2320 | 2308 |
| Y02794 | 1.333 | 0.99 | 0.98 | 1182 | 518 | 487 |
| C83027 | 1.332 | 1.25 | 1.28 | 1183 | 2379 | 2692 |
| F81016 | 1.332 | 1.06 | 1.08 | 1184 | 830 | 921 |
| N82676 | 1.332 | 0.94 | 0.96 | 1185 | 403 | 443 |
| M83045 | 1.332 | 1.05 | 1.02 | 1186 | 778 | 630 |
| P81169 | 1.332 | 1.14 | 1.13 | 1187 | 1299 | 1206 |
| C83633 | 1.332 | 1.07 | 1.06 | 1188 | 839 | 817 |
| F81067 | 1.332 | 1.06 | 1.07 | 1189 | 814 | 825 |
| C85033 | 1.332 | 1.10 | 1.12 | 1190 | 1061 | 1177 |
| N81049 | 1.332 | 1.21 | 1.19 | 1191 | 1922 | 1709 |
| A88007 | 1.332 | 1.19 | 1.18 | 1192 | 1718 | 1655 |
| P87634 | 1.331 | 1.10 | 1.12 | 1193 | 1076 | 1202 |
| C84660 | 1.331 | 1.00 | 1.00 | 1194 | 557 | 566 |

| | | | | | | |
|---|---|---|---|---|---|---|
| P84051 | 1.331 | 1.10 | 1.12 | 1195 | 1075 | 1171 |
| B81619 | 1.331 | 1.09 | 1.08 | 1196 | 965 | 956 |
| D83012 | 1.331 | 1.11 | 1.10 | 1197 | 1102 | 1064 |
| M83073 | 1.330 | 1.11 | 1.07 | 1198 | 1125 | 845 |
| P84021 | 1.330 | 1.12 | 1.14 | 1199 | 1175 | 1348 |
| D83055 | 1.329 | 1.21 | 1.21 | 1200 | 1968 | 1953 |
| N81048 | 1.329 | 1.15 | 1.13 | 1201 | 1387 | 1298 |
| J82125 | 1.329 | 1.02 | 1.02 | 1202 | 664 | 654 |
| Y01221 | 1.329 | 1.21 | 1.21 | 1203 | 1936 | 1995 |
| E84004 | 1.329 | 1.01 | 0.99 | 1204 | 629 | 554 |
| B87027 | 1.329 | 1.09 | 1.12 | 1205 | 1037 | 1190 |
| L84027 | 1.328 | 1.01 | 1.01 | 1206 | 611 | 620 |
| L82042 | 1.328 | 1.24 | 1.24 | 1207 | 2317 | 2299 |
| K81083 | 1.328 | 1.04 | 1.06 | 1208 | 746 | 803 |
| C82029 | 1.328 | 1.29 | 1.28 | 1209 | 2929 | 2773 |
| N84002 | 1.328 | 1.30 | 1.29 | 1210 | 2994 | 2836 |
| A88012 | 1.327 | 1.35 | 1.33 | 1211 | 3709 | 3450 |
| B86081 | 1.327 | 1.06 | 1.08 | 1212 | 851 | 913 |
| H81058 | 1.327 | 0.98 | 0.96 | 1213 | 524 | 456 |
| K82615 | 1.327 | 0.93 | 0.93 | 1214 | 395 | 388 |
| P85014 | 1.327 | 1.10 | 1.12 | 1215 | 1098 | 1237 |
| A89032 | 1.327 | 1.23 | 1.24 | 1216 | 2208 | 2316 |
| D81020 | 1.327 | 1.08 | 1.09 | 1217 | 980 | 1015 |
| D82104 | 1.326 | 1.10 | 1.09 | 1218 | 1078 | 1020 |
| P86014 | 1.326 | 1.23 | 1.25 | 1219 | 2207 | 2463 |
| K84062 | 1.326 | 1.01 | 1.01 | 1220 | 625 | 624 |
| F84004 | 1.326 | 1.10 | 1.08 | 1221 | 1087 | 965 |
| E83668 | 1.326 | 0.90 | 0.88 | 1222 | 316 | 289 |
| P86009 | 1.326 | 1.21 | 1.23 | 1223 | 1979 | 2187 |
| Y01165 | 1.325 | 1.06 | 1.04 | 1224 | 828 | 720 |
| G83064 | 1.325 | 1.02 | 1.01 | 1225 | 653 | 641 |
| D82080 | 1.325 | 1.17 | 1.16 | 1226 | 1621 | 1501 |
| P81031 | 1.325 | 1.12 | 1.09 | 1227 | 1199 | 1028 |
| P85015 | 1.325 | 1.18 | 1.19 | 1228 | 1664 | 1813 |
| F81048 | 1.325 | 1.01 | 1.00 | 1229 | 644 | 582 |
| P91020 | 1.325 | 1.13 | 1.13 | 1230 | 1285 | 1283 |
| A82631 | 1.324 | 1.15 | 1.13 | 1231 | 1480 | 1331 |
| G82028 | 1.324 | 1.13 | 1.10 | 1232 | 1269 | 1092 |
| P81684 | 1.324 | 1.32 | 1.37 | 1233 | 3403 | 4074 |
| F81073 | 1.324 | 1.11 | 1.12 | 1234 | 1164 | 1214 |
| A81022 | 1.324 | 1.21 | 1.22 | 1235 | 2053 | 2077 |
| G82076 | 1.324 | 1.09 | 1.09 | 1236 | 1069 | 1063 |
| F86652 | 1.323 | 1.03 | 1.02 | 1237 | 721 | 656 |
| B82031 | 1.323 | 1.09 | 1.07 | 1238 | 1033 | 906 |
| A83014 | 1.323 | 1.24 | 1.25 | 1239 | 2323 | 2445 |
| K81067 | 1.323 | 1.05 | 1.05 | 1240 | 794 | 815 |
| L82014 | 1.323 | 1.22 | 1.22 | 1241 | 2145 | 2081 |
| Y00774 | 1.322 | 1.27 | 1.28 | 1242 | 2770 | 2778 |
| H85053 | 1.321 | 0.96 | 0.96 | 1243 | 481 | 448 |
| A88002 | 1.321 | 1.31 | 1.30 | 1244 | 3234 | 3139 |

| | | | | | | |
|---|---|---|---|---|---|---|
| D81060 | 1.321 | 1.13 | 1.12 | 1245 | 1289 | 1227 |
| N84041 | 1.321 | 1.07 | 1.06 | 1246 | 902 | 886 |
| M92627 | 1.321 | 1.24 | 1.25 | 1247 | 2413 | 2458 |
| E82040 | 1.320 | 1.06 | 1.06 | 1248 | 859 | 862 |
| B87028 | 1.320 | 1.25 | 1.27 | 1249 | 2479 | 2708 |
| Y00078 | 1.320 | 1.01 | 0.97 | 1250 | 628 | 488 |
| P81750 | 1.320 | 1.06 | 1.05 | 1251 | 855 | 808 |
| L83006 | 1.320 | 1.23 | 1.24 | 1252 | 2281 | 2355 |
| M83065 | 1.320 | 1.13 | 1.09 | 1253 | 1300 | 1053 |
| B86070 | 1.320 | 1.28 | 1.29 | 1254 | 2842 | 3009 |
| F81618 | 1.320 | 1.29 | 1.28 | 1255 | 3020 | 2940 |
| P82652 | 1.319 | 1.32 | 1.32 | 1256 | 3357 | 3415 |
| B83033 | 1.319 | 1.21 | 1.22 | 1257 | 2030 | 2164 |
| G82650 | 1.319 | 1.12 | 1.14 | 1258 | 1254 | 1418 |
| N83030 | 1.319 | 1.09 | 1.11 | 1259 | 1056 | 1167 |
| N83035 | 1.319 | 1.39 | 1.39 | 1260 | 4350 | 4447 |
| C83628 | 1.318 | 1.08 | 1.07 | 1261 | 991 | 914 |
| A82022 | 1.318 | 1.11 | 1.14 | 1262 | 1218 | 1412 |
| L84045 | 1.318 | 1.13 | 1.12 | 1263 | 1366 | 1278 |
| Y02129 | 1.318 | 1.16 | 1.16 | 1264 | 1592 | 1580 |
| B86014 | 1.318 | 1.10 | 1.11 | 1265 | 1140 | 1228 |
| A82024 | 1.318 | 1.24 | 1.27 | 1266 | 2457 | 2777 |
| B86020 | 1.317 | 1.12 | 1.14 | 1267 | 1293 | 1450 |
| N81114 | 1.317 | 1.12 | 1.11 | 1268 | 1260 | 1198 |
| A83022 | 1.317 | 1.21 | 1.22 | 1269 | 2063 | 2141 |
| N81111 | 1.317 | 1.12 | 1.10 | 1270 | 1298 | 1168 |
| G82712 | 1.317 | 0.98 | 0.98 | 1271 | 559 | 557 |
| A82037 | 1.316 | 1.33 | 1.34 | 1272 | 3689 | 3705 |
| N81045 | 1.316 | 1.13 | 1.14 | 1273 | 1330 | 1451 |
| D81040 | 1.316 | 1.22 | 1.20 | 1274 | 2200 | 1988 |
| G82026 | 1.316 | 1.07 | 1.08 | 1275 | 962 | 1038 |
| L82023 | 1.316 | 1.16 | 1.16 | 1276 | 1587 | 1562 |
| A86030 | 1.316 | 1.23 | 1.24 | 1277 | 2309 | 2425 |
| F81130 | 1.315 | 1.07 | 1.06 | 1278 | 984 | 864 |
| A82044 | 1.315 | 1.27 | 1.30 | 1279 | 2815 | 3163 |
| J83019 | 1.315 | 1.11 | 1.10 | 1280 | 1200 | 1123 |
| B82042 | 1.314 | 1.16 | 1.16 | 1281 | 1653 | 1597 |
| P83019 | 1.314 | 1.17 | 1.17 | 1282 | 1697 | 1721 |
| J82117 | 1.314 | 1.20 | 1.20 | 1283 | 1993 | 2037 |
| G82158 | 1.314 | 1.01 | 1.02 | 1284 | 673 | 690 |
| C86039 | 1.314 | 1.18 | 1.20 | 1285 | 1808 | 1989 |
| A82012 | 1.314 | 1.25 | 1.27 | 1286 | 2530 | 2786 |
| M81037 | 1.314 | 1.13 | 1.13 | 1287 | 1356 | 1361 |
| C88045 | 1.313 | 1.15 | 1.16 | 1288 | 1496 | 1602 |
| P81044 | 1.313 | 1.26 | 1.22 | 1289 | 2704 | 2268 |
| P88020 | 1.313 | 1.12 | 1.12 | 1290 | 1301 | 1312 |
| B83611 | 1.313 | 1.25 | 1.26 | 1291 | 2588 | 2759 |
| D83010 | 1.313 | 1.36 | 1.35 | 1292 | 4081 | 3985 |
| B82092 | 1.313 | 1.21 | 1.23 | 1293 | 2147 | 2310 |
| J83044 | 1.313 | 1.04 | 1.03 | 1294 | 796 | 746 |

| | | | | | | |
|---|---|---|---|---|---|---|
| A83015 | 1.312 | 1.22 | 1.22 | 1295 | 2213 | 2291 |
| C83007 | 1.312 | 1.05 | 1.05 | 1296 | 879 | 823 |
| D82003 | 1.312 | 1.14 | 1.15 | 1297 | 1431 | 1591 |
| J81633 | 1.312 | 1.13 | 1.12 | 1298 | 1380 | 1322 |
| E84005 | 1.312 | 1.07 | 1.04 | 1299 | 976 | 788 |
| P92004 | 1.311 | 1.18 | 1.19 | 1300 | 1818 | 1896 |
| E85744 | 1.311 | 0.91 | 0.91 | 1301 | 392 | 371 |
| E84040 | 1.311 | 0.99 | 0.97 | 1302 | 599 | 521 |
| J82210 | 1.311 | 1.22 | 1.20 | 1303 | 2303 | 2054 |
| B87019 | 1.311 | 1.18 | 1.20 | 1304 | 1812 | 1990 |
| D81074 | 1.311 | 1.00 | 1.00 | 1305 | 621 | 623 |
| F81691 | 1.311 | 1.01 | 1.02 | 1306 | 665 | 698 |
| P81681 | 1.311 | 1.29 | 1.33 | 1307 | 3129 | 3684 |
| D81619 | 1.310 | 1.04 | 1.04 | 1308 | 824 | 807 |
| B86009 | 1.310 | 1.18 | 1.19 | 1309 | 1823 | 1960 |
| P81133 | 1.310 | 1.23 | 1.22 | 1310 | 2326 | 2329 |
| P82613 | 1.310 | 1.22 | 1.23 | 1311 | 2295 | 2404 |
| P84012 | 1.310 | 1.19 | 1.21 | 1312 | 1990 | 2191 |
| C83083 | 1.310 | 1.19 | 1.22 | 1313 | 1976 | 2250 |
| F81644 | 1.310 | 1.07 | 1.08 | 1314 | 1011 | 1042 |
| M83050 | 1.310 | 1.05 | 1.03 | 1315 | 849 | 784 |
| B87025 | 1.310 | 1.35 | 1.35 | 1316 | 3933 | 4055 |
| M83037 | 1.310 | 1.11 | 1.06 | 1317 | 1229 | 958 |
| C82068 | 1.310 | 1.17 | 1.16 | 1318 | 1768 | 1652 |
| G83052 | 1.309 | 1.07 | 1.06 | 1319 | 972 | 905 |
| C84074 | 1.309 | 1.27 | 1.29 | 1320 | 2923 | 3100 |
| P83603 | 1.309 | 1.34 | 1.33 | 1321 | 3899 | 3776 |
| M83033 | 1.309 | 1.20 | 1.24 | 1322 | 2119 | 2516 |
| F81700 | 1.309 | 1.13 | 1.13 | 1323 | 1420 | 1384 |
| C82047 | 1.308 | 1.03 | 1.03 | 1324 | 767 | 786 |
| B81616 | 1.308 | 1.22 | 1.26 | 1325 | 2310 | 2811 |
| C86611 | 1.308 | 1.16 | 1.19 | 1326 | 1694 | 1925 |
| C87002 | 1.308 | 1.20 | 1.21 | 1327 | 2072 | 2144 |
| L84055 | 1.308 | 1.06 | 1.05 | 1328 | 945 | 853 |
| E85728 | 1.308 | 1.14 | 1.14 | 1329 | 1456 | 1459 |
| C82053 | 1.308 | 1.23 | 1.22 | 1330 | 2431 | 2323 |
| D82062 | 1.308 | 1.05 | 1.04 | 1331 | 916 | 835 |
| P84043 | 1.308 | 1.16 | 1.17 | 1332 | 1646 | 1797 |
| F82045 | 1.307 | 1.01 | 1.01 | 1333 | 679 | 689 |
| C83008 | 1.307 | 1.13 | 1.11 | 1334 | 1395 | 1250 |
| M83698 | 1.307 | 1.12 | 1.08 | 1335 | 1373 | 1086 |
| B86094 | 1.307 | 1.18 | 1.19 | 1336 | 1833 | 1943 |
| L81089 | 1.307 | 1.16 | 1.16 | 1337 | 1669 | 1669 |
| G82049 | 1.307 | 1.17 | 1.16 | 1338 | 1826 | 1684 |
| D83078 | 1.307 | 1.04 | 1.05 | 1339 | 854 | 904 |
| P81054 | 1.307 | 1.23 | 1.27 | 1340 | 2359 | 2876 |
| G82211 | 1.307 | 1.25 | 1.24 | 1341 | 2684 | 2568 |
| N85024 | 1.307 | 1.22 | 1.21 | 1342 | 2325 | 2148 |
| N84037 | 1.306 | 1.17 | 1.16 | 1343 | 1771 | 1666 |
| G82715 | 1.306 | 1.00 | 1.00 | 1344 | 660 | 651 |

| | | | | | | |
|---|---|---|---|---|---|---|
| K81608 | 1.306 | 1.05 | 1.07 | 1345 | 921 | 990 |
| M84050 | 1.306 | 1.12 | 1.10 | 1346 | 1338 | 1235 |
| G82648 | 1.306 | 1.05 | 1.06 | 1347 | 897 | 959 |
| M83111 | 1.306 | 1.49 | 1.45 | 1348 | 5749 | 5370 |
| D82085 | 1.306 | 1.06 | 1.06 | 1349 | 987 | 947 |
| M84065 | 1.306 | 1.15 | 1.14 | 1350 | 1623 | 1494 |
| M84008 | 1.306 | 1.02 | 0.99 | 1351 | 756 | 611 |
| F82022 | 1.305 | 1.07 | 1.07 | 1352 | 1045 | 1030 |
| L84042 | 1.305 | 1.21 | 1.20 | 1353 | 2256 | 2143 |
| C84712 | 1.305 | 1.01 | 1.03 | 1354 | 710 | 777 |
| P85007 | 1.305 | 1.19 | 1.21 | 1355 | 2028 | 2261 |
| E84009 | 1.305 | 1.12 | 1.08 | 1356 | 1336 | 1106 |
| M81070 | 1.305 | 1.13 | 1.10 | 1357 | 1427 | 1223 |
| L82047 | 1.304 | 1.27 | 1.26 | 1358 | 2908 | 2797 |
| F81164 | 1.304 | 1.10 | 1.11 | 1359 | 1223 | 1293 |
| P92007 | 1.304 | 1.25 | 1.25 | 1360 | 2658 | 2648 |
| G83007 | 1.304 | 1.10 | 1.09 | 1361 | 1232 | 1182 |
| N81074 | 1.304 | 1.20 | 1.17 | 1362 | 2133 | 1829 |
| P85025 | 1.304 | 1.09 | 1.11 | 1363 | 1159 | 1343 |
| M88623 | 1.303 | 1.13 | 1.14 | 1364 | 1425 | 1523 |
| N81057 | 1.303 | 1.20 | 1.21 | 1365 | 2082 | 2215 |
| M84620 | 1.303 | 1.00 | 1.01 | 1366 | 683 | 691 |
| E84713 | 1.303 | 1.06 | 1.03 | 1367 | 958 | 811 |
| C87609 | 1.303 | 1.27 | 1.27 | 1368 | 2902 | 2929 |
| B85611 | 1.303 | 1.24 | 1.25 | 1369 | 2638 | 2693 |
| A86005 | 1.303 | 1.23 | 1.24 | 1370 | 2512 | 2560 |
| G82091 | 1.303 | 1.07 | 1.10 | 1371 | 1065 | 1269 |
| E85735 | 1.303 | 1.00 | 0.99 | 1372 | 689 | 643 |
| K83004 | 1.303 | 1.06 | 1.02 | 1373 | 955 | 753 |
| P81607 | 1.302 | 1.15 | 1.19 | 1374 | 1663 | 1978 |
| P89023 | 1.302 | 1.14 | 1.16 | 1375 | 1572 | 1767 |
| G82006 | 1.302 | 1.00 | 1.01 | 1376 | 684 | 721 |
| E82004 | 1.302 | 1.02 | 1.01 | 1377 | 748 | 697 |
| C82073 | 1.302 | 1.08 | 1.08 | 1378 | 1099 | 1103 |
| P86605 | 1.302 | 1.10 | 1.11 | 1379 | 1207 | 1292 |
| G82667 | 1.302 | 0.98 | 0.99 | 1380 | 588 | 645 |
| M81083 | 1.302 | 1.11 | 1.08 | 1381 | 1329 | 1126 |
| M88618 | 1.302 | 1.08 | 1.10 | 1382 | 1135 | 1251 |
| C87031 | 1.302 | 1.41 | 1.39 | 1383 | 4863 | 4726 |
| F86066 | 1.302 | 0.96 | 0.94 | 1384 | 531 | 471 |
| L83113 | 1.301 | 1.19 | 1.20 | 1385 | 2083 | 2195 |
| Y02653 | 1.301 | 1.23 | 1.21 | 1386 | 2464 | 2234 |
| L81069 | 1.301 | 1.07 | 1.05 | 1387 | 1049 | 940 |
| D82024 | 1.301 | 1.04 | 1.03 | 1388 | 842 | 798 |
| A82004 | 1.301 | 1.16 | 1.13 | 1389 | 1746 | 1477 |
| M86028 | 1.301 | 1.29 | 1.28 | 1390 | 3298 | 3087 |
| P86013 | 1.301 | 1.14 | 1.16 | 1391 | 1569 | 1781 |
| F81081 | 1.301 | 1.10 | 1.11 | 1392 | 1284 | 1341 |
| M81017 | 1.300 | 1.04 | 1.05 | 1393 | 899 | 933 |
| P87032 | 1.300 | 1.18 | 1.20 | 1394 | 2003 | 2201 |

| | | | | | | |
|---|---|---|---|---|---|---|
| N82109 | 1.299 | 1.27 | 1.27 | 1395 | 3027 | 3083 |
| A83636 | 1.299 | 1.29 | 1.28 | 1396 | 3207 | 3160 |
| L85048 | 1.299 | 1.09 | 1.10 | 1397 | 1204 | 1238 |
| M91628 | 1.299 | 1.46 | 1.48 | 1398 | 5526 | 5769 |
| N81117 | 1.299 | 1.28 | 1.25 | 1399 | 3127 | 2721 |
| H83034 | 1.299 | 0.97 | 0.96 | 1400 | 560 | 536 |
| Y03656 | 1.298 | 1.19 | 1.17 | 1401 | 2027 | 1909 |
| L82617 | 1.298 | 1.21 | 1.21 | 1402 | 2306 | 2328 |
| A81006 | 1.298 | 1.18 | 1.18 | 1403 | 1994 | 1970 |
| C83037 | 1.298 | 1.11 | 1.10 | 1404 | 1316 | 1302 |
| J82090 | 1.298 | 1.10 | 1.11 | 1405 | 1259 | 1369 |
| D82044 | 1.298 | 1.27 | 1.26 | 1406 | 3019 | 2970 |
| A89604 | 1.298 | 1.49 | 1.48 | 1407 | 5874 | 5722 |
| P81659 | 1.298 | 1.06 | 1.10 | 1408 | 997 | 1297 |
| A88020 | 1.297 | 1.35 | 1.35 | 1409 | 4188 | 4114 |
| J81047 | 1.297 | 1.03 | 1.04 | 1410 | 843 | 860 |
| E86010 | 1.297 | 1.08 | 1.07 | 1411 | 1116 | 1055 |
| L81119 | 1.297 | 1.38 | 1.34 | 1412 | 4638 | 3963 |
| M85670 | 1.297 | 1.13 | 1.12 | 1413 | 1473 | 1439 |
| J82066 | 1.297 | 1.04 | 1.03 | 1414 | 878 | 829 |
| K82036 | 1.297 | 1.10 | 1.08 | 1415 | 1288 | 1141 |
| L83103 | 1.297 | 1.23 | 1.23 | 1416 | 2513 | 2513 |
| H85095 | 1.297 | 0.97 | 0.96 | 1417 | 568 | 538 |
| B81004 | 1.297 | 1.30 | 1.28 | 1418 | 3394 | 3238 |
| M81011 | 1.297 | 1.06 | 1.05 | 1419 | 1053 | 962 |
| F82648 | 1.296 | 1.11 | 1.10 | 1420 | 1370 | 1261 |
| E86019 | 1.296 | 1.14 | 1.13 | 1421 | 1629 | 1530 |
| A82062 | 1.296 | 1.24 | 1.26 | 1422 | 2625 | 2909 |
| P82018 | 1.296 | 1.18 | 1.18 | 1423 | 1956 | 1993 |
| C88095 | 1.296 | 1.20 | 1.21 | 1424 | 2190 | 2347 |
| G82744 | 1.296 | 1.04 | 1.06 | 1425 | 867 | 1005 |
| A89029 | 1.296 | 1.19 | 1.20 | 1426 | 2152 | 2202 |
| P84032 | 1.296 | 1.19 | 1.20 | 1427 | 2078 | 2236 |
| G82684 | 1.295 | 1.04 | 1.08 | 1428 | 895 | 1119 |
| C83617 | 1.295 | 1.10 | 1.10 | 1429 | 1272 | 1285 |
| C84078 | 1.295 | 1.09 | 1.11 | 1430 | 1201 | 1352 |
| P81735 | 1.295 | 1.07 | 1.06 | 1431 | 1081 | 1000 |
| E85064 | 1.295 | 1.12 | 1.12 | 1432 | 1443 | 1420 |
| H83023 | 1.295 | 1.03 | 1.02 | 1433 | 829 | 770 |
| K82025 | 1.295 | 1.02 | 1.01 | 1434 | 772 | 735 |
| G81057 | 1.295 | 1.22 | 1.17 | 1435 | 2405 | 1879 |
| M85127 | 1.295 | 1.26 | 1.26 | 1436 | 2897 | 2899 |
| G85723 | 1.295 | 0.96 | 0.96 | 1437 | 552 | 559 |
| M83638 | 1.295 | 1.05 | 1.08 | 1438 | 946 | 1156 |
| C84002 | 1.294 | 1.28 | 1.26 | 1439 | 3177 | 3013 |
| M86008 | 1.294 | 1.19 | 1.17 | 1440 | 2107 | 1880 |
| H81068 | 1.294 | 1.02 | 0.99 | 1441 | 781 | 653 |
| K83028 | 1.294 | 1.18 | 1.18 | 1442 | 2032 | 1987 |
| P81737 | 1.294 | 1.22 | 1.19 | 1443 | 2429 | 2091 |
| C83071 | 1.294 | 1.20 | 1.24 | 1444 | 2237 | 2726 |

| | | | | | | |
|---|---|---|---|---|---|---|
| P81035 | 1.294 | 1.37 | 1.37 | 1445 | 4442 | 4481 |
| F81090 | 1.294 | 1.05 | 1.01 | 1446 | 943 | 755 |
| F81219 | 1.294 | 1.08 | 1.09 | 1447 | 1172 | 1207 |
| M83030 | 1.293 | 1.09 | 1.09 | 1448 | 1213 | 1195 |
| P81099 | 1.293 | 1.13 | 1.14 | 1449 | 1558 | 1586 |
| H85637 | 1.293 | 0.94 | 0.92 | 1450 | 490 | 442 |
| C83038 | 1.293 | 1.09 | 1.09 | 1451 | 1239 | 1232 |
| M84062 | 1.293 | 1.04 | 1.04 | 1452 | 931 | 939 |
| P81121 | 1.293 | 1.26 | 1.25 | 1453 | 2950 | 2830 |
| C85003 | 1.293 | 1.26 | 1.27 | 1454 | 2964 | 3040 |
| A89013 | 1.293 | 1.43 | 1.42 | 1455 | 5238 | 5121 |
| F81065 | 1.293 | 1.00 | 0.98 | 1456 | 701 | 629 |
| P84040 | 1.293 | 1.06 | 1.08 | 1457 | 1055 | 1159 |
| F81645 | 1.293 | 1.17 | 1.17 | 1458 | 1928 | 1921 |
| E82083 | 1.292 | 1.05 | 1.03 | 1459 | 967 | 851 |
| C83626 | 1.292 | 1.02 | 1.06 | 1460 | 798 | 1026 |
| M85013 | 1.292 | 1.10 | 1.09 | 1461 | 1328 | 1217 |
| D83080 | 1.292 | 1.04 | 1.04 | 1462 | 903 | 926 |
| C84001 | 1.292 | 1.26 | 1.24 | 1463 | 2941 | 2766 |
| C81649 | 1.292 | 1.10 | 1.09 | 1464 | 1346 | 1249 |
| E81065 | 1.292 | 1.05 | 1.07 | 1465 | 1016 | 1097 |
| G83061 | 1.291 | 1.02 | 1.01 | 1466 | 799 | 759 |
| M81038 | 1.291 | 1.15 | 1.12 | 1467 | 1755 | 1466 |
| C83058 | 1.291 | 1.16 | 1.15 | 1468 | 1820 | 1732 |
| F81193 | 1.291 | 1.14 | 1.13 | 1469 | 1678 | 1524 |
| C83652 | 1.291 | 1.27 | 1.30 | 1470 | 3045 | 3504 |
| F81198 | 1.291 | 1.04 | 1.05 | 1471 | 932 | 967 |
| E85643 | 1.291 | 1.10 | 1.11 | 1472 | 1354 | 1372 |
| B85610 | 1.291 | 1.02 | 1.03 | 1473 | 815 | 833 |
| C87024 | 1.291 | 1.08 | 1.09 | 1474 | 1192 | 1282 |
| F82030 | 1.290 | 1.08 | 1.07 | 1475 | 1176 | 1136 |
| D82051 | 1.290 | 1.25 | 1.24 | 1476 | 2813 | 2711 |
| A82019 | 1.290 | 1.13 | 1.15 | 1477 | 1551 | 1778 |
| N81017 | 1.290 | 1.04 | 1.02 | 1478 | 926 | 824 |
| G85125 | 1.290 | 1.06 | 1.06 | 1479 | 1079 | 1054 |
| K83043 | 1.290 | 1.07 | 1.08 | 1480 | 1112 | 1192 |
| P86004 | 1.290 | 1.10 | 1.11 | 1481 | 1314 | 1438 |
| P84016 | 1.290 | 1.17 | 1.19 | 1482 | 1978 | 2194 |
| L84071 | 1.289 | 1.17 | 1.16 | 1483 | 1933 | 1811 |
| A89027 | 1.289 | 1.19 | 1.20 | 1484 | 2144 | 2298 |
| C84057 | 1.289 | 1.19 | 1.19 | 1485 | 2125 | 2156 |
| G81041 | 1.289 | 1.23 | 1.18 | 1486 | 2679 | 2067 |
| F85686 | 1.289 | 1.08 | 1.07 | 1487 | 1165 | 1114 |
| J82002 | 1.289 | 1.22 | 1.23 | 1488 | 2570 | 2615 |
| Y02867 | 1.289 | 1.13 | 1.16 | 1489 | 1577 | 1893 |
| D81645 | 1.289 | 1.14 | 1.13 | 1490 | 1628 | 1605 |
| N81068 | 1.289 | 1.16 | 1.14 | 1491 | 1902 | 1686 |
| M92010 | 1.289 | 1.05 | 1.07 | 1492 | 1027 | 1139 |
| A83632 | 1.288 | 1.36 | 1.35 | 1493 | 4440 | 4291 |
| C84709 | 1.288 | 1.06 | 1.05 | 1494 | 1094 | 1003 |

| | | | | | | |
|---|---|---|---|---|---|---|
| G82038 | 1.288 | 1.29 | 1.29 | 1495 | 3437 | 3458 |
| F81120 | 1.288 | 1.06 | 1.08 | 1496 | 1077 | 1189 |
| A83051 | 1.288 | 1.36 | 1.35 | 1497 | 4396 | 4299 |
| A81036 | 1.287 | 1.21 | 1.22 | 1498 | 2421 | 2483 |
| B82073 | 1.287 | 1.15 | 1.13 | 1499 | 1773 | 1572 |
| C84695 | 1.287 | 1.16 | 1.18 | 1500 | 1923 | 2057 |
| A82009 | 1.287 | 1.30 | 1.32 | 1501 | 3613 | 3959 |
| J83014 | 1.287 | 1.05 | 1.04 | 1502 | 1014 | 951 |
| C87608 | 1.287 | 1.12 | 1.14 | 1503 | 1531 | 1685 |
| M82059 | 1.287 | 1.05 | 1.07 | 1504 | 1008 | 1115 |
| F85666 | 1.287 | 1.06 | 1.05 | 1505 | 1063 | 1013 |
| M83629 | 1.287 | 1.26 | 1.28 | 1506 | 2987 | 3388 |
| C88039 | 1.287 | 1.47 | 1.47 | 1507 | 5784 | 5766 |
| E85707 | 1.287 | 1.11 | 1.10 | 1508 | 1437 | 1365 |
| M84029 | 1.286 | 1.01 | 1.01 | 1509 | 797 | 782 |
| A83007 | 1.286 | 1.38 | 1.37 | 1510 | 4727 | 4625 |
| J83642 | 1.286 | 1.16 | 1.14 | 1511 | 1889 | 1713 |
| M83020 | 1.286 | 0.98 | 0.98 | 1512 | 678 | 652 |
| E82657 | 1.285 | 1.04 | 1.05 | 1513 | 956 | 1022 |
| E85633 | 1.285 | 0.99 | 0.99 | 1514 | 699 | 716 |
| A81039 | 1.285 | 1.04 | 1.05 | 1515 | 936 | 1040 |
| N81070 | 1.285 | 1.12 | 1.10 | 1516 | 1506 | 1371 |
| P91625 | 1.285 | 1.34 | 1.33 | 1517 | 4239 | 4086 |
| M84066 | 1.285 | 1.02 | 1.02 | 1518 | 838 | 841 |
| M91036 | 1.285 | 1.22 | 1.23 | 1519 | 2639 | 2723 |
| B82060 | 1.284 | 1.15 | 1.15 | 1520 | 1828 | 1766 |
| B86015 | 1.284 | 1.11 | 1.13 | 1521 | 1482 | 1600 |
| D82029 | 1.284 | 1.06 | 1.05 | 1522 | 1084 | 1039 |
| B87018 | 1.284 | 1.12 | 1.14 | 1523 | 1556 | 1759 |
| Y01139 | 1.284 | 1.16 | 1.20 | 1524 | 1958 | 2342 |
| F83027 | 1.283 | 1.07 | 1.08 | 1525 | 1149 | 1230 |
| F83007 | 1.283 | 1.09 | 1.09 | 1526 | 1291 | 1311 |
| K83058 | 1.283 | 1.13 | 1.14 | 1527 | 1605 | 1730 |
| K84024 | 1.283 | 1.06 | 1.05 | 1528 | 1088 | 1045 |
| P82006 | 1.283 | 1.12 | 1.13 | 1529 | 1513 | 1623 |
| D81064 | 1.283 | 1.38 | 1.43 | 1530 | 4799 | 5356 |
| E84653 | 1.283 | 0.96 | 0.94 | 1531 | 573 | 515 |
| N82050 | 1.283 | 1.28 | 1.29 | 1532 | 3317 | 3487 |
| Y02710 | 1.282 | 0.99 | 1.01 | 1533 | 720 | 781 |
| P87639 | 1.282 | 1.24 | 1.25 | 1534 | 2785 | 2937 |
| D83027 | 1.282 | 1.02 | 1.03 | 1535 | 858 | 943 |
| B83028 | 1.282 | 1.16 | 1.18 | 1536 | 1940 | 2089 |
| H85076 | 1.282 | 1.15 | 1.14 | 1537 | 1867 | 1742 |
| A82623 | 1.282 | 1.24 | 1.25 | 1538 | 2911 | 2941 |
| H81620 | 1.281 | 0.96 | 0.95 | 1539 | 603 | 564 |
| B83602 | 1.281 | 1.09 | 1.11 | 1540 | 1352 | 1502 |
| N82041 | 1.281 | 1.27 | 1.28 | 1541 | 3167 | 3441 |
| B85030 | 1.281 | 1.12 | 1.13 | 1542 | 1585 | 1629 |
| M85059 | 1.281 | 1.31 | 1.28 | 1543 | 3855 | 3380 |
| M81010 | 1.281 | 1.23 | 1.21 | 1544 | 2698 | 2447 |

| | | | | | | |
|---|---|---|---|---|---|---|
| N81614 | 1.281 | 1.09 | 1.07 | 1545 | 1327 | 1193 |
| F86008 | 1.281 | 1.03 | 1.01 | 1546 | 901 | 814 |
| A81618 | 1.281 | 1.17 | 1.18 | 1547 | 2098 | 2140 |
| A89021 | 1.280 | 1.26 | 1.27 | 1548 | 3122 | 3293 |
| N84005 | 1.280 | 1.12 | 1.12 | 1549 | 1570 | 1594 |
| H82011 | 1.280 | 1.14 | 1.14 | 1550 | 1732 | 1754 |
| Y00955 | 1.280 | 1.34 | 1.38 | 1551 | 4288 | 4745 |
| M84618 | 1.280 | 1.10 | 1.09 | 1552 | 1386 | 1349 |
| E82115 | 1.280 | 1.02 | 1.02 | 1553 | 884 | 873 |
| H81132 | 1.280 | 0.97 | 0.97 | 1554 | 638 | 636 |
| M85146 | 1.280 | 1.12 | 1.11 | 1555 | 1566 | 1511 |
| K81019 | 1.279 | 1.03 | 1.01 | 1556 | 907 | 801 |
| G82058 | 1.279 | 1.08 | 1.07 | 1557 | 1286 | 1158 |
| J82127 | 1.279 | 1.18 | 1.21 | 1558 | 2217 | 2577 |
| G82018 | 1.279 | 1.08 | 1.12 | 1559 | 1271 | 1528 |
| A86037 | 1.279 | 1.45 | 1.44 | 1560 | 5626 | 5535 |
| H82069 | 1.279 | 1.24 | 1.23 | 1561 | 2922 | 2814 |
| N82049 | 1.279 | 1.34 | 1.34 | 1562 | 4261 | 4254 |
| Y02342 | 1.279 | 0.85 | 0.86 | 1563 | 324 | 337 |
| N85020 | 1.278 | 1.21 | 1.20 | 1564 | 2586 | 2453 |
| A82020 | 1.278 | 1.19 | 1.21 | 1565 | 2296 | 2587 |
| Y02344 | 1.278 | 1.19 | 1.23 | 1566 | 2301 | 2745 |
| A83073 | 1.278 | 1.42 | 1.42 | 1567 | 5375 | 5343 |
| J83035 | 1.278 | 1.07 | 1.08 | 1568 | 1209 | 1258 |
| B85638 | 1.278 | 1.13 | 1.13 | 1569 | 1661 | 1672 |
| K82026 | 1.278 | 1.18 | 1.17 | 1570 | 2206 | 2024 |
| P91629 | 1.277 | 1.11 | 1.12 | 1571 | 1509 | 1554 |
| M85015 | 1.277 | 1.05 | 1.05 | 1572 | 1086 | 1085 |
| P81152 | 1.277 | 1.04 | 1.04 | 1573 | 1046 | 1027 |
| A81058 | 1.277 | 1.13 | 1.15 | 1574 | 1693 | 1870 |
| N81052 | 1.277 | 1.10 | 1.09 | 1575 | 1414 | 1320 |
| C82614 | 1.277 | 1.08 | 1.09 | 1576 | 1312 | 1385 |
| N83013 | 1.277 | 1.08 | 1.10 | 1577 | 1294 | 1408 |
| M92004 | 1.277 | 1.14 | 1.16 | 1578 | 1827 | 2004 |
| E85697 | 1.277 | 1.03 | 1.02 | 1579 | 982 | 900 |
| N85019 | 1.277 | 1.22 | 1.21 | 1580 | 2622 | 2491 |
| P81087 | 1.277 | 1.47 | 1.45 | 1581 | 5829 | 5596 |
| G82753 | 1.276 | 0.99 | 1.01 | 1582 | 735 | 838 |
| C87620 | 1.276 | 1.37 | 1.37 | 1583 | 4770 | 4735 |
| B83617 | 1.276 | 1.22 | 1.24 | 1584 | 2660 | 2897 |
| M91013 | 1.276 | 1.23 | 1.24 | 1585 | 2823 | 2919 |
| M81094 | 1.276 | 1.07 | 1.05 | 1586 | 1178 | 1095 |
| M83036 | 1.276 | 1.14 | 1.13 | 1587 | 1829 | 1665 |
| A87007 | 1.276 | 1.19 | 1.20 | 1588 | 2329 | 2477 |
| K82020 | 1.276 | 1.06 | 1.04 | 1589 | 1123 | 1002 |
| P84645 | 1.276 | 1.20 | 1.22 | 1590 | 2419 | 2630 |
| C84024 | 1.276 | 1.14 | 1.13 | 1591 | 1776 | 1699 |
| P81143 | 1.276 | 1.11 | 1.08 | 1592 | 1487 | 1317 |
| M91640 | 1.276 | 1.28 | 1.29 | 1593 | 3455 | 3664 |
| G82110 | 1.275 | 0.96 | 0.95 | 1594 | 622 | 581 |

| | | | | | | |
|---|---|---|---|---|---|---|
| L84606 | 1.275 | 1.09 | 1.08 | 1595 | 1344 | 1289 |
| M91025 | 1.275 | 1.10 | 1.11 | 1596 | 1468 | 1548 |
| H82020 | 1.275 | 1.13 | 1.12 | 1597 | 1738 | 1620 |
| B85036 | 1.275 | 1.21 | 1.20 | 1598 | 2544 | 2499 |
| Y02581 | 1.275 | 1.04 | 1.02 | 1599 | 1034 | 894 |
| C88090 | 1.275 | 1.33 | 1.33 | 1600 | 4179 | 4155 |
| N81035 | 1.275 | 1.20 | 1.21 | 1601 | 2395 | 2547 |
| K83021 | 1.275 | 1.09 | 1.09 | 1602 | 1381 | 1357 |
| N85044 | 1.275 | 1.16 | 1.15 | 1603 | 1988 | 1883 |
| A88011 | 1.275 | 1.26 | 1.25 | 1604 | 3140 | 3079 |
| C83635 | 1.275 | 1.31 | 1.33 | 1605 | 3886 | 4196 |
| C82116 | 1.274 | 1.29 | 1.29 | 1606 | 3692 | 3697 |
| J81035 | 1.274 | 1.20 | 1.19 | 1607 | 2400 | 2336 |
| B86048 | 1.274 | 1.12 | 1.13 | 1608 | 1579 | 1765 |
| H85116 | 1.274 | 0.95 | 0.95 | 1609 | 607 | 586 |
| N81053 | 1.274 | 1.08 | 1.06 | 1610 | 1283 | 1176 |
| P82030 | 1.274 | 1.15 | 1.16 | 1611 | 1914 | 2048 |
| A81019 | 1.274 | 1.21 | 1.22 | 1612 | 2569 | 2691 |
| H85113 | 1.274 | 1.03 | 1.02 | 1613 | 973 | 927 |
| A86024 | 1.274 | 1.06 | 1.08 | 1614 | 1180 | 1271 |
| C84045 | 1.273 | 1.12 | 1.11 | 1615 | 1586 | 1549 |
| B84021 | 1.273 | 1.13 | 1.15 | 1616 | 1761 | 1926 |
| K83619 | 1.273 | 0.93 | 0.95 | 1617 | 534 | 588 |
| Y02469 | 1.273 | 0.96 | 0.97 | 1618 | 623 | 649 |
| B85010 | 1.273 | 1.12 | 1.12 | 1619 | 1601 | 1604 |
| D81048 | 1.272 | 1.09 | 1.07 | 1620 | 1361 | 1267 |
| N82051 | 1.272 | 1.15 | 1.17 | 1621 | 1980 | 2123 |
| M81019 | 1.272 | 1.18 | 1.20 | 1622 | 2225 | 2438 |
| G82138 | 1.272 | 1.15 | 1.16 | 1623 | 1931 | 2061 |
| C85014 | 1.272 | 1.21 | 1.22 | 1624 | 2597 | 2702 |
| M86041 | 1.271 | 1.19 | 1.18 | 1625 | 2316 | 2241 |
| B81037 | 1.271 | 1.24 | 1.23 | 1626 | 2920 | 2860 |
| N84613 | 1.271 | 1.18 | 1.18 | 1627 | 2259 | 2199 |
| E82107 | 1.271 | 0.99 | 0.98 | 1628 | 752 | 704 |
| Y00758 | 1.271 | 1.12 | 1.13 | 1629 | 1686 | 1736 |
| M83011 | 1.271 | 1.20 | 1.16 | 1630 | 2526 | 2022 |
| B86016 | 1.271 | 1.20 | 1.22 | 1631 | 2538 | 2687 |
| N81618 | 1.271 | 1.22 | 1.22 | 1632 | 2720 | 2771 |
| N84036 | 1.271 | 0.95 | 0.96 | 1633 | 619 | 642 |
| E81041 | 1.271 | 1.08 | 1.09 | 1634 | 1287 | 1364 |
| C82680 | 1.270 | 1.20 | 1.20 | 1635 | 2468 | 2500 |
| A87005 | 1.270 | 1.12 | 1.13 | 1636 | 1638 | 1787 |
| B81075 | 1.270 | 0.95 | 0.99 | 1637 | 585 | 765 |
| B85038 | 1.270 | 1.16 | 1.15 | 1638 | 2017 | 1934 |
| C84613 | 1.270 | 1.00 | 1.01 | 1639 | 834 | 830 |
| M83044 | 1.270 | 1.16 | 1.13 | 1640 | 2033 | 1796 |
| P81085 | 1.270 | 1.28 | 1.29 | 1641 | 3610 | 3672 |
| A81011 | 1.270 | 1.34 | 1.36 | 1642 | 4342 | 4734 |
| P86608 | 1.270 | 1.06 | 1.08 | 1643 | 1197 | 1330 |
| N82115 | 1.270 | 1.38 | 1.37 | 1644 | 4912 | 4857 |

| | | | | | | |
|---|---|---|---|---|---|---|
| H85087 | 1.269 | 1.14 | 1.11 | 1645 | 1851 | 1565 |
| C84672 | 1.269 | 1.01 | 1.03 | 1646 | 874 | 1016 |
| C83052 | 1.269 | 1.10 | 1.10 | 1647 | 1471 | 1485 |
| F81151 | 1.269 | 1.22 | 1.22 | 1648 | 2794 | 2737 |
| M86002 | 1.269 | 1.24 | 1.22 | 1649 | 2951 | 2748 |
| B82049 | 1.269 | 1.11 | 1.10 | 1650 | 1600 | 1509 |
| B85044 | 1.269 | 1.17 | 1.17 | 1651 | 2163 | 2227 |
| C85026 | 1.269 | 1.23 | 1.24 | 1652 | 2880 | 3026 |
| Y02644 | 1.269 | 1.20 | 1.21 | 1653 | 2564 | 2618 |
| P82629 | 1.268 | 1.24 | 1.25 | 1654 | 3074 | 3187 |
| Y03079 | 1.268 | 1.16 | 1.17 | 1655 | 2106 | 2220 |
| C83082 | 1.268 | 1.14 | 1.18 | 1656 | 1880 | 2354 |
| F81128 | 1.268 | 1.18 | 1.18 | 1657 | 2275 | 2326 |
| C87020 | 1.268 | 1.26 | 1.26 | 1658 | 3341 | 3363 |
| D83608 | 1.268 | 1.18 | 1.18 | 1659 | 2339 | 2279 |
| M81072 | 1.268 | 1.06 | 1.07 | 1660 | 1212 | 1272 |
| E81076 | 1.268 | 1.12 | 1.13 | 1661 | 1681 | 1758 |
| H81118 | 1.267 | 1.04 | 1.02 | 1662 | 1083 | 912 |
| N85012 | 1.267 | 1.15 | 1.14 | 1663 | 2004 | 1871 |
| G82719 | 1.267 | 0.98 | 1.01 | 1664 | 717 | 850 |
| H81076 | 1.267 | 1.02 | 1.02 | 1665 | 974 | 977 |
| F84010 | 1.267 | 0.99 | 0.97 | 1666 | 784 | 703 |
| E84044 | 1.267 | 1.02 | 1.00 | 1667 | 947 | 809 |
| P84640 | 1.267 | 1.06 | 1.09 | 1668 | 1191 | 1398 |
| P81040 | 1.267 | 1.13 | 1.12 | 1669 | 1736 | 1711 |
| F84083 | 1.267 | 1.05 | 1.05 | 1670 | 1147 | 1112 |
| D81021 | 1.266 | 1.12 | 1.11 | 1671 | 1710 | 1612 |
| C85020 | 1.266 | 1.11 | 1.12 | 1672 | 1589 | 1725 |
| P81057 | 1.266 | 1.12 | 1.11 | 1673 | 1667 | 1609 |
| A89036 | 1.266 | 1.22 | 1.22 | 1674 | 2748 | 2764 |
| N85013 | 1.266 | 1.14 | 1.14 | 1675 | 1886 | 1851 |
| G81026 | 1.266 | 1.13 | 1.16 | 1676 | 1811 | 2130 |
| P81005 | 1.266 | 1.30 | 1.32 | 1677 | 3856 | 4229 |
| N81107 | 1.265 | 1.18 | 1.16 | 1678 | 2285 | 2129 |
| C84052 | 1.265 | 1.05 | 1.05 | 1679 | 1155 | 1178 |
| P81070 | 1.265 | 1.28 | 1.32 | 1680 | 3701 | 4243 |
| K84613 | 1.265 | 0.93 | 0.93 | 1681 | 544 | 551 |
| B85011 | 1.265 | 1.05 | 1.06 | 1682 | 1166 | 1220 |
| P81137 | 1.265 | 1.17 | 1.19 | 1683 | 2209 | 2399 |
| E82061 | 1.265 | 1.06 | 1.06 | 1684 | 1217 | 1196 |
| N85023 | 1.265 | 1.21 | 1.20 | 1685 | 2730 | 2572 |
| D83082 | 1.265 | 1.08 | 1.08 | 1686 | 1397 | 1403 |
| B86057 | 1.265 | 1.09 | 1.11 | 1687 | 1452 | 1574 |
| H83012 | 1.264 | 0.98 | 0.97 | 1688 | 762 | 711 |
| N81059 | 1.264 | 1.08 | 1.07 | 1689 | 1349 | 1304 |
| C83011 | 1.264 | 1.14 | 1.14 | 1690 | 1918 | 1882 |
| J83034 | 1.264 | 1.11 | 1.10 | 1691 | 1615 | 1521 |
| P88018 | 1.264 | 1.12 | 1.12 | 1692 | 1747 | 1690 |
| E81021 | 1.264 | 1.17 | 1.16 | 1693 | 2263 | 2117 |
| M86026 | 1.264 | 1.52 | 1.47 | 1694 | 6471 | 5962 |

| | | | | | | |
|---|---|---|---|---|---|---|
| B81119 | 1.264 | 1.19 | 1.24 | 1695 | 2486 | 3038 |
| C82028 | 1.264 | 1.17 | 1.17 | 1696 | 2260 | 2226 |
| L81032 | 1.263 | 1.14 | 1.14 | 1697 | 1913 | 1898 |
| P83021 | 1.263 | 1.13 | 1.13 | 1698 | 1798 | 1788 |
| N81025 | 1.263 | 1.13 | 1.10 | 1699 | 1783 | 1571 |
| N83001 | 1.263 | 1.21 | 1.22 | 1700 | 2744 | 2841 |
| P85018 | 1.263 | 1.24 | 1.26 | 1701 | 3113 | 3324 |
| E86005 | 1.263 | 1.07 | 1.06 | 1702 | 1322 | 1246 |
| B83628 | 1.263 | 1.11 | 1.12 | 1703 | 1588 | 1772 |
| M88014 | 1.263 | 1.22 | 1.22 | 1704 | 2841 | 2869 |
| G81019 | 1.263 | 0.99 | 0.98 | 1705 | 831 | 733 |
| L81067 | 1.263 | 1.18 | 1.18 | 1706 | 2314 | 2318 |
| E84068 | 1.263 | 1.10 | 1.07 | 1707 | 1505 | 1334 |
| M84003 | 1.263 | 1.10 | 1.10 | 1708 | 1521 | 1504 |
| F86691 | 1.262 | 1.06 | 1.05 | 1709 | 1258 | 1142 |
| N81065 | 1.262 | 1.11 | 1.10 | 1710 | 1670 | 1551 |
| F85652 | 1.262 | 0.99 | 0.98 | 1711 | 806 | 760 |
| C82082 | 1.262 | 1.16 | 1.16 | 1712 | 2092 | 2097 |
| G83010 | 1.262 | 1.18 | 1.16 | 1713 | 2374 | 2124 |
| N81015 | 1.262 | 1.08 | 1.06 | 1714 | 1371 | 1243 |
| L84073 | 1.262 | 1.22 | 1.21 | 1715 | 2894 | 2696 |
| M83042 | 1.262 | 1.09 | 1.06 | 1716 | 1488 | 1239 |
| A82647 | 1.261 | 1.24 | 1.20 | 1717 | 3085 | 2644 |
| D82035 | 1.261 | 1.32 | 1.31 | 1718 | 4228 | 4192 |
| F85045 | 1.261 | 0.86 | 0.87 | 1719 | 374 | 384 |
| K83005 | 1.261 | 1.12 | 1.15 | 1720 | 1713 | 2035 |
| Y00140 | 1.261 | 1.37 | 1.35 | 1721 | 4964 | 4633 |
| M84005 | 1.261 | 1.05 | 1.05 | 1722 | 1195 | 1180 |
| P81067 | 1.261 | 1.11 | 1.11 | 1723 | 1676 | 1613 |
| B83055 | 1.260 | 1.17 | 1.18 | 1724 | 2222 | 2346 |
| A83008 | 1.260 | 1.46 | 1.44 | 1725 | 5867 | 5733 |
| P92041 | 1.260 | 1.09 | 1.10 | 1726 | 1486 | 1598 |
| B86003 | 1.260 | 1.03 | 1.04 | 1727 | 1051 | 1117 |
| A81016 | 1.260 | 1.12 | 1.14 | 1728 | 1765 | 1928 |
| J82165 | 1.260 | 1.07 | 1.08 | 1729 | 1311 | 1414 |
| A81047 | 1.260 | 1.24 | 1.24 | 1730 | 3089 | 3109 |
| G82075 | 1.260 | 1.04 | 1.04 | 1731 | 1127 | 1116 |
| C84051 | 1.259 | 1.06 | 1.09 | 1732 | 1224 | 1467 |
| P81664 | 1.259 | 1.20 | 1.18 | 1733 | 2592 | 2444 |
| H81644 | 1.259 | 0.97 | 0.94 | 1734 | 719 | 627 |
| C84043 | 1.259 | 1.19 | 1.20 | 1735 | 2494 | 2603 |
| C82081 | 1.259 | 1.13 | 1.13 | 1736 | 1822 | 1838 |
| P86010 | 1.259 | 1.19 | 1.21 | 1737 | 2490 | 2701 |
| N84615 | 1.259 | 1.25 | 1.24 | 1738 | 3285 | 3127 |
| E85623 | 1.259 | 1.06 | 1.07 | 1739 | 1303 | 1342 |
| H81618 | 1.258 | 1.07 | 1.04 | 1740 | 1325 | 1104 |
| N85053 | 1.258 | 1.13 | 1.12 | 1741 | 1908 | 1790 |
| L85030 | 1.258 | 1.18 | 1.18 | 1742 | 2372 | 2382 |
| A82058 | 1.258 | 1.21 | 1.24 | 1743 | 2825 | 3180 |
| P86018 | 1.258 | 1.23 | 1.25 | 1744 | 3030 | 3282 |

| | | | | | | |
|---|---|---|---|---|---|---|
| Y02477 | 1.258 | 1.04 | 1.05 | 1745 | 1136 | 1216 |
| H81133 | 1.258 | 0.98 | 0.96 | 1746 | 786 | 678 |
| M89001 | 1.258 | 1.19 | 1.15 | 1747 | 2584 | 2049 |
| A82005 | 1.258 | 1.04 | 1.01 | 1748 | 1107 | 975 |
| C81014 | 1.258 | 1.25 | 1.24 | 1749 | 3251 | 3213 |
| E81057 | 1.257 | 1.08 | 1.08 | 1750 | 1432 | 1406 |
| N84035 | 1.257 | 1.22 | 1.21 | 1751 | 2910 | 2717 |
| M81045 | 1.257 | 1.09 | 1.07 | 1752 | 1530 | 1356 |
| M84043 | 1.257 | 1.01 | 1.02 | 1753 | 971 | 998 |
| F81606 | 1.257 | 1.27 | 1.28 | 1754 | 3680 | 3694 |
| L83642 | 1.256 | 1.17 | 1.19 | 1755 | 2330 | 2550 |
| J82132 | 1.256 | 1.12 | 1.10 | 1756 | 1766 | 1616 |
| P88010 | 1.256 | 1.84 | 1.76 | 1757 | 7266 | 7216 |
| E82051 | 1.256 | 1.03 | 1.02 | 1758 | 1073 | 1012 |
| G82085 | 1.256 | 1.03 | 1.02 | 1759 | 1101 | 1031 |
| F85004 | 1.255 | 1.14 | 1.13 | 1760 | 2008 | 1885 |
| B81005 | 1.255 | 1.20 | 1.22 | 1761 | 2655 | 2907 |
| G85106 | 1.255 | 1.04 | 1.04 | 1762 | 1162 | 1150 |
| D82028 | 1.255 | 1.26 | 1.23 | 1763 | 3518 | 3111 |
| P84056 | 1.254 | 1.14 | 1.16 | 1764 | 2016 | 2189 |
| B81635 | 1.254 | 1.27 | 1.31 | 1765 | 3574 | 4286 |
| E81006 | 1.254 | 1.01 | 1.02 | 1766 | 994 | 1032 |
| B83042 | 1.254 | 1.26 | 1.25 | 1767 | 3457 | 3379 |
| F81036 | 1.254 | 1.03 | 1.04 | 1768 | 1111 | 1172 |
| P81184 | 1.254 | 1.14 | 1.12 | 1769 | 1970 | 1839 |
| P81089 | 1.254 | 1.34 | 1.33 | 1770 | 4675 | 4463 |
| J82004 | 1.254 | 1.13 | 1.14 | 1771 | 1868 | 1964 |
| K82012 | 1.254 | 1.04 | 1.02 | 1772 | 1171 | 1050 |
| P81046 | 1.254 | 1.10 | 1.10 | 1773 | 1649 | 1585 |
| E81036 | 1.254 | 1.14 | 1.13 | 1774 | 2019 | 1935 |
| K83024 | 1.253 | 1.15 | 1.14 | 1775 | 2097 | 2047 |
| M88643 | 1.253 | 1.09 | 1.11 | 1776 | 1547 | 1682 |
| C83650 | 1.253 | 1.31 | 1.33 | 1777 | 4240 | 4526 |
| L83071 | 1.253 | 1.11 | 1.12 | 1778 | 1687 | 1820 |
| D81008 | 1.253 | 1.20 | 1.24 | 1779 | 2691 | 3255 |
| N81072 | 1.253 | 1.35 | 1.34 | 1780 | 4750 | 4619 |
| D81084 | 1.253 | 1.01 | 1.00 | 1781 | 995 | 880 |
| L82043 | 1.253 | 1.04 | 1.05 | 1782 | 1198 | 1236 |
| P81159 | 1.253 | 1.41 | 1.45 | 1783 | 5518 | 5853 |
| M87021 | 1.253 | 1.10 | 1.10 | 1784 | 1655 | 1614 |
| D81046 | 1.252 | 1.12 | 1.17 | 1785 | 1859 | 2312 |
| N81628 | 1.252 | 1.09 | 1.08 | 1786 | 1543 | 1447 |
| P81039 | 1.252 | 1.11 | 1.12 | 1787 | 1727 | 1814 |
| M83001 | 1.252 | 1.21 | 1.24 | 1788 | 2843 | 3234 |
| Y00260 | 1.252 | 1.01 | 1.01 | 1789 | 1000 | 974 |
| K83036 | 1.252 | 1.14 | 1.14 | 1790 | 2090 | 1983 |
| G82888 | 1.252 | 0.87 | 0.87 | 1791 | 431 | 408 |
| Y02842 | 1.252 | 0.83 | 0.83 | 1792 | 348 | 335 |
| L85020 | 1.252 | 1.09 | 1.07 | 1793 | 1567 | 1427 |
| M91008 | 1.252 | 1.18 | 1.19 | 1794 | 2441 | 2628 |

| | | | | | | |
|---|---|---|---|---|---|---|
| K83069 | 1.252 | 1.39 | 1.38 | 1795 | 5278 | 5094 |
| B81002 | 1.251 | 1.19 | 1.23 | 1796 | 2578 | 3131 |
| A81610 | 1.251 | 1.09 | 1.10 | 1797 | 1584 | 1657 |
| P81186 | 1.251 | 1.26 | 1.24 | 1798 | 3465 | 3231 |
| A87016 | 1.251 | 1.26 | 1.26 | 1799 | 3482 | 3615 |
| F85044 | 1.251 | 1.03 | 1.02 | 1800 | 1100 | 1078 |
| C83078 | 1.251 | 1.07 | 1.12 | 1801 | 1417 | 1807 |
| P87657 | 1.251 | 1.23 | 1.25 | 1802 | 3151 | 3461 |
| B85051 | 1.251 | 1.12 | 1.12 | 1803 | 1854 | 1856 |
| P82624 | 1.251 | 1.10 | 1.11 | 1804 | 1644 | 1746 |
| M83108 | 1.251 | 1.13 | 1.08 | 1805 | 1932 | 1491 |
| D81004 | 1.251 | 1.13 | 1.13 | 1806 | 1996 | 1920 |
| H85065 | 1.251 | 0.98 | 0.96 | 1807 | 826 | 701 |
| H85653 | 1.251 | 1.08 | 1.07 | 1808 | 1504 | 1419 |
| M83023 | 1.251 | 1.17 | 1.18 | 1809 | 2437 | 2482 |
| J82178 | 1.250 | 1.01 | 1.01 | 1810 | 983 | 968 |
| G82215 | 1.250 | 1.02 | 1.02 | 1811 | 1039 | 1068 |
| F81697 | 1.250 | 1.10 | 1.10 | 1812 | 1683 | 1693 |
| H81055 | 1.250 | 1.07 | 1.06 | 1813 | 1423 | 1299 |
| B81031 | 1.250 | 1.11 | 1.14 | 1814 | 1777 | 2001 |
| P92008 | 1.250 | 1.32 | 1.33 | 1815 | 4426 | 4485 |
| L83015 | 1.250 | 1.15 | 1.16 | 1816 | 2192 | 2321 |
| E81004 | 1.249 | 1.05 | 1.04 | 1817 | 1233 | 1211 |
| C83035 | 1.249 | 1.11 | 1.10 | 1818 | 1741 | 1627 |
| N81094 | 1.249 | 1.17 | 1.14 | 1819 | 2385 | 2050 |
| F81122 | 1.249 | 1.07 | 1.07 | 1820 | 1426 | 1381 |
| P89013 | 1.249 | 1.40 | 1.41 | 1821 | 5390 | 5498 |
| J82115 | 1.249 | 1.06 | 1.06 | 1822 | 1317 | 1360 |
| A88613 | 1.249 | 1.34 | 1.31 | 1823 | 4671 | 4326 |
| F81035 | 1.249 | 1.06 | 1.06 | 1824 | 1362 | 1337 |
| J82122 | 1.249 | 1.09 | 1.10 | 1825 | 1606 | 1692 |
| M83113 | 1.249 | 1.17 | 1.15 | 1826 | 2422 | 2209 |
| C83044 | 1.249 | 1.22 | 1.21 | 1827 | 3026 | 2902 |
| C85623 | 1.249 | 0.95 | 0.97 | 1828 | 670 | 762 |
| D82041 | 1.248 | 1.11 | 1.09 | 1829 | 1762 | 1626 |
| P84631 | 1.248 | 1.15 | 1.17 | 1830 | 2215 | 2465 |
| G83006 | 1.248 | 1.11 | 1.09 | 1831 | 1737 | 1622 |
| G81061 | 1.248 | 1.17 | 1.14 | 1832 | 2394 | 2104 |
| C84667 | 1.248 | 1.14 | 1.13 | 1833 | 2066 | 1959 |
| A82053 | 1.248 | 1.16 | 1.13 | 1834 | 2318 | 1991 |
| N81067 | 1.248 | 1.03 | 1.01 | 1835 | 1146 | 1021 |
| C86030 | 1.247 | 1.34 | 1.36 | 1836 | 4741 | 4964 |
| P86003 | 1.247 | 1.07 | 1.09 | 1837 | 1404 | 1599 |
| C88010 | 1.247 | 1.17 | 1.18 | 1838 | 2361 | 2582 |
| L81094 | 1.247 | 1.32 | 1.31 | 1839 | 4500 | 4310 |
| B82081 | 1.247 | 1.03 | 1.03 | 1840 | 1161 | 1144 |
| E82043 | 1.247 | 1.04 | 1.05 | 1841 | 1219 | 1305 |
| M84617 | 1.247 | 1.01 | 1.01 | 1842 | 1022 | 1041 |
| A82063 | 1.247 | 1.03 | 1.04 | 1843 | 1173 | 1218 |
| C86020 | 1.247 | 1.18 | 1.21 | 1844 | 2573 | 2891 |

| | | | | | | |
|---|---|---|---|---|---|---|
| P92011 | 1.247 | 1.18 | 1.18 | 1845 | 2507 | 2570 |
| M83064 | 1.247 | 1.08 | 1.06 | 1846 | 1470 | 1394 |
| C83057 | 1.246 | 1.23 | 1.21 | 1847 | 3121 | 2946 |
| A83033 | 1.246 | 1.25 | 1.25 | 1848 | 3466 | 3517 |
| D81032 | 1.246 | 1.09 | 1.09 | 1849 | 1641 | 1564 |
| C81082 | 1.246 | 1.06 | 1.06 | 1850 | 1367 | 1382 |
| M85179 | 1.246 | 1.20 | 1.18 | 1851 | 2787 | 2546 |
| M84024 | 1.246 | 1.08 | 1.08 | 1852 | 1528 | 1531 |
| A81612 | 1.246 | 1.21 | 1.24 | 1853 | 2878 | 3393 |
| J83008 | 1.246 | 1.07 | 1.06 | 1854 | 1446 | 1380 |
| B87008 | 1.246 | 1.28 | 1.30 | 1855 | 3887 | 4167 |
| Y00902 | 1.246 | 0.92 | 0.92 | 1856 | 589 | 578 |
| E81059 | 1.246 | 1.13 | 1.13 | 1857 | 1989 | 1955 |
| P87022 | 1.246 | 1.27 | 1.28 | 1858 | 3840 | 3984 |
| C88656 | 1.246 | 1.18 | 1.20 | 1859 | 2565 | 2820 |
| J82060 | 1.245 | 1.13 | 1.14 | 1860 | 2051 | 2120 |
| A83003 | 1.245 | 1.29 | 1.29 | 1861 | 4005 | 4093 |
| E81018 | 1.245 | 1.04 | 1.05 | 1862 | 1227 | 1310 |
| J81050 | 1.245 | 1.18 | 1.17 | 1863 | 2552 | 2474 |
| H85005 | 1.245 | 0.99 | 0.97 | 1864 | 883 | 767 |
| P84052 | 1.245 | 1.21 | 1.22 | 1865 | 2982 | 3094 |
| D82081 | 1.245 | 1.23 | 1.25 | 1866 | 3161 | 3435 |
| K83077 | 1.245 | 1.13 | 1.15 | 1867 | 2026 | 2177 |
| Y01695 | 1.245 | 1.03 | 1.04 | 1868 | 1163 | 1244 |
| D81025 | 1.245 | 1.09 | 1.05 | 1869 | 1643 | 1327 |
| K83003 | 1.245 | 1.07 | 1.08 | 1870 | 1467 | 1547 |
| M82021 | 1.245 | 1.26 | 1.26 | 1871 | 3658 | 3633 |
| G81030 | 1.245 | 0.99 | 0.98 | 1872 | 918 | 820 |
| P81170 | 1.244 | 1.21 | 1.25 | 1873 | 2989 | 3553 |
| P81103 | 1.244 | 1.24 | 1.22 | 1874 | 3302 | 3121 |
| G82126 | 1.244 | 1.17 | 1.18 | 1875 | 2402 | 2588 |
| A82055 | 1.244 | 1.27 | 1.27 | 1876 | 3802 | 3804 |
| N81005 | 1.244 | 1.11 | 1.10 | 1877 | 1852 | 1668 |
| F81711 | 1.244 | 1.43 | 1.42 | 1878 | 5738 | 5624 |
| H81024 | 1.244 | 0.96 | 0.96 | 1879 | 744 | 752 |
| Y02815 | 1.244 | 1.23 | 1.23 | 1880 | 3152 | 3275 |
| H82006 | 1.244 | 1.14 | 1.14 | 1881 | 2088 | 2176 |
| M92040 | 1.243 | 1.10 | 1.12 | 1882 | 1731 | 1876 |
| K81024 | 1.243 | 1.04 | 1.05 | 1883 | 1240 | 1295 |
| M89027 | 1.243 | 1.29 | 1.23 | 1884 | 4017 | 3256 |
| D82098 | 1.243 | 1.29 | 1.29 | 1885 | 4129 | 4115 |
| J83057 | 1.243 | 1.07 | 1.08 | 1886 | 1448 | 1513 |
| C88060 | 1.243 | 1.19 | 1.22 | 1887 | 2758 | 3067 |
| B86643 | 1.243 | 1.31 | 1.31 | 1888 | 4301 | 4390 |
| D81024 | 1.243 | 1.16 | 1.16 | 1889 | 2291 | 2334 |
| G85120 | 1.243 | 1.01 | 1.02 | 1890 | 1040 | 1079 |
| J81625 | 1.243 | 1.04 | 1.04 | 1891 | 1228 | 1222 |
| J83047 | 1.243 | 1.06 | 1.07 | 1892 | 1357 | 1441 |
| P81692 | 1.242 | 1.22 | 1.21 | 1893 | 3136 | 2924 |
| P91604 | 1.242 | 1.00 | 1.01 | 1894 | 1001 | 1056 |

| | | | | | | |
|---|---|---|---|---|---|---|
| N81109 | 1.242 | 1.07 | 1.07 | 1895 | 1484 | 1456 |
| B83035 | 1.242 | 1.19 | 1.20 | 1896 | 2745 | 2846 |
| D82026 | 1.242 | 1.13 | 1.14 | 1897 | 2025 | 2165 |
| E82082 | 1.242 | 1.14 | 1.12 | 1898 | 2111 | 1941 |
| N82113 | 1.242 | 1.09 | 1.11 | 1899 | 1682 | 1816 |
| N81091 | 1.242 | 1.00 | 0.99 | 1900 | 1021 | 899 |
| D81081 | 1.242 | 1.15 | 1.14 | 1901 | 2277 | 2154 |
| L83112 | 1.242 | 1.18 | 1.19 | 1902 | 2604 | 2743 |
| J83005 | 1.242 | 1.06 | 1.05 | 1903 | 1377 | 1294 |
| G83047 | 1.241 | 0.99 | 0.98 | 1904 | 933 | 872 |
| C88011 | 1.241 | 1.34 | 1.34 | 1905 | 4763 | 4781 |
| M87623 | 1.241 | 1.11 | 1.11 | 1906 | 1796 | 1805 |
| J81073 | 1.241 | 1.21 | 1.21 | 1907 | 3025 | 3002 |
| N83053 | 1.241 | 1.08 | 1.10 | 1908 | 1578 | 1769 |
| J82198 | 1.241 | 1.06 | 1.05 | 1909 | 1390 | 1323 |
| P87015 | 1.241 | 1.48 | 1.49 | 1910 | 6224 | 6338 |
| M92002 | 1.241 | 1.30 | 1.31 | 1911 | 4306 | 4425 |
| B85028 | 1.241 | 1.17 | 1.17 | 1912 | 2471 | 2467 |
| G82604 | 1.240 | 1.03 | 1.03 | 1913 | 1214 | 1215 |
| M81056 | 1.240 | 1.14 | 1.12 | 1914 | 2137 | 1951 |
| G81089 | 1.240 | 1.06 | 1.08 | 1915 | 1368 | 1610 |
| F81072 | 1.240 | 1.04 | 1.02 | 1916 | 1238 | 1102 |
| B86678 | 1.240 | 1.13 | 1.14 | 1917 | 2015 | 2142 |
| E86640 | 1.240 | 0.99 | 0.98 | 1918 | 917 | 848 |
| D83026 | 1.240 | 1.05 | 1.06 | 1919 | 1323 | 1397 |
| A89031 | 1.240 | 1.19 | 1.19 | 1920 | 2760 | 2775 |
| B83026 | 1.240 | 1.09 | 1.11 | 1921 | 1677 | 1843 |
| H81638 | 1.239 | 1.01 | 0.99 | 1922 | 1068 | 920 |
| G81027 | 1.239 | 1.10 | 1.11 | 1923 | 1775 | 1890 |
| C84656 | 1.239 | 1.25 | 1.23 | 1924 | 3552 | 3235 |
| K82014 | 1.239 | 1.07 | 1.04 | 1925 | 1442 | 1288 |
| C88027 | 1.239 | 1.18 | 1.19 | 1926 | 2623 | 2792 |
| A88608 | 1.239 | 1.31 | 1.29 | 1927 | 4368 | 4157 |
| F81127 | 1.239 | 1.00 | 1.00 | 1928 | 975 | 973 |
| N81069 | 1.239 | 1.04 | 1.03 | 1929 | 1241 | 1188 |
| C82072 | 1.239 | 1.13 | 1.12 | 1930 | 2046 | 1949 |
| K84040 | 1.239 | 1.00 | 0.99 | 1931 | 979 | 960 |
| M86019 | 1.239 | 1.13 | 1.12 | 1932 | 2077 | 1950 |
| J82152 | 1.239 | 1.09 | 1.12 | 1933 | 1637 | 1929 |
| B82037 | 1.239 | 1.21 | 1.23 | 1934 | 3029 | 3360 |
| P81029 | 1.238 | 1.16 | 1.17 | 1935 | 2368 | 2571 |
| D83002 | 1.238 | 1.18 | 1.18 | 1936 | 2688 | 2610 |
| B84012 | 1.238 | 1.14 | 1.16 | 1937 | 2231 | 2443 |
| L82057 | 1.238 | 1.12 | 1.12 | 1938 | 1965 | 1977 |
| M86016 | 1.238 | 1.14 | 1.13 | 1939 | 2210 | 2056 |
| B81628 | 1.238 | 1.07 | 1.10 | 1940 | 1515 | 1731 |
| C83080 | 1.238 | 1.08 | 1.06 | 1941 | 1552 | 1448 |
| A87022 | 1.238 | 1.10 | 1.11 | 1942 | 1733 | 1849 |
| P81086 | 1.237 | 1.23 | 1.22 | 1943 | 3300 | 3224 |
| G82019 | 1.237 | 1.04 | 1.03 | 1944 | 1256 | 1173 |

| | | | | | | |
|---|---|---|---|---|---|---|
| M86033 | 1.237 | 1.25 | 1.22 | 1945 | 3544 | 3164 |
| M83110 | 1.237 | 1.20 | 1.19 | 1946 | 2918 | 2765 |
| M81025 | 1.237 | 1.14 | 1.11 | 1947 | 2155 | 1858 |
| B81693 | 1.237 | 1.03 | 1.05 | 1948 | 1216 | 1363 |
| K81069 | 1.237 | 0.99 | 0.97 | 1949 | 986 | 837 |
| E85083 | 1.236 | 1.32 | 1.32 | 1950 | 4621 | 4635 |
| M84015 | 1.236 | 1.04 | 1.03 | 1951 | 1290 | 1242 |
| B81634 | 1.236 | 1.16 | 1.20 | 1952 | 2469 | 2995 |
| M91660 | 1.236 | 1.21 | 1.23 | 1953 | 3043 | 3349 |
| P81088 | 1.236 | 1.23 | 1.23 | 1954 | 3352 | 3344 |
| M81075 | 1.236 | 1.09 | 1.07 | 1955 | 1734 | 1529 |
| P81014 | 1.236 | 1.14 | 1.15 | 1956 | 2166 | 2353 |
| M83096 | 1.236 | 1.15 | 1.11 | 1957 | 2364 | 1887 |
| A87003 | 1.236 | 1.27 | 1.28 | 1958 | 3901 | 4004 |
| C81662 | 1.235 | 1.12 | 1.12 | 1959 | 1951 | 1998 |
| E82002 | 1.235 | 1.08 | 1.08 | 1960 | 1631 | 1632 |
| N83050 | 1.235 | 1.23 | 1.23 | 1961 | 3271 | 3409 |
| P81059 | 1.235 | 1.19 | 1.19 | 1962 | 2859 | 2828 |
| D81607 | 1.235 | 1.03 | 1.02 | 1963 | 1205 | 1135 |
| E82050 | 1.235 | 1.07 | 1.05 | 1964 | 1483 | 1391 |
| C82096 | 1.235 | 1.13 | 1.12 | 1965 | 2139 | 2010 |
| F81025 | 1.235 | 1.12 | 1.13 | 1966 | 2042 | 2087 |
| F86010 | 1.235 | 1.03 | 1.02 | 1967 | 1242 | 1127 |
| A82065 | 1.234 | 1.39 | 1.33 | 1968 | 5426 | 4798 |
| B85033 | 1.234 | 1.02 | 1.03 | 1969 | 1160 | 1203 |
| C86005 | 1.234 | 1.39 | 1.40 | 1970 | 5457 | 5591 |
| B81013 | 1.234 | 1.07 | 1.07 | 1971 | 1560 | 1526 |
| P82023 | 1.234 | 1.06 | 1.08 | 1972 | 1438 | 1569 |
| E85018 | 1.234 | 1.00 | 1.00 | 1973 | 1062 | 1018 |
| E81035 | 1.234 | 1.13 | 1.12 | 1974 | 2095 | 1979 |
| L83657 | 1.234 | 1.08 | 1.08 | 1975 | 1639 | 1578 |
| F81725 | 1.234 | 1.36 | 1.31 | 1976 | 5125 | 4479 |
| A83028 | 1.234 | 1.11 | 1.12 | 1977 | 1896 | 1994 |
| C83072 | 1.234 | 1.07 | 1.11 | 1978 | 1537 | 1939 |
| G82681 | 1.233 | 1.01 | 1.02 | 1979 | 1126 | 1154 |
| P81149 | 1.233 | 1.20 | 1.19 | 1980 | 2915 | 2863 |
| A81051 | 1.233 | 1.10 | 1.13 | 1981 | 1836 | 2080 |
| L83147 | 1.233 | 1.04 | 1.06 | 1982 | 1305 | 1462 |
| H82055 | 1.233 | 1.00 | 0.98 | 1983 | 1044 | 901 |
| N81041 | 1.233 | 1.09 | 1.08 | 1984 | 1715 | 1648 |
| G82048 | 1.233 | 1.11 | 1.12 | 1985 | 1973 | 2018 |
| H81656 | 1.232 | 1.01 | 0.98 | 1986 | 1109 | 930 |
| M88022 | 1.232 | 1.14 | 1.15 | 1987 | 2247 | 2389 |
| N83020 | 1.232 | 1.32 | 1.33 | 1988 | 4689 | 4821 |
| F81223 | 1.232 | 1.08 | 1.08 | 1989 | 1618 | 1673 |
| M87009 | 1.232 | 1.10 | 1.10 | 1990 | 1878 | 1832 |
| P88618 | 1.232 | 1.10 | 1.10 | 1991 | 1847 | 1817 |
| N81060 | 1.232 | 1.15 | 1.13 | 1992 | 2371 | 2107 |
| Y02886 | 1.232 | 1.12 | 1.12 | 1993 | 1997 | 2020 |
| G82229 | 1.232 | 1.01 | 1.02 | 1994 | 1134 | 1187 |

| | | | | | | |
|---|---|---|---|---|---|---|
| C82016 | 1.231 | 1.02 | 1.00 | 1995 | 1156 | 1083 |
| A89035 | 1.231 | 1.31 | 1.32 | 1996 | 4567 | 4651 |
| A82034 | 1.231 | 1.18 | 1.14 | 1997 | 2678 | 2305 |
| C81023 | 1.231 | 1.09 | 1.07 | 1998 | 1739 | 1589 |
| L84044 | 1.231 | 1.21 | 1.20 | 1999 | 3163 | 3049 |
| F85707 | 1.230 | 0.87 | 0.87 | 2000 | 482 | 474 |
| J81002 | 1.230 | 1.32 | 1.30 | 2001 | 4624 | 4356 |
| M81049 | 1.230 | 1.09 | 1.10 | 2002 | 1774 | 1844 |
| H81062 | 1.230 | 0.98 | 0.98 | 2003 | 951 | 954 |
| M83103 | 1.230 | 1.10 | 1.06 | 2004 | 1887 | 1496 |
| M83117 | 1.230 | 1.19 | 1.18 | 2005 | 2898 | 2757 |
| M84012 | 1.230 | 1.07 | 1.07 | 2006 | 1603 | 1535 |
| P89011 | 1.230 | 1.30 | 1.32 | 2007 | 4434 | 4673 |
| D81073 | 1.230 | 1.03 | 1.03 | 2008 | 1247 | 1277 |
| C81052 | 1.229 | 1.14 | 1.14 | 2009 | 2241 | 2252 |
| D82031 | 1.229 | 1.09 | 1.08 | 2010 | 1760 | 1701 |
| F81720 | 1.229 | 1.05 | 1.05 | 2011 | 1405 | 1432 |
| A83009 | 1.229 | 1.15 | 1.16 | 2012 | 2420 | 2539 |
| K81065 | 1.229 | 0.91 | 0.92 | 2013 | 612 | 634 |
| H81123 | 1.229 | 1.01 | 1.01 | 2014 | 1122 | 1125 |
| M83700 | 1.229 | 1.05 | 1.09 | 2015 | 1411 | 1782 |
| K83032 | 1.229 | 1.06 | 1.07 | 2016 | 1477 | 1534 |
| P81197 | 1.229 | 1.09 | 1.11 | 2017 | 1730 | 1958 |
| B81602 | 1.229 | 1.26 | 1.25 | 2018 | 3807 | 3716 |
| M87017 | 1.229 | 1.23 | 1.22 | 2019 | 3383 | 3280 |
| P87624 | 1.228 | 1.06 | 1.09 | 2020 | 1479 | 1779 |
| F82006 | 1.228 | 1.06 | 1.05 | 2021 | 1453 | 1455 |
| D83049 | 1.228 | 1.12 | 1.13 | 2022 | 2071 | 2132 |
| H83046 | 1.228 | 1.24 | 1.21 | 2023 | 3567 | 3129 |
| J81013 | 1.228 | 1.12 | 1.12 | 2024 | 2058 | 2065 |
| B81039 | 1.228 | 1.21 | 1.24 | 2025 | 3189 | 3621 |
| G85003 | 1.228 | 0.92 | 0.94 | 2026 | 656 | 713 |
| K83055 | 1.228 | 1.03 | 1.05 | 2027 | 1302 | 1416 |
| L81117 | 1.228 | 1.08 | 1.07 | 2028 | 1680 | 1568 |
| H85056 | 1.228 | 0.91 | 0.90 | 2029 | 635 | 567 |
| M85007 | 1.228 | 1.17 | 1.15 | 2030 | 2603 | 2437 |
| K83051 | 1.228 | 1.12 | 1.12 | 2031 | 2059 | 2040 |
| C83046 | 1.228 | 1.05 | 1.05 | 2032 | 1421 | 1428 |
| B81026 | 1.227 | 1.22 | 1.24 | 2033 | 3350 | 3653 |
| M85045 | 1.227 | 1.16 | 1.14 | 2034 | 2511 | 2344 |
| M83079 | 1.227 | 1.21 | 1.16 | 2035 | 3142 | 2532 |
| C88048 | 1.227 | 1.18 | 1.19 | 2036 | 2802 | 2911 |
| G82185 | 1.227 | 1.03 | 1.04 | 2037 | 1261 | 1358 |
| B81007 | 1.227 | 1.13 | 1.15 | 2038 | 2177 | 2456 |
| B83013 | 1.227 | 1.14 | 1.15 | 2039 | 2271 | 2391 |
| K82051 | 1.227 | 1.01 | 0.99 | 2040 | 1170 | 1033 |
| B86049 | 1.227 | 1.03 | 1.05 | 2041 | 1308 | 1429 |
| B83018 | 1.226 | 1.08 | 1.09 | 2042 | 1691 | 1773 |
| A87004 | 1.226 | 1.22 | 1.23 | 2043 | 3336 | 3533 |
| Y02464 | 1.226 | 1.09 | 1.09 | 2044 | 1763 | 1818 |

| | | | | | | |
|---|---|---|---|---|---|---|
| K82037 | 1.226 | 1.05 | 1.03 | 2045 | 1464 | 1308 |
| B81010 | 1.226 | 1.06 | 1.06 | 2046 | 1529 | 1536 |
| G81033 | 1.226 | 1.12 | 1.16 | 2047 | 2105 | 2528 |
| M92027 | 1.226 | 1.25 | 1.26 | 2048 | 3819 | 3907 |
| J81081 | 1.226 | 1.15 | 1.15 | 2049 | 2489 | 2442 |
| B81697 | 1.226 | 1.15 | 1.18 | 2050 | 2480 | 2747 |
| J81059 | 1.226 | 1.07 | 1.07 | 2051 | 1559 | 1575 |
| A88003 | 1.226 | 1.35 | 1.33 | 2052 | 5088 | 4842 |
| P89609 | 1.226 | 1.25 | 1.26 | 2053 | 3781 | 3982 |
| L83081 | 1.226 | 1.08 | 1.08 | 2054 | 1654 | 1727 |
| P82643 | 1.226 | 0.98 | 0.99 | 2055 | 937 | 1046 |
| E81010 | 1.225 | 1.06 | 1.07 | 2056 | 1517 | 1624 |
| P87003 | 1.225 | 1.16 | 1.18 | 2057 | 2561 | 2847 |
| H85105 | 1.225 | 1.00 | 1.00 | 2058 | 1114 | 1084 |
| F86083 | 1.225 | 1.15 | 1.13 | 2059 | 2497 | 2260 |
| A81067 | 1.224 | 1.20 | 1.19 | 2060 | 3016 | 2928 |
| B84610 | 1.224 | 1.11 | 1.13 | 2061 | 2023 | 2272 |
| B81631 | 1.224 | 1.22 | 1.25 | 2062 | 3404 | 3809 |
| C82051 | 1.224 | 1.08 | 1.08 | 2063 | 1671 | 1738 |
| M81015 | 1.224 | 1.08 | 1.06 | 2064 | 1689 | 1567 |
| K82061 | 1.224 | 1.10 | 1.07 | 2065 | 1873 | 1581 |
| A83045 | 1.224 | 1.23 | 1.23 | 2066 | 3486 | 3564 |
| L84069 | 1.224 | 1.26 | 1.24 | 2067 | 3903 | 3596 |
| J83036 | 1.224 | 1.07 | 1.08 | 2068 | 1580 | 1691 |
| K83006 | 1.223 | 1.14 | 1.14 | 2069 | 2377 | 2345 |
| N82084 | 1.223 | 1.24 | 1.26 | 2070 | 3659 | 3921 |
| M91006 | 1.223 | 1.28 | 1.29 | 2071 | 4238 | 4417 |
| N81089 | 1.223 | 1.18 | 1.16 | 2072 | 2824 | 2609 |
| M83146 | 1.223 | 1.27 | 1.30 | 2073 | 4054 | 4492 |
| J81070 | 1.223 | 1.11 | 1.12 | 2074 | 2061 | 2072 |
| P84065 | 1.223 | 1.09 | 1.11 | 2075 | 1781 | 1968 |
| C88032 | 1.223 | 1.06 | 1.08 | 2076 | 1520 | 1676 |
| F81746 | 1.223 | 0.95 | 0.96 | 2077 | 822 | 844 |
| N85046 | 1.223 | 1.17 | 1.16 | 2078 | 2705 | 2542 |
| M83701 | 1.222 | 1.29 | 1.29 | 2079 | 4418 | 4368 |
| G83034 | 1.222 | 1.09 | 1.08 | 2080 | 1791 | 1756 |
| K82603 | 1.222 | 1.18 | 1.16 | 2081 | 2786 | 2541 |
| F82039 | 1.222 | 1.03 | 1.03 | 2082 | 1280 | 1347 |
| N83027 | 1.222 | 1.44 | 1.45 | 2083 | 6110 | 6170 |
| Y00969 | 1.222 | 1.11 | 1.11 | 2084 | 2020 | 2031 |
| F81068 | 1.222 | 1.08 | 1.08 | 2085 | 1728 | 1706 |
| A89007 | 1.222 | 1.20 | 1.21 | 2086 | 3047 | 3209 |
| K82029 | 1.222 | 0.99 | 0.97 | 2087 | 1031 | 911 |
| Y00090 | 1.222 | 1.15 | 1.13 | 2088 | 2483 | 2222 |
| H84015 | 1.221 | 0.98 | 0.96 | 2089 | 988 | 858 |
| L85016 | 1.221 | 1.27 | 1.26 | 2090 | 4050 | 3958 |
| C82041 | 1.221 | 1.05 | 1.05 | 2091 | 1435 | 1484 |
| P89002 | 1.221 | 1.16 | 1.18 | 2092 | 2556 | 2794 |
| F81045 | 1.221 | 1.00 | 1.01 | 2093 | 1139 | 1210 |
| G81025 | 1.221 | 1.43 | 1.36 | 2094 | 5973 | 5227 |

| | | | | | | |
|---|---|---|---|---|---|---|
| M92022 | 1.221 | 1.19 | 1.20 | 2095 | 3017 | 3107 |
| P84626 | 1.221 | 1.09 | 1.11 | 2096 | 1813 | 2085 |
| C86034 | 1.221 | 1.29 | 1.31 | 2097 | 4425 | 4664 |
| B81074 | 1.221 | 1.38 | 1.41 | 2098 | 5490 | 5815 |
| P84071 | 1.221 | 0.91 | 0.93 | 2099 | 633 | 726 |
| E81052 | 1.221 | 1.09 | 1.09 | 2100 | 1863 | 1798 |
| C82044 | 1.221 | 1.11 | 1.08 | 2101 | 2031 | 1771 |
| F86664 | 1.220 | 0.93 | 0.94 | 2102 | 707 | 769 |
| N83636 | 1.220 | 1.35 | 1.34 | 2103 | 5123 | 5041 |
| A89611 | 1.220 | 0.90 | 0.92 | 2104 | 600 | 681 |
| F81138 | 1.220 | 1.09 | 1.09 | 2105 | 1901 | 1853 |
| K82017 | 1.220 | 1.03 | 1.01 | 2106 | 1304 | 1186 |
| F84014 | 1.220 | 1.12 | 1.11 | 2107 | 2181 | 2016 |
| C88053 | 1.220 | 0.96 | 0.98 | 2108 | 852 | 989 |
| N85034 | 1.220 | 1.24 | 1.22 | 2109 | 3755 | 3485 |
| E82046 | 1.219 | 1.05 | 1.03 | 2110 | 1499 | 1353 |
| M82046 | 1.219 | 1.25 | 1.24 | 2111 | 3813 | 3756 |
| M85078 | 1.219 | 1.16 | 1.15 | 2112 | 2671 | 2562 |
| M82027 | 1.219 | 1.13 | 1.13 | 2113 | 2230 | 2352 |
| G82122 | 1.219 | 0.97 | 0.98 | 2114 | 966 | 1025 |
| J82039 | 1.219 | 1.17 | 1.15 | 2115 | 2706 | 2540 |
| H81097 | 1.219 | 1.04 | 1.04 | 2116 | 1410 | 1393 |
| D83059 | 1.219 | 1.01 | 1.02 | 2117 | 1206 | 1274 |
| K81622 | 1.218 | 0.95 | 0.94 | 2118 | 847 | 751 |
| N83610 | 1.218 | 1.31 | 1.30 | 2119 | 4627 | 4621 |
| P82660 | 1.218 | 1.16 | 1.17 | 2120 | 2599 | 2719 |
| C84694 | 1.218 | 1.08 | 1.10 | 2121 | 1787 | 2003 |
| C88638 | 1.218 | 1.21 | 1.21 | 2122 | 3204 | 3352 |
| N81008 | 1.218 | 1.15 | 1.13 | 2123 | 2533 | 2337 |
| L81049 | 1.218 | 1.08 | 1.07 | 2124 | 1721 | 1656 |
| L83655 | 1.218 | 1.10 | 1.06 | 2125 | 1953 | 1561 |
| D81031 | 1.218 | 1.05 | 1.04 | 2126 | 1503 | 1443 |
| G83037 | 1.218 | 1.04 | 1.03 | 2127 | 1428 | 1375 |
| P85002 | 1.218 | 1.26 | 1.27 | 2128 | 4037 | 4205 |
| C85010 | 1.218 | 1.24 | 1.25 | 2129 | 3770 | 3874 |
| P83605 | 1.217 | 1.12 | 1.13 | 2130 | 2198 | 2255 |
| E81026 | 1.217 | 1.08 | 1.09 | 2131 | 1804 | 1855 |
| C83065 | 1.217 | 1.14 | 1.14 | 2132 | 2408 | 2377 |
| B81027 | 1.217 | 1.27 | 1.31 | 2133 | 4222 | 4763 |
| H81031 | 1.217 | 0.97 | 0.97 | 2134 | 953 | 952 |
| N84625 | 1.217 | 0.92 | 0.92 | 2135 | 677 | 692 |
| C82106 | 1.217 | 1.23 | 1.22 | 2136 | 3638 | 3402 |
| E81056 | 1.217 | 1.10 | 1.09 | 2137 | 1969 | 1840 |
| M91609 | 1.217 | 1.18 | 1.19 | 2138 | 2860 | 3062 |
| G81052 | 1.217 | 1.03 | 1.00 | 2139 | 1358 | 1109 |
| G82097 | 1.217 | 1.08 | 1.09 | 2140 | 1782 | 1875 |
| A81014 | 1.217 | 1.42 | 1.41 | 2141 | 5948 | 5845 |
| E82070 | 1.217 | 0.97 | 0.96 | 2142 | 934 | 868 |
| G82052 | 1.216 | 1.22 | 1.23 | 2143 | 3513 | 3679 |
| C85001 | 1.216 | 1.32 | 1.31 | 2144 | 4854 | 4754 |

| | | | | | | |
|---|---|---|---|---|---|---|
| Y03052 | 1.216 | 1.11 | 1.12 | 2145 | 2068 | 2163 |
| K81073 | 1.216 | 0.99 | 1.00 | 2146 | 1108 | 1113 |
| K81006 | 1.216 | 0.97 | 0.97 | 2147 | 944 | 928 |
| P92016 | 1.216 | 1.13 | 1.14 | 2148 | 2324 | 2466 |
| B81603 | 1.216 | 1.09 | 1.11 | 2149 | 1874 | 2101 |
| N81120 | 1.216 | 1.05 | 1.03 | 2150 | 1485 | 1350 |
| D82013 | 1.215 | 1.00 | 0.99 | 2151 | 1148 | 1091 |
| A86036 | 1.215 | 1.00 | 1.01 | 2152 | 1138 | 1254 |
| B86041 | 1.215 | 1.03 | 1.05 | 2153 | 1339 | 1495 |
| N81062 | 1.215 | 1.10 | 1.08 | 2154 | 1972 | 1827 |
| C81096 | 1.215 | 1.33 | 1.31 | 2155 | 5014 | 4804 |
| P81732 | 1.215 | 1.09 | 1.13 | 2156 | 1949 | 2335 |
| C82626 | 1.215 | 1.14 | 1.14 | 2157 | 2478 | 2432 |
| K83601 | 1.214 | 1.08 | 1.12 | 2158 | 1817 | 2179 |
| G82165 | 1.214 | 1.06 | 1.09 | 2159 | 1620 | 1945 |
| Y00198 | 1.214 | 1.11 | 1.13 | 2160 | 2131 | 2401 |
| H85030 | 1.214 | 1.03 | 1.01 | 2161 | 1347 | 1256 |
| B83629 | 1.214 | 1.20 | 1.21 | 2162 | 3172 | 3361 |
| C81033 | 1.213 | 1.32 | 1.30 | 2163 | 4865 | 4668 |
| F81147 | 1.213 | 1.21 | 1.22 | 2164 | 3395 | 3537 |
| K83618 | 1.213 | 1.05 | 1.06 | 2165 | 1518 | 1628 |
| L83130 | 1.213 | 1.17 | 1.17 | 2166 | 2879 | 2824 |
| C84033 | 1.213 | 1.19 | 1.16 | 2167 | 3064 | 2736 |
| F81069 | 1.213 | 1.06 | 1.06 | 2168 | 1593 | 1659 |
| B85644 | 1.213 | 1.05 | 1.05 | 2169 | 1501 | 1542 |
| C84628 | 1.213 | 0.96 | 0.98 | 2170 | 911 | 1060 |
| N81021 | 1.212 | 1.06 | 1.05 | 2171 | 1645 | 1555 |
| A82023 | 1.212 | 1.47 | 1.49 | 2172 | 6457 | 6595 |
| F85076 | 1.212 | 0.98 | 0.97 | 2173 | 1026 | 1001 |
| J83046 | 1.212 | 1.08 | 1.08 | 2174 | 1881 | 1795 |
| F82020 | 1.212 | 1.13 | 1.12 | 2175 | 2338 | 2265 |
| P84651 | 1.212 | 1.15 | 1.17 | 2176 | 2619 | 2822 |
| D82054 | 1.212 | 1.19 | 1.17 | 2177 | 3115 | 2884 |
| J82063 | 1.211 | 1.01 | 1.00 | 2178 | 1236 | 1175 |
| B81685 | 1.211 | 1.17 | 1.21 | 2179 | 2863 | 3443 |
| N85051 | 1.211 | 1.20 | 1.18 | 2180 | 3202 | 3023 |
| G83015 | 1.211 | 1.17 | 1.15 | 2181 | 2853 | 2647 |
| L84020 | 1.211 | 1.03 | 1.02 | 2182 | 1422 | 1351 |
| L83624 | 1.211 | 1.31 | 1.32 | 2183 | 4839 | 4870 |
| A82006 | 1.211 | 1.17 | 1.15 | 2184 | 2905 | 2576 |
| C84103 | 1.211 | 1.06 | 1.07 | 2185 | 1614 | 1792 |
| C81070 | 1.211 | 1.09 | 1.10 | 2186 | 1966 | 2051 |
| B83070 | 1.211 | 1.24 | 1.26 | 2187 | 3864 | 4133 |
| J81044 | 1.211 | 1.11 | 1.10 | 2188 | 2120 | 2084 |
| D82032 | 1.211 | 1.13 | 1.12 | 2189 | 2398 | 2282 |
| P81154 | 1.210 | 1.21 | 1.18 | 2190 | 3422 | 2978 |
| D82039 | 1.210 | 1.20 | 1.19 | 2191 | 3261 | 3194 |
| P87654 | 1.210 | 1.19 | 1.21 | 2192 | 3123 | 3401 |
| Y02893 | 1.210 | 1.18 | 1.17 | 2193 | 2978 | 2799 |
| C84648 | 1.210 | 1.06 | 1.06 | 2194 | 1668 | 1621 |

| | | | | | | |
|---|---|---|---|---|---|---|
| F82014 | 1.210 | 1.13 | 1.12 | 2195 | 2390 | 2317 |
| C83059 | 1.210 | 1.14 | 1.13 | 2196 | 2503 | 2362 |
| N83604 | 1.210 | 1.33 | 1.32 | 2197 | 5035 | 4948 |
| B86672 | 1.210 | 1.18 | 1.19 | 2198 | 2967 | 3166 |
| E86028 | 1.209 | 0.99 | 0.99 | 2199 | 1150 | 1131 |
| B81062 | 1.209 | 1.18 | 1.18 | 2200 | 3052 | 3055 |
| P82037 | 1.209 | 1.17 | 1.18 | 2201 | 2862 | 2968 |
| K81633 | 1.209 | 0.96 | 0.96 | 2202 | 919 | 931 |
| M88009 | 1.209 | 1.16 | 1.17 | 2203 | 2764 | 2898 |
| L83076 | 1.209 | 1.13 | 1.15 | 2204 | 2458 | 2679 |
| A86028 | 1.209 | 1.14 | 1.15 | 2205 | 2485 | 2686 |
| A83042 | 1.209 | 1.26 | 1.27 | 2206 | 4209 | 4276 |
| N81016 | 1.209 | 1.04 | 1.03 | 2207 | 1497 | 1396 |
| A88614 | 1.209 | 1.21 | 1.20 | 2208 | 3373 | 3239 |
| M83082 | 1.208 | 1.25 | 1.29 | 2209 | 4055 | 4516 |
| M89009 | 1.208 | 1.21 | 1.19 | 2210 | 3400 | 3172 |
| C83023 | 1.208 | 1.16 | 1.15 | 2211 | 2747 | 2656 |
| A86006 | 1.208 | 1.12 | 1.14 | 2212 | 2253 | 2478 |
| L83608 | 1.208 | 1.08 | 1.06 | 2213 | 1834 | 1653 |
| C84077 | 1.208 | 1.13 | 1.15 | 2214 | 2393 | 2632 |
| C86018 | 1.208 | 1.24 | 1.26 | 2215 | 3928 | 4171 |
| C88022 | 1.208 | 1.25 | 1.26 | 2216 | 3999 | 4238 |
| G81097 | 1.208 | 1.06 | 1.04 | 2217 | 1708 | 1493 |
| P81100 | 1.208 | 1.21 | 1.19 | 2218 | 3427 | 3113 |
| A82047 | 1.207 | 1.20 | 1.20 | 2219 | 3225 | 3316 |
| N81064 | 1.207 | 1.20 | 1.21 | 2220 | 3278 | 3412 |
| P88017 | 1.207 | 1.23 | 1.21 | 2221 | 3706 | 3529 |
| B83039 | 1.207 | 1.24 | 1.25 | 2222 | 3947 | 4024 |
| P86015 | 1.207 | 1.16 | 1.17 | 2223 | 2722 | 2904 |
| N84618 | 1.207 | 1.09 | 1.09 | 2224 | 2005 | 2014 |
| M91029 | 1.207 | 1.33 | 1.34 | 2225 | 5092 | 5150 |
| H85029 | 1.207 | 1.05 | 1.04 | 2226 | 1611 | 1470 |
| H82061 | 1.206 | 1.04 | 1.04 | 2227 | 1519 | 1525 |
| P89016 | 1.206 | 1.34 | 1.36 | 2228 | 5235 | 5425 |
| P88604 | 1.206 | 1.32 | 1.30 | 2229 | 4997 | 4739 |
| E81038 | 1.206 | 1.13 | 1.12 | 2230 | 2415 | 2351 |
| A83038 | 1.206 | 1.07 | 1.08 | 2231 | 1785 | 1900 |
| P92019 | 1.206 | 1.19 | 1.20 | 2232 | 3211 | 3327 |
| D82068 | 1.206 | 1.22 | 1.22 | 2233 | 3626 | 3618 |
| N82103 | 1.206 | 1.20 | 1.22 | 2234 | 3374 | 3566 |
| L81034 | 1.205 | 1.15 | 1.13 | 2235 | 2723 | 2488 |
| M91015 | 1.205 | 1.15 | 1.17 | 2236 | 2718 | 2953 |
| M83637 | 1.205 | 1.09 | 1.12 | 2237 | 1963 | 2366 |
| J82215 | 1.205 | 1.19 | 1.23 | 2238 | 3214 | 3722 |
| M85011 | 1.205 | 1.16 | 1.14 | 2239 | 2773 | 2574 |
| E81073 | 1.205 | 0.99 | 0.99 | 2240 | 1141 | 1165 |
| M87013 | 1.205 | 1.10 | 1.11 | 2241 | 2080 | 2223 |
| F85015 | 1.205 | 0.99 | 0.98 | 2242 | 1118 | 1088 |
| P91004 | 1.205 | 0.96 | 0.97 | 2243 | 978 | 1029 |
| C81110 | 1.205 | 0.99 | 0.97 | 2244 | 1131 | 1008 |

| | | | | | | |
|---|---|---|---|---|---|---|
| M88031 | 1.205 | 1.16 | 1.17 | 2245 | 2856 | 2976 |
| C82027 | 1.204 | 1.20 | 1.19 | 2246 | 3362 | 3210 |
| H85055 | 1.204 | 1.02 | 1.00 | 2247 | 1365 | 1259 |
| P89006 | 1.204 | 1.09 | 1.11 | 2248 | 1971 | 2198 |
| G83026 | 1.204 | 1.05 | 1.04 | 2249 | 1650 | 1516 |
| A89042 | 1.204 | 1.27 | 1.27 | 2250 | 4305 | 4388 |
| A81041 | 1.204 | 1.23 | 1.26 | 2251 | 3777 | 4199 |
| M86017 | 1.204 | 1.10 | 1.09 | 2252 | 2143 | 2034 |
| N81036 | 1.204 | 1.16 | 1.15 | 2253 | 2808 | 2676 |
| P91623 | 1.204 | 1.03 | 1.04 | 2254 | 1466 | 1517 |
| H82077 | 1.204 | 1.05 | 1.05 | 2255 | 1666 | 1660 |
| G82107 | 1.204 | 1.22 | 1.23 | 2256 | 3717 | 3879 |
| C86021 | 1.204 | 1.29 | 1.31 | 2257 | 4653 | 4829 |
| H84050 | 1.204 | 1.09 | 1.07 | 2258 | 1999 | 1783 |
| C84064 | 1.204 | 1.14 | 1.16 | 2259 | 2555 | 2858 |
| N81071 | 1.203 | 1.13 | 1.11 | 2260 | 2417 | 2259 |
| B81065 | 1.203 | 1.16 | 1.18 | 2261 | 2837 | 3145 |
| M82018 | 1.203 | 1.08 | 1.09 | 2262 | 1929 | 2011 |
| K81051 | 1.203 | 1.01 | 0.98 | 2263 | 1273 | 1108 |
| A83030 | 1.203 | 1.15 | 1.16 | 2264 | 2742 | 2867 |
| F83053 | 1.203 | 0.98 | 1.00 | 2265 | 1117 | 1197 |
| C81638 | 1.203 | 1.19 | 1.18 | 2266 | 3158 | 3138 |
| C83641 | 1.203 | 1.24 | 1.23 | 2267 | 3902 | 3805 |
| M87026 | 1.203 | 1.15 | 1.15 | 2268 | 2761 | 2740 |
| C82066 | 1.203 | 1.08 | 1.08 | 2269 | 1964 | 1911 |
| A83012 | 1.203 | 1.34 | 1.34 | 2270 | 5279 | 5224 |
| G82735 | 1.202 | 0.98 | 0.97 | 2271 | 1093 | 1043 |
| M81082 | 1.202 | 1.07 | 1.05 | 2272 | 1879 | 1658 |
| P81066 | 1.202 | 1.30 | 1.33 | 2273 | 4781 | 5145 |
| K81023 | 1.202 | 1.04 | 1.03 | 2274 | 1534 | 1476 |
| G82164 | 1.202 | 1.04 | 1.05 | 2275 | 1553 | 1601 |
| K81056 | 1.202 | 1.01 | 1.01 | 2276 | 1306 | 1336 |
| B85019 | 1.201 | 1.13 | 1.13 | 2277 | 2481 | 2544 |
| N85057 | 1.201 | 1.17 | 1.16 | 2278 | 3022 | 2916 |
| K83030 | 1.201 | 1.14 | 1.13 | 2279 | 2568 | 2507 |
| M81605 | 1.201 | 1.06 | 1.04 | 2280 | 1751 | 1518 |
| Y02322 | 1.201 | 1.20 | 1.20 | 2281 | 3408 | 3467 |
| L85013 | 1.201 | 1.17 | 1.16 | 2282 | 3034 | 2913 |
| B86004 | 1.201 | 1.08 | 1.11 | 2283 | 1998 | 2219 |
| G82198 | 1.200 | 1.09 | 1.12 | 2284 | 2064 | 2429 |
| L85616 | 1.200 | 1.10 | 1.06 | 2285 | 2157 | 1794 |
| M82028 | 1.200 | 1.09 | 1.11 | 2286 | 2049 | 2242 |
| C82112 | 1.200 | 1.13 | 1.14 | 2287 | 2557 | 2590 |
| P81128 | 1.200 | 1.18 | 1.14 | 2288 | 3092 | 2619 |
| A82050 | 1.200 | 1.21 | 1.21 | 2289 | 3598 | 3611 |
| E82117 | 1.200 | 0.99 | 1.00 | 2290 | 1189 | 1248 |
| P82005 | 1.200 | 1.15 | 1.17 | 2291 | 2790 | 2966 |
| F81739 | 1.200 | 1.33 | 1.31 | 2292 | 5139 | 4905 |
| G81676 | 1.200 | 1.07 | 1.09 | 2293 | 1903 | 2013 |
| D81023 | 1.200 | 1.01 | 1.02 | 2294 | 1369 | 1421 |

| | | | | | | |
|---|---|---|---|---|---|---|
| M85749 | 1.200 | 1.15 | 1.15 | 2295 | 2766 | 2712 |
| C81012 | 1.200 | 1.15 | 1.14 | 2296 | 2734 | 2595 |
| K82032 | 1.199 | 1.01 | 1.01 | 2297 | 1351 | 1316 |
| A83036 | 1.199 | 1.12 | 1.14 | 2298 | 2386 | 2612 |
| N85059 | 1.199 | 1.06 | 1.06 | 2299 | 1784 | 1770 |
| A82008 | 1.199 | 1.20 | 1.23 | 2300 | 3399 | 3856 |
| P81699 | 1.199 | 1.11 | 1.16 | 2301 | 2307 | 2862 |
| A87009 | 1.199 | 1.09 | 1.11 | 2302 | 2085 | 2248 |
| P83611 | 1.199 | 1.11 | 1.11 | 2303 | 2274 | 2327 |
| M85776 | 1.199 | 1.10 | 1.09 | 2304 | 2201 | 2027 |
| D82043 | 1.199 | 1.16 | 1.16 | 2305 | 2865 | 2885 |
| N84009 | 1.199 | 1.15 | 1.15 | 2306 | 2743 | 2716 |
| C82046 | 1.199 | 1.09 | 1.09 | 2307 | 2123 | 2044 |
| E82132 | 1.199 | 1.09 | 1.09 | 2308 | 2117 | 2007 |
| H83033 | 1.199 | 1.06 | 1.05 | 2309 | 1805 | 1710 |
| M83692 | 1.199 | 1.20 | 1.19 | 2310 | 3415 | 3301 |
| K84058 | 1.199 | 0.98 | 0.98 | 2311 | 1121 | 1105 |
| C86038 | 1.199 | 1.16 | 1.18 | 2312 | 2858 | 3226 |
| P81018 | 1.199 | 1.19 | 1.18 | 2313 | 3254 | 3156 |
| M83025 | 1.199 | 1.08 | 1.09 | 2314 | 1962 | 2082 |
| K82021 | 1.199 | 1.06 | 1.04 | 2315 | 1794 | 1617 |
| M85081 | 1.198 | 1.20 | 1.18 | 2316 | 3416 | 3192 |
| G81077 | 1.198 | 1.08 | 1.04 | 2317 | 2006 | 1608 |
| M87015 | 1.198 | 1.28 | 1.26 | 2318 | 4574 | 4353 |
| B82066 | 1.198 | 1.09 | 1.09 | 2319 | 2091 | 2088 |
| B84013 | 1.198 | 1.13 | 1.15 | 2320 | 2587 | 2812 |
| B82062 | 1.198 | 1.16 | 1.17 | 2321 | 2877 | 3086 |
| C88025 | 1.198 | 1.25 | 1.26 | 2322 | 4181 | 4339 |
| N85633 | 1.197 | 1.04 | 1.04 | 2323 | 1633 | 1576 |
| P81701 | 1.197 | 1.08 | 1.06 | 2324 | 2012 | 1764 |
| G81048 | 1.197 | 1.07 | 1.11 | 2325 | 1921 | 2277 |
| B86056 | 1.197 | 0.96 | 0.98 | 2326 | 1013 | 1145 |
| C83061 | 1.197 | 1.20 | 1.22 | 2327 | 3461 | 3771 |
| M88032 | 1.197 | 1.19 | 1.19 | 2328 | 3240 | 3350 |
| L82026 | 1.197 | 1.25 | 1.24 | 2329 | 4170 | 4081 |
| P83012 | 1.197 | 1.11 | 1.12 | 2330 | 2328 | 2411 |
| C83653 | 1.197 | 1.04 | 1.03 | 2331 | 1591 | 1505 |
| F84015 | 1.197 | 1.02 | 1.03 | 2332 | 1441 | 1543 |
| M83632 | 1.197 | 1.21 | 1.16 | 2333 | 3563 | 2886 |
| D83014 | 1.196 | 1.15 | 1.12 | 2334 | 2801 | 2418 |
| H82641 | 1.196 | 1.30 | 1.29 | 2335 | 4882 | 4690 |
| F81651 | 1.196 | 1.06 | 1.07 | 2336 | 1802 | 1878 |
| H81021 | 1.196 | 0.94 | 0.94 | 2337 | 860 | 861 |
| D82036 | 1.196 | 1.10 | 1.10 | 2338 | 2191 | 2150 |
| A88006 | 1.196 | 1.45 | 1.42 | 2339 | 6405 | 6109 |
| B86075 | 1.196 | 1.07 | 1.09 | 2340 | 1943 | 2108 |
| C85008 | 1.196 | 1.20 | 1.22 | 2341 | 3491 | 3717 |
| D81010 | 1.196 | 1.12 | 1.11 | 2342 | 2383 | 2304 |
| N83620 | 1.196 | 1.23 | 1.23 | 2343 | 3889 | 3877 |
| P88011 | 1.195 | 1.18 | 1.16 | 2344 | 3110 | 2958 |

| | | | | | | |
|---|---|---|---|---|---|---|
| E81636 | 1.195 | 1.19 | 1.18 | 2345 | 3323 | 3149 |
| F81118 | 1.195 | 1.06 | 1.03 | 2346 | 1778 | 1483 |
| C84066 | 1.195 | 1.20 | 1.17 | 2347 | 3406 | 3084 |
| B86023 | 1.195 | 1.08 | 1.10 | 2348 | 1984 | 2193 |
| C85009 | 1.195 | 1.09 | 1.10 | 2349 | 2113 | 2190 |
| L81018 | 1.195 | 1.08 | 1.08 | 2350 | 2039 | 1963 |
| K82013 | 1.195 | 1.13 | 1.12 | 2351 | 2610 | 2490 |
| G83017 | 1.195 | 1.03 | 1.02 | 2352 | 1498 | 1430 |
| L82021 | 1.194 | 1.21 | 1.22 | 2353 | 3655 | 3760 |
| B82611 | 1.194 | 1.20 | 1.21 | 2354 | 3539 | 3718 |
| H82035 | 1.194 | 0.98 | 0.98 | 2355 | 1168 | 1163 |
| F82019 | 1.194 | 1.15 | 1.16 | 2356 | 2811 | 2935 |
| B85061 | 1.194 | 1.09 | 1.09 | 2357 | 2103 | 2166 |
| E86033 | 1.194 | 1.06 | 1.05 | 2358 | 1800 | 1733 |
| C87010 | 1.194 | 1.25 | 1.24 | 2359 | 4172 | 4130 |
| P89015 | 1.194 | 1.14 | 1.16 | 2360 | 2656 | 2933 |
| J83027 | 1.194 | 1.21 | 1.21 | 2361 | 3590 | 3681 |
| F81047 | 1.194 | 1.16 | 1.18 | 2362 | 2943 | 3237 |
| P89012 | 1.193 | 1.24 | 1.26 | 2363 | 4066 | 4314 |
| N83009 | 1.193 | 1.27 | 1.27 | 2364 | 4451 | 4454 |
| M91612 | 1.193 | 1.30 | 1.30 | 2365 | 4866 | 4921 |
| P86006 | 1.193 | 1.18 | 1.20 | 2366 | 3236 | 3597 |
| A82015 | 1.193 | 1.26 | 1.27 | 2367 | 4323 | 4563 |
| F82625 | 1.193 | 0.99 | 1.01 | 2368 | 1266 | 1370 |
| L83021 | 1.193 | 1.10 | 1.12 | 2369 | 2258 | 2520 |
| M87601 | 1.193 | 1.17 | 1.16 | 2370 | 3061 | 2922 |
| P84637 | 1.193 | 1.23 | 1.24 | 2371 | 3970 | 4125 |
| K81636 | 1.192 | 1.01 | 1.01 | 2372 | 1359 | 1392 |
| F81087 | 1.192 | 1.17 | 1.17 | 2373 | 3104 | 3056 |
| C86015 | 1.192 | 1.27 | 1.28 | 2374 | 4437 | 4695 |
| K81025 | 1.192 | 0.99 | 0.96 | 2375 | 1202 | 1070 |
| L81042 | 1.192 | 1.02 | 1.02 | 2376 | 1476 | 1452 |
| N81073 | 1.192 | 1.12 | 1.10 | 2377 | 2442 | 2285 |
| C88047 | 1.192 | 1.31 | 1.32 | 2378 | 5071 | 5165 |
| B86062 | 1.192 | 1.31 | 1.31 | 2379 | 5021 | 5000 |
| A87615 | 1.192 | 1.15 | 1.17 | 2380 | 2914 | 3066 |
| D83062 | 1.192 | 1.29 | 1.29 | 2381 | 4754 | 4743 |
| Y01794 | 1.192 | 1.13 | 1.13 | 2382 | 2560 | 2638 |
| A83031 | 1.191 | 1.16 | 1.16 | 2383 | 2986 | 3042 |
| P81061 | 1.191 | 1.30 | 1.32 | 2384 | 4977 | 5187 |
| P82029 | 1.191 | 1.36 | 1.35 | 2385 | 5625 | 5527 |
| L83646 | 1.191 | 1.19 | 1.20 | 2386 | 3375 | 3524 |
| B83058 | 1.191 | 1.09 | 1.11 | 2387 | 2194 | 2368 |
| B86001 | 1.191 | 1.15 | 1.16 | 2388 | 2917 | 3043 |
| J84016 | 1.191 | 1.35 | 1.35 | 2389 | 5534 | 5542 |
| Y02274 | 1.191 | 1.07 | 1.07 | 2390 | 1900 | 1966 |
| B81011 | 1.191 | 1.17 | 1.22 | 2391 | 3097 | 3799 |
| E86017 | 1.191 | 1.00 | 0.99 | 2392 | 1320 | 1279 |
| C86626 | 1.191 | 1.05 | 1.08 | 2393 | 1764 | 2063 |
| Y04333 | 1.191 | 0.94 | 0.94 | 2394 | 913 | 946 |

| | | | | | | |
|---|---|---|---|---|---|---|
| M83718 | 1.191 | 1.10 | 1.06 | 2395 | 2255 | 1819 |
| G82137 | 1.191 | 1.01 | 1.01 | 2396 | 1378 | 1395 |
| A87011 | 1.190 | 1.09 | 1.11 | 2397 | 2153 | 2360 |
| A82608 | 1.190 | 1.08 | 1.05 | 2398 | 2069 | 1775 |
| A81064 | 1.190 | 1.05 | 1.07 | 2399 | 1714 | 1905 |
| P82640 | 1.190 | 1.11 | 1.13 | 2400 | 2352 | 2591 |
| P85010 | 1.190 | 1.19 | 1.21 | 2401 | 3413 | 3678 |
| P88005 | 1.190 | 1.16 | 1.15 | 2402 | 2973 | 2905 |
| B83009 | 1.190 | 1.24 | 1.24 | 2403 | 4194 | 4213 |
| H82050 | 1.190 | 1.09 | 1.10 | 2404 | 2174 | 2303 |
| G84004 | 1.190 | 1.01 | 1.00 | 2405 | 1383 | 1355 |
| L83020 | 1.190 | 1.16 | 1.13 | 2406 | 3031 | 2658 |
| M81068 | 1.190 | 1.19 | 1.17 | 2407 | 3386 | 3122 |
| Y02583 | 1.190 | 0.80 | 0.81 | 2408 | 400 | 424 |
| P81095 | 1.190 | 1.20 | 1.23 | 2409 | 3620 | 3962 |
| K83008 | 1.189 | 1.10 | 1.11 | 2410 | 2297 | 2455 |
| B86002 | 1.189 | 1.08 | 1.09 | 2411 | 2048 | 2158 |
| H81043 | 1.189 | 1.01 | 1.01 | 2412 | 1396 | 1413 |
| G82186 | 1.189 | 1.09 | 1.09 | 2413 | 2178 | 2110 |
| M83018 | 1.189 | 1.10 | 1.06 | 2414 | 2305 | 1902 |
| N81050 | 1.189 | 1.08 | 1.06 | 2415 | 2035 | 1810 |
| E86026 | 1.189 | 1.01 | 1.00 | 2416 | 1406 | 1339 |
| E85023 | 1.189 | 1.30 | 1.29 | 2417 | 4884 | 4863 |
| P81020 | 1.189 | 1.28 | 1.29 | 2418 | 4650 | 4800 |
| E82018 | 1.189 | 1.04 | 1.04 | 2419 | 1626 | 1661 |
| A88008 | 1.189 | 1.35 | 1.33 | 2420 | 5564 | 5304 |
| G82117 | 1.189 | 1.10 | 1.12 | 2421 | 2322 | 2489 |
| L83089 | 1.189 | 1.09 | 1.11 | 2422 | 2156 | 2420 |
| F81030 | 1.189 | 1.10 | 1.09 | 2423 | 2251 | 2207 |
| C82093 | 1.188 | 0.99 | 0.98 | 2424 | 1255 | 1169 |
| H85028 | 1.188 | 0.88 | 0.86 | 2425 | 641 | 593 |
| P87020 | 1.188 | 1.12 | 1.15 | 2426 | 2554 | 2871 |
| E85121 | 1.188 | 1.06 | 1.07 | 2427 | 1839 | 1918 |
| E86020 | 1.188 | 0.98 | 0.97 | 2428 | 1190 | 1147 |
| J82213 | 1.188 | 1.20 | 1.20 | 2429 | 3637 | 3624 |
| K81087 | 1.188 | 0.98 | 0.97 | 2430 | 1169 | 1134 |
| E81061 | 1.188 | 1.02 | 1.02 | 2431 | 1532 | 1520 |
| F85663 | 1.187 | 1.05 | 1.04 | 2432 | 1757 | 1719 |
| N81645 | 1.187 | 1.14 | 1.12 | 2433 | 2828 | 2584 |
| B81658 | 1.187 | 1.10 | 1.09 | 2434 | 2250 | 2216 |
| K83042 | 1.187 | 1.10 | 1.12 | 2435 | 2349 | 2511 |
| M84018 | 1.187 | 1.07 | 1.07 | 2436 | 1961 | 1956 |
| L83080 | 1.187 | 1.26 | 1.27 | 2437 | 4505 | 4648 |
| A81608 | 1.187 | 1.20 | 1.20 | 2438 | 3650 | 3619 |
| F86057 | 1.187 | 1.07 | 1.04 | 2439 | 1939 | 1716 |
| B81090 | 1.187 | 1.26 | 1.27 | 2440 | 4407 | 4606 |
| H81065 | 1.186 | 1.04 | 1.02 | 2441 | 1690 | 1522 |
| H85112 | 1.186 | 0.79 | 0.78 | 2442 | 390 | 365 |
| F82008 | 1.186 | 1.08 | 1.08 | 2443 | 2112 | 2068 |
| B84002 | 1.186 | 1.12 | 1.15 | 2444 | 2606 | 2881 |

| | | | | | | |
|---|---|---|---|---|---|---|
| B86061 | 1.186 | 1.08 | 1.10 | 2445 | 2073 | 2264 |
| D83045 | 1.186 | 1.19 | 1.19 | 2446 | 3479 | 3541 |
| N82621 | 1.186 | 1.28 | 1.28 | 2447 | 4705 | 4733 |
| C86019 | 1.186 | 1.08 | 1.10 | 2448 | 2060 | 2302 |
| N83012 | 1.186 | 1.14 | 1.16 | 2449 | 2840 | 3044 |
| L84015 | 1.186 | 1.04 | 1.05 | 2450 | 1672 | 1784 |
| E81003 | 1.186 | 1.09 | 1.08 | 2451 | 2180 | 2121 |
| F86026 | 1.186 | 1.03 | 1.04 | 2452 | 1564 | 1729 |
| H85031 | 1.185 | 0.98 | 0.97 | 2453 | 1222 | 1138 |
| Y00445 | 1.185 | 1.06 | 1.08 | 2454 | 1897 | 2146 |
| G82087 | 1.185 | 1.10 | 1.09 | 2455 | 2288 | 2205 |
| C83649 | 1.185 | 1.10 | 1.09 | 2456 | 2331 | 2247 |
| G82063 | 1.185 | 1.15 | 1.16 | 2457 | 2985 | 3115 |
| C82012 | 1.185 | 1.18 | 1.16 | 2458 | 3252 | 3081 |
| C84127 | 1.185 | 1.30 | 1.33 | 2459 | 5046 | 5339 |
| C87606 | 1.185 | 1.23 | 1.23 | 2460 | 4101 | 4090 |
| F81200 | 1.185 | 1.21 | 1.22 | 2461 | 3804 | 3889 |
| M88635 | 1.185 | 1.14 | 1.15 | 2462 | 2776 | 3017 |
| A89016 | 1.184 | 1.18 | 1.19 | 2463 | 3348 | 3550 |
| N83010 | 1.184 | 1.26 | 1.27 | 2464 | 4534 | 4681 |
| Y00297 | 1.184 | 1.19 | 1.19 | 2465 | 3557 | 3514 |
| P86021 | 1.184 | 1.14 | 1.16 | 2466 | 2867 | 3096 |
| J81012 | 1.184 | 1.04 | 1.04 | 2467 | 1743 | 1739 |
| P84673 | 1.184 | 1.04 | 1.05 | 2468 | 1696 | 1837 |
| P83006 | 1.184 | 1.04 | 1.04 | 2469 | 1720 | 1749 |
| H83008 | 1.184 | 1.08 | 1.07 | 2470 | 2136 | 2023 |
| B82023 | 1.184 | 1.07 | 1.06 | 2471 | 2029 | 1944 |
| N85620 | 1.184 | 1.24 | 1.23 | 2472 | 4237 | 4070 |
| H85024 | 1.183 | 0.99 | 0.97 | 2473 | 1279 | 1157 |
| M92607 | 1.183 | 1.18 | 1.19 | 2474 | 3290 | 3546 |
| J82059 | 1.183 | 0.99 | 0.99 | 2475 | 1276 | 1276 |
| N85028 | 1.183 | 1.23 | 1.21 | 2476 | 4072 | 3839 |
| D82020 | 1.183 | 1.06 | 1.05 | 2477 | 1925 | 1860 |
| B86667 | 1.183 | 1.06 | 1.08 | 2478 | 1952 | 2105 |
| F81132 | 1.183 | 1.07 | 1.06 | 2479 | 2024 | 1937 |
| N83017 | 1.183 | 1.14 | 1.16 | 2480 | 2886 | 3106 |
| J83018 | 1.183 | 1.11 | 1.09 | 2481 | 2411 | 2276 |
| C83611 | 1.183 | 1.03 | 1.03 | 2482 | 1632 | 1625 |
| B84017 | 1.183 | 1.19 | 1.20 | 2483 | 3550 | 3663 |
| K83041 | 1.183 | 0.98 | 0.99 | 2484 | 1211 | 1335 |
| B81043 | 1.183 | 1.12 | 1.14 | 2485 | 2542 | 2854 |
| B81092 | 1.183 | 1.27 | 1.26 | 2486 | 4564 | 4548 |
| C84026 | 1.183 | 1.18 | 1.15 | 2487 | 3280 | 2960 |
| E85035 | 1.183 | 1.10 | 1.08 | 2488 | 2298 | 2131 |
| N81108 | 1.182 | 0.99 | 0.99 | 2489 | 1332 | 1325 |
| Y01291 | 1.182 | 0.99 | 1.01 | 2490 | 1309 | 1440 |
| J81018 | 1.182 | 1.08 | 1.08 | 2491 | 2151 | 2127 |
| H81099 | 1.182 | 0.91 | 0.90 | 2492 | 793 | 739 |
| J81609 | 1.182 | 1.20 | 1.17 | 2493 | 3616 | 3262 |
| J84019 | 1.182 | 1.17 | 1.17 | 2494 | 3190 | 3308 |

| | | | | | | |
|---|---|---|---|---|---|---|
| B81022 | 1.182 | 1.21 | 1.23 | 2495 | 3853 | 4162 |
| F81729 | 1.182 | 1.14 | 1.14 | 2496 | 2899 | 2835 |
| M82604 | 1.182 | 1.05 | 1.05 | 2497 | 1872 | 1836 |
| J83013 | 1.182 | 1.09 | 1.07 | 2498 | 2224 | 2055 |
| M83007 | 1.181 | 1.12 | 1.13 | 2499 | 2654 | 2768 |
| M91004 | 1.181 | 1.30 | 1.31 | 2500 | 5098 | 5183 |
| N84626 | 1.181 | 1.05 | 1.06 | 2501 | 1890 | 1888 |
| N82095 | 1.181 | 1.32 | 1.32 | 2502 | 5314 | 5275 |
| G81012 | 1.181 | 1.20 | 1.17 | 2503 | 3633 | 3242 |
| L81021 | 1.181 | 1.12 | 1.09 | 2504 | 2600 | 2325 |
| B83659 | 1.181 | 1.34 | 1.34 | 2505 | 5492 | 5546 |
| L84046 | 1.180 | 1.18 | 1.18 | 2506 | 3447 | 3358 |
| A81631 | 1.180 | 1.20 | 1.23 | 2507 | 3644 | 4085 |
| N84605 | 1.180 | 1.23 | 1.22 | 2508 | 4189 | 3979 |
| M85679 | 1.180 | 1.10 | 1.10 | 2509 | 2384 | 2343 |
| M84028 | 1.180 | 1.08 | 1.08 | 2510 | 2183 | 2128 |
| L83128 | 1.180 | 1.12 | 1.14 | 2511 | 2591 | 2802 |
| F82025 | 1.180 | 1.16 | 1.15 | 2512 | 3076 | 3085 |
| L81124 | 1.180 | 1.05 | 1.03 | 2513 | 1862 | 1649 |
| P81081 | 1.180 | 1.23 | 1.27 | 2514 | 4174 | 4707 |
| D81086 | 1.179 | 1.03 | 1.00 | 2515 | 1657 | 1373 |
| P86620 | 1.179 | 1.43 | 1.41 | 2516 | 6389 | 6235 |
| A89026 | 1.179 | 1.14 | 1.15 | 2517 | 2938 | 3071 |
| G83647 | 1.179 | 1.03 | 1.02 | 2518 | 1651 | 1582 |
| M82601 | 1.179 | 1.22 | 1.22 | 2519 | 3983 | 3987 |
| K83052 | 1.179 | 1.04 | 1.01 | 2520 | 1767 | 1480 |
| G83049 | 1.179 | 1.13 | 1.11 | 2521 | 2728 | 2579 |
| G83028 | 1.179 | 1.05 | 1.04 | 2522 | 1840 | 1714 |
| M92024 | 1.179 | 1.07 | 1.08 | 2523 | 2065 | 2206 |
| G85099 | 1.179 | 1.17 | 1.19 | 2524 | 3309 | 3520 |
| B83056 | 1.179 | 1.26 | 1.27 | 2525 | 4554 | 4639 |
| J81058 | 1.178 | 1.06 | 1.05 | 2526 | 1982 | 1884 |
| P83010 | 1.178 | 1.21 | 1.20 | 2527 | 3865 | 3807 |
| D81047 | 1.178 | 1.03 | 1.02 | 2528 | 1630 | 1532 |
| J84008 | 1.178 | 1.15 | 1.14 | 2529 | 2961 | 2927 |
| M86021 | 1.178 | 1.05 | 1.04 | 2530 | 1844 | 1789 |
| D81044 | 1.178 | 1.09 | 1.05 | 2531 | 2242 | 1866 |
| E81631 | 1.178 | 1.02 | 1.03 | 2532 | 1608 | 1695 |
| P81083 | 1.178 | 1.21 | 1.19 | 2533 | 3822 | 3666 |
| L83070 | 1.178 | 1.08 | 1.07 | 2534 | 2186 | 2075 |
| K83018 | 1.178 | 1.01 | 0.98 | 2535 | 1461 | 1268 |
| B86071 | 1.178 | 1.10 | 1.12 | 2536 | 2476 | 2685 |
| G85104 | 1.178 | 1.16 | 1.16 | 2537 | 3159 | 3222 |
| N85018 | 1.178 | 1.24 | 1.22 | 2538 | 4279 | 4037 |
| K81078 | 1.178 | 0.99 | 0.99 | 2539 | 1333 | 1376 |
| L81037 | 1.177 | 1.13 | 1.14 | 2540 | 2827 | 2888 |
| M84070 | 1.177 | 0.91 | 0.91 | 2541 | 821 | 826 |
| D83005 | 1.177 | 1.12 | 1.11 | 2542 | 2608 | 2538 |
| M87034 | 1.177 | 1.12 | 1.12 | 2543 | 2649 | 2617 |
| A86016 | 1.177 | 1.04 | 1.06 | 2544 | 1815 | 1982 |

| | | | | | | |
|---|---|---|---|---|---|---|
| Y00293 | 1.177 | 1.03 | 1.02 | 2545 | 1648 | 1637 |
| C81604 | 1.177 | 1.25 | 1.23 | 2546 | 4441 | 4178 |
| L83627 | 1.177 | 1.20 | 1.17 | 2547 | 3780 | 3328 |
| B85001 | 1.177 | 1.10 | 1.10 | 2548 | 2450 | 2457 |
| P89008 | 1.177 | 1.23 | 1.25 | 2549 | 4227 | 4507 |
| F85072 | 1.176 | 1.01 | 1.01 | 2550 | 1525 | 1487 |
| G82808 | 1.176 | 1.09 | 1.09 | 2551 | 2327 | 2330 |
| J82015 | 1.176 | 1.06 | 1.06 | 2552 | 1960 | 1931 |
| F81031 | 1.176 | 1.22 | 1.23 | 2553 | 4049 | 4183 |
| N81039 | 1.176 | 1.16 | 1.14 | 2554 | 3145 | 2889 |
| M85111 | 1.176 | 1.18 | 1.16 | 2555 | 3473 | 3185 |
| N82074 | 1.176 | 1.12 | 1.14 | 2556 | 2645 | 2878 |
| F82679 | 1.176 | 0.93 | 0.94 | 2557 | 954 | 1019 |
| F85678 | 1.176 | 1.01 | 1.01 | 2558 | 1502 | 1492 |
| A87015 | 1.176 | 1.27 | 1.28 | 2559 | 4716 | 4810 |
| L83125 | 1.175 | 1.05 | 1.07 | 2560 | 1875 | 2113 |
| K81001 | 1.175 | 1.00 | 1.00 | 2561 | 1458 | 1434 |
| Y02767 | 1.175 | 0.94 | 0.97 | 2562 | 1029 | 1221 |
| P84679 | 1.175 | 1.05 | 1.07 | 2563 | 1927 | 2109 |
| L83092 | 1.175 | 1.13 | 1.14 | 2564 | 2750 | 3006 |
| L82618 | 1.175 | 1.27 | 1.27 | 2565 | 4783 | 4713 |
| E82079 | 1.175 | 1.05 | 1.05 | 2566 | 1938 | 1919 |
| B83032 | 1.175 | 1.21 | 1.22 | 2567 | 3924 | 4084 |
| A81621 | 1.175 | 1.21 | 1.23 | 2568 | 3982 | 4166 |
| J82034 | 1.175 | 1.08 | 1.05 | 2569 | 2188 | 1913 |
| H82015 | 1.174 | 1.15 | 1.15 | 2570 | 3059 | 3101 |
| P81053 | 1.174 | 1.20 | 1.22 | 2571 | 3778 | 4077 |
| E81016 | 1.174 | 1.11 | 1.12 | 2572 | 2624 | 2722 |
| G81661 | 1.174 | 1.05 | 1.07 | 2573 | 1948 | 2126 |
| C87016 | 1.174 | 1.11 | 1.12 | 2574 | 2583 | 2720 |
| Y02606 | 1.174 | 1.12 | 1.13 | 2575 | 2757 | 2788 |
| C81104 | 1.174 | 1.13 | 1.10 | 2576 | 2848 | 2480 |
| L82010 | 1.174 | 1.15 | 1.15 | 2577 | 3035 | 3057 |
| J83010 | 1.173 | 1.06 | 1.05 | 2578 | 2036 | 1947 |
| N82083 | 1.173 | 1.11 | 1.13 | 2579 | 2620 | 2813 |
| P92002 | 1.173 | 1.14 | 1.14 | 2580 | 2912 | 3033 |
| F82641 | 1.173 | 0.94 | 0.94 | 2581 | 1052 | 1057 |
| H82031 | 1.173 | 1.07 | 1.08 | 2582 | 2087 | 2217 |
| Y02735 | 1.173 | 1.16 | 1.17 | 2583 | 3169 | 3347 |
| N85052 | 1.173 | 1.18 | 1.17 | 2584 | 3536 | 3386 |
| C84061 | 1.173 | 1.13 | 1.15 | 2585 | 2836 | 3058 |
| L81053 | 1.173 | 1.18 | 1.18 | 2586 | 3549 | 3598 |
| D81622 | 1.173 | 1.10 | 1.14 | 2587 | 2501 | 2952 |
| C88070 | 1.173 | 1.20 | 1.22 | 2588 | 3852 | 4043 |
| P81082 | 1.173 | 1.16 | 1.15 | 2589 | 3195 | 3137 |
| E82080 | 1.173 | 1.08 | 1.07 | 2590 | 2257 | 2103 |
| C88009 | 1.172 | 1.31 | 1.32 | 2591 | 5321 | 5408 |
| P88600 | 1.172 | 1.09 | 1.09 | 2592 | 2357 | 2397 |
| G82666 | 1.172 | 1.30 | 1.31 | 2593 | 5118 | 5212 |
| M85006 | 1.172 | 1.18 | 1.16 | 2594 | 3581 | 3323 |

| | | | | | | |
|---|---|---|---|---|---|---|
| M85756 | 1.172 | 1.09 | 1.08 | 2595 | 2353 | 2297 |
| M84035 | 1.172 | 1.03 | 1.02 | 2596 | 1722 | 1619 |
| D82632 | 1.172 | 1.17 | 1.18 | 2597 | 3420 | 3574 |
| J81028 | 1.172 | 1.29 | 1.28 | 2598 | 5068 | 4903 |
| Y03366 | 1.172 | 1.15 | 1.17 | 2599 | 3108 | 3410 |
| D82046 | 1.172 | 1.11 | 1.10 | 2600 | 2662 | 2555 |
| A86004 | 1.172 | 1.03 | 1.04 | 2601 | 1756 | 1862 |
| K82033 | 1.172 | 1.09 | 1.07 | 2602 | 2378 | 2133 |
| G82061 | 1.171 | 1.01 | 1.02 | 2603 | 1573 | 1640 |
| L83097 | 1.171 | 1.07 | 1.06 | 2604 | 2100 | 2036 |
| H81080 | 1.171 | 0.97 | 0.96 | 2605 | 1267 | 1174 |
| F84681 | 1.171 | 0.94 | 0.93 | 2606 | 1064 | 982 |
| H81015 | 1.171 | 0.93 | 0.92 | 2607 | 1005 | 919 |
| C81004 | 1.171 | 1.11 | 1.11 | 2608 | 2632 | 2589 |
| F81713 | 1.171 | 0.91 | 0.91 | 2609 | 891 | 877 |
| P81003 | 1.171 | 1.15 | 1.15 | 2610 | 3090 | 3182 |
| B87032 | 1.171 | 1.16 | 1.18 | 2611 | 3272 | 3543 |
| D82070 | 1.170 | 1.19 | 1.19 | 2612 | 3715 | 3711 |
| B81035 | 1.170 | 1.06 | 1.11 | 2613 | 2099 | 2599 |
| P92637 | 1.170 | 1.04 | 1.05 | 2614 | 1888 | 1971 |
| M81065 | 1.170 | 1.15 | 1.13 | 2615 | 3114 | 2920 |
| D82034 | 1.170 | 1.10 | 1.09 | 2616 | 2520 | 2428 |
| G82079 | 1.170 | 1.24 | 1.25 | 2617 | 4355 | 4490 |
| G84005 | 1.170 | 1.14 | 1.12 | 2618 | 2955 | 2790 |
| D82002 | 1.170 | 1.14 | 1.13 | 2619 | 3011 | 2817 |
| M89021 | 1.170 | 1.24 | 1.22 | 2620 | 4391 | 4110 |
| E81033 | 1.170 | 1.00 | 1.00 | 2621 | 1455 | 1463 |
| Y00486 | 1.170 | 0.93 | 0.94 | 2622 | 1010 | 1065 |
| C82075 | 1.170 | 1.11 | 1.11 | 2623 | 2631 | 2634 |
| L83011 | 1.170 | 1.12 | 1.10 | 2624 | 2765 | 2472 |
| M83139 | 1.170 | 1.19 | 1.22 | 2625 | 3645 | 4160 |
| P82031 | 1.170 | 1.07 | 1.09 | 2626 | 2175 | 2369 |
| C82023 | 1.170 | 1.18 | 1.18 | 2627 | 3605 | 3593 |
| C82667 | 1.169 | 1.16 | 1.16 | 2628 | 3228 | 3278 |
| D81085 | 1.169 | 1.00 | 0.99 | 2629 | 1489 | 1410 |
| M83627 | 1.169 | 1.21 | 1.24 | 2630 | 4026 | 4396 |
| F81091 | 1.169 | 1.11 | 1.10 | 2631 | 2601 | 2553 |
| B84014 | 1.169 | 1.17 | 1.18 | 2632 | 3363 | 3667 |
| F82604 | 1.169 | 1.01 | 1.02 | 2633 | 1583 | 1680 |
| N83624 | 1.169 | 1.26 | 1.27 | 2634 | 4729 | 4805 |
| G82073 | 1.169 | 1.00 | 1.00 | 2635 | 1460 | 1510 |
| N82046 | 1.168 | 1.05 | 1.06 | 2636 | 1942 | 2070 |
| M84004 | 1.168 | 1.04 | 1.03 | 2637 | 1898 | 1802 |
| Y02321 | 1.168 | 1.12 | 1.13 | 2638 | 2751 | 2839 |
| M87037 | 1.168 | 1.07 | 1.07 | 2639 | 2214 | 2186 |
| M88024 | 1.168 | 1.10 | 1.11 | 2640 | 2519 | 2697 |
| A83644 | 1.168 | 1.26 | 1.26 | 2641 | 4662 | 4698 |
| B82036 | 1.168 | 1.11 | 1.11 | 2642 | 2643 | 2637 |
| N82093 | 1.168 | 1.16 | 1.17 | 2643 | 3253 | 3383 |
| L81038 | 1.168 | 1.03 | 1.04 | 2644 | 1788 | 1859 |

| | | | | | | |
|---|---|---|---|---|---|---|
| Y02688 | 1.168 | 0.87 | 0.86 | 2645 | 687 | 664 |
| G82799 | 1.168 | 1.14 | 1.15 | 2646 | 3009 | 3144 |
| H85052 | 1.168 | 1.13 | 1.10 | 2647 | 2936 | 2569 |
| M85652 | 1.168 | 1.01 | 1.00 | 2648 | 1541 | 1499 |
| D81063 | 1.167 | 0.96 | 0.96 | 2649 | 1182 | 1231 |
| G82206 | 1.167 | 0.94 | 0.96 | 2650 | 1090 | 1166 |
| B85660 | 1.167 | 1.07 | 1.07 | 2651 | 2182 | 2208 |
| L84028 | 1.167 | 1.27 | 1.25 | 2652 | 4861 | 4600 |
| E86004 | 1.167 | 1.01 | 1.01 | 2653 | 1627 | 1559 |
| M84061 | 1.167 | 1.09 | 1.10 | 2654 | 2453 | 2535 |
| C83068 | 1.167 | 1.04 | 1.03 | 2655 | 1842 | 1735 |
| Y02811 | 1.167 | 0.86 | 0.86 | 2656 | 666 | 665 |
| M92630 | 1.167 | 1.28 | 1.29 | 2657 | 4962 | 5038 |
| B86039 | 1.167 | 1.00 | 1.02 | 2658 | 1523 | 1712 |
| K81607 | 1.167 | 1.17 | 1.15 | 2659 | 3498 | 3261 |
| M92031 | 1.167 | 1.15 | 1.17 | 2660 | 3146 | 3480 |
| F82017 | 1.166 | 1.08 | 1.08 | 2661 | 2272 | 2287 |
| F86012 | 1.166 | 0.98 | 0.96 | 2662 | 1337 | 1205 |
| J82094 | 1.166 | 1.04 | 1.03 | 2663 | 1924 | 1761 |
| Y00028 | 1.166 | 0.98 | 0.99 | 2664 | 1363 | 1445 |
| P92031 | 1.166 | 1.15 | 1.15 | 2665 | 3103 | 3252 |
| N82107 | 1.166 | 1.14 | 1.15 | 2666 | 2999 | 3203 |
| N81619 | 1.166 | 1.19 | 1.19 | 2667 | 3716 | 3790 |
| B81024 | 1.166 | 1.02 | 1.03 | 2668 | 1702 | 1750 |
| A81035 | 1.166 | 1.17 | 1.19 | 2669 | 3502 | 3731 |
| L84043 | 1.166 | 1.09 | 1.08 | 2670 | 2461 | 2371 |
| F84641 | 1.166 | 1.12 | 1.11 | 2671 | 2838 | 2614 |
| H81119 | 1.166 | 1.00 | 0.97 | 2672 | 1490 | 1324 |
| M85706 | 1.166 | 1.14 | 1.12 | 2673 | 3065 | 2845 |
| F86637 | 1.166 | 1.09 | 1.08 | 2674 | 2491 | 2289 |
| L83628 | 1.166 | 1.17 | 1.14 | 2675 | 3481 | 3010 |
| M85801 | 1.165 | 1.10 | 1.09 | 2676 | 2547 | 2493 |
| M83100 | 1.165 | 1.23 | 1.26 | 2677 | 4280 | 4794 |
| C82024 | 1.165 | 1.17 | 1.18 | 2678 | 3496 | 3625 |
| D82015 | 1.165 | 1.30 | 1.29 | 2679 | 5220 | 5137 |
| C81025 | 1.165 | 1.10 | 1.10 | 2680 | 2605 | 2600 |
| B84016 | 1.165 | 1.15 | 1.16 | 2681 | 3187 | 3385 |
| P87002 | 1.165 | 1.18 | 1.20 | 2682 | 3599 | 3994 |
| P81731 | 1.165 | 1.13 | 1.17 | 2683 | 2952 | 3526 |
| L84031 | 1.165 | 1.13 | 1.11 | 2684 | 2906 | 2683 |
| H82058 | 1.165 | 1.09 | 1.08 | 2685 | 2456 | 2370 |
| M84059 | 1.165 | 1.23 | 1.22 | 2686 | 4326 | 4281 |
| C81050 | 1.164 | 1.20 | 1.20 | 2687 | 3976 | 3919 |
| N85008 | 1.164 | 1.11 | 1.11 | 2688 | 2697 | 2662 |
| C84081 | 1.164 | 1.19 | 1.21 | 2689 | 3805 | 4119 |
| B81021 | 1.164 | 1.21 | 1.25 | 2690 | 4041 | 4700 |
| G81043 | 1.164 | 1.01 | 0.99 | 2691 | 1619 | 1461 |
| P81063 | 1.164 | 1.30 | 1.34 | 2692 | 5282 | 5625 |
| L82053 | 1.164 | 1.24 | 1.23 | 2693 | 4414 | 4393 |
| M81627 | 1.164 | 1.03 | 1.02 | 2694 | 1832 | 1689 |

| | | | | | | |
|---|---|---|---|---|---|---|
| L85004 | 1.164 | 1.19 | 1.19 | 2695 | 3763 | 3784 |
| N85616 | 1.164 | 1.12 | 1.10 | 2696 | 2779 | 2641 |
| F85048 | 1.164 | 1.05 | 1.05 | 2697 | 2010 | 1957 |
| P88607 | 1.164 | 1.18 | 1.18 | 2698 | 3720 | 3685 |
| F85058 | 1.163 | 1.04 | 1.04 | 2699 | 1957 | 1917 |
| N84029 | 1.163 | 1.21 | 1.21 | 2700 | 4147 | 4083 |
| C81063 | 1.163 | 1.10 | 1.09 | 2701 | 2562 | 2475 |
| E85718 | 1.163 | 0.95 | 0.94 | 2702 | 1167 | 1130 |
| F86085 | 1.162 | 1.02 | 1.00 | 2703 | 1752 | 1588 |
| L85008 | 1.162 | 1.07 | 1.05 | 2704 | 2290 | 2076 |
| K82039 | 1.162 | 1.03 | 1.02 | 2705 | 1845 | 1777 |
| D83018 | 1.162 | 1.15 | 1.15 | 2706 | 3209 | 3326 |
| D83039 | 1.162 | 1.11 | 1.12 | 2707 | 2715 | 2807 |
| F82677 | 1.162 | 1.03 | 1.04 | 2708 | 1830 | 1904 |
| C88033 | 1.162 | 1.29 | 1.30 | 2709 | 5181 | 5228 |
| L82018 | 1.162 | 1.15 | 1.15 | 2710 | 3198 | 3290 |
| J81006 | 1.162 | 1.16 | 1.15 | 2711 | 3311 | 3329 |
| A81013 | 1.162 | 1.15 | 1.15 | 2712 | 3248 | 3299 |
| G82218 | 1.161 | 1.00 | 0.99 | 2713 | 1596 | 1465 |
| P87016 | 1.161 | 1.06 | 1.09 | 2714 | 2141 | 2497 |
| M85005 | 1.161 | 1.13 | 1.13 | 2715 | 3033 | 2951 |
| L81026 | 1.161 | 1.06 | 1.06 | 2716 | 2162 | 2092 |
| F81010 | 1.161 | 1.13 | 1.13 | 2717 | 2977 | 2971 |
| L83057 | 1.160 | 1.24 | 1.23 | 2718 | 4556 | 4436 |
| N82081 | 1.160 | 1.40 | 1.40 | 2719 | 6259 | 6326 |
| C81095 | 1.160 | 1.10 | 1.10 | 2720 | 2627 | 2604 |
| B81688 | 1.160 | 1.26 | 1.29 | 2721 | 4790 | 5160 |
| J82041 | 1.160 | 1.19 | 1.16 | 2722 | 3775 | 3444 |
| J82155 | 1.160 | 1.13 | 1.14 | 2723 | 3023 | 3176 |
| G83031 | 1.160 | 1.09 | 1.08 | 2724 | 2474 | 2406 |
| M81035 | 1.160 | 1.08 | 1.09 | 2725 | 2440 | 2536 |
| A88005 | 1.160 | 1.26 | 1.25 | 2726 | 4826 | 4666 |
| G81614 | 1.160 | 1.05 | 1.03 | 2727 | 2108 | 1809 |
| C82031 | 1.160 | 1.09 | 1.09 | 2728 | 2466 | 2502 |
| K82065 | 1.160 | 0.95 | 0.95 | 2729 | 1215 | 1184 |
| P82001 | 1.159 | 1.23 | 1.23 | 2730 | 4348 | 4416 |
| F81113 | 1.159 | 0.92 | 0.93 | 2731 | 1003 | 1069 |
| C82114 | 1.159 | 1.04 | 1.05 | 2732 | 1995 | 2012 |
| L82050 | 1.159 | 1.18 | 1.17 | 2733 | 3665 | 3635 |
| D83029 | 1.159 | 1.04 | 1.06 | 2734 | 2009 | 2116 |
| F86638 | 1.159 | 1.11 | 1.12 | 2735 | 2700 | 2890 |
| A82617 | 1.159 | 1.12 | 1.11 | 2736 | 2928 | 2758 |
| L81029 | 1.159 | 1.02 | 1.02 | 2737 | 1810 | 1804 |
| H85683 | 1.159 | 1.00 | 0.98 | 2738 | 1539 | 1400 |
| M83054 | 1.159 | 1.16 | 1.17 | 2739 | 3446 | 3567 |
| K81075 | 1.159 | 1.05 | 1.05 | 2740 | 2044 | 2111 |
| B86103 | 1.158 | 1.17 | 1.18 | 2741 | 3561 | 3779 |
| N85058 | 1.158 | 1.09 | 1.09 | 2742 | 2543 | 2524 |
| B87002 | 1.158 | 1.11 | 1.14 | 2743 | 2767 | 3128 |
| M88008 | 1.158 | 1.17 | 1.18 | 2744 | 3556 | 3732 |

| | | | | | | |
|---|---|---|---|---|---|---|
| F81163 | 1.158 | 1.11 | 1.06 | 2745 | 2799 | 2237 |
| G82754 | 1.158 | 1.08 | 1.06 | 2746 | 2341 | 2212 |
| B85024 | 1.158 | 1.17 | 1.17 | 2747 | 3602 | 3642 |
| E81027 | 1.158 | 1.14 | 1.13 | 2748 | 3185 | 3022 |
| P91011 | 1.158 | 0.99 | 1.00 | 2749 | 1450 | 1550 |
| P92634 | 1.158 | 1.03 | 1.04 | 2750 | 1891 | 2006 |
| D82600 | 1.158 | 1.32 | 1.33 | 2751 | 5562 | 5616 |
| N85003 | 1.158 | 1.17 | 1.15 | 2752 | 3582 | 3369 |
| G82670 | 1.158 | 1.08 | 1.11 | 2753 | 2443 | 2774 |
| H82092 | 1.158 | 0.97 | 0.95 | 2754 | 1384 | 1226 |
| M81029 | 1.158 | 1.09 | 1.08 | 2755 | 2545 | 2426 |
| B87004 | 1.158 | 1.18 | 1.19 | 2756 | 3687 | 3906 |
| F86030 | 1.157 | 1.03 | 1.05 | 2757 | 1876 | 2053 |
| P85020 | 1.157 | 1.14 | 1.15 | 2758 | 3094 | 3333 |
| E82663 | 1.157 | 0.98 | 0.98 | 2759 | 1454 | 1444 |
| M86612 | 1.157 | 1.09 | 1.08 | 2760 | 2517 | 2454 |
| J82100 | 1.157 | 1.22 | 1.24 | 2761 | 4343 | 4650 |
| K83015 | 1.157 | 1.10 | 1.10 | 2762 | 2650 | 2651 |
| F82630 | 1.157 | 1.14 | 1.13 | 2763 | 3168 | 3037 |
| C81101 | 1.157 | 1.10 | 1.10 | 2764 | 2637 | 2629 |
| Y02517 | 1.157 | 1.06 | 1.07 | 2765 | 2252 | 2267 |
| E81077 | 1.157 | 1.05 | 1.05 | 2766 | 2101 | 2041 |
| G83062 | 1.157 | 1.03 | 1.02 | 2767 | 1906 | 1793 |
| C82048 | 1.157 | 1.03 | 1.03 | 2768 | 1871 | 1897 |
| M92029 | 1.157 | 1.20 | 1.22 | 2769 | 4057 | 4267 |
| P81025 | 1.157 | 1.23 | 1.25 | 2770 | 4394 | 4764 |
| B83061 | 1.157 | 1.14 | 1.15 | 2771 | 3133 | 3277 |
| B82054 | 1.156 | 1.20 | 1.22 | 2772 | 4008 | 4271 |
| C88050 | 1.156 | 1.32 | 1.32 | 2773 | 5505 | 5553 |
| K83013 | 1.156 | 1.09 | 1.09 | 2774 | 2551 | 2508 |
| N82090 | 1.156 | 1.19 | 1.21 | 2775 | 3968 | 4135 |
| G82203 | 1.156 | 1.04 | 1.06 | 2776 | 1946 | 2243 |
| L82004 | 1.156 | 1.14 | 1.14 | 2777 | 3206 | 3250 |
| A81602 | 1.156 | 1.17 | 1.17 | 2778 | 3607 | 3674 |
| L84080 | 1.156 | 1.15 | 1.14 | 2779 | 3258 | 3218 |
| E85069 | 1.156 | 1.03 | 1.03 | 2780 | 1858 | 1869 |
| P81710 | 1.156 | 1.14 | 1.15 | 2781 | 3126 | 3314 |
| N84630 | 1.156 | 1.08 | 1.08 | 2782 | 2462 | 2427 |
| J81004 | 1.155 | 1.05 | 1.05 | 2783 | 2132 | 2135 |
| E81014 | 1.155 | 1.12 | 1.11 | 2784 | 2946 | 2831 |
| K83020 | 1.155 | 1.01 | 1.02 | 2785 | 1707 | 1825 |
| C86003 | 1.155 | 1.36 | 1.38 | 2786 | 5967 | 6134 |
| K83039 | 1.155 | 1.19 | 1.18 | 2787 | 3937 | 3825 |
| F81080 | 1.155 | 1.03 | 1.04 | 2788 | 1855 | 1969 |
| P92023 | 1.155 | 1.24 | 1.24 | 2789 | 4568 | 4611 |
| B86673 | 1.155 | 1.32 | 1.33 | 2790 | 5513 | 5649 |
| F81134 | 1.155 | 1.18 | 1.17 | 2791 | 3728 | 3603 |
| M85105 | 1.155 | 1.22 | 1.21 | 2792 | 4380 | 4159 |
| J81644 | 1.155 | 0.98 | 0.97 | 2793 | 1407 | 1389 |
| A83023 | 1.155 | 1.40 | 1.39 | 2794 | 6291 | 6277 |

| | | | | | | |
|---|---|---|---|---|---|---|
| C81086 | 1.155 | 1.02 | 1.02 | 2795 | 1850 | 1845 |
| P81153 | 1.155 | 1.29 | 1.29 | 2796 | 5225 | 5244 |
| P81117 | 1.154 | 1.23 | 1.21 | 2797 | 4483 | 4285 |
| P81714 | 1.154 | 1.24 | 1.29 | 2798 | 4631 | 5197 |
| A82010 | 1.154 | 1.17 | 1.20 | 2799 | 3669 | 4102 |
| E86036 | 1.154 | 1.03 | 1.02 | 2800 | 1885 | 1830 |
| L81044 | 1.154 | 1.13 | 1.11 | 2801 | 3078 | 2800 |
| P89027 | 1.154 | 1.16 | 1.18 | 2802 | 3456 | 3781 |
| H82025 | 1.154 | 1.03 | 1.05 | 2803 | 1954 | 2093 |
| P81181 | 1.154 | 1.10 | 1.09 | 2804 | 2710 | 2633 |
| J82187 | 1.154 | 1.16 | 1.16 | 2805 | 3516 | 3579 |
| H81081 | 1.154 | 1.08 | 1.06 | 2806 | 2432 | 2192 |
| A87002 | 1.154 | 1.32 | 1.31 | 2807 | 5548 | 5491 |
| E83028 | 1.154 | 1.10 | 1.07 | 2808 | 2669 | 2300 |
| G82015 | 1.154 | 1.09 | 1.10 | 2809 | 2585 | 2733 |
| F81074 | 1.154 | 1.14 | 1.13 | 2810 | 3170 | 3099 |
| L82056 | 1.154 | 1.18 | 1.18 | 2811 | 3800 | 3831 |
| H81131 | 1.154 | 0.96 | 0.95 | 2812 | 1340 | 1240 |
| F81186 | 1.153 | 1.11 | 1.11 | 2813 | 2806 | 2780 |
| G83046 | 1.153 | 1.02 | 1.01 | 2814 | 1865 | 1768 |
| N81018 | 1.153 | 1.04 | 1.03 | 2815 | 2043 | 1901 |
| D82007 | 1.153 | 1.28 | 1.29 | 2816 | 5178 | 5286 |
| G82060 | 1.153 | 1.11 | 1.11 | 2817 | 2788 | 2853 |
| C85619 | 1.153 | 1.21 | 1.22 | 2818 | 4187 | 4325 |
| L84022 | 1.153 | 1.06 | 1.06 | 2819 | 2212 | 2273 |
| H81013 | 1.153 | 1.02 | 1.00 | 2820 | 1779 | 1662 |
| J81055 | 1.153 | 1.18 | 1.18 | 2821 | 3876 | 3806 |
| E82106 | 1.153 | 0.99 | 0.98 | 2822 | 1522 | 1437 |
| G83029 | 1.153 | 1.00 | 0.99 | 2823 | 1599 | 1579 |
| L81098 | 1.153 | 1.18 | 1.18 | 2824 | 3830 | 3833 |
| B85621 | 1.153 | 1.33 | 1.32 | 2825 | 5700 | 5589 |
| J82151 | 1.152 | 1.17 | 1.16 | 2826 | 3631 | 3571 |
| J83039 | 1.152 | 1.07 | 1.06 | 2827 | 2389 | 2290 |
| F84735 | 1.152 | 0.99 | 0.98 | 2828 | 1565 | 1471 |
| B85612 | 1.152 | 1.34 | 1.32 | 2829 | 5782 | 5570 |
| P81051 | 1.152 | 1.16 | 1.18 | 2830 | 3493 | 3885 |
| J83601 | 1.152 | 1.02 | 1.01 | 2831 | 1799 | 1748 |
| C84065 | 1.152 | 1.07 | 1.07 | 2832 | 2344 | 2331 |
| K81644 | 1.152 | 0.96 | 0.97 | 2833 | 1326 | 1366 |
| M83089 | 1.152 | 1.23 | 1.18 | 2834 | 4504 | 3792 |
| M83052 | 1.152 | 0.96 | 0.96 | 2835 | 1331 | 1287 |
| L83131 | 1.152 | 1.16 | 1.17 | 2836 | 3571 | 3682 |
| A87008 | 1.152 | 1.02 | 1.04 | 2837 | 1841 | 2000 |
| L84072 | 1.151 | 1.11 | 1.09 | 2838 | 2829 | 2653 |
| H81667 | 1.151 | 0.94 | 0.93 | 2839 | 1187 | 1099 |
| P85026 | 1.151 | 1.16 | 1.18 | 2840 | 3591 | 3835 |
| A89012 | 1.151 | 1.18 | 1.19 | 2841 | 3821 | 3944 |
| M82012 | 1.151 | 1.11 | 1.12 | 2842 | 2884 | 3036 |
| C88084 | 1.151 | 1.32 | 1.32 | 2843 | 5616 | 5629 |
| N83002 | 1.151 | 1.22 | 1.23 | 2844 | 4411 | 4584 |

| | | | | | | |
|---|---|---|---|---|---|---|
| P81721 | 1.151 | 1.15 | 1.17 | 2845 | 3430 | 3752 |
| B82033 | 1.151 | 1.17 | 1.19 | 2846 | 3667 | 4008 |
| C88061 | 1.151 | 0.97 | 1.00 | 2847 | 1419 | 1643 |
| C83055 | 1.151 | 1.17 | 1.20 | 2848 | 3754 | 4131 |
| M86633 | 1.151 | 1.11 | 1.10 | 2849 | 2852 | 2770 |
| K81084 | 1.151 | 0.96 | 0.95 | 2850 | 1350 | 1266 |
| D81050 | 1.151 | 1.09 | 1.09 | 2851 | 2668 | 2613 |
| M85699 | 1.150 | 1.25 | 1.23 | 2852 | 4739 | 4603 |
| K83076 | 1.150 | 1.11 | 1.12 | 2853 | 2850 | 3035 |
| B83020 | 1.150 | 1.12 | 1.14 | 2854 | 3021 | 3214 |
| C81027 | 1.150 | 1.18 | 1.17 | 2855 | 3827 | 3719 |
| J81076 | 1.150 | 1.17 | 1.15 | 2856 | 3765 | 3433 |
| F81097 | 1.150 | 1.04 | 1.05 | 2857 | 2052 | 2162 |
| L82028 | 1.150 | 1.03 | 1.04 | 2858 | 1947 | 2021 |
| M83148 | 1.150 | 1.05 | 1.05 | 2859 | 2173 | 2153 |
| N85022 | 1.150 | 1.30 | 1.28 | 2860 | 5398 | 5131 |
| Y02787 | 1.150 | 0.93 | 0.95 | 2861 | 1106 | 1253 |
| M81054 | 1.150 | 1.14 | 1.12 | 2862 | 3231 | 3068 |
| G82021 | 1.150 | 1.05 | 1.05 | 2863 | 2168 | 2174 |
| L83003 | 1.150 | 1.22 | 1.21 | 2864 | 4454 | 4294 |
| B86033 | 1.150 | 1.08 | 1.10 | 2865 | 2498 | 2776 |
| Y03124 | 1.150 | 1.23 | 1.24 | 2866 | 4488 | 4725 |
| J84010 | 1.150 | 1.23 | 1.23 | 2867 | 4558 | 4506 |
| P92653 | 1.149 | 1.20 | 1.21 | 2868 | 4190 | 4244 |
| C81611 | 1.149 | 1.07 | 1.07 | 2869 | 2334 | 2363 |
| D81043 | 1.149 | 1.10 | 1.08 | 2870 | 2695 | 2575 |
| P86026 | 1.149 | 1.15 | 1.16 | 2871 | 3370 | 3600 |
| P81734 | 1.149 | 1.22 | 1.24 | 2872 | 4344 | 4714 |
| L81006 | 1.149 | 1.05 | 1.06 | 2873 | 2226 | 2332 |
| E85696 | 1.149 | 0.90 | 0.90 | 2874 | 961 | 936 |
| K82008 | 1.149 | 0.98 | 0.96 | 2875 | 1462 | 1329 |
| Y02660 | 1.149 | 1.17 | 1.16 | 2876 | 3690 | 3582 |
| P83608 | 1.149 | 1.19 | 1.19 | 2877 | 4031 | 4052 |
| A81004 | 1.149 | 1.16 | 1.17 | 2878 | 3566 | 3749 |
| M82051 | 1.148 | 1.13 | 1.13 | 2879 | 3120 | 3151 |
| F83678 | 1.148 | 1.17 | 1.17 | 2880 | 3694 | 3698 |
| G82671 | 1.148 | 0.95 | 0.97 | 2881 | 1243 | 1387 |
| G81694 | 1.148 | 0.92 | 0.94 | 2882 | 1091 | 1191 |
| J82033 | 1.148 | 1.04 | 1.03 | 2883 | 2079 | 1961 |
| C84144 | 1.148 | 1.02 | 1.04 | 2884 | 1912 | 2086 |
| B85025 | 1.148 | 1.11 | 1.11 | 2885 | 2893 | 2975 |
| P87008 | 1.148 | 1.21 | 1.23 | 2886 | 4292 | 4645 |
| K83610 | 1.147 | 0.96 | 0.97 | 2887 | 1324 | 1442 |
| F81152 | 1.147 | 1.08 | 1.05 | 2888 | 2523 | 2244 |
| H81060 | 1.147 | 1.00 | 0.98 | 2889 | 1717 | 1507 |
| G82118 | 1.147 | 1.08 | 1.08 | 2890 | 2593 | 2534 |
| Y01839 | 1.147 | 1.10 | 1.12 | 2891 | 2819 | 2984 |
| K83079 | 1.147 | 1.04 | 1.08 | 2892 | 2138 | 2581 |
| J81612 | 1.147 | 1.27 | 1.22 | 2893 | 5059 | 4426 |
| L81039 | 1.147 | 0.98 | 0.97 | 2894 | 1507 | 1449 |

| | | | | | | |
|---|---|---|---|---|---|---|
| F81758 | 1.147 | 1.18 | 1.19 | 2895 | 3946 | 4104 |
| B85623 | 1.147 | 1.15 | 1.16 | 2896 | 3460 | 3545 |
| C88038 | 1.147 | 1.20 | 1.21 | 2897 | 4133 | 4370 |
| M83109 | 1.147 | 1.13 | 1.17 | 2898 | 3131 | 3737 |
| M85171 | 1.147 | 1.08 | 1.07 | 2899 | 2504 | 2392 |
| G82698 | 1.147 | 0.98 | 0.99 | 2900 | 1500 | 1633 |
| G81050 | 1.147 | 1.16 | 1.18 | 2901 | 3596 | 3860 |
| C82043 | 1.147 | 1.09 | 1.09 | 2902 | 2689 | 2706 |
| K82043 | 1.147 | 1.08 | 1.06 | 2903 | 2558 | 2269 |
| G82160 | 1.146 | 1.17 | 1.21 | 2904 | 3812 | 4322 |
| L82035 | 1.146 | 1.20 | 1.21 | 2905 | 4230 | 4313 |
| J83049 | 1.146 | 1.12 | 1.10 | 2906 | 3006 | 2829 |
| N85643 | 1.146 | 1.18 | 1.17 | 2907 | 3916 | 3708 |
| M84001 | 1.146 | 1.14 | 1.14 | 2908 | 3295 | 3291 |
| M83076 | 1.146 | 1.05 | 1.09 | 2909 | 2261 | 2663 |
| F81125 | 1.146 | 1.07 | 1.05 | 2910 | 2410 | 2160 |
| N82646 | 1.146 | 1.11 | 1.12 | 2911 | 2904 | 3072 |
| G83635 | 1.146 | 1.05 | 1.05 | 2912 | 2267 | 2239 |
| P81072 | 1.146 | 1.24 | 1.28 | 2913 | 4678 | 5200 |
| E85058 | 1.145 | 1.10 | 1.09 | 2914 | 2798 | 2670 |
| B85620 | 1.145 | 1.08 | 1.09 | 2915 | 2574 | 2713 |
| M83625 | 1.145 | 1.18 | 1.22 | 2916 | 3951 | 4482 |
| P89612 | 1.145 | 1.08 | 1.10 | 2917 | 2617 | 2819 |
| F81108 | 1.145 | 1.10 | 1.11 | 2918 | 2812 | 2925 |
| M82031 | 1.145 | 1.15 | 1.14 | 2919 | 3459 | 3325 |
| G83043 | 1.145 | 1.05 | 1.04 | 2920 | 2235 | 2096 |
| F82021 | 1.145 | 0.98 | 0.98 | 2921 | 1514 | 1541 |
| L84023 | 1.145 | 1.12 | 1.08 | 2922 | 3067 | 2566 |
| F84670 | 1.145 | 1.06 | 1.05 | 2923 | 2376 | 2251 |
| P84682 | 1.145 | 1.02 | 1.04 | 2924 | 1920 | 2134 |
| F81019 | 1.145 | 1.09 | 1.10 | 2925 | 2690 | 2826 |
| P88013 | 1.145 | 1.11 | 1.11 | 2926 | 2980 | 2932 |
| L83056 | 1.145 | 1.16 | 1.13 | 2927 | 3648 | 3169 |
| E83003 | 1.144 | 1.00 | 0.98 | 2928 | 1700 | 1515 |
| B84615 | 1.144 | 1.10 | 1.12 | 2929 | 2871 | 3124 |
| P81215 | 1.144 | 1.25 | 1.26 | 2930 | 4871 | 4968 |
| A83044 | 1.144 | 1.28 | 1.26 | 2931 | 5208 | 5027 |
| K81034 | 1.144 | 1.01 | 1.02 | 2932 | 1792 | 1889 |
| M81004 | 1.144 | 1.09 | 1.08 | 2933 | 2664 | 2521 |
| P84684 | 1.144 | 1.20 | 1.23 | 2934 | 4164 | 4566 |
| M87027 | 1.144 | 1.19 | 1.18 | 2935 | 4122 | 3991 |
| P82015 | 1.144 | 1.16 | 1.17 | 2936 | 3636 | 3863 |
| B86106 | 1.144 | 1.09 | 1.10 | 2937 | 2755 | 2883 |
| H81128 | 1.144 | 0.96 | 0.95 | 2938 | 1392 | 1303 |
| E85699 | 1.144 | 1.17 | 1.15 | 2939 | 3806 | 3455 |
| B81656 | 1.144 | 1.31 | 1.32 | 2940 | 5567 | 5636 |
| P81115 | 1.143 | 1.28 | 1.32 | 2941 | 5313 | 5655 |
| C84646 | 1.143 | 1.07 | 1.05 | 2942 | 2418 | 2229 |
| N81024 | 1.143 | 1.22 | 1.18 | 2943 | 4421 | 4010 |
| B86006 | 1.143 | 1.10 | 1.11 | 2944 | 2774 | 2989 |

| | | | | | | |
|---|---|---|---|---|---|---|
| G82016 | 1.143 | 0.96 | 0.96 | 2945 | 1388 | 1417 |
| B85018 | 1.143 | 1.22 | 1.21 | 2946 | 4480 | 4392 |
| C84016 | 1.143 | 1.31 | 1.33 | 2947 | 5553 | 5783 |
| N84023 | 1.143 | 1.30 | 1.28 | 2948 | 5480 | 5204 |
| L85050 | 1.142 | 1.12 | 1.09 | 2949 | 3109 | 2710 |
| M83141 | 1.142 | 1.14 | 1.15 | 2950 | 3333 | 3490 |
| G85726 | 1.142 | 0.99 | 0.99 | 2951 | 1617 | 1650 |
| M91604 | 1.142 | 1.27 | 1.27 | 2952 | 5144 | 5171 |
| K82049 | 1.142 | 1.04 | 1.02 | 2953 | 2118 | 1933 |
| F81092 | 1.142 | 1.04 | 1.05 | 2954 | 2096 | 2256 |
| L81087 | 1.142 | 1.04 | 1.05 | 2955 | 2142 | 2231 |
| F86064 | 1.142 | 0.97 | 0.96 | 2956 | 1494 | 1401 |
| B86051 | 1.142 | 1.14 | 1.16 | 2957 | 3344 | 3639 |
| P83620 | 1.142 | 1.07 | 1.08 | 2958 | 2508 | 2583 |
| F86696 | 1.142 | 0.97 | 0.99 | 2959 | 1465 | 1639 |
| A81056 | 1.142 | 1.18 | 1.18 | 2960 | 3934 | 3940 |
| N82009 | 1.141 | 1.06 | 1.08 | 2961 | 2340 | 2596 |
| B81068 | 1.141 | 1.15 | 1.14 | 2962 | 3521 | 3439 |
| Y02319 | 1.141 | 0.86 | 0.88 | 2963 | 775 | 828 |
| P82020 | 1.141 | 1.26 | 1.27 | 2964 | 5105 | 5190 |
| M84069 | 1.141 | 1.03 | 1.03 | 2965 | 2070 | 2025 |
| M83046 | 1.141 | 1.15 | 1.11 | 2966 | 3527 | 2992 |
| E87663 | 1.141 | 1.08 | 1.08 | 2967 | 2640 | 2598 |
| E82035 | 1.141 | 1.12 | 1.11 | 2968 | 3117 | 2934 |
| F85684 | 1.141 | 1.07 | 1.06 | 2969 | 2449 | 2357 |
| K83047 | 1.141 | 1.13 | 1.16 | 2970 | 3175 | 3751 |
| L83027 | 1.141 | 1.10 | 1.11 | 2971 | 2888 | 2943 |
| M84011 | 1.141 | 1.10 | 1.10 | 2972 | 2892 | 2903 |
| M88013 | 1.141 | 1.25 | 1.26 | 2973 | 4988 | 5074 |
| J82134 | 1.141 | 1.15 | 1.13 | 2974 | 3520 | 3245 |
| M85694 | 1.140 | 1.17 | 1.16 | 2975 | 3879 | 3724 |
| M87030 | 1.140 | 1.32 | 1.31 | 2976 | 5741 | 5605 |
| L84001 | 1.140 | 1.05 | 1.05 | 2977 | 2311 | 2218 |
| M87020 | 1.140 | 1.05 | 1.05 | 2978 | 2293 | 2339 |
| F82643 | 1.140 | 0.99 | 1.00 | 2979 | 1704 | 1708 |
| K81027 | 1.140 | 1.03 | 1.03 | 2980 | 2109 | 2071 |
| L85021 | 1.140 | 1.10 | 1.07 | 2981 | 2869 | 2495 |
| H81033 | 1.140 | 0.99 | 0.96 | 2982 | 1699 | 1433 |
| C81020 | 1.140 | 1.21 | 1.17 | 2983 | 4433 | 3862 |
| M83068 | 1.140 | 1.17 | 1.20 | 2984 | 3888 | 4345 |
| J82180 | 1.140 | 1.06 | 1.05 | 2985 | 2396 | 2225 |
| C81055 | 1.140 | 1.44 | 1.42 | 2986 | 6734 | 6590 |
| P81700 | 1.140 | 1.13 | 1.12 | 2987 | 3192 | 3153 |
| M84036 | 1.140 | 1.00 | 1.00 | 2988 | 1744 | 1744 |
| J81051 | 1.140 | 1.18 | 1.18 | 2989 | 4048 | 3988 |
| B83025 | 1.140 | 1.13 | 1.14 | 2990 | 3250 | 3473 |
| K81077 | 1.139 | 1.06 | 1.06 | 2991 | 2343 | 2356 |
| C84698 | 1.139 | 1.16 | 1.17 | 2992 | 3735 | 3909 |
| G82080 | 1.139 | 1.06 | 1.05 | 2993 | 2401 | 2341 |
| N81118 | 1.139 | 1.02 | 1.01 | 2994 | 2000 | 1865 |

| | | | | | | |
|---|---|---|---|---|---|---|
| M85663 | 1.139 | 0.99 | 0.98 | 2995 | 1665 | 1583 |
| F86006 | 1.139 | 0.97 | 0.98 | 2996 | 1447 | 1607 |
| D83076 | 1.139 | 1.08 | 1.07 | 2997 | 2590 | 2559 |
| M82043 | 1.139 | 1.12 | 1.12 | 2998 | 3144 | 3233 |
| Y02657 | 1.138 | 1.15 | 1.18 | 2999 | 3618 | 3973 |
| J81043 | 1.138 | 1.16 | 1.15 | 3000 | 3670 | 3539 |
| J81075 | 1.138 | 1.11 | 1.11 | 3001 | 3048 | 3061 |
| K83064 | 1.138 | 1.01 | 1.02 | 3002 | 1882 | 1952 |
| G83027 | 1.138 | 1.07 | 1.06 | 3003 | 2509 | 2394 |
| G84007 | 1.138 | 1.04 | 1.04 | 3004 | 2232 | 2168 |
| G82111 | 1.138 | 1.11 | 1.12 | 3005 | 2979 | 3195 |
| K84028 | 1.138 | 1.06 | 1.05 | 3006 | 2407 | 2359 |
| G83672 | 1.137 | 1.04 | 1.04 | 3007 | 2238 | 2161 |
| M83726 | 1.137 | 0.96 | 0.94 | 3008 | 1451 | 1280 |
| M83031 | 1.137 | 1.09 | 1.05 | 3009 | 2797 | 2358 |
| P81712 | 1.137 | 1.19 | 1.22 | 3010 | 4161 | 4549 |
| Y00542 | 1.137 | 1.06 | 1.05 | 3011 | 2451 | 2348 |
| G82763 | 1.137 | 1.03 | 1.06 | 3012 | 2148 | 2476 |
| B83037 | 1.137 | 1.20 | 1.21 | 3013 | 4262 | 4407 |
| C81017 | 1.137 | 1.20 | 1.20 | 3014 | 4312 | 4309 |
| J84004 | 1.137 | 1.10 | 1.10 | 3015 | 2909 | 2912 |
| K81036 | 1.137 | 1.03 | 1.01 | 3016 | 2124 | 1886 |
| N84043 | 1.137 | 1.12 | 1.11 | 3017 | 3191 | 3110 |
| M88002 | 1.137 | 1.23 | 1.25 | 3018 | 4767 | 4914 |
| B86034 | 1.137 | 1.06 | 1.08 | 3019 | 2366 | 2606 |
| B81113 | 1.136 | 1.25 | 1.27 | 3020 | 4951 | 5217 |
| C86029 | 1.136 | 1.13 | 1.16 | 3021 | 3368 | 3763 |
| C86009 | 1.136 | 1.16 | 1.18 | 3022 | 3696 | 4065 |
| E82630 | 1.136 | 0.95 | 0.93 | 3023 | 1374 | 1234 |
| C84036 | 1.136 | 1.13 | 1.15 | 3024 | 3255 | 3692 |
| A89009 | 1.136 | 1.20 | 1.21 | 3025 | 4285 | 4469 |
| J81054 | 1.136 | 1.03 | 1.04 | 3026 | 2146 | 2204 |
| N81079 | 1.136 | 1.05 | 1.03 | 3027 | 2337 | 2147 |
| M92015 | 1.136 | 1.20 | 1.22 | 3028 | 4322 | 4555 |
| L82066 | 1.136 | 1.19 | 1.19 | 3029 | 4160 | 4211 |
| C88054 | 1.136 | 1.10 | 1.12 | 3030 | 2932 | 3141 |
| B86059 | 1.135 | 1.06 | 1.08 | 3031 | 2434 | 2616 |
| C82094 | 1.135 | 1.02 | 1.02 | 3032 | 1959 | 1981 |
| M82005 | 1.135 | 1.06 | 1.06 | 3033 | 2399 | 2481 |
| A81009 | 1.135 | 1.09 | 1.11 | 3034 | 2870 | 3126 |
| F85035 | 1.135 | 1.01 | 1.01 | 3035 | 1905 | 1903 |
| N81030 | 1.135 | 1.10 | 1.07 | 3036 | 2876 | 2621 |
| L82012 | 1.135 | 1.13 | 1.14 | 3037 | 3355 | 3442 |
| A81002 | 1.135 | 1.26 | 1.26 | 3038 | 5138 | 5112 |
| M85016 | 1.135 | 1.20 | 1.18 | 3039 | 4325 | 4108 |
| F81044 | 1.134 | 1.08 | 1.09 | 3040 | 2666 | 2753 |
| D81623 | 1.134 | 1.07 | 1.07 | 3041 | 2607 | 2564 |
| M88018 | 1.134 | 1.17 | 1.18 | 3042 | 3952 | 4105 |
| M81033 | 1.134 | 1.05 | 1.02 | 3043 | 2312 | 2029 |
| D81082 | 1.134 | 1.06 | 1.05 | 3044 | 2505 | 2376 |

| | | | | | | |
|---|---|---|---|---|---|---|
| G81685 | 1.134 | 1.16 | 1.14 | 3045 | 3848 | 3471 |
| J81065 | 1.134 | 1.14 | 1.13 | 3046 | 3421 | 3371 |
| D81033 | 1.134 | 1.04 | 1.03 | 3047 | 2264 | 2136 |
| B87041 | 1.134 | 1.29 | 1.30 | 3048 | 5436 | 5563 |
| E81074 | 1.134 | 1.07 | 1.07 | 3049 | 2589 | 2557 |
| B86028 | 1.134 | 1.26 | 1.26 | 3050 | 5158 | 5177 |
| B81047 | 1.133 | 1.16 | 1.20 | 3051 | 3785 | 4374 |
| L85045 | 1.133 | 1.12 | 1.12 | 3052 | 3193 | 3205 |
| H82032 | 1.133 | 1.08 | 1.09 | 3053 | 2676 | 2855 |
| Y00159 | 1.133 | 1.21 | 1.20 | 3054 | 4491 | 4337 |
| D82010 | 1.133 | 1.33 | 1.33 | 3055 | 5886 | 5933 |
| H83048 | 1.133 | 1.13 | 1.12 | 3056 | 3304 | 3189 |
| P84022 | 1.133 | 1.17 | 1.19 | 3057 | 3989 | 4233 |
| C87007 | 1.133 | 1.10 | 1.12 | 3058 | 3000 | 3264 |
| J82020 | 1.133 | 1.02 | 1.01 | 3059 | 2067 | 1927 |
| G82092 | 1.133 | 0.98 | 0.97 | 3060 | 1642 | 1544 |
| A81033 | 1.133 | 1.41 | 1.41 | 3061 | 6585 | 6561 |
| G81037 | 1.133 | 0.98 | 0.97 | 3062 | 1662 | 1500 |
| H85021 | 1.133 | 1.02 | 1.01 | 3063 | 1992 | 1892 |
| J81052 | 1.133 | 1.21 | 1.21 | 3064 | 4551 | 4486 |
| E81062 | 1.133 | 1.11 | 1.10 | 3065 | 3077 | 3030 |
| P81006 | 1.132 | 1.11 | 1.11 | 3066 | 3024 | 3119 |
| K82058 | 1.132 | 1.06 | 1.03 | 3067 | 2438 | 2183 |
| M85056 | 1.132 | 1.14 | 1.12 | 3068 | 3543 | 3294 |
| J84018 | 1.132 | 1.21 | 1.21 | 3069 | 4557 | 4524 |
| M89024 | 1.132 | 1.12 | 1.08 | 3070 | 3157 | 2681 |
| B83620 | 1.132 | 1.13 | 1.15 | 3071 | 3441 | 3699 |
| M87023 | 1.132 | 1.11 | 1.11 | 3072 | 3099 | 3078 |
| F82663 | 1.132 | 0.96 | 0.96 | 3073 | 1475 | 1478 |
| L82046 | 1.132 | 1.05 | 1.05 | 3074 | 2350 | 2395 |
| G85013 | 1.132 | 0.93 | 0.93 | 3075 | 1245 | 1262 |
| M83009 | 1.132 | 1.10 | 1.07 | 3076 | 3013 | 2558 |
| P81780 | 1.132 | 1.23 | 1.26 | 3077 | 4842 | 5179 |
| L85011 | 1.132 | 1.09 | 1.09 | 3078 | 2807 | 2870 |
| M83097 | 1.132 | 1.08 | 1.04 | 3079 | 2724 | 2274 |
| K81017 | 1.132 | 1.05 | 1.05 | 3080 | 2416 | 2421 |
| D82060 | 1.131 | 1.13 | 1.13 | 3081 | 3367 | 3468 |
| G82035 | 1.131 | 1.10 | 1.11 | 3082 | 2984 | 3183 |
| K84007 | 1.131 | 1.04 | 1.03 | 3083 | 2245 | 2100 |
| G82124 | 1.131 | 1.19 | 1.16 | 3084 | 4235 | 3797 |
| K82024 | 1.131 | 1.04 | 1.02 | 3085 | 2269 | 1996 |
| F81131 | 1.131 | 1.08 | 1.05 | 3086 | 2711 | 2364 |
| L85007 | 1.131 | 1.15 | 1.16 | 3087 | 3737 | 3789 |
| N83003 | 1.131 | 1.40 | 1.40 | 3088 | 6553 | 6502 |
| G84013 | 1.131 | 1.06 | 1.05 | 3089 | 2502 | 2372 |
| P84644 | 1.131 | 1.04 | 1.06 | 3090 | 2262 | 2563 |
| M85123 | 1.131 | 1.14 | 1.14 | 3091 | 3586 | 3516 |
| G81011 | 1.130 | 1.07 | 1.08 | 3092 | 2571 | 2783 |
| N85054 | 1.130 | 1.08 | 1.08 | 3093 | 2796 | 2744 |
| P81646 | 1.130 | 1.14 | 1.15 | 3094 | 3494 | 3688 |

| | | | | | | |
|---|---|---|---|---|---|---|
| M84624 | 1.130 | 1.06 | 1.05 | 3095 | 2465 | 2415 |
| N82067 | 1.130 | 1.15 | 1.16 | 3096 | 3628 | 3821 |
| H83024 | 1.130 | 0.97 | 0.97 | 3097 | 1574 | 1537 |
| P86023 | 1.130 | 1.24 | 1.25 | 3098 | 4890 | 5072 |
| G81024 | 1.130 | 0.97 | 0.95 | 3099 | 1544 | 1422 |
| K82066 | 1.130 | 1.08 | 1.06 | 3100 | 2733 | 2512 |
| E85007 | 1.130 | 0.93 | 0.91 | 3101 | 1234 | 1124 |
| C84053 | 1.130 | 1.14 | 1.16 | 3102 | 3577 | 3878 |
| H82060 | 1.130 | 1.08 | 1.05 | 3103 | 2696 | 2422 |
| G82121 | 1.130 | 1.11 | 1.15 | 3104 | 3166 | 3706 |
| L81079 | 1.130 | 1.08 | 1.07 | 3105 | 2702 | 2592 |
| C83041 | 1.129 | 1.16 | 1.20 | 3106 | 3816 | 4450 |
| D83019 | 1.129 | 1.06 | 1.06 | 3107 | 2488 | 2580 |
| F81111 | 1.129 | 1.04 | 1.00 | 3108 | 2234 | 1895 |
| K82005 | 1.129 | 1.04 | 1.02 | 3109 | 2236 | 2058 |
| A81048 | 1.129 | 1.19 | 1.20 | 3110 | 4340 | 4422 |
| F81623 | 1.129 | 1.04 | 1.05 | 3111 | 2282 | 2375 |
| F81216 | 1.129 | 1.01 | 1.00 | 3112 | 2011 | 1842 |
| N81066 | 1.129 | 1.20 | 1.21 | 3113 | 4461 | 4620 |
| G81074 | 1.128 | 1.15 | 1.19 | 3114 | 3793 | 4255 |
| K83056 | 1.128 | 1.03 | 1.04 | 3115 | 2229 | 2349 |
| F86655 | 1.128 | 0.93 | 0.92 | 3116 | 1248 | 1170 |
| P85003 | 1.128 | 1.05 | 1.07 | 3117 | 2369 | 2660 |
| C88024 | 1.128 | 1.05 | 1.07 | 3118 | 2392 | 2666 |
| A86018 | 1.128 | 1.00 | 1.02 | 3119 | 1853 | 2030 |
| L83115 | 1.128 | 1.26 | 1.21 | 3120 | 5157 | 4609 |
| C85006 | 1.128 | 1.13 | 1.14 | 3121 | 3448 | 3656 |
| C84046 | 1.128 | 1.22 | 1.24 | 3122 | 4755 | 4902 |
| G82647 | 1.128 | 0.96 | 0.97 | 3123 | 1472 | 1545 |
| N85634 | 1.128 | 1.44 | 1.40 | 3124 | 6776 | 6529 |
| C83002 | 1.128 | 1.14 | 1.13 | 3125 | 3545 | 3492 |
| M83727 | 1.127 | 1.21 | 1.24 | 3126 | 4612 | 4966 |
| M86032 | 1.127 | 1.11 | 1.10 | 3127 | 3164 | 3020 |
| J82008 | 1.127 | 1.11 | 1.10 | 3128 | 3162 | 3018 |
| A89025 | 1.127 | 1.06 | 1.08 | 3129 | 2566 | 2728 |
| C82061 | 1.127 | 1.11 | 1.11 | 3130 | 3178 | 3230 |
| L82037 | 1.127 | 1.19 | 1.19 | 3131 | 4260 | 4284 |
| C88043 | 1.127 | 1.02 | 1.04 | 3132 | 2110 | 2322 |
| B81664 | 1.127 | 1.24 | 1.26 | 3133 | 5001 | 5155 |
| H85018 | 1.127 | 0.97 | 0.96 | 3134 | 1562 | 1506 |
| B82020 | 1.127 | 1.14 | 1.10 | 3135 | 3551 | 3001 |
| K82011 | 1.127 | 0.95 | 0.93 | 3136 | 1399 | 1284 |
| K84027 | 1.126 | 1.08 | 1.05 | 3137 | 2847 | 2380 |
| C85004 | 1.126 | 1.14 | 1.15 | 3138 | 3546 | 3818 |
| M88645 | 1.126 | 1.12 | 1.13 | 3139 | 3279 | 3503 |
| P82626 | 1.126 | 1.24 | 1.24 | 3140 | 4994 | 4999 |
| M86020 | 1.126 | 1.08 | 1.06 | 3141 | 2784 | 2594 |
| G82221 | 1.126 | 0.97 | 0.98 | 3142 | 1576 | 1671 |
| B87044 | 1.126 | 1.14 | 1.16 | 3143 | 3555 | 3916 |
| A82071 | 1.126 | 1.14 | 1.17 | 3144 | 3604 | 4032 |

| | | | | | | |
|---|---|---|---|---|---|---|
| Y02626 | 1.126 | 1.23 | 1.24 | 3145 | 4811 | 4995 |
| E82638 | 1.126 | 1.03 | 1.03 | 3146 | 2160 | 2159 |
| Y02807 | 1.126 | 0.87 | 0.88 | 3147 | 881 | 934 |
| J82184 | 1.126 | 1.11 | 1.10 | 3148 | 3150 | 3014 |
| B82059 | 1.126 | 1.14 | 1.13 | 3149 | 3597 | 3513 |
| P83023 | 1.125 | 1.08 | 1.08 | 3150 | 2781 | 2859 |
| E86014 | 1.125 | 0.94 | 0.94 | 3151 | 1400 | 1367 |
| J82206 | 1.125 | 0.96 | 0.93 | 3152 | 1495 | 1273 |
| G82688 | 1.125 | 0.96 | 0.96 | 3153 | 1545 | 1489 |
| B82069 | 1.125 | 1.07 | 1.07 | 3154 | 2739 | 2738 |
| P81185 | 1.125 | 1.06 | 1.05 | 3155 | 2549 | 2492 |
| M81034 | 1.125 | 1.12 | 1.11 | 3156 | 3389 | 3198 |
| B83038 | 1.125 | 1.19 | 1.20 | 3157 | 4272 | 4503 |
| P88009 | 1.125 | 1.33 | 1.31 | 3158 | 5945 | 5822 |
| B83008 | 1.125 | 1.17 | 1.17 | 3159 | 4010 | 4144 |
| L85046 | 1.125 | 1.24 | 1.20 | 3160 | 4956 | 4542 |
| M81039 | 1.125 | 1.07 | 1.04 | 3161 | 2647 | 2365 |
| N84004 | 1.125 | 1.17 | 1.17 | 3162 | 4086 | 4061 |
| C83033 | 1.124 | 1.26 | 1.28 | 3163 | 5206 | 5453 |
| B85008 | 1.124 | 1.09 | 1.09 | 3164 | 2944 | 2979 |
| J82104 | 1.124 | 1.12 | 1.11 | 3165 | 3330 | 3206 |
| M81001 | 1.124 | 1.16 | 1.19 | 3166 | 3996 | 4324 |
| C83062 | 1.124 | 1.21 | 1.20 | 3167 | 4603 | 4532 |
| J81010 | 1.124 | 1.26 | 1.21 | 3168 | 5221 | 4658 |
| B86077 | 1.124 | 1.15 | 1.16 | 3169 | 3761 | 3970 |
| H84630 | 1.124 | 0.91 | 0.90 | 3170 | 1174 | 1076 |
| F81159 | 1.124 | 1.03 | 1.04 | 3171 | 2202 | 2311 |
| M88639 | 1.124 | 1.31 | 1.33 | 3172 | 5795 | 5984 |
| P81078 | 1.124 | 1.15 | 1.18 | 3173 | 3767 | 4226 |
| C87004 | 1.124 | 1.27 | 1.27 | 3174 | 5337 | 5366 |
| P82034 | 1.124 | 1.10 | 1.12 | 3175 | 3112 | 3346 |
| E82019 | 1.124 | 1.02 | 1.03 | 3176 | 2127 | 2203 |
| N83032 | 1.124 | 1.34 | 1.33 | 3177 | 6036 | 6002 |
| F82031 | 1.124 | 1.03 | 1.04 | 3178 | 2246 | 2393 |
| Y00996 | 1.123 | 0.76 | 0.76 | 3179 | 474 | 464 |
| M82029 | 1.123 | 1.23 | 1.24 | 3180 | 4853 | 5011 |
| M81009 | 1.123 | 1.02 | 1.01 | 3181 | 2150 | 2008 |
| M83725 | 1.123 | 1.17 | 1.21 | 3182 | 4145 | 4594 |
| Y00912 | 1.123 | 1.16 | 1.15 | 3183 | 3881 | 3852 |
| N82035 | 1.123 | 1.15 | 1.17 | 3184 | 3875 | 4165 |
| F81012 | 1.123 | 1.12 | 1.12 | 3185 | 3343 | 3416 |
| N81092 | 1.123 | 1.11 | 1.09 | 3186 | 3227 | 3000 |
| M92609 | 1.123 | 1.16 | 1.18 | 3187 | 3994 | 4219 |
| B81048 | 1.122 | 1.02 | 1.06 | 3188 | 2130 | 2623 |
| C88008 | 1.122 | 1.10 | 1.12 | 3189 | 3134 | 3394 |
| C87015 | 1.122 | 1.19 | 1.20 | 3190 | 4412 | 4561 |
| M84007 | 1.122 | 1.15 | 1.10 | 3191 | 3782 | 3059 |
| D81034 | 1.122 | 1.14 | 1.13 | 3192 | 3708 | 3521 |
| B83657 | 1.122 | 0.83 | 0.85 | 3193 | 732 | 800 |
| M83693 | 1.122 | 0.94 | 0.94 | 3194 | 1408 | 1409 |

| | | | | | | |
|---|---|---|---|---|---|---|
| M83072 | 1.122 | 1.20 | 1.20 | 3195 | 4512 | 4498 |
| J82026 | 1.122 | 1.15 | 1.14 | 3196 | 3892 | 3720 |
| G83025 | 1.122 | 1.09 | 1.08 | 3197 | 3004 | 2857 |
| G82096 | 1.122 | 1.04 | 1.04 | 3198 | 2335 | 2405 |
| C88648 | 1.121 | 1.17 | 1.19 | 3199 | 4152 | 4351 |
| C82611 | 1.121 | 1.01 | 1.00 | 3200 | 2094 | 1910 |
| M84042 | 1.121 | 1.04 | 1.00 | 3201 | 2360 | 1936 |
| N82665 | 1.121 | 1.12 | 1.13 | 3202 | 3426 | 3565 |
| L83136 | 1.121 | 1.21 | 1.17 | 3203 | 4641 | 4193 |
| J82022 | 1.121 | 1.13 | 1.14 | 3204 | 3519 | 3645 |
| E81022 | 1.121 | 0.97 | 0.97 | 3205 | 1684 | 1663 |
| G82072 | 1.121 | 1.20 | 1.23 | 3206 | 4484 | 4935 |
| L81036 | 1.121 | 1.15 | 1.14 | 3207 | 3834 | 3647 |
| H82003 | 1.121 | 1.05 | 1.05 | 3208 | 2524 | 2450 |
| B83642 | 1.121 | 1.21 | 1.23 | 3209 | 4702 | 4942 |
| D82030 | 1.121 | 1.06 | 1.05 | 3210 | 2594 | 2479 |
| A83061 | 1.121 | 1.06 | 1.08 | 3211 | 2635 | 2810 |
| D83007 | 1.121 | 1.11 | 1.10 | 3212 | 3173 | 3146 |
| G85052 | 1.121 | 0.95 | 0.95 | 3213 | 1493 | 1503 |
| G82732 | 1.120 | 1.01 | 1.01 | 3214 | 2056 | 2069 |
| P83005 | 1.120 | 1.31 | 1.30 | 3215 | 5802 | 5723 |
| M92001 | 1.120 | 1.34 | 1.34 | 3216 | 6068 | 6113 |
| N82077 | 1.120 | 1.41 | 1.40 | 3217 | 6627 | 6613 |
| J82646 | 1.120 | 1.12 | 1.09 | 3218 | 3360 | 3050 |
| E85077 | 1.120 | 1.00 | 1.02 | 3219 | 1926 | 2173 |
| M92013 | 1.120 | 1.21 | 1.22 | 3220 | 4711 | 4832 |
| B82628 | 1.120 | 1.35 | 1.37 | 3221 | 6208 | 6366 |
| M85117 | 1.120 | 1.20 | 1.18 | 3222 | 4525 | 4269 |
| B83626 | 1.120 | 1.09 | 1.11 | 3223 | 3005 | 3251 |
| F81636 | 1.119 | 0.97 | 0.97 | 3224 | 1636 | 1694 |
| B84011 | 1.119 | 1.12 | 1.14 | 3225 | 3325 | 3670 |
| L83111 | 1.119 | 1.14 | 1.14 | 3226 | 3721 | 3788 |
| J82083 | 1.119 | 1.23 | 1.26 | 3227 | 4893 | 5313 |
| E81028 | 1.119 | 1.12 | 1.13 | 3228 | 3443 | 3613 |
| H81017 | 1.119 | 0.96 | 0.93 | 3229 | 1549 | 1362 |
| A83047 | 1.119 | 1.19 | 1.19 | 3230 | 4475 | 4477 |
| C83031 | 1.119 | 1.28 | 1.27 | 3231 | 5508 | 5448 |
| Y00025 | 1.119 | 1.17 | 1.18 | 3232 | 4110 | 4251 |
| D83001 | 1.119 | 1.10 | 1.07 | 3233 | 3082 | 2742 |
| L81024 | 1.119 | 1.10 | 1.09 | 3234 | 3070 | 3015 |
| B81647 | 1.119 | 1.18 | 1.20 | 3235 | 4245 | 4491 |
| L83054 | 1.119 | 1.32 | 1.28 | 3236 | 5946 | 5539 |
| G85129 | 1.119 | 0.90 | 0.92 | 3237 | 1158 | 1241 |
| M89019 | 1.119 | 1.06 | 1.03 | 3238 | 2687 | 2340 |
| H83053 | 1.119 | 1.01 | 1.00 | 3239 | 2022 | 1932 |
| C81028 | 1.119 | 1.09 | 1.08 | 3240 | 2971 | 2931 |
| B87040 | 1.119 | 1.17 | 1.19 | 3241 | 4146 | 4403 |
| E85117 | 1.119 | 1.11 | 1.08 | 3242 | 3237 | 2850 |
| J83017 | 1.119 | 1.06 | 1.05 | 3243 | 2614 | 2515 |
| E84083 | 1.118 | 1.04 | 1.02 | 3244 | 2370 | 2138 |

| | | | | | | |
|---|---|---|---|---|---|---|
| M88042 | 1.118 | 1.03 | 1.05 | 3245 | 2294 | 2486 |
| E83624 | 1.118 | 0.99 | 0.97 | 3246 | 1848 | 1647 |
| L83018 | 1.118 | 0.92 | 0.94 | 3247 | 1270 | 1431 |
| P84064 | 1.118 | 1.19 | 1.19 | 3248 | 4361 | 4457 |
| E81635 | 1.118 | 1.05 | 1.05 | 3249 | 2515 | 2485 |
| B85041 | 1.118 | 1.26 | 1.26 | 3250 | 5310 | 5320 |
| M84044 | 1.118 | 1.04 | 1.04 | 3251 | 2365 | 2433 |
| F86702 | 1.118 | 0.98 | 0.97 | 3252 | 1754 | 1645 |
| D83061 | 1.118 | 1.03 | 1.04 | 3253 | 2308 | 2419 |
| E82640 | 1.118 | 1.08 | 1.06 | 3254 | 2849 | 2688 |
| M81022 | 1.118 | 1.03 | 1.04 | 3255 | 2284 | 2436 |
| M83035 | 1.118 | 1.00 | 1.00 | 3256 | 1981 | 1992 |
| D83035 | 1.118 | 1.16 | 1.16 | 3257 | 4002 | 4051 |
| A83013 | 1.117 | 1.12 | 1.12 | 3258 | 3434 | 3483 |
| M91625 | 1.117 | 1.13 | 1.16 | 3259 | 3647 | 3997 |
| C83032 | 1.117 | 1.19 | 1.21 | 3260 | 4363 | 4702 |
| E86018 | 1.117 | 1.13 | 1.12 | 3261 | 3600 | 3437 |
| M87629 | 1.117 | 1.19 | 1.18 | 3262 | 4403 | 4312 |
| M91019 | 1.117 | 1.34 | 1.35 | 3263 | 6098 | 6198 |
| E84007 | 1.117 | 1.08 | 1.07 | 3264 | 2960 | 2752 |
| C84025 | 1.117 | 1.04 | 1.03 | 3265 | 2477 | 2258 |
| M87003 | 1.117 | 1.08 | 1.08 | 3266 | 2972 | 2956 |
| C83025 | 1.117 | 1.14 | 1.14 | 3267 | 3811 | 3746 |
| K84034 | 1.117 | 0.98 | 0.96 | 3268 | 1821 | 1606 |
| B81006 | 1.117 | 1.52 | 1.50 | 3269 | 7092 | 7037 |
| C87006 | 1.117 | 1.25 | 1.25 | 3270 | 5236 | 5249 |
| G82809 | 1.116 | 1.01 | 1.01 | 3271 | 2081 | 2102 |
| N81063 | 1.116 | 1.14 | 1.12 | 3272 | 3743 | 3413 |
| H82034 | 1.116 | 1.15 | 1.15 | 3273 | 3858 | 3846 |
| B81023 | 1.116 | 1.19 | 1.21 | 3274 | 4405 | 4772 |
| P81107 | 1.116 | 1.09 | 1.07 | 3275 | 3010 | 2801 |
| Y00155 | 1.116 | 0.99 | 0.97 | 3276 | 1846 | 1715 |
| B87039 | 1.116 | 1.14 | 1.15 | 3277 | 3713 | 3978 |
| M92003 | 1.116 | 1.30 | 1.30 | 3278 | 5722 | 5747 |
| L83107 | 1.116 | 1.24 | 1.24 | 3279 | 5074 | 5147 |
| C88018 | 1.116 | 1.07 | 1.08 | 3280 | 2793 | 2974 |
| P87610 | 1.116 | 1.16 | 1.19 | 3281 | 4056 | 4400 |
| B83002 | 1.116 | 1.00 | 1.02 | 3282 | 2014 | 2213 |
| J82133 | 1.116 | 1.22 | 1.25 | 3283 | 4926 | 5281 |
| H82008 | 1.115 | 1.02 | 1.02 | 3284 | 2243 | 2200 |
| L85607 | 1.115 | 1.06 | 1.06 | 3285 | 2659 | 2690 |
| M87621 | 1.115 | 1.08 | 1.07 | 3286 | 2940 | 2865 |
| M81003 | 1.115 | 1.14 | 1.13 | 3287 | 3779 | 3612 |
| G84627 | 1.115 | 0.99 | 0.98 | 3288 | 1877 | 1826 |
| M83669 | 1.115 | 1.15 | 1.19 | 3289 | 3977 | 4460 |
| G82056 | 1.115 | 1.04 | 1.05 | 3290 | 2382 | 2523 |
| F85026 | 1.115 | 1.16 | 1.16 | 3291 | 4033 | 4000 |
| Y00411 | 1.115 | 1.36 | 1.35 | 3292 | 6293 | 6213 |
| M81608 | 1.115 | 1.19 | 1.17 | 3293 | 4536 | 4249 |
| N81029 | 1.115 | 1.14 | 1.12 | 3294 | 3742 | 3466 |

| | | | | | | |
|---|---|---|---|---|---|---|
| M83126 | 1.115 | 1.18 | 1.22 | 3295 | 4276 | 4874 |
| D83058 | 1.115 | 1.10 | 1.11 | 3296 | 3143 | 3320 |
| B83014 | 1.115 | 1.08 | 1.09 | 3297 | 2901 | 3098 |
| A81046 | 1.115 | 1.16 | 1.16 | 3298 | 4044 | 4136 |
| H84062 | 1.114 | 0.96 | 0.95 | 3299 | 1660 | 1533 |
| K81020 | 1.114 | 0.99 | 0.97 | 3300 | 1895 | 1747 |
| A81001 | 1.114 | 1.24 | 1.24 | 3301 | 5087 | 5095 |
| H82042 | 1.114 | 1.11 | 1.12 | 3302 | 3342 | 3497 |
| J82113 | 1.114 | 1.06 | 1.10 | 3303 | 2749 | 3199 |
| B86107 | 1.114 | 1.09 | 1.12 | 3304 | 3105 | 3459 |
| F81009 | 1.114 | 1.04 | 1.01 | 3305 | 2397 | 2064 |
| E85727 | 1.114 | 1.02 | 1.01 | 3306 | 2203 | 2106 |
| J83029 | 1.114 | 1.14 | 1.13 | 3307 | 3825 | 3609 |
| P85016 | 1.114 | 1.05 | 1.07 | 3308 | 2595 | 2879 |
| B85606 | 1.113 | 1.31 | 1.31 | 3309 | 5847 | 5848 |
| N81082 | 1.113 | 1.14 | 1.11 | 3310 | 3756 | 3407 |
| J82166 | 1.113 | 1.17 | 1.16 | 3311 | 4218 | 4063 |
| M84034 | 1.113 | 0.99 | 0.99 | 3312 | 1916 | 1946 |
| L85006 | 1.113 | 1.09 | 1.06 | 3313 | 3071 | 2703 |
| J83045 | 1.113 | 1.04 | 1.03 | 3314 | 2403 | 2319 |
| A88001 | 1.113 | 1.27 | 1.25 | 3315 | 5453 | 5274 |
| K84047 | 1.113 | 1.07 | 1.04 | 3316 | 2834 | 2501 |
| P83024 | 1.113 | 1.22 | 1.22 | 3317 | 4872 | 4881 |
| L84009 | 1.113 | 1.02 | 1.02 | 3318 | 2220 | 2188 |
| K82048 | 1.113 | 1.13 | 1.11 | 3319 | 3734 | 3332 |
| C84011 | 1.113 | 1.09 | 1.11 | 3320 | 3075 | 3403 |
| H85682 | 1.113 | 0.98 | 0.95 | 3321 | 1780 | 1563 |
| G83642 | 1.113 | 1.02 | 1.01 | 3322 | 2196 | 2095 |
| B86666 | 1.113 | 1.27 | 1.30 | 3323 | 5522 | 5772 |
| J82053 | 1.112 | 1.13 | 1.11 | 3324 | 3654 | 3370 |
| Y02838 | 1.112 | 1.01 | 1.03 | 3325 | 2176 | 2307 |
| B81014 | 1.112 | 1.40 | 1.39 | 3326 | 6657 | 6570 |
| F81119 | 1.112 | 1.03 | 1.03 | 3327 | 2406 | 2430 |
| M85164 | 1.112 | 1.13 | 1.13 | 3328 | 3674 | 3614 |
| B85634 | 1.112 | 1.22 | 1.22 | 3329 | 4900 | 4960 |
| F81142 | 1.112 | 1.13 | 1.13 | 3330 | 3691 | 3622 |
| M83125 | 1.112 | 1.13 | 1.13 | 3331 | 3746 | 3743 |
| J82139 | 1.112 | 1.08 | 1.07 | 3332 | 3002 | 2873 |
| P81689 | 1.112 | 1.19 | 1.15 | 3333 | 4489 | 4028 |
| B86067 | 1.112 | 1.06 | 1.08 | 3334 | 2727 | 2964 |
| M87008 | 1.112 | 1.08 | 1.08 | 3335 | 2997 | 3008 |
| P83009 | 1.112 | 1.09 | 1.10 | 3336 | 3135 | 3289 |
| H81005 | 1.112 | 1.06 | 1.03 | 3337 | 2740 | 2409 |
| P81096 | 1.112 | 1.11 | 1.12 | 3338 | 3337 | 3532 |
| N82669 | 1.112 | 1.38 | 1.37 | 3339 | 6494 | 6440 |
| B85004 | 1.112 | 1.25 | 1.24 | 3340 | 5266 | 5178 |
| E86609 | 1.112 | 1.01 | 1.01 | 3341 | 2165 | 2112 |
| D82063 | 1.112 | 1.21 | 1.19 | 3342 | 4774 | 4509 |
| M88633 | 1.112 | 1.15 | 1.17 | 3343 | 3954 | 4215 |
| H84030 | 1.111 | 0.95 | 0.94 | 3344 | 1536 | 1453 |

| | | | | | | |
|---|---|---|---|---|---|---|
| M89010 | 1.111 | 0.99 | 0.97 | 3345 | 1975 | 1740 |
| F84642 | 1.111 | 0.86 | 0.85 | 3346 | 930 | 883 |
| M84047 | 1.111 | 1.04 | 1.04 | 3347 | 2522 | 2519 |
| G82037 | 1.111 | 0.99 | 0.99 | 3348 | 1991 | 1906 |
| C82080 | 1.111 | 1.23 | 1.24 | 3349 | 5026 | 5089 |
| P81772 | 1.111 | 1.17 | 1.18 | 3350 | 4296 | 4406 |
| Y00918 | 1.111 | 1.13 | 1.11 | 3351 | 3649 | 3465 |
| F81099 | 1.111 | 1.04 | 1.03 | 3352 | 2532 | 2396 |
| E83037 | 1.111 | 1.07 | 1.04 | 3353 | 2821 | 2554 |
| A87019 | 1.111 | 1.18 | 1.19 | 3354 | 4321 | 4525 |
| K83012 | 1.111 | 0.99 | 1.01 | 3355 | 1986 | 2139 |
| L81062 | 1.111 | 1.03 | 1.04 | 3356 | 2380 | 2484 |
| C84131 | 1.111 | 1.16 | 1.16 | 3357 | 4166 | 4079 |
| C88040 | 1.110 | 1.08 | 1.10 | 3358 | 3003 | 3266 |
| J81090 | 1.110 | 1.29 | 1.27 | 3359 | 5708 | 5555 |
| B82028 | 1.110 | 1.20 | 1.16 | 3360 | 4726 | 4207 |
| J81038 | 1.110 | 1.21 | 1.20 | 3361 | 4752 | 4665 |
| A88016 | 1.110 | 1.16 | 1.16 | 3362 | 4124 | 4187 |
| F84050 | 1.110 | 0.99 | 0.98 | 3363 | 1945 | 1828 |
| E84702 | 1.110 | 1.05 | 1.04 | 3364 | 2629 | 2468 |
| G81040 | 1.110 | 1.02 | 0.99 | 3365 | 2265 | 1984 |
| H82014 | 1.110 | 1.12 | 1.12 | 3366 | 3621 | 3494 |
| M86046 | 1.110 | 1.14 | 1.14 | 3367 | 3913 | 3798 |
| M91613 | 1.110 | 1.11 | 1.13 | 3368 | 3381 | 3745 |
| F84739 | 1.110 | 1.00 | 0.99 | 3369 | 2076 | 1948 |
| B85055 | 1.109 | 1.12 | 1.13 | 3370 | 3558 | 3714 |
| M86013 | 1.109 | 1.07 | 1.06 | 3371 | 2874 | 2763 |
| C82055 | 1.109 | 1.02 | 1.02 | 3372 | 2302 | 2296 |
| H82029 | 1.109 | 1.09 | 1.10 | 3373 | 3130 | 3359 |
| P85001 | 1.109 | 1.13 | 1.15 | 3374 | 3672 | 3986 |
| K81026 | 1.109 | 0.99 | 0.99 | 3375 | 1911 | 1954 |
| H85009 | 1.109 | 0.86 | 0.85 | 3376 | 950 | 843 |
| F81088 | 1.109 | 1.13 | 1.14 | 3377 | 3748 | 3854 |
| C82663 | 1.109 | 1.12 | 1.11 | 3378 | 3500 | 3484 |
| K83031 | 1.109 | 1.05 | 1.06 | 3379 | 2673 | 2730 |
| C83022 | 1.109 | 1.14 | 1.14 | 3380 | 3871 | 3834 |
| C84683 | 1.109 | 1.10 | 1.11 | 3381 | 3197 | 3449 |
| K83066 | 1.109 | 1.15 | 1.15 | 3382 | 4016 | 4040 |
| B81077 | 1.109 | 1.12 | 1.15 | 3383 | 3554 | 3999 |
| H81095 | 1.109 | 1.00 | 0.98 | 3384 | 2047 | 1861 |
| N81093 | 1.109 | 1.16 | 1.13 | 3385 | 4142 | 3808 |
| M81005 | 1.108 | 1.18 | 1.15 | 3386 | 4388 | 4082 |
| C82001 | 1.108 | 1.00 | 0.99 | 3387 | 2135 | 1972 |
| N81047 | 1.108 | 1.07 | 1.06 | 3388 | 2890 | 2714 |
| M92011 | 1.108 | 1.05 | 1.07 | 3389 | 2626 | 2856 |
| Y00050 | 1.108 | 1.11 | 1.11 | 3390 | 3429 | 3500 |
| C87008 | 1.108 | 1.17 | 1.18 | 3391 | 4253 | 4437 |
| B86096 | 1.108 | 1.26 | 1.26 | 3392 | 5462 | 5449 |
| G82224 | 1.108 | 0.94 | 0.94 | 3393 | 1491 | 1498 |
| M91614 | 1.108 | 1.21 | 1.21 | 3394 | 4780 | 4856 |

| | | | | | | |
|---|---|---|---|---|---|---|
| M83075 | 1.108 | 1.08 | 1.11 | 3395 | 2983 | 3454 |
| M91647 | 1.107 | 1.32 | 1.33 | 3396 | 6066 | 6136 |
| B81017 | 1.107 | 1.18 | 1.22 | 3397 | 4423 | 4949 |
| G84003 | 1.107 | 1.06 | 1.05 | 3398 | 2746 | 2627 |
| F85632 | 1.107 | 1.06 | 1.07 | 3399 | 2769 | 2906 |
| K82053 | 1.107 | 1.04 | 1.03 | 3400 | 2596 | 2383 |
| B84003 | 1.107 | 1.06 | 1.08 | 3401 | 2752 | 3021 |
| A89038 | 1.107 | 1.11 | 1.12 | 3402 | 3511 | 3689 |
| J82609 | 1.107 | 1.14 | 1.13 | 3403 | 3938 | 3734 |
| A81025 | 1.107 | 1.16 | 1.17 | 3404 | 4178 | 4278 |
| M82057 | 1.107 | 1.01 | 1.02 | 3405 | 2184 | 2320 |
| F81001 | 1.107 | 1.16 | 1.16 | 3406 | 4219 | 4197 |
| G82059 | 1.107 | 0.98 | 0.97 | 3407 | 1892 | 1824 |
| C82107 | 1.107 | 1.21 | 1.22 | 3408 | 4858 | 4947 |
| J81049 | 1.107 | 1.10 | 1.10 | 3409 | 3301 | 3366 |
| N81042 | 1.107 | 1.07 | 1.06 | 3410 | 2887 | 2727 |
| A82060 | 1.107 | 1.22 | 1.24 | 3411 | 5024 | 5259 |
| C82103 | 1.107 | 1.15 | 1.14 | 3412 | 3973 | 3942 |
| L83014 | 1.107 | 1.17 | 1.17 | 3413 | 4307 | 4383 |
| L83038 | 1.107 | 1.04 | 1.03 | 3414 | 2550 | 2398 |
| M86638 | 1.106 | 1.14 | 1.13 | 3415 | 3869 | 3702 |
| L84075 | 1.106 | 1.22 | 1.21 | 3416 | 5018 | 4912 |
| D83084 | 1.106 | 1.04 | 1.05 | 3417 | 2615 | 2695 |
| P81073 | 1.106 | 1.44 | 1.48 | 3418 | 6863 | 7013 |
| L85039 | 1.106 | 1.10 | 1.08 | 3419 | 3296 | 3074 |
| D82005 | 1.106 | 1.41 | 1.37 | 3420 | 6709 | 6472 |
| C82036 | 1.106 | 1.01 | 0.99 | 3421 | 2172 | 2017 |
| H82043 | 1.106 | 1.35 | 1.33 | 3422 | 6282 | 6116 |
| M89013 | 1.106 | 1.20 | 1.15 | 3423 | 4669 | 4076 |
| B81056 | 1.106 | 1.11 | 1.16 | 3424 | 3409 | 4149 |
| P91017 | 1.106 | 1.05 | 1.06 | 3425 | 2707 | 2791 |
| N85015 | 1.106 | 1.11 | 1.10 | 3426 | 3495 | 3343 |
| L84059 | 1.105 | 1.04 | 1.05 | 3427 | 2612 | 2689 |
| H81026 | 1.105 | 1.01 | 1.01 | 3428 | 2254 | 2228 |
| L81068 | 1.105 | 0.93 | 0.93 | 3429 | 1440 | 1423 |
| H83039 | 1.105 | 1.05 | 1.04 | 3430 | 2685 | 2567 |
| D82057 | 1.105 | 1.45 | 1.42 | 3431 | 6910 | 6780 |
| Y01795 | 1.105 | 0.95 | 0.96 | 3432 | 1594 | 1687 |
| B81666 | 1.105 | 0.94 | 0.95 | 3433 | 1546 | 1596 |
| P81172 | 1.105 | 1.15 | 1.19 | 3434 | 4053 | 4623 |
| G82125 | 1.105 | 1.03 | 1.01 | 3435 | 2388 | 2271 |
| D83064 | 1.105 | 1.19 | 1.15 | 3436 | 4561 | 4091 |
| B83050 | 1.105 | 1.25 | 1.25 | 3437 | 5345 | 5342 |
| P88023 | 1.105 | 1.11 | 1.11 | 3438 | 3410 | 3462 |
| K81656 | 1.104 | 0.95 | 0.95 | 3439 | 1607 | 1595 |
| P81076 | 1.104 | 1.16 | 1.15 | 3440 | 4143 | 4050 |
| L82045 | 1.104 | 1.10 | 1.11 | 3441 | 3387 | 3486 |
| K82055 | 1.104 | 1.10 | 1.07 | 3442 | 3269 | 2963 |
| H81613 | 1.104 | 0.93 | 0.91 | 3443 | 1459 | 1286 |
| F81078 | 1.104 | 0.92 | 0.94 | 3444 | 1353 | 1540 |

| | | | | | | |
|---|---|---|---|---|---|---|
| J82079 | 1.104 | 1.03 | 1.01 | 3445 | 2447 | 2246 |
| P81058 | 1.104 | 1.22 | 1.25 | 3446 | 5037 | 5314 |
| B82101 | 1.104 | 1.18 | 1.20 | 3447 | 4467 | 4721 |
| K83029 | 1.104 | 1.04 | 1.06 | 3448 | 2611 | 2761 |
| J82142 | 1.103 | 1.01 | 1.01 | 3449 | 2270 | 2214 |
| D81633 | 1.103 | 1.01 | 1.01 | 3450 | 2273 | 2210 |
| M88016 | 1.103 | 1.42 | 1.41 | 3451 | 6806 | 6744 |
| B84004 | 1.103 | 1.05 | 1.07 | 3452 | 2699 | 2987 |
| G82228 | 1.103 | 1.10 | 1.11 | 3453 | 3354 | 3474 |
| K82042 | 1.103 | 1.07 | 1.05 | 3454 | 3001 | 2671 |
| H84608 | 1.103 | 0.93 | 0.91 | 3455 | 1492 | 1345 |
| J81046 | 1.103 | 1.12 | 1.12 | 3456 | 3603 | 3696 |
| Y02597 | 1.103 | 1.13 | 1.16 | 3457 | 3747 | 4265 |
| J81005 | 1.103 | 1.19 | 1.17 | 3458 | 4691 | 4343 |
| M83713 | 1.103 | 1.31 | 1.34 | 3459 | 5956 | 6260 |
| K83027 | 1.103 | 1.03 | 1.05 | 3460 | 2516 | 2655 |
| K83620 | 1.103 | 1.11 | 1.07 | 3461 | 3438 | 2967 |
| F81075 | 1.102 | 1.12 | 1.11 | 3462 | 3611 | 3588 |
| J83060 | 1.102 | 1.17 | 1.16 | 3463 | 4341 | 4212 |
| F81215 | 1.102 | 0.99 | 0.94 | 3464 | 1985 | 1577 |
| M83613 | 1.102 | 1.12 | 1.08 | 3465 | 3704 | 3093 |
| P92020 | 1.102 | 1.23 | 1.23 | 3466 | 5128 | 5125 |
| C86606 | 1.102 | 1.15 | 1.17 | 3467 | 4138 | 4405 |
| G81023 | 1.102 | 1.12 | 1.08 | 3468 | 3745 | 3159 |
| N83043 | 1.102 | 1.39 | 1.37 | 3469 | 6633 | 6546 |
| E86029 | 1.102 | 1.01 | 1.00 | 3470 | 2227 | 2137 |
| B83015 | 1.102 | 1.22 | 1.22 | 3471 | 5007 | 5046 |
| M83047 | 1.102 | 1.15 | 1.19 | 3472 | 4144 | 4683 |
| C83054 | 1.102 | 1.13 | 1.11 | 3473 | 3795 | 3570 |
| P87039 | 1.101 | 1.28 | 1.30 | 3474 | 5712 | 5932 |
| F84679 | 1.101 | 1.10 | 1.09 | 3475 | 3327 | 3221 |
| E82048 | 1.101 | 1.04 | 1.04 | 3476 | 2581 | 2607 |
| P88034 | 1.101 | 1.19 | 1.18 | 3477 | 4651 | 4565 |
| C82009 | 1.101 | 1.09 | 1.07 | 3478 | 3247 | 3031 |
| C83014 | 1.101 | 1.19 | 1.23 | 3479 | 4661 | 5101 |
| K81042 | 1.101 | 1.03 | 1.01 | 3480 | 2444 | 2283 |
| M91602 | 1.101 | 1.31 | 1.31 | 3481 | 5999 | 5991 |
| G81085 | 1.101 | 1.15 | 1.10 | 3482 | 4095 | 3423 |
| Y02887 | 1.101 | 0.86 | 0.88 | 3483 | 1023 | 1120 |
| F81665 | 1.101 | 0.94 | 0.94 | 3484 | 1533 | 1560 |
| F84043 | 1.101 | 1.07 | 1.08 | 3485 | 3015 | 3196 |
| M86015 | 1.100 | 1.10 | 1.09 | 3486 | 3351 | 3253 |
| C82003 | 1.100 | 0.99 | 1.00 | 3487 | 2041 | 2149 |
| C82033 | 1.100 | 1.14 | 1.14 | 3488 | 3939 | 3939 |
| F81704 | 1.100 | 1.00 | 0.98 | 3489 | 2140 | 1916 |
| A82621 | 1.100 | 1.05 | 1.08 | 3490 | 2805 | 3179 |
| P81013 | 1.100 | 1.17 | 1.18 | 3491 | 4432 | 4552 |
| D83054 | 1.100 | 1.13 | 1.13 | 3492 | 3787 | 3865 |
| C82007 | 1.100 | 1.10 | 1.10 | 3493 | 3472 | 3405 |
| E81009 | 1.100 | 1.09 | 1.08 | 3494 | 3229 | 3170 |

| | | | | | | |
|---|---|---|---|---|---|---|
| G81017 | 1.100 | 1.11 | 1.11 | 3495 | 3490 | 3629 |
| K83070 | 1.100 | 1.08 | 1.05 | 3496 | 3165 | 2746 |
| N83635 | 1.100 | 1.07 | 1.09 | 3497 | 3054 | 3269 |
| K82022 | 1.100 | 1.01 | 0.99 | 3498 | 2315 | 2083 |
| M83034 | 1.100 | 1.29 | 1.30 | 3499 | 5857 | 5913 |
| P88002 | 1.099 | 1.08 | 1.08 | 3500 | 3111 | 3217 |
| E84708 | 1.099 | 1.03 | 1.02 | 3501 | 2541 | 2384 |
| J84015 | 1.099 | 1.02 | 1.03 | 3502 | 2428 | 2503 |
| P88632 | 1.099 | 1.12 | 1.11 | 3503 | 3682 | 3601 |
| F81632 | 1.099 | 1.15 | 1.14 | 3504 | 4107 | 3990 |
| G82200 | 1.099 | 1.01 | 1.00 | 3505 | 2268 | 2157 |
| A82045 | 1.099 | 1.24 | 1.25 | 3506 | 5309 | 5360 |
| L81122 | 1.099 | 1.09 | 1.08 | 3507 | 3291 | 3193 |
| L83096 | 1.099 | 1.16 | 1.15 | 3508 | 4284 | 4129 |
| F84032 | 1.099 | 0.97 | 0.96 | 3509 | 1894 | 1800 |
| C88037 | 1.099 | 1.13 | 1.14 | 3510 | 3844 | 4057 |
| H82615 | 1.098 | 1.02 | 1.02 | 3511 | 2363 | 2413 |
| P89025 | 1.098 | 1.12 | 1.14 | 3512 | 3705 | 4047 |
| C81029 | 1.098 | 1.24 | 1.24 | 3513 | 5356 | 5272 |
| B83622 | 1.098 | 1.01 | 1.03 | 3514 | 2319 | 2522 |
| K83011 | 1.098 | 1.12 | 1.15 | 3515 | 3684 | 4227 |
| B81112 | 1.098 | 1.09 | 1.13 | 3516 | 3287 | 3946 |
| K81616 | 1.098 | 1.21 | 1.22 | 3517 | 4952 | 5110 |
| L84616 | 1.098 | 1.04 | 1.05 | 3518 | 2644 | 2735 |
| H85038 | 1.098 | 1.05 | 1.03 | 3519 | 2839 | 2565 |
| G81103 | 1.098 | 1.02 | 1.04 | 3520 | 2414 | 2631 |
| P87027 | 1.098 | 1.03 | 1.06 | 3521 | 2576 | 2851 |
| B81055 | 1.098 | 1.21 | 1.24 | 3522 | 4985 | 5293 |
| P81774 | 1.098 | 1.28 | 1.27 | 3523 | 5699 | 5685 |
| J82007 | 1.098 | 1.15 | 1.14 | 3524 | 4186 | 4014 |
| J82214 | 1.098 | 1.10 | 1.08 | 3525 | 3365 | 3155 |
| A82048 | 1.097 | 1.15 | 1.15 | 3526 | 4159 | 4240 |
| G81036 | 1.097 | 1.00 | 1.02 | 3527 | 2169 | 2374 |
| G83004 | 1.097 | 1.04 | 1.03 | 3528 | 2661 | 2551 |
| L82068 | 1.097 | 1.14 | 1.14 | 3529 | 4004 | 4053 |
| B83010 | 1.097 | 1.24 | 1.25 | 3530 | 5298 | 5383 |
| J81027 | 1.097 | 1.19 | 1.19 | 3531 | 4722 | 4687 |
| D82084 | 1.097 | 1.12 | 1.11 | 3532 | 3757 | 3587 |
| J83037 | 1.097 | 0.99 | 0.98 | 3533 | 2037 | 1967 |
| N84018 | 1.097 | 1.13 | 1.13 | 3534 | 3915 | 3965 |
| H81664 | 1.097 | 0.91 | 0.92 | 3535 | 1360 | 1411 |
| N82037 | 1.097 | 1.18 | 1.20 | 3536 | 4594 | 4867 |
| M86030 | 1.097 | 1.00 | 0.99 | 3537 | 2154 | 2098 |
| C88041 | 1.097 | 1.05 | 1.07 | 3538 | 2804 | 3025 |
| C82102 | 1.096 | 1.07 | 1.07 | 3539 | 3087 | 3080 |
| B82074 | 1.096 | 1.15 | 1.13 | 3540 | 4199 | 3900 |
| G81663 | 1.096 | 0.98 | 1.01 | 3541 | 2034 | 2270 |
| N82004 | 1.096 | 1.18 | 1.17 | 3542 | 4539 | 4515 |
| G82020 | 1.096 | 1.18 | 1.19 | 3543 | 4583 | 4766 |
| P83609 | 1.096 | 1.07 | 1.08 | 3544 | 3057 | 3148 |

| | | | | | | |
|---|---|---|---|---|---|---|
| F82015 | 1.096 | 1.03 | 1.04 | 3545 | 2628 | 2657 |
| L83004 | 1.096 | 1.11 | 1.11 | 3546 | 3693 | 3634 |
| M83127 | 1.095 | 1.09 | 1.13 | 3547 | 3358 | 3894 |
| L81008 | 1.095 | 1.09 | 1.09 | 3548 | 3259 | 3362 |
| C84092 | 1.095 | 1.11 | 1.13 | 3549 | 3629 | 3926 |
| P85017 | 1.095 | 1.21 | 1.22 | 3550 | 4938 | 5135 |
| C84091 | 1.095 | 1.15 | 1.16 | 3551 | 4182 | 4402 |
| J81068 | 1.095 | 1.14 | 1.11 | 3552 | 4047 | 3661 |
| H81054 | 1.095 | 1.07 | 1.04 | 3553 | 3044 | 2642 |
| Y03663 | 1.095 | 0.94 | 0.92 | 3554 | 1624 | 1469 |
| M85070 | 1.094 | 1.08 | 1.07 | 3555 | 3220 | 3082 |
| K83026 | 1.094 | 1.07 | 1.10 | 3556 | 3041 | 3563 |
| L85012 | 1.094 | 1.09 | 1.06 | 3557 | 3303 | 2949 |
| D83003 | 1.094 | 1.12 | 1.12 | 3558 | 3849 | 3761 |
| F81214 | 1.094 | 1.12 | 1.12 | 3559 | 3789 | 3853 |
| L83019 | 1.094 | 1.23 | 1.24 | 3560 | 5204 | 5414 |
| M92044 | 1.094 | 1.22 | 1.24 | 3561 | 5180 | 5355 |
| F81027 | 1.094 | 1.11 | 1.12 | 3562 | 3576 | 3849 |
| L84047 | 1.094 | 1.07 | 1.03 | 3563 | 3060 | 2624 |
| M89004 | 1.094 | 1.06 | 1.03 | 3564 | 2969 | 2578 |
| D83619 | 1.094 | 1.12 | 1.13 | 3565 | 3837 | 3884 |
| N85048 | 1.094 | 1.14 | 1.14 | 3566 | 4114 | 4016 |
| B81109 | 1.094 | 1.17 | 1.19 | 3567 | 4449 | 4750 |
| M85030 | 1.094 | 1.10 | 1.09 | 3568 | 3579 | 3418 |
| P81150 | 1.094 | 1.18 | 1.15 | 3569 | 4659 | 4274 |
| P84676 | 1.093 | 0.96 | 0.99 | 3570 | 1866 | 2099 |
| Y02812 | 1.093 | 0.81 | 0.81 | 3571 | 745 | 732 |
| E81046 | 1.093 | 1.04 | 1.04 | 3572 | 2719 | 2672 |
| C84150 | 1.093 | 1.11 | 1.10 | 3573 | 3731 | 3469 |
| G84025 | 1.093 | 1.00 | 0.99 | 3574 | 2193 | 2090 |
| L83053 | 1.093 | 1.16 | 1.13 | 3575 | 4310 | 3908 |
| B83627 | 1.093 | 1.11 | 1.12 | 3576 | 3668 | 3880 |
| G82679 | 1.092 | 1.05 | 1.08 | 3577 | 2881 | 3216 |
| D82027 | 1.092 | 1.32 | 1.32 | 3578 | 6162 | 6151 |
| K82028 | 1.092 | 1.02 | 0.99 | 3579 | 2430 | 2181 |
| N82076 | 1.092 | 1.09 | 1.10 | 3580 | 3314 | 3561 |
| M82060 | 1.092 | 1.04 | 1.05 | 3581 | 2741 | 2833 |
| F81095 | 1.092 | 1.10 | 1.10 | 3582 | 3585 | 3592 |
| J83009 | 1.092 | 1.14 | 1.15 | 3583 | 4111 | 4239 |
| M91033 | 1.092 | 1.12 | 1.13 | 3584 | 3835 | 4036 |
| G83053 | 1.092 | 1.05 | 1.04 | 3585 | 2891 | 2762 |
| D82050 | 1.092 | 1.10 | 1.09 | 3586 | 3542 | 3457 |
| M83051 | 1.092 | 1.14 | 1.10 | 3587 | 4090 | 3549 |
| M92008 | 1.092 | 1.10 | 1.11 | 3588 | 3501 | 3750 |
| C82058 | 1.092 | 1.18 | 1.18 | 3589 | 4610 | 4602 |
| B83017 | 1.091 | 1.16 | 1.17 | 3590 | 4319 | 4480 |
| E81044 | 1.091 | 1.02 | 1.02 | 3591 | 2546 | 2510 |
| L83005 | 1.091 | 1.22 | 1.21 | 3592 | 5151 | 4989 |
| G85055 | 1.091 | 0.95 | 0.97 | 3593 | 1772 | 1894 |
| M87011 | 1.091 | 1.10 | 1.10 | 3594 | 3541 | 3519 |

| | | | | | | |
|---|---|---|---|---|---|---|
| A89623 | 1.091 | 1.06 | 1.08 | 3595 | 2965 | 3272 |
| K84063 | 1.091 | 0.87 | 0.87 | 3596 | 1130 | 1098 |
| P92635 | 1.091 | 1.19 | 1.21 | 3597 | 4849 | 4991 |
| D83009 | 1.091 | 1.17 | 1.17 | 3598 | 4502 | 4538 |
| J82101 | 1.090 | 1.17 | 1.17 | 3599 | 4495 | 4598 |
| D82011 | 1.090 | 1.09 | 1.10 | 3600 | 3391 | 3643 |
| E82008 | 1.090 | 1.12 | 1.11 | 3601 | 3878 | 3753 |
| E85716 | 1.090 | 1.01 | 1.00 | 3602 | 2355 | 2314 |
| B82041 | 1.090 | 1.16 | 1.14 | 3603 | 4409 | 4121 |
| C84717 | 1.090 | 1.15 | 1.17 | 3604 | 4258 | 4529 |
| J82121 | 1.090 | 1.14 | 1.12 | 3605 | 4130 | 3850 |
| G81051 | 1.090 | 1.12 | 1.07 | 3606 | 3794 | 3188 |
| Y00079 | 1.090 | 1.04 | 1.06 | 3607 | 2703 | 2955 |
| M84037 | 1.090 | 1.16 | 1.15 | 3608 | 4329 | 4350 |
| M82023 | 1.090 | 1.05 | 1.05 | 3609 | 2861 | 2926 |
| E83016 | 1.090 | 0.95 | 0.93 | 3610 | 1729 | 1590 |
| N82091 | 1.090 | 1.17 | 1.18 | 3611 | 4519 | 4659 |
| M83661 | 1.090 | 1.24 | 1.26 | 3612 | 5438 | 5650 |
| B83023 | 1.089 | 1.17 | 1.18 | 3613 | 4565 | 4742 |
| G82062 | 1.089 | 1.02 | 1.02 | 3614 | 2548 | 2548 |
| H82047 | 1.089 | 1.05 | 1.06 | 3615 | 2846 | 3065 |
| G85035 | 1.089 | 0.99 | 1.00 | 3616 | 2221 | 2284 |
| D83081 | 1.089 | 1.13 | 1.13 | 3617 | 3980 | 4013 |
| K82044 | 1.089 | 0.96 | 0.95 | 3618 | 1869 | 1726 |
| C84123 | 1.089 | 1.35 | 1.33 | 3619 | 6450 | 6279 |
| F81034 | 1.089 | 1.03 | 1.00 | 3620 | 2630 | 2257 |
| F81213 | 1.089 | 1.19 | 1.20 | 3621 | 4819 | 4930 |
| N83033 | 1.089 | 1.25 | 1.26 | 3622 | 5560 | 5604 |
| G85005 | 1.088 | 1.14 | 1.14 | 3623 | 4099 | 4216 |
| M85026 | 1.088 | 1.04 | 1.03 | 3624 | 2716 | 2675 |
| C82054 | 1.088 | 1.11 | 1.12 | 3625 | 3796 | 3864 |
| P88617 | 1.088 | 1.22 | 1.20 | 3626 | 5188 | 4937 |
| C81616 | 1.088 | 1.30 | 1.30 | 3627 | 6038 | 6058 |
| G82032 | 1.088 | 1.06 | 1.06 | 3628 | 2976 | 3003 |
| C84018 | 1.088 | 1.09 | 1.11 | 3629 | 3424 | 3725 |
| G83628 | 1.088 | 0.94 | 0.94 | 3630 | 1685 | 1679 |
| M91032 | 1.088 | 1.15 | 1.17 | 3631 | 4291 | 4581 |
| J82619 | 1.088 | 1.10 | 1.11 | 3632 | 3570 | 3686 |
| C82010 | 1.088 | 1.13 | 1.09 | 3633 | 3965 | 3510 |
| H81050 | 1.088 | 0.97 | 0.97 | 3634 | 1977 | 2009 |
| J82099 | 1.087 | 0.93 | 0.91 | 3635 | 1613 | 1390 |
| B87030 | 1.087 | 1.24 | 1.26 | 3636 | 5386 | 5588 |
| H85115 | 1.087 | 1.10 | 1.09 | 3637 | 3624 | 3440 |
| N82104 | 1.087 | 1.25 | 1.27 | 3638 | 5552 | 5734 |
| P81176 | 1.087 | 1.20 | 1.17 | 3639 | 4929 | 4585 |
| F84727 | 1.087 | 1.12 | 1.11 | 3640 | 3941 | 3721 |
| L84058 | 1.087 | 1.13 | 1.13 | 3641 | 4074 | 4066 |
| A82651 | 1.087 | 0.97 | 0.95 | 3642 | 2002 | 1752 |
| E82001 | 1.087 | 0.98 | 0.98 | 3643 | 2114 | 2060 |
| H81615 | 1.087 | 0.91 | 0.92 | 3644 | 1449 | 1482 |

| | | | | | | |
|---|---|---|---|---|---|---|
| J82073 | 1.087 | 1.06 | 1.07 | 3645 | 2990 | 3143 |
| E82088 | 1.087 | 1.08 | 1.08 | 3646 | 3305 | 3286 |
| J83002 | 1.086 | 1.13 | 1.14 | 3647 | 4028 | 4223 |
| P87658 | 1.086 | 1.09 | 1.12 | 3648 | 3514 | 3869 |
| L84016 | 1.086 | 1.09 | 1.09 | 3649 | 3517 | 3434 |
| M83619 | 1.086 | 1.29 | 1.32 | 3650 | 5981 | 6270 |
| Y00984 | 1.086 | 1.10 | 1.08 | 3651 | 3675 | 3304 |
| C87005 | 1.086 | 1.25 | 1.26 | 3652 | 5556 | 5610 |
| E84011 | 1.086 | 1.00 | 0.98 | 3653 | 2276 | 2079 |
| M85115 | 1.086 | 1.11 | 1.11 | 3654 | 3783 | 3730 |
| E81001 | 1.086 | 1.01 | 1.02 | 3655 | 2439 | 2556 |
| F81112 | 1.086 | 1.01 | 1.03 | 3656 | 2470 | 2635 |
| K84060 | 1.086 | 0.97 | 0.96 | 3657 | 1967 | 1930 |
| F86642 | 1.085 | 1.06 | 1.04 | 3658 | 3012 | 2840 |
| F86013 | 1.085 | 0.95 | 0.93 | 3659 | 1803 | 1641 |
| G81002 | 1.085 | 1.12 | 1.13 | 3660 | 3857 | 4127 |
| L85001 | 1.085 | 1.07 | 1.04 | 3661 | 3235 | 2872 |
| D83610 | 1.085 | 1.01 | 1.01 | 3662 | 2387 | 2402 |
| P91021 | 1.085 | 1.10 | 1.11 | 3663 | 3662 | 3801 |
| L82011 | 1.085 | 1.14 | 1.14 | 3664 | 4176 | 4222 |
| H82048 | 1.085 | 1.23 | 1.21 | 3665 | 5328 | 5116 |
| B81622 | 1.085 | 1.12 | 1.12 | 3666 | 3961 | 3966 |
| A83024 | 1.085 | 1.34 | 1.34 | 3667 | 6428 | 6432 |
| H81134 | 1.085 | 1.02 | 1.01 | 3668 | 2553 | 2424 |
| C82610 | 1.085 | 0.99 | 0.99 | 3669 | 2171 | 2197 |
| M81617 | 1.085 | 1.11 | 1.13 | 3670 | 3790 | 4109 |
| H81072 | 1.085 | 1.01 | 0.99 | 3671 | 2510 | 2169 |
| G82069 | 1.084 | 1.08 | 1.11 | 3672 | 3263 | 3803 |
| E83030 | 1.084 | 1.05 | 1.03 | 3673 | 2927 | 2682 |
| G83022 | 1.084 | 1.02 | 1.02 | 3674 | 2521 | 2531 |
| E84690 | 1.084 | 0.95 | 0.94 | 3675 | 1789 | 1681 |
| G81053 | 1.084 | 1.33 | 1.29 | 3676 | 6295 | 5982 |
| J83618 | 1.084 | 0.99 | 0.98 | 3677 | 2195 | 2170 |
| M81090 | 1.084 | 1.03 | 1.02 | 3678 | 2732 | 2601 |
| M89012 | 1.084 | 1.30 | 1.24 | 3679 | 6097 | 5507 |
| J82169 | 1.084 | 1.00 | 0.99 | 3680 | 2304 | 2178 |
| B81060 | 1.084 | 1.54 | 1.50 | 3681 | 7167 | 7116 |
| B86109 | 1.084 | 0.89 | 0.91 | 3682 | 1264 | 1425 |
| K82027 | 1.084 | 1.01 | 1.00 | 3683 | 2467 | 2403 |
| G85662 | 1.083 | 0.95 | 0.96 | 3684 | 1801 | 1914 |
| B83049 | 1.083 | 1.18 | 1.19 | 3685 | 4768 | 4880 |
| J84014 | 1.083 | 1.11 | 1.11 | 3686 | 3739 | 3796 |
| C82039 | 1.083 | 1.00 | 1.00 | 3687 | 2381 | 2412 |
| K81076 | 1.083 | 0.95 | 0.94 | 3688 | 1857 | 1741 |
| F81222 | 1.083 | 0.98 | 0.99 | 3689 | 2115 | 2230 |
| K83010 | 1.083 | 1.06 | 1.08 | 3690 | 3100 | 3334 |
| M82041 | 1.083 | 1.11 | 1.11 | 3691 | 3791 | 3842 |
| L84033 | 1.083 | 1.05 | 1.06 | 3692 | 2993 | 3064 |
| H81091 | 1.083 | 1.03 | 1.00 | 3693 | 2729 | 2390 |
| A86010 | 1.082 | 1.01 | 1.03 | 3694 | 2500 | 2704 |

| | | | | | | |
|---|---|---|---|---|---|---|
| P85011 | 1.082 | 1.07 | 1.09 | 3695 | 3281 | 3554 |
| H81028 | 1.082 | 1.04 | 1.01 | 3696 | 2800 | 2446 |
| N82092 | 1.082 | 1.09 | 1.11 | 3697 | 3573 | 3891 |
| M87024 | 1.082 | 1.20 | 1.19 | 3698 | 5050 | 4951 |
| M84017 | 1.082 | 0.99 | 0.99 | 3699 | 2239 | 2232 |
| P81132 | 1.082 | 1.19 | 1.19 | 3700 | 4934 | 4924 |
| M83697 | 1.082 | 1.32 | 1.32 | 3701 | 6309 | 6297 |
| G82156 | 1.082 | 0.98 | 0.99 | 3702 | 2126 | 2238 |
| L81128 | 1.082 | 1.10 | 1.09 | 3703 | 3699 | 3608 |
| G82768 | 1.082 | 0.98 | 0.98 | 3704 | 2149 | 2152 |
| L81043 | 1.082 | 1.27 | 1.23 | 3705 | 5796 | 5429 |
| K82076 | 1.082 | 0.95 | 0.95 | 3706 | 1864 | 1833 |
| P88019 | 1.082 | 1.13 | 1.12 | 3707 | 4045 | 3953 |
| K81041 | 1.081 | 1.02 | 1.02 | 3708 | 2582 | 2652 |
| P82012 | 1.081 | 1.24 | 1.25 | 3709 | 5540 | 5595 |
| C86007 | 1.081 | 1.17 | 1.19 | 3710 | 4657 | 4959 |
| J84017 | 1.081 | 1.17 | 1.17 | 3711 | 4696 | 4661 |
| G81087 | 1.081 | 1.09 | 1.05 | 3712 | 3529 | 3004 |
| C81080 | 1.081 | 1.16 | 1.15 | 3713 | 4463 | 4352 |
| N85005 | 1.081 | 1.13 | 1.12 | 3714 | 4135 | 4005 |
| N82106 | 1.081 | 1.18 | 1.20 | 3715 | 4836 | 4980 |
| J81025 | 1.081 | 1.13 | 1.09 | 3716 | 4165 | 3632 |
| C88092 | 1.081 | 1.19 | 1.21 | 3717 | 4961 | 5115 |
| M87620 | 1.081 | 0.95 | 0.95 | 3718 | 1816 | 1864 |
| E82073 | 1.081 | 0.96 | 0.97 | 3719 | 1987 | 2078 |
| E87057 | 1.081 | 1.07 | 1.06 | 3720 | 3233 | 3167 |
| J84012 | 1.081 | 1.00 | 1.01 | 3721 | 2404 | 2460 |
| H81056 | 1.081 | 1.01 | 0.99 | 3722 | 2506 | 2235 |
| C84084 | 1.080 | 1.13 | 1.10 | 3723 | 4092 | 3759 |
| B82011 | 1.080 | 1.20 | 1.22 | 3724 | 5042 | 5305 |
| L84057 | 1.080 | 1.10 | 1.09 | 3725 | 3627 | 3527 |
| L81030 | 1.080 | 1.02 | 1.01 | 3726 | 2572 | 2487 |
| M85781 | 1.080 | 1.15 | 1.14 | 3727 | 4431 | 4304 |
| P91627 | 1.080 | 1.16 | 1.15 | 3728 | 4486 | 4432 |
| C88085 | 1.080 | 1.22 | 1.24 | 3729 | 5336 | 5469 |
| L85005 | 1.080 | 1.23 | 1.23 | 3730 | 5460 | 5393 |
| D81012 | 1.080 | 1.01 | 0.98 | 3731 | 2463 | 2125 |
| E81060 | 1.080 | 1.04 | 1.04 | 3732 | 2885 | 2816 |
| M82030 | 1.080 | 1.19 | 1.19 | 3733 | 4897 | 4893 |
| M83133 | 1.079 | 1.13 | 1.17 | 3734 | 4075 | 4644 |
| P83004 | 1.079 | 1.11 | 1.11 | 3735 | 3817 | 3836 |
| M88030 | 1.079 | 1.38 | 1.39 | 3736 | 6715 | 6751 |
| C86011 | 1.079 | 1.15 | 1.18 | 3737 | 4460 | 4833 |
| L83100 | 1.079 | 1.08 | 1.10 | 3738 | 3380 | 3727 |
| P81704 | 1.079 | 1.21 | 1.23 | 3739 | 5142 | 5394 |
| C84063 | 1.079 | 1.09 | 1.10 | 3740 | 3578 | 3800 |
| G81100 | 1.079 | 1.17 | 1.15 | 3741 | 4688 | 4471 |
| E86625 | 1.079 | 1.00 | 0.99 | 3742 | 2345 | 2286 |
| H84058 | 1.079 | 1.07 | 1.06 | 3743 | 3321 | 3105 |
| E84699 | 1.079 | 1.16 | 1.13 | 3744 | 4490 | 4184 |

| | | | | | | |
|---|---|---|---|---|---|---|
| H81090 | 1.079 | 1.00 | 0.97 | 3745 | 2412 | 2042 |
| B87031 | 1.078 | 1.14 | 1.16 | 3746 | 4345 | 4604 |
| M87006 | 1.078 | 1.25 | 1.24 | 3747 | 5648 | 5534 |
| L84050 | 1.078 | 1.04 | 1.03 | 3748 | 2875 | 2798 |
| E86012 | 1.078 | 1.02 | 1.01 | 3749 | 2602 | 2533 |
| P81691 | 1.078 | 1.04 | 1.04 | 3750 | 2882 | 2930 |
| B82017 | 1.078 | 1.17 | 1.19 | 3751 | 4764 | 4913 |
| B83006 | 1.078 | 1.19 | 1.21 | 3752 | 5015 | 5154 |
| M83015 | 1.078 | 1.22 | 1.23 | 3753 | 5369 | 5404 |
| H81023 | 1.078 | 0.98 | 0.96 | 3754 | 2228 | 1940 |
| L82052 | 1.078 | 1.09 | 1.09 | 3755 | 3583 | 3638 |
| P84046 | 1.078 | 1.07 | 1.09 | 3756 | 3264 | 3604 |
| L83120 | 1.078 | 1.13 | 1.12 | 3757 | 4108 | 4026 |
| D81087 | 1.078 | 1.14 | 1.13 | 3758 | 4354 | 4234 |
| B84618 | 1.078 | 1.20 | 1.21 | 3759 | 5119 | 5250 |
| P88007 | 1.078 | 1.03 | 1.04 | 3760 | 2775 | 2848 |
| P89019 | 1.077 | 1.28 | 1.29 | 3761 | 5900 | 6041 |
| E81049 | 1.077 | 1.08 | 1.08 | 3762 | 3522 | 3406 |
| G82065 | 1.077 | 1.06 | 1.06 | 3763 | 3188 | 3254 |
| C83024 | 1.077 | 1.11 | 1.10 | 3764 | 3927 | 3735 |
| M81055 | 1.077 | 1.20 | 1.17 | 3765 | 5078 | 4662 |
| B82068 | 1.077 | 1.13 | 1.16 | 3766 | 4185 | 4518 |
| M85172 | 1.077 | 1.16 | 1.15 | 3767 | 4533 | 4386 |
| D83015 | 1.077 | 1.27 | 1.26 | 3768 | 5861 | 5789 |
| E85046 | 1.077 | 1.06 | 1.07 | 3769 | 3215 | 3307 |
| M85156 | 1.077 | 1.11 | 1.10 | 3770 | 3959 | 3827 |
| E81024 | 1.077 | 1.07 | 1.06 | 3771 | 3241 | 3223 |
| M85753 | 1.077 | 1.26 | 1.23 | 3772 | 5703 | 5452 |
| A82068 | 1.077 | 1.22 | 1.18 | 3773 | 5354 | 4883 |
| M83012 | 1.077 | 1.19 | 1.14 | 3774 | 4948 | 4263 |
| E81015 | 1.077 | 1.08 | 1.08 | 3775 | 3507 | 3451 |
| A83630 | 1.077 | 1.27 | 1.27 | 3776 | 5862 | 5883 |
| Y03362 | 1.077 | 0.93 | 0.96 | 3777 | 1735 | 1974 |
| A81005 | 1.077 | 1.11 | 1.12 | 3778 | 3890 | 4001 |
| L84053 | 1.076 | 1.11 | 1.09 | 3779 | 3867 | 3654 |
| L85036 | 1.076 | 1.07 | 1.04 | 3780 | 3266 | 2917 |
| P88606 | 1.076 | 1.08 | 1.07 | 3781 | 3419 | 3378 |
| L84024 | 1.076 | 1.25 | 1.23 | 3782 | 5640 | 5495 |
| B82100 | 1.076 | 1.20 | 1.18 | 3783 | 5057 | 4860 |
| D81058 | 1.076 | 1.01 | 1.00 | 3784 | 2525 | 2416 |
| N81087 | 1.076 | 1.09 | 1.07 | 3785 | 3660 | 3365 |
| M88038 | 1.076 | 1.22 | 1.23 | 3786 | 5383 | 5496 |
| H81044 | 1.076 | 1.10 | 1.09 | 3787 | 3760 | 3671 |
| K83049 | 1.076 | 1.05 | 1.02 | 3788 | 3062 | 2694 |
| D82058 | 1.076 | 1.26 | 1.27 | 3789 | 5745 | 5920 |
| B86037 | 1.076 | 1.18 | 1.21 | 3790 | 4875 | 5172 |
| E85012 | 1.076 | 1.30 | 1.30 | 3791 | 6132 | 6108 |
| E85118 | 1.076 | 0.89 | 0.91 | 3792 | 1341 | 1481 |
| D81612 | 1.076 | 1.02 | 1.01 | 3793 | 2692 | 2527 |
| Y00304 | 1.075 | 1.06 | 1.05 | 3794 | 3243 | 3053 |

| | | | | | | |
|---|---|---|---|---|---|---|
| G81658 | 1.075 | 1.19 | 1.22 | 3795 | 5029 | 5369 |
| C81018 | 1.075 | 1.10 | 1.10 | 3796 | 3823 | 3824 |
| J81626 | 1.075 | 1.09 | 1.07 | 3797 | 3688 | 3414 |
| J81056 | 1.075 | 1.18 | 1.18 | 3798 | 4923 | 4843 |
| K81061 | 1.075 | 1.02 | 1.02 | 3799 | 2657 | 2659 |
| H85032 | 1.075 | 1.00 | 0.99 | 3800 | 2436 | 2361 |
| L81035 | 1.075 | 1.03 | 1.03 | 3801 | 2762 | 2803 |
| D82059 | 1.075 | 1.20 | 1.17 | 3802 | 5102 | 4811 |
| G82024 | 1.075 | 1.15 | 1.15 | 3803 | 4477 | 4449 |
| J83630 | 1.074 | 1.10 | 1.09 | 3804 | 3784 | 3665 |
| H83028 | 1.074 | 1.02 | 1.01 | 3805 | 2735 | 2608 |
| F81105 | 1.074 | 1.03 | 1.03 | 3806 | 2868 | 2868 |
| Y02827 | 1.074 | 1.07 | 1.10 | 3807 | 3369 | 3772 |
| C84019 | 1.074 | 1.08 | 1.08 | 3808 | 3531 | 3478 |
| E81072 | 1.074 | 1.02 | 1.02 | 3809 | 2736 | 2684 |
| D81049 | 1.074 | 1.07 | 1.07 | 3810 | 3288 | 3315 |
| M82025 | 1.074 | 1.14 | 1.14 | 3811 | 4408 | 4371 |
| C83017 | 1.074 | 1.08 | 1.06 | 3812 | 3475 | 3302 |
| G82012 | 1.074 | 1.00 | 1.01 | 3813 | 2472 | 2585 |
| N82014 | 1.074 | 1.11 | 1.13 | 3814 | 3922 | 4191 |
| J82149 | 1.074 | 1.01 | 1.02 | 3815 | 2528 | 2645 |
| L83036 | 1.074 | 1.12 | 1.09 | 3816 | 4100 | 3620 |
| P92643 | 1.074 | 1.29 | 1.29 | 3817 | 6114 | 6122 |
| B84009 | 1.073 | 1.07 | 1.09 | 3818 | 3315 | 3650 |
| N84001 | 1.073 | 1.03 | 1.03 | 3819 | 2831 | 2896 |
| F84619 | 1.073 | 0.96 | 0.98 | 3820 | 2050 | 2211 |
| A83070 | 1.073 | 1.11 | 1.12 | 3821 | 3992 | 4072 |
| K83025 | 1.073 | 1.00 | 1.01 | 3822 | 2425 | 2561 |
| E83011 | 1.073 | 1.08 | 1.05 | 3823 | 3467 | 3104 |
| P85602 | 1.073 | 0.96 | 0.99 | 3824 | 2045 | 2301 |
| M88627 | 1.073 | 1.23 | 1.24 | 3825 | 5503 | 5609 |
| B82004 | 1.073 | 1.07 | 1.07 | 3826 | 3299 | 3353 |
| J82648 | 1.073 | 1.12 | 1.15 | 3827 | 4039 | 4473 |
| J83048 | 1.073 | 1.15 | 1.14 | 3828 | 4478 | 4305 |
| P92642 | 1.073 | 1.33 | 1.32 | 3829 | 6431 | 6383 |
| F81021 | 1.073 | 1.23 | 1.23 | 3830 | 5499 | 5518 |
| L85609 | 1.072 | 1.10 | 1.07 | 3831 | 3843 | 3373 |
| C88035 | 1.072 | 1.11 | 1.12 | 3832 | 3964 | 4194 |
| P89618 | 1.072 | 1.24 | 1.25 | 3833 | 5568 | 5746 |
| D82604 | 1.072 | 1.24 | 1.24 | 3834 | 5584 | 5579 |
| B83064 | 1.072 | 1.21 | 1.21 | 3835 | 5233 | 5252 |
| F81006 | 1.072 | 1.13 | 1.13 | 3836 | 4236 | 4245 |
| B81057 | 1.072 | 1.14 | 1.19 | 3837 | 4371 | 4972 |
| F82666 | 1.072 | 1.03 | 1.03 | 3838 | 2851 | 2804 |
| E85107 | 1.072 | 0.98 | 0.98 | 3839 | 2199 | 2245 |
| G82099 | 1.072 | 0.99 | 1.00 | 3840 | 2351 | 2439 |
| N82662 | 1.071 | 1.16 | 1.17 | 3841 | 4585 | 4790 |
| N82648 | 1.071 | 1.08 | 1.10 | 3842 | 3572 | 3893 |
| D81028 | 1.071 | 0.99 | 0.99 | 3843 | 2426 | 2324 |
| B86093 | 1.071 | 1.14 | 1.16 | 3844 | 4404 | 4624 |

| | | | | | | |
|---|---|---|---|---|---|---|
| L81009 | 1.071 | 1.18 | 1.18 | 3845 | 4876 | 4888 |
| C82095 | 1.071 | 1.06 | 1.06 | 3846 | 3184 | 3292 |
| M83049 | 1.071 | 1.17 | 1.15 | 3847 | 4829 | 4592 |
| P82633 | 1.071 | 1.17 | 1.19 | 3848 | 4848 | 4985 |
| E86042 | 1.071 | 0.97 | 0.97 | 3849 | 2167 | 2094 |
| L81085 | 1.071 | 1.15 | 1.12 | 3850 | 4513 | 4175 |
| G82234 | 1.071 | 1.18 | 1.16 | 3851 | 4924 | 4706 |
| M88041 | 1.071 | 1.21 | 1.23 | 3852 | 5296 | 5511 |
| M83038 | 1.071 | 1.18 | 1.22 | 3853 | 4945 | 5428 |
| B87003 | 1.071 | 1.15 | 1.17 | 3854 | 4520 | 4853 |
| P84070 | 1.071 | 1.14 | 1.15 | 3855 | 4335 | 4569 |
| B82079 | 1.071 | 1.07 | 1.08 | 3856 | 3444 | 3507 |
| Y02790 | 1.070 | 0.96 | 0.98 | 3857 | 2084 | 2262 |
| L81055 | 1.070 | 1.12 | 1.11 | 3858 | 4151 | 3967 |
| J82058 | 1.070 | 1.04 | 1.02 | 3859 | 2998 | 2760 |
| F85039 | 1.070 | 0.94 | 0.94 | 3860 | 1893 | 1872 |
| N81027 | 1.070 | 1.09 | 1.08 | 3861 | 3719 | 3556 |
| D83006 | 1.070 | 1.06 | 1.03 | 3862 | 3275 | 2923 |
| F81084 | 1.070 | 1.20 | 1.21 | 3863 | 5219 | 5235 |
| C88083 | 1.070 | 1.04 | 1.05 | 3864 | 2934 | 3220 |
| J83059 | 1.070 | 1.15 | 1.16 | 3865 | 4581 | 4640 |
| H85025 | 1.070 | 1.04 | 1.04 | 3866 | 3056 | 2965 |
| G82162 | 1.070 | 1.12 | 1.14 | 3867 | 4094 | 4428 |
| E85682 | 1.070 | 1.12 | 1.12 | 3868 | 4083 | 4096 |
| M85084 | 1.070 | 1.18 | 1.17 | 3869 | 4944 | 4753 |
| F81184 | 1.069 | 1.04 | 1.02 | 3870 | 3037 | 2808 |
| F86009 | 1.069 | 1.03 | 1.01 | 3871 | 2857 | 2667 |
| L83047 | 1.069 | 1.19 | 1.18 | 3872 | 5084 | 4963 |
| H81034 | 1.069 | 0.99 | 0.99 | 3873 | 2347 | 2423 |
| M83061 | 1.069 | 1.26 | 1.29 | 3874 | 5809 | 6169 |
| F86034 | 1.069 | 1.02 | 1.01 | 3875 | 2822 | 2597 |
| L82070 | 1.069 | 1.10 | 1.10 | 3876 | 3866 | 3922 |
| C81045 | 1.068 | 1.17 | 1.16 | 3877 | 4852 | 4684 |
| F84121 | 1.068 | 1.15 | 1.13 | 3878 | 4526 | 4318 |
| F81737 | 1.068 | 0.90 | 0.86 | 3879 | 1510 | 1219 |
| G81088 | 1.068 | 1.03 | 1.01 | 3880 | 2883 | 2661 |
| K83014 | 1.068 | 1.03 | 1.04 | 3881 | 2845 | 2988 |
| B82105 | 1.068 | 1.17 | 1.15 | 3882 | 4820 | 4547 |
| M85087 | 1.068 | 1.17 | 1.15 | 3883 | 4807 | 4582 |
| B85059 | 1.068 | 1.06 | 1.07 | 3884 | 3289 | 3398 |
| L83105 | 1.068 | 1.15 | 1.15 | 3885 | 4591 | 4572 |
| E85712 | 1.068 | 1.02 | 1.03 | 3886 | 2780 | 2838 |
| F81170 | 1.068 | 0.98 | 0.98 | 3887 | 2287 | 2306 |
| L84010 | 1.068 | 1.16 | 1.14 | 3888 | 4762 | 4499 |
| L83017 | 1.067 | 1.24 | 1.22 | 3889 | 5642 | 5482 |
| B82075 | 1.067 | 1.12 | 1.11 | 3890 | 4113 | 4044 |
| H85077 | 1.067 | 0.95 | 0.94 | 3891 | 2013 | 1834 |
| A83071 | 1.067 | 1.18 | 1.19 | 3892 | 5030 | 5079 |
| C81026 | 1.067 | 1.14 | 1.12 | 3893 | 4415 | 4206 |
| G82704 | 1.067 | 1.05 | 1.08 | 3894 | 3171 | 3591 |

| | | | | | | |
|---|---|---|---|---|---|---|
| C81002 | 1.067 | 1.21 | 1.21 | 3895 | 5391 | 5307 |
| Y02506 | 1.067 | 1.04 | 1.05 | 3896 | 3038 | 3165 |
| K84005 | 1.067 | 1.07 | 1.04 | 3897 | 3532 | 3051 |
| E85053 | 1.067 | 1.02 | 1.02 | 3898 | 2791 | 2827 |
| E82072 | 1.066 | 1.07 | 1.06 | 3899 | 3506 | 3351 |
| J83016 | 1.066 | 1.02 | 1.02 | 3900 | 2844 | 2741 |
| G85009 | 1.066 | 1.34 | 1.30 | 3901 | 6528 | 6224 |
| K81040 | 1.066 | 1.01 | 1.01 | 3902 | 2714 | 2725 |
| B84008 | 1.066 | 1.05 | 1.08 | 3903 | 3218 | 3581 |
| H81040 | 1.066 | 1.07 | 1.05 | 3904 | 3396 | 3173 |
| B82010 | 1.066 | 1.13 | 1.12 | 3905 | 4328 | 4262 |
| G85089 | 1.066 | 0.99 | 1.00 | 3906 | 2493 | 2605 |
| L85017 | 1.066 | 1.14 | 1.14 | 3907 | 4438 | 4478 |
| E81008 | 1.066 | 1.06 | 1.07 | 3908 | 3349 | 3495 |
| M81048 | 1.065 | 1.26 | 1.23 | 3909 | 5821 | 5532 |
| Y02466 | 1.065 | 0.93 | 0.91 | 3910 | 1745 | 1618 |
| P81214 | 1.065 | 1.19 | 1.22 | 3911 | 5075 | 5433 |
| P92626 | 1.065 | 1.27 | 1.27 | 3912 | 5955 | 6021 |
| D81078 | 1.065 | 1.03 | 1.02 | 3913 | 2947 | 2805 |
| B81064 | 1.065 | 1.22 | 1.24 | 3914 | 5463 | 5699 |
| L81025 | 1.065 | 1.10 | 1.09 | 3915 | 3936 | 3769 |
| M85624 | 1.065 | 1.11 | 1.10 | 3916 | 4104 | 3903 |
| P84074 | 1.065 | 1.06 | 1.09 | 3917 | 3353 | 3726 |
| M81091 | 1.065 | 1.11 | 1.10 | 3918 | 4106 | 3937 |
| C86609 | 1.065 | 1.06 | 1.08 | 3919 | 3284 | 3695 |
| K81082 | 1.065 | 0.95 | 0.96 | 3920 | 2021 | 2145 |
| G81098 | 1.065 | 1.12 | 1.09 | 3921 | 4134 | 3820 |
| C82076 | 1.065 | 1.01 | 1.00 | 3922 | 2680 | 2593 |
| C82644 | 1.065 | 1.12 | 1.12 | 3923 | 4243 | 4261 |
| F81721 | 1.065 | 0.98 | 0.98 | 3924 | 2279 | 2278 |
| H83002 | 1.065 | 0.99 | 0.98 | 3925 | 2460 | 2350 |
| M81089 | 1.064 | 1.15 | 1.17 | 3926 | 4562 | 4901 |
| G82227 | 1.064 | 1.16 | 1.17 | 3927 | 4747 | 4931 |
| P88028 | 1.064 | 1.07 | 1.06 | 3928 | 3432 | 3399 |
| C81001 | 1.064 | 1.29 | 1.28 | 3929 | 6134 | 6039 |
| H85103 | 1.064 | 1.08 | 1.06 | 3930 | 3671 | 3422 |
| E82095 | 1.064 | 0.95 | 0.95 | 3931 | 2062 | 1962 |
| P81002 | 1.064 | 1.19 | 1.19 | 3932 | 5083 | 5153 |
| M84038 | 1.064 | 1.10 | 1.10 | 3933 | 3979 | 3925 |
| L83045 | 1.064 | 1.07 | 1.06 | 3934 | 3525 | 3419 |
| P92012 | 1.064 | 1.18 | 1.19 | 3935 | 4978 | 5093 |
| J83011 | 1.064 | 1.18 | 1.17 | 3936 | 5028 | 4834 |
| J81087 | 1.063 | 1.09 | 1.10 | 3937 | 3854 | 3901 |
| C81022 | 1.063 | 1.20 | 1.17 | 3938 | 5284 | 4909 |
| P88610 | 1.063 | 1.19 | 1.17 | 3939 | 5114 | 4918 |
| E85750 | 1.063 | 0.98 | 0.98 | 3940 | 2362 | 2333 |
| L84051 | 1.063 | 1.08 | 1.07 | 3941 | 3630 | 3578 |
| M86037 | 1.063 | 1.01 | 1.01 | 3942 | 2708 | 2649 |
| P81634 | 1.063 | 1.19 | 1.22 | 3943 | 5182 | 5474 |
| C85005 | 1.063 | 1.33 | 1.33 | 3944 | 6513 | 6551 |

| | | | | | | |
|---|---|---|---|---|---|---|
| N85009 | 1.063 | 1.29 | 1.26 | 3945 | 6133 | 5889 |
| M82010 | 1.063 | 1.06 | 1.06 | 3946 | 3376 | 3408 |
| H82076 | 1.063 | 1.10 | 1.10 | 3947 | 3920 | 3949 |
| N82099 | 1.063 | 1.23 | 1.24 | 3948 | 5592 | 5642 |
| A82030 | 1.063 | 1.05 | 1.03 | 3949 | 3182 | 2914 |
| C83029 | 1.063 | 1.19 | 1.19 | 3950 | 5207 | 5126 |
| C84116 | 1.063 | 1.15 | 1.16 | 3951 | 4598 | 4827 |
| D83067 | 1.062 | 1.01 | 1.02 | 3952 | 2709 | 2842 |
| A81029 | 1.062 | 1.14 | 1.15 | 3953 | 4545 | 4654 |
| N81032 | 1.062 | 1.05 | 1.04 | 3954 | 3274 | 3174 |
| F81728 | 1.062 | 1.09 | 1.07 | 3955 | 3766 | 3589 |
| C84142 | 1.062 | 1.10 | 1.11 | 3956 | 3942 | 4111 |
| C84067 | 1.062 | 1.28 | 1.30 | 3957 | 6136 | 6248 |
| A82650 | 1.062 | 1.10 | 1.07 | 3958 | 4020 | 3605 |
| M81600 | 1.062 | 1.09 | 1.08 | 3959 | 3803 | 3644 |
| H82045 | 1.061 | 1.16 | 1.15 | 3960 | 4757 | 4730 |
| H85649 | 1.061 | 0.93 | 0.92 | 3961 | 1831 | 1688 |
| N84013 | 1.061 | 1.15 | 1.15 | 3962 | 4710 | 4722 |
| M91007 | 1.061 | 1.17 | 1.19 | 3963 | 4915 | 5099 |
| M92026 | 1.061 | 1.09 | 1.11 | 3964 | 3832 | 4134 |
| M87019 | 1.061 | 1.15 | 1.14 | 3965 | 4642 | 4574 |
| H82021 | 1.061 | 1.06 | 1.05 | 3966 | 3312 | 3306 |
| C84034 | 1.061 | 1.21 | 1.22 | 3967 | 5367 | 5460 |
| H81027 | 1.061 | 0.95 | 0.95 | 3968 | 2018 | 2005 |
| H81094 | 1.061 | 0.97 | 0.95 | 3969 | 2205 | 2038 |
| H82009 | 1.061 | 1.10 | 1.10 | 3970 | 4003 | 4035 |
| C83009 | 1.060 | 1.15 | 1.18 | 3971 | 4605 | 5061 |
| B81036 | 1.060 | 1.10 | 1.09 | 3972 | 3929 | 3914 |
| B81045 | 1.060 | 1.20 | 1.22 | 3973 | 5297 | 5477 |
| A83048 | 1.060 | 1.07 | 1.08 | 3974 | 3492 | 3677 |
| D81014 | 1.060 | 1.12 | 1.10 | 3975 | 4223 | 4046 |
| G82142 | 1.060 | 1.02 | 1.01 | 3976 | 2864 | 2782 |
| G82074 | 1.060 | 0.98 | 0.99 | 3977 | 2354 | 2462 |
| J82135 | 1.060 | 1.00 | 0.97 | 3978 | 2648 | 2253 |
| D82066 | 1.059 | 1.18 | 1.16 | 3979 | 5113 | 4807 |
| H81075 | 1.059 | 1.01 | 1.00 | 3980 | 2754 | 2586 |
| M85158 | 1.059 | 1.17 | 1.16 | 3981 | 4992 | 4771 |
| C81075 | 1.059 | 1.09 | 1.09 | 3982 | 3880 | 3918 |
| J81634 | 1.059 | 1.01 | 1.01 | 3983 | 2753 | 2734 |
| P89014 | 1.059 | 1.24 | 1.26 | 3984 | 5737 | 5925 |
| N82039 | 1.058 | 1.12 | 1.14 | 3985 | 4221 | 4501 |
| F86020 | 1.058 | 0.89 | 0.88 | 3986 | 1457 | 1383 |
| M84009 | 1.058 | 1.00 | 1.01 | 3987 | 2694 | 2779 |
| A83054 | 1.058 | 1.25 | 1.26 | 3988 | 5820 | 5914 |
| J81077 | 1.058 | 1.10 | 1.09 | 3989 | 3960 | 3837 |
| E81025 | 1.057 | 1.18 | 1.18 | 3990 | 5085 | 5108 |
| A82035 | 1.057 | 1.11 | 1.09 | 3991 | 4097 | 3904 |
| L85611 | 1.057 | 1.04 | 1.02 | 3992 | 3095 | 2893 |
| J82141 | 1.057 | 1.13 | 1.14 | 3993 | 4416 | 4556 |
| N82655 | 1.057 | 1.34 | 1.34 | 3994 | 6597 | 6608 |

| | | | | | | |
|---|---|---|---|---|---|---|
| M88006 | 1.057 | 1.14 | 1.15 | 3995 | 4544 | 4774 |
| H84016 | 1.057 | 0.98 | 0.97 | 3996 | 2487 | 2315 |
| H82083 | 1.057 | 1.00 | 1.02 | 3997 | 2683 | 2882 |
| E84022 | 1.057 | 1.09 | 1.07 | 3998 | 3919 | 3590 |
| D83050 | 1.057 | 1.09 | 1.10 | 3999 | 3930 | 4027 |
| N82100 | 1.057 | 1.16 | 1.18 | 4000 | 4827 | 5035 |
| D81035 | 1.056 | 1.06 | 1.05 | 4001 | 3480 | 3384 |
| J81019 | 1.056 | 1.09 | 1.09 | 4002 | 3972 | 3936 |
| N83049 | 1.056 | 1.16 | 1.18 | 4003 | 4860 | 5040 |
| P92029 | 1.056 | 1.10 | 1.10 | 4004 | 4018 | 4117 |
| C83004 | 1.056 | 1.06 | 1.05 | 4005 | 3407 | 3377 |
| C82088 | 1.056 | 1.17 | 1.18 | 4006 | 5003 | 5085 |
| M91010 | 1.056 | 1.15 | 1.17 | 4007 | 4792 | 5006 |
| M91011 | 1.056 | 1.08 | 1.11 | 4008 | 3836 | 4145 |
| H82099 | 1.056 | 1.27 | 1.25 | 4009 | 6014 | 5910 |
| M82024 | 1.055 | 1.02 | 1.02 | 4010 | 2872 | 2939 |
| A82629 | 1.055 | 1.36 | 1.38 | 4011 | 6755 | 6824 |
| B81655 | 1.055 | 1.19 | 1.21 | 4012 | 5192 | 5484 |
| K84008 | 1.055 | 1.05 | 1.03 | 4013 | 3378 | 3034 |
| P81032 | 1.055 | 1.23 | 1.27 | 4014 | 5719 | 6071 |
| N82024 | 1.055 | 1.08 | 1.10 | 4015 | 3808 | 4030 |
| E84013 | 1.055 | 1.05 | 1.03 | 4016 | 3329 | 3112 |
| A82007 | 1.055 | 1.19 | 1.22 | 4017 | 5211 | 5551 |
| J81048 | 1.055 | 1.00 | 1.00 | 4018 | 2686 | 2739 |
| M81006 | 1.055 | 1.00 | 1.01 | 4019 | 2712 | 2825 |
| E85739 | 1.055 | 1.03 | 1.02 | 4020 | 3058 | 3019 |
| L81013 | 1.055 | 1.06 | 1.07 | 4021 | 3442 | 3586 |
| N83614 | 1.055 | 1.07 | 1.08 | 4022 | 3608 | 3811 |
| C82600 | 1.055 | 1.05 | 1.06 | 4023 | 3338 | 3430 |
| E85006 | 1.054 | 1.08 | 1.08 | 4024 | 3718 | 3780 |
| F83056 | 1.054 | 1.10 | 1.10 | 4025 | 4076 | 4120 |
| G82711 | 1.054 | 1.03 | 1.06 | 4026 | 3072 | 3506 |
| M91629 | 1.054 | 1.05 | 1.07 | 4027 | 3371 | 3646 |
| C81658 | 1.054 | 1.04 | 1.04 | 4028 | 3156 | 3247 |
| C82078 | 1.054 | 1.08 | 1.08 | 4029 | 3818 | 3847 |
| Y02933 | 1.054 | 1.22 | 1.23 | 4030 | 5608 | 5720 |
| G82105 | 1.054 | 1.10 | 1.12 | 4031 | 4126 | 4367 |
| N81010 | 1.054 | 1.11 | 1.09 | 4032 | 4191 | 3983 |
| M87001 | 1.054 | 1.06 | 1.06 | 4033 | 3565 | 3573 |
| C83631 | 1.054 | 1.06 | 1.06 | 4034 | 3454 | 3530 |
| A82018 | 1.054 | 1.14 | 1.16 | 4035 | 4587 | 4928 |
| K83059 | 1.054 | 1.22 | 1.24 | 4036 | 5537 | 5755 |
| J83064 | 1.054 | 1.05 | 1.06 | 4037 | 3319 | 3512 |
| A82057 | 1.054 | 1.06 | 1.08 | 4038 | 3537 | 3777 |
| M84030 | 1.054 | 1.09 | 1.10 | 4039 | 3991 | 4020 |
| M91616 | 1.054 | 1.26 | 1.28 | 4040 | 5959 | 6126 |
| F86703 | 1.054 | 1.02 | 1.00 | 4041 | 2939 | 2749 |
| M85046 | 1.054 | 1.05 | 1.04 | 4042 | 3307 | 3259 |
| E81069 | 1.054 | 1.30 | 1.28 | 4043 | 6310 | 6153 |
| F81121 | 1.054 | 1.00 | 1.01 | 4044 | 2675 | 2815 |

| | | | | | | |
|---|---|---|---|---|---|---|
| M83624 | 1.054 | 1.41 | 1.42 | 4045 | 6957 | 7007 |
| L83094 | 1.053 | 1.12 | 1.12 | 4046 | 4315 | 4331 |
| L84615 | 1.053 | 1.05 | 1.04 | 4047 | 3331 | 3268 |
| P89003 | 1.053 | 1.17 | 1.20 | 4048 | 5010 | 5311 |
| J83030 | 1.053 | 1.07 | 1.06 | 4049 | 3601 | 3508 |
| K81092 | 1.053 | 0.92 | 0.90 | 4050 | 1809 | 1630 |
| C84124 | 1.053 | 1.11 | 1.13 | 4051 | 4233 | 4505 |
| C84047 | 1.053 | 1.16 | 1.13 | 4052 | 4868 | 4476 |
| G84016 | 1.053 | 0.99 | 0.99 | 4053 | 2579 | 2543 |
| P88016 | 1.053 | 1.09 | 1.09 | 4054 | 4001 | 3972 |
| K81089 | 1.053 | 0.98 | 1.00 | 4055 | 2492 | 2654 |
| L81059 | 1.053 | 1.05 | 1.04 | 4056 | 3332 | 3207 |
| K82030 | 1.052 | 1.06 | 1.03 | 4057 | 3464 | 3184 |
| F81024 | 1.052 | 1.07 | 1.06 | 4058 | 3594 | 3583 |
| A83043 | 1.052 | 1.23 | 1.25 | 4059 | 5709 | 5856 |
| E82034 | 1.052 | 1.09 | 1.06 | 4060 | 3956 | 3585 |
| L82013 | 1.052 | 1.16 | 1.16 | 4061 | 4881 | 4862 |
| K83080 | 1.052 | 1.04 | 1.04 | 4062 | 3181 | 3204 |
| G85021 | 1.052 | 1.01 | 1.02 | 4063 | 2818 | 2936 |
| P91014 | 1.052 | 1.08 | 1.09 | 4064 | 3847 | 3976 |
| P82625 | 1.052 | 1.10 | 1.11 | 4065 | 4043 | 4283 |
| M83041 | 1.052 | 1.13 | 1.09 | 4066 | 4537 | 4048 |
| P86002 | 1.052 | 1.36 | 1.38 | 4067 | 6760 | 6839 |
| A86003 | 1.052 | 0.89 | 0.91 | 4068 | 1538 | 1683 |
| E83044 | 1.051 | 0.98 | 0.95 | 4069 | 2448 | 2182 |
| F82653 | 1.051 | 0.90 | 0.90 | 4070 | 1604 | 1634 |
| L85026 | 1.051 | 1.14 | 1.15 | 4071 | 4679 | 4748 |
| N82002 | 1.051 | 1.35 | 1.35 | 4072 | 6721 | 6722 |
| E85126 | 1.051 | 1.00 | 0.99 | 4073 | 2713 | 2643 |
| L83083 | 1.051 | 1.16 | 1.16 | 4074 | 4927 | 4865 |
| P88042 | 1.051 | 1.06 | 1.06 | 4075 | 3469 | 3518 |
| J82097 | 1.051 | 1.13 | 1.12 | 4076 | 4552 | 4338 |
| K82009 | 1.051 | 1.02 | 1.01 | 4077 | 2933 | 2874 |
| M87638 | 1.051 | 1.26 | 1.24 | 4078 | 5978 | 5849 |
| M82050 | 1.051 | 1.10 | 1.10 | 4079 | 4149 | 4201 |
| N82097 | 1.051 | 1.17 | 1.18 | 4080 | 5002 | 5107 |
| E86629 | 1.051 | 0.94 | 0.94 | 4081 | 2093 | 2032 |
| F86708 | 1.050 | 0.99 | 1.01 | 4082 | 2634 | 2892 |
| N81028 | 1.050 | 1.08 | 1.08 | 4083 | 3914 | 3785 |
| C83060 | 1.050 | 1.16 | 1.15 | 4084 | 4870 | 4823 |
| A81611 | 1.050 | 1.10 | 1.11 | 4085 | 4063 | 4302 |
| F84038 | 1.050 | 1.10 | 1.11 | 4086 | 4117 | 4266 |
| P84611 | 1.050 | 0.92 | 0.94 | 4087 | 1870 | 2073 |
| C83043 | 1.050 | 1.20 | 1.20 | 4088 | 5456 | 5361 |
| H82095 | 1.050 | 1.19 | 1.20 | 4089 | 5306 | 5426 |
| M88021 | 1.050 | 1.09 | 1.11 | 4090 | 4034 | 4290 |
| L83145 | 1.050 | 1.27 | 1.24 | 4091 | 6071 | 5811 |
| F86731 | 1.050 | 0.95 | 0.93 | 4092 | 2164 | 1980 |
| M83066 | 1.050 | 1.12 | 1.15 | 4093 | 4427 | 4868 |
| M86010 | 1.049 | 1.11 | 1.10 | 4094 | 4290 | 4209 |

| | | | | | | |
|---|---|---|---|---|---|---|
| J83644 | 1.049 | 1.03 | 1.04 | 4095 | 3080 | 3273 |
| L82058 | 1.049 | 1.14 | 1.15 | 4096 | 4694 | 4767 |
| E87756 | 1.049 | 1.03 | 1.03 | 4097 | 3186 | 3186 |
| D81602 | 1.049 | 1.07 | 1.05 | 4098 | 3677 | 3463 |
| Y02117 | 1.049 | 0.98 | 0.99 | 4099 | 2527 | 2602 |
| L83138 | 1.049 | 1.05 | 1.07 | 4100 | 3377 | 3729 |
| B82072 | 1.049 | 1.10 | 1.09 | 4101 | 4091 | 4017 |
| N85617 | 1.049 | 1.03 | 1.03 | 4102 | 3101 | 3136 |
| A88023 | 1.049 | 1.16 | 1.16 | 4103 | 4928 | 4917 |
| M89005 | 1.049 | 1.14 | 1.10 | 4104 | 4623 | 4154 |
| F83018 | 1.049 | 1.10 | 1.09 | 4105 | 4127 | 3943 |
| F81038 | 1.048 | 1.08 | 1.03 | 4106 | 3870 | 3171 |
| B85054 | 1.048 | 1.15 | 1.15 | 4107 | 4784 | 4787 |
| F82650 | 1.048 | 0.95 | 0.97 | 4108 | 2223 | 2367 |
| K83023 | 1.048 | 1.09 | 1.08 | 4109 | 3957 | 3941 |
| P81173 | 1.048 | 1.09 | 1.06 | 4110 | 3971 | 3651 |
| N81084 | 1.048 | 1.04 | 1.03 | 4111 | 3262 | 3154 |
| J82157 | 1.048 | 1.08 | 1.08 | 4112 | 3918 | 3859 |
| M83071 | 1.048 | 1.21 | 1.16 | 4113 | 5512 | 4957 |
| F85676 | 1.048 | 0.97 | 0.97 | 4114 | 2452 | 2417 |
| P84053 | 1.048 | 1.03 | 1.05 | 4115 | 3149 | 3427 |
| J81062 | 1.048 | 1.08 | 1.08 | 4116 | 3906 | 3924 |
| P84037 | 1.048 | 0.95 | 0.97 | 4117 | 2197 | 2459 |
| K83614 | 1.047 | 1.24 | 1.26 | 4118 | 5798 | 6027 |
| B82005 | 1.047 | 1.03 | 1.02 | 4119 | 3132 | 3102 |
| P92001 | 1.047 | 1.19 | 1.20 | 4120 | 5326 | 5396 |
| C84612 | 1.047 | 1.06 | 1.05 | 4121 | 3652 | 3390 |
| J82112 | 1.047 | 1.07 | 1.06 | 4122 | 3799 | 3659 |
| N81020 | 1.047 | 1.14 | 1.14 | 4123 | 4746 | 4660 |
| F82609 | 1.047 | 1.11 | 1.11 | 4124 | 4336 | 4273 |
| B81042 | 1.047 | 1.05 | 1.06 | 4125 | 3474 | 3558 |
| E85021 | 1.047 | 1.03 | 1.03 | 4126 | 3125 | 3158 |
| D82023 | 1.047 | 1.05 | 1.04 | 4127 | 3388 | 3285 |
| B86025 | 1.047 | 0.92 | 0.94 | 4128 | 1910 | 2115 |
| F81061 | 1.047 | 1.23 | 1.20 | 4129 | 5787 | 5427 |
| L81031 | 1.046 | 1.06 | 1.07 | 4130 | 3592 | 3736 |
| G85015 | 1.046 | 1.00 | 1.01 | 4131 | 2833 | 2981 |
| E83633 | 1.046 | 1.16 | 1.13 | 4132 | 4953 | 4579 |
| M81073 | 1.046 | 1.24 | 1.22 | 4133 | 5850 | 5645 |
| P92024 | 1.046 | 1.14 | 1.15 | 4134 | 4738 | 4858 |
| M82056 | 1.046 | 0.96 | 0.98 | 4135 | 2292 | 2525 |
| C83016 | 1.046 | 1.09 | 1.13 | 4136 | 4065 | 4601 |
| B83030 | 1.046 | 1.23 | 1.23 | 4137 | 5732 | 5765 |
| Y02332 | 1.046 | 1.15 | 1.16 | 4138 | 4828 | 4983 |
| H83031 | 1.046 | 1.08 | 1.07 | 4139 | 3925 | 3744 |
| M85741 | 1.045 | 1.09 | 1.08 | 4140 | 4078 | 3931 |
| L85051 | 1.045 | 1.13 | 1.13 | 4141 | 4601 | 4587 |
| F81004 | 1.045 | 1.08 | 1.04 | 4142 | 3907 | 3404 |
| L84008 | 1.045 | 1.08 | 1.09 | 4143 | 3969 | 4095 |
| L82030 | 1.045 | 1.08 | 1.09 | 4144 | 3990 | 4025 |

| | | | | | | |
|---|---|---|---|---|---|---|
| L85601 | 1.045 | 1.10 | 1.11 | 4145 | 4184 | 4319 |
| H81048 | 1.045 | 0.92 | 0.91 | 4146 | 1861 | 1760 |
| F85027 | 1.045 | 1.03 | 1.02 | 4147 | 3124 | 3103 |
| F84070 | 1.045 | 1.06 | 1.04 | 4148 | 3593 | 3400 |
| P89602 | 1.045 | 1.13 | 1.15 | 4149 | 4595 | 4911 |
| N82058 | 1.045 | 1.17 | 1.18 | 4150 | 5096 | 5201 |
| L82622 | 1.045 | 1.00 | 1.01 | 4151 | 2873 | 2998 |
| E83036 | 1.045 | 0.97 | 0.95 | 4152 | 2423 | 2180 |
| B85005 | 1.044 | 1.01 | 1.02 | 4153 | 2942 | 3046 |
| C87003 | 1.044 | 1.19 | 1.20 | 4154 | 5327 | 5446 |
| J83053 | 1.044 | 1.05 | 1.04 | 4155 | 3405 | 3356 |
| K83002 | 1.044 | 1.13 | 1.16 | 4156 | 4575 | 4946 |
| D83069 | 1.044 | 1.10 | 1.10 | 4157 | 4195 | 4293 |
| G82089 | 1.044 | 1.01 | 1.02 | 4158 | 2926 | 3032 |
| B86035 | 1.044 | 1.22 | 1.22 | 4159 | 5705 | 5684 |
| C88088 | 1.044 | 1.35 | 1.36 | 4160 | 6750 | 6813 |
| Y02885 | 1.044 | 1.10 | 1.10 | 4161 | 4220 | 4260 |
| C81034 | 1.044 | 1.06 | 1.05 | 4162 | 3642 | 3568 |
| M81020 | 1.044 | 1.09 | 1.12 | 4163 | 4116 | 4504 |
| H83050 | 1.044 | 0.97 | 0.96 | 4164 | 2424 | 2338 |
| C82623 | 1.043 | 0.99 | 1.00 | 4165 | 2759 | 2795 |
| A86008 | 1.043 | 1.11 | 1.12 | 4166 | 4297 | 4484 |
| A87014 | 1.043 | 1.17 | 1.18 | 4167 | 5132 | 5221 |
| D81017 | 1.043 | 0.97 | 0.94 | 4168 | 2475 | 2119 |
| P91035 | 1.043 | 1.13 | 1.13 | 4169 | 4613 | 4699 |
| P92030 | 1.043 | 1.02 | 1.03 | 4170 | 3068 | 3202 |
| Y02960 | 1.043 | 0.88 | 0.90 | 4171 | 1516 | 1718 |
| B81645 | 1.043 | 1.20 | 1.24 | 4172 | 5440 | 5872 |
| H83021 | 1.043 | 1.12 | 1.11 | 4173 | 4498 | 4387 |
| C83003 | 1.043 | 1.08 | 1.08 | 4174 | 3923 | 3920 |
| E85026 | 1.043 | 0.96 | 0.96 | 4175 | 2300 | 2373 |
| N81085 | 1.043 | 1.06 | 1.04 | 4176 | 3634 | 3411 |
| H82064 | 1.043 | 1.05 | 1.07 | 4177 | 3535 | 3774 |
| B86625 | 1.042 | 1.15 | 1.17 | 4178 | 4873 | 5102 |
| B82078 | 1.042 | 1.11 | 1.11 | 4179 | 4430 | 4334 |
| P92015 | 1.042 | 1.30 | 1.30 | 4180 | 6420 | 6429 |
| F84017 | 1.042 | 1.01 | 0.99 | 4181 | 2924 | 2707 |
| J82052 | 1.042 | 1.04 | 1.01 | 4182 | 3401 | 2948 |
| L84026 | 1.042 | 1.10 | 1.09 | 4183 | 4208 | 4146 |
| E82644 | 1.042 | 0.88 | 0.88 | 4184 | 1548 | 1527 |
| J82147 | 1.042 | 0.98 | 0.98 | 4185 | 2641 | 2622 |
| B81663 | 1.042 | 0.98 | 1.01 | 4186 | 2663 | 3048 |
| F84696 | 1.042 | 1.08 | 1.08 | 4187 | 4006 | 3938 |
| B87020 | 1.042 | 1.15 | 1.18 | 4188 | 4925 | 5223 |
| C83074 | 1.042 | 1.21 | 1.19 | 4189 | 5541 | 5409 |
| D83040 | 1.041 | 1.05 | 1.05 | 4190 | 3588 | 3595 |
| A89028 | 1.041 | 1.16 | 1.18 | 4191 | 5055 | 5258 |
| C81615 | 1.041 | 1.41 | 1.37 | 4192 | 7007 | 6844 |
| H81039 | 1.041 | 0.95 | 0.94 | 4193 | 2299 | 2184 |
| M87007 | 1.041 | 1.15 | 1.15 | 4194 | 4933 | 4873 |

| | | | | | | |
|---|---|---|---|---|---|---|
| K82038 | 1.041 | 1.11 | 1.08 | 4195 | 4352 | 3945 |
| M88640 | 1.041 | 1.32 | 1.34 | 4196 | 6588 | 6714 |
| F84658 | 1.041 | 1.08 | 1.06 | 4197 | 3944 | 3728 |
| H83010 | 1.041 | 0.92 | 0.92 | 4198 | 1950 | 1907 |
| P88623 | 1.041 | 1.12 | 1.12 | 4199 | 4555 | 4597 |
| G82751 | 1.041 | 0.98 | 0.99 | 4200 | 2677 | 2731 |
| N81125 | 1.041 | 1.10 | 1.08 | 4201 | 4283 | 4023 |
| M84060 | 1.041 | 1.05 | 1.05 | 4202 | 3540 | 3569 |
| G82002 | 1.041 | 1.09 | 1.10 | 4203 | 4167 | 4332 |
| Y03363 | 1.040 | 0.94 | 0.97 | 4204 | 2204 | 2461 |
| K83007 | 1.040 | 1.13 | 1.13 | 4205 | 4687 | 4627 |
| M87010 | 1.040 | 1.22 | 1.20 | 4206 | 5710 | 5519 |
| K81059 | 1.040 | 1.02 | 1.02 | 4207 | 3155 | 3140 |
| D81022 | 1.040 | 1.08 | 1.09 | 4208 | 4024 | 4179 |
| C81074 | 1.040 | 1.16 | 1.15 | 4209 | 5054 | 4908 |
| J82069 | 1.040 | 1.04 | 1.03 | 4210 | 3453 | 3228 |
| E83638 | 1.040 | 0.94 | 0.93 | 4211 | 2185 | 2033 |
| M87025 | 1.040 | 1.33 | 1.31 | 4212 | 6634 | 6566 |
| P89029 | 1.040 | 1.24 | 1.25 | 4213 | 5951 | 6049 |
| L85010 | 1.040 | 1.24 | 1.24 | 4214 | 5915 | 5898 |
| E84696 | 1.040 | 1.13 | 1.11 | 4215 | 4632 | 4359 |
| L81027 | 1.040 | 1.01 | 1.00 | 4216 | 2963 | 2843 |
| G85722 | 1.040 | 1.00 | 1.01 | 4217 | 2832 | 2969 |
| P88625 | 1.040 | 1.18 | 1.17 | 4218 | 5259 | 5139 |
| D83038 | 1.039 | 1.07 | 1.07 | 4219 | 3846 | 3822 |
| G84017 | 1.039 | 1.01 | 1.01 | 4220 | 3036 | 2991 |
| C81106 | 1.039 | 1.19 | 1.17 | 4221 | 5407 | 5141 |
| L82022 | 1.039 | 1.27 | 1.26 | 4222 | 6175 | 6130 |
| A86031 | 1.039 | 1.06 | 1.07 | 4223 | 3710 | 3881 |
| C82669 | 1.039 | 1.04 | 1.04 | 4224 | 3334 | 3381 |
| E84069 | 1.039 | 1.13 | 1.10 | 4225 | 4736 | 4360 |
| M85055 | 1.039 | 1.00 | 0.99 | 4226 | 2835 | 2750 |
| M85047 | 1.039 | 1.07 | 1.07 | 4227 | 3926 | 3830 |
| G85124 | 1.039 | 1.09 | 1.10 | 4228 | 4192 | 4242 |
| L81132 | 1.039 | 1.03 | 1.02 | 4229 | 3203 | 3117 |
| B87013 | 1.039 | 1.18 | 1.20 | 4230 | 5347 | 5525 |
| B82030 | 1.039 | 1.07 | 1.07 | 4231 | 3809 | 3826 |
| E81031 | 1.039 | 1.11 | 1.10 | 4232 | 4362 | 4317 |
| A83010 | 1.039 | 1.14 | 1.14 | 4233 | 4805 | 4828 |
| C81076 | 1.038 | 1.05 | 1.05 | 4234 | 3524 | 3551 |
| F81013 | 1.038 | 0.98 | 0.99 | 4235 | 2609 | 2767 |
| F81102 | 1.038 | 0.99 | 0.95 | 4236 | 2721 | 2254 |
| E82011 | 1.038 | 1.03 | 1.02 | 4237 | 3221 | 3147 |
| A82031 | 1.038 | 1.21 | 1.18 | 4238 | 5600 | 5325 |
| F81020 | 1.038 | 1.06 | 1.06 | 4239 | 3797 | 3778 |
| M84016 | 1.038 | 1.06 | 1.05 | 4240 | 3729 | 3577 |
| C84035 | 1.038 | 1.12 | 1.11 | 4241 | 4522 | 4470 |
| C88019 | 1.038 | 1.15 | 1.17 | 4242 | 5022 | 5133 |
| M86038 | 1.038 | 1.08 | 1.07 | 4243 | 4021 | 3875 |
| B81063 | 1.038 | 1.11 | 1.14 | 4244 | 4417 | 4770 |

| | | | | | | |
|---|---|---|---|---|---|---|
| E83017 | 1.038 | 1.04 | 1.02 | 4245 | 3468 | 3191 |
| F81733 | 1.038 | 1.21 | 1.22 | 4246 | 5599 | 5750 |
| M81021 | 1.037 | 1.04 | 1.02 | 4247 | 3483 | 3215 |
| N81006 | 1.037 | 1.02 | 1.01 | 4248 | 3174 | 3095 |
| J83001 | 1.037 | 1.12 | 1.13 | 4249 | 4560 | 4657 |
| A81007 | 1.037 | 1.25 | 1.29 | 4250 | 6079 | 6370 |
| J83026 | 1.037 | 1.08 | 1.07 | 4251 | 4042 | 3929 |
| E82027 | 1.037 | 0.99 | 1.00 | 4252 | 2830 | 2944 |
| Y02181 | 1.037 | 1.08 | 1.10 | 4253 | 4059 | 4279 |
| P91008 | 1.037 | 1.03 | 1.04 | 4254 | 3361 | 3531 |
| M83094 | 1.037 | 1.19 | 1.22 | 4255 | 5414 | 5743 |
| M85019 | 1.037 | 1.23 | 1.21 | 4256 | 5823 | 5696 |
| E84077 | 1.037 | 1.04 | 1.02 | 4257 | 3462 | 3241 |
| C88031 | 1.036 | 1.13 | 1.14 | 4258 | 4652 | 4845 |
| L83069 | 1.036 | 1.22 | 1.21 | 4259 | 5761 | 5690 |
| F85625 | 1.036 | 0.94 | 0.94 | 4260 | 2249 | 2249 |
| B83029 | 1.036 | 1.27 | 1.27 | 4261 | 6193 | 6211 |
| P92017 | 1.036 | 1.17 | 1.19 | 4262 | 5254 | 5407 |
| J83043 | 1.036 | 1.05 | 1.05 | 4263 | 3685 | 3606 |
| L82051 | 1.036 | 1.10 | 1.10 | 4264 | 4269 | 4307 |
| D82096 | 1.036 | 1.12 | 1.12 | 4265 | 4538 | 4676 |
| N84028 | 1.035 | 1.29 | 1.27 | 4266 | 6377 | 6218 |
| G82600 | 1.035 | 1.18 | 1.20 | 4267 | 5368 | 5517 |
| F81041 | 1.035 | 1.16 | 1.16 | 4268 | 5067 | 5087 |
| F81674 | 1.035 | 0.96 | 0.96 | 4269 | 2446 | 2473 |
| F84669 | 1.035 | 1.05 | 1.04 | 4270 | 3697 | 3540 |
| H81057 | 1.035 | 1.05 | 1.04 | 4271 | 3663 | 3452 |
| K83044 | 1.035 | 1.11 | 1.11 | 4272 | 4506 | 4440 |
| F86045 | 1.035 | 1.02 | 1.04 | 4273 | 3180 | 3477 |
| M83140 | 1.035 | 1.20 | 1.20 | 4274 | 5563 | 5593 |
| G85121 | 1.035 | 0.97 | 0.98 | 4275 | 2567 | 2709 |
| B87602 | 1.035 | 1.16 | 1.19 | 4276 | 5141 | 5436 |
| J81041 | 1.035 | 1.10 | 1.11 | 4277 | 4390 | 4438 |
| J83052 | 1.035 | 0.97 | 0.96 | 4278 | 2534 | 2441 |
| M82019 | 1.034 | 1.05 | 1.06 | 4279 | 3622 | 3755 |
| P81733 | 1.034 | 1.04 | 1.02 | 4280 | 3523 | 3276 |
| B81088 | 1.034 | 1.11 | 1.11 | 4281 | 4481 | 4455 |
| J83024 | 1.034 | 1.26 | 1.25 | 4282 | 6150 | 6091 |
| P86001 | 1.034 | 1.20 | 1.21 | 4283 | 5545 | 5724 |
| K82018 | 1.034 | 1.08 | 1.05 | 4284 | 4013 | 3658 |
| K84052 | 1.034 | 1.02 | 1.01 | 4285 | 3128 | 3092 |
| L84036 | 1.034 | 1.08 | 1.09 | 4286 | 4089 | 4259 |
| K84075 | 1.034 | 0.98 | 0.95 | 4287 | 2674 | 2387 |
| J81640 | 1.034 | 1.29 | 1.27 | 4288 | 6391 | 6301 |
| P82002 | 1.034 | 1.18 | 1.19 | 4289 | 5416 | 5478 |
| F81157 | 1.034 | 1.84 | 1.80 | 4290 | 7308 | 7307 |
| J82208 | 1.034 | 1.12 | 1.12 | 4291 | 4639 | 4703 |
| B86064 | 1.034 | 1.09 | 1.11 | 4292 | 4211 | 4489 |
| L81050 | 1.033 | 1.02 | 1.02 | 4293 | 3232 | 3243 |
| N83054 | 1.033 | 1.10 | 1.12 | 4294 | 4384 | 4610 |

| | | | | | | |
|---|---|---|---|---|---|---|
| C87029 | 1.033 | 1.17 | 1.18 | 4295 | 5241 | 5317 |
| Y01163 | 1.033 | 1.19 | 1.19 | 4296 | 5517 | 5505 |
| E83613 | 1.033 | 1.09 | 1.06 | 4297 | 4168 | 3748 |
| G82810 | 1.033 | 1.23 | 1.23 | 4298 | 5842 | 5944 |
| L81103 | 1.033 | 1.06 | 1.05 | 4299 | 3749 | 3709 |
| L81655 | 1.033 | 0.92 | 0.91 | 4300 | 1983 | 1891 |
| P84030 | 1.033 | 1.05 | 1.07 | 4301 | 3595 | 3927 |
| N85006 | 1.033 | 1.05 | 1.05 | 4302 | 3661 | 3673 |
| E82009 | 1.033 | 0.96 | 0.95 | 4303 | 2499 | 2407 |
| M84063 | 1.032 | 1.00 | 1.00 | 4304 | 2996 | 2980 |
| P81027 | 1.032 | 1.21 | 1.21 | 4305 | 5735 | 5742 |
| K81645 | 1.032 | 1.03 | 1.05 | 4306 | 3425 | 3655 |
| C87604 | 1.032 | 1.12 | 1.12 | 4307 | 4586 | 4710 |
| L83123 | 1.032 | 1.17 | 1.19 | 4308 | 5276 | 5450 |
| G81016 | 1.032 | 1.16 | 1.14 | 4309 | 5160 | 4875 |
| P83612 | 1.031 | 1.13 | 1.13 | 4310 | 4785 | 4784 |
| E82652 | 1.031 | 1.06 | 1.05 | 4311 | 3877 | 3690 |
| N81043 | 1.031 | 1.11 | 1.09 | 4312 | 4474 | 4289 |
| K83017 | 1.031 | 1.13 | 1.12 | 4313 | 4825 | 4738 |
| D81053 | 1.031 | 1.10 | 1.10 | 4314 | 4316 | 4373 |
| A82027 | 1.031 | 1.14 | 1.11 | 4315 | 4975 | 4550 |
| G82036 | 1.031 | 1.10 | 1.11 | 4316 | 4395 | 4591 |
| J82669 | 1.031 | 1.00 | 1.03 | 4317 | 3040 | 3448 |
| E84678 | 1.031 | 0.97 | 0.96 | 4318 | 2646 | 2514 |
| K84003 | 1.031 | 0.98 | 0.98 | 4319 | 2725 | 2699 |
| L85023 | 1.030 | 1.02 | 1.00 | 4320 | 3257 | 2945 |
| J82154 | 1.030 | 1.11 | 1.09 | 4321 | 4518 | 4298 |
| M84603 | 1.030 | 1.14 | 1.13 | 4322 | 4914 | 4816 |
| H83018 | 1.030 | 1.00 | 0.99 | 4323 | 2992 | 2910 |
| M84031 | 1.030 | 1.03 | 1.02 | 4324 | 3435 | 3341 |
| G84625 | 1.030 | 0.96 | 0.95 | 4325 | 2473 | 2414 |
| G82064 | 1.030 | 1.08 | 1.09 | 4326 | 4084 | 4297 |
| M86014 | 1.030 | 1.02 | 1.02 | 4327 | 3316 | 3270 |
| L81082 | 1.030 | 1.00 | 1.01 | 4328 | 3053 | 3125 |
| M92030 | 1.030 | 1.24 | 1.25 | 4329 | 6039 | 6165 |
| M81604 | 1.030 | 1.15 | 1.13 | 4330 | 5016 | 4809 |
| F81046 | 1.030 | 1.16 | 1.17 | 4331 | 5147 | 5246 |
| G84023 | 1.029 | 1.07 | 1.06 | 4332 | 3988 | 3905 |
| F82647 | 1.029 | 1.06 | 1.07 | 4333 | 3896 | 4080 |
| J82054 | 1.029 | 1.00 | 1.00 | 4334 | 3014 | 3041 |
| K82034 | 1.029 | 1.12 | 1.09 | 4335 | 4628 | 4333 |
| L81649 | 1.029 | 0.89 | 0.89 | 4336 | 1723 | 1717 |
| P88026 | 1.029 | 1.06 | 1.06 | 4337 | 3862 | 3868 |
| P84004 | 1.029 | 1.15 | 1.16 | 4338 | 5052 | 5219 |
| H82096 | 1.029 | 1.22 | 1.23 | 4339 | 5806 | 5949 |
| E85034 | 1.029 | 1.10 | 1.10 | 4340 | 4456 | 4465 |
| C83005 | 1.029 | 1.31 | 1.33 | 4341 | 6611 | 6708 |
| N84006 | 1.029 | 1.20 | 1.20 | 4342 | 5656 | 5583 |
| F81040 | 1.028 | 1.19 | 1.18 | 4343 | 5521 | 5419 |
| B86019 | 1.028 | 0.97 | 0.99 | 4344 | 2672 | 2895 |

| | | | | | | |
|---|---|---|---|---|---|---|
| C81066 | 1.028 | 1.05 | 1.03 | 4345 | 3656 | 3515 |
| A87600 | 1.028 | 1.08 | 1.10 | 4346 | 4140 | 4378 |
| M85034 | 1.028 | 1.04 | 1.03 | 4347 | 3515 | 3421 |
| B85058 | 1.028 | 1.11 | 1.11 | 4348 | 4497 | 4517 |
| H82066 | 1.028 | 1.16 | 1.15 | 4349 | 5255 | 5083 |
| C84060 | 1.028 | 1.21 | 1.23 | 4350 | 5746 | 5952 |
| K82062 | 1.028 | 1.13 | 1.11 | 4351 | 4798 | 4541 |
| K81046 | 1.028 | 0.99 | 0.97 | 4352 | 2935 | 2674 |
| M85733 | 1.028 | 1.10 | 1.09 | 4353 | 4424 | 4308 |
| P81182 | 1.028 | 1.23 | 1.28 | 4354 | 5894 | 6376 |
| G84029 | 1.027 | 1.09 | 1.08 | 4355 | 4347 | 4228 |
| J82128 | 1.027 | 1.11 | 1.12 | 4356 | 4578 | 4705 |
| H81009 | 1.027 | 1.04 | 1.03 | 4357 | 3666 | 3470 |
| B82013 | 1.027 | 1.02 | 1.02 | 4358 | 3270 | 3298 |
| J82064 | 1.027 | 1.07 | 1.04 | 4359 | 4015 | 3637 |
| M91611 | 1.027 | 1.23 | 1.23 | 4360 | 5906 | 5971 |
| N81086 | 1.027 | 0.95 | 0.94 | 4361 | 2375 | 2294 |
| C81041 | 1.027 | 1.16 | 1.16 | 4362 | 5167 | 5186 |
| F81738 | 1.027 | 0.95 | 0.95 | 4363 | 2427 | 2470 |
| M85154 | 1.027 | 1.17 | 1.16 | 4364 | 5374 | 5240 |
| F84708 | 1.026 | 0.93 | 0.92 | 4365 | 2218 | 2043 |
| D82042 | 1.026 | 1.11 | 1.10 | 4366 | 4572 | 4458 |
| B87022 | 1.026 | 1.27 | 1.28 | 4367 | 6363 | 6436 |
| A83057 | 1.026 | 1.38 | 1.36 | 4368 | 6932 | 6874 |
| B83044 | 1.026 | 1.21 | 1.22 | 4369 | 5764 | 5807 |
| J82065 | 1.026 | 1.02 | 1.00 | 4370 | 3320 | 3116 |
| F82646 | 1.026 | 1.07 | 1.07 | 4371 | 4112 | 4064 |
| K83040 | 1.026 | 1.11 | 1.12 | 4372 | 4579 | 4746 |
| C82063 | 1.026 | 1.17 | 1.17 | 4373 | 5388 | 5374 |
| F85654 | 1.026 | 1.16 | 1.14 | 4374 | 5247 | 4988 |
| M89002 | 1.026 | 1.27 | 1.22 | 4375 | 6322 | 5851 |
| C84003 | 1.025 | 1.10 | 1.10 | 4376 | 4465 | 4429 |
| C85019 | 1.025 | 1.19 | 1.20 | 4377 | 5587 | 5662 |
| F86001 | 1.025 | 1.00 | 1.01 | 4378 | 3032 | 3267 |
| B82034 | 1.025 | 1.05 | 1.05 | 4379 | 3850 | 3793 |
| L81063 | 1.025 | 1.12 | 1.11 | 4380 | 4682 | 4567 |
| B85031 | 1.025 | 1.09 | 1.10 | 4381 | 4381 | 4445 |
| L82054 | 1.025 | 1.04 | 1.04 | 4382 | 3612 | 3715 |
| H81016 | 1.025 | 1.02 | 0.99 | 4383 | 3328 | 2985 |
| L81022 | 1.025 | 1.07 | 1.07 | 4384 | 4012 | 4137 |
| D83047 | 1.025 | 1.14 | 1.14 | 4385 | 5044 | 5026 |
| J82144 | 1.025 | 1.01 | 0.99 | 4386 | 3147 | 2973 |
| F86086 | 1.024 | 0.92 | 0.94 | 4387 | 2158 | 2385 |
| L85028 | 1.024 | 1.31 | 1.30 | 4388 | 6614 | 6565 |
| C88057 | 1.024 | 1.14 | 1.16 | 4389 | 4996 | 5211 |
| M91018 | 1.024 | 1.27 | 1.27 | 4390 | 6305 | 6312 |
| A83622 | 1.024 | 1.54 | 1.52 | 4391 | 7226 | 7211 |
| M83048 | 1.024 | 1.08 | 1.04 | 4392 | 4226 | 3669 |
| J82062 | 1.024 | 1.20 | 1.21 | 4393 | 5728 | 5804 |
| M83027 | 1.024 | 1.11 | 1.07 | 4394 | 4633 | 4156 |

| | | | | | | |
|---|---|---|---|---|---|---|
| P92034 | 1.024 | 1.21 | 1.21 | 4395 | 5768 | 5831 |
| C83075 | 1.024 | 1.07 | 1.06 | 4396 | 4125 | 3954 |
| L85060 | 1.024 | 1.20 | 1.18 | 4397 | 5673 | 5418 |
| M84021 | 1.024 | 1.00 | 1.00 | 4398 | 3049 | 3157 |
| F85679 | 1.024 | 1.00 | 1.01 | 4399 | 3106 | 3257 |
| G82727 | 1.024 | 1.08 | 1.10 | 4400 | 4205 | 4530 |
| N81034 | 1.024 | 1.04 | 1.03 | 4401 | 3722 | 3475 |
| C86605 | 1.024 | 1.24 | 1.26 | 4402 | 6064 | 6253 |
| Y00278 | 1.024 | 1.19 | 1.20 | 4403 | 5603 | 5708 |
| M85038 | 1.024 | 1.01 | 1.01 | 4404 | 3260 | 3281 |
| K83625 | 1.023 | 0.99 | 0.99 | 4405 | 2970 | 2961 |
| A83035 | 1.023 | 1.19 | 1.20 | 4406 | 5569 | 5637 |
| C87621 | 1.023 | 1.13 | 1.15 | 4407 | 4892 | 5053 |
| L84060 | 1.023 | 1.13 | 1.12 | 4408 | 4845 | 4789 |
| M81024 | 1.023 | 1.06 | 1.05 | 4409 | 3985 | 3871 |
| E85130 | 1.023 | 0.96 | 0.97 | 4410 | 2652 | 2665 |
| C81084 | 1.023 | 1.15 | 1.14 | 4411 | 5174 | 5043 |
| L83116 | 1.023 | 1.14 | 1.11 | 4412 | 4974 | 4577 |
| H83022 | 1.023 | 1.05 | 1.04 | 4413 | 3798 | 3680 |
| B86042 | 1.023 | 1.20 | 1.20 | 4414 | 5678 | 5737 |
| J82001 | 1.023 | 1.05 | 1.07 | 4415 | 3868 | 4041 |
| M92043 | 1.022 | 1.15 | 1.17 | 4416 | 5185 | 5350 |
| H84031 | 1.022 | 1.00 | 0.98 | 4417 | 3073 | 2809 |
| M92640 | 1.022 | 0.95 | 0.97 | 4418 | 2484 | 2668 |
| P88021 | 1.022 | 1.20 | 1.20 | 4419 | 5639 | 5656 |
| H81067 | 1.022 | 0.90 | 0.89 | 4420 | 1937 | 1852 |
| F84092 | 1.022 | 0.97 | 0.95 | 4421 | 2670 | 2529 |
| L84048 | 1.022 | 1.10 | 1.07 | 4422 | 4570 | 4107 |
| L84017 | 1.022 | 1.11 | 1.10 | 4423 | 4664 | 4456 |
| G85682 | 1.022 | 1.15 | 1.16 | 4424 | 5195 | 5323 |
| G84015 | 1.022 | 0.98 | 0.98 | 4425 | 2810 | 2781 |
| G85097 | 1.022 | 1.02 | 1.02 | 4426 | 3384 | 3395 |
| B82061 | 1.022 | 1.21 | 1.16 | 4427 | 5776 | 5328 |
| C82079 | 1.022 | 1.07 | 1.07 | 4428 | 4096 | 4100 |
| H83626 | 1.022 | 1.04 | 1.03 | 4429 | 3653 | 3528 |
| H81641 | 1.021 | 1.04 | 1.05 | 4430 | 3725 | 3817 |
| M87041 | 1.021 | 1.13 | 1.12 | 4431 | 4922 | 4840 |
| K84038 | 1.021 | 1.11 | 1.10 | 4432 | 4668 | 4511 |
| F84661 | 1.021 | 1.06 | 1.04 | 4433 | 3997 | 3713 |
| M83678 | 1.021 | 1.21 | 1.25 | 4434 | 5774 | 6208 |
| P81042 | 1.021 | 1.37 | 1.40 | 4435 | 6920 | 7014 |
| D83036 | 1.021 | 1.12 | 1.13 | 4436 | 4801 | 4900 |
| F81043 | 1.021 | 1.05 | 1.03 | 4437 | 3860 | 3576 |
| B81644 | 1.021 | 1.04 | 1.08 | 4438 | 3679 | 4257 |
| F86038 | 1.020 | 0.96 | 0.98 | 4439 | 2653 | 2918 |
| E84062 | 1.020 | 1.05 | 1.03 | 4440 | 3833 | 3523 |
| J83028 | 1.020 | 1.08 | 1.07 | 4441 | 4256 | 4122 |
| G81065 | 1.020 | 1.11 | 1.13 | 4442 | 4723 | 4940 |
| L81072 | 1.020 | 1.02 | 1.00 | 4443 | 3326 | 3197 |
| D81051 | 1.020 | 1.07 | 1.07 | 4444 | 4203 | 4141 |

| | | | | | | |
|---|---|---|---|---|---|---|
| F82674 | 1.020 | 0.99 | 0.99 | 4445 | 2957 | 2993 |
| D82045 | 1.020 | 1.10 | 1.09 | 4446 | 4516 | 4408 |
| G82129 | 1.020 | 1.01 | 1.03 | 4447 | 3205 | 3580 |
| M85033 | 1.020 | 1.07 | 1.06 | 4448 | 4141 | 4067 |
| F81089 | 1.019 | 1.07 | 1.05 | 4449 | 4128 | 3841 |
| G84011 | 1.019 | 0.95 | 0.95 | 4450 | 2496 | 2494 |
| L85014 | 1.019 | 1.08 | 1.04 | 4451 | 4247 | 3782 |
| C84133 | 1.019 | 1.10 | 1.08 | 4452 | 4588 | 4358 |
| F86658 | 1.019 | 0.98 | 0.97 | 4453 | 2896 | 2724 |
| B83621 | 1.019 | 1.12 | 1.14 | 4454 | 4759 | 4998 |
| M82011 | 1.019 | 1.21 | 1.21 | 4455 | 5799 | 5800 |
| M81074 | 1.019 | 1.22 | 1.20 | 4456 | 5882 | 5700 |
| D83057 | 1.019 | 1.03 | 1.03 | 4457 | 3509 | 3631 |
| L85015 | 1.019 | 1.08 | 1.08 | 4458 | 4268 | 4315 |
| M85068 | 1.018 | 1.04 | 1.03 | 4459 | 3657 | 3641 |
| E84020 | 1.018 | 1.22 | 1.19 | 4460 | 5940 | 5666 |
| F81116 | 1.018 | 1.19 | 1.19 | 4461 | 5581 | 5663 |
| M91643 | 1.018 | 1.18 | 1.20 | 4462 | 5528 | 5739 |
| K84015 | 1.018 | 1.06 | 1.03 | 4463 | 3966 | 3627 |
| G82039 | 1.018 | 1.09 | 1.11 | 4464 | 4450 | 4674 |
| J82029 | 1.018 | 1.31 | 1.29 | 4465 | 6647 | 6541 |
| M83129 | 1.018 | 1.13 | 1.16 | 4466 | 4940 | 5330 |
| A83034 | 1.018 | 1.11 | 1.12 | 4467 | 4777 | 4847 |
| F81613 | 1.017 | 1.22 | 1.22 | 4468 | 5903 | 5948 |
| C81115 | 1.017 | 1.33 | 1.29 | 4469 | 6787 | 6564 |
| H81077 | 1.017 | 1.02 | 0.98 | 4470 | 3423 | 2915 |
| F81149 | 1.017 | 1.07 | 1.07 | 4471 | 4163 | 4153 |
| J82119 | 1.017 | 1.06 | 1.04 | 4472 | 4069 | 3703 |
| B82622 | 1.017 | 1.14 | 1.13 | 4473 | 5130 | 4955 |
| F86627 | 1.017 | 1.02 | 1.03 | 4474 | 3397 | 3660 |
| M84046 | 1.017 | 1.00 | 1.00 | 4475 | 3208 | 3168 |
| L85608 | 1.017 | 1.37 | 1.34 | 4476 | 6942 | 6842 |
| A88025 | 1.017 | 1.22 | 1.21 | 4477 | 5887 | 5885 |
| N83633 | 1.017 | 1.32 | 1.33 | 4478 | 6746 | 6767 |
| E82041 | 1.017 | 0.99 | 1.00 | 4479 | 3042 | 3178 |
| Y00105 | 1.017 | 1.05 | 1.02 | 4480 | 3851 | 3547 |
| E81037 | 1.017 | 1.11 | 1.10 | 4481 | 4786 | 4656 |
| K84020 | 1.017 | 1.02 | 1.00 | 4482 | 3487 | 3211 |
| F85017 | 1.017 | 1.01 | 1.02 | 4483 | 3294 | 3420 |
| C84010 | 1.016 | 1.07 | 1.05 | 4484 | 4216 | 3930 |
| B82018 | 1.016 | 1.00 | 0.99 | 4485 | 3200 | 3118 |
| N84003 | 1.016 | 1.13 | 1.13 | 4486 | 4970 | 4996 |
| G85034 | 1.016 | 0.99 | 0.99 | 4487 | 3069 | 3073 |
| C84049 | 1.016 | 1.05 | 1.05 | 4488 | 3931 | 3933 |
| Y02884 | 1.016 | 1.11 | 1.13 | 4489 | 4744 | 4932 |
| P81028 | 1.016 | 1.09 | 1.11 | 4490 | 4410 | 4708 |
| D83037 | 1.016 | 1.10 | 1.08 | 4491 | 4599 | 4275 |
| K83022 | 1.016 | 1.09 | 1.06 | 4492 | 4406 | 4006 |
| M86001 | 1.016 | 1.19 | 1.17 | 4493 | 5698 | 5472 |
| M88004 | 1.016 | 1.24 | 1.24 | 4494 | 6117 | 6197 |

| | | | | | | |
|---|---|---|---|---|---|---|
| E81030 | 1.016 | 1.32 | 1.31 | 4495 | 6711 | 6681 |
| L83031 | 1.015 | 1.16 | 1.15 | 4496 | 5342 | 5210 |
| P81036 | 1.015 | 1.24 | 1.29 | 4497 | 6166 | 6591 |
| C81081 | 1.015 | 1.20 | 1.18 | 4498 | 5718 | 5552 |
| H83014 | 1.015 | 1.03 | 1.02 | 4499 | 3609 | 3491 |
| B83027 | 1.015 | 1.14 | 1.15 | 4500 | 5111 | 5237 |
| E82098 | 1.015 | 1.02 | 1.03 | 4501 | 3477 | 3623 |
| E85001 | 1.015 | 1.05 | 1.04 | 4502 | 3911 | 3787 |
| M81057 | 1.015 | 1.21 | 1.19 | 4503 | 5826 | 5703 |
| A82049 | 1.015 | 1.16 | 1.16 | 4504 | 5311 | 5341 |
| L81019 | 1.015 | 1.02 | 1.01 | 4505 | 3411 | 3345 |
| D81618 | 1.015 | 1.06 | 1.07 | 4506 | 4119 | 4258 |
| M81081 | 1.015 | 1.09 | 1.07 | 4507 | 4523 | 4217 |
| L85003 | 1.015 | 1.14 | 1.13 | 4508 | 5152 | 4974 |
| J83615 | 1.015 | 1.09 | 1.08 | 4509 | 4510 | 4364 |
| M81041 | 1.015 | 1.13 | 1.15 | 4510 | 5053 | 5231 |
| G85123 | 1.015 | 0.93 | 0.94 | 4511 | 2283 | 2408 |
| J82203 | 1.015 | 1.03 | 1.04 | 4512 | 3617 | 3766 |
| E82123 | 1.015 | 1.07 | 1.07 | 4513 | 4171 | 4169 |
| G82071 | 1.015 | 1.12 | 1.12 | 4514 | 4824 | 4922 |
| F81165 | 1.014 | 0.91 | 0.90 | 4515 | 2121 | 1975 |
| G81035 | 1.014 | 1.05 | 1.03 | 4516 | 3921 | 3704 |
| H81087 | 1.014 | 1.08 | 1.07 | 4517 | 4358 | 4188 |
| M86003 | 1.014 | 1.02 | 1.02 | 4518 | 3530 | 3472 |
| K84065 | 1.014 | 1.03 | 1.04 | 4519 | 3724 | 3738 |
| B81051 | 1.014 | 1.02 | 1.03 | 4520 | 3575 | 3657 |
| G81099 | 1.014 | 1.15 | 1.13 | 4521 | 5277 | 5036 |
| M81064 | 1.014 | 1.08 | 1.05 | 4522 | 4413 | 4018 |
| L85031 | 1.013 | 1.13 | 1.14 | 4523 | 5017 | 5051 |
| B82044 | 1.013 | 1.04 | 1.04 | 4524 | 3772 | 3887 |
| L83637 | 1.013 | 1.31 | 1.30 | 4525 | 6677 | 6656 |
| G82025 | 1.013 | 1.00 | 1.00 | 4526 | 3249 | 3309 |
| B83016 | 1.013 | 1.28 | 1.28 | 4527 | 6484 | 6503 |
| B85021 | 1.013 | 1.24 | 1.24 | 4528 | 6195 | 6209 |
| H82059 | 1.012 | 1.17 | 1.16 | 4529 | 5496 | 5397 |
| D82019 | 1.012 | 1.25 | 1.26 | 4530 | 6251 | 6361 |
| F85032 | 1.012 | 0.98 | 0.98 | 4531 | 2995 | 3007 |
| M84612 | 1.012 | 1.11 | 1.06 | 4532 | 4821 | 4161 |
| P91631 | 1.012 | 0.98 | 0.99 | 4533 | 2954 | 3088 |
| E82025 | 1.012 | 1.00 | 0.98 | 4534 | 3245 | 3016 |
| M84002 | 1.012 | 1.11 | 1.12 | 4535 | 4838 | 4895 |
| F82614 | 1.012 | 1.04 | 1.04 | 4536 | 3841 | 3773 |
| G85119 | 1.012 | 1.08 | 1.07 | 4537 | 4318 | 4252 |
| D83013 | 1.012 | 1.15 | 1.14 | 4538 | 5302 | 5196 |
| G83009 | 1.012 | 1.00 | 0.99 | 4539 | 3238 | 3162 |
| M82045 | 1.012 | 1.22 | 1.21 | 4540 | 5934 | 5940 |
| M89026 | 1.012 | 1.08 | 1.05 | 4541 | 4422 | 3932 |
| N85025 | 1.011 | 1.11 | 1.10 | 4542 | 4766 | 4701 |
| B87029 | 1.011 | 1.19 | 1.21 | 4543 | 5679 | 5890 |
| L85038 | 1.011 | 1.12 | 1.09 | 4544 | 4930 | 4510 |

| | | | | | | |
|---|---|---|---|---|---|---|
| P84652 | 1.011 | 1.03 | 1.05 | 4545 | 3635 | 4021 |
| G82230 | 1.011 | 0.99 | 1.02 | 4546 | 3138 | 3535 |
| M84041 | 1.011 | 1.12 | 1.12 | 4547 | 4942 | 4927 |
| M82032 | 1.011 | 1.06 | 1.06 | 4548 | 4093 | 4132 |
| G84035 | 1.011 | 0.99 | 1.00 | 4549 | 3160 | 3225 |
| G82120 | 1.011 | 0.98 | 0.98 | 4550 | 3007 | 2947 |
| E82077 | 1.011 | 0.93 | 0.92 | 4551 | 2336 | 2280 |
| G84020 | 1.011 | 1.02 | 1.01 | 4552 | 3548 | 3447 |
| P81092 | 1.011 | 1.39 | 1.43 | 4553 | 7041 | 7119 |
| A82014 | 1.011 | 1.15 | 1.16 | 4554 | 5325 | 5424 |
| H81036 | 1.010 | 0.95 | 0.95 | 4555 | 2621 | 2673 |
| H84040 | 1.010 | 0.97 | 0.95 | 4556 | 2900 | 2677 |
| G85026 | 1.010 | 1.03 | 1.04 | 4557 | 3702 | 3855 |
| P88008 | 1.010 | 1.11 | 1.11 | 4558 | 4857 | 4866 |
| C82062 | 1.010 | 0.99 | 1.00 | 4559 | 3141 | 3296 |
| L83007 | 1.010 | 1.24 | 1.20 | 4560 | 6173 | 5832 |
| B82006 | 1.010 | 1.08 | 1.07 | 4561 | 4471 | 4311 |
| J83004 | 1.010 | 1.02 | 1.02 | 4562 | 3560 | 3496 |
| P86624 | 1.010 | 1.22 | 1.23 | 4563 | 6046 | 6164 |
| P82607 | 1.010 | 1.22 | 1.23 | 4564 | 6053 | 6097 |
| C82109 | 1.010 | 1.03 | 1.02 | 4565 | 3769 | 3572 |
| N85002 | 1.010 | 1.09 | 1.08 | 4566 | 4530 | 4468 |
| E82626 | 1.009 | 1.03 | 1.02 | 4567 | 3744 | 3610 |
| D81030 | 1.009 | 1.13 | 1.13 | 4568 | 5121 | 4994 |
| N84011 | 1.009 | 1.20 | 1.19 | 4569 | 5816 | 5697 |
| E81064 | 1.009 | 0.96 | 0.97 | 4570 | 2814 | 2942 |
| K81101 | 1.009 | 1.01 | 1.01 | 4571 | 3414 | 3479 |
| C86613 | 1.009 | 1.21 | 1.23 | 4572 | 5963 | 6162 |
| G81104 | 1.009 | 1.14 | 1.16 | 4573 | 5237 | 5415 |
| M86007 | 1.009 | 1.14 | 1.13 | 4574 | 5163 | 5009 |
| H83015 | 1.009 | 0.98 | 0.97 | 4575 | 2975 | 2921 |
| F81085 | 1.009 | 1.01 | 0.97 | 4576 | 3451 | 2821 |
| M88026 | 1.009 | 1.20 | 1.20 | 4577 | 5810 | 5843 |
| C82627 | 1.009 | 1.16 | 1.16 | 4578 | 5405 | 5411 |
| J81616 | 1.008 | 1.01 | 1.00 | 4579 | 3439 | 3249 |
| F82607 | 1.008 | 0.98 | 0.98 | 4580 | 2991 | 3027 |
| B82025 | 1.008 | 1.11 | 1.13 | 4581 | 4846 | 5082 |
| K82079 | 1.008 | 1.08 | 1.05 | 4582 | 4393 | 4038 |
| G82631 | 1.008 | 1.02 | 1.05 | 4583 | 3559 | 3952 |
| B85026 | 1.008 | 1.01 | 1.02 | 4584 | 3503 | 3628 |
| G84032 | 1.008 | 1.09 | 1.08 | 4585 | 4548 | 4459 |
| M85042 | 1.008 | 1.00 | 0.99 | 4586 | 3223 | 3232 |
| P81079 | 1.008 | 1.12 | 1.12 | 4587 | 4971 | 4990 |
| B82029 | 1.008 | 1.06 | 1.05 | 4588 | 4150 | 4068 |
| F86032 | 1.008 | 0.97 | 0.96 | 4589 | 2921 | 2732 |
| E83038 | 1.008 | 1.14 | 1.11 | 4590 | 5223 | 4846 |
| E85015 | 1.008 | 1.16 | 1.16 | 4591 | 5479 | 5389 |
| K82074 | 1.007 | 0.98 | 0.98 | 4592 | 3039 | 2997 |
| K82073 | 1.007 | 1.06 | 1.03 | 4593 | 4115 | 3768 |
| P81642 | 1.007 | 1.08 | 1.07 | 4594 | 4439 | 4341 |

| | | | | | | |
|---|---|---|---|---|---|---|
| J83054 | 1.007 | 1.13 | 1.13 | 4595 | 5065 | 5118 |
| C84055 | 1.007 | 1.12 | 1.10 | 4596 | 4932 | 4680 |
| C84017 | 1.007 | 1.07 | 1.05 | 4597 | 4353 | 4088 |
| E85044 | 1.007 | 1.08 | 1.07 | 4598 | 4402 | 4330 |
| C86017 | 1.007 | 1.16 | 1.18 | 4599 | 5437 | 5691 |
| K82031 | 1.007 | 1.04 | 1.02 | 4600 | 3935 | 3630 |
| M83088 | 1.007 | 1.14 | 1.13 | 4601 | 5171 | 5071 |
| C84113 | 1.007 | 1.18 | 1.16 | 4602 | 5666 | 5479 |
| K81055 | 1.006 | 0.97 | 0.95 | 4603 | 2913 | 2678 |
| F81158 | 1.006 | 1.22 | 1.22 | 4604 | 6094 | 6060 |
| P81726 | 1.006 | 1.25 | 1.31 | 4605 | 6353 | 6715 |
| M82035 | 1.006 | 1.07 | 1.07 | 4606 | 4271 | 4361 |
| A82070 | 1.006 | 1.02 | 1.00 | 4607 | 3643 | 3322 |
| B82063 | 1.006 | 1.23 | 1.25 | 4608 | 6142 | 6311 |
| C81640 | 1.006 | 1.02 | 1.01 | 4609 | 3553 | 3482 |
| A88022 | 1.006 | 1.20 | 1.19 | 4610 | 5844 | 5764 |
| N82026 | 1.006 | 1.05 | 1.07 | 4611 | 4088 | 4384 |
| J81067 | 1.005 | 1.11 | 1.10 | 4612 | 4830 | 4751 |
| J82212 | 1.005 | 0.98 | 1.00 | 4613 | 3083 | 3311 |
| B87017 | 1.005 | 1.13 | 1.15 | 4614 | 5162 | 5402 |
| F81218 | 1.004 | 0.99 | 0.99 | 4615 | 3183 | 3297 |
| G83651 | 1.004 | 1.01 | 1.00 | 4616 | 3510 | 3428 |
| F86692 | 1.004 | 1.12 | 1.10 | 4617 | 5027 | 4817 |
| M84067 | 1.004 | 1.07 | 1.06 | 4618 | 4282 | 4185 |
| M81066 | 1.004 | 1.10 | 1.08 | 4619 | 4699 | 4543 |
| C81100 | 1.004 | 1.21 | 1.20 | 4620 | 5936 | 5880 |
| K82631 | 1.004 | 0.89 | 0.89 | 4621 | 2040 | 2026 |
| H83625 | 1.004 | 1.05 | 1.05 | 4622 | 4120 | 4031 |
| B85022 | 1.004 | 1.08 | 1.09 | 4623 | 4501 | 4570 |
| J81086 | 1.004 | 1.03 | 1.03 | 4624 | 3831 | 3883 |
| C84654 | 1.004 | 1.06 | 1.07 | 4625 | 4158 | 4385 |
| M82616 | 1.004 | 1.19 | 1.20 | 4626 | 5788 | 5866 |
| H82091 | 1.004 | 1.24 | 1.25 | 4627 | 6240 | 6365 |
| B82038 | 1.004 | 1.16 | 1.18 | 4628 | 5511 | 5695 |
| K81012 | 1.003 | 0.97 | 0.98 | 4629 | 2981 | 3047 |
| B82007 | 1.003 | 1.16 | 1.12 | 4630 | 5471 | 4970 |
| B83012 | 1.003 | 1.12 | 1.14 | 4631 | 5039 | 5192 |
| L81077 | 1.003 | 1.05 | 1.06 | 4632 | 4121 | 4232 |
| E84684 | 1.003 | 1.08 | 1.06 | 4633 | 4493 | 4282 |
| C88034 | 1.003 | 0.97 | 0.99 | 4634 | 2907 | 3227 |
| E85075 | 1.003 | 1.04 | 1.03 | 4635 | 3910 | 3843 |
| N82073 | 1.003 | 1.04 | 1.06 | 4636 | 3967 | 4272 |
| J83025 | 1.003 | 1.08 | 1.09 | 4637 | 4547 | 4678 |
| H84637 | 1.003 | 1.03 | 1.01 | 4638 | 3758 | 3559 |
| P88031 | 1.003 | 1.22 | 1.21 | 4639 | 6095 | 5977 |
| J82207 | 1.003 | 0.93 | 0.95 | 4640 | 2514 | 2698 |
| L81016 | 1.003 | 1.13 | 1.10 | 4641 | 5104 | 4838 |
| A88013 | 1.003 | 1.22 | 1.21 | 4642 | 6051 | 5990 |
| C84107 | 1.003 | 1.07 | 1.07 | 4643 | 4356 | 4381 |
| J81061 | 1.003 | 1.26 | 1.25 | 4644 | 6435 | 6344 |

| | | | | | | |
|---|---|---|---|---|---|---|
| E84056 | 1.002 | 1.09 | 1.07 | 4645 | 4611 | 4369 |
| M81042 | 1.002 | 1.09 | 1.06 | 4646 | 4670 | 4303 |
| K83019 | 1.002 | 1.04 | 1.04 | 4647 | 3953 | 3989 |
| P92005 | 1.002 | 1.22 | 1.23 | 4648 | 6118 | 6226 |
| D83021 | 1.002 | 1.09 | 1.08 | 4649 | 4607 | 4483 |
| E86605 | 1.002 | 1.04 | 1.03 | 4650 | 3984 | 3882 |
| C82119 | 1.002 | 1.03 | 1.03 | 4651 | 3872 | 3888 |
| M84616 | 1.002 | 1.12 | 1.11 | 4652 | 5006 | 4882 |
| F81640 | 1.002 | 1.12 | 1.12 | 4653 | 4983 | 5013 |
| P81655 | 1.002 | 1.01 | 1.00 | 4654 | 3564 | 3340 |
| N84020 | 1.002 | 1.07 | 1.07 | 4655 | 4382 | 4433 |
| J82051 | 1.002 | 1.02 | 1.01 | 4656 | 3741 | 3575 |
| D81630 | 1.002 | 0.88 | 0.89 | 4657 | 1944 | 2074 |
| P81754 | 1.001 | 1.19 | 1.22 | 4658 | 5803 | 6120 |
| B81020 | 1.001 | 1.14 | 1.18 | 4659 | 5248 | 5715 |
| M85732 | 1.001 | 1.25 | 1.22 | 4660 | 6410 | 6119 |
| P81069 | 1.001 | 1.11 | 1.09 | 4661 | 4906 | 4718 |
| J82625 | 1.001 | 1.02 | 1.02 | 4662 | 3686 | 3691 |
| J82035 | 1.001 | 0.97 | 0.94 | 4663 | 2930 | 2664 |
| G82687 | 1.001 | 1.21 | 1.22 | 4664 | 5971 | 6067 |
| E82081 | 1.001 | 0.97 | 0.98 | 4665 | 2925 | 3076 |
| H83627 | 1.001 | 0.92 | 0.92 | 4666 | 2409 | 2379 |
| H83631 | 1.001 | 0.98 | 0.97 | 4667 | 3055 | 2986 |
| L81046 | 1.001 | 1.04 | 1.03 | 4668 | 3950 | 3872 |
| J82188 | 1.001 | 0.98 | 0.96 | 4669 | 3050 | 2900 |
| J82016 | 1.001 | 1.00 | 0.99 | 4670 | 3417 | 3319 |
| H84043 | 1.001 | 0.88 | 0.86 | 4671 | 1941 | 1786 |
| A89002 | 1.001 | 1.27 | 1.27 | 4672 | 6493 | 6499 |
| F84031 | 1.000 | 1.15 | 1.15 | 4673 | 5455 | 5351 |
| C84048 | 1.000 | 1.09 | 1.08 | 4674 | 4735 | 4495 |
| J83038 | 1.000 | 1.23 | 1.22 | 4675 | 6159 | 6135 |
| P81055 | 1.000 | 1.11 | 1.09 | 4676 | 4963 | 4667 |
| C81090 | 1.000 | 1.24 | 1.21 | 4677 | 6313 | 6042 |
| J82220 | 1.000 | 0.88 | 0.87 | 4678 | 1955 | 1848 |
| B81665 | 1.000 | 1.08 | 1.11 | 4679 | 4507 | 4877 |
| H83004 | 1.000 | 1.00 | 1.00 | 4680 | 3418 | 3368 |
| D81637 | 1.000 | 0.86 | 0.86 | 4681 | 1795 | 1763 |
| A86029 | 1.000 | 1.14 | 1.14 | 4682 | 5320 | 5336 |
| C88015 | 1.000 | 1.21 | 1.23 | 4683 | 6018 | 6171 |
| G84010 | 1.000 | 1.00 | 0.99 | 4684 | 3359 | 3335 |
| M87002 | 0.999 | 1.13 | 1.13 | 4685 | 5187 | 5114 |
| M89017 | 0.999 | 1.10 | 1.07 | 4686 | 4843 | 4377 |
| M88020 | 0.999 | 1.04 | 1.04 | 4687 | 4032 | 3974 |
| C84691 | 0.999 | 0.92 | 0.95 | 4688 | 2433 | 2715 |
| L83086 | 0.999 | 1.16 | 1.16 | 4689 | 5504 | 5544 |
| L85054 | 0.999 | 1.14 | 1.14 | 4690 | 5335 | 5327 |
| B81041 | 0.999 | 1.15 | 1.14 | 4691 | 5377 | 5290 |
| D82073 | 0.999 | 1.10 | 1.11 | 4692 | 4833 | 4950 |
| F85681 | 0.998 | 0.90 | 0.90 | 4693 | 2159 | 2167 |
| N81051 | 0.998 | 1.15 | 1.12 | 4694 | 5431 | 5092 |

| | | | | | | |
|---|---|---|---|---|---|---|
| E85708 | 0.998 | 0.94 | 0.93 | 4695 | 2618 | 2530 |
| N81077 | 0.998 | 1.09 | 1.08 | 4696 | 4745 | 4605 |
| E85088 | 0.998 | 1.16 | 1.15 | 4697 | 5494 | 5434 |
| N81026 | 0.998 | 1.06 | 1.05 | 4698 | 4274 | 4164 |
| C82025 | 0.998 | 1.11 | 1.09 | 4699 | 5020 | 4712 |
| Y03222 | 0.998 | 1.38 | 1.36 | 4700 | 7030 | 6969 |
| F82661 | 0.998 | 0.92 | 0.93 | 4701 | 2358 | 2517 |
| E84646 | 0.998 | 0.87 | 0.86 | 4702 | 1904 | 1743 |
| P84023 | 0.998 | 0.94 | 0.96 | 4703 | 2665 | 2950 |
| H82067 | 0.997 | 1.17 | 1.18 | 4704 | 5660 | 5760 |
| P85021 | 0.997 | 1.16 | 1.18 | 4705 | 5575 | 5780 |
| P81104 | 0.997 | 1.25 | 1.26 | 4706 | 6417 | 6522 |
| M83059 | 0.997 | 1.02 | 0.99 | 4707 | 3773 | 3348 |
| C86623 | 0.997 | 1.40 | 1.41 | 4708 | 7082 | 7111 |
| M83102 | 0.997 | 1.13 | 1.16 | 4709 | 5250 | 5559 |
| M82040 | 0.997 | 1.00 | 1.00 | 4710 | 3382 | 3460 |
| C81021 | 0.997 | 1.15 | 1.12 | 4711 | 5409 | 5066 |
| P81730 | 0.997 | 1.12 | 1.17 | 4712 | 5156 | 5601 |
| E85098 | 0.997 | 0.96 | 0.97 | 4713 | 2895 | 2999 |
| K84055 | 0.997 | 1.03 | 1.03 | 4714 | 3904 | 3829 |
| C84120 | 0.997 | 1.06 | 1.06 | 4715 | 4257 | 4292 |
| H82056 | 0.997 | 0.97 | 0.97 | 4716 | 3008 | 3060 |
| C81099 | 0.997 | 1.18 | 1.17 | 4717 | 5724 | 5631 |
| J82056 | 0.997 | 1.13 | 1.11 | 4718 | 5191 | 4977 |
| J82174 | 0.997 | 0.97 | 0.96 | 4719 | 3081 | 2977 |
| M85113 | 0.997 | 1.12 | 1.11 | 4720 | 5082 | 4916 |
| M88624 | 0.997 | 1.07 | 1.09 | 4721 | 4508 | 4688 |
| N84007 | 0.996 | 1.23 | 1.21 | 4722 | 6184 | 6081 |
| F81690 | 0.996 | 1.20 | 1.17 | 4723 | 5979 | 5599 |
| B86008 | 0.996 | 1.02 | 1.04 | 4724 | 3698 | 4015 |
| E85745 | 0.996 | 1.11 | 1.11 | 4725 | 4955 | 4997 |
| P81065 | 0.996 | 1.09 | 1.12 | 4726 | 4693 | 5105 |
| B85002 | 0.996 | 1.08 | 1.08 | 4727 | 4582 | 4629 |
| N83055 | 0.996 | 1.25 | 1.25 | 4728 | 6423 | 6416 |
| B81084 | 0.996 | 1.12 | 1.12 | 4729 | 5080 | 5076 |
| C81654 | 0.996 | 1.05 | 1.04 | 4730 | 4173 | 4098 |
| L83041 | 0.996 | 1.08 | 1.07 | 4731 | 4550 | 4466 |
| B82050 | 0.996 | 1.13 | 1.12 | 4732 | 5198 | 5143 |
| M83639 | 0.996 | 1.30 | 1.34 | 4733 | 6751 | 6918 |
| M81008 | 0.996 | 1.12 | 1.12 | 4734 | 5064 | 5100 |
| H85686 | 0.996 | 0.93 | 0.92 | 4735 | 2531 | 2505 |
| G85024 | 0.996 | 1.17 | 1.17 | 4736 | 5615 | 5613 |
| G82031 | 0.996 | 1.00 | 1.00 | 4737 | 3458 | 3548 |
| A82003 | 0.996 | 1.26 | 1.22 | 4738 | 6488 | 6117 |
| J81020 | 0.996 | 1.17 | 1.13 | 4739 | 5658 | 5271 |
| C86012 | 0.995 | 1.12 | 1.14 | 4740 | 5116 | 5372 |
| C86037 | 0.995 | 1.23 | 1.25 | 4741 | 6264 | 6455 |
| L85612 | 0.995 | 1.09 | 1.08 | 4742 | 4674 | 4685 |
| A83005 | 0.995 | 1.27 | 1.26 | 4743 | 6558 | 6525 |
| G82133 | 0.995 | 1.06 | 1.08 | 4744 | 4327 | 4670 |

| | | | | | | |
|---|---|---|---|---|---|---|
| K84031 | 0.995 | 1.06 | 1.04 | 4745 | 4324 | 4152 |
| C82123 | 0.995 | 1.04 | 1.03 | 4746 | 4060 | 3977 |
| H85035 | 0.995 | 1.04 | 1.03 | 4747 | 4103 | 3896 |
| A82021 | 0.995 | 1.15 | 1.16 | 4748 | 5422 | 5550 |
| G81031 | 0.994 | 1.06 | 1.10 | 4749 | 4398 | 4886 |
| J84005 | 0.994 | 1.06 | 1.06 | 4750 | 4287 | 4363 |
| M84040 | 0.994 | 1.02 | 1.02 | 4751 | 3776 | 3733 |
| F81057 | 0.994 | 0.98 | 0.98 | 4752 | 3153 | 3190 |
| H85007 | 0.994 | 0.92 | 0.90 | 4753 | 2459 | 2233 |
| E81029 | 0.994 | 1.12 | 1.12 | 4754 | 5197 | 5090 |
| G84629 | 0.994 | 0.95 | 0.95 | 4755 | 2854 | 2796 |
| M81084 | 0.994 | 1.01 | 0.99 | 4756 | 3726 | 3432 |
| C81118 | 0.993 | 1.24 | 1.25 | 4757 | 6376 | 6450 |
| Y00252 | 0.993 | 1.15 | 1.15 | 4758 | 5461 | 5502 |
| J81053 | 0.993 | 1.14 | 1.12 | 4759 | 5349 | 5047 |
| M85098 | 0.993 | 0.97 | 0.97 | 4760 | 3107 | 3091 |
| A88601 | 0.993 | 1.39 | 1.35 | 4761 | 7068 | 6951 |
| G85036 | 0.993 | 1.04 | 1.05 | 4762 | 4087 | 4202 |
| F81007 | 0.993 | 1.15 | 1.13 | 4763 | 5520 | 5213 |
| J84020 | 0.993 | 1.11 | 1.11 | 4764 | 4991 | 4975 |
| H83016 | 0.993 | 1.00 | 0.99 | 4765 | 3512 | 3417 |
| E84017 | 0.993 | 1.05 | 1.03 | 4766 | 4183 | 4002 |
| F81101 | 0.993 | 0.98 | 0.98 | 4767 | 3224 | 3288 |
| L83085 | 0.993 | 1.11 | 1.13 | 4768 | 5040 | 5256 |
| M91637 | 0.993 | 1.20 | 1.21 | 4769 | 6009 | 6077 |
| C81083 | 0.993 | 1.07 | 1.06 | 4770 | 4549 | 4328 |
| M82606 | 0.993 | 1.00 | 1.02 | 4771 | 3499 | 3765 |
| A82013 | 0.992 | 1.15 | 1.13 | 4772 | 5470 | 5262 |
| M87612 | 0.992 | 1.08 | 1.08 | 4773 | 4690 | 4719 |
| F81153 | 0.992 | 1.09 | 1.10 | 4774 | 4778 | 4854 |
| M85097 | 0.992 | 1.08 | 1.07 | 4775 | 4580 | 4475 |
| C82056 | 0.992 | 1.02 | 1.02 | 4776 | 3764 | 3764 |
| H85674 | 0.992 | 0.97 | 0.96 | 4777 | 3063 | 2983 |
| P89021 | 0.992 | 1.22 | 1.24 | 4778 | 6170 | 6333 |
| C84621 | 0.992 | 0.96 | 0.95 | 4779 | 3028 | 2852 |
| L83099 | 0.992 | 1.21 | 1.16 | 4780 | 6040 | 5573 |
| P87661 | 0.992 | 1.15 | 1.17 | 4781 | 5498 | 5752 |
| M88010 | 0.991 | 1.20 | 1.20 | 4782 | 5953 | 6034 |
| B87600 | 0.991 | 1.26 | 1.27 | 4783 | 6526 | 6614 |
| J82146 | 0.991 | 1.04 | 1.03 | 4784 | 4154 | 4019 |
| A82025 | 0.991 | 1.06 | 1.04 | 4785 | 4444 | 4073 |
| P81125 | 0.991 | 1.17 | 1.20 | 4786 | 5730 | 6009 |
| G84021 | 0.991 | 1.01 | 1.01 | 4787 | 3678 | 3701 |
| G82067 | 0.991 | 1.01 | 1.02 | 4788 | 3753 | 3832 |
| D83030 | 0.991 | 1.14 | 1.14 | 4789 | 5387 | 5337 |
| G82658 | 0.991 | 1.06 | 1.09 | 4790 | 4314 | 4803 |
| Y02900 | 0.991 | 0.84 | 0.84 | 4791 | 1625 | 1651 |
| P81778 | 0.991 | 1.30 | 1.32 | 4792 | 6758 | 6843 |
| F86036 | 0.991 | 0.93 | 0.95 | 4793 | 2613 | 2823 |
| C81114 | 0.991 | 1.05 | 1.03 | 4794 | 4259 | 3935 |

| | | | | | | |
|---|---|---|---|---|---|---|
| L83098 | 0.990 | 1.11 | 1.13 | 4795 | 5041 | 5239 |
| K82610 | 0.990 | 1.05 | 1.04 | 4796 | 4213 | 4078 |
| H85662 | 0.990 | 0.88 | 0.88 | 4797 | 2074 | 2046 |
| E83035 | 0.990 | 1.04 | 1.02 | 4798 | 4139 | 3848 |
| B86013 | 0.990 | 1.01 | 1.03 | 4799 | 3732 | 4029 |
| E84658 | 0.990 | 1.00 | 0.98 | 4800 | 3568 | 3263 |
| J81064 | 0.990 | 1.22 | 1.22 | 4801 | 6189 | 6167 |
| A83610 | 0.990 | 1.28 | 1.28 | 4802 | 6629 | 6636 |
| E82071 | 0.990 | 0.93 | 0.92 | 4803 | 2598 | 2537 |
| L81014 | 0.990 | 1.06 | 1.05 | 4804 | 4366 | 4320 |
| F81749 | 0.990 | 1.06 | 1.04 | 4805 | 4392 | 4143 |
| F84620 | 0.990 | 0.98 | 0.99 | 4806 | 3213 | 3431 |
| D82025 | 0.989 | 1.25 | 1.21 | 4807 | 6432 | 6160 |
| P84665 | 0.989 | 1.10 | 1.12 | 4808 | 4902 | 5168 |
| Y03587 | 0.989 | 0.98 | 0.99 | 4809 | 3267 | 3372 |
| J82103 | 0.989 | 1.09 | 1.07 | 4810 | 4844 | 4580 |
| F81109 | 0.989 | 0.96 | 0.97 | 4811 | 3046 | 3181 |
| M85689 | 0.989 | 1.07 | 1.09 | 4812 | 4541 | 4791 |
| P92038 | 0.989 | 1.05 | 1.06 | 4813 | 4229 | 4389 |
| K82004 | 0.989 | 0.99 | 0.97 | 4814 | 3440 | 3219 |
| M85133 | 0.989 | 1.25 | 1.25 | 4815 | 6466 | 6456 |
| J81040 | 0.989 | 1.22 | 1.21 | 4816 | 6209 | 6076 |
| M82006 | 0.989 | 1.20 | 1.19 | 4817 | 5992 | 5958 |
| C84037 | 0.988 | 1.09 | 1.09 | 4818 | 4859 | 4822 |
| M83138 | 0.988 | 1.04 | 1.08 | 4819 | 4210 | 4749 |
| C84693 | 0.988 | 1.08 | 1.11 | 4820 | 4753 | 5018 |
| P92033 | 0.988 | 1.13 | 1.14 | 4821 | 5285 | 5387 |
| J82093 | 0.988 | 1.24 | 1.22 | 4822 | 6426 | 6250 |
| E85003 | 0.987 | 0.89 | 0.91 | 4823 | 2216 | 2451 |
| N85014 | 0.987 | 1.00 | 1.00 | 4824 | 3651 | 3649 |
| L83067 | 0.987 | 1.21 | 1.18 | 4825 | 6123 | 5841 |
| C85007 | 0.987 | 1.26 | 1.27 | 4826 | 6561 | 6589 |
| F82013 | 0.987 | 0.94 | 0.94 | 4827 | 2738 | 2772 |
| M85792 | 0.987 | 1.25 | 1.24 | 4828 | 6490 | 6384 |
| L84084 | 0.987 | 1.13 | 1.09 | 4829 | 5268 | 4779 |
| G83630 | 0.987 | 0.98 | 0.97 | 4830 | 3335 | 3246 |
| E86615 | 0.987 | 1.03 | 1.02 | 4831 | 4062 | 3956 |
| P87035 | 0.987 | 1.05 | 1.07 | 4832 | 4309 | 4638 |
| A83641 | 0.987 | 1.16 | 1.15 | 4833 | 5609 | 5523 |
| K84017 | 0.987 | 1.03 | 1.01 | 4834 | 4007 | 3700 |
| K83048 | 0.986 | 1.15 | 1.15 | 4835 | 5533 | 5576 |
| J82114 | 0.986 | 1.22 | 1.22 | 4836 | 6190 | 6214 |
| N85041 | 0.986 | 1.09 | 1.08 | 4837 | 4818 | 4689 |
| H81032 | 0.986 | 0.97 | 0.98 | 4838 | 3201 | 3303 |
| G82106 | 0.986 | 1.04 | 1.06 | 4839 | 4162 | 4522 |
| L84038 | 0.986 | 1.05 | 1.04 | 4840 | 4263 | 4140 |
| M81027 | 0.986 | 1.01 | 0.99 | 4841 | 3759 | 3499 |
| H81052 | 0.986 | 1.02 | 1.02 | 4842 | 3975 | 3957 |
| C81113 | 0.986 | 1.06 | 1.07 | 4843 | 4458 | 4554 |
| N82052 | 0.986 | 1.23 | 1.23 | 4844 | 6285 | 6369 |

| | | | | | | |
|---|---|---|---|---|---|---|
| E84685 | 0.985 | 0.98 | 0.97 | 4845 | 3282 | 3161 |
| H85006 | 0.985 | 1.03 | 1.00 | 4846 | 4068 | 3675 |
| Y00351 | 0.985 | 1.08 | 1.09 | 4847 | 4709 | 4898 |
| K84037 | 0.985 | 1.02 | 0.99 | 4848 | 3894 | 3536 |
| P84017 | 0.985 | 1.12 | 1.14 | 4849 | 5289 | 5512 |
| H84623 | 0.985 | 0.94 | 0.92 | 4850 | 2816 | 2611 |
| L85024 | 0.985 | 1.21 | 1.21 | 4851 | 6152 | 6138 |
| J81021 | 0.985 | 1.06 | 1.05 | 4852 | 4419 | 4411 |
| B82609 | 0.985 | 1.05 | 1.08 | 4853 | 4357 | 4737 |
| C81058 | 0.984 | 1.32 | 1.30 | 4854 | 6895 | 6788 |
| L81669 | 0.984 | 1.06 | 1.07 | 4855 | 4531 | 4671 |
| F81730 | 0.984 | 1.06 | 1.05 | 4856 | 4482 | 4410 |
| E85040 | 0.984 | 0.93 | 0.93 | 4857 | 2717 | 2640 |
| F84698 | 0.984 | 0.98 | 0.98 | 4858 | 3345 | 3355 |
| D81045 | 0.984 | 1.09 | 1.08 | 4859 | 4894 | 4819 |
| F86011 | 0.984 | 1.03 | 1.04 | 4860 | 4029 | 4277 |
| D81606 | 0.984 | 0.95 | 0.95 | 4861 | 2959 | 2982 |
| L82059 | 0.983 | 1.16 | 1.16 | 4862 | 5674 | 5718 |
| B82019 | 0.983 | 1.00 | 1.00 | 4863 | 3632 | 3668 |
| B82067 | 0.983 | 1.08 | 1.08 | 4864 | 4715 | 4723 |
| N83608 | 0.983 | 1.21 | 1.21 | 4865 | 6155 | 6220 |
| F86022 | 0.983 | 1.10 | 1.07 | 4866 | 4981 | 4677 |
| L81028 | 0.983 | 1.12 | 1.10 | 4867 | 5226 | 5032 |
| H81107 | 0.983 | 0.91 | 0.91 | 4868 | 2529 | 2440 |
| M83092 | 0.983 | 1.07 | 1.06 | 4869 | 4686 | 4568 |
| J81032 | 0.983 | 1.15 | 1.12 | 4870 | 5588 | 5260 |
| F84711 | 0.983 | 1.07 | 1.08 | 4871 | 4645 | 4830 |
| H82007 | 0.982 | 1.17 | 1.16 | 4872 | 5772 | 5683 |
| H81047 | 0.982 | 0.98 | 0.95 | 4873 | 3286 | 2994 |
| E84706 | 0.982 | 1.17 | 1.15 | 4874 | 5797 | 5564 |
| F81076 | 0.982 | 0.99 | 0.98 | 4875 | 3589 | 3453 |
| D82100 | 0.982 | 1.20 | 1.18 | 4876 | 6112 | 5946 |
| E84061 | 0.982 | 1.13 | 1.10 | 4877 | 5358 | 5048 |
| M83738 | 0.982 | 1.17 | 1.20 | 4878 | 5759 | 6103 |
| D83004 | 0.982 | 1.05 | 1.06 | 4879 | 4401 | 4571 |
| M82047 | 0.981 | 1.12 | 1.12 | 4880 | 5299 | 5332 |
| B86036 | 0.981 | 1.15 | 1.17 | 4881 | 5621 | 5802 |
| H81089 | 0.981 | 0.97 | 0.96 | 4882 | 3273 | 3123 |
| B86017 | 0.981 | 0.97 | 0.99 | 4883 | 3210 | 3488 |
| F81079 | 0.981 | 1.12 | 1.12 | 4884 | 5224 | 5229 |
| P88024 | 0.981 | 1.05 | 1.05 | 4885 | 4376 | 4446 |
| G83013 | 0.981 | 1.04 | 1.04 | 4886 | 4281 | 4230 |
| C88052 | 0.981 | 1.01 | 1.03 | 4887 | 3792 | 4106 |
| H83030 | 0.981 | 0.98 | 0.97 | 4888 | 3385 | 3295 |
| G84607 | 0.980 | 1.04 | 1.03 | 4889 | 4294 | 4210 |
| J82197 | 0.980 | 0.94 | 0.94 | 4890 | 2866 | 2877 |
| C81013 | 0.980 | 1.10 | 1.10 | 4891 | 5005 | 4981 |
| E81034 | 0.980 | 1.08 | 1.07 | 4892 | 4748 | 4686 |
| F82649 | 0.980 | 1.04 | 1.04 | 4893 | 4289 | 4256 |
| F85697 | 0.980 | 0.94 | 0.95 | 4894 | 2931 | 3045 |

| | | | | | | |
|---|---|---|---|---|---|---|
| H82078 | 0.980 | 1.09 | 1.10 | 4895 | 4931 | 5060 |
| L81120 | 0.980 | 1.08 | 1.08 | 4896 | 4788 | 4876 |
| G85029 | 0.980 | 0.93 | 0.94 | 4897 | 2803 | 2837 |
| H82030 | 0.979 | 1.04 | 1.02 | 4898 | 4275 | 3981 |
| L81123 | 0.979 | 1.05 | 1.03 | 4899 | 4367 | 4224 |
| M83617 | 0.979 | 1.15 | 1.15 | 4900 | 5628 | 5602 |
| K84072 | 0.979 | 1.09 | 1.07 | 4901 | 4998 | 4647 |
| Y01177 | 0.979 | 0.87 | 0.89 | 4902 | 2089 | 2263 |
| H81051 | 0.979 | 0.99 | 0.97 | 4903 | 3615 | 3283 |
| P88025 | 0.979 | 1.03 | 1.03 | 4904 | 4102 | 4177 |
| J83003 | 0.979 | 1.13 | 1.12 | 4905 | 5466 | 5296 |
| G82212 | 0.978 | 0.98 | 0.99 | 4906 | 3428 | 3616 |
| J81631 | 0.978 | 1.15 | 1.12 | 4907 | 5655 | 5308 |
| G81003 | 0.978 | 1.22 | 1.24 | 4908 | 6324 | 6453 |
| A81023 | 0.978 | 1.09 | 1.10 | 4909 | 4913 | 5070 |
| L83118 | 0.978 | 1.22 | 1.22 | 4910 | 6308 | 6332 |
| P92651 | 0.978 | 1.14 | 1.15 | 4911 | 5529 | 5638 |
| E82063 | 0.978 | 1.00 | 1.00 | 4912 | 3768 | 3813 |
| M89007 | 0.978 | 1.12 | 1.08 | 4913 | 5286 | 4864 |
| D82099 | 0.978 | 1.02 | 1.02 | 4914 | 3987 | 4060 |
| E82099 | 0.977 | 1.04 | 1.03 | 4915 | 4244 | 4173 |
| K82035 | 0.977 | 0.95 | 0.93 | 4916 | 3051 | 2785 |
| F81023 | 0.977 | 0.97 | 0.93 | 4917 | 3277 | 2751 |
| C81642 | 0.977 | 1.00 | 0.98 | 4918 | 3681 | 3511 |
| F84702 | 0.977 | 0.92 | 0.92 | 4919 | 2616 | 2639 |
| J83643 | 0.977 | 0.99 | 0.99 | 4920 | 3640 | 3562 |
| J82126 | 0.977 | 1.04 | 1.05 | 4921 | 4265 | 4464 |
| G82027 | 0.977 | 1.08 | 1.09 | 4922 | 4823 | 4987 |
| J84003 | 0.977 | 1.12 | 1.12 | 4923 | 5359 | 5294 |
| P81165 | 0.977 | 1.31 | 1.32 | 4924 | 6855 | 6922 |
| C81053 | 0.977 | 1.15 | 1.14 | 4925 | 5647 | 5536 |
| A89015 | 0.977 | 1.18 | 1.19 | 4926 | 5932 | 6078 |
| J82181 | 0.977 | 1.05 | 1.04 | 4927 | 4429 | 4349 |
| J82023 | 0.977 | 0.90 | 0.90 | 4928 | 2435 | 2386 |
| M81067 | 0.977 | 0.99 | 0.99 | 4929 | 3646 | 3557 |
| N81115 | 0.977 | 1.07 | 1.05 | 4930 | 4673 | 4394 |
| F86639 | 0.977 | 0.97 | 0.99 | 4931 | 3244 | 3552 |
| M85028 | 0.976 | 1.03 | 1.02 | 4932 | 4148 | 4058 |
| K84043 | 0.976 | 1.06 | 1.03 | 4933 | 4589 | 4236 |
| L84007 | 0.976 | 0.98 | 0.98 | 4934 | 3445 | 3493 |
| G82013 | 0.976 | 1.02 | 1.01 | 4935 | 4030 | 3851 |
| F81716 | 0.976 | 0.99 | 1.00 | 4936 | 3614 | 3742 |
| C82122 | 0.976 | 1.13 | 1.14 | 4937 | 5493 | 5538 |
| P88041 | 0.976 | 0.98 | 0.98 | 4938 | 3431 | 3555 |
| M86040 | 0.976 | 1.09 | 1.08 | 4939 | 4946 | 4815 |
| B86030 | 0.976 | 0.96 | 0.98 | 4940 | 3154 | 3502 |
| Y00280 | 0.976 | 1.00 | 1.01 | 4941 | 3762 | 3915 |
| K82047 | 0.976 | 1.04 | 1.02 | 4942 | 4338 | 4007 |
| P81136 | 0.976 | 1.26 | 1.26 | 4943 | 6638 | 6627 |
| C85023 | 0.976 | 1.32 | 1.32 | 4944 | 6907 | 6906 |

| | | | | | | |
|---|---|---|---|---|---|---|
| K81022 | 0.975 | 0.98 | 0.96 | 4945 | 3433 | 3152 |
| L84021 | 0.975 | 1.15 | 1.13 | 4946 | 5651 | 5493 |
| E82059 | 0.975 | 0.97 | 0.96 | 4947 | 3324 | 3244 |
| J81057 | 0.975 | 1.15 | 1.14 | 4948 | 5645 | 5584 |
| G82793 | 0.975 | 1.32 | 1.31 | 4949 | 6926 | 6897 |
| C84076 | 0.975 | 1.15 | 1.17 | 4950 | 5654 | 5830 |
| E85113 | 0.975 | 0.97 | 0.96 | 4951 | 3268 | 3200 |
| C87030 | 0.975 | 1.21 | 1.22 | 4952 | 6243 | 6314 |
| M82055 | 0.975 | 1.12 | 1.12 | 4953 | 5384 | 5347 |
| L81004 | 0.975 | 1.03 | 1.01 | 4954 | 4215 | 3968 |
| L82048 | 0.975 | 1.14 | 1.15 | 4955 | 5538 | 5618 |
| E82022 | 0.975 | 1.02 | 1.01 | 4956 | 4051 | 3923 |
| G83005 | 0.974 | 1.19 | 1.17 | 4957 | 6067 | 5899 |
| M85177 | 0.974 | 1.21 | 1.17 | 4958 | 6217 | 5839 |
| G82108 | 0.974 | 1.23 | 1.25 | 4959 | 6437 | 6556 |
| J82190 | 0.974 | 1.01 | 1.00 | 4960 | 3908 | 3794 |
| D82056 | 0.974 | 1.16 | 1.15 | 4961 | 5813 | 5681 |
| N82645 | 0.974 | 1.12 | 1.12 | 4962 | 5319 | 5392 |
| P89020 | 0.974 | 1.22 | 1.23 | 4963 | 6329 | 6479 |
| E84620 | 0.974 | 1.07 | 1.06 | 4964 | 4782 | 4632 |
| A87020 | 0.974 | 1.05 | 1.07 | 4965 | 4529 | 4818 |
| L85624 | 0.973 | 1.21 | 1.21 | 4966 | 6290 | 6287 |
| C81037 | 0.973 | 1.04 | 1.04 | 4967 | 4386 | 4365 |
| M88035 | 0.973 | 1.24 | 1.25 | 4968 | 6506 | 6562 |
| D83016 | 0.973 | 1.21 | 1.21 | 4969 | 6262 | 6254 |
| F81195 | 0.972 | 1.09 | 1.09 | 4970 | 5032 | 5004 |
| C82002 | 0.972 | 1.06 | 1.06 | 4971 | 4663 | 4612 |
| K82057 | 0.972 | 1.03 | 1.03 | 4972 | 4246 | 4241 |
| L82620 | 0.972 | 1.31 | 1.30 | 4973 | 6884 | 6840 |
| P81191 | 0.972 | 1.19 | 1.19 | 4974 | 6077 | 6069 |
| F85687 | 0.972 | 1.02 | 1.01 | 4975 | 4052 | 4022 |
| K84045 | 0.972 | 1.06 | 1.06 | 4976 | 4646 | 4599 |
| C84029 | 0.972 | 1.08 | 1.07 | 4977 | 4862 | 4775 |
| F85055 | 0.972 | 1.05 | 1.05 | 4978 | 4576 | 4488 |
| H81037 | 0.972 | 1.02 | 1.00 | 4979 | 4123 | 3899 |
| K82633 | 0.971 | 0.87 | 0.87 | 4980 | 2179 | 2196 |
| P81113 | 0.971 | 1.12 | 1.10 | 4981 | 5441 | 5084 |
| B83661 | 0.971 | 1.23 | 1.24 | 4982 | 6439 | 6528 |
| P84050 | 0.971 | 1.22 | 1.23 | 4983 | 6342 | 6467 |
| M86012 | 0.971 | 1.13 | 1.13 | 4984 | 5531 | 5463 |
| E85672 | 0.970 | 0.98 | 1.00 | 4985 | 3569 | 3873 |
| H81035 | 0.970 | 0.97 | 0.94 | 4986 | 3310 | 2959 |
| C81036 | 0.970 | 1.13 | 1.12 | 4987 | 5472 | 5431 |
| J83022 | 0.970 | 0.95 | 0.96 | 4988 | 3079 | 3287 |
| L83025 | 0.970 | 1.18 | 1.17 | 4989 | 5990 | 5943 |
| L84018 | 0.970 | 1.03 | 1.02 | 4990 | 4293 | 4128 |
| G85623 | 0.970 | 0.90 | 0.90 | 4991 | 2539 | 2573 |
| L83129 | 0.970 | 1.24 | 1.23 | 4992 | 6559 | 6464 |
| C86023 | 0.970 | 1.18 | 1.20 | 4993 | 5980 | 6192 |
| N81081 | 0.970 | 0.96 | 0.94 | 4994 | 3239 | 3028 |

| | | | | | | |
|---|---|---|---|---|---|---|
| E85111 | 0.969 | 0.95 | 0.95 | 4995 | 3093 | 3208 |
| K84041 | 0.969 | 1.00 | 0.97 | 4996 | 3838 | 3438 |
| P81210 | 0.969 | 1.21 | 1.18 | 4997 | 6332 | 5994 |
| L84613 | 0.969 | 1.21 | 1.20 | 4998 | 6335 | 6236 |
| N82066 | 0.969 | 1.14 | 1.16 | 4999 | 5601 | 5835 |
| C87012 | 0.969 | 1.19 | 1.20 | 5000 | 6120 | 6175 |
| J81036 | 0.969 | 1.18 | 1.18 | 5001 | 6032 | 5986 |
| J81621 | 0.969 | 1.17 | 1.15 | 5002 | 5897 | 5759 |
| E87017 | 0.969 | 0.96 | 0.95 | 5003 | 3283 | 3229 |
| M85143 | 0.969 | 1.17 | 1.16 | 5004 | 5950 | 5817 |
| H83040 | 0.969 | 0.92 | 0.92 | 5005 | 2817 | 2784 |
| L82009 | 0.969 | 1.12 | 1.12 | 5006 | 5381 | 5422 |
| E85721 | 0.969 | 1.04 | 1.05 | 5007 | 4473 | 4536 |
| E82044 | 0.969 | 0.98 | 0.97 | 5008 | 3508 | 3446 |
| H85033 | 0.968 | 1.04 | 1.02 | 5009 | 4360 | 4172 |
| L83058 | 0.968 | 1.12 | 1.09 | 5010 | 5467 | 5033 |
| L85052 | 0.968 | 1.15 | 1.12 | 5011 | 5750 | 5388 |
| B87001 | 0.968 | 1.12 | 1.14 | 5012 | 5396 | 5630 |
| E87026 | 0.968 | 1.42 | 1.36 | 5013 | 7171 | 7063 |
| E81050 | 0.968 | 1.02 | 1.01 | 5014 | 4153 | 4087 |
| F82003 | 0.968 | 1.12 | 1.12 | 5015 | 5400 | 5464 |
| G82690 | 0.968 | 1.08 | 1.08 | 5016 | 4916 | 4926 |
| D82048 | 0.968 | 1.06 | 1.08 | 5017 | 4728 | 4965 |
| B81016 | 0.968 | 1.04 | 1.07 | 5018 | 4455 | 4851 |
| E84057 | 0.968 | 1.03 | 1.01 | 5019 | 4241 | 3964 |
| M85175 | 0.968 | 0.97 | 0.97 | 5020 | 3364 | 3397 |
| C81065 | 0.967 | 1.18 | 1.16 | 5021 | 6012 | 5825 |
| J83603 | 0.967 | 0.99 | 0.98 | 5022 | 3733 | 3687 |
| M85124 | 0.967 | 1.00 | 0.99 | 5023 | 3861 | 3767 |
| C84059 | 0.967 | 1.21 | 1.19 | 5024 | 6263 | 6118 |
| B86642 | 0.967 | 1.18 | 1.19 | 5025 | 6037 | 6150 |
| D82621 | 0.967 | 1.36 | 1.34 | 5026 | 7066 | 6995 |
| A81015 | 0.967 | 1.20 | 1.21 | 5027 | 6222 | 6329 |
| G82760 | 0.967 | 1.14 | 1.17 | 5028 | 5686 | 5936 |
| F85016 | 0.967 | 0.93 | 0.94 | 5029 | 2968 | 2996 |
| H81079 | 0.967 | 1.06 | 1.03 | 5030 | 4684 | 4280 |
| B82080 | 0.967 | 1.08 | 1.07 | 5031 | 4958 | 4869 |
| D81016 | 0.967 | 1.05 | 1.01 | 5032 | 4515 | 4039 |
| K84004 | 0.967 | 1.04 | 1.03 | 5033 | 4470 | 4327 |
| M82038 | 0.966 | 1.08 | 1.08 | 5034 | 4947 | 4978 |
| D82067 | 0.966 | 1.26 | 1.27 | 5035 | 6653 | 6725 |
| A87612 | 0.966 | 1.19 | 1.21 | 5036 | 6153 | 6296 |
| M81040 | 0.966 | 1.18 | 1.16 | 5037 | 6019 | 5877 |
| E87052 | 0.966 | 1.05 | 1.04 | 5038 | 4553 | 4420 |
| G82730 | 0.966 | 1.00 | 0.99 | 5039 | 3863 | 3795 |
| M89015 | 0.966 | 1.03 | 1.01 | 5040 | 4334 | 3998 |
| M81002 | 0.966 | 1.06 | 1.09 | 5041 | 4771 | 5049 |
| C81061 | 0.965 | 1.14 | 1.12 | 5042 | 5659 | 5432 |
| M83006 | 0.965 | 0.99 | 1.00 | 5043 | 3810 | 3870 |
| L83639 | 0.965 | 1.03 | 1.04 | 5044 | 4317 | 4443 |

| | | | | | | |
|---|---|---|---|---|---|---|
| F81115 | 0.965 | 1.06 | 1.06 | 5045 | 4685 | 4760 |
| K84001 | 0.965 | 0.91 | 0.89 | 5046 | 2682 | 2471 |
| E86011 | 0.965 | 1.07 | 1.06 | 5047 | 4903 | 4783 |
| Y00268 | 0.965 | 0.90 | 0.89 | 5048 | 2633 | 2496 |
| H85003 | 0.965 | 0.94 | 0.95 | 5049 | 3091 | 3248 |
| C83020 | 0.965 | 1.14 | 1.13 | 5050 | 5661 | 5571 |
| C84086 | 0.965 | 1.00 | 0.99 | 5051 | 3948 | 3757 |
| D82001 | 0.965 | 1.12 | 1.10 | 5052 | 5435 | 5205 |
| J83625 | 0.965 | 0.97 | 0.96 | 5053 | 3484 | 3426 |
| M84014 | 0.964 | 0.97 | 0.97 | 5054 | 3412 | 3542 |
| G84027 | 0.964 | 0.93 | 0.93 | 5055 | 2958 | 2962 |
| L81101 | 0.964 | 1.11 | 1.10 | 5056 | 5402 | 5257 |
| K82007 | 0.964 | 0.97 | 0.95 | 5057 | 3526 | 3258 |
| F85030 | 0.964 | 1.05 | 1.05 | 5058 | 4608 | 4692 |
| E86006 | 0.964 | 1.01 | 1.00 | 5059 | 4036 | 3995 |
| M85062 | 0.964 | 1.12 | 1.11 | 5060 | 5489 | 5382 |
| N81112 | 0.964 | 1.02 | 1.00 | 5061 | 4155 | 3993 |
| C84020 | 0.964 | 1.12 | 1.16 | 5062 | 5510 | 5852 |
| K84035 | 0.964 | 1.00 | 0.98 | 5063 | 3943 | 3626 |
| A82046 | 0.964 | 1.06 | 1.03 | 5064 | 4789 | 4423 |
| M84023 | 0.964 | 0.98 | 0.97 | 5065 | 3676 | 3584 |
| M81080 | 0.964 | 1.07 | 1.05 | 5066 | 4867 | 4669 |
| H83009 | 0.963 | 1.00 | 0.99 | 5067 | 3912 | 3823 |
| P88012 | 0.963 | 1.16 | 1.15 | 5068 | 5868 | 5836 |
| M83665 | 0.963 | 1.09 | 1.10 | 5069 | 5149 | 5278 |
| J82025 | 0.963 | 1.01 | 0.99 | 5070 | 4022 | 3892 |
| E82054 | 0.963 | 0.94 | 0.92 | 5071 | 3086 | 2864 |
| G81086 | 0.963 | 1.00 | 0.99 | 5072 | 4000 | 3770 |
| M85037 | 0.963 | 1.10 | 1.09 | 5073 | 5210 | 5104 |
| C83067 | 0.963 | 1.18 | 1.16 | 5074 | 6129 | 5935 |
| L85025 | 0.963 | 1.06 | 1.07 | 5075 | 4822 | 4872 |
| H84033 | 0.963 | 0.97 | 0.96 | 5076 | 3497 | 3330 |
| M83067 | 0.963 | 1.08 | 1.09 | 5077 | 4986 | 5086 |
| G81642 | 0.962 | 1.07 | 1.09 | 5078 | 4851 | 5068 |
| A89024 | 0.962 | 1.32 | 1.33 | 5079 | 6968 | 6991 |
| H84618 | 0.962 | 1.05 | 1.03 | 5080 | 4640 | 4399 |
| C82678 | 0.962 | 1.19 | 1.19 | 5081 | 6149 | 6179 |
| G81032 | 0.962 | 1.03 | 1.05 | 5082 | 4351 | 4613 |
| D81038 | 0.962 | 1.05 | 1.05 | 5083 | 4704 | 4709 |
| K82617 | 0.962 | 1.03 | 1.03 | 5084 | 4420 | 4323 |
| L83055 | 0.962 | 1.25 | 1.25 | 5085 | 6635 | 6621 |
| L85047 | 0.962 | 1.01 | 0.99 | 5086 | 4109 | 3890 |
| B84007 | 0.962 | 0.99 | 1.01 | 5087 | 3801 | 4139 |
| J82091 | 0.962 | 1.05 | 1.07 | 5088 | 4697 | 4885 |
| P92028 | 0.961 | 1.26 | 1.25 | 5089 | 6713 | 6674 |
| M83078 | 0.961 | 1.04 | 1.04 | 5090 | 4509 | 4496 |
| F84624 | 0.961 | 1.03 | 1.04 | 5091 | 4375 | 4527 |
| C84710 | 0.961 | 1.23 | 1.26 | 5092 | 6505 | 6688 |
| H84007 | 0.961 | 0.91 | 0.89 | 5093 | 2681 | 2509 |
| D83011 | 0.961 | 1.19 | 1.19 | 5094 | 6172 | 6200 |

| | | | | | | |
|---|---|---|---|---|---|---|
| C88078 | 0.961 | 1.13 | 1.15 | 5095 | 5637 | 5775 |
| F86635 | 0.961 | 1.14 | 1.12 | 5096 | 5688 | 5475 |
| G81049 | 0.961 | 1.17 | 1.14 | 5097 | 5954 | 5701 |
| J82082 | 0.961 | 1.04 | 1.03 | 5098 | 4546 | 4347 |
| G84019 | 0.961 | 1.20 | 1.18 | 5099 | 6312 | 6146 |
| J83058 | 0.961 | 1.01 | 1.01 | 5100 | 4118 | 4089 |
| K84014 | 0.961 | 0.99 | 0.97 | 5101 | 3820 | 3505 |
| M85029 | 0.960 | 1.14 | 1.12 | 5102 | 5675 | 5554 |
| E82091 | 0.960 | 1.04 | 1.03 | 5103 | 4527 | 4397 |
| F82051 | 0.960 | 1.05 | 1.05 | 5104 | 4604 | 4741 |
| P81015 | 0.960 | 1.15 | 1.15 | 5105 | 5845 | 5791 |
| G85690 | 0.960 | 0.92 | 0.93 | 5106 | 2820 | 2957 |
| M82022 | 0.960 | 1.12 | 1.11 | 5107 | 5500 | 5455 |
| M85052 | 0.960 | 1.09 | 1.08 | 5108 | 5234 | 5025 |
| B81082 | 0.959 | 1.02 | 1.03 | 5109 | 4311 | 4398 |
| J82640 | 0.959 | 1.08 | 1.07 | 5110 | 5008 | 4915 |
| F85688 | 0.959 | 0.91 | 0.92 | 5111 | 2756 | 2861 |
| N83628 | 0.959 | 1.22 | 1.23 | 5112 | 6467 | 6557 |
| M81013 | 0.959 | 1.13 | 1.10 | 5113 | 5605 | 5338 |
| B83041 | 0.959 | 1.13 | 1.14 | 5114 | 5669 | 5717 |
| D82613 | 0.959 | 1.20 | 1.21 | 5115 | 6307 | 6424 |
| H83029 | 0.959 | 0.99 | 0.99 | 5116 | 3897 | 3867 |
| F84111 | 0.959 | 0.96 | 0.95 | 5117 | 3436 | 3321 |
| C83634 | 0.959 | 1.29 | 1.31 | 5118 | 6878 | 6949 |
| J83632 | 0.959 | 1.07 | 1.06 | 5119 | 4935 | 4831 |
| F84666 | 0.959 | 0.95 | 0.93 | 5120 | 3212 | 3077 |
| M85693 | 0.958 | 1.03 | 1.02 | 5121 | 4385 | 4346 |
| C88046 | 0.958 | 1.14 | 1.16 | 5122 | 5734 | 5929 |
| E84617 | 0.958 | 0.98 | 0.96 | 5123 | 3707 | 3375 |
| C81091 | 0.958 | 1.07 | 1.07 | 5124 | 4990 | 4961 |
| P83623 | 0.958 | 1.05 | 1.06 | 5125 | 4707 | 4825 |
| J82092 | 0.958 | 1.05 | 1.06 | 5126 | 4730 | 4890 |
| B83067 | 0.958 | 1.14 | 1.16 | 5127 | 5790 | 5912 |
| N83047 | 0.958 | 1.08 | 1.10 | 5128 | 5109 | 5291 |
| K81102 | 0.958 | 0.96 | 0.97 | 5129 | 3463 | 3538 |
| C82035 | 0.958 | 1.09 | 1.10 | 5130 | 5213 | 5282 |
| J82074 | 0.958 | 1.00 | 0.99 | 5131 | 4070 | 3928 |
| C81054 | 0.958 | 1.08 | 1.08 | 5132 | 5043 | 5056 |
| E83639 | 0.957 | 1.11 | 1.09 | 5133 | 5488 | 5208 |
| B81049 | 0.957 | 1.15 | 1.19 | 5134 | 5831 | 6246 |
| M86035 | 0.957 | 1.23 | 1.21 | 5135 | 6525 | 6420 |
| N81044 | 0.957 | 1.12 | 1.10 | 5136 | 5551 | 5365 |
| M88040 | 0.957 | 1.11 | 1.13 | 5137 | 5427 | 5594 |
| E82661 | 0.957 | 1.05 | 1.04 | 5138 | 4680 | 4535 |
| K83622 | 0.957 | 1.02 | 1.06 | 5139 | 4349 | 4814 |
| P84689 | 0.957 | 0.96 | 0.99 | 5140 | 3471 | 3844 |
| Y00137 | 0.957 | 1.13 | 1.13 | 5141 | 5611 | 5658 |
| J81082 | 0.957 | 1.09 | 1.07 | 5142 | 5257 | 5010 |
| P81010 | 0.956 | 1.06 | 1.04 | 5143 | 4835 | 4562 |
| G82123 | 0.956 | 1.01 | 1.04 | 5144 | 4175 | 4534 |

| | | | | | | |
|---|---|---|---|---|---|---|
| A89040 | 0.956 | 1.36 | 1.36 | 5145 | 7091 | 7088 |
| F86641 | 0.956 | 0.93 | 0.92 | 5146 | 3098 | 2938 |
| C84106 | 0.956 | 1.16 | 1.19 | 5147 | 5925 | 6216 |
| H81104 | 0.956 | 1.00 | 0.99 | 5148 | 4067 | 3910 |
| D81036 | 0.956 | 1.18 | 1.17 | 5149 | 6104 | 6019 |
| M85009 | 0.956 | 1.10 | 1.10 | 5150 | 5399 | 5279 |
| P92602 | 0.956 | 1.19 | 1.20 | 5151 | 6273 | 6325 |
| F82001 | 0.956 | 0.94 | 0.95 | 5152 | 3119 | 3279 |
| C88652 | 0.956 | 0.94 | 0.96 | 5153 | 3194 | 3544 |
| F84087 | 0.956 | 1.03 | 1.03 | 5154 | 4528 | 4508 |
| F84022 | 0.956 | 0.94 | 0.93 | 5155 | 3222 | 3052 |
| L81093 | 0.956 | 1.07 | 1.07 | 5156 | 4959 | 5014 |
| E83025 | 0.955 | 0.98 | 0.96 | 5157 | 3723 | 3445 |
| A82028 | 0.955 | 1.07 | 1.09 | 5158 | 5048 | 5218 |
| C84005 | 0.955 | 1.08 | 1.06 | 5159 | 5127 | 4878 |
| B81653 | 0.955 | 1.05 | 1.05 | 5160 | 4787 | 4758 |
| E84021 | 0.955 | 0.98 | 0.97 | 5161 | 3774 | 3594 |
| G82017 | 0.955 | 1.04 | 1.04 | 5162 | 4559 | 4622 |
| L81064 | 0.955 | 1.22 | 1.20 | 5163 | 6512 | 6345 |
| G85651 | 0.955 | 0.91 | 0.91 | 5164 | 2768 | 2832 |
| M91016 | 0.954 | 1.12 | 1.14 | 5165 | 5617 | 5798 |
| F84714 | 0.954 | 1.02 | 1.02 | 5166 | 4313 | 4354 |
| P84678 | 0.954 | 1.14 | 1.17 | 5167 | 5783 | 6038 |
| Y02663 | 0.954 | 1.06 | 1.08 | 5168 | 4856 | 5124 |
| H83005 | 0.954 | 0.91 | 0.90 | 5169 | 2777 | 2718 |
| P92619 | 0.954 | 1.11 | 1.12 | 5170 | 5451 | 5572 |
| K83065 | 0.954 | 1.09 | 1.09 | 5171 | 5272 | 5226 |
| E82066 | 0.954 | 0.92 | 0.92 | 5172 | 2948 | 2894 |
| H81019 | 0.954 | 1.04 | 1.04 | 5173 | 4616 | 4642 |
| L84037 | 0.954 | 1.03 | 1.03 | 5174 | 4514 | 4434 |
| G81082 | 0.954 | 1.00 | 0.97 | 5175 | 4136 | 3636 |
| D82106 | 0.954 | 1.00 | 1.01 | 5176 | 4040 | 4287 |
| C83001 | 0.954 | 1.05 | 1.09 | 5177 | 4791 | 5263 |
| D83028 | 0.954 | 1.10 | 1.11 | 5178 | 5389 | 5435 |
| L81054 | 0.954 | 1.20 | 1.19 | 5179 | 6340 | 6298 |
| L85042 | 0.953 | 1.16 | 1.16 | 5180 | 5987 | 5988 |
| K84006 | 0.953 | 1.03 | 1.00 | 5181 | 4446 | 4170 |
| E87733 | 0.953 | 1.17 | 1.15 | 5182 | 6044 | 5905 |
| B85032 | 0.953 | 1.09 | 1.10 | 5183 | 5218 | 5315 |
| H82084 | 0.953 | 1.08 | 1.08 | 5184 | 5134 | 5057 |
| C84129 | 0.953 | 1.15 | 1.17 | 5185 | 5878 | 6106 |
| H83019 | 0.953 | 0.95 | 0.94 | 5186 | 3308 | 3317 |
| G85012 | 0.953 | 1.06 | 1.06 | 5187 | 4969 | 4844 |
| C82030 | 0.953 | 1.14 | 1.15 | 5188 | 5785 | 5895 |
| L82025 | 0.953 | 1.14 | 1.15 | 5189 | 5830 | 5870 |
| E83024 | 0.953 | 1.00 | 0.98 | 5190 | 4077 | 3775 |
| F85033 | 0.953 | 0.98 | 0.98 | 5191 | 3845 | 3845 |
| J82027 | 0.953 | 1.04 | 1.04 | 5192 | 4672 | 4630 |
| G83060 | 0.953 | 1.02 | 1.01 | 5193 | 4387 | 4301 |
| B86047 | 0.953 | 1.02 | 1.04 | 5194 | 4379 | 4663 |

| | | | | | | |
|---|---|---|---|---|---|---|
| E85743 | 0.952 | 1.05 | 1.05 | 5195 | 4731 | 4806 |
| C83637 | 0.952 | 1.26 | 1.25 | 5196 | 6763 | 6712 |
| A89023 | 0.952 | 1.21 | 1.21 | 5197 | 6407 | 6484 |
| E85054 | 0.952 | 1.07 | 1.08 | 5198 | 5086 | 5081 |
| H85041 | 0.952 | 0.94 | 0.92 | 5199 | 3219 | 3005 |
| K82060 | 0.952 | 0.96 | 0.96 | 5200 | 3547 | 3534 |
| C81044 | 0.952 | 1.21 | 1.19 | 5201 | 6415 | 6245 |
| M87036 | 0.952 | 1.08 | 1.07 | 5202 | 5143 | 5042 |
| H84010 | 0.952 | 0.94 | 0.93 | 5203 | 3226 | 3090 |
| K84618 | 0.952 | 0.96 | 0.94 | 5204 | 3489 | 3240 |
| J82061 | 0.952 | 1.01 | 0.97 | 5205 | 4212 | 3758 |
| B81677 | 0.952 | 1.15 | 1.17 | 5206 | 5892 | 6090 |
| A86015 | 0.952 | 0.90 | 0.92 | 5207 | 2701 | 2972 |
| K81070 | 0.952 | 0.95 | 0.93 | 5208 | 3390 | 3150 |
| M86027 | 0.952 | 1.04 | 1.03 | 5209 | 4643 | 4586 |
| A82029 | 0.951 | 1.20 | 1.21 | 5210 | 6385 | 6462 |
| F81698 | 0.951 | 1.05 | 1.05 | 5211 | 4756 | 4786 |
| H82065 | 0.951 | 1.24 | 1.25 | 5212 | 6656 | 6713 |
| F85682 | 0.951 | 0.88 | 0.88 | 5213 | 2518 | 2506 |
| B83022 | 0.951 | 1.18 | 1.20 | 5214 | 6185 | 6342 |
| G84030 | 0.951 | 1.02 | 1.02 | 5215 | 4364 | 4335 |
| L83106 | 0.951 | 1.15 | 1.15 | 5216 | 5933 | 5934 |
| L83647 | 0.951 | 1.19 | 1.17 | 5217 | 6320 | 6102 |
| N85032 | 0.951 | 1.13 | 1.13 | 5218 | 5748 | 5736 |
| F81066 | 0.951 | 1.12 | 1.10 | 5219 | 5641 | 5354 |
| H84635 | 0.951 | 0.94 | 0.93 | 5220 | 3256 | 3114 |
| N81080 | 0.951 | 1.09 | 1.07 | 5221 | 5339 | 5058 |
| P83017 | 0.951 | 1.09 | 1.09 | 5222 | 5290 | 5301 |
| Y03602 | 0.951 | 1.05 | 1.03 | 5223 | 4775 | 4590 |
| J81074 | 0.951 | 1.05 | 1.03 | 5224 | 4751 | 4544 |
| E85127 | 0.950 | 0.97 | 0.98 | 5225 | 3727 | 3812 |
| L83051 | 0.950 | 1.10 | 1.10 | 5226 | 5454 | 5352 |
| P84027 | 0.950 | 1.05 | 1.07 | 5227 | 4814 | 5059 |
| G83044 | 0.950 | 0.99 | 0.99 | 5228 | 4009 | 3955 |
| E82028 | 0.950 | 0.96 | 0.95 | 5229 | 3485 | 3424 |
| M83705 | 0.950 | 1.03 | 1.03 | 5230 | 4540 | 4497 |
| K83053 | 0.950 | 1.03 | 1.04 | 5231 | 4542 | 4672 |
| M81032 | 0.950 | 1.08 | 1.06 | 5232 | 5161 | 4941 |
| F82612 | 0.950 | 1.10 | 1.11 | 5233 | 5449 | 5516 |
| P81736 | 0.950 | 1.22 | 1.26 | 5234 | 6564 | 6771 |
| L84032 | 0.950 | 1.16 | 1.15 | 5235 | 6011 | 5922 |
| L83048 | 0.950 | 1.18 | 1.20 | 5236 | 6196 | 6359 |
| A82036 | 0.950 | 1.19 | 1.17 | 5237 | 6311 | 6072 |
| M91021 | 0.950 | 1.35 | 1.36 | 5238 | 7078 | 7100 |
| E85605 | 0.950 | 1.01 | 1.00 | 5239 | 4207 | 4092 |
| C82037 | 0.949 | 1.34 | 1.34 | 5240 | 7064 | 7062 |
| A83040 | 0.949 | 1.20 | 1.20 | 5241 | 6387 | 6397 |
| D82037 | 0.949 | 1.04 | 1.03 | 5242 | 4700 | 4608 |
| M85103 | 0.949 | 1.06 | 1.06 | 5243 | 4972 | 4904 |
| H84629 | 0.949 | 0.94 | 0.93 | 5244 | 3297 | 3142 |

| | | | | | | |
|---|---|---|---|---|---|---|
| C88044 | 0.949 | 1.06 | 1.08 | 5245 | 4887 | 5134 |
| M81023 | 0.949 | 1.08 | 1.07 | 5246 | 5230 | 5069 |
| L84041 | 0.949 | 1.01 | 1.02 | 5247 | 4299 | 4474 |
| C88076 | 0.949 | 1.06 | 1.08 | 5248 | 5000 | 5122 |
| D82016 | 0.949 | 1.10 | 1.09 | 5249 | 5469 | 5333 |
| C81032 | 0.949 | 1.19 | 1.15 | 5250 | 6301 | 5968 |
| L83079 | 0.948 | 1.09 | 1.06 | 5251 | 5300 | 4956 |
| L85041 | 0.948 | 1.26 | 1.24 | 5252 | 6744 | 6652 |
| C81089 | 0.948 | 1.33 | 1.32 | 5253 | 7047 | 7011 |
| N81014 | 0.948 | 0.96 | 0.96 | 5254 | 3625 | 3662 |
| G83019 | 0.948 | 1.11 | 1.11 | 5255 | 5582 | 5485 |
| C83010 | 0.948 | 0.98 | 0.98 | 5256 | 3945 | 3912 |
| A83619 | 0.947 | 1.51 | 1.48 | 5257 | 7258 | 7243 |
| E86024 | 0.947 | 1.13 | 1.12 | 5258 | 5721 | 5678 |
| L84063 | 0.947 | 0.98 | 0.97 | 5259 | 3824 | 3712 |
| C88051 | 0.947 | 1.12 | 1.14 | 5260 | 5671 | 5897 |
| E81005 | 0.947 | 1.15 | 1.16 | 5261 | 5924 | 6032 |
| L83061 | 0.947 | 1.11 | 1.14 | 5262 | 5550 | 5810 |
| K82010 | 0.947 | 0.96 | 0.94 | 5263 | 3533 | 3342 |
| H83001 | 0.947 | 0.97 | 0.96 | 5264 | 3752 | 3648 |
| P81686 | 0.947 | 1.07 | 1.08 | 5265 | 5150 | 5144 |
| J82106 | 0.947 | 1.00 | 0.98 | 5266 | 4156 | 3840 |
| C84072 | 0.947 | 1.13 | 1.14 | 5267 | 5726 | 5875 |
| G85696 | 0.947 | 0.91 | 0.92 | 5268 | 2903 | 3070 |
| C87009 | 0.947 | 1.08 | 1.09 | 5269 | 5205 | 5371 |
| K84610 | 0.947 | 0.98 | 0.96 | 5270 | 3932 | 3640 |
| D83044 | 0.946 | 1.24 | 1.24 | 5271 | 6650 | 6665 |
| M81016 | 0.946 | 1.15 | 1.13 | 5272 | 5964 | 5785 |
| F84720 | 0.946 | 1.07 | 1.08 | 5273 | 5056 | 5189 |
| Y01127 | 0.946 | 1.11 | 1.12 | 5274 | 5574 | 5611 |
| C82662 | 0.946 | 0.90 | 0.91 | 5275 | 2783 | 2887 |
| B86012 | 0.946 | 1.15 | 1.16 | 5276 | 5927 | 6026 |
| F84088 | 0.946 | 1.12 | 1.10 | 5277 | 5623 | 5468 |
| N82033 | 0.946 | 1.26 | 1.26 | 5278 | 6808 | 6783 |
| B83040 | 0.946 | 1.05 | 1.06 | 5279 | 4815 | 5012 |
| M89601 | 0.946 | 0.97 | 0.94 | 5280 | 3750 | 3364 |
| A83072 | 0.946 | 1.10 | 1.11 | 5281 | 5412 | 5574 |
| H84055 | 0.946 | 0.85 | 0.84 | 5282 | 2219 | 2066 |
| K81043 | 0.946 | 1.04 | 1.05 | 5283 | 4760 | 4889 |
| P84062 | 0.946 | 1.13 | 1.14 | 5284 | 5720 | 5884 |
| M88620 | 0.945 | 1.10 | 1.12 | 5285 | 5491 | 5634 |
| E84029 | 0.945 | 1.07 | 1.05 | 5286 | 5076 | 4884 |
| H82049 | 0.945 | 1.08 | 1.10 | 5287 | 5281 | 5398 |
| M86624 | 0.945 | 1.12 | 1.11 | 5288 | 5663 | 5568 |
| D82040 | 0.945 | 1.10 | 1.11 | 5289 | 5434 | 5521 |
| E84645 | 0.945 | 0.96 | 0.94 | 5290 | 3562 | 3374 |
| F82638 | 0.945 | 0.93 | 0.94 | 5291 | 3246 | 3305 |
| P91009 | 0.945 | 1.18 | 1.18 | 5292 | 6192 | 6221 |
| G82143 | 0.945 | 1.07 | 1.08 | 5293 | 5184 | 5273 |
| H85048 | 0.945 | 0.89 | 0.87 | 5294 | 2642 | 2452 |

| | | | | | | |
|---|---|---|---|---|---|---|
| L81065 | 0.945 | 1.03 | 1.02 | 5295 | 4571 | 4442 |
| K81004 | 0.945 | 1.07 | 1.07 | 5296 | 5073 | 5050 |
| B86038 | 0.945 | 1.07 | 1.09 | 5297 | 5164 | 5386 |
| L82036 | 0.944 | 1.14 | 1.14 | 5298 | 5855 | 5902 |
| K81018 | 0.944 | 0.96 | 0.95 | 5299 | 3683 | 3525 |
| C81038 | 0.944 | 1.07 | 1.07 | 5300 | 5168 | 5169 |
| B81080 | 0.944 | 1.23 | 1.28 | 5301 | 6612 | 6863 |
| Y00352 | 0.944 | 0.92 | 0.92 | 5302 | 3102 | 3075 |
| J83007 | 0.944 | 1.03 | 1.02 | 5303 | 4602 | 4493 |
| J81011 | 0.944 | 1.18 | 1.14 | 5304 | 6203 | 5850 |
| H82023 | 0.943 | 1.31 | 1.32 | 5305 | 7005 | 7016 |
| C84696 | 0.943 | 1.03 | 1.02 | 5306 | 4676 | 4472 |
| G85134 | 0.943 | 0.92 | 0.92 | 5307 | 3018 | 3089 |
| G81613 | 0.943 | 1.00 | 1.02 | 5308 | 4267 | 4502 |
| E82062 | 0.943 | 1.04 | 1.04 | 5309 | 4765 | 4824 |
| N83026 | 0.943 | 1.19 | 1.20 | 5310 | 6360 | 6398 |
| J81039 | 0.943 | 1.15 | 1.15 | 5311 | 6015 | 6036 |
| F84668 | 0.943 | 0.90 | 0.91 | 5312 | 2809 | 3029 |
| J83031 | 0.943 | 1.17 | 1.17 | 5313 | 6131 | 6203 |
| M83024 | 0.943 | 1.09 | 1.08 | 5314 | 5406 | 5289 |
| M85035 | 0.942 | 1.23 | 1.20 | 5315 | 6615 | 6461 |
| P89026 | 0.942 | 1.31 | 1.32 | 5316 | 6999 | 7030 |
| H81004 | 0.942 | 1.01 | 0.99 | 5317 | 4298 | 4200 |
| E83006 | 0.942 | 0.94 | 0.92 | 5318 | 3346 | 3130 |
| L83143 | 0.942 | 1.02 | 0.99 | 5319 | 4453 | 4059 |
| F81633 | 0.942 | 1.10 | 1.12 | 5320 | 5509 | 5673 |
| K81045 | 0.942 | 0.99 | 0.99 | 5321 | 4082 | 4204 |
| J81647 | 0.942 | 1.06 | 1.05 | 5322 | 5038 | 4855 |
| C81010 | 0.942 | 1.10 | 1.08 | 5323 | 5516 | 5300 |
| H82046 | 0.942 | 1.10 | 1.10 | 5324 | 5474 | 5473 |
| L81106 | 0.942 | 1.07 | 1.06 | 5325 | 5135 | 5037 |
| D82071 | 0.941 | 1.09 | 1.08 | 5326 | 5382 | 5253 |
| J82087 | 0.941 | 1.18 | 1.18 | 5327 | 6239 | 6299 |
| K84042 | 0.941 | 1.03 | 1.00 | 5328 | 4593 | 4321 |
| E81011 | 0.941 | 1.04 | 1.04 | 5329 | 4841 | 4761 |
| P81179 | 0.941 | 1.12 | 1.12 | 5330 | 5731 | 5664 |
| K83068 | 0.941 | 1.05 | 1.02 | 5331 | 4910 | 4514 |
| J81014 | 0.941 | 1.07 | 1.08 | 5332 | 5222 | 5251 |
| G82100 | 0.940 | 1.07 | 1.07 | 5333 | 5145 | 5120 |
| L85055 | 0.940 | 1.03 | 1.04 | 5334 | 4721 | 4799 |
| N83601 | 0.940 | 1.42 | 1.41 | 5335 | 7193 | 7183 |
| H82643 | 0.940 | 1.12 | 1.12 | 5336 | 5733 | 5668 |
| M85736 | 0.940 | 1.19 | 1.17 | 5337 | 6345 | 6193 |
| P81741 | 0.940 | 1.19 | 1.17 | 5338 | 6352 | 6180 |
| B86100 | 0.940 | 1.08 | 1.10 | 5339 | 5242 | 5461 |
| M86034 | 0.940 | 1.05 | 1.04 | 5340 | 4950 | 4859 |
| E85700 | 0.940 | 1.11 | 1.09 | 5341 | 5634 | 5438 |
| H85011 | 0.940 | 0.95 | 0.94 | 5342 | 3623 | 3387 |
| L81131 | 0.940 | 0.96 | 0.97 | 5343 | 3641 | 3838 |
| F86023 | 0.940 | 0.94 | 0.93 | 5344 | 3449 | 3274 |

| | | | | | | |
|---|---|---|---|---|---|---|
| D83041 | 0.940 | 1.05 | 1.07 | 5345 | 4987 | 5106 |
| E83012 | 0.939 | 1.00 | 0.98 | 5346 | 4277 | 3947 |
| L81635 | 0.939 | 1.21 | 1.17 | 5347 | 6531 | 6225 |
| L81073 | 0.939 | 1.03 | 1.03 | 5348 | 4732 | 4652 |
| J82195 | 0.939 | 0.98 | 0.98 | 5349 | 4023 | 4033 |
| G82051 | 0.939 | 1.08 | 1.11 | 5350 | 5287 | 5567 |
| G83018 | 0.939 | 1.05 | 1.03 | 5351 | 4885 | 4744 |
| K81094 | 0.939 | 0.93 | 0.93 | 5352 | 3217 | 3236 |
| E85714 | 0.939 | 0.98 | 0.99 | 5353 | 4071 | 4150 |
| C82659 | 0.939 | 1.00 | 1.00 | 5354 | 4251 | 4348 |
| D83070 | 0.939 | 1.18 | 1.17 | 5355 | 6274 | 6190 |
| H82094 | 0.939 | 1.30 | 1.31 | 5356 | 6980 | 6990 |
| P81140 | 0.939 | 1.58 | 1.60 | 5357 | 7288 | 7293 |
| K82070 | 0.939 | 1.01 | 0.98 | 5358 | 4359 | 4069 |
| E85041 | 0.939 | 0.93 | 0.94 | 5359 | 3306 | 3396 |
| P91018 | 0.939 | 1.18 | 1.18 | 5360 | 6296 | 6292 |
| E84680 | 0.938 | 1.03 | 1.01 | 5361 | 4719 | 4404 |
| C88016 | 0.938 | 1.02 | 1.04 | 5362 | 4517 | 4812 |
| L83108 | 0.938 | 1.08 | 1.09 | 5363 | 5376 | 5417 |
| L85027 | 0.938 | 1.08 | 1.09 | 5364 | 5372 | 5413 |
| M92654 | 0.938 | 1.16 | 1.17 | 5365 | 6125 | 6237 |
| P92646 | 0.938 | 1.07 | 1.07 | 5366 | 5179 | 5243 |
| E86003 | 0.938 | 1.05 | 1.04 | 5367 | 5013 | 4879 |
| F81062 | 0.938 | 1.04 | 1.02 | 5368 | 4837 | 4617 |
| K82001 | 0.938 | 0.99 | 0.97 | 5369 | 4224 | 3950 |
| F86679 | 0.938 | 0.97 | 0.99 | 5370 | 3893 | 4180 |
| P92639 | 0.937 | 1.18 | 1.19 | 5371 | 6303 | 6415 |
| G81046 | 0.937 | 1.07 | 1.09 | 5372 | 5215 | 5423 |
| N84025 | 0.937 | 1.23 | 1.23 | 5373 | 6693 | 6655 |
| M83080 | 0.937 | 1.08 | 1.04 | 5374 | 5295 | 4826 |
| L81041 | 0.937 | 1.13 | 1.13 | 5375 | 5858 | 5837 |
| L81052 | 0.937 | 1.05 | 1.04 | 5376 | 4949 | 4850 |
| D83051 | 0.937 | 1.04 | 1.06 | 5377 | 4901 | 5007 |
| C83018 | 0.937 | 1.23 | 1.22 | 5378 | 6649 | 6588 |
| P84061 | 0.937 | 1.13 | 1.15 | 5379 | 5819 | 6050 |
| N84026 | 0.937 | 1.01 | 1.01 | 5380 | 4383 | 4412 |
| G85088 | 0.937 | 0.98 | 0.99 | 5381 | 3998 | 4124 |
| D82009 | 0.937 | 1.30 | 1.27 | 5382 | 6981 | 6858 |
| G83001 | 0.937 | 1.02 | 1.02 | 5383 | 4609 | 4559 |
| H82004 | 0.936 | 0.96 | 0.94 | 5384 | 3751 | 3464 |
| K84059 | 0.936 | 1.04 | 1.03 | 5385 | 4847 | 4769 |
| E84023 | 0.936 | 0.97 | 0.96 | 5386 | 3882 | 3707 |
| H81002 | 0.936 | 0.91 | 0.91 | 5387 | 3084 | 3024 |
| M83115 | 0.936 | 1.22 | 1.22 | 5388 | 6632 | 6603 |
| Y01756 | 0.936 | 1.13 | 1.12 | 5389 | 5880 | 5794 |
| H85082 | 0.936 | 0.98 | 0.96 | 5390 | 4061 | 3754 |
| H82013 | 0.936 | 1.00 | 1.02 | 5391 | 4346 | 4540 |
| H81053 | 0.936 | 1.00 | 0.96 | 5392 | 4266 | 3786 |
| H82017 | 0.936 | 1.05 | 1.03 | 5393 | 4999 | 4693 |
| G82135 | 0.935 | 0.93 | 0.93 | 5394 | 3340 | 3312 |

| | | | | | | |
|---|---|---|---|---|---|---|
| M92649 | 0.935 | 1.25 | 1.25 | 5395 | 6784 | 6772 |
| C84602 | 0.935 | 0.99 | 1.02 | 5396 | 4201 | 4539 |
| H82038 | 0.935 | 1.19 | 1.18 | 5397 | 6398 | 6322 |
| F85036 | 0.935 | 0.97 | 0.97 | 5398 | 3885 | 3866 |
| L85002 | 0.935 | 1.13 | 1.11 | 5399 | 5864 | 5689 |
| M83123 | 0.935 | 1.19 | 1.23 | 5400 | 6443 | 6680 |
| B86633 | 0.935 | 1.58 | 1.56 | 5401 | 7290 | 7283 |
| N81002 | 0.935 | 1.01 | 1.00 | 5402 | 4511 | 4375 |
| C84136 | 0.935 | 1.08 | 1.10 | 5403 | 5317 | 5520 |
| F86621 | 0.934 | 0.94 | 0.96 | 5404 | 3538 | 3828 |
| G85046 | 0.934 | 0.97 | 0.99 | 5405 | 3995 | 4225 |
| H84032 | 0.934 | 0.89 | 0.87 | 5406 | 2792 | 2620 |
| L82038 | 0.934 | 1.21 | 1.21 | 5407 | 6592 | 6576 |
| F84122 | 0.934 | 1.05 | 1.05 | 5408 | 5061 | 5003 |
| C82628 | 0.934 | 1.04 | 1.05 | 5409 | 4891 | 4958 |
| C81069 | 0.934 | 1.09 | 1.09 | 5410 | 5514 | 5508 |
| L83059 | 0.934 | 1.11 | 1.11 | 5411 | 5636 | 5686 |
| M85142 | 0.934 | 1.32 | 1.30 | 5412 | 7058 | 6972 |
| A88014 | 0.934 | 1.29 | 1.27 | 5413 | 6974 | 6887 |
| F81718 | 0.934 | 1.00 | 1.01 | 5414 | 4302 | 4500 |
| L83607 | 0.934 | 1.25 | 1.24 | 5415 | 6804 | 6762 |
| E82104 | 0.933 | 1.04 | 1.03 | 5416 | 4864 | 4802 |
| F82639 | 0.933 | 0.94 | 0.95 | 5417 | 3504 | 3607 |
| E81019 | 0.933 | 1.12 | 1.11 | 5418 | 5786 | 5646 |
| N81632 | 0.932 | 1.02 | 1.01 | 5419 | 4596 | 4444 |
| F86607 | 0.932 | 0.92 | 0.94 | 5420 | 3216 | 3522 |
| Y00185 | 0.932 | 0.93 | 0.93 | 5421 | 3347 | 3331 |
| N85040 | 0.932 | 1.23 | 1.21 | 5422 | 6678 | 6571 |
| C84004 | 0.932 | 1.17 | 1.18 | 5423 | 6219 | 6348 |
| E82076 | 0.932 | 0.92 | 0.91 | 5424 | 3179 | 3177 |
| B82002 | 0.932 | 1.02 | 1.02 | 5425 | 4615 | 4696 |
| M84621 | 0.932 | 1.10 | 1.10 | 5426 | 5580 | 5577 |
| H81071 | 0.932 | 1.02 | 0.99 | 5427 | 4617 | 4268 |
| E81032 | 0.931 | 0.95 | 0.96 | 5428 | 3736 | 3897 |
| D83024 | 0.931 | 1.02 | 1.03 | 5429 | 4597 | 4765 |
| C81060 | 0.931 | 1.14 | 1.11 | 5430 | 6001 | 5710 |
| H81109 | 0.931 | 0.92 | 0.92 | 5431 | 3293 | 3212 |
| M85048 | 0.931 | 1.20 | 1.19 | 5432 | 6545 | 6428 |
| F81619 | 0.931 | 1.04 | 1.06 | 5433 | 4895 | 5158 |
| M83134 | 0.931 | 1.04 | 1.07 | 5434 | 4899 | 5319 |
| E82613 | 0.931 | 1.24 | 1.22 | 5435 | 6772 | 6664 |
| P81037 | 0.930 | 1.28 | 1.25 | 5436 | 6946 | 6794 |
| J82110 | 0.930 | 0.95 | 0.92 | 5437 | 3771 | 3337 |
| C87022 | 0.930 | 1.25 | 1.25 | 5438 | 6799 | 6814 |
| H81029 | 0.930 | 0.97 | 0.94 | 5439 | 3986 | 3560 |
| M86005 | 0.930 | 1.16 | 1.15 | 5440 | 6200 | 6112 |
| L82016 | 0.930 | 1.23 | 1.22 | 5441 | 6698 | 6682 |
| F85703 | 0.930 | 0.93 | 0.93 | 5442 | 3402 | 3425 |
| H81064 | 0.929 | 1.04 | 1.00 | 5443 | 4921 | 4441 |
| G83633 | 0.929 | 1.06 | 1.05 | 5444 | 5166 | 5065 |

| | | | | | | |
|---|---|---|---|---|---|---|
| P81056 | 0.929 | 1.19 | 1.20 | 5445 | 6489 | 6545 |
| J82164 | 0.929 | 1.03 | 1.02 | 5446 | 4761 | 4716 |
| N82116 | 0.929 | 1.04 | 1.06 | 5447 | 4960 | 5163 |
| M81629 | 0.929 | 1.05 | 1.03 | 5448 | 5077 | 4780 |
| Y02260 | 0.929 | 0.93 | 0.92 | 5449 | 3366 | 3357 |
| F85615 | 0.928 | 1.06 | 1.06 | 5450 | 5189 | 5222 |
| M85697 | 0.928 | 1.31 | 1.28 | 5451 | 7033 | 6952 |
| H81642 | 0.928 | 1.03 | 1.01 | 5452 | 4776 | 4564 |
| F86666 | 0.928 | 0.93 | 0.95 | 5453 | 3478 | 3740 |
| K81032 | 0.928 | 0.99 | 0.99 | 5454 | 4270 | 4270 |
| Y02757 | 0.928 | 0.94 | 0.96 | 5455 | 3664 | 3934 |
| L84056 | 0.928 | 1.05 | 1.06 | 5456 | 5136 | 5233 |
| D82064 | 0.927 | 1.05 | 1.04 | 5457 | 5070 | 4973 |
| J82129 | 0.927 | 1.02 | 1.02 | 5458 | 4769 | 4679 |
| H84034 | 0.927 | 1.03 | 1.01 | 5459 | 4907 | 4616 |
| D81041 | 0.927 | 0.97 | 0.97 | 5460 | 4079 | 3971 |
| N85001 | 0.927 | 1.07 | 1.06 | 5461 | 5304 | 5209 |
| B81052 | 0.927 | 0.98 | 1.02 | 5462 | 4137 | 4729 |
| H85026 | 0.927 | 0.87 | 0.86 | 5463 | 2651 | 2526 |
| D82618 | 0.927 | 1.07 | 1.07 | 5464 | 5344 | 5364 |
| D83053 | 0.926 | 1.15 | 1.15 | 5465 | 6145 | 6187 |
| A82072 | 0.926 | 1.75 | 1.77 | 5466 | 7310 | 7310 |
| J81034 | 0.926 | 1.07 | 1.06 | 5467 | 5361 | 5266 |
| F83673 | 0.926 | 1.00 | 1.01 | 5468 | 4466 | 4557 |
| E82015 | 0.926 | 0.96 | 0.94 | 5469 | 3883 | 3676 |
| H81658 | 0.926 | 1.00 | 0.97 | 5470 | 4457 | 4097 |
| B85622 | 0.926 | 1.17 | 1.18 | 5471 | 6343 | 6374 |
| J83021 | 0.926 | 1.05 | 1.04 | 5472 | 5060 | 4939 |
| D82087 | 0.926 | 1.05 | 1.06 | 5473 | 5137 | 5264 |
| F81209 | 0.926 | 1.12 | 1.12 | 5474 | 5833 | 5903 |
| C81092 | 0.926 | 1.01 | 1.01 | 5475 | 4629 | 4626 |
| Y00475 | 0.926 | 1.25 | 1.26 | 5476 | 6846 | 6864 |
| C81067 | 0.925 | 1.10 | 1.09 | 5477 | 5631 | 5592 |
| H85695 | 0.925 | 1.00 | 0.98 | 5478 | 4476 | 4237 |
| B86052 | 0.925 | 1.03 | 1.05 | 5479 | 4816 | 5052 |
| L83137 | 0.925 | 1.17 | 1.16 | 5480 | 6373 | 6266 |
| M84032 | 0.925 | 1.00 | 1.00 | 5481 | 4443 | 4418 |
| N81088 | 0.925 | 1.07 | 1.05 | 5482 | 5301 | 5113 |
| G85027 | 0.925 | 1.09 | 1.09 | 5483 | 5558 | 5598 |
| P81711 | 0.925 | 1.09 | 1.14 | 5484 | 5561 | 6043 |
| P81077 | 0.925 | 1.20 | 1.16 | 5485 | 6568 | 6280 |
| J82688 | 0.925 | 0.93 | 0.92 | 5486 | 3476 | 3338 |
| L83101 | 0.925 | 1.18 | 1.15 | 5487 | 6380 | 6168 |
| E86632 | 0.925 | 0.92 | 0.91 | 5488 | 3265 | 3284 |
| G82764 | 0.925 | 1.17 | 1.20 | 5489 | 6316 | 6550 |
| E85042 | 0.925 | 0.97 | 0.99 | 5490 | 4046 | 4295 |
| L84617 | 0.924 | 0.99 | 0.99 | 5491 | 4278 | 4340 |
| P92014 | 0.924 | 1.10 | 1.11 | 5492 | 5677 | 5781 |
| C84105 | 0.924 | 1.05 | 1.07 | 5493 | 5177 | 5381 |
| P82025 | 0.924 | 1.10 | 1.11 | 5494 | 5672 | 5782 |

| | | | | | | |
|---|---|---|---|---|---|---|
| E85736 | 0.924 | 0.99 | 0.98 | 5495 | 4308 | 4231 |
| P85019 | 0.924 | 1.12 | 1.13 | 5496 | 5817 | 6022 |
| M84010 | 0.924 | 1.08 | 1.08 | 5497 | 5483 | 5430 |
| Y01008 | 0.924 | 1.26 | 1.27 | 5498 | 6898 | 6928 |
| M87014 | 0.924 | 1.11 | 1.11 | 5499 | 5740 | 5749 |
| L83087 | 0.924 | 1.25 | 1.22 | 5500 | 6828 | 6679 |
| H81070 | 0.924 | 0.95 | 0.94 | 5501 | 3826 | 3652 |
| M86605 | 0.924 | 1.06 | 1.05 | 5502 | 5288 | 5176 |
| L83073 | 0.924 | 1.30 | 1.28 | 5503 | 7010 | 6944 |
| E82049 | 0.924 | 1.09 | 1.07 | 5504 | 5583 | 5403 |
| C81051 | 0.924 | 1.01 | 1.01 | 5505 | 4637 | 4694 |
| L83040 | 0.924 | 1.07 | 1.03 | 5506 | 5316 | 4919 |
| E85733 | 0.924 | 1.16 | 1.16 | 5507 | 6214 | 6302 |
| Y00212 | 0.924 | 0.88 | 0.88 | 5508 | 2771 | 2818 |
| M86018 | 0.923 | 1.00 | 0.99 | 5509 | 4472 | 4395 |
| B86011 | 0.923 | 1.10 | 1.12 | 5510 | 5653 | 5906 |
| P81134 | 0.923 | 1.22 | 1.24 | 5511 | 6704 | 6786 |
| F81144 | 0.923 | 1.03 | 1.04 | 5512 | 4874 | 5016 |
| N81022 | 0.923 | 1.00 | 0.99 | 5513 | 4521 | 4401 |
| D81009 | 0.923 | 1.00 | 0.99 | 5514 | 4468 | 4357 |
| J83042 | 0.923 | 1.03 | 1.02 | 5515 | 4869 | 4776 |
| J84013 | 0.923 | 1.26 | 1.25 | 5516 | 6869 | 6846 |
| B86648 | 0.923 | 1.24 | 1.24 | 5517 | 6773 | 6812 |
| C81049 | 0.923 | 1.18 | 1.17 | 5518 | 6478 | 6391 |
| F81169 | 0.923 | 0.96 | 0.95 | 5519 | 3963 | 3791 |
| M85086 | 0.923 | 1.12 | 1.10 | 5520 | 5883 | 5745 |
| L83064 | 0.923 | 1.12 | 1.15 | 5521 | 5917 | 6156 |
| J82010 | 0.923 | 1.13 | 1.11 | 5522 | 5966 | 5793 |
| F86644 | 0.923 | 0.97 | 0.99 | 5523 | 4132 | 4379 |
| G82802 | 0.923 | 0.97 | 0.98 | 5524 | 4080 | 4220 |
| L85066 | 0.922 | 1.08 | 1.08 | 5525 | 5446 | 5487 |
| E84058 | 0.922 | 1.09 | 1.06 | 5526 | 5557 | 5283 |
| C88622 | 0.922 | 1.24 | 1.26 | 5527 | 6820 | 6880 |
| E87045 | 0.922 | 0.91 | 0.91 | 5528 | 3230 | 3201 |
| L85032 | 0.922 | 1.04 | 1.05 | 5529 | 5031 | 5117 |
| J82116 | 0.922 | 1.02 | 0.99 | 5530 | 4749 | 4415 |
| F86025 | 0.922 | 1.04 | 1.02 | 5531 | 5025 | 4808 |
| B81089 | 0.922 | 1.11 | 1.15 | 5532 | 5760 | 6166 |
| P81695 | 0.922 | 1.02 | 1.04 | 5533 | 4808 | 4993 |
| N84627 | 0.921 | 1.19 | 1.18 | 5534 | 6502 | 6421 |
| J81022 | 0.921 | 1.34 | 1.32 | 5535 | 7128 | 7072 |
| H81101 | 0.921 | 0.95 | 0.93 | 5536 | 3814 | 3501 |
| C81634 | 0.921 | 1.13 | 1.12 | 5537 | 6013 | 5892 |
| P84026 | 0.921 | 1.02 | 1.05 | 5538 | 4850 | 5166 |
| J81042 | 0.921 | 1.03 | 1.03 | 5539 | 4883 | 4892 |
| C84125 | 0.921 | 1.07 | 1.05 | 5540 | 5346 | 5175 |
| L81102 | 0.921 | 1.12 | 1.10 | 5541 | 5873 | 5651 |
| J81030 | 0.921 | 1.14 | 1.13 | 5542 | 6055 | 5969 |
| F84009 | 0.921 | 0.92 | 0.91 | 5543 | 3379 | 3260 |
| B86670 | 0.921 | 1.06 | 1.07 | 5544 | 5308 | 5437 |

| | | | | | | |
|---|---|---|---|---|---|---|
| C83048 | 0.920 | 1.06 | 1.05 | 5545 | 5240 | 5103 |
| B81642 | 0.920 | 1.31 | 1.33 | 5546 | 7051 | 7096 |
| M91650 | 0.920 | 1.38 | 1.38 | 5547 | 7172 | 7175 |
| B81095 | 0.920 | 1.20 | 1.24 | 5548 | 6598 | 6787 |
| E82037 | 0.920 | 0.92 | 0.91 | 5549 | 3318 | 3300 |
| M84025 | 0.920 | 1.05 | 1.05 | 5550 | 5133 | 5174 |
| E82013 | 0.920 | 1.00 | 0.98 | 5551 | 4563 | 4329 |
| M83084 | 0.920 | 1.17 | 1.17 | 5552 | 6354 | 6389 |
| E84014 | 0.920 | 1.02 | 1.00 | 5553 | 4832 | 4520 |
| J82067 | 0.920 | 0.95 | 0.95 | 5554 | 3786 | 3816 |
| B86044 | 0.920 | 1.03 | 1.05 | 5555 | 4936 | 5162 |
| C84633 | 0.920 | 1.01 | 1.03 | 5556 | 4614 | 4969 |
| C82098 | 0.920 | 1.11 | 1.10 | 5557 | 5808 | 5758 |
| C81077 | 0.920 | 1.22 | 1.20 | 5558 | 6706 | 6620 |
| H84632 | 0.920 | 0.90 | 0.88 | 5559 | 3096 | 2901 |
| M85108 | 0.919 | 1.19 | 1.18 | 5560 | 6547 | 6480 |
| F81071 | 0.919 | 1.01 | 1.01 | 5561 | 4660 | 4636 |
| B81069 | 0.919 | 1.44 | 1.43 | 5562 | 7238 | 7222 |
| P81760 | 0.919 | 1.26 | 1.29 | 5563 | 6894 | 7008 |
| L84065 | 0.919 | 1.01 | 1.01 | 5564 | 4655 | 4675 |
| P81033 | 0.918 | 1.30 | 1.26 | 5565 | 7048 | 6905 |
| G84624 | 0.918 | 0.96 | 0.96 | 5566 | 4035 | 4009 |
| M83026 | 0.918 | 1.09 | 1.05 | 5567 | 5610 | 5173 |
| L82039 | 0.918 | 1.00 | 1.00 | 5568 | 4569 | 4637 |
| P81647 | 0.918 | 1.13 | 1.12 | 5569 | 5985 | 5979 |
| N82054 | 0.918 | 1.15 | 1.16 | 5570 | 6254 | 6341 |
| G85057 | 0.918 | 1.00 | 1.01 | 5571 | 4625 | 4740 |
| H82640 | 0.918 | 0.92 | 0.90 | 5572 | 3393 | 3175 |
| M81026 | 0.918 | 1.11 | 1.09 | 5573 | 5822 | 5627 |
| Y02633 | 0.918 | 1.16 | 1.16 | 5574 | 6286 | 6327 |
| L83002 | 0.918 | 1.24 | 1.20 | 5575 | 6803 | 6598 |
| L83658 | 0.918 | 1.01 | 1.03 | 5576 | 4692 | 4971 |
| L83084 | 0.917 | 1.15 | 1.11 | 5577 | 6179 | 5855 |
| L85062 | 0.917 | 1.19 | 1.15 | 5578 | 6548 | 6205 |
| H83608 | 0.917 | 1.00 | 0.99 | 5579 | 4532 | 4462 |
| E83020 | 0.917 | 0.94 | 0.92 | 5580 | 3740 | 3498 |
| N83622 | 0.917 | 1.31 | 1.30 | 5581 | 7079 | 7045 |
| B81029 | 0.917 | 1.18 | 1.18 | 5582 | 6503 | 6466 |
| L81023 | 0.917 | 0.96 | 0.97 | 5583 | 4073 | 4218 |
| E85024 | 0.917 | 1.13 | 1.11 | 5584 | 6059 | 5900 |
| D82078 | 0.916 | 1.05 | 1.04 | 5585 | 5186 | 5039 |
| M85065 | 0.916 | 1.11 | 1.10 | 5586 | 5846 | 5744 |
| K84023 | 0.916 | 1.20 | 1.17 | 5587 | 6628 | 6372 |
| H81003 | 0.916 | 1.01 | 1.00 | 5588 | 4742 | 4589 |
| J82196 | 0.916 | 1.20 | 1.18 | 5589 | 6601 | 6469 |
| F85020 | 0.916 | 0.94 | 0.93 | 5590 | 3714 | 3693 |
| E84042 | 0.916 | 0.98 | 0.97 | 5591 | 4377 | 4195 |
| J82124 | 0.915 | 0.99 | 0.96 | 5592 | 4397 | 4118 |
| E81012 | 0.915 | 1.02 | 1.01 | 5593 | 4834 | 4797 |
| K81657 | 0.915 | 0.90 | 0.89 | 5594 | 3118 | 3120 |

| | | | | | | |
|---|---|---|---|---|---|---|
| F85010 | 0.915 | 1.04 | 1.03 | 5595 | 5089 | 5022 |
| D81631 | 0.915 | 0.90 | 0.91 | 5596 | 3148 | 3318 |
| M85083 | 0.915 | 1.05 | 1.05 | 5597 | 5249 | 5195 |
| P81047 | 0.915 | 1.16 | 1.18 | 5598 | 6348 | 6514 |
| F86078 | 0.915 | 0.98 | 1.00 | 5599 | 4365 | 4614 |
| F84039 | 0.915 | 1.02 | 1.02 | 5600 | 4904 | 4897 |
| M85020 | 0.914 | 1.14 | 1.13 | 5601 | 6156 | 6045 |
| M83681 | 0.914 | 1.03 | 1.00 | 5602 | 4993 | 4578 |
| L81020 | 0.914 | 0.93 | 0.92 | 5603 | 3584 | 3489 |
| M83056 | 0.914 | 1.15 | 1.16 | 5604 | 6204 | 6309 |
| M83640 | 0.914 | 1.09 | 1.05 | 5605 | 5633 | 5193 |
| J81024 | 0.914 | 0.97 | 0.97 | 5606 | 4157 | 4182 |
| P81138 | 0.914 | 1.23 | 1.23 | 5607 | 6767 | 6805 |
| H85047 | 0.914 | 0.96 | 0.94 | 5608 | 4098 | 3783 |
| K81047 | 0.914 | 0.97 | 0.95 | 5609 | 4169 | 3902 |
| F81137 | 0.914 | 1.01 | 1.02 | 5610 | 4740 | 4849 |
| H84625 | 0.914 | 1.02 | 1.00 | 5611 | 4896 | 4649 |
| N82062 | 0.913 | 1.03 | 1.05 | 5612 | 5019 | 5248 |
| L85044 | 0.913 | 1.10 | 1.10 | 5613 | 5811 | 5820 |
| N81102 | 0.913 | 1.35 | 1.31 | 5614 | 7147 | 7070 |
| C81046 | 0.913 | 1.09 | 1.08 | 5615 | 5736 | 5569 |
| M85715 | 0.913 | 1.14 | 1.13 | 5616 | 6158 | 6088 |
| E82124 | 0.913 | 1.00 | 1.01 | 5617 | 4644 | 4762 |
| E82055 | 0.912 | 1.04 | 1.03 | 5618 | 5165 | 4986 |
| F84716 | 0.912 | 0.87 | 0.89 | 5619 | 2889 | 3108 |
| J82009 | 0.912 | 1.12 | 1.11 | 5620 | 5958 | 5864 |
| L85619 | 0.912 | 1.15 | 1.15 | 5621 | 6260 | 6271 |
| B81050 | 0.912 | 1.20 | 1.19 | 5622 | 6618 | 6584 |
| G82011 | 0.911 | 0.94 | 0.96 | 5623 | 3828 | 4203 |
| E85049 | 0.911 | 1.02 | 1.03 | 5624 | 4911 | 4976 |
| H81110 | 0.911 | 0.95 | 0.95 | 5625 | 3993 | 4034 |
| H84051 | 0.911 | 1.01 | 0.99 | 5626 | 4800 | 4596 |
| F81757 | 0.911 | 1.06 | 1.08 | 5627 | 5459 | 5556 |
| G81039 | 0.911 | 1.24 | 1.20 | 5628 | 6861 | 6631 |
| D82076 | 0.910 | 1.09 | 1.10 | 5629 | 5665 | 5876 |
| G83030 | 0.910 | 1.02 | 1.01 | 5630 | 4965 | 4836 |
| M83132 | 0.910 | 1.00 | 0.97 | 5631 | 4630 | 4250 |
| F84034 | 0.910 | 1.09 | 1.08 | 5632 | 5725 | 5620 |
| E85103 | 0.910 | 1.24 | 1.25 | 5633 | 6858 | 6890 |
| J81003 | 0.910 | 0.94 | 0.94 | 5634 | 3788 | 3858 |
| K81021 | 0.910 | 1.00 | 0.99 | 5635 | 4698 | 4528 |
| L81017 | 0.910 | 0.95 | 0.96 | 5636 | 3940 | 4147 |
| B86010 | 0.910 | 1.01 | 1.03 | 5637 | 4772 | 5044 |
| H81042 | 0.910 | 1.05 | 1.01 | 5638 | 5246 | 4820 |
| E81020 | 0.910 | 1.10 | 1.08 | 5639 | 5779 | 5661 |
| M81044 | 0.910 | 1.12 | 1.10 | 5640 | 6000 | 5846 |
| G82652 | 0.909 | 1.22 | 1.24 | 5641 | 6745 | 6877 |
| E85045 | 0.909 | 1.11 | 1.09 | 5642 | 5912 | 5671 |
| D83074 | 0.909 | 0.99 | 1.00 | 5643 | 4592 | 4727 |
| A81027 | 0.909 | 1.04 | 1.05 | 5644 | 5202 | 5340 |

| | | | | | | |
|---|---|---|---|---|---|---|
| P81740 | 0.909 | 1.02 | 1.00 | 5645 | 4976 | 4732 |
| E83010 | 0.909 | 1.00 | 0.98 | 5646 | 4649 | 4391 |
| G81680 | 0.909 | 1.16 | 1.18 | 5647 | 6414 | 6523 |
| C81007 | 0.909 | 1.08 | 1.08 | 5648 | 5632 | 5665 |
| E85029 | 0.909 | 1.02 | 1.04 | 5649 | 4995 | 5157 |
| E82017 | 0.909 | 0.94 | 0.93 | 5650 | 3917 | 3683 |
| C82022 | 0.909 | 1.00 | 0.98 | 5651 | 4622 | 4453 |
| L83035 | 0.909 | 1.15 | 1.14 | 5652 | 6319 | 6238 |
| C83040 | 0.909 | 1.16 | 1.14 | 5653 | 6382 | 6257 |
| C84094 | 0.909 | 1.15 | 1.15 | 5654 | 6334 | 6286 |
| J83041 | 0.908 | 1.00 | 1.00 | 5655 | 4718 | 4641 |
| J81017 | 0.908 | 1.08 | 1.06 | 5656 | 5644 | 5467 |
| C81015 | 0.908 | 1.16 | 1.15 | 5657 | 6395 | 6319 |
| K81072 | 0.908 | 0.95 | 0.96 | 5658 | 3981 | 4116 |
| P87627 | 0.908 | 1.05 | 1.08 | 5659 | 5366 | 5633 |
| M85757 | 0.908 | 1.00 | 0.99 | 5660 | 4647 | 4631 |
| F82033 | 0.908 | 1.03 | 1.03 | 5661 | 5124 | 5062 |
| M92643 | 0.908 | 0.95 | 0.96 | 5662 | 4019 | 4208 |
| B86058 | 0.908 | 1.13 | 1.14 | 5663 | 6100 | 6232 |
| G81029 | 0.908 | 1.22 | 1.20 | 5664 | 6768 | 6650 |
| J82145 | 0.908 | 1.02 | 1.01 | 5665 | 4979 | 4813 |
| B83019 | 0.908 | 0.99 | 1.01 | 5666 | 4566 | 4841 |
| C81035 | 0.908 | 1.17 | 1.16 | 5667 | 6455 | 6431 |
| P89022 | 0.908 | 1.16 | 1.18 | 5668 | 6402 | 6552 |
| J82088 | 0.907 | 1.11 | 1.12 | 5669 | 5937 | 6055 |
| E85732 | 0.907 | 1.00 | 0.99 | 5670 | 4773 | 4595 |
| C87018 | 0.907 | 1.12 | 1.13 | 5671 | 6002 | 6139 |
| N83637 | 0.907 | 1.12 | 1.13 | 5672 | 5993 | 6107 |
| B86018 | 0.907 | 1.14 | 1.16 | 5673 | 6258 | 6405 |
| E83653 | 0.906 | 0.98 | 0.96 | 5674 | 4485 | 4246 |
| N82617 | 0.906 | 0.89 | 0.91 | 5675 | 3137 | 3429 |
| H82051 | 0.906 | 0.97 | 0.98 | 5676 | 4295 | 4523 |
| M85075 | 0.906 | 1.12 | 1.11 | 5677 | 5997 | 5954 |
| L82024 | 0.906 | 1.34 | 1.33 | 5678 | 7143 | 7125 |
| A83006 | 0.906 | 1.23 | 1.23 | 5679 | 6850 | 6832 |
| H82016 | 0.905 | 1.17 | 1.16 | 5680 | 6465 | 6381 |
| Y00184 | 0.905 | 0.91 | 0.93 | 5681 | 3505 | 3802 |
| E84008 | 0.905 | 1.10 | 1.07 | 5682 | 5875 | 5590 |
| C83073 | 0.905 | 1.03 | 1.07 | 5683 | 5094 | 5530 |
| J82136 | 0.904 | 0.97 | 0.97 | 5684 | 4373 | 4372 |
| P81762 | 0.904 | 1.10 | 1.10 | 5685 | 5834 | 5842 |
| G81684 | 0.904 | 0.94 | 0.96 | 5686 | 3905 | 4248 |
| P87026 | 0.904 | 1.12 | 1.15 | 5687 | 6049 | 6304 |
| J82036 | 0.904 | 1.02 | 1.00 | 5688 | 5023 | 4724 |
| F83051 | 0.904 | 0.94 | 0.95 | 5689 | 3898 | 4062 |
| J83646 | 0.904 | 0.99 | 1.00 | 5690 | 4635 | 4792 |
| E86612 | 0.904 | 1.01 | 1.01 | 5691 | 4905 | 4871 |
| G84006 | 0.904 | 1.05 | 1.04 | 5692 | 5329 | 5269 |
| E84015 | 0.904 | 1.10 | 1.08 | 5693 | 5825 | 5647 |
| E85734 | 0.904 | 0.98 | 0.97 | 5694 | 4479 | 4355 |

| | | | | | | |
|---|---|---|---|---|---|---|
| J81637 | 0.904 | 1.09 | 1.07 | 5695 | 5757 | 5619 |
| M88630 | 0.903 | 1.08 | 1.10 | 5696 | 5676 | 5871 |
| M87016 | 0.903 | 1.15 | 1.13 | 5697 | 6321 | 6227 |
| E82045 | 0.903 | 0.99 | 0.97 | 5698 | 4577 | 4344 |
| L83095 | 0.903 | 1.15 | 1.12 | 5699 | 6359 | 6087 |
| M88625 | 0.903 | 1.01 | 1.03 | 5700 | 4888 | 5078 |
| J82018 | 0.903 | 1.01 | 1.00 | 5701 | 4908 | 4801 |
| G85685 | 0.902 | 1.04 | 1.04 | 5702 | 5318 | 5288 |
| B82012 | 0.902 | 1.02 | 1.02 | 5703 | 5045 | 5034 |
| D82017 | 0.902 | 1.14 | 1.14 | 5704 | 6212 | 6316 |
| J82123 | 0.902 | 1.03 | 1.01 | 5705 | 5200 | 4962 |
| C87603 | 0.902 | 1.17 | 1.17 | 5706 | 6515 | 6526 |
| H82027 | 0.902 | 0.98 | 0.96 | 5707 | 4459 | 4221 |
| C82052 | 0.902 | 1.13 | 1.12 | 5708 | 6177 | 6075 |
| M88616 | 0.902 | 1.25 | 1.25 | 5709 | 6924 | 6942 |
| P81017 | 0.902 | 1.04 | 1.03 | 5710 | 5303 | 5151 |
| C82034 | 0.902 | 0.97 | 0.97 | 5711 | 4320 | 4439 |
| E81045 | 0.901 | 1.10 | 1.10 | 5712 | 5928 | 5869 |
| A82038 | 0.901 | 1.05 | 1.04 | 5713 | 5442 | 5254 |
| D81625 | 0.900 | 0.90 | 0.90 | 5714 | 3322 | 3476 |
| P88015 | 0.900 | 1.02 | 1.03 | 5715 | 5095 | 5109 |
| F86018 | 0.900 | 1.00 | 1.02 | 5716 | 4812 | 5005 |
| A88009 | 0.900 | 1.07 | 1.07 | 5717 | 5572 | 5615 |
| F84086 | 0.900 | 1.07 | 1.05 | 5718 | 5576 | 5410 |
| P92630 | 0.900 | 1.10 | 1.11 | 5719 | 5957 | 6008 |
| N81121 | 0.900 | 1.11 | 1.09 | 5720 | 5995 | 5778 |
| C88005 | 0.900 | 1.06 | 1.08 | 5721 | 5525 | 5702 |
| L81644 | 0.900 | 0.99 | 0.98 | 5722 | 4724 | 4615 |
| M91014 | 0.899 | 1.25 | 1.27 | 5723 | 6925 | 6994 |
| H81074 | 0.899 | 0.99 | 0.97 | 5724 | 4681 | 4366 |
| M92629 | 0.899 | 1.10 | 1.11 | 5725 | 5911 | 6030 |
| M88005 | 0.899 | 1.06 | 1.07 | 5726 | 5485 | 5635 |
| M81616 | 0.899 | 1.03 | 1.06 | 5727 | 5169 | 5470 |
| H81116 | 0.899 | 1.06 | 1.03 | 5728 | 5506 | 5207 |
| G83673 | 0.899 | 1.01 | 1.01 | 5729 | 4973 | 4938 |
| G85081 | 0.899 | 0.94 | 0.95 | 5730 | 3978 | 4112 |
| H82053 | 0.899 | 1.14 | 1.15 | 5731 | 6257 | 6357 |
| E87739 | 0.899 | 0.94 | 0.93 | 5732 | 3955 | 3857 |
| H85057 | 0.899 | 0.83 | 0.82 | 5733 | 2540 | 2378 |
| L81088 | 0.899 | 1.11 | 1.11 | 5734 | 6008 | 6051 |
| L83146 | 0.899 | 1.09 | 1.09 | 5735 | 5852 | 5857 |
| B83624 | 0.899 | 1.04 | 1.06 | 5736 | 5364 | 5524 |
| H81086 | 0.899 | 0.96 | 0.95 | 5737 | 4264 | 4148 |
| F83052 | 0.899 | 0.98 | 0.97 | 5738 | 4496 | 4427 |
| L85035 | 0.899 | 1.09 | 1.09 | 5739 | 5865 | 5904 |
| J82046 | 0.899 | 1.02 | 1.00 | 5740 | 5081 | 4887 |
| C81005 | 0.898 | 1.20 | 1.19 | 5741 | 6691 | 6639 |
| H81046 | 0.898 | 0.96 | 0.95 | 5742 | 4330 | 4151 |
| N81040 | 0.898 | 1.11 | 1.08 | 5743 | 5996 | 5754 |
| H81129 | 0.898 | 0.91 | 0.89 | 5744 | 3619 | 3271 |

| | | | | | | |
|---|---|---|---|---|---|---|
| F81205 | 0.898 | 1.31 | 1.29 | 5745 | 7096 | 7055 |
| F82610 | 0.898 | 1.04 | 1.04 | 5746 | 5350 | 5295 |
| L85019 | 0.897 | 1.20 | 1.18 | 5747 | 6697 | 6624 |
| Y03755 | 0.897 | 0.90 | 0.90 | 5748 | 3398 | 3456 |
| C81016 | 0.897 | 1.10 | 1.10 | 5749 | 5930 | 5967 |
| G82649 | 0.897 | 1.16 | 1.16 | 5750 | 6477 | 6473 |
| P82003 | 0.897 | 1.08 | 1.10 | 5751 | 5758 | 5945 |
| E83031 | 0.897 | 0.94 | 0.92 | 5752 | 4058 | 3819 |
| C81073 | 0.896 | 1.10 | 1.10 | 5753 | 5908 | 5928 |
| C81003 | 0.896 | 1.09 | 1.08 | 5754 | 5866 | 5779 |
| A88611 | 0.896 | 1.24 | 1.23 | 5755 | 6919 | 6879 |
| A86033 | 0.896 | 0.95 | 0.98 | 5756 | 4232 | 4537 |
| L83666 | 0.896 | 1.18 | 1.16 | 5757 | 6606 | 6520 |
| P84042 | 0.896 | 1.10 | 1.12 | 5758 | 5991 | 6148 |
| D82624 | 0.896 | 0.97 | 0.96 | 5759 | 4492 | 4382 |
| M91621 | 0.896 | 1.17 | 1.19 | 5760 | 6563 | 6695 |
| C84030 | 0.896 | 1.09 | 1.09 | 5761 | 5881 | 5860 |
| G82139 | 0.895 | 1.08 | 1.10 | 5762 | 5766 | 6007 |
| L84078 | 0.895 | 0.99 | 0.99 | 5763 | 4743 | 4728 |
| J82042 | 0.895 | 1.06 | 1.05 | 5764 | 5535 | 5462 |
| L85033 | 0.895 | 1.15 | 1.15 | 5765 | 6400 | 6399 |
| M91626 | 0.895 | 1.15 | 1.17 | 5766 | 6446 | 6543 |
| H84014 | 0.894 | 0.88 | 0.87 | 5767 | 3176 | 3039 |
| B82008 | 0.894 | 1.13 | 1.12 | 5768 | 6228 | 6182 |
| F81684 | 0.894 | 1.19 | 1.19 | 5769 | 6651 | 6707 |
| Y02212 | 0.894 | 1.19 | 1.18 | 5770 | 6696 | 6646 |
| P81129 | 0.894 | 1.13 | 1.10 | 5771 | 6225 | 6024 |
| G84002 | 0.894 | 0.93 | 0.92 | 5772 | 3874 | 3861 |
| K82023 | 0.894 | 0.99 | 0.98 | 5773 | 4806 | 4593 |
| G82098 | 0.894 | 1.05 | 1.05 | 5774 | 5468 | 5497 |
| K81097 | 0.894 | 0.95 | 0.93 | 5775 | 4180 | 4011 |
| A86035 | 0.894 | 1.17 | 1.19 | 5776 | 6599 | 6684 |
| H83025 | 0.894 | 1.00 | 0.99 | 5777 | 4889 | 4785 |
| L82061 | 0.893 | 1.14 | 1.14 | 5778 | 6314 | 6360 |
| M81014 | 0.893 | 1.11 | 1.10 | 5779 | 6102 | 5963 |
| L83148 | 0.893 | 1.21 | 1.21 | 5780 | 6816 | 6817 |
| E85657 | 0.893 | 0.92 | 0.93 | 5781 | 3829 | 3911 |
| C82067 | 0.893 | 1.10 | 1.10 | 5782 | 5921 | 5989 |
| K82618 | 0.893 | 0.96 | 0.94 | 5783 | 4300 | 4094 |
| C84703 | 0.893 | 0.97 | 0.96 | 5784 | 4494 | 4316 |
| N81033 | 0.893 | 0.98 | 0.97 | 5785 | 4626 | 4546 |
| C81062 | 0.892 | 1.10 | 1.10 | 5786 | 6004 | 5996 |
| G81076 | 0.892 | 0.99 | 1.01 | 5787 | 4758 | 5024 |
| J83062 | 0.892 | 1.13 | 1.12 | 5788 | 6250 | 6159 |
| C82121 | 0.892 | 1.11 | 1.11 | 5789 | 6069 | 6064 |
| P87004 | 0.892 | 1.26 | 1.28 | 5790 | 7015 | 7054 |
| H81088 | 0.892 | 0.97 | 0.97 | 5791 | 4543 | 4551 |
| N84611 | 0.892 | 1.31 | 1.30 | 5792 | 7118 | 7083 |
| E83629 | 0.892 | 1.14 | 1.10 | 5793 | 6331 | 5978 |
| B86086 | 0.891 | 0.91 | 0.93 | 5794 | 3711 | 4012 |

| | | | | | | |
|---|---|---|---|---|---|---|
| P85606 | 0.891 | 1.05 | 1.07 | 5795 | 5542 | 5726 |
| L85056 | 0.891 | 1.14 | 1.14 | 5796 | 6374 | 6407 |
| F86081 | 0.891 | 0.99 | 0.97 | 5797 | 4796 | 4588 |
| K84002 | 0.891 | 1.02 | 0.99 | 5798 | 5115 | 4793 |
| K81014 | 0.891 | 0.98 | 0.99 | 5799 | 4667 | 4777 |
| L83009 | 0.891 | 1.20 | 1.21 | 5800 | 6736 | 6802 |
| F84677 | 0.891 | 1.08 | 1.07 | 5801 | 5835 | 5674 |
| E82075 | 0.891 | 1.10 | 1.09 | 5802 | 5961 | 5888 |
| E81007 | 0.890 | 1.03 | 1.02 | 5803 | 5227 | 5164 |
| M83093 | 0.890 | 1.08 | 1.04 | 5804 | 5828 | 5440 |
| M82014 | 0.890 | 1.10 | 1.10 | 5805 | 5983 | 6046 |
| G84001 | 0.890 | 0.93 | 0.93 | 5806 | 3949 | 3948 |
| Y02164 | 0.890 | 1.07 | 1.06 | 5807 | 5704 | 5652 |
| C82092 | 0.890 | 1.07 | 1.07 | 5808 | 5687 | 5719 |
| L85009 | 0.890 | 1.21 | 1.19 | 5809 | 6800 | 6723 |
| M83691 | 0.890 | 1.06 | 1.08 | 5810 | 5643 | 5819 |
| K81007 | 0.890 | 0.98 | 0.99 | 5811 | 4713 | 4755 |
| L83050 | 0.890 | 1.22 | 1.21 | 5812 | 6871 | 6835 |
| G85102 | 0.889 | 1.00 | 1.01 | 5813 | 4886 | 4992 |
| C83051 | 0.889 | 1.01 | 1.05 | 5814 | 5110 | 5528 |
| E86015 | 0.889 | 1.05 | 1.03 | 5815 | 5486 | 5346 |
| E82658 | 0.889 | 0.95 | 0.93 | 5816 | 4248 | 3992 |
| B81070 | 0.889 | 1.44 | 1.42 | 5817 | 7257 | 7245 |
| M84055 | 0.889 | 1.13 | 1.13 | 5818 | 6338 | 6307 |
| N81009 | 0.889 | 1.09 | 1.07 | 5819 | 5901 | 5729 |
| E83018 | 0.888 | 1.01 | 0.99 | 5820 | 5049 | 4795 |
| K81062 | 0.888 | 0.94 | 0.95 | 5821 | 4217 | 4296 |
| K81060 | 0.888 | 0.91 | 0.91 | 5822 | 3738 | 3741 |
| M92006 | 0.888 | 1.12 | 1.14 | 5823 | 6231 | 6402 |
| E86038 | 0.888 | 0.95 | 0.95 | 5824 | 4339 | 4288 |
| C88091 | 0.888 | 1.36 | 1.36 | 5825 | 7183 | 7192 |
| E83032 | 0.888 | 0.94 | 0.93 | 5826 | 4225 | 3969 |
| M83650 | 0.887 | 1.01 | 1.05 | 5827 | 5099 | 5506 |
| M82016 | 0.887 | 1.16 | 1.16 | 5828 | 6534 | 6535 |
| N81055 | 0.887 | 1.10 | 1.08 | 5829 | 6010 | 5814 |
| F82016 | 0.887 | 1.03 | 1.03 | 5830 | 5340 | 5324 |
| M88637 | 0.887 | 1.23 | 1.26 | 5831 | 6917 | 7005 |
| L84067 | 0.887 | 1.07 | 1.06 | 5832 | 5767 | 5676 |
| J81031 | 0.886 | 1.04 | 1.04 | 5833 | 5428 | 5445 |
| J81628 | 0.886 | 1.20 | 1.18 | 5834 | 6789 | 6668 |
| F86049 | 0.886 | 0.98 | 0.99 | 5835 | 4706 | 4899 |
| L85065 | 0.886 | 1.19 | 1.17 | 5836 | 6716 | 6638 |
| A86012 | 0.886 | 1.13 | 1.14 | 5837 | 6304 | 6430 |
| G82088 | 0.885 | 1.02 | 1.03 | 5838 | 5262 | 5357 |
| P91603 | 0.885 | 1.05 | 1.06 | 5839 | 5527 | 5603 |
| B81692 | 0.885 | 0.99 | 1.04 | 5840 | 4918 | 5406 |
| P83001 | 0.885 | 1.02 | 1.03 | 5841 | 5271 | 5362 |
| M82003 | 0.885 | 1.11 | 1.12 | 5842 | 6130 | 6231 |
| H82026 | 0.885 | 0.98 | 1.00 | 5843 | 4714 | 4944 |
| C81097 | 0.884 | 1.14 | 1.12 | 5844 | 6434 | 6242 |

| | | | | | | |
|---|---|---|---|---|---|---|
| G83058 | 0.884 | 1.07 | 1.07 | 5845 | 5800 | 5770 |
| E85115 | 0.884 | 1.10 | 1.08 | 5846 | 6056 | 5894 |
| C84676 | 0.884 | 1.01 | 1.03 | 5847 | 5100 | 5363 |
| K84049 | 0.884 | 0.84 | 0.84 | 5848 | 2789 | 2769 |
| J82012 | 0.884 | 1.17 | 1.15 | 5849 | 6620 | 6509 |
| E81002 | 0.884 | 1.09 | 1.08 | 5850 | 5920 | 5921 |
| C85015 | 0.884 | 1.24 | 1.24 | 5851 | 6955 | 6966 |
| J84007 | 0.884 | 1.23 | 1.22 | 5852 | 6901 | 6885 |
| G81006 | 0.884 | 1.11 | 1.12 | 5853 | 6147 | 6295 |
| B84005 | 0.884 | 1.10 | 1.12 | 5854 | 6101 | 6275 |
| L83043 | 0.883 | 1.02 | 1.02 | 5855 | 5243 | 5292 |
| G85039 | 0.883 | 0.95 | 0.96 | 5856 | 4372 | 4435 |
| G85032 | 0.883 | 1.00 | 1.01 | 5857 | 5033 | 5136 |
| L84002 | 0.883 | 1.13 | 1.12 | 5858 | 6351 | 6282 |
| E87038 | 0.883 | 1.03 | 1.02 | 5859 | 5360 | 5206 |
| P83015 | 0.883 | 1.28 | 1.27 | 5860 | 7070 | 7046 |
| P89017 | 0.883 | 1.19 | 1.21 | 5861 | 6766 | 6875 |
| C81030 | 0.882 | 1.17 | 1.16 | 5862 | 6644 | 6611 |
| P92648 | 0.882 | 1.18 | 1.18 | 5863 | 6680 | 6706 |
| J82168 | 0.882 | 1.13 | 1.13 | 5864 | 6317 | 6388 |
| K82046 | 0.882 | 0.94 | 0.92 | 5865 | 4193 | 3961 |
| L85022 | 0.882 | 1.17 | 1.16 | 5866 | 6613 | 6602 |
| J82177 | 0.882 | 1.10 | 1.11 | 5867 | 6073 | 6155 |
| M84020 | 0.882 | 1.02 | 1.02 | 5868 | 5312 | 5215 |
| G81073 | 0.882 | 1.11 | 1.13 | 5869 | 6198 | 6371 |
| C88049 | 0.882 | 1.14 | 1.15 | 5870 | 6454 | 6547 |
| J82072 | 0.882 | 1.04 | 1.03 | 5871 | 5523 | 5385 |
| G85042 | 0.881 | 1.00 | 1.00 | 5872 | 5072 | 5021 |
| M86022 | 0.881 | 1.06 | 1.04 | 5873 | 5650 | 5529 |
| P89033 | 0.881 | 1.27 | 1.28 | 5874 | 7062 | 7081 |
| M85717 | 0.881 | 1.45 | 1.40 | 5875 | 7261 | 7237 |
| C84039 | 0.881 | 0.98 | 1.00 | 5876 | 4804 | 5073 |
| K81052 | 0.881 | 1.03 | 1.03 | 5877 | 5370 | 5353 |
| C81039 | 0.881 | 1.12 | 1.11 | 5878 | 6297 | 6244 |
| K84033 | 0.881 | 1.03 | 1.00 | 5879 | 5362 | 5002 |
| L84006 | 0.880 | 0.94 | 0.93 | 5880 | 4197 | 4189 |
| H81010 | 0.880 | 0.88 | 0.86 | 5881 | 3470 | 3097 |
| J81009 | 0.880 | 1.21 | 1.20 | 5882 | 6839 | 6797 |
| E85060 | 0.880 | 1.04 | 1.03 | 5883 | 5507 | 5400 |
| E85640 | 0.880 | 0.92 | 0.92 | 5884 | 3962 | 4045 |
| K81630 | 0.880 | 0.97 | 0.95 | 5885 | 4606 | 4421 |
| G82041 | 0.880 | 0.93 | 0.94 | 5886 | 4204 | 4253 |
| M83623 | 0.880 | 1.08 | 1.11 | 5887 | 5888 | 6229 |
| G83039 | 0.880 | 1.16 | 1.15 | 5888 | 6617 | 6568 |
| H84017 | 0.879 | 0.88 | 0.87 | 5889 | 3452 | 3265 |
| C82021 | 0.879 | 1.14 | 1.13 | 5890 | 6463 | 6413 |
| M85176 | 0.879 | 1.22 | 1.21 | 5891 | 6893 | 6845 |
| G81667 | 0.879 | 1.03 | 1.05 | 5892 | 5401 | 5653 |
| H85656 | 0.879 | 0.97 | 0.96 | 5893 | 4619 | 4494 |
| M92007 | 0.879 | 1.22 | 1.24 | 5894 | 6902 | 6964 |

| | | | | | | |
|---|---|---|---|---|---|---|
| N81651 | 0.879 | 0.97 | 0.98 | 5895 | 4654 | 4848 |
| N81046 | 0.879 | 1.06 | 1.04 | 5896 | 5714 | 5522 |
| Y01922 | 0.879 | 1.13 | 1.13 | 5897 | 6388 | 6403 |
| H81020 | 0.879 | 0.91 | 0.90 | 5898 | 3891 | 3762 |
| K82015 | 0.879 | 1.08 | 1.07 | 5899 | 5931 | 5829 |
| G83016 | 0.878 | 0.95 | 0.95 | 5900 | 4428 | 4424 |
| E82064 | 0.878 | 0.97 | 0.97 | 5901 | 4734 | 4646 |
| M82004 | 0.878 | 1.10 | 1.10 | 5902 | 6135 | 6158 |
| E82129 | 0.878 | 0.93 | 0.93 | 5903 | 4198 | 4168 |
| H82072 | 0.878 | 1.01 | 1.01 | 5904 | 5212 | 5202 |
| J82005 | 0.878 | 0.99 | 0.99 | 5905 | 4943 | 4936 |
| N83007 | 0.878 | 1.29 | 1.29 | 5906 | 7103 | 7109 |
| A82039 | 0.877 | 1.17 | 1.20 | 5907 | 6637 | 6806 |
| M92039 | 0.877 | 1.10 | 1.11 | 5908 | 6109 | 6233 |
| P89007 | 0.877 | 1.21 | 1.23 | 5909 | 6882 | 6960 |
| J82138 | 0.877 | 0.98 | 0.95 | 5910 | 4877 | 4461 |
| C82014 | 0.877 | 1.13 | 1.12 | 5911 | 6365 | 6291 |
| E85114 | 0.877 | 1.09 | 1.09 | 5912 | 6035 | 6017 |
| K81647 | 0.877 | 0.86 | 0.87 | 5913 | 3196 | 3339 |
| E85119 | 0.877 | 1.11 | 1.12 | 5914 | 6229 | 6285 |
| K81613 | 0.877 | 1.01 | 1.01 | 5915 | 5170 | 5232 |
| K84044 | 0.877 | 0.98 | 0.97 | 5916 | 4809 | 4736 |
| N81038 | 0.877 | 1.11 | 1.09 | 5917 | 6261 | 6079 |
| E85052 | 0.876 | 1.01 | 1.00 | 5918 | 5228 | 5127 |
| G85086 | 0.876 | 0.96 | 0.97 | 5919 | 4584 | 4717 |
| P81667 | 0.876 | 1.14 | 1.12 | 5920 | 6475 | 6356 |
| P87660 | 0.876 | 1.03 | 1.06 | 5921 | 5421 | 5725 |
| H82086 | 0.876 | 1.15 | 1.14 | 5922 | 6554 | 6507 |
| D82102 | 0.876 | 1.17 | 1.18 | 5923 | 6669 | 6733 |
| C83047 | 0.876 | 1.06 | 1.05 | 5924 | 5747 | 5677 |
| E85125 | 0.876 | 0.89 | 0.91 | 5925 | 3606 | 3876 |
| Y00075 | 0.875 | 1.14 | 1.12 | 5926 | 6495 | 6364 |
| J81083 | 0.875 | 1.07 | 1.06 | 5927 | 5838 | 5740 |
| P84035 | 0.875 | 0.95 | 0.97 | 5928 | 4462 | 4782 |
| Y01011 | 0.875 | 0.96 | 0.96 | 5929 | 4658 | 4573 |
| J82156 | 0.875 | 1.13 | 1.12 | 5930 | 6416 | 6305 |
| P81043 | 0.875 | 1.33 | 1.36 | 5931 | 7169 | 7206 |
| F86028 | 0.875 | 1.01 | 1.00 | 5932 | 5267 | 5029 |
| Y01051 | 0.875 | 1.07 | 1.07 | 5933 | 5840 | 5867 |
| H82040 | 0.874 | 0.99 | 0.99 | 5934 | 5034 | 4982 |
| M85163 | 0.874 | 1.05 | 1.04 | 5935 | 5683 | 5558 |
| M88012 | 0.874 | 1.14 | 1.15 | 5936 | 6483 | 6558 |
| K82016 | 0.874 | 1.03 | 1.02 | 5937 | 5448 | 5391 |
| L82006 | 0.874 | 1.02 | 1.02 | 5938 | 5343 | 5401 |
| L81127 | 0.874 | 0.96 | 0.96 | 5939 | 4666 | 4697 |
| B82053 | 0.874 | 1.11 | 1.07 | 5940 | 6227 | 5931 |
| L83039 | 0.874 | 1.14 | 1.16 | 5941 | 6520 | 6662 |
| G82653 | 0.874 | 0.94 | 0.96 | 5942 | 4333 | 4691 |
| F81032 | 0.873 | 1.11 | 1.11 | 5943 | 6253 | 6243 |
| F85701 | 0.873 | 1.07 | 1.07 | 5944 | 5916 | 5908 |

| | | | | | | |
|---|---|---|---|---|---|---|
| E83650 | 0.873 | 0.98 | 0.96 | 5945 | 4957 | 4715 |
| E83027 | 0.873 | 0.93 | 0.91 | 5946 | 4214 | 4003 |
| L83075 | 0.872 | 1.06 | 1.06 | 5947 | 5752 | 5816 |
| P89030 | 0.872 | 1.21 | 1.23 | 5948 | 6897 | 6961 |
| F85029 | 0.872 | 1.02 | 1.01 | 5949 | 5365 | 5310 |
| H82052 | 0.872 | 0.99 | 1.01 | 5950 | 5011 | 5234 |
| L82029 | 0.872 | 1.04 | 1.05 | 5951 | 5591 | 5648 |
| E84032 | 0.872 | 1.00 | 0.98 | 5952 | 5108 | 4934 |
| L83044 | 0.872 | 1.18 | 1.19 | 5953 | 6741 | 6820 |
| C84619 | 0.872 | 1.05 | 1.08 | 5954 | 5742 | 5981 |
| E84657 | 0.872 | 1.04 | 1.02 | 5955 | 5618 | 5395 |
| Y02378 | 0.872 | 1.24 | 1.24 | 5956 | 6992 | 6986 |
| F81117 | 0.872 | 1.00 | 1.00 | 5957 | 5199 | 5159 |
| E85128 | 0.872 | 0.90 | 0.92 | 5958 | 3895 | 4176 |
| Y01281 | 0.871 | 1.21 | 1.19 | 5959 | 6875 | 6815 |
| C81031 | 0.871 | 1.17 | 1.16 | 5960 | 6679 | 6641 |
| N82108 | 0.871 | 0.98 | 1.01 | 5961 | 4982 | 5267 |
| P81062 | 0.871 | 1.17 | 1.14 | 5962 | 6720 | 6539 |
| H81122 | 0.871 | 0.93 | 0.91 | 5963 | 4242 | 3917 |
| C82086 | 0.871 | 1.05 | 1.05 | 5964 | 5693 | 5670 |
| G85113 | 0.871 | 1.02 | 1.03 | 5965 | 5425 | 5494 |
| M85174 | 0.871 | 1.02 | 1.02 | 5966 | 5429 | 5380 |
| M86006 | 0.871 | 0.98 | 0.97 | 5967 | 4879 | 4839 |
| H81663 | 0.870 | 0.88 | 0.87 | 5968 | 3534 | 3392 |
| Y00347 | 0.870 | 1.11 | 1.08 | 5969 | 6326 | 6047 |
| G85041 | 0.870 | 0.92 | 0.93 | 5970 | 4085 | 4247 |
| E82085 | 0.870 | 0.97 | 0.98 | 5971 | 4802 | 4933 |
| L84025 | 0.870 | 1.01 | 1.01 | 5972 | 5239 | 5277 |
| P91007 | 0.870 | 1.08 | 1.09 | 5973 | 6020 | 6085 |
| B82104 | 0.869 | 0.96 | 0.96 | 5974 | 4720 | 4711 |
| F84097 | 0.869 | 1.03 | 1.01 | 5975 | 5497 | 5349 |
| L83026 | 0.869 | 1.17 | 1.16 | 5976 | 6694 | 6676 |
| M84013 | 0.869 | 1.00 | 1.00 | 5977 | 5209 | 5180 |
| M85153 | 0.869 | 1.15 | 1.15 | 5978 | 6616 | 6574 |
| H81045 | 0.869 | 0.99 | 0.96 | 5979 | 5036 | 4747 |
| H82010 | 0.868 | 1.05 | 1.04 | 5980 | 5680 | 5628 |
| F82002 | 0.868 | 0.99 | 0.99 | 5981 | 5120 | 5080 |
| H81066 | 0.868 | 0.96 | 0.93 | 5982 | 4634 | 4306 |
| C84085 | 0.867 | 1.07 | 1.09 | 5983 | 5895 | 6115 |
| G82634 | 0.867 | 1.07 | 1.08 | 5984 | 5949 | 6063 |
| F81083 | 0.867 | 1.06 | 1.06 | 5985 | 5815 | 5812 |
| L83030 | 0.867 | 1.16 | 1.17 | 5986 | 6646 | 6720 |
| K82006 | 0.867 | 1.03 | 1.01 | 5987 | 5546 | 5312 |
| C84682 | 0.867 | 0.94 | 0.97 | 5988 | 4503 | 4852 |
| L83644 | 0.867 | 1.10 | 1.10 | 5989 | 6235 | 6278 |
| F86675 | 0.866 | 0.94 | 0.92 | 5990 | 4435 | 4235 |
| G84009 | 0.866 | 1.01 | 1.00 | 5991 | 5280 | 5268 |
| G81083 | 0.866 | 0.99 | 1.01 | 5992 | 5091 | 5303 |
| B86069 | 0.866 | 0.90 | 0.91 | 5993 | 3842 | 4113 |
| H82036 | 0.866 | 1.06 | 1.04 | 5994 | 5843 | 5643 |

| | | | | | | |
|---|---|---|---|---|---|---|
| G85028 | 0.866 | 0.99 | 0.99 | 5995 | 5069 | 5140 |
| G84609 | 0.866 | 1.07 | 1.05 | 5996 | 5907 | 5796 |
| K84071 | 0.865 | 1.03 | 1.01 | 5997 | 5595 | 5359 |
| J82633 | 0.865 | 1.11 | 1.10 | 5998 | 6299 | 6239 |
| E82105 | 0.865 | 1.04 | 1.03 | 5999 | 5670 | 5526 |
| Y01964 | 0.865 | 0.92 | 0.91 | 6000 | 4255 | 4056 |
| L83016 | 0.865 | 0.92 | 0.90 | 6001 | 4200 | 3898 |
| M81621 | 0.865 | 1.07 | 1.05 | 6002 | 5902 | 5813 |
| F81742 | 0.865 | 0.83 | 0.84 | 6003 | 2988 | 3133 |
| P84018 | 0.865 | 0.85 | 0.87 | 6004 | 3139 | 3509 |
| C82639 | 0.864 | 1.04 | 1.04 | 6005 | 5695 | 5709 |
| P81177 | 0.864 | 1.04 | 1.06 | 6005 | 5690 | 5844 |
| E84704 | 0.864 | 1.10 | 1.08 | 6006 | 6237 | 6111 |
| D82628 | 0.864 | 1.16 | 1.13 | 6007 | 6664 | 6517 |
| E82069 | 0.864 | 0.92 | 0.91 | 6008 | 4249 | 4071 |
| M85735 | 0.864 | 1.18 | 1.17 | 6009 | 6790 | 6760 |
| K84624 | 0.864 | 0.97 | 0.94 | 6010 | 4831 | 4533 |
| E82643 | 0.864 | 1.01 | 1.00 | 6011 | 5397 | 5270 |
| B81061 | 0.863 | 1.08 | 1.07 | 6012 | 6029 | 6015 |
| L81060 | 0.863 | 1.01 | 1.00 | 6013 | 5433 | 5241 |
| E84018 | 0.863 | 1.06 | 1.04 | 6014 | 5891 | 5675 |
| B86682 | 0.863 | 1.03 | 1.04 | 6015 | 5544 | 5707 |
| H82041 | 0.863 | 1.15 | 1.14 | 6016 | 6609 | 6573 |
| Y00092 | 0.863 | 0.86 | 0.87 | 6017 | 3292 | 3599 |
| D83022 | 0.862 | 1.18 | 1.18 | 6018 | 6775 | 6777 |
| J83645 | 0.862 | 1.02 | 1.03 | 6019 | 5515 | 5617 |
| F83674 | 0.862 | 1.05 | 1.06 | 6020 | 5791 | 5891 |
| G84039 | 0.862 | 1.00 | 0.99 | 6021 | 5252 | 5199 |
| Y00033 | 0.862 | 0.91 | 0.91 | 6022 | 4064 | 4190 |
| M82033 | 0.861 | 1.06 | 1.06 | 6023 | 5856 | 5930 |
| J82049 | 0.861 | 1.08 | 1.04 | 6024 | 6080 | 5687 |
| G82152 | 0.860 | 1.04 | 1.04 | 6025 | 5696 | 5713 |
| F85675 | 0.860 | 0.93 | 0.94 | 6026 | 4389 | 4553 |
| C88028 | 0.860 | 0.95 | 0.97 | 6027 | 4648 | 4952 |
| G82226 | 0.860 | 1.04 | 1.07 | 6028 | 5763 | 6040 |
| M87005 | 0.860 | 1.12 | 1.12 | 6029 | 6474 | 6454 |
| J81016 | 0.860 | 1.06 | 1.04 | 6030 | 5877 | 5732 |
| H82070 | 0.860 | 1.09 | 1.06 | 6031 | 6164 | 5919 |
| H85090 | 0.860 | 0.98 | 0.96 | 6032 | 5066 | 4861 |
| C82040 | 0.859 | 1.02 | 1.02 | 6033 | 5547 | 5514 |
| E85057 | 0.859 | 0.97 | 0.98 | 6034 | 4989 | 5015 |
| K84622 | 0.859 | 0.97 | 0.94 | 6035 | 4954 | 4628 |
| N84008 | 0.859 | 1.13 | 1.13 | 6036 | 6539 | 6538 |
| H81632 | 0.859 | 1.10 | 1.09 | 6037 | 6315 | 6202 |
| C82015 | 0.859 | 1.32 | 1.31 | 6038 | 7174 | 7170 |
| G85006 | 0.859 | 0.99 | 0.99 | 6039 | 5196 | 5194 |
| P91013 | 0.859 | 1.08 | 1.08 | 6040 | 6090 | 6129 |
| H83013 | 0.859 | 1.02 | 1.02 | 6041 | 5539 | 5476 |
| F81744 | 0.859 | 1.17 | 1.18 | 6042 | 6747 | 6793 |
| E83041 | 0.859 | 0.91 | 0.90 | 6043 | 4131 | 3980 |

| | | | | | | |
|---|---|---|---|---|---|---|
| F84046 | 0.858 | 1.10 | 1.10 | 6044 | 6280 | 6276 |
| F81003 | 0.858 | 1.11 | 1.12 | 6045 | 6378 | 6459 |
| N84038 | 0.858 | 1.25 | 1.24 | 6046 | 7072 | 7020 |
| M85064 | 0.858 | 0.94 | 0.94 | 6047 | 4590 | 4513 |
| E82084 | 0.858 | 0.97 | 0.96 | 6048 | 4909 | 4773 |
| B86066 | 0.858 | 1.03 | 1.05 | 6049 | 5635 | 5863 |
| K82078 | 0.858 | 0.96 | 0.94 | 6050 | 4810 | 4576 |
| G82154 | 0.858 | 1.20 | 1.22 | 6051 | 6887 | 6981 |
| D82022 | 0.857 | 1.11 | 1.10 | 6052 | 6427 | 6294 |
| B82095 | 0.857 | 1.06 | 1.05 | 6053 | 5914 | 5882 |
| L81080 | 0.857 | 1.03 | 1.02 | 6054 | 5612 | 5509 |
| J81066 | 0.857 | 1.04 | 1.04 | 6055 | 5765 | 5727 |
| L83065 | 0.857 | 1.17 | 1.19 | 6056 | 6791 | 6888 |
| G85045 | 0.856 | 0.96 | 0.97 | 6057 | 4840 | 4943 |
| L81648 | 0.856 | 1.07 | 1.08 | 6058 | 6084 | 6183 |
| F84741 | 0.856 | 1.13 | 1.11 | 6059 | 6536 | 6406 |
| P85612 | 0.856 | 1.21 | 1.24 | 6060 | 6958 | 7024 |
| E85649 | 0.856 | 0.93 | 0.95 | 6061 | 4448 | 4720 |
| F81672 | 0.856 | 1.18 | 1.18 | 6062 | 6825 | 6819 |
| G82082 | 0.856 | 0.91 | 0.92 | 6063 | 4177 | 4342 |
| E81047 | 0.855 | 0.95 | 0.94 | 6064 | 4677 | 4634 |
| J83629 | 0.855 | 1.13 | 1.12 | 6065 | 6549 | 6533 |
| E86016 | 0.855 | 1.03 | 1.02 | 6066 | 5662 | 5597 |
| A89034 | 0.855 | 1.00 | 1.02 | 6067 | 5380 | 5540 |
| D82047 | 0.855 | 1.13 | 1.14 | 6068 | 6586 | 6630 |
| B82003 | 0.855 | 0.99 | 0.98 | 6069 | 5244 | 5130 |
| F84672 | 0.855 | 1.32 | 1.29 | 6070 | 7181 | 7145 |
| N82641 | 0.854 | 1.07 | 1.09 | 6071 | 6089 | 6240 |
| G85050 | 0.854 | 1.06 | 1.05 | 6072 | 5938 | 5917 |
| H82012 | 0.854 | 1.23 | 1.24 | 6073 | 7016 | 7036 |
| G82095 | 0.854 | 1.08 | 1.10 | 6074 | 6122 | 6358 |
| P81041 | 0.854 | 1.07 | 1.09 | 6075 | 6088 | 6251 |
| B82097 | 0.854 | 1.07 | 1.05 | 6076 | 6108 | 5937 |
| G83668 | 0.854 | 1.00 | 1.00 | 6077 | 5417 | 5405 |
| K81010 | 0.854 | 0.93 | 0.92 | 6078 | 4452 | 4300 |
| M81047 | 0.854 | 1.22 | 1.20 | 6079 | 6997 | 6921 |
| G85087 | 0.853 | 0.92 | 0.92 | 6080 | 4400 | 4419 |
| Y02594 | 0.853 | 1.11 | 1.06 | 6081 | 6419 | 6020 |
| F84016 | 0.853 | 1.03 | 1.03 | 6082 | 5706 | 5680 |
| F86712 | 0.853 | 0.94 | 0.96 | 6083 | 4683 | 4953 |
| A83046 | 0.853 | 1.19 | 1.20 | 6084 | 6889 | 6934 |
| G82780 | 0.852 | 0.98 | 0.98 | 6085 | 5172 | 5152 |
| E87735 | 0.852 | 1.16 | 1.12 | 6086 | 6730 | 6554 |
| C82649 | 0.852 | 1.07 | 1.03 | 6087 | 6030 | 5748 |
| J82161 | 0.852 | 1.11 | 1.09 | 6088 | 6421 | 6281 |
| H84048 | 0.852 | 0.89 | 0.88 | 6089 | 4011 | 3814 |
| B81097 | 0.852 | 1.26 | 1.30 | 6090 | 7101 | 7162 |
| G84022 | 0.852 | 0.99 | 0.99 | 6091 | 5324 | 5220 |
| J83063 | 0.852 | 0.85 | 0.86 | 6092 | 3356 | 3617 |
| B86655 | 0.852 | 1.06 | 1.08 | 6093 | 5972 | 6163 |

| | | | | | | |
|---|---|---|---|---|---|---|
| M85136 | 0.852 | 1.10 | 1.08 | 6094 | 6336 | 6217 |
| G82205 | 0.851 | 0.94 | 0.93 | 6095 | 4712 | 4575 |
| A89603 | 0.851 | 1.22 | 1.22 | 6096 | 6986 | 6977 |
| F85043 | 0.851 | 1.03 | 1.02 | 6097 | 5681 | 5612 |
| M83711 | 0.851 | 1.19 | 1.23 | 6098 | 6891 | 7018 |
| G82161 | 0.851 | 1.08 | 1.10 | 6099 | 6154 | 6393 |
| B81030 | 0.851 | 1.17 | 1.21 | 6100 | 6823 | 6957 |
| E85008 | 0.851 | 0.99 | 1.01 | 6101 | 5260 | 5457 |
| M85069 | 0.851 | 1.11 | 1.10 | 6102 | 6448 | 6382 |
| M84629 | 0.851 | 1.03 | 1.03 | 6103 | 5697 | 5738 |
| C82657 | 0.851 | 1.15 | 1.15 | 6104 | 6703 | 6690 |
| M92016 | 0.850 | 1.07 | 1.08 | 6105 | 6074 | 6199 |
| L83023 | 0.850 | 1.22 | 1.20 | 6106 | 6990 | 6954 |
| J81613 | 0.850 | 1.10 | 1.10 | 6107 | 6355 | 6337 |
| M82002 | 0.850 | 1.00 | 1.00 | 6108 | 5371 | 5442 |
| C86026 | 0.850 | 1.25 | 1.27 | 6109 | 7088 | 7128 |
| P85022 | 0.849 | 1.09 | 1.11 | 6110 | 6328 | 6495 |
| L81084 | 0.849 | 1.08 | 1.08 | 6111 | 6168 | 6204 |
| P81084 | 0.849 | 1.14 | 1.16 | 6112 | 6663 | 6755 |
| G85133 | 0.849 | 0.98 | 0.99 | 6113 | 5183 | 5280 |
| G85705 | 0.849 | 0.87 | 0.87 | 6114 | 3700 | 3739 |
| L83024 | 0.849 | 1.09 | 1.06 | 6115 | 6279 | 5987 |
| H84053 | 0.849 | 1.03 | 1.02 | 6116 | 5769 | 5575 |
| Y02325 | 0.849 | 1.02 | 1.04 | 6117 | 5657 | 5801 |
| C84122 | 0.849 | 1.06 | 1.08 | 6118 | 5989 | 6223 |
| E85051 | 0.849 | 1.00 | 1.00 | 6119 | 5395 | 5421 |
| C84115 | 0.849 | 0.99 | 0.97 | 6120 | 5274 | 5077 |
| H81078 | 0.849 | 0.99 | 0.98 | 6121 | 5348 | 5149 |
| H82028 | 0.849 | 1.01 | 0.98 | 6122 | 5519 | 5242 |
| P92035 | 0.848 | 1.15 | 1.15 | 6123 | 6695 | 6736 |
| A82074 | 0.848 | 1.20 | 1.17 | 6124 | 6939 | 6803 |
| E81043 | 0.848 | 0.97 | 0.97 | 6125 | 5106 | 5030 |
| L84040 | 0.848 | 1.06 | 1.07 | 6126 | 6045 | 6157 |
| C84042 | 0.848 | 1.21 | 1.20 | 6127 | 6987 | 6955 |
| P84038 | 0.848 | 1.07 | 1.09 | 6128 | 6106 | 6340 |
| Y03049 | 0.848 | 0.89 | 0.91 | 6129 | 4014 | 4264 |
| P89005 | 0.848 | 1.13 | 1.15 | 6130 | 6589 | 6732 |
| N82089 | 0.848 | 1.08 | 1.10 | 6131 | 6236 | 6377 |
| F81756 | 0.847 | 1.23 | 1.22 | 6132 | 7043 | 7015 |
| Y00412 | 0.847 | 1.24 | 1.23 | 6133 | 7057 | 7031 |
| F86616 | 0.847 | 1.05 | 1.07 | 6134 | 5913 | 6128 |
| G81020 | 0.847 | 0.88 | 0.90 | 6135 | 3873 | 4174 |
| F84676 | 0.847 | 0.98 | 0.98 | 6136 | 5264 | 5255 |
| J83050 | 0.846 | 1.03 | 1.02 | 6137 | 5771 | 5694 |
| N85007 | 0.846 | 1.12 | 1.12 | 6138 | 6555 | 6549 |
| L85018 | 0.846 | 1.26 | 1.25 | 6139 | 7108 | 7077 |
| M82619 | 0.845 | 0.99 | 1.00 | 6140 | 5293 | 5483 |
| C84008 | 0.845 | 1.10 | 1.09 | 6141 | 6411 | 6375 |
| P84010 | 0.845 | 1.06 | 1.07 | 6142 | 6048 | 6195 |
| M81078 | 0.845 | 1.01 | 0.99 | 6143 | 5554 | 5285 |

| | | | | | | |
|---|---|---|---|---|---|---|
| G82741 | 0.845 | 0.98 | 1.01 | 6144 | 5245 | 5547 |
| B83031 | 0.844 | 1.09 | 1.10 | 6145 | 6361 | 6452 |
| G82775 | 0.844 | 1.02 | 1.04 | 6146 | 5624 | 5915 |
| P83621 | 0.844 | 1.10 | 1.11 | 6147 | 6468 | 6534 |
| E85726 | 0.844 | 0.96 | 0.97 | 6148 | 5063 | 5132 |
| B82014 | 0.844 | 1.03 | 1.03 | 6149 | 5805 | 5808 |
| F86042 | 0.844 | 1.05 | 1.03 | 6150 | 5939 | 5786 |
| E85099 | 0.844 | 0.92 | 0.92 | 6151 | 4445 | 4512 |
| Y02423 | 0.843 | 0.90 | 0.89 | 6152 | 4250 | 4042 |
| L84005 | 0.843 | 1.05 | 1.05 | 6153 | 5922 | 5959 |
| F83002 | 0.843 | 0.98 | 0.99 | 6154 | 5283 | 5378 |
| P84009 | 0.843 | 1.06 | 1.08 | 6155 | 6052 | 6267 |
| G84008 | 0.843 | 0.88 | 0.88 | 6156 | 3958 | 3975 |
| F85007 | 0.843 | 0.98 | 0.99 | 6157 | 5273 | 5348 |
| P81146 | 0.842 | 1.09 | 1.14 | 6158 | 6366 | 6686 |
| M86009 | 0.842 | 0.88 | 0.88 | 6159 | 3974 | 3951 |
| B81058 | 0.842 | 1.23 | 1.27 | 6160 | 7053 | 7131 |
| N81101 | 0.842 | 1.01 | 1.00 | 6161 | 5597 | 5456 |
| F82686 | 0.842 | 0.90 | 0.91 | 6162 | 4202 | 4409 |
| G82104 | 0.841 | 0.99 | 1.00 | 6163 | 5450 | 5443 |
| G84024 | 0.841 | 0.98 | 0.98 | 6164 | 5333 | 5276 |
| H82089 | 0.841 | 1.00 | 0.97 | 6165 | 5477 | 5188 |
| E83657 | 0.841 | 0.94 | 0.92 | 6166 | 4813 | 4583 |
| L83046 | 0.841 | 1.08 | 1.07 | 6167 | 6325 | 6241 |
| Y03112 | 0.841 | 1.16 | 1.17 | 6168 | 6781 | 6849 |
| L81061 | 0.841 | 1.01 | 1.02 | 6169 | 5630 | 5682 |
| N82094 | 0.841 | 1.22 | 1.23 | 6170 | 7040 | 7056 |
| G85019 | 0.841 | 0.97 | 0.97 | 6171 | 5216 | 5216 |
| A86007 | 0.841 | 0.84 | 0.86 | 6172 | 3450 | 3747 |
| D82018 | 0.840 | 1.04 | 1.04 | 6173 | 5943 | 5887 |
| M82044 | 0.840 | 1.12 | 1.12 | 6174 | 6610 | 6596 |
| L81632 | 0.840 | 0.91 | 0.91 | 6175 | 4447 | 4413 |
| M83130 | 0.840 | 1.24 | 1.27 | 6176 | 7071 | 7146 |
| C81648 | 0.840 | 0.98 | 0.99 | 6177 | 5357 | 5377 |
| F86004 | 0.840 | 0.97 | 0.98 | 6178 | 5126 | 5335 |
| E81013 | 0.840 | 0.96 | 0.97 | 6179 | 5090 | 5119 |
| F81114 | 0.840 | 1.11 | 1.11 | 6180 | 6566 | 6521 |
| F83648 | 0.840 | 1.06 | 1.06 | 6181 | 6078 | 6152 |
| F85645 | 0.840 | 0.97 | 0.97 | 6182 | 5131 | 5236 |
| E87024 | 0.839 | 1.10 | 1.08 | 6183 | 6486 | 6343 |
| G84033 | 0.839 | 0.98 | 0.97 | 6184 | 5251 | 5225 |
| C82038 | 0.839 | 1.09 | 1.08 | 6185 | 6393 | 6272 |
| C81094 | 0.839 | 1.07 | 1.07 | 6186 | 6206 | 6252 |
| E81633 | 0.838 | 1.05 | 1.06 | 6187 | 5984 | 6105 |
| E85116 | 0.838 | 1.07 | 1.07 | 6188 | 6174 | 6196 |
| F83023 | 0.838 | 0.97 | 0.96 | 6189 | 5159 | 5031 |
| F83033 | 0.838 | 1.09 | 1.10 | 6190 | 6409 | 6500 |
| M86039 | 0.838 | 1.05 | 1.05 | 6191 | 6023 | 6016 |
| M82034 | 0.838 | 1.06 | 1.07 | 6192 | 6121 | 6185 |
| E85723 | 0.837 | 1.05 | 1.06 | 6193 | 6087 | 6142 |

| | | | | | | |
|---|---|---|---|---|---|---|
| D81066 | 0.837 | 0.95 | 0.92 | 6194 | 4941 | 4607 |
| H84018 | 0.837 | 0.97 | 0.95 | 6195 | 5201 | 4979 |
| M91619 | 0.837 | 1.08 | 1.10 | 6196 | 6278 | 6487 |
| H82621 | 0.837 | 1.08 | 1.07 | 6197 | 6349 | 6274 |
| E81626 | 0.837 | 0.95 | 0.94 | 6198 | 4939 | 4906 |
| C81047 | 0.837 | 1.19 | 1.18 | 6199 | 6936 | 6929 |
| M88619 | 0.837 | 1.23 | 1.24 | 6200 | 7075 | 7082 |
| Y00206 | 0.837 | 0.93 | 0.92 | 6201 | 4779 | 4618 |
| J82131 | 0.837 | 1.11 | 1.10 | 6202 | 6551 | 6482 |
| J82030 | 0.836 | 1.06 | 1.05 | 6203 | 6115 | 6057 |
| J82192 | 0.836 | 0.91 | 0.90 | 6204 | 4469 | 4376 |
| F84053 | 0.836 | 0.98 | 0.96 | 6205 | 5292 | 5129 |
| N81001 | 0.836 | 1.09 | 1.08 | 6206 | 6422 | 6300 |
| F85002 | 0.836 | 1.04 | 1.04 | 6207 | 5976 | 5955 |
| H85002 | 0.836 | 0.94 | 0.92 | 6208 | 4817 | 4560 |
| H82087 | 0.836 | 1.30 | 1.29 | 6209 | 7177 | 7174 |
| E85050 | 0.836 | 0.99 | 1.00 | 6210 | 5452 | 5503 |
| P91016 | 0.836 | 1.02 | 1.03 | 6211 | 5789 | 5881 |
| Y02571 | 0.836 | 0.93 | 0.93 | 6212 | 4795 | 4757 |
| P81071 | 0.836 | 1.16 | 1.14 | 6213 | 6829 | 6754 |
| C88062 | 0.836 | 0.86 | 0.89 | 6214 | 3839 | 4181 |
| L85043 | 0.835 | 1.05 | 1.03 | 6215 | 6060 | 5893 |
| P84650 | 0.835 | 1.06 | 1.08 | 6216 | 6139 | 6367 |
| H85061 | 0.835 | 0.85 | 0.83 | 6217 | 3587 | 3389 |
| J82017 | 0.835 | 1.07 | 1.05 | 6218 | 6241 | 6100 |
| H85114 | 0.835 | 0.80 | 0.79 | 6219 | 2945 | 2793 |
| G85011 | 0.835 | 0.85 | 0.87 | 6220 | 3703 | 3895 |
| E85635 | 0.835 | 0.88 | 0.88 | 6221 | 4027 | 4142 |
| E86610 | 0.835 | 0.98 | 0.98 | 6222 | 5373 | 5322 |
| G84040 | 0.834 | 0.99 | 0.99 | 6223 | 5481 | 5444 |
| C82005 | 0.834 | 1.15 | 1.15 | 6224 | 6811 | 6801 |
| G81094 | 0.834 | 0.89 | 0.91 | 6225 | 4231 | 4558 |
| N81013 | 0.834 | 1.04 | 1.02 | 6226 | 5926 | 5795 |
| G84018 | 0.834 | 0.94 | 0.94 | 6227 | 4937 | 4925 |
| D82006 | 0.834 | 1.10 | 1.08 | 6228 | 6497 | 6387 |
| C81068 | 0.833 | 1.12 | 1.11 | 6229 | 6604 | 6581 |
| F81197 | 0.833 | 1.01 | 1.01 | 6230 | 5702 | 5716 |
| J82089 | 0.833 | 1.05 | 1.04 | 6231 | 6027 | 5970 |
| G82042 | 0.833 | 1.04 | 1.03 | 6232 | 5968 | 5886 |
| M81043 | 0.833 | 1.16 | 1.15 | 6233 | 6847 | 6774 |
| H83037 | 0.833 | 0.90 | 0.90 | 6234 | 4378 | 4336 |
| L85053 | 0.833 | 1.14 | 1.12 | 6235 | 6769 | 6661 |
| J82218 | 0.832 | 0.92 | 0.91 | 6236 | 4573 | 4451 |
| F83666 | 0.832 | 0.98 | 0.99 | 6237 | 5393 | 5481 |
| F84717 | 0.832 | 0.90 | 0.88 | 6238 | 4304 | 4158 |
| H85037 | 0.832 | 0.97 | 0.96 | 6239 | 5322 | 5111 |
| F82007 | 0.832 | 1.02 | 1.02 | 6240 | 5793 | 5790 |
| L81125 | 0.831 | 0.94 | 0.95 | 6241 | 4968 | 5054 |
| E84012 | 0.831 | 0.92 | 0.91 | 6242 | 4620 | 4452 |
| M85759 | 0.831 | 1.23 | 1.22 | 6243 | 7085 | 7038 |

| | | | | | | |
|---|---|---|---|---|---|---|
| B83604 | 0.831 | 1.24 | 1.26 | 6244 | 7105 | 7133 |
| F83682 | 0.831 | 0.92 | 0.91 | 6245 | 4600 | 4519 |
| C87622 | 0.831 | 0.88 | 0.90 | 6246 | 4105 | 4362 |
| L82049 | 0.831 | 1.17 | 1.16 | 6247 | 6908 | 6865 |
| J82163 | 0.831 | 1.11 | 1.10 | 6248 | 6590 | 6511 |
| E82131 | 0.831 | 1.05 | 1.05 | 6249 | 6091 | 6059 |
| G85016 | 0.831 | 0.90 | 0.91 | 6250 | 4370 | 4545 |
| P85607 | 0.831 | 1.14 | 1.16 | 6251 | 6777 | 6841 |
| E87047 | 0.830 | 0.97 | 0.97 | 6252 | 5338 | 5299 |
| B81085 | 0.830 | 1.12 | 1.16 | 6253 | 6623 | 6882 |
| H82100 | 0.830 | 1.03 | 1.03 | 6254 | 5909 | 5926 |
| L83010 | 0.830 | 1.06 | 1.05 | 6255 | 6194 | 6123 |
| L82008 | 0.830 | 1.17 | 1.18 | 6256 | 6915 | 6939 |
| E81017 | 0.830 | 1.12 | 1.11 | 6257 | 6643 | 6609 |
| C87023 | 0.830 | 1.36 | 1.35 | 6258 | 7241 | 7235 |
| P81769 | 0.830 | 1.09 | 1.09 | 6259 | 6480 | 6474 |
| M81061 | 0.830 | 1.09 | 1.08 | 6260 | 6472 | 6363 |
| E86001 | 0.830 | 1.05 | 1.04 | 6261 | 6082 | 6035 |
| F83008 | 0.830 | 1.02 | 1.03 | 6262 | 5849 | 5951 |
| J81646 | 0.829 | 1.12 | 1.11 | 6263 | 6645 | 6615 |
| F81123 | 0.829 | 1.08 | 1.05 | 6264 | 6372 | 6147 |
| K81063 | 0.829 | 0.96 | 0.96 | 6265 | 5190 | 5203 |
| L85040 | 0.829 | 1.05 | 1.06 | 6266 | 6128 | 6184 |
| D81070 | 0.829 | 0.85 | 0.83 | 6267 | 3673 | 3376 |
| Y01066 | 0.829 | 0.99 | 1.00 | 6268 | 5536 | 5641 |
| K84032 | 0.828 | 0.99 | 0.99 | 6269 | 5559 | 5492 |
| F86705 | 0.828 | 0.95 | 0.97 | 6270 | 5129 | 5306 |
| H85045 | 0.828 | 0.92 | 0.90 | 6271 | 4695 | 4431 |
| A83635 | 0.828 | 1.15 | 1.16 | 6272 | 6835 | 6872 |
| F81070 | 0.828 | 1.15 | 1.14 | 6273 | 6824 | 6769 |
| C81071 | 0.828 | 1.10 | 1.10 | 6274 | 6571 | 6569 |
| F84621 | 0.828 | 1.03 | 1.04 | 6275 | 5889 | 6048 |
| E87649 | 0.828 | 0.98 | 0.98 | 6276 | 5473 | 5373 |
| E84693 | 0.828 | 0.96 | 0.95 | 6277 | 5256 | 5019 |
| L83102 | 0.827 | 1.12 | 1.10 | 6278 | 6676 | 6580 |
| C81040 | 0.827 | 1.00 | 1.01 | 6279 | 5684 | 5757 |
| G85001 | 0.827 | 1.00 | 1.00 | 6280 | 5593 | 5614 |
| J82201 | 0.827 | 0.99 | 0.99 | 6281 | 5571 | 5561 |
| H81103 | 0.827 | 0.98 | 0.95 | 6282 | 5419 | 5088 |
| J83633 | 0.827 | 1.08 | 1.09 | 6283 | 6442 | 6504 |
| H84060 | 0.827 | 0.89 | 0.87 | 6284 | 4273 | 4101 |
| E87006 | 0.827 | 0.96 | 0.96 | 6285 | 5270 | 5182 |
| B86675 | 0.827 | 1.21 | 1.21 | 6286 | 7037 | 7034 |
| C84650 | 0.826 | 1.13 | 1.15 | 6287 | 6738 | 6837 |
| E87706 | 0.826 | 1.03 | 1.02 | 6288 | 5982 | 5862 |
| B86029 | 0.826 | 1.08 | 1.10 | 6289 | 6379 | 6542 |
| Y02936 | 0.826 | 0.98 | 1.01 | 6290 | 5478 | 5730 |
| E85030 | 0.826 | 0.90 | 0.89 | 6291 | 4499 | 4380 |
| E87037 | 0.826 | 0.93 | 0.93 | 6292 | 4878 | 4835 |
| E81075 | 0.825 | 1.00 | 1.01 | 6293 | 5691 | 5809 |

| | | | | | | |
|---|---|---|---|---|---|---|
| G83002 | 0.825 | 1.05 | 1.04 | 6294 | 6176 | 6074 |
| F83615 | 0.825 | 0.86 | 0.86 | 6295 | 3859 | 3886 |
| P81112 | 0.825 | 1.22 | 1.22 | 6296 | 7060 | 7066 |
| G84621 | 0.825 | 1.00 | 0.99 | 6297 | 5619 | 5580 |
| P84635 | 0.825 | 1.06 | 1.08 | 6298 | 6215 | 6448 |
| H83042 | 0.824 | 1.06 | 1.05 | 6299 | 6220 | 6215 |
| H84039 | 0.824 | 0.89 | 0.88 | 6300 | 4337 | 4186 |
| G82635 | 0.823 | 1.13 | 1.15 | 6301 | 6740 | 6859 |
| A83055 | 0.823 | 1.07 | 1.08 | 6302 | 6300 | 6451 |
| G83012 | 0.823 | 0.99 | 0.98 | 6303 | 5549 | 5500 |
| D82008 | 0.823 | 1.07 | 1.09 | 6304 | 6390 | 6512 |
| M85716 | 0.823 | 1.02 | 1.01 | 6305 | 5859 | 5818 |
| E82006 | 0.823 | 1.06 | 1.06 | 6306 | 6275 | 6265 |
| H83020 | 0.823 | 1.08 | 1.07 | 6307 | 6404 | 6317 |
| G81021 | 0.823 | 0.98 | 0.97 | 6308 | 5532 | 5399 |
| E85129 | 0.823 | 1.03 | 1.04 | 6309 | 6022 | 6104 |
| K81002 | 0.822 | 0.96 | 0.96 | 6310 | 5263 | 5265 |
| F84012 | 0.822 | 1.04 | 1.04 | 6311 | 6119 | 6099 |
| J81029 | 0.822 | 1.08 | 1.06 | 6312 | 6441 | 6293 |
| M81093 | 0.822 | 1.16 | 1.14 | 6313 | 6888 | 6796 |
| M85082 | 0.821 | 1.18 | 1.17 | 6314 | 6948 | 6925 |
| M83121 | 0.821 | 1.05 | 1.01 | 6315 | 6165 | 5827 |
| P81045 | 0.821 | 0.99 | 1.01 | 6316 | 5629 | 5821 |
| N81075 | 0.820 | 1.01 | 1.01 | 6317 | 5824 | 5838 |
| E85059 | 0.820 | 0.99 | 0.98 | 6318 | 5607 | 5533 |
| D81616 | 0.820 | 1.03 | 1.04 | 6319 | 6006 | 6110 |
| G81056 | 0.820 | 0.98 | 1.00 | 6320 | 5555 | 5728 |
| P81668 | 0.820 | 1.34 | 1.33 | 6321 | 7235 | 7224 |
| Y00020 | 0.820 | 1.02 | 1.03 | 6322 | 5910 | 5992 |
| M82048 | 0.820 | 1.07 | 1.08 | 6323 | 6401 | 6447 |
| G81009 | 0.820 | 0.94 | 0.96 | 6324 | 5062 | 5284 |
| F84060 | 0.820 | 0.93 | 0.95 | 6325 | 4984 | 5167 |
| B82016 | 0.819 | 1.09 | 1.08 | 6326 | 6508 | 6494 |
| L83077 | 0.819 | 1.09 | 1.05 | 6327 | 6544 | 6247 |
| E83050 | 0.819 | 0.88 | 0.87 | 6328 | 4332 | 4138 |
| K84051 | 0.819 | 1.25 | 1.21 | 6329 | 7146 | 7047 |
| E84003 | 0.818 | 1.14 | 1.13 | 6330 | 6826 | 6753 |
| F84710 | 0.818 | 0.96 | 0.96 | 6331 | 5353 | 5345 |
| F81206 | 0.818 | 1.13 | 1.13 | 6332 | 6780 | 6765 |
| K81057 | 0.817 | 0.98 | 0.98 | 6333 | 5573 | 5581 |
| F84018 | 0.817 | 0.97 | 0.98 | 6334 | 5411 | 5566 |
| E85110 | 0.817 | 0.94 | 0.96 | 6335 | 5148 | 5329 |
| C81627 | 0.817 | 1.11 | 1.11 | 6336 | 6660 | 6654 |
| L82001 | 0.816 | 1.03 | 1.03 | 6337 | 6016 | 6093 |
| G83631 | 0.816 | 1.02 | 1.02 | 6338 | 5904 | 5938 |
| G85681 | 0.816 | 0.92 | 0.92 | 6339 | 4855 | 4910 |
| F86700 | 0.816 | 1.19 | 1.20 | 6340 | 7027 | 7052 |
| F85008 | 0.816 | 0.94 | 0.95 | 6341 | 5117 | 5191 |
| H81085 | 0.816 | 0.88 | 0.86 | 6342 | 4286 | 3996 |
| N83041 | 0.816 | 1.19 | 1.21 | 6343 | 7026 | 7060 |

| | | | | | | |
|---|---|---|---|---|---|---|
| J83056 | 0.815 | 0.99 | 0.98 | 6344 | 5646 | 5585 |
| F85053 | 0.815 | 0.94 | 0.94 | 6345 | 5103 | 5075 |
| G85644 | 0.815 | 0.94 | 0.94 | 6346 | 5093 | 5067 |
| J83020 | 0.815 | 1.05 | 1.04 | 6347 | 6197 | 6132 |
| M88019 | 0.815 | 0.99 | 1.01 | 6348 | 5689 | 5874 |
| F84033 | 0.815 | 0.94 | 0.96 | 6349 | 5154 | 5326 |
| H85049 | 0.814 | 0.82 | 0.81 | 6350 | 3528 | 3354 |
| G85708 | 0.814 | 0.88 | 0.89 | 6351 | 4369 | 4521 |
| M83733 | 0.814 | 1.08 | 1.07 | 6352 | 6485 | 6422 |
| E85004 | 0.814 | 1.02 | 1.01 | 6353 | 5965 | 5859 |
| C82634 | 0.814 | 1.03 | 1.03 | 6354 | 6070 | 6095 |
| E84084 | 0.814 | 1.16 | 1.15 | 6355 | 6930 | 6868 |
| F83671 | 0.813 | 1.01 | 1.02 | 6356 | 5841 | 5961 |
| G84604 | 0.813 | 0.98 | 0.98 | 6357 | 5620 | 5582 |
| L81047 | 0.813 | 1.09 | 1.08 | 6358 | 6587 | 6527 |
| F83039 | 0.813 | 1.03 | 1.04 | 6359 | 6099 | 6219 |
| L81057 | 0.813 | 1.06 | 1.07 | 6360 | 6381 | 6426 |
| Y01050 | 0.813 | 1.15 | 1.15 | 6361 | 6877 | 6881 |
| C81064 | 0.813 | 1.07 | 1.07 | 6362 | 6418 | 6449 |
| L81045 | 0.813 | 1.07 | 1.06 | 6363 | 6445 | 6362 |
| F82012 | 0.812 | 1.09 | 1.09 | 6364 | 6573 | 6582 |
| F84093 | 0.812 | 0.91 | 0.90 | 6365 | 4793 | 4655 |
| K82064 | 0.812 | 0.90 | 0.90 | 6366 | 4703 | 4682 |
| B82071 | 0.812 | 1.02 | 1.01 | 6367 | 5942 | 5947 |
| C81072 | 0.811 | 1.05 | 1.05 | 6368 | 6288 | 6328 |
| F84044 | 0.811 | 1.00 | 1.00 | 6369 | 5801 | 5803 |
| K84019 | 0.811 | 1.03 | 1.00 | 6370 | 6096 | 5805 |
| C81019 | 0.811 | 1.10 | 1.09 | 6371 | 6624 | 6604 |
| D83615 | 0.811 | 1.18 | 1.18 | 6372 | 7013 | 6997 |
| H83622 | 0.810 | 1.00 | 0.99 | 6373 | 5775 | 5714 |
| F84685 | 0.810 | 0.94 | 0.96 | 6374 | 5194 | 5368 |
| H83623 | 0.810 | 0.91 | 0.91 | 6375 | 4797 | 4756 |
| H82002 | 0.810 | 1.05 | 1.07 | 6376 | 6268 | 6434 |
| Y00522 | 0.809 | 0.94 | 0.94 | 6377 | 5229 | 5214 |
| G82219 | 0.809 | 1.21 | 1.23 | 6378 | 7093 | 7117 |
| M86622 | 0.809 | 0.96 | 0.96 | 6379 | 5476 | 5465 |
| F81173 | 0.809 | 1.14 | 1.13 | 6380 | 6857 | 6829 |
| F84054 | 0.808 | 1.08 | 1.07 | 6381 | 6524 | 6505 |
| L83042 | 0.808 | 1.05 | 1.02 | 6382 | 6270 | 5993 |
| E82113 | 0.808 | 0.96 | 0.95 | 6383 | 5464 | 5358 |
| D82012 | 0.808 | 1.12 | 1.14 | 6384 | 6792 | 6847 |
| H82022 | 0.808 | 1.01 | 0.99 | 6385 | 5890 | 5688 |
| F84724 | 0.808 | 0.92 | 0.92 | 6386 | 5012 | 4894 |
| A86020 | 0.808 | 0.91 | 0.93 | 6387 | 4803 | 5045 |
| M87028 | 0.808 | 1.15 | 1.14 | 6388 | 6890 | 6878 |
| F85065 | 0.807 | 1.04 | 1.05 | 6389 | 6213 | 6303 |
| C81048 | 0.807 | 1.05 | 1.05 | 6390 | 6318 | 6353 |
| B83641 | 0.807 | 1.20 | 1.21 | 6391 | 7056 | 7086 |
| F81098 | 0.807 | 1.08 | 1.07 | 6392 | 6552 | 6508 |
| Y00057 | 0.807 | 0.88 | 0.88 | 6393 | 4464 | 4430 |

| | | | | | | |
|---|---|---|---|---|---|---|
| K82045 | 0.807 | 1.04 | 1.02 | 6394 | 6232 | 6062 |
| M88043 | 0.807 | 1.03 | 1.04 | 6395 | 6157 | 6273 |
| M87602 | 0.806 | 1.08 | 1.08 | 6396 | 6572 | 6531 |
| G81001 | 0.806 | 1.11 | 1.13 | 6397 | 6743 | 6838 |
| J81033 | 0.806 | 0.93 | 0.94 | 6398 | 5140 | 5161 |
| H85101 | 0.806 | 0.94 | 0.93 | 6399 | 5269 | 5063 |
| M85600 | 0.806 | 1.14 | 1.12 | 6400 | 6854 | 6775 |
| F81086 | 0.806 | 1.11 | 1.11 | 6401 | 6710 | 6746 |
| D83043 | 0.806 | 1.16 | 1.16 | 6402 | 6938 | 6956 |
| E86009 | 0.805 | 1.05 | 1.04 | 6403 | 6339 | 6234 |
| J81624 | 0.805 | 1.01 | 1.01 | 6404 | 5998 | 6023 |
| M82013 | 0.805 | 1.04 | 1.04 | 6405 | 6226 | 6289 |
| G82014 | 0.805 | 1.04 | 1.07 | 6406 | 6252 | 6488 |
| F83022 | 0.805 | 0.94 | 0.93 | 6407 | 5203 | 5123 |
| E85055 | 0.805 | 0.93 | 0.95 | 6408 | 5107 | 5318 |
| F81028 | 0.805 | 1.01 | 1.02 | 6409 | 5988 | 6092 |
| H81025 | 0.804 | 1.00 | 1.00 | 6410 | 5860 | 5918 |
| H84025 | 0.804 | 1.04 | 1.02 | 6411 | 6223 | 6053 |
| F85046 | 0.804 | 0.95 | 0.96 | 6412 | 5418 | 5499 |
| G85053 | 0.804 | 0.93 | 0.94 | 6413 | 5153 | 5238 |
| E87008 | 0.804 | 1.03 | 1.02 | 6414 | 6151 | 6061 |
| N81100 | 0.804 | 1.01 | 0.99 | 6415 | 5919 | 5806 |
| Y00454 | 0.803 | 0.94 | 0.95 | 6416 | 5294 | 5321 |
| G85118 | 0.802 | 1.05 | 1.06 | 6417 | 6357 | 6414 |
| L84012 | 0.802 | 1.01 | 1.00 | 6418 | 5947 | 5868 |
| L84004 | 0.802 | 1.13 | 1.09 | 6419 | 6860 | 6648 |
| B85006 | 0.802 | 1.04 | 1.04 | 6420 | 6238 | 6324 |
| J82028 | 0.802 | 0.98 | 0.99 | 6421 | 5668 | 5854 |
| B86031 | 0.801 | 1.07 | 1.09 | 6422 | 6538 | 6640 |
| K83621 | 0.800 | 0.90 | 0.91 | 6423 | 4794 | 4929 |
| B86005 | 0.800 | 1.16 | 1.17 | 6424 | 6967 | 6998 |
| M83013 | 0.800 | 1.11 | 1.07 | 6425 | 6737 | 6519 |
| G85114 | 0.800 | 1.03 | 1.05 | 6426 | 6218 | 6350 |
| L81118 | 0.799 | 1.01 | 1.01 | 6427 | 6042 | 6014 |
| K83009 | 0.799 | 0.98 | 0.99 | 6428 | 5685 | 5787 |
| C82042 | 0.798 | 1.09 | 1.09 | 6429 | 6667 | 6691 |
| F81094 | 0.798 | 1.09 | 1.10 | 6430 | 6642 | 6696 |
| P88006 | 0.798 | 1.09 | 1.09 | 6431 | 6684 | 6673 |
| P83008 | 0.798 | 1.13 | 1.14 | 6432 | 6881 | 6895 |
| P81722 | 0.798 | 1.39 | 1.43 | 6433 | 7271 | 7282 |
| M86610 | 0.798 | 0.98 | 0.98 | 6434 | 5773 | 5777 |
| C82050 | 0.798 | 1.12 | 1.11 | 6435 | 6832 | 6785 |
| E82031 | 0.797 | 0.95 | 0.94 | 6436 | 5410 | 5298 |
| M83090 | 0.797 | 1.33 | 1.35 | 6437 | 7239 | 7260 |
| F84080 | 0.797 | 1.03 | 1.04 | 6438 | 6221 | 6354 |
| F83064 | 0.797 | 1.02 | 1.02 | 6439 | 6111 | 6124 |
| E84624 | 0.796 | 1.12 | 1.10 | 6440 | 6815 | 6728 |
| E85617 | 0.796 | 0.91 | 0.92 | 6441 | 4967 | 5028 |
| M87618 | 0.796 | 0.99 | 0.99 | 6442 | 5818 | 5828 |
| B82057 | 0.796 | 1.06 | 1.06 | 6443 | 6519 | 6516 |

| | | | | | | |
|---|---|---|---|---|---|---|
| M92647 | 0.796 | 1.21 | 1.22 | 6444 | 7111 | 7127 |
| J82628 | 0.796 | 0.97 | 0.94 | 6445 | 5638 | 5309 |
| E85108 | 0.796 | 1.00 | 1.00 | 6446 | 5918 | 5976 |
| F86005 | 0.795 | 0.99 | 1.00 | 6447 | 5814 | 5965 |
| E84080 | 0.795 | 0.90 | 0.89 | 6448 | 4920 | 4768 |
| E82058 | 0.795 | 0.86 | 0.87 | 6449 | 4331 | 4467 |
| B86651 | 0.794 | 1.14 | 1.16 | 6450 | 6922 | 6982 |
| G83067 | 0.794 | 0.95 | 0.95 | 6451 | 5487 | 5447 |
| E87065 | 0.794 | 1.03 | 1.02 | 6452 | 6248 | 6172 |
| C84095 | 0.794 | 1.16 | 1.17 | 6453 | 6975 | 7027 |
| Y02713 | 0.794 | 1.14 | 1.16 | 6454 | 6935 | 7001 |
| E85600 | 0.794 | 1.03 | 1.02 | 6455 | 6267 | 6201 |
| E84070 | 0.794 | 0.97 | 0.95 | 6456 | 5717 | 5513 |
| J82639 | 0.793 | 1.05 | 1.03 | 6457 | 6406 | 6261 |
| K83035 | 0.793 | 1.05 | 1.06 | 6458 | 6433 | 6489 |
| F86074 | 0.793 | 0.93 | 0.95 | 6459 | 5332 | 5515 |
| C84069 | 0.792 | 1.06 | 1.10 | 6460 | 6546 | 6729 |
| E87011 | 0.792 | 1.07 | 1.06 | 6461 | 6575 | 6492 |
| E84601 | 0.792 | 0.93 | 0.92 | 6462 | 5305 | 5142 |
| L83052 | 0.792 | 1.06 | 1.08 | 6463 | 6542 | 6670 |
| C81059 | 0.792 | 1.25 | 1.24 | 6464 | 7168 | 7159 |
| H83609 | 0.792 | 1.07 | 1.06 | 6465 | 6569 | 6498 |
| B82091 | 0.792 | 1.04 | 1.04 | 6466 | 6397 | 6396 |
| L81010 | 0.792 | 1.12 | 1.10 | 6467 | 6833 | 6756 |
| K84036 | 0.792 | 1.01 | 0.98 | 6468 | 6065 | 5858 |
| C82651 | 0.792 | 1.26 | 1.26 | 6469 | 7187 | 7181 |
| E84059 | 0.791 | 1.01 | 1.01 | 6470 | 6086 | 6137 |
| H83007 | 0.791 | 0.97 | 0.96 | 6471 | 5692 | 5587 |
| G81007 | 0.791 | 0.99 | 0.98 | 6472 | 5869 | 5761 |
| K81080 | 0.791 | 0.94 | 0.94 | 6473 | 5404 | 5384 |
| L81600 | 0.790 | 0.98 | 0.97 | 6474 | 5837 | 5698 |
| H84006 | 0.790 | 0.93 | 0.91 | 6475 | 5291 | 5020 |
| G82733 | 0.790 | 0.94 | 0.94 | 6476 | 5394 | 5416 |
| M85014 | 0.790 | 1.15 | 1.14 | 6477 | 6962 | 6927 |
| A82026 | 0.789 | 1.12 | 1.08 | 6478 | 6848 | 6685 |
| C82671 | 0.789 | 1.07 | 1.08 | 6479 | 6621 | 6678 |
| N83015 | 0.789 | 1.20 | 1.20 | 6480 | 7106 | 7112 |
| E85019 | 0.789 | 0.97 | 0.97 | 6481 | 5716 | 5763 |
| E87746 | 0.788 | 0.92 | 0.92 | 6482 | 5217 | 5138 |
| M92042 | 0.788 | 1.10 | 1.11 | 6483 | 6753 | 6823 |
| N85047 | 0.788 | 1.13 | 1.12 | 6484 | 6911 | 6883 |
| F86625 | 0.788 | 1.01 | 1.03 | 6485 | 6143 | 6315 |
| E83053 | 0.787 | 1.03 | 1.01 | 6486 | 6281 | 6125 |
| J82130 | 0.787 | 0.92 | 0.90 | 6487 | 5258 | 5008 |
| G85096 | 0.787 | 0.95 | 0.96 | 6488 | 5501 | 5600 |
| P87659 | 0.786 | 1.08 | 1.11 | 6489 | 6675 | 6807 |
| E85122 | 0.786 | 0.87 | 0.88 | 6490 | 4618 | 4731 |
| E84076 | 0.786 | 1.03 | 1.01 | 6491 | 6302 | 6178 |
| E83008 | 0.786 | 0.94 | 0.93 | 6492 | 5495 | 5316 |
| L81033 | 0.786 | 1.05 | 1.06 | 6493 | 6516 | 6548 |

| | | | | | | |
|---|---|---|---|---|---|---|
| J82006 | 0.786 | 1.11 | 1.14 | 6494 | 6809 | 6943 |
| P91633 | 0.786 | 1.08 | 1.08 | 6495 | 6654 | 6703 |
| F84021 | 0.786 | 0.92 | 0.94 | 6496 | 5275 | 5451 |
| P87651 | 0.786 | 0.78 | 0.81 | 6497 | 3242 | 3710 |
| L83066 | 0.786 | 1.03 | 1.00 | 6498 | 6292 | 6068 |
| F84119 | 0.785 | 0.95 | 0.97 | 6499 | 5596 | 5751 |
| G84630 | 0.785 | 1.12 | 1.11 | 6500 | 6870 | 6827 |
| G84628 | 0.785 | 1.01 | 1.01 | 6501 | 6181 | 6114 |
| L81656 | 0.785 | 0.90 | 0.91 | 6502 | 5009 | 5091 |
| N82082 | 0.785 | 1.21 | 1.22 | 6503 | 7133 | 7142 |
| E87003 | 0.785 | 0.98 | 0.97 | 6504 | 5848 | 5768 |
| M81012 | 0.785 | 1.05 | 1.04 | 6505 | 6530 | 6465 |
| F83025 | 0.785 | 1.07 | 1.06 | 6506 | 6600 | 6555 |
| E85109 | 0.785 | 0.91 | 0.92 | 6507 | 5122 | 5181 |
| P81758 | 0.785 | 1.14 | 1.16 | 6508 | 6973 | 7012 |
| K81050 | 0.784 | 0.95 | 0.96 | 6509 | 5622 | 5657 |
| Y03402 | 0.783 | 0.99 | 0.99 | 6510 | 5969 | 5957 |
| E84663 | 0.783 | 1.02 | 1.01 | 6511 | 6277 | 6181 |
| E85636 | 0.783 | 0.92 | 0.95 | 6512 | 5323 | 5545 |
| E81611 | 0.783 | 0.99 | 0.99 | 6513 | 6003 | 5972 |
| L85064 | 0.782 | 1.08 | 1.08 | 6514 | 6666 | 6700 |
| F81207 | 0.782 | 1.04 | 1.04 | 6515 | 6436 | 6481 |
| G85051 | 0.782 | 0.86 | 0.87 | 6516 | 4524 | 4643 |
| F81133 | 0.782 | 1.10 | 1.10 | 6517 | 6785 | 6810 |
| B86095 | 0.782 | 1.18 | 1.19 | 6518 | 7081 | 7084 |
| M86048 | 0.782 | 0.99 | 0.98 | 6519 | 6005 | 5939 |
| M81018 | 0.781 | 1.12 | 1.10 | 6520 | 6886 | 6826 |
| G83654 | 0.781 | 1.03 | 1.02 | 6521 | 6396 | 6318 |
| Y01132 | 0.781 | 0.84 | 0.83 | 6522 | 4303 | 4126 |
| P84034 | 0.781 | 0.93 | 0.96 | 6523 | 5420 | 5669 |
| H82063 | 0.780 | 1.10 | 1.09 | 6524 | 6783 | 6770 |
| K84050 | 0.780 | 1.01 | 0.99 | 6525 | 6167 | 5985 |
| M85671 | 0.780 | 1.15 | 1.13 | 6526 | 7009 | 6941 |
| M85025 | 0.780 | 0.90 | 0.90 | 6527 | 5051 | 4984 |
| C84028 | 0.780 | 1.02 | 1.00 | 6528 | 6244 | 6096 |
| F84105 | 0.780 | 0.96 | 0.97 | 6529 | 5667 | 5826 |
| E86022 | 0.780 | 0.99 | 0.98 | 6530 | 5977 | 5956 |
| L81012 | 0.780 | 0.90 | 0.91 | 6531 | 5058 | 5170 |
| G85048 | 0.779 | 0.95 | 0.96 | 6532 | 5614 | 5735 |
| E82060 | 0.779 | 0.91 | 0.90 | 6533 | 5175 | 5064 |
| E84626 | 0.779 | 1.07 | 1.06 | 6534 | 6671 | 6575 |
| B82088 | 0.779 | 1.18 | 1.19 | 6535 | 7080 | 7101 |
| E86041 | 0.779 | 1.03 | 1.03 | 6536 | 6412 | 6355 |
| L85029 | 0.778 | 1.09 | 1.08 | 6537 | 6793 | 6697 |
| H82005 | 0.778 | 1.00 | 1.00 | 6538 | 6144 | 6133 |
| P87648 | 0.777 | 0.80 | 0.83 | 6539 | 3712 | 4163 |
| E85016 | 0.777 | 0.96 | 0.98 | 6540 | 5756 | 5950 |
| L85037 | 0.777 | 1.10 | 1.07 | 6541 | 6837 | 6689 |
| L84077 | 0.777 | 1.10 | 1.10 | 6542 | 6801 | 6798 |
| H82044 | 0.777 | 1.00 | 1.00 | 6543 | 6092 | 6101 |

| | | | | | | |
|---|---|---|---|---|---|---|
| G85061 | 0.777 | 1.03 | 1.03 | 6544 | 6375 | 6439 |
| J82605 | 0.777 | 0.87 | 0.89 | 6545 | 4701 | 4923 |
| C81660 | 0.777 | 1.20 | 1.19 | 6546 | 7134 | 7110 |
| G83021 | 0.777 | 0.94 | 0.94 | 6547 | 5578 | 5548 |
| N81623 | 0.776 | 1.03 | 1.03 | 6548 | 6430 | 6378 |
| N82079 | 0.776 | 0.91 | 0.93 | 6549 | 5193 | 5454 |
| K81610 | 0.776 | 0.94 | 0.94 | 6550 | 5566 | 5549 |
| K81074 | 0.776 | 0.83 | 0.82 | 6551 | 4234 | 4123 |
| G85083 | 0.775 | 0.94 | 0.95 | 6552 | 5586 | 5692 |
| E84075 | 0.775 | 1.02 | 1.00 | 6553 | 6333 | 6121 |
| H84042 | 0.775 | 1.00 | 0.98 | 6554 | 6124 | 5975 |
| E84676 | 0.775 | 1.13 | 1.11 | 6555 | 6969 | 6869 |
| L81078 | 0.775 | 0.92 | 0.93 | 6556 | 5341 | 5471 |
| P81157 | 0.775 | 1.13 | 1.10 | 6557 | 6954 | 6850 |
| E85014 | 0.775 | 0.90 | 0.91 | 6558 | 5176 | 5247 |
| B82083 | 0.774 | 1.02 | 1.01 | 6559 | 6298 | 6256 |
| B86623 | 0.774 | 0.85 | 0.87 | 6560 | 4535 | 4778 |
| F83658 | 0.774 | 0.98 | 0.97 | 6561 | 5986 | 5901 |
| P81208 | 0.774 | 1.18 | 1.18 | 6562 | 7090 | 7103 |
| M82008 | 0.774 | 1.09 | 1.09 | 6563 | 6778 | 6808 |
| P84669 | 0.774 | 0.91 | 0.93 | 6564 | 5214 | 5439 |
| M89030 | 0.774 | 0.93 | 0.90 | 6565 | 5444 | 5146 |
| E82053 | 0.773 | 0.99 | 0.98 | 6566 | 6058 | 6010 |
| F86689 | 0.773 | 0.93 | 0.95 | 6567 | 5484 | 5659 |
| L84003 | 0.773 | 0.89 | 0.90 | 6568 | 5047 | 5184 |
| K83607 | 0.773 | 1.18 | 1.21 | 6569 | 7089 | 7147 |
| K81103 | 0.772 | 0.98 | 0.98 | 6570 | 5941 | 5999 |
| K84082 | 0.772 | 0.99 | 0.98 | 6571 | 6031 | 6013 |
| K84048 | 0.772 | 1.01 | 1.00 | 6572 | 6276 | 6186 |
| L81056 | 0.772 | 0.96 | 0.94 | 6573 | 5777 | 5626 |
| J82143 | 0.772 | 0.95 | 0.94 | 6574 | 5664 | 5623 |
| E85028 | 0.772 | 0.94 | 0.94 | 6575 | 5585 | 5640 |
| N82664 | 0.771 | 1.04 | 1.06 | 6576 | 6527 | 6632 |
| P89010 | 0.771 | 1.15 | 1.18 | 6577 | 7036 | 7085 |
| G81028 | 0.771 | 0.92 | 0.95 | 6578 | 5443 | 5654 |
| Y00999 | 0.771 | 0.91 | 0.91 | 6579 | 5231 | 5334 |
| F86058 | 0.771 | 0.92 | 0.93 | 6580 | 5363 | 5541 |
| P83025 | 0.770 | 0.96 | 0.97 | 6581 | 5827 | 5942 |
| M83608 | 0.770 | 1.06 | 1.02 | 6582 | 6636 | 6380 |
| P91029 | 0.770 | 1.15 | 1.15 | 6583 | 7024 | 7017 |
| F85049 | 0.769 | 0.99 | 0.99 | 6584 | 6083 | 6143 |
| C84090 | 0.769 | 0.98 | 1.01 | 6585 | 6007 | 6290 |
| F82619 | 0.769 | 0.95 | 0.95 | 6586 | 5723 | 5753 |
| K84079 | 0.769 | 0.90 | 0.88 | 6587 | 5232 | 4967 |
| B86654 | 0.769 | 1.00 | 1.02 | 6588 | 6202 | 6410 |
| M91020 | 0.768 | 1.08 | 1.11 | 6589 | 6779 | 6896 |
| F85011 | 0.768 | 1.23 | 1.22 | 6590 | 7176 | 7169 |
| L83648 | 0.768 | 0.91 | 0.93 | 6591 | 5330 | 5557 |
| G81634 | 0.768 | 1.08 | 1.05 | 6592 | 6762 | 6635 |
| E85032 | 0.768 | 0.93 | 0.95 | 6593 | 5577 | 5776 |

| | | | | | | |
|---|---|---|---|---|---|---|
| L85034 | 0.768 | 1.02 | 1.01 | 6594 | 6403 | 6288 |
| P88633 | 0.767 | 1.17 | 1.17 | 6595 | 7095 | 7089 |
| H81069 | 0.767 | 0.98 | 0.97 | 6596 | 6050 | 5941 |
| H81084 | 0.767 | 0.95 | 0.92 | 6597 | 5739 | 5459 |
| K84073 | 0.767 | 0.96 | 0.96 | 6598 | 5853 | 5861 |
| H85618 | 0.766 | 1.11 | 1.10 | 6599 | 6906 | 6866 |
| M82007 | 0.766 | 1.20 | 1.20 | 6600 | 7140 | 7139 |
| E87762 | 0.766 | 0.98 | 0.96 | 6601 | 6017 | 5911 |
| E84026 | 0.766 | 0.97 | 0.96 | 6602 | 5952 | 5840 |
| P92620 | 0.765 | 1.09 | 1.09 | 6603 | 6827 | 6855 |
| F82028 | 0.765 | 1.05 | 1.05 | 6604 | 6625 | 6605 |
| D81003 | 0.765 | 0.94 | 0.92 | 6605 | 5713 | 5412 |
| E85112 | 0.765 | 1.00 | 1.01 | 6606 | 6247 | 6313 |
| K81028 | 0.765 | 0.92 | 0.91 | 6607 | 5423 | 5297 |
| Y00312 | 0.765 | 1.03 | 1.03 | 6608 | 6481 | 6496 |
| H84057 | 0.765 | 0.93 | 0.92 | 6609 | 5598 | 5420 |
| H85111 | 0.764 | 0.93 | 0.91 | 6610 | 5589 | 5379 |
| P87649 | 0.764 | 1.00 | 1.03 | 6611 | 6272 | 6532 |
| F83015 | 0.764 | 1.00 | 1.01 | 6612 | 6207 | 6323 |
| Y00403 | 0.763 | 0.87 | 0.89 | 6613 | 4880 | 5055 |
| Y02520 | 0.763 | 0.93 | 0.95 | 6614 | 5590 | 5788 |
| P81213 | 0.763 | 0.91 | 0.91 | 6615 | 5334 | 5344 |
| J82216 | 0.763 | 1.00 | 0.99 | 6616 | 6245 | 6173 |
| E84002 | 0.762 | 0.98 | 0.97 | 6617 | 6103 | 5995 |
| F83060 | 0.761 | 0.99 | 1.00 | 6618 | 6146 | 6264 |
| F83681 | 0.761 | 1.04 | 1.05 | 6619 | 6595 | 6633 |
| G83024 | 0.761 | 0.98 | 0.97 | 6620 | 6057 | 6018 |
| H84059 | 0.761 | 0.91 | 0.90 | 6621 | 5439 | 5245 |
| F84041 | 0.761 | 0.96 | 0.97 | 6622 | 5905 | 6052 |
| H85680 | 0.761 | 0.89 | 0.87 | 6623 | 5155 | 4945 |
| F86082 | 0.761 | 1.15 | 1.13 | 6624 | 7050 | 6979 |
| L84039 | 0.761 | 1.07 | 1.07 | 6625 | 6756 | 6750 |
| G81042 | 0.761 | 0.96 | 0.98 | 6626 | 5876 | 6084 |
| F84008 | 0.761 | 0.95 | 0.97 | 6627 | 5839 | 6005 |
| K81068 | 0.760 | 0.94 | 0.93 | 6628 | 5729 | 5586 |
| K81640 | 0.760 | 0.85 | 0.86 | 6629 | 4656 | 4759 |
| G85706 | 0.760 | 0.90 | 0.91 | 6630 | 5307 | 5458 |
| C82084 | 0.759 | 1.05 | 1.06 | 6631 | 6681 | 6724 |
| F82005 | 0.759 | 1.05 | 1.05 | 6632 | 6640 | 6663 |
| K84046 | 0.758 | 1.02 | 0.99 | 6633 | 6456 | 6263 |
| E82014 | 0.758 | 0.91 | 0.91 | 6634 | 5458 | 5367 |
| E83021 | 0.758 | 0.98 | 0.97 | 6635 | 6161 | 5998 |
| M85669 | 0.758 | 1.03 | 1.02 | 6636 | 6521 | 6485 |
| J83032 | 0.757 | 1.04 | 1.03 | 6637 | 6594 | 6567 |
| H81041 | 0.757 | 0.98 | 0.99 | 6638 | 6138 | 6206 |
| C82620 | 0.757 | 1.03 | 1.04 | 6639 | 6535 | 6592 |
| M85041 | 0.757 | 0.83 | 0.83 | 6640 | 4436 | 4448 |
| E85123 | 0.757 | 0.85 | 0.86 | 6641 | 4717 | 4837 |
| Y01090 | 0.757 | 0.95 | 0.94 | 6642 | 5872 | 5771 |
| E87002 | 0.756 | 0.94 | 0.93 | 6643 | 5707 | 5622 |

| | | | | | | |
|---|---|---|---|---|---|---|
| C82070 | 0.756 | 1.20 | 1.20 | 6644 | 7155 | 7152 |
| J82183 | 0.756 | 1.01 | 1.02 | 6645 | 6425 | 6530 |
| J82075 | 0.756 | 1.08 | 1.07 | 6646 | 6851 | 6800 |
| H81130 | 0.755 | 0.94 | 0.93 | 6647 | 5780 | 5644 |
| E84067 | 0.755 | 0.95 | 0.94 | 6648 | 5870 | 5762 |
| M88629 | 0.755 | 1.03 | 1.05 | 6649 | 6582 | 6693 |
| G81095 | 0.755 | 1.10 | 1.14 | 6650 | 6934 | 7042 |
| E85683 | 0.755 | 0.89 | 0.89 | 6651 | 5253 | 5185 |
| G81018 | 0.755 | 0.93 | 0.95 | 6652 | 5652 | 5907 |
| P81757 | 0.754 | 0.96 | 0.99 | 6653 | 5944 | 6308 |
| H81643 | 0.754 | 0.94 | 0.95 | 6654 | 5804 | 5878 |
| E81054 | 0.753 | 1.05 | 1.06 | 6655 | 6674 | 6748 |
| G85136 | 0.753 | 0.85 | 0.86 | 6656 | 4737 | 4896 |
| G85002 | 0.753 | 0.83 | 0.84 | 6657 | 4487 | 4653 |
| F83004 | 0.753 | 1.03 | 1.04 | 6658 | 6556 | 6619 |
| Y00189 | 0.753 | 1.04 | 1.04 | 6659 | 6622 | 6660 |
| F81183 | 0.753 | 1.07 | 1.06 | 6660 | 6788 | 6742 |
| H83049 | 0.752 | 1.07 | 1.05 | 6661 | 6794 | 6701 |
| M85074 | 0.752 | 0.98 | 0.98 | 6662 | 6182 | 6154 |
| G85022 | 0.752 | 1.00 | 1.01 | 6663 | 6344 | 6446 |
| M87605 | 0.751 | 1.10 | 1.09 | 6664 | 6914 | 6898 |
| H84619 | 0.751 | 0.86 | 0.85 | 6665 | 4917 | 4788 |
| M86613 | 0.751 | 1.02 | 1.00 | 6666 | 6523 | 6408 |
| L81075 | 0.751 | 0.87 | 0.89 | 6667 | 5097 | 5230 |
| E83649 | 0.751 | 0.87 | 0.86 | 6668 | 5079 | 4920 |
| J82019 | 0.751 | 0.99 | 0.98 | 6669 | 6249 | 6230 |
| H85659 | 0.751 | 0.84 | 0.83 | 6670 | 4725 | 4531 |
| F83652 | 0.750 | 0.89 | 0.91 | 6671 | 5352 | 5480 |
| G85673 | 0.750 | 0.91 | 0.92 | 6672 | 5524 | 5607 |
| K84617 | 0.750 | 0.92 | 0.91 | 6673 | 5570 | 5510 |
| Y03664 | 0.749 | 0.84 | 0.83 | 6674 | 4665 | 4487 |
| K84025 | 0.749 | 0.99 | 0.96 | 6675 | 6246 | 6029 |
| A88015 | 0.749 | 1.35 | 1.33 | 6676 | 7273 | 7268 |
| N81607 | 0.749 | 1.30 | 1.26 | 6677 | 7255 | 7223 |
| P87028 | 0.748 | 1.13 | 1.16 | 6678 | 7035 | 7098 |
| Y02121 | 0.747 | 1.26 | 1.22 | 6679 | 7223 | 7195 |
| Y00560 | 0.747 | 1.02 | 1.01 | 6680 | 6522 | 6490 |
| J81078 | 0.747 | 1.02 | 1.00 | 6681 | 6537 | 6423 |
| Y02928 | 0.747 | 0.88 | 0.87 | 6682 | 5265 | 5128 |
| F83012 | 0.746 | 0.98 | 0.99 | 6683 | 6211 | 6320 |
| C81108 | 0.746 | 1.05 | 1.03 | 6684 | 6757 | 6628 |
| F84003 | 0.745 | 1.02 | 1.03 | 6685 | 6578 | 6645 |
| F83020 | 0.745 | 0.93 | 0.93 | 6686 | 5781 | 5741 |
| E84681 | 0.745 | 1.04 | 1.02 | 6687 | 6699 | 6578 |
| E83039 | 0.745 | 0.80 | 0.79 | 6688 | 4196 | 4054 |
| M89608 | 0.744 | 0.99 | 0.96 | 6689 | 6327 | 6065 |
| M92028 | 0.744 | 0.97 | 0.98 | 6690 | 6137 | 6258 |
| E83045 | 0.744 | 1.05 | 1.02 | 6691 | 6718 | 6594 |
| E87034 | 0.744 | 0.93 | 0.92 | 6692 | 5753 | 5693 |
| E82094 | 0.744 | 0.92 | 0.92 | 6693 | 5711 | 5706 |

| | | | | | | |
|---|---|---|---|---|---|---|
| E85713 | 0.743 | 1.11 | 1.11 | 6694 | 7006 | 6974 |
| E84638 | 0.743 | 0.99 | 0.97 | 6695 | 6306 | 6207 |
| M88626 | 0.743 | 1.28 | 1.29 | 6696 | 7244 | 7252 |
| L84014 | 0.743 | 1.09 | 1.09 | 6697 | 6937 | 6919 |
| F84077 | 0.743 | 0.94 | 0.93 | 6698 | 5896 | 5767 |
| H83624 | 0.742 | 0.92 | 0.92 | 6699 | 5743 | 5660 |
| P84005 | 0.742 | 0.84 | 0.86 | 6700 | 4733 | 5001 |
| L83134 | 0.742 | 1.16 | 1.17 | 6701 | 7109 | 7141 |
| E85020 | 0.742 | 0.89 | 0.91 | 6702 | 5424 | 5621 |
| F84062 | 0.742 | 1.12 | 1.11 | 6703 | 7025 | 6988 |
| M91024 | 0.742 | 1.27 | 1.28 | 6704 | 7240 | 7246 |
| Y00726 | 0.742 | 1.24 | 1.25 | 6705 | 7213 | 7218 |
| H81073 | 0.742 | 0.92 | 0.91 | 6706 | 5649 | 5565 |
| E84039 | 0.742 | 1.07 | 1.04 | 6707 | 6840 | 6730 |
| F85066 | 0.741 | 0.93 | 0.94 | 6708 | 5762 | 5873 |
| F83006 | 0.741 | 1.03 | 1.03 | 6709 | 6668 | 6626 |
| N83025 | 0.741 | 1.42 | 1.42 | 6710 | 7298 | 7297 |
| C84624 | 0.740 | 1.07 | 1.07 | 6711 | 6873 | 6856 |
| H85001 | 0.740 | 0.80 | 0.79 | 6712 | 4252 | 4099 |
| E85685 | 0.740 | 0.88 | 0.90 | 6713 | 5315 | 5531 |
| C86022 | 0.740 | 1.17 | 1.19 | 6714 | 7136 | 7164 |
| F84118 | 0.739 | 0.91 | 0.91 | 6715 | 5604 | 5632 |
| H81082 | 0.739 | 1.03 | 1.01 | 6716 | 6639 | 6544 |
| M89016 | 0.739 | 1.07 | 1.04 | 6717 | 6865 | 6719 |
| E84030 | 0.739 | 1.07 | 1.05 | 6718 | 6841 | 6759 |
| J82171 | 0.739 | 1.03 | 1.05 | 6719 | 6686 | 6758 |
| Y01812 | 0.739 | 1.11 | 1.08 | 6720 | 7014 | 6899 |
| P89018 | 0.738 | 1.31 | 1.32 | 6721 | 7260 | 7269 |
| J83033 | 0.738 | 1.15 | 1.15 | 6722 | 7099 | 7107 |
| G81047 | 0.738 | 0.89 | 0.92 | 6723 | 5482 | 5711 |
| H82039 | 0.738 | 1.15 | 1.14 | 6724 | 7107 | 7078 |
| M85145 | 0.737 | 1.11 | 1.10 | 6725 | 7017 | 6984 |
| E83049 | 0.737 | 0.96 | 0.94 | 6726 | 6140 | 5973 |
| M89028 | 0.737 | 0.97 | 0.95 | 6727 | 6191 | 6006 |
| G85109 | 0.737 | 0.90 | 0.91 | 6728 | 5543 | 5667 |
| F84079 | 0.737 | 0.92 | 0.93 | 6729 | 5770 | 5797 |
| N81031 | 0.737 | 1.09 | 1.06 | 6730 | 6928 | 6848 |
| M85110 | 0.737 | 1.13 | 1.13 | 6731 | 7077 | 7050 |
| P85620 | 0.736 | 1.10 | 1.11 | 6732 | 6984 | 6992 |
| J82076 | 0.736 | 1.11 | 1.12 | 6733 | 7018 | 7028 |
| Y00471 | 0.736 | 1.04 | 1.03 | 6734 | 6700 | 6658 |
| L83139 | 0.736 | 1.17 | 1.16 | 6735 | 7148 | 7122 |
| K82621 | 0.735 | 0.96 | 0.94 | 6736 | 6178 | 6003 |
| J82663 | 0.734 | 0.84 | 0.86 | 6737 | 4898 | 5096 |
| L81070 | 0.733 | 0.99 | 0.99 | 6738 | 6459 | 6438 |
| F83680 | 0.733 | 0.99 | 0.99 | 6739 | 6399 | 6476 |
| F83045 | 0.733 | 0.92 | 0.93 | 6740 | 5778 | 5924 |
| F83624 | 0.733 | 0.84 | 0.86 | 6741 | 4966 | 5098 |
| G85030 | 0.733 | 0.91 | 0.92 | 6742 | 5727 | 5756 |
| F84052 | 0.733 | 1.03 | 1.01 | 6743 | 6702 | 6607 |

| | | | | | | |
|---|---|---|---|---|---|---|
| H81030 | 0.732 | 1.02 | 1.01 | 6744 | 6641 | 6563 |
| F84719 | 0.732 | 0.97 | 0.98 | 6745 | 6266 | 6392 |
| E87754 | 0.732 | 0.85 | 0.84 | 6746 | 5004 | 4954 |
| F84682 | 0.731 | 0.97 | 0.97 | 6747 | 6265 | 6269 |
| G85132 | 0.731 | 0.93 | 0.94 | 6748 | 5923 | 5980 |
| P84639 | 0.731 | 1.10 | 1.13 | 6749 | 7012 | 7065 |
| L81108 | 0.731 | 0.93 | 0.92 | 6750 | 5863 | 5823 |
| Y00058 | 0.731 | 0.94 | 0.95 | 6751 | 6025 | 6131 |
| G85716 | 0.730 | 1.03 | 1.04 | 6752 | 6729 | 6757 |
| E82023 | 0.730 | 0.98 | 0.98 | 6753 | 6367 | 6400 |
| H83611 | 0.730 | 1.00 | 0.99 | 6754 | 6514 | 6478 |
| G85695 | 0.729 | 0.88 | 0.89 | 6755 | 5379 | 5488 |
| D81620 | 0.729 | 0.96 | 0.97 | 6756 | 6188 | 6268 |
| M85701 | 0.728 | 1.12 | 1.11 | 6757 | 7067 | 7044 |
| G85112 | 0.728 | 0.88 | 0.88 | 6758 | 5385 | 5441 |
| Y03063 | 0.728 | 0.87 | 0.89 | 6759 | 5378 | 5486 |
| G82665 | 0.728 | 1.34 | 1.36 | 6760 | 7277 | 7281 |
| B86043 | 0.727 | 1.15 | 1.16 | 6761 | 7126 | 7140 |
| J82194 | 0.727 | 1.08 | 1.09 | 6762 | 6945 | 6970 |
| L83082 | 0.727 | 1.05 | 1.03 | 6763 | 6821 | 6737 |
| F85069 | 0.727 | 0.86 | 0.88 | 6764 | 5261 | 5376 |
| F83660 | 0.727 | 0.95 | 0.96 | 6765 | 6126 | 6255 |
| D81056 | 0.726 | 0.86 | 0.83 | 6766 | 5173 | 4891 |
| D82069 | 0.726 | 1.01 | 1.03 | 6767 | 6626 | 6745 |
| F84096 | 0.726 | 0.96 | 0.98 | 6768 | 6255 | 6373 |
| G81090 | 0.726 | 0.99 | 1.01 | 6769 | 6500 | 6601 |
| J83636 | 0.726 | 0.99 | 0.99 | 6770 | 6533 | 6493 |
| E84074 | 0.726 | 1.01 | 0.99 | 6771 | 6608 | 6515 |
| P89613 | 0.725 | 1.09 | 1.11 | 6772 | 6985 | 7051 |
| D81002 | 0.725 | 0.89 | 0.87 | 6773 | 5579 | 5331 |
| G85073 | 0.725 | 0.94 | 0.95 | 6774 | 6034 | 6149 |
| C85028 | 0.725 | 1.15 | 1.16 | 6775 | 7132 | 7148 |
| E83026 | 0.725 | 0.98 | 0.97 | 6776 | 6438 | 6310 |
| G85698 | 0.724 | 0.94 | 0.95 | 6777 | 6062 | 6174 |
| B82077 | 0.724 | 1.13 | 1.15 | 6778 | 7087 | 7135 |
| P84039 | 0.723 | 1.05 | 1.07 | 6779 | 6842 | 6933 |
| J82102 | 0.723 | 1.08 | 1.09 | 6780 | 6952 | 7000 |
| L83127 | 0.722 | 1.09 | 1.10 | 6781 | 6993 | 7019 |
| H83011 | 0.722 | 0.94 | 0.93 | 6782 | 6054 | 6025 |
| K83050 | 0.722 | 1.00 | 1.01 | 6783 | 6596 | 6672 |
| E82068 | 0.722 | 0.95 | 0.94 | 6784 | 6163 | 6073 |
| C84044 | 0.722 | 1.00 | 1.02 | 6785 | 6565 | 6716 |
| E83046 | 0.722 | 1.15 | 1.11 | 6786 | 7137 | 7057 |
| B81100 | 0.721 | 1.11 | 1.11 | 6787 | 7063 | 7059 |
| G81054 | 0.721 | 1.00 | 1.01 | 6788 | 6567 | 6677 |
| C82032 | 0.721 | 1.11 | 1.11 | 6789 | 7046 | 7049 |
| E85658 | 0.721 | 0.93 | 0.92 | 6790 | 5975 | 5960 |
| Y02636 | 0.721 | 0.88 | 0.90 | 6791 | 5502 | 5679 |
| G82820 | 0.720 | 1.23 | 1.25 | 6792 | 7224 | 7244 |
| K81651 | 0.720 | 0.94 | 0.94 | 6793 | 6061 | 6127 |

| | | | | | | |
|---|---|---|---|---|---|---|
| E84028 | 0.719 | 1.03 | 1.01 | 6794 | 6739 | 6666 |
| L81007 | 0.719 | 0.92 | 0.93 | 6795 | 5884 | 6011 |
| L84030 | 0.719 | 0.99 | 0.99 | 6796 | 6504 | 6559 |
| B86074 | 0.719 | 1.01 | 1.04 | 6797 | 6683 | 6781 |
| Y00561 | 0.719 | 0.94 | 0.93 | 6798 | 6081 | 6028 |
| Y00328 | 0.719 | 1.01 | 1.01 | 6799 | 6662 | 6649 |
| P88043 | 0.719 | 1.42 | 1.39 | 6800 | 7301 | 7295 |
| H84027 | 0.719 | 0.86 | 0.85 | 6801 | 5351 | 5198 |
| J82098 | 0.718 | 1.04 | 1.04 | 6802 | 6836 | 6818 |
| F86060 | 0.718 | 1.03 | 1.02 | 6803 | 6786 | 6711 |
| E87016 | 0.718 | 0.87 | 0.87 | 6804 | 5432 | 5390 |
| F85642 | 0.717 | 0.95 | 0.95 | 6805 | 6199 | 6191 |
| Y01206 | 0.717 | 0.86 | 0.85 | 6806 | 5355 | 5156 |
| E86626 | 0.717 | 0.99 | 0.98 | 6807 | 6570 | 6524 |
| D81615 | 0.717 | 0.96 | 0.97 | 6808 | 6284 | 6394 |
| B81617 | 0.717 | 1.06 | 1.08 | 6809 | 6892 | 6971 |
| F85019 | 0.717 | 0.99 | 1.00 | 6810 | 6557 | 6606 |
| F86087 | 0.717 | 0.99 | 0.98 | 6811 | 6581 | 6483 |
| L81091 | 0.716 | 0.87 | 0.88 | 6812 | 5415 | 5543 |
| H84012 | 0.716 | 0.87 | 0.85 | 6813 | 5447 | 5261 |
| E83007 | 0.716 | 1.06 | 1.04 | 6814 | 6921 | 6816 |
| F84747 | 0.716 | 0.81 | 0.81 | 6815 | 4708 | 4796 |
| F84051 | 0.716 | 1.03 | 1.03 | 6816 | 6771 | 6766 |
| L83049 | 0.716 | 1.08 | 1.07 | 6817 | 7000 | 6959 |
| M86004 | 0.715 | 0.84 | 0.84 | 6818 | 5101 | 5097 |
| Y00612 | 0.715 | 0.92 | 0.92 | 6819 | 5935 | 5974 |
| G81045 | 0.715 | 0.90 | 0.89 | 6820 | 5744 | 5672 |
| M85782 | 0.715 | 0.78 | 0.79 | 6821 | 4399 | 4414 |
| M88610 | 0.715 | 1.08 | 1.09 | 6822 | 6994 | 7026 |
| Y03035 | 0.714 | 0.92 | 0.93 | 6823 | 5960 | 6056 |
| F82670 | 0.714 | 0.96 | 0.96 | 6824 | 6323 | 6330 |
| E81615 | 0.714 | 1.04 | 1.03 | 6825 | 6818 | 6791 |
| P84616 | 0.714 | 0.99 | 1.01 | 6826 | 6579 | 6709 |
| F82040 | 0.714 | 0.97 | 0.98 | 6827 | 6453 | 6537 |
| L84049 | 0.714 | 1.02 | 1.03 | 6828 | 6742 | 6782 |
| K84012 | 0.713 | 0.96 | 0.94 | 6829 | 6370 | 6140 |
| H85100 | 0.713 | 0.84 | 0.82 | 6830 | 5112 | 4907 |
| F86073 | 0.713 | 0.93 | 0.94 | 6831 | 6043 | 6222 |
| L81130 | 0.712 | 1.04 | 1.03 | 6832 | 6838 | 6799 |
| Y02177 | 0.712 | 0.98 | 0.99 | 6833 | 6510 | 6587 |
| M85072 | 0.712 | 1.13 | 1.12 | 6834 | 7115 | 7091 |
| L84034 | 0.711 | 1.08 | 1.07 | 6835 | 6998 | 6968 |
| E87755 | 0.711 | 0.90 | 0.89 | 6836 | 5755 | 5731 |
| H85051 | 0.710 | 0.88 | 0.87 | 6837 | 5594 | 5489 |
| C88068 | 0.710 | 0.95 | 0.97 | 6838 | 6269 | 6468 |
| H82098 | 0.710 | 1.00 | 1.01 | 6839 | 6661 | 6739 |
| E85740 | 0.710 | 0.95 | 0.96 | 6840 | 6242 | 6349 |
| C81042 | 0.709 | 1.02 | 1.03 | 6841 | 6764 | 6795 |
| F81100 | 0.709 | 1.04 | 1.05 | 6842 | 6880 | 6892 |
| M85676 | 0.709 | 1.20 | 1.20 | 6843 | 7210 | 7205 |

| | | | | | | |
|---|---|---|---|---|---|---|
| F86062 | 0.709 | 1.01 | 1.03 | 6844 | 6719 | 6804 |
| M92612 | 0.708 | 1.23 | 1.24 | 6845 | 7229 | 7250 |
| G85085 | 0.708 | 0.91 | 0.92 | 6846 | 5974 | 6070 |
| G85642 | 0.708 | 0.89 | 0.89 | 6847 | 5754 | 5774 |
| H83051 | 0.707 | 0.97 | 0.96 | 6848 | 6464 | 6419 |
| C81006 | 0.706 | 1.19 | 1.18 | 6849 | 7198 | 7190 |
| F84734 | 0.705 | 0.92 | 0.91 | 6850 | 6076 | 6004 |
| H84002 | 0.705 | 0.88 | 0.87 | 6851 | 5701 | 5560 |
| E82603 | 0.705 | 1.05 | 1.02 | 6852 | 6904 | 6811 |
| F84115 | 0.705 | 0.96 | 0.97 | 6853 | 6384 | 6497 |
| H85088 | 0.705 | 0.90 | 0.88 | 6854 | 5871 | 5705 |
| M83107 | 0.705 | 1.78 | 1.82 | 6855 | 7311 | 7311 |
| L83012 | 0.704 | 1.12 | 1.12 | 6856 | 7123 | 7113 |
| M87617 | 0.704 | 1.18 | 1.17 | 6857 | 7189 | 7184 |
| E84031 | 0.704 | 1.06 | 1.05 | 6858 | 6960 | 6908 |
| G85712 | 0.704 | 0.90 | 0.90 | 6859 | 5832 | 5879 |
| E83656 | 0.704 | 0.97 | 0.96 | 6860 | 6540 | 6401 |
| F83683 | 0.704 | 1.11 | 1.10 | 6861 | 7098 | 7074 |
| E87681 | 0.704 | 0.87 | 0.87 | 6862 | 5613 | 5578 |
| N83619 | 0.704 | 1.17 | 1.19 | 6863 | 7188 | 7202 |
| E83658 | 0.704 | 0.91 | 0.89 | 6864 | 5970 | 5799 |
| F84081 | 0.703 | 0.99 | 0.99 | 6865 | 6605 | 6634 |
| G85095 | 0.702 | 0.97 | 0.97 | 6866 | 6543 | 6536 |
| F83632 | 0.702 | 0.97 | 0.96 | 6867 | 6517 | 6457 |
| E85715 | 0.702 | 0.96 | 0.97 | 6868 | 6470 | 6518 |
| N81090 | 0.702 | 1.06 | 1.05 | 6869 | 6983 | 6931 |
| M81007 | 0.701 | 1.04 | 1.02 | 6870 | 6872 | 6833 |
| M85021 | 0.701 | 0.92 | 0.92 | 6871 | 6127 | 6089 |
| G85084 | 0.701 | 0.93 | 0.94 | 6872 | 6210 | 6259 |
| F83063 | 0.700 | 0.97 | 0.98 | 6873 | 6511 | 6579 |
| F83032 | 0.700 | 0.91 | 0.92 | 6874 | 6021 | 6145 |
| Y00316 | 0.699 | 0.93 | 0.92 | 6875 | 6230 | 6083 |
| J81072 | 0.699 | 0.96 | 0.96 | 6876 | 6452 | 6491 |
| F85024 | 0.698 | 1.00 | 0.99 | 6877 | 6722 | 6675 |
| J82050 | 0.698 | 0.93 | 0.91 | 6878 | 6183 | 6001 |
| F83010 | 0.697 | 0.90 | 0.91 | 6879 | 5885 | 6037 |
| M92041 | 0.697 | 1.07 | 1.08 | 6880 | 7008 | 7033 |
| G85038 | 0.697 | 0.95 | 0.96 | 6881 | 6368 | 6470 |
| E87648 | 0.697 | 0.89 | 0.88 | 6882 | 5807 | 5784 |
| C82060 | 0.697 | 1.11 | 1.12 | 6883 | 7119 | 7134 |
| E85033 | 0.696 | 0.95 | 0.96 | 6884 | 6371 | 6501 |
| C88079 | 0.696 | 0.95 | 0.97 | 6885 | 6440 | 6586 |
| P85614 | 0.696 | 1.25 | 1.26 | 6886 | 7256 | 7262 |
| M85058 | 0.696 | 0.96 | 0.95 | 6887 | 6469 | 6404 |
| E87029 | 0.696 | 0.91 | 0.90 | 6888 | 6033 | 5983 |
| K84054 | 0.695 | 1.00 | 0.97 | 6889 | 6748 | 6593 |
| Y02476 | 0.695 | 0.78 | 0.79 | 6890 | 4636 | 4752 |
| J82071 | 0.692 | 1.03 | 1.02 | 6891 | 6866 | 6831 |
| F84013 | 0.691 | 0.87 | 0.88 | 6892 | 5682 | 5865 |
| E87714 | 0.691 | 0.91 | 0.90 | 6893 | 6075 | 6000 |

| | | | | | | |
|---|---|---|---|---|---|---|
| F83048 | 0.691 | 0.90 | 0.89 | 6894 | 5994 | 5964 |
| C81652 | 0.691 | 1.05 | 1.04 | 6895 | 6953 | 6945 |
| G85100 | 0.691 | 0.91 | 0.92 | 6896 | 6105 | 6212 |
| H81007 | 0.690 | 0.92 | 0.91 | 6897 | 6186 | 6094 |
| E85630 | 0.690 | 0.93 | 0.93 | 6898 | 6256 | 6306 |
| B81675 | 0.690 | 1.10 | 1.15 | 6899 | 7110 | 7171 |
| M85060 | 0.690 | 1.17 | 1.16 | 6900 | 7195 | 7182 |
| L82041 | 0.689 | 1.11 | 1.11 | 6901 | 7120 | 7123 |
| F81176 | 0.689 | 1.18 | 1.19 | 6902 | 7204 | 7212 |
| M85774 | 0.689 | 1.03 | 1.03 | 6903 | 6918 | 6915 |
| H82057 | 0.689 | 1.01 | 1.01 | 6904 | 6817 | 6825 |
| H85634 | 0.688 | 0.87 | 0.86 | 6905 | 5751 | 5606 |
| F86698 | 0.687 | 1.07 | 1.05 | 6906 | 7042 | 6983 |
| C81653 | 0.687 | 1.02 | 1.03 | 6907 | 6876 | 6902 |
| D81005 | 0.687 | 0.80 | 0.78 | 6908 | 4919 | 4704 |
| P87613 | 0.687 | 1.28 | 1.29 | 6909 | 7268 | 7271 |
| K81100 | 0.687 | 0.84 | 0.84 | 6910 | 5413 | 5490 |
| Y00589 | 0.686 | 1.06 | 1.06 | 6911 | 7020 | 7003 |
| F84069 | 0.685 | 0.99 | 1.00 | 6912 | 6705 | 6764 |
| M91017 | 0.685 | 1.30 | 1.32 | 6913 | 7278 | 7285 |
| G82693 | 0.685 | 1.09 | 1.11 | 6914 | 7097 | 7121 |
| M88647 | 0.685 | 1.05 | 1.07 | 6915 | 7001 | 7041 |
| G85724 | 0.685 | 0.81 | 0.83 | 6916 | 5146 | 5287 |
| G85054 | 0.685 | 1.02 | 1.03 | 6917 | 6883 | 6914 |
| E85025 | 0.685 | 0.89 | 0.91 | 6918 | 5929 | 6141 |
| E87682 | 0.684 | 0.88 | 0.88 | 6919 | 5898 | 5896 |
| H84044 | 0.684 | 0.93 | 0.91 | 6920 | 6341 | 6188 |
| F84706 | 0.683 | 0.97 | 0.96 | 6921 | 6607 | 6560 |
| J82044 | 0.683 | 1.06 | 1.05 | 6922 | 7022 | 6976 |
| M83723 | 0.683 | 1.21 | 1.21 | 6923 | 7234 | 7238 |
| L81090 | 0.683 | 0.87 | 0.89 | 6924 | 5812 | 5966 |
| G81070 | 0.683 | 0.94 | 0.96 | 6925 | 6444 | 6577 |
| J83619 | 0.682 | 1.04 | 1.03 | 6926 | 6959 | 6937 |
| F85014 | 0.682 | 0.87 | 0.88 | 6927 | 5794 | 5923 |
| F81666 | 0.682 | 1.08 | 1.08 | 6928 | 7083 | 7075 |
| E87050 | 0.682 | 0.96 | 0.95 | 6929 | 6560 | 6506 |
| E87021 | 0.681 | 1.01 | 1.00 | 6930 | 6843 | 6778 |
| C81009 | 0.680 | 1.16 | 1.16 | 6931 | 7201 | 7194 |
| E87009 | 0.680 | 0.97 | 0.96 | 6932 | 6658 | 6617 |
| Y00568 | 0.679 | 1.00 | 0.97 | 6933 | 6795 | 6653 |
| L81071 | 0.679 | 0.88 | 0.88 | 6934 | 5893 | 5909 |
| H83043 | 0.679 | 0.98 | 0.98 | 6935 | 6725 | 6699 |
| F85063 | 0.679 | 0.85 | 0.86 | 6936 | 5602 | 5712 |
| F85623 | 0.679 | 0.95 | 0.96 | 6937 | 6576 | 6622 |
| F82671 | 0.678 | 1.04 | 1.03 | 6938 | 6961 | 6946 |
| F83017 | 0.677 | 0.94 | 0.93 | 6939 | 6473 | 6437 |
| F84117 | 0.677 | 0.94 | 0.96 | 6940 | 6487 | 6600 |
| P91619 | 0.676 | 1.00 | 1.01 | 6941 | 6819 | 6870 |
| B82021 | 0.676 | 1.03 | 1.03 | 6942 | 6951 | 6935 |
| F84731 | 0.676 | 0.98 | 0.98 | 6943 | 6754 | 6721 |

| | | | | | | |
|---|---|---|---|---|---|---|
| M92014 | 0.675 | 1.09 | 1.11 | 6944 | 7114 | 7137 |
| E87609 | 0.675 | 0.89 | 0.89 | 6945 | 6085 | 6054 |
| K84076 | 0.675 | 0.93 | 0.93 | 6946 | 6461 | 6418 |
| E87071 | 0.675 | 0.84 | 0.83 | 6947 | 5530 | 5501 |
| G81038 | 0.675 | 0.90 | 0.92 | 6948 | 6171 | 6368 |
| E83009 | 0.675 | 0.91 | 0.89 | 6949 | 6205 | 6080 |
| H85078 | 0.674 | 1.03 | 1.01 | 6950 | 6941 | 6884 |
| E86637 | 0.674 | 1.05 | 1.04 | 6951 | 7031 | 6999 |
| L81081 | 0.674 | 0.84 | 0.85 | 6952 | 5565 | 5721 |
| H85012 | 0.674 | 0.82 | 0.80 | 6953 | 5331 | 5148 |
| G85010 | 0.673 | 0.94 | 0.95 | 6954 | 6482 | 6553 |
| C84112 | 0.673 | 1.11 | 1.11 | 6955 | 7150 | 7143 |
| P84072 | 0.672 | 0.90 | 0.92 | 6956 | 6141 | 6352 |
| K84009 | 0.672 | 0.93 | 0.92 | 6957 | 6424 | 6351 |
| E85693 | 0.671 | 0.87 | 0.86 | 6958 | 5854 | 5833 |
| E83654 | 0.671 | 1.00 | 0.99 | 6959 | 6831 | 6790 |
| D81013 | 0.668 | 0.84 | 0.82 | 6960 | 5606 | 5375 |
| F84749 | 0.668 | 0.90 | 0.89 | 6961 | 6234 | 6177 |
| F82027 | 0.668 | 1.05 | 1.06 | 6962 | 7028 | 7053 |
| H85066 | 0.667 | 0.84 | 0.83 | 6963 | 5627 | 5504 |
| A82654 | 0.667 | 1.17 | 1.18 | 6964 | 7217 | 7233 |
| L82015 | 0.666 | 1.10 | 1.11 | 6965 | 7149 | 7154 |
| F83003 | 0.666 | 0.88 | 0.88 | 6966 | 6093 | 6082 |
| L83078 | 0.665 | 1.27 | 1.27 | 6967 | 7275 | 7272 |
| J82081 | 0.665 | 0.85 | 0.87 | 6968 | 5792 | 5953 |
| F82055 | 0.663 | 1.00 | 0.99 | 6969 | 6864 | 6867 |
| F85669 | 0.663 | 0.90 | 0.91 | 6970 | 6233 | 6331 |
| G82702 | 0.663 | 1.02 | 1.03 | 6971 | 6940 | 6985 |
| F81192 | 0.662 | 1.16 | 1.15 | 6972 | 7209 | 7208 |
| M83017 | 0.662 | 1.34 | 1.33 | 6973 | 7297 | 7296 |
| M85010 | 0.661 | 1.28 | 1.28 | 6974 | 7283 | 7277 |
| G83680 | 0.661 | 0.96 | 0.96 | 6975 | 6701 | 6694 |
| C84080 | 0.661 | 1.04 | 1.04 | 6976 | 7034 | 7035 |
| M85797 | 0.660 | 0.94 | 0.93 | 6977 | 6577 | 6540 |
| B83043 | 0.660 | 1.00 | 1.01 | 6978 | 6900 | 6953 |
| G81638 | 0.659 | 0.86 | 0.88 | 6979 | 5962 | 6161 |
| F84601 | 0.659 | 0.96 | 0.97 | 6980 | 6712 | 6776 |
| E84048 | 0.659 | 1.05 | 1.04 | 6981 | 7073 | 7029 |
| E85680 | 0.659 | 0.91 | 0.91 | 6982 | 6394 | 6425 |
| F83677 | 0.658 | 0.90 | 0.89 | 6983 | 6283 | 6249 |
| E85071 | 0.658 | 1.10 | 1.09 | 6984 | 7161 | 7132 |
| F81015 | 0.658 | 1.03 | 0.99 | 6985 | 7003 | 6886 |
| F83042 | 0.657 | 0.92 | 0.92 | 6986 | 6491 | 6463 |
| G85004 | 0.656 | 1.02 | 1.02 | 6987 | 6971 | 6996 |
| E85748 | 0.655 | 0.80 | 0.83 | 6988 | 5403 | 5608 |
| E82656 | 0.655 | 0.90 | 0.88 | 6989 | 6330 | 6189 |
| G85633 | 0.655 | 0.99 | 1.00 | 6990 | 6896 | 6936 |
| M85611 | 0.655 | 0.95 | 0.94 | 6991 | 6648 | 6623 |
| M85085 | 0.654 | 0.95 | 0.95 | 6992 | 6689 | 6687 |
| K81081 | 0.654 | 0.91 | 0.91 | 6993 | 6413 | 6460 |

| | | | | | | |
|---|---|---|---|---|---|---|
| H81038 | 0.654 | 0.87 | 0.86 | 6994 | 6047 | 5997 |
| H83044 | 0.654 | 0.91 | 0.91 | 6995 | 6460 | 6433 |
| G82662 | 0.652 | 0.92 | 0.93 | 6996 | 6501 | 6599 |
| G82175 | 0.652 | 1.03 | 1.05 | 6997 | 7038 | 7064 |
| J82629 | 0.651 | 1.01 | 1.00 | 6998 | 6982 | 6924 |
| H81061 | 0.651 | 0.93 | 0.94 | 6999 | 6602 | 6647 |
| L82007 | 0.651 | 1.13 | 1.14 | 7000 | 7200 | 7203 |
| F85064 | 0.650 | 0.96 | 0.97 | 7001 | 6765 | 6828 |
| Y00507 | 0.649 | 0.99 | 0.98 | 7002 | 6885 | 6852 |
| G85130 | 0.649 | 0.94 | 0.95 | 7003 | 6673 | 6717 |
| L81637 | 0.649 | 1.01 | 1.00 | 7004 | 6977 | 6938 |
| H81647 | 0.649 | 0.86 | 0.84 | 7005 | 6028 | 5824 |
| G81656 | 0.648 | 0.94 | 0.96 | 7006 | 6665 | 6768 |
| C84104 | 0.648 | 0.97 | 0.99 | 7007 | 6807 | 6916 |
| F84730 | 0.647 | 0.84 | 0.83 | 7008 | 5836 | 5773 |
| G82812 | 0.646 | 1.10 | 1.11 | 7009 | 7166 | 7177 |
| E84635 | 0.645 | 0.98 | 0.97 | 7010 | 6867 | 6822 |
| F85071 | 0.644 | 0.95 | 0.95 | 7011 | 6724 | 6749 |
| G85632 | 0.644 | 0.95 | 0.94 | 7012 | 6731 | 6702 |
| B86032 | 0.642 | 1.42 | 1.44 | 7013 | 7307 | 7309 |
| F83011 | 0.642 | 0.90 | 0.90 | 7014 | 6458 | 6444 |
| E84709 | 0.641 | 0.96 | 0.94 | 7015 | 6782 | 6726 |
| M85178 | 0.641 | 1.46 | 1.44 | 7016 | 7309 | 7308 |
| E85625 | 0.641 | 0.90 | 0.90 | 7017 | 6479 | 6442 |
| E83621 | 0.640 | 1.00 | 0.98 | 7018 | 6963 | 6889 |
| F83034 | 0.640 | 1.02 | 1.02 | 7019 | 7032 | 7040 |
| H84061 | 0.640 | 0.86 | 0.85 | 7020 | 6116 | 6031 |
| C84664 | 0.639 | 0.99 | 1.02 | 7021 | 6949 | 7025 |
| B82026 | 0.639 | 1.13 | 1.12 | 7022 | 7206 | 7200 |
| F85031 | 0.638 | 0.92 | 0.92 | 7023 | 6584 | 6625 |
| F83019 | 0.638 | 0.99 | 0.98 | 7024 | 6931 | 6903 |
| F84035 | 0.637 | 1.00 | 1.02 | 7025 | 6996 | 7023 |
| E83644 | 0.637 | 1.15 | 1.12 | 7026 | 7225 | 7199 |
| M85063 | 0.637 | 1.09 | 1.07 | 7027 | 7164 | 7150 |
| N84010 | 0.636 | 1.08 | 1.08 | 7028 | 7154 | 7158 |
| F83664 | 0.636 | 0.94 | 0.95 | 7029 | 6733 | 6784 |
| K84026 | 0.634 | 0.88 | 0.88 | 7030 | 6364 | 6336 |
| H85069 | 0.634 | 0.86 | 0.85 | 7031 | 6180 | 6044 |
| F84030 | 0.634 | 1.00 | 1.00 | 7032 | 6995 | 6987 |
| E84036 | 0.633 | 0.99 | 0.97 | 7033 | 6950 | 6900 |
| L81112 | 0.633 | 0.88 | 0.89 | 7034 | 6350 | 6477 |
| Y00080 | 0.632 | 1.01 | 1.02 | 7035 | 7021 | 7058 |
| Y02686 | 0.632 | 0.88 | 0.89 | 7036 | 6383 | 6471 |
| B82027 | 0.632 | 1.03 | 1.03 | 7037 | 7076 | 7067 |
| G85138 | 0.631 | 0.93 | 0.93 | 7038 | 6717 | 6718 |
| H85664 | 0.631 | 0.78 | 0.77 | 7039 | 5430 | 5302 |
| M92012 | 0.631 | 1.15 | 1.17 | 7040 | 7231 | 7251 |
| E84667 | 0.631 | 0.99 | 0.97 | 7041 | 6966 | 6912 |
| D81001 | 0.630 | 0.86 | 0.84 | 7042 | 6187 | 6012 |
| K84080 | 0.630 | 0.87 | 0.87 | 7043 | 6287 | 6262 |

| | | | | | | |
|---|---|---|---|---|---|---|
| D81054 | 0.630 | 0.82 | 0.80 | 7044 | 5851 | 5639 |
| F83665 | 0.630 | 0.84 | 0.84 | 7045 | 6026 | 6033 |
| F83055 | 0.629 | 0.89 | 0.89 | 7046 | 6447 | 6443 |
| M85721 | 0.629 | 0.88 | 0.88 | 7047 | 6408 | 6390 |
| H82037 | 0.629 | 1.37 | 1.37 | 7048 | 7305 | 7305 |
| K84011 | 0.627 | 0.90 | 0.89 | 7049 | 6541 | 6510 |
| M85088 | 0.627 | 1.02 | 1.01 | 7050 | 7061 | 7039 |
| H83017 | 0.627 | 0.99 | 0.98 | 7051 | 6979 | 6950 |
| Y02721 | 0.626 | 1.04 | 1.06 | 7052 | 7122 | 7138 |
| E83632 | 0.626 | 1.07 | 1.04 | 7053 | 7160 | 7104 |
| G85031 | 0.625 | 0.95 | 0.96 | 7054 | 6844 | 6862 |
| F84063 | 0.623 | 0.96 | 0.97 | 7055 | 6868 | 6930 |
| F82009 | 0.623 | 1.06 | 1.06 | 7056 | 7157 | 7157 |
| E84051 | 0.623 | 1.04 | 1.03 | 7057 | 7124 | 7093 |
| E87753 | 0.622 | 0.93 | 0.93 | 7058 | 6761 | 6731 |
| E85091 | 0.622 | 0.90 | 0.90 | 7059 | 6580 | 6610 |
| F83686 | 0.621 | 0.89 | 0.91 | 7060 | 6532 | 6629 |
| E85628 | 0.621 | 0.92 | 0.93 | 7061 | 6714 | 6747 |
| E87010 | 0.621 | 0.88 | 0.87 | 7062 | 6462 | 6412 |
| H81006 | 0.621 | 0.94 | 0.91 | 7063 | 6814 | 6657 |
| F84647 | 0.621 | 0.87 | 0.87 | 7064 | 6362 | 6379 |
| E85038 | 0.620 | 0.89 | 0.91 | 7065 | 6507 | 6643 |
| E85062 | 0.619 | 0.87 | 0.86 | 7066 | 6369 | 6339 |
| G83065 | 0.618 | 0.84 | 0.85 | 7067 | 6169 | 6194 |
| H85075 | 0.618 | 0.89 | 0.88 | 7068 | 6583 | 6475 |
| K81655 | 0.617 | 1.05 | 1.03 | 7069 | 7142 | 7108 |
| F85013 | 0.617 | 0.91 | 0.92 | 7070 | 6687 | 6741 |
| E85066 | 0.615 | 0.86 | 0.87 | 7071 | 6358 | 6417 |
| G81044 | 0.614 | 0.90 | 0.92 | 7072 | 6619 | 6735 |
| G82697 | 0.613 | 1.10 | 1.12 | 7073 | 7202 | 7219 |
| E86619 | 0.613 | 0.97 | 0.97 | 7074 | 6965 | 6947 |
| F84123 | 0.613 | 0.83 | 0.84 | 7075 | 6107 | 6144 |
| E87005 | 0.613 | 0.91 | 0.90 | 7076 | 6670 | 6651 |
| B81091 | 0.612 | 1.00 | 1.03 | 7077 | 7059 | 7120 |
| M86029 | 0.612 | 0.86 | 0.86 | 7078 | 6356 | 6347 |
| G85105 | 0.612 | 0.89 | 0.90 | 7079 | 6562 | 6637 |
| Y02957 | 0.612 | 0.76 | 0.77 | 7080 | 5408 | 5537 |
| F82642 | 0.611 | 1.15 | 1.15 | 7081 | 7246 | 7248 |
| C84117 | 0.611 | 0.77 | 0.79 | 7082 | 5465 | 5704 |
| E83005 | 0.611 | 0.93 | 0.91 | 7083 | 6813 | 6734 |
| Y00449 | 0.611 | 0.86 | 0.89 | 7084 | 6347 | 6572 |
| F84036 | 0.610 | 0.97 | 0.98 | 7085 | 6970 | 7010 |
| E84705 | 0.608 | 0.90 | 0.89 | 7086 | 6688 | 6618 |
| E87701 | 0.608 | 0.85 | 0.85 | 7087 | 6337 | 6334 |
| Y02672 | 0.608 | 0.85 | 0.85 | 7088 | 6346 | 6321 |
| Y03506 | 0.607 | 0.90 | 0.91 | 7089 | 6685 | 6740 |
| K81605 | 0.606 | 0.76 | 0.77 | 7090 | 5475 | 5562 |
| B81032 | 0.606 | 1.07 | 1.11 | 7091 | 7180 | 7217 |
| A83011 | 0.606 | 0.85 | 0.86 | 7092 | 6294 | 6458 |
| G85044 | 0.606 | 0.98 | 0.99 | 7093 | 7029 | 7048 |

| | | | | | | |
|---|---|---|---|---|---|---|
| F84718 | 0.605 | 0.97 | 0.97 | 7094 | 6978 | 6973 |
| E85005 | 0.604 | 0.96 | 0.98 | 7095 | 6943 | 7009 |
| F84074 | 0.604 | 1.15 | 1.13 | 7096 | 7251 | 7241 |
| E87004 | 0.603 | 0.89 | 0.89 | 7097 | 6630 | 6612 |
| E87061 | 0.603 | 0.80 | 0.80 | 7098 | 5899 | 5927 |
| F84124 | 0.602 | 0.87 | 0.86 | 7099 | 6498 | 6445 |
| E84674 | 0.601 | 0.87 | 0.86 | 7100 | 6509 | 6435 |
| E84669 | 0.601 | 1.03 | 1.02 | 7101 | 7144 | 7115 |
| P84683 | 0.601 | 0.83 | 0.85 | 7102 | 6160 | 6385 |
| H85027 | 0.601 | 0.85 | 0.85 | 7103 | 6392 | 6335 |
| E85719 | 0.600 | 0.83 | 0.86 | 7104 | 6216 | 6427 |
| Y02671 | 0.600 | 0.82 | 0.82 | 7105 | 6113 | 6086 |
| B86108 | 0.600 | 1.02 | 1.04 | 7106 | 7117 | 7149 |
| E85694 | 0.600 | 0.91 | 0.91 | 7107 | 6749 | 6773 |
| F86626 | 0.599 | 0.83 | 0.85 | 7108 | 6201 | 6395 |
| F85061 | 0.599 | 0.87 | 0.88 | 7109 | 6518 | 6583 |
| M85002 | 0.598 | 0.93 | 0.93 | 7110 | 6856 | 6851 |
| F84686 | 0.597 | 0.81 | 0.83 | 7111 | 6063 | 6228 |
| F85023 | 0.597 | 1.14 | 1.12 | 7112 | 7247 | 7240 |
| F85700 | 0.596 | 0.90 | 0.90 | 7113 | 6727 | 6727 |
| C84605 | 0.595 | 1.01 | 1.04 | 7114 | 7121 | 7160 |
| J81645 | 0.595 | 0.92 | 0.93 | 7115 | 6852 | 6871 |
| F84631 | 0.595 | 0.96 | 0.94 | 7116 | 6976 | 6932 |
| G85025 | 0.594 | 0.91 | 0.92 | 7117 | 6774 | 6830 |
| G85647 | 0.593 | 0.87 | 0.88 | 7118 | 6593 | 6671 |
| E87013 | 0.593 | 0.88 | 0.88 | 7119 | 6652 | 6642 |
| F84656 | 0.592 | 0.88 | 0.88 | 7120 | 6631 | 6644 |
| E83013 | 0.592 | 0.92 | 0.90 | 7121 | 6834 | 6761 |
| F83059 | 0.592 | 0.93 | 0.92 | 7122 | 6899 | 6834 |
| C84151 | 0.591 | 0.98 | 1.00 | 7123 | 7052 | 7094 |
| P81038 | 0.590 | 1.18 | 1.14 | 7124 | 7267 | 7256 |
| F82010 | 0.590 | 1.19 | 1.17 | 7125 | 7274 | 7267 |
| F84055 | 0.587 | 0.90 | 0.90 | 7126 | 6759 | 6763 |
| Y02471 | 0.586 | 0.90 | 0.93 | 7127 | 6797 | 6913 |
| G85023 | 0.586 | 0.94 | 0.96 | 7128 | 6964 | 6993 |
| F83623 | 0.586 | 0.87 | 0.87 | 7129 | 6591 | 6597 |
| F86088 | 0.586 | 1.08 | 1.10 | 7130 | 7211 | 7227 |
| C83053 | 0.584 | 1.08 | 1.07 | 7131 | 7216 | 7201 |
| E87007 | 0.584 | 0.82 | 0.82 | 7132 | 6289 | 6284 |
| G85094 | 0.584 | 0.93 | 0.93 | 7133 | 6909 | 6910 |
| F83057 | 0.584 | 0.93 | 0.93 | 7134 | 6929 | 6909 |
| F85628 | 0.584 | 1.03 | 1.03 | 7135 | 7156 | 7163 |
| E83637 | 0.583 | 0.90 | 0.88 | 7136 | 6770 | 6698 |
| Y00200 | 0.583 | 0.84 | 0.84 | 7137 | 6451 | 6441 |
| C84101 | 0.583 | 1.11 | 1.13 | 7138 | 7236 | 7253 |
| E84006 | 0.580 | 1.06 | 1.04 | 7139 | 7192 | 7173 |
| C84013 | 0.578 | 1.19 | 1.19 | 7140 | 7279 | 7274 |
| Y02987 | 0.576 | 0.97 | 0.96 | 7141 | 7074 | 7032 |
| L81040 | 0.574 | 1.11 | 1.09 | 7142 | 7245 | 7231 |
| L84054 | 0.574 | 1.09 | 1.04 | 7143 | 7228 | 7187 |

| | | | | | | |
|---|---|---|---|---|---|---|
| F84742 | 0.574 | 0.93 | 0.92 | 7144 | 6972 | 6940 |
| F84729 | 0.573 | 1.03 | 1.01 | 7145 | 7170 | 7155 |
| Y03441 | 0.573 | 0.79 | 0.79 | 7146 | 6072 | 6066 |
| B83660 | 0.573 | 1.18 | 1.20 | 7147 | 7276 | 7280 |
| P81011 | 0.573 | 1.07 | 1.08 | 7148 | 7214 | 7226 |
| Y04664 | 0.572 | 0.99 | 0.98 | 7149 | 7129 | 7102 |
| P81155 | 0.570 | 1.22 | 1.24 | 7150 | 7293 | 7294 |
| K84605 | 0.569 | 0.77 | 0.77 | 7151 | 5879 | 5916 |
| C84114 | 0.569 | 1.19 | 1.20 | 7152 | 7280 | 7287 |
| L81643 | 0.568 | 1.11 | 1.09 | 7153 | 7249 | 7234 |
| K84621 | 0.566 | 0.93 | 0.93 | 7154 | 6988 | 6978 |
| D83073 | 0.566 | 1.07 | 1.07 | 7155 | 7219 | 7220 |
| H85020 | 0.566 | 1.14 | 1.12 | 7156 | 7264 | 7255 |
| E85677 | 0.566 | 0.87 | 0.87 | 7157 | 6728 | 6752 |
| B85062 | 0.564 | 0.74 | 0.76 | 7158 | 5715 | 5834 |
| E86034 | 0.564 | 1.01 | 1.00 | 7159 | 7163 | 7153 |
| E86618 | 0.564 | 1.00 | 0.99 | 7160 | 7151 | 7136 |
| M92035 | 0.563 | 1.00 | 1.02 | 7161 | 7152 | 7172 |
| B86068 | 0.563 | 1.04 | 1.06 | 7162 | 7197 | 7215 |
| M85100 | 0.563 | 1.09 | 1.08 | 7163 | 7237 | 7230 |
| G85674 | 0.562 | 0.83 | 0.84 | 7164 | 6499 | 6585 |
| K84016 | 0.562 | 0.83 | 0.83 | 7165 | 6529 | 6513 |
| M91026 | 0.561 | 1.31 | 1.31 | 7166 | 7306 | 7306 |
| Y03135 | 0.561 | 0.89 | 0.90 | 7167 | 6862 | 6907 |
| F84114 | 0.560 | 0.90 | 0.90 | 7168 | 6903 | 6920 |
| K84030 | 0.559 | 1.05 | 1.02 | 7169 | 7203 | 7176 |
| L81015 | 0.557 | 1.15 | 1.16 | 7170 | 7270 | 7270 |
| B82098 | 0.555 | 0.98 | 0.97 | 7171 | 7127 | 7114 |
| G85711 | 0.554 | 0.90 | 0.91 | 7172 | 6933 | 6965 |
| F84740 | 0.554 | 0.85 | 0.84 | 7173 | 6655 | 6616 |
| Y01962 | 0.554 | 0.78 | 0.79 | 7174 | 6148 | 6283 |
| M83670 | 0.553 | 0.77 | 0.78 | 7175 | 6041 | 6210 |
| G85014 | 0.553 | 0.98 | 0.99 | 7176 | 7131 | 7144 |
| G84041 | 0.552 | 0.97 | 0.97 | 7177 | 7125 | 7118 |
| E87741 | 0.549 | 0.91 | 0.91 | 7178 | 6991 | 6967 |
| E84665 | 0.548 | 0.96 | 0.95 | 7179 | 7113 | 7080 |
| E85074 | 0.548 | 0.87 | 0.89 | 7180 | 6802 | 6901 |
| H84020 | 0.547 | 0.81 | 0.80 | 7181 | 6476 | 6411 |
| P81093 | 0.547 | 1.05 | 1.03 | 7182 | 7218 | 7204 |
| E84647 | 0.546 | 1.05 | 1.04 | 7183 | 7220 | 7207 |
| E84086 | 0.546 | 0.85 | 0.84 | 7184 | 6726 | 6683 |
| K84021 | 0.545 | 0.85 | 0.84 | 7185 | 6708 | 6704 |
| Y01719 | 0.544 | 0.93 | 0.95 | 7186 | 7054 | 7079 |
| P84630 | 0.544 | 0.87 | 0.90 | 7187 | 6853 | 6948 |
| N82059 | 0.539 | 0.89 | 0.92 | 7188 | 6944 | 7021 |
| E87063 | 0.538 | 0.83 | 0.83 | 7189 | 6659 | 6659 |
| Y02589 | 0.537 | 0.81 | 0.83 | 7190 | 6574 | 6692 |
| J82024 | 0.536 | 0.93 | 0.94 | 7191 | 7065 | 7099 |
| E87720 | 0.536 | 0.84 | 0.84 | 7192 | 6735 | 6738 |
| E85692 | 0.535 | 0.87 | 0.87 | 7193 | 6874 | 6860 |

| | | | | | | |
|---|---|---|---|---|---|---|
| E87742 | 0.533 | 1.06 | 1.05 | 7194 | 7232 | 7228 |
| Y02847 | 0.533 | 0.83 | 0.85 | 7195 | 6682 | 6809 |
| F86044 | 0.532 | 0.99 | 1.01 | 7196 | 7173 | 7198 |
| G85047 | 0.531 | 0.92 | 0.93 | 7197 | 7055 | 7073 |
| F84660 | 0.531 | 0.96 | 0.94 | 7198 | 7135 | 7106 |
| G81034 | 0.531 | 0.92 | 0.94 | 7199 | 7045 | 7090 |
| M83708 | 0.531 | 1.22 | 1.26 | 7200 | 7299 | 7303 |
| E85673 | 0.530 | 0.98 | 1.00 | 7201 | 7165 | 7189 |
| P81064 | 0.529 | 0.85 | 0.86 | 7202 | 6796 | 6876 |
| F83633 | 0.524 | 0.87 | 0.87 | 7203 | 6912 | 6911 |
| D81037 | 0.523 | 0.79 | 0.77 | 7204 | 6496 | 6346 |
| D83046 | 0.522 | 1.02 | 1.03 | 7205 | 7215 | 7225 |
| B82103 | 0.522 | 1.07 | 1.06 | 7206 | 7250 | 7249 |
| E87046 | 0.521 | 0.82 | 0.82 | 7207 | 6723 | 6710 |
| H84005 | 0.519 | 0.87 | 0.85 | 7208 | 6923 | 6861 |
| F85067 | 0.518 | 1.00 | 1.01 | 7209 | 7196 | 7210 |
| F81141 | 0.517 | 0.80 | 0.81 | 7210 | 6603 | 6667 |
| Y01655 | 0.517 | 0.81 | 0.82 | 7211 | 6672 | 6743 |
| B81118 | 0.516 | 1.11 | 1.14 | 7212 | 7272 | 7279 |
| L81133 | 0.512 | 0.72 | 0.74 | 7213 | 6024 | 6176 |
| J82055 | 0.512 | 1.02 | 1.04 | 7214 | 7227 | 7242 |
| G85721 | 0.511 | 0.91 | 0.91 | 7215 | 7069 | 7068 |
| E84049 | 0.511 | 1.14 | 1.12 | 7216 | 7286 | 7275 |
| E85725 | 0.508 | 1.02 | 1.02 | 7217 | 7222 | 7229 |
| G85020 | 0.507 | 0.85 | 0.86 | 7218 | 6913 | 6958 |
| C82105 | 0.506 | 0.97 | 0.98 | 7219 | 7185 | 7193 |
| E84066 | 0.505 | 1.01 | 1.00 | 7220 | 7221 | 7209 |
| F84072 | 0.505 | 0.83 | 0.85 | 7221 | 6830 | 6904 |
| E87702 | 0.504 | 0.88 | 0.88 | 7222 | 7011 | 7004 |
| E87665 | 0.503 | 0.88 | 0.87 | 7223 | 7023 | 7006 |
| F85034 | 0.501 | 0.99 | 1.00 | 7224 | 7205 | 7213 |
| C82124 | 0.500 | 0.74 | 0.75 | 7225 | 6271 | 6386 |
| D81026 | 0.498 | 1.14 | 1.14 | 7226 | 7289 | 7290 |
| G85091 | 0.498 | 0.90 | 0.90 | 7227 | 7084 | 7087 |
| E84701 | 0.497 | 0.96 | 0.95 | 7228 | 7179 | 7167 |
| G85715 | 0.496 | 0.87 | 0.87 | 7229 | 7019 | 7022 |
| G85135 | 0.494 | 1.22 | 1.22 | 7230 | 7304 | 7304 |
| M82042 | 0.493 | 1.11 | 1.12 | 7231 | 7282 | 7284 |
| E87745 | 0.491 | 0.86 | 0.86 | 7232 | 7004 | 6980 |
| G81646 | 0.490 | 0.92 | 0.94 | 7233 | 7141 | 7166 |
| F83635 | 0.490 | 1.01 | 1.01 | 7234 | 7233 | 7232 |
| F84635 | 0.488 | 0.90 | 0.91 | 7235 | 7102 | 7124 |
| E87737 | 0.488 | 0.83 | 0.83 | 7236 | 6905 | 6894 |
| M85684 | 0.484 | 0.91 | 0.91 | 7237 | 7139 | 7130 |
| C88007 | 0.483 | 0.78 | 0.80 | 7238 | 6692 | 6792 |
| D82088 | 0.480 | 0.75 | 0.77 | 7239 | 6550 | 6669 |
| M85118 | 0.480 | 1.04 | 1.04 | 7240 | 7259 | 7259 |
| P84605 | 0.479 | 1.07 | 1.09 | 7241 | 7269 | 7273 |
| C82020 | 0.478 | 0.78 | 0.79 | 7242 | 6732 | 6779 |
| E87637 | 0.478 | 1.01 | 1.00 | 7243 | 7243 | 7236 |

| | | | | | | |
|---|---|---|---|---|---|---|
| F84636 | 0.475 | 0.90 | 0.92 | 7244 | 7138 | 7161 |
| G85137 | 0.472 | 0.86 | 0.87 | 7245 | 7039 | 7061 |
| E87715 | 0.472 | 0.84 | 0.83 | 7246 | 6989 | 6975 |
| F83005 | 0.471 | 0.72 | 0.72 | 7247 | 6386 | 6409 |
| L81086 | 0.471 | 1.04 | 1.02 | 7248 | 7263 | 7254 |
| B82047 | 0.470 | 0.80 | 0.80 | 7249 | 6845 | 6853 |
| P84068 | 0.469 | 1.00 | 1.03 | 7250 | 7242 | 7261 |
| G81013 | 0.468 | 0.99 | 1.02 | 7251 | 7230 | 7258 |
| H84023 | 0.466 | 0.85 | 0.84 | 7252 | 7049 | 7002 |
| C81629 | 0.466 | 1.10 | 1.11 | 7253 | 7287 | 7289 |
| F83058 | 0.465 | 0.79 | 0.79 | 7254 | 6849 | 6854 |
| E83622 | 0.463 | 0.93 | 0.91 | 7255 | 7178 | 7165 |
| E85746 | 0.462 | 0.87 | 0.86 | 7256 | 7094 | 7071 |
| E84025 | 0.462 | 0.88 | 0.87 | 7257 | 7116 | 7092 |
| P92607 | 0.461 | 1.15 | 1.16 | 7258 | 7300 | 7302 |
| E84637 | 0.461 | 0.94 | 0.93 | 7259 | 7194 | 7180 |
| Y02622 | 0.460 | 0.76 | 0.79 | 7260 | 6707 | 6873 |
| E85013 | 0.455 | 0.92 | 0.93 | 7261 | 7175 | 7188 |
| E87066 | 0.453 | 0.86 | 0.86 | 7262 | 7104 | 7095 |
| Y01929 | 0.451 | 1.10 | 1.11 | 7263 | 7291 | 7291 |
| N82117 | 0.449 | 0.79 | 0.82 | 7264 | 6916 | 6989 |
| K84013 | 0.449 | 0.80 | 0.80 | 7265 | 6927 | 6926 |
| H84639 | 0.448 | 0.78 | 0.77 | 7266 | 6879 | 6836 |
| E84656 | 0.448 | 1.08 | 1.06 | 7267 | 7284 | 7276 |
| L83616 | 0.447 | 1.11 | 1.13 | 7268 | 7295 | 7298 |
| F85640 | 0.447 | 0.92 | 0.92 | 7269 | 7186 | 7196 |
| M85167 | 0.445 | 0.74 | 0.74 | 7270 | 6690 | 6705 |
| E84078 | 0.444 | 1.16 | 1.14 | 7271 | 7303 | 7301 |
| F83061 | 0.441 | 0.91 | 0.91 | 7272 | 7190 | 7185 |
| K84078 | 0.441 | 0.76 | 0.76 | 7273 | 6805 | 6789 |
| G85127 | 0.441 | 0.91 | 0.92 | 7274 | 7182 | 7191 |
| F86704 | 0.438 | 0.93 | 0.92 | 7275 | 7207 | 7197 |
| F84025 | 0.438 | 1.06 | 1.06 | 7276 | 7281 | 7278 |
| M85680 | 0.438 | 0.91 | 0.91 | 7277 | 7191 | 7186 |
| L81622 | 0.437 | 0.88 | 0.89 | 7278 | 7153 | 7168 |
| F84694 | 0.432 | 0.98 | 1.00 | 7279 | 7253 | 7264 |
| E86030 | 0.425 | 0.74 | 0.75 | 7280 | 6810 | 6821 |
| Y02002 | 0.423 | 0.74 | 0.76 | 7281 | 6798 | 6893 |
| E84063 | 0.423 | 0.86 | 0.85 | 7282 | 7145 | 7129 |
| F86701 | 0.419 | 0.96 | 0.98 | 7283 | 7252 | 7263 |
| Y00492 | 0.418 | 0.86 | 0.86 | 7284 | 7159 | 7151 |
| F84692 | 0.418 | 0.91 | 0.92 | 7285 | 7208 | 7221 |
| G85040 | 0.414 | 0.99 | 0.99 | 7286 | 7265 | 7266 |
| B86110 | 0.407 | 0.72 | 0.74 | 7287 | 6752 | 6857 |
| C84023 | 0.403 | 0.73 | 0.75 | 7288 | 6822 | 6923 |
| Y02747 | 0.402 | 0.88 | 0.93 | 7289 | 7199 | 7239 |
| E87048 | 0.401 | 0.82 | 0.81 | 7290 | 7112 | 7105 |
| J82199 | 0.399 | 0.72 | 0.74 | 7291 | 6812 | 6891 |
| E87722 | 0.398 | 0.80 | 0.80 | 7292 | 7086 | 7076 |
| C82111 | 0.396 | 0.73 | 0.74 | 7293 | 6859 | 6917 |

| | | | | | | |
|---|---|---|---|---|---|---|
| F81708 | 0.393 | 0.89 | 0.89 | 7294 | 7212 | 7214 |
| E85124 | 0.387 | 1.03 | 1.05 | 7295 | 7294 | 7292 |
| E87069 | 0.384 | 0.85 | 0.85 | 7296 | 7184 | 7179 |
| L81617 | 0.379 | 0.74 | 0.74 | 7297 | 6956 | 6962 |
| E87043 | 0.375 | 0.82 | 0.82 | 7298 | 7158 | 7156 |
| B81104 | 0.375 | 0.80 | 0.84 | 7299 | 7130 | 7178 |
| J82080 | 0.369 | 0.74 | 0.76 | 7300 | 7002 | 7043 |
| G85076 | 0.368 | 0.78 | 0.79 | 7301 | 7100 | 7126 |
| F83050 | 0.368 | 0.94 | 0.94 | 7302 | 7262 | 7265 |
| N81624 | 0.367 | 1.07 | 1.06 | 7303 | 7302 | 7300 |
| F83043 | 0.364 | 0.72 | 0.72 | 7304 | 6947 | 6963 |
| F85705 | 0.362 | 0.91 | 0.92 | 7305 | 7254 | 7257 |
| C84714 | 0.358 | 0.74 | 0.77 | 7306 | 7044 | 7097 |
| M88044 | 0.356 | 1.02 | 1.04 | 7307 | 7296 | 7299 |
| F83044 | 0.343 | 0.99 | 0.98 | 7308 | 7292 | 7288 |
| F81042 | 0.342 | 0.97 | 0.98 | 7309 | 7285 | 7286 |
| E87070 | 0.338 | 0.88 | 0.87 | 7310 | 7248 | 7247 |
